# A Sterically Open Ruthenium-Based Photocage Activated by Red and Far-Red Light for a Wide Range of Drugs

**DOI:** 10.1021/jacs.5c14772

**Published:** 2025-11-18

**Authors:** Yurii Husiev, Sina Katharina Götzfried, Matthijs L. A. Hakkennes, Daria Kotova, Isabelle Tutein Nolthenius, Corjan van de Griend, Andrew C. Johns, Selda Abyar, Maxime A. Siegler, Alexander Kornienko, Sylvestre Bonnet

**Affiliations:** † Leiden Institute of Chemistry, 4496 Universiteit Leiden, Einsteinweg 55, 2333 CC Leiden, Netherlands; ‡ Department of Chemistry, 1466 Johns Hopkins University, 3400 N Charles St., Baltimore, Maryland 21218, United States; § Department of Chemistry and Biochemistry, 7174 Texas State University, 601 University Dr., San Marcos, Texas 78666, United States

## Abstract

Herein we report a novel ruthenium-based photocage for photoactivated chemotherapy (PACT) that can deliver a variety of experimental and clinically approved anticancer drugs using red and far-red light. The new caging moiety is based on the polypyridine pentadentate ligand *N* ^6^,*N* ^*6″*^-di­(pyridin-2-yl)-[2,2′:6′,2″-terpyridine]-6,6″-diamine (baptpy), which once coordinated to ruthenium­(II) can form the helically chiral [Ru­(baptpy)­(**L**)]­X_2_ prodrugs [**6**]­Cl_2_-[**16**]­Cl_2_. A total of ten active pharmaceutical ingredients (**L**) have been successfully conjugated to this photocage, including well-known agents such as **Albendazole**, **Gemcitabine**, **Bosutinib**, **Neratinib**, and **Ponatinib**. The X-ray crystal structures of seven complexes were obtained, showing coordination of ligand **L** via its thioether, nitrile, pyridine or imidazole moieties. All prepared ruthenium compounds showed selective photosubstitution of the monodentate ligand under 625 (red) and 730 nm (far-red) light irradiation with good (0.005–0.05) to excellent (0.05–0.10) quantum yields, while no singlet oxygen generation was observed. As calculated by density functional theory and time-dependent density functional theory, the high-wavelength light absorption of [Ru­(baptpy)­(**L**)]­X_2_ complexes and their favorable ligand exchange behavior under low-energy light are the consequence of the strong distortion of the first coordination sphere, combined with the electronic effect of the amine bridges of the baptpy ligand. Preliminary biological activity of complexes [**6**]­Cl_2_-[**16**]­Cl_2_ was investigated *in vitro* by cytotoxicity studies in the dark and under red light irradiation in normoxic A375 and U-87MG human cancer cell lines. Several of the obtained PACT prodrugs exhibited micro- to nanomolar EC_50_ values upon red light activation, with photoindexes as high as 7.5. [**7**]­Cl_2_ showed a photoindex of 6.2 upon far-red light activation (730 nm), which is unprecedented for a ruthenium-based PACT, while the ruthenium cage itself showed very low toxicity in both the dark and light irradiation.

## Graphical abstract

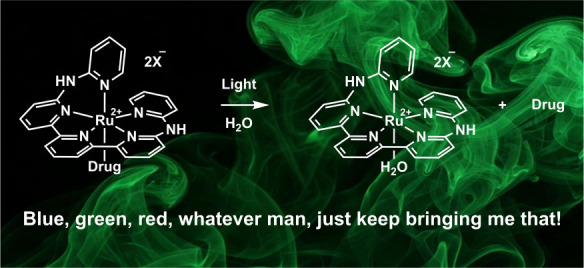

## Introduction

Photoactivated chemotherapy (PACT) represents a new modality in cancer treatment that integrates some principles of photodynamic (PDT) and photothermal therapy (PTT) with traditional chemotherapy.^1–4^ Unlike PDT and PTT, the PACT approach capitalizes on the ability of certain organic molecules and metal complexes to conjugate chemotherapy agents via thermally stable but photolabile chemical bonds, thereby suppressing their biological activity until light exposure triggers the cleavage. This mechanism allows for achieving great spatial and temporal control over drug activation, significantly reducing systemic toxicity and providing a unique way of physically targeting cancer even with nonspecific medication.^5^

Many examples of PACT have been reported over the years, both using purely organic photocages^6,7^ and those whose release mechanism relies on metals such as zinc, iron, ruthenium, iridium, or osmium.^8–14^ While the first class represents a more classical approach in medicinal chemistry as no precious elements are involved,^15^ the second tries to benefit from the added value of the metal-based cage, that can act in **Cisplatin**-like manner and generate a synergistic antitumor effect.^16^ Ruthenium complexes, in particular, such as **NAMI-A**, **KP1019** and **BOLD-100** can interact with albumin and transferrin in blood plasma, collagens and actins on the cell surface, or regulatory enzymes and DNA inside cells.^17^ It is therefore not surprising that a great deal of the PACT prodrug family is represented by ruthenium­(II) compounds, which among other factors allow for high water solubility, good metabolic stability, high molar absorption coefficients, and efficient photocleavage with low-energy light. Another attractive feature is the synthetic applicability toward existing small molecule drugs that coordination cages can grant. Pyridines, for instance, account for nearly 14% of US Food and Drug Administration (FDA) database,^18^ but there are also nitriles, thioethers, imidazoles and various *N*-heterocycles that can coordinate to transition metals with ease. Including ruthenium in PACT compounds also opens the opportunity of radio-labeling with ^97^Ru or ^103^Ru for medical imaging,^19^ of studying the prodrug biodistribution using inductively coupled plasma mass spectrometry (ICP-MS), and allows to build potent multiaction agents.^20,21^

All ruthenium­(II) complexes are usually octahedral and typically consist of several polydentate ligands like 2,2′-bipyridine (bpy) or 2,2′:6′,2″-terpyridine (tpy) arranged in various ways, which ultimately controls the overall photochemical behavior (Figure 1).^1^ Due to the entropy-driven chelate effect, ligands of higher denticity like tpy are harder to dissociate from the metal center than bidentate bpy or monodentate pyridine, thus providing good selectivity in photosubstitution reactions. As the vast majority of small molecule drugs have only one coordination site at best, two or even three different agents can be caged by a single ruthenium­(II) center simultaneously. In practice, however, one drug per cage is the most common assembly, unless the cage itself is also a drug,^22^ as photorelease of the secondary or tertiary independent moiety requires significantly longer light irradiation times. For example, complexes like [Ru­(tpy)­(**L**)_3_]^2+^, [Ru­(bpy)_2_(**L**)_2_]^2+^, [Ru­(bapbpy)­(**L**)_2_]^2+^ (bapbpy = *N* ^6^,*N* ^*6′*^-di­(pyridin-2-yl)-[2,2′-bipyridine]-6,6′-diamine), [Ru­(tpa)­(**L**)_2_]^2+^ (tpa = tris­(2-pyridylmethyl)­amine) and [Ru­(tpy)­(bpy)­(**L**)]^2+^ were shown to exchange at least one of their monodentate ligands **L** to water, acetonitrile (ACN), dimethyl sulfoxide (DMSO), or chloride anion under visible light irradiation.^23–27^ Depending on the environment and the exact compound structure, the activation wavelength can be anywhere between ultraviolet (UV) and near-infrared (NIR), with the last being most desirable due to deeper tissue penetration and lower collateral radiation harm.^28^

**Figure 1:**
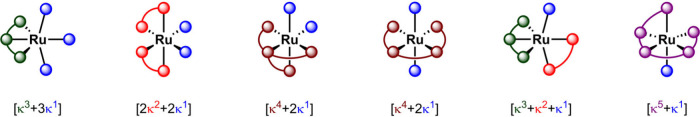
Overview of the most common ruthenium-based photocages containing one to three vacant binding sites (in blue; κ^n^ indicates denticity of ligands).

A wide variety of tools can be exploited to make ruthenium-based coordination photocages more reactive toward low-energy light. One of them is the *trans* effect,^29^ which consists in weakening Ru-**L** bond by placing a suitable moiety *trans* to the leaving ligand **L**. Namely, it is possible to use a π-acceptor, which competes for the metal d-orbital electrons involved in the back-bonding to ligand **L**, or a π-donor that increases electron density on the metal so much that antibonding orbitals involving **L** start being populated. One such example is [Ru­(tpy)­(acac)­(ACN)]^+^ (acac^–^ = acetylacetonate) in which replacement of bpy by acac^–^ leads to a significant increase in the red and far-red light photoreactivity.^30–32^ Another strategy is to create steric hindrance between **L** and the cage, which can ease the photosubstitution of strong-field ligands and bidentate payloads, as for example in formerly reported [Ru­(tpy)­(biq)­(**STF-31**)]^2+^ (biq = 2,2′-biquinoline) or [Ru­(dmbpy)_2_(**IP-4T**)]^2+^ (dmbpy = 6,6′-dimethyl-2,2′-dipyridyl).^33,34^ The single bond attachment of ligand **L**, however, may not always be sufficiently thermally stable.^35^ Additionally, certain ligands or chemical modifications may be detrimental to ligand photosubstitution and shift the photoreactivity of ruthenium-based complexes toward phosphorescence, reactive oxygen species (ROS) generation, or nonradiative decay.^36–38^ Overall, the design of PACT prodrugs requires careful balance between the number of drugs incorporated, their denticity, and the ease of their photorelease.

In this work, we expanded the toolbox of ruthenium­(II) photocages for PACT by introducing the new pentadentate ligand *N* ^6^,*N* ^*6″*^-di­(pyridin-2-yl)-[2,2′:6′,2″-terpyridine]-6,6″-diamine (baptpy) and its complex [Ru­(baptpy)­Cl]­Cl. This compound can be used to produce [Ru­(baptpy)­(**L**)]^2+^ prodrugs capable of efficiently photoreleasing different coordinative moieties using red (625 nm) or even far-red (730 nm) light. A wide range of monodentate ligands can be photocaged using this new ruthenium complex, including amines, thioethers, nitriles, pyridines, β-carbolines, imidazoles, and imidazo­[1.2-b]­pyridazines. Practical examples include the experimental anticancer drugs **Entinostat**,^39^ **STF-31**,^40^ **QC-82**,^41^ **RAD-51-IN-1**,^42^ **Norharmane**,^43^ **MTI**,^44^ **NSC745885**,^45,46^ as well as the clinically approved **Albendazole**, **Gemcitabine**, **Bosutinib**, **Neratinib**, **Ponatinib** and **Pazopanib**. Photosubstitution quantum yields were found to be high in spite of the low-energy light trigger. Significant efforts were invested into understanding the limits of this new photocage for PACT, the differences of the [κ^5^+κ^1^] octahedral system from the previously studied scaffolds, and the reasons for its reactivity toward far-red light.

## Results and Discussion

### Synthesis and Characterization

The conversion of tpy into baptpy and the preparation of complexes [**4**]­Cl-[**16**]­Cl_2_ was accomplished by the route shown in Figure 2. A multigram synthesis of [**4**]Cl was performed in four consecutive steps from commercially available tpy with an overall yield of 71%. The baptpy ligand **3** was obtained by Buchwald-Hartwig cross-coupling of 2-bromopyridine and recently reported [2,2′:6′,2″-terpyridine]-6,6″-diamine (**2**).^47^ For the caging of inhibitors such as thioether **MTI** that poorly tolerate *in situ* usage of AgPF_6_, the aqua complex [**5**]­(PF_6_)_2_ was also prepared. Additionally, to evaluate the photocytotoxicity of the ruthenium moiety itself, complex [**6**]­Cl_2_ was made, assuming that pyridine had low toxicity to cancer cells.^48^ Finally, the caging of ten known drugs **L** was performed using either complex [**4**]Cl with AgPF_6_ in acetone/H_2_O = 1/1 (v/v), or [**5**]­(PF_6_)_2_ in DMF at 50 °C for 24 h under N_2_ in the dark. During reaction workup, the hexafluorophosphate counterions were replaced by chloride whenever possible, to improve the water solubility of the final ruthenium conjugates. All the compounds presented in this study were characterized with NMR, ESI-MS, HRMS, elemental analysis, UV–Vis and single crystal X-ray diffraction when applicable, the respective data can be found in the Supporting Information.

**Figure 2:**
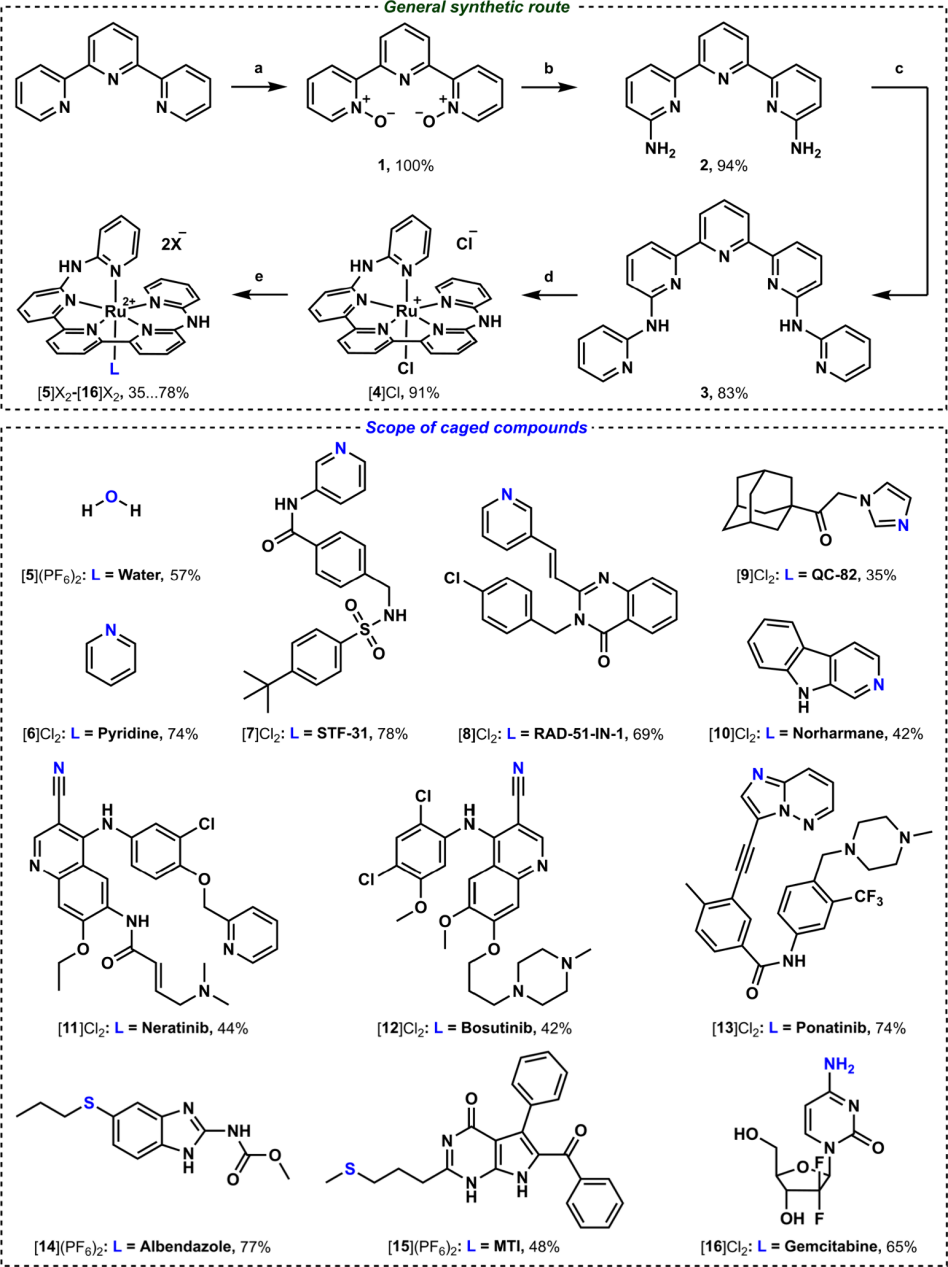
General **s**ynthetic route toward compounds [**5**]­X_2_-[**16**]­X_2_, where X stands for Cl or PF_6_, and **L** for the photocaged drug. Reaction conditions: (a) *m*CPBA, DCM, r.t., 24 h; (b) (i) Potassium phthalimide, TEA, TsCl, ACN, r.t., 24 h; (ii) N_2_H_4_·H_2_O, H_2_O, 80 °C, 24 h; (c) 2-Bromopyridine, Pd­(dba)_2_, *rac*-BINAP, *t*-BuOK, Toluene, N_2_, 110 °C, 48 h; (d) [Ru­(*p-*cymene)­Cl_2_]_2_, MeOH, N_2_, 65 °C, 24 h; (e) **L**, AgPF_6_, Acetone/H_2_O = 1/1 or DMF, N_2_, 50 °C, 24 h.

It is worth mentioning that the [Ru­(baptpy)­(**L**)]^2+^ photocage is helically chiral, as can be seen from the crystal structures shown in Figure 3. Therefore, it forms a mixture of enantiomers when bound to nonchiral drugs like **STF-31** in [**7**]­Cl_2_ for example, and a mixture of diastereomers when bound to chiral inhibitors like **Gemcitabine** in [**16**]­Cl_2_. At this stage, we did not try to separate these isomers and used them as mixtures. Apart from the set of drugs shown in Figure 2, several others were tried that, however, did not yield the desired prodrugs. For example, thiadiazole **NSC745885** failed coordinating to cage [**4**]Cl despite multiple reports on similar heterocyclic moiety being able to bind ruthenium.^49,50^ A similar issue occurred with **Pazopanib**, either due to the poor binding affinity of monodentate 2*H*-indazoles, or because of the sterically demanding methyl group next to a possible coordination site.^51,52^ The pyridine-based drug **Entinostat** showed promises for caging with [**4**]­Cl, but the reproducibility of the synthesis proved to be poor due to the benzimidazole condensation reaction on the other end of this molecule. A few other drugs including **Ibrutinib**, **Telmisartan**, **Erlotinib**, **Tacrine** and **Losartan** failed to coordinate to the photocage [**4**]­Cl. It should also be noted that organic drugs with multiple coordination sites, such as **Nilotinib** or **Apatinib**, may generate regioselectivity issues during preparation and even be double caged. This does not mean that photocaging is impossible for these compounds, but synthesis would likely require extensive purification and the reaction yield would probably be low. Despite these limits, [**4**]Cl and [**5**]­(PF_6_)_2_ appeared as highly versatile ruthenium­(II) precursors for the caging of monodentate inhibitors, as an unprecedented variety of 10 chemotherapy drugs were obtained at minor synthetic costs.

**Figure 3:**
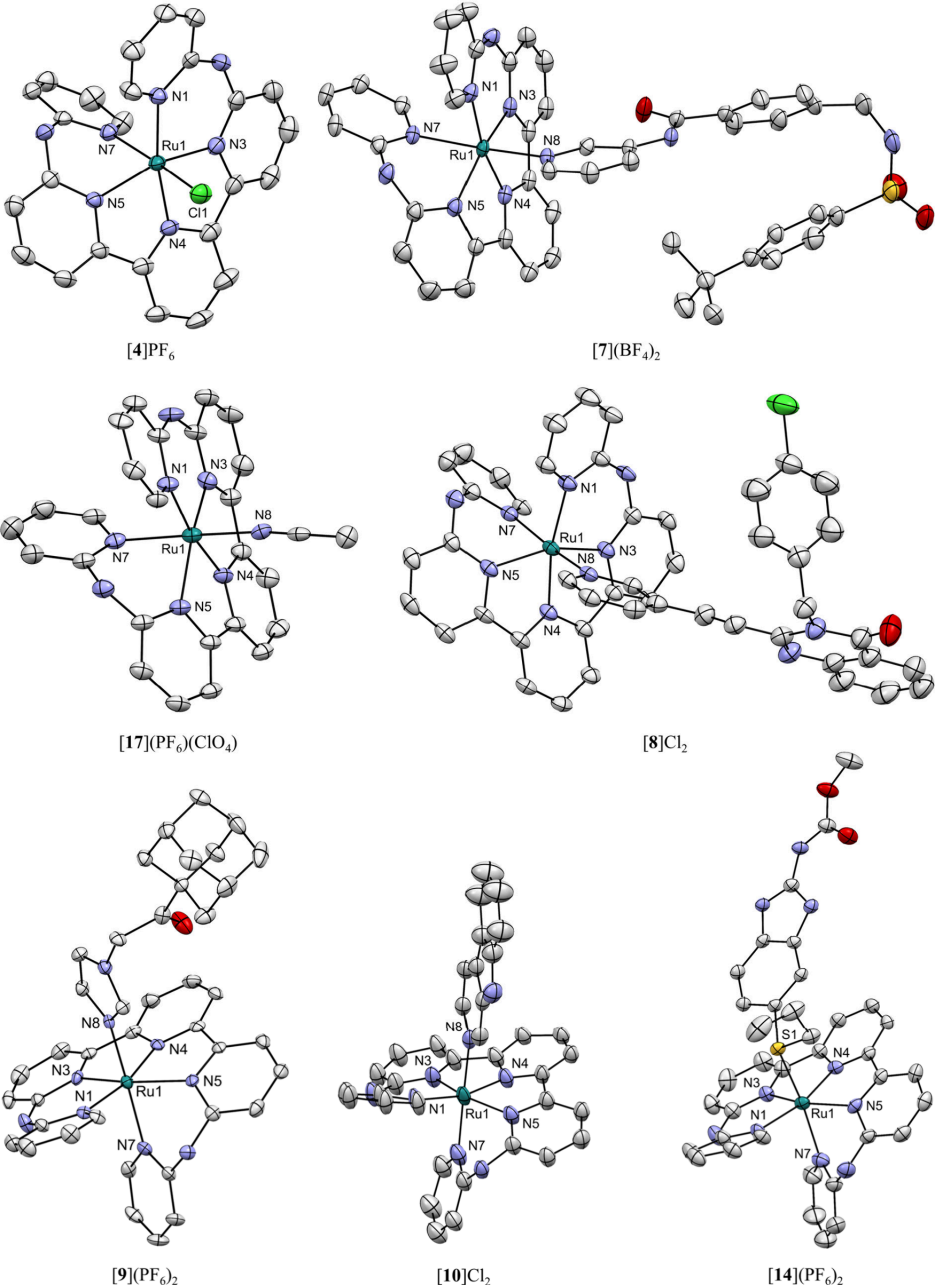
Displacement ellipsoid plots (50% probability level) for the crystal structures of complexes [**4**]­PF_6_, [**7**]­(BF_4_)_2_, [**8**]­Cl_2_, [**9**]­(PF_6_)_2_, [**10**]­Cl_2_, [**14**]­(PF_6_)_2_, and [**17**]­(PF_6_)­(ClO_4_). All hydrogen atoms, cocrystallized solvent molecules, and counterions have been omitted for clarity.

### Single Crystal X-ray Crystallography

Crystal structures were obtained for seven baptpy ruthenium­(II) complexes, i.e., [**4**]­PF_6_, [**7**]­(BF_4_)_2_, [**8**]­Cl_2_, [**9**]­(PF_6_)_2_, [**10**]­Cl_2_, [**14**]­(PF_6_)_2_, and [**17**]­(PF_6_)­(ClO_4_) derived from [**5**]­(PF_6_)_2_ (Figure 3). All of the presented ruthenium compounds were found to be highly soluble in polar solvents like MeOH, especially with chloride counterions, and in acetone or ACN for the PF_6_ salts. The chloride compounds were poorly soluble in acetone or EtOAc and all complexes were not soluble in nonpolar solvents such as Et_2_O or pentane. The solubility in organic solvents changed drastically upon counterion exchange, and this property was extensively exploited not only for purification but also for growing crystals. Single crystals of [**4**]­PF_6_, for instance, were obtained by dissolving the material in MeOH and then adding a few drops of aqueous 60% HPF_6_. Similarly, a few drops of the HBF_4_ diethyl ether complex were used to obtain crystals of [**7**]­(BF_4_)_2_. Unlike [**4**]­PF_6_ and [**7**]­(BF_4_)_2_, compound [**9**]­(PF_6_)_2_ under these two conditions produced enantiopure crystals in the P1 space group, breaking the common centrosymmetric trend where both enantiomers cocrystallize. For compounds [**8**]­Cl_2_ and [**10**]­Cl_2_ ion exchange did not yield single crystals, and thus a more traditional way of vapor diffusion with DMF/THF (solvent/counter-solvent) was used. The same technique was applied to complex [**14**]­(PF_6_)_2_ but with acetone/Et_2_O, and [**17**]­(PF_6_)­(ClO_4_) with ACN/Et_2_O. Finally, compounds with large aliphatic tails such as [**11**]­Cl_2_ **-**[**13**]­Cl_2_ or diastereomeric mixtures [**16**]­Cl_2_ failed to produce any crystals despite a huge variety of conditions tried. Overall, all obtained crystal structures showed that [Ru­(baptpy)­(**L**)]^2+^ complexes are in distorted octahedral geometries with the baptpy ligand helically enveloping the ruthenium­(II) center and occupying five of the six coordination sites. In the structures of [**8**]­Cl_2_ and [**10**]­Cl_2_ the chloride counterions formed strong hydrogen bonds with the two NH-bridging groups of the baptpy ligand. In [**14**]­(PF_6_)_2_ the **Albendazole** molecule underwent displaced π-stacking with one of the baptpy rings, seemingly providing stabilization to the overall structure. Other π-π interactions were usually found between two enantiomers like in the structure of [**4**]­PF_6_, within the inhibitor as in [**7**]­(BF_4_)_2_, or between coordinated drug moieties as in [**8**]­Cl_2_. All crystallography data and a selection of bond lengths and angles can be found in the Supporting Information.

### Thermal Stability and Photochemistry

The thermal stability and photochemical characterization of ruthenium-based PACT compounds typically include measurement of their molar extinction coefficient (ε_λ_), the evolution of their absorption and ^1^H NMR spectra under light irradiation, mass spectrometry analysis before and after light irradiation ideally coupled to HPLC, as well as photosubstitution (Φ_λ_), singlet oxygen (Φ_Δ_), and photoluminescence (Φ_P_) quantum yields. Due to the high dependence of ligand-exchange photoreactions on the environment,^53^ all these analyses are preferably done in a unique solvent to obtain a complete view of the (photo)­reactivity of these complexes in a single set of conditions. Here, the photochemical characterization of prodrugs [**6**]­Cl_2_-[**16**]­Cl_2_ was mostly performed in ACN. Of course, pure water is a closer replica of biological environments, but incorporation of highly hydrophobic moieties such as **STF-31**, **MTI** or **Ponatinib** into ruthenium complex makes them water-soluble only when they are bound to the metal. Once photoreleased, they immediately precipitated out of the aqueous solution, thus preventing reliable spectroscopic data collection. This problem can be avoided by adding MeOH or acetone to prevent precipitation, but ^1^O_2_ detection is challenging in water while it can be performed easily in ACN. Additionally, in cell-growing medium or in cells a wide range of ligands may substitute the photocleaved organic inhibitor, thus the ruthenium-containing photoproduct is difficult to identify. Quantification of the photoreactivity can only be done when the photoproduct is clearly identified, for example, [Ru­(baptpy)­(ACN)]^2+^ when performing irradiation in ACN. DMSO is another possibility, but it is less favorable than ACN due to possible S/O–Ru linkage photoisomerization reactions that complicate spectrophotometric analysis of ligand exchange.^54^ Due to its high viscosity, hygroscopicity, and singlet oxygen quenching properties, this solvent may modify the photoreactivity of ruthenium complexes.^55^ Overall, ACN afforded the best compromise between the solubility, solvent coordination properties, and feasibility of all spectroscopic techniques to characterize the thermal stability and photochemistry of these ruthenium complexes. Still, we also performed stability experiments in water containing 2% v/v MeOH and light activation experiments in cell culture media to verify how the results found in acetonitrile translated to aqueous solutions.

Remarkably, in ACN the ruthenium cage [**4**]Cl showed light absorption over the entire visible spectrum, up to 800 nm. To some extent, this property has transferred to the photocaged complexes [**6**]­Cl_2_-[**16**]­Cl_2_: all showed good (500 M^–1^cm^–1^) to high (2000 M^–1^cm^–1^) molar absorption coefficients for red light (625 nm), while values at 730 nm were low (8.4 M^–1^cm^–1^) to average (up to 600 M^–1^cm^–1^), notably compared to Turro’s monocationic [Ru­(tpy)­(acac)­(ACN)]^+^ or [Ru­(dqpy)­(acac)­(ACN)]^+^ photocages (dqpy = 2,6-di­(quinolin-2-yl)­pyridine) that have molar absorption coefficients around 700 nm, i.e., ∼ 1000–2000 M^–1^cm^–1^,^56,57^ or Zhang’s dinuclear compound (8700 M^–1^cm^–1^ at 700 nm).^58^ Most importantly, all compounds except the one based on **Gemcitabine** ([**16**]­Cl_2_) showed excellent dark stability in ACN over 24 h at 25 °C (Figures S141, S144, S147, S150, S153, S156, S159, S162, S165, S168, and S171). To verify that thermal stability was also good in aqueous solutions, we also performed thermal stability studies for [**6**]­Cl_2_-[**16**]­Cl_2_ (0.1 mM) in water containing 2% v/v MeOH, using both UV–Vis spectroscopy and HPLC (Figures S172–S193). For compounds [**6**]­Cl_2_-[**10**]­Cl_2_, [**12**]­Cl_2_, [**14**]­(PF_6_)_2_, [**15**]­(PF_6_)_2_ and [**16**]­Cl_2_ we found good dark stability in aqueous solution, with limited (<8%) decrease of the HPLC peak area of the initial complex after 24 h at 25 °C. Interestingly, [**11**]­Cl_2_ and [**13**]­Cl_2_ were found thermally unstable in such conditions (Figures S182–S183 and S186–S187, respectively) while they were thermally stable in ACN. This observation suggested ligand-specific degradation processes in the presence of water . Surprisingly, the **Gemcitabine** complex [**16**]­Cl_2_ was found to be more thermally stable in water containing 2% v/v MeOH than in pure ACN, suggesting that the excellent coordination properties of ACN may be responsible for thermal degradation of [**16**]­Cl_2_ in this solvent. Overall, these studies concluded the [Ru­(baptpy)­(L)]^2+^ scaffold was essentially stable in aqueous and acetonitrile solutions at room temperature in the dark, but that differences in the rate of thermal degradation arise between these two solvent (e.g., for [**16**]­Cl_2_) while ligand-specific thermal decomposition processes might also take place (e.g., for [**11**]­Cl_2_ and for [**13**]­Cl_2_).

To study the photoreactivity of these complexes, low-energy light irradiation of [Ru­(baptpy)­(**L**)]^2+^ conjugates was further followed by mass spectrometry and UV–Vis spectroscopy in acetonitrile, ^1^H NMR spectroscopy in DMSO-*d_6_*, and HPLC in Opti-MEM complete, using red to far-red light sources. In these experiments, mass spectrometry (Figure S195–S204), HPLC (Figure S232–S244), and ^1^H NMR (Figures 5 and 6) were used first, to qualitatively establish the nature of the photoproducts and hence of the photoreaction. Once the photosubstitution reaction was established, we used UV–Vis data under a known photon flux to determine its quantum yield in acetonitrile, which for a [Ru­(baptpy)­(**L**)]^2+^ complex also represented the quantum yield of uncaging of drug **L**. Indeed, mass spectrometry, UV–Vis spectroscopy, HPLC, and ^1^H NMR studies unequivocally proved that all ruthenium compounds (including [**16**]^2+^) underwent photochemical cleavage of the coordination bond between ruthenium and the monodentate ligand **L**, producing the free intact drug **L** together with a solvent- or chloride-bound ruthenium photocage (see below). Such selective ligand exchange reactions occurred sometimes with low quantum yields (∼0.001 for imidazole-based [**9**]^2+^), but also sometimes with excellent ones (up to 0.100 at 625 nm and 0.0528 at 730 nm for thioether-based complex [**15**]^2+^). Such low-energy photoreactivity is crucial for biological applications as the PDT optical window lies between 600 and 1200 nm.^28^ To complete the overview on the photochemical reactivity of these complexes, the measurements of their quantum yields for photoluminescence and for singlet oxygen phosphorescence were also conducted in ACN at 25 °C, which showed negligible values typical of purely PACT compounds (Φ_P_ < 0.0002, Φ_Δ_ < 0.01). The individual data set for each compound can be found in the Supporting Information, and the main photochemical parameters are summarized in Table 1.

**Table 1:** Absorption Maxima (λ_max_), Molar Absorption Coefficients (ε_625_ and ε_730_), Photosubstitution (Φ_625_ and Φ_730_), Phosphorescence (Φ_P_) and Singlet Oxygen (Φ_Δ_) Quantum Yields in Air-Saturated ACN at 25 °C for Compounds [**4**]­Cl, [**6**]­Cl_2_-[**16**]­Cl_2_ ^a^

**Compound**	**λ**_**max**_, **nm**	**ε**_**625**_, **M** ^**‑1**^ **cm** ^**‑1**^	**ε**_**730**_, **M** ^**‑1**^ **cm** ^**‑1**^	**Φ**_**625**_	**Φ**_**730**_	**Φ**_**P**_	**Φ**_**Δ**_
[**4**]Cl	523	2020	1460	–	–	0.00002	0.003
[**6**]Cl_2_	489	1310	36	0.0118	0.0202	0.00004	0.001
[**7**]Cl_2_	488	1380	37	0.0119	0.0138	0.00002	0.003
[**8**]Cl_2_	486	1320	74	0.0197	0.0155	0.00004	0.007
[**9**]Cl_2_	501	1590	144	0.0008	0.0015	0.00003	0.004
[**10**]Cl_2_	496	1710	132	0.0021	0.0022	0.00006	0.004
[**11**]Cl_2_	478	1060	222	0.0613	0.0024	0.00011	0.004
[**12**]Cl_2_	475	963	126	0.0799	0.0041	0.00002	0.002
[**13**]Cl_2_	491	1870	582	0.0184	0.0026	0.00016	0.006
[**14**](PF_6_)_2_	486	692	40	0.0745	0.0084	0.00009	0.005
[**15**](PF_6_)_2_	482	509	8.4	0.1000	0.0528	0.00007	0.003
[**16**]Cl_2_	504	2100	631	–	–	–	–

a Unlike all other compounds, [**16**]­Cl_2_ was found thermally unstable in ACN at 25 °C over 24 h. Φ_P_ and Φ_Δ_ were measured under 450 nm blue light excitation.

As a typical example, the UV–Vis data for [**13**]­Cl_2_ irradiated with 625 nm red and 730 nm far-red light in ACN is shown in Figure 4. The evolution of its absorption spectrum in ACN under far-red light appeared as a single photochemical step with well-defined isosbestic points, which afforded the solvent-bound ruthenium photocage [Ru­(baptpy)­(ACN)]­Cl_2_ ([**17**]­Cl_2_) and free **Ponatinib** (Figure 4c). This finding was supported by mass spectral comparison of dark and irradiated samples, indicating the complete conversion of [**13**]^2+^ (*m*/*z* = 525.6) into [**17**]­Cl^+^ (*m*/*z* = 595.1) and free **Ponatinib** (*m*/*z* = 533.2). The irradiation with red light, however, revealed the presence of a secondary photoreaction that followed **Ponatinib** photosubstitution (Figure 4b). This process was defined by an increase of the absorbance in the 650–800 nm region and the appearance of a ^1^MLCT peak at 523 nm, both characteristic for the chloride complex [**4**]­Cl. According to our analysis, the ACN complex [**17**]­Cl_2_ formed after initial drug release under red light irradiation, underwent further photodissociation toward [**4**]­Cl, as confirmed by the appearance of [**4**]^+^ (*m*/*z* = 554.0). The [**4**]^+^ signal was also present in the red light irradiated samples of [**7**]­Cl_2_, [**9**]­Cl_2_, and [**12**]­Cl_2_, though most of the time it overlapped with the other peak of the acetonitrile complex [**17**–H]^+^ (*m*/*z* = 559.1), or even free drug as in case of **Neratinib** prodrug [**11**]­Cl_2_ (*m*/*z* = 557.5). The same photoinduced chloride association process was found to take place in DMSO solutions, and it is consistent with previous reports on [κ^3^+κ^2^+κ^1^] ruthenium­(II) complexes.^59^ For example, the evolution of the ^1^H NMR spectrum of [**7**]­Cl_2_ in DMSO-*d*_6_ upon red and far-red light irradiation (Figure 5 and Figure 6) showed the appearance of peaks at 8.92, 7.86, and 4.09 ppm (marked with (^)) characteristic for the free **STF-31**, thereby aligning with the UV–Vis, mass spectrometry, and HPLC observations. In the case of red light, this main photosubstitution process was also accompanied by slow conversion of the primary photoproduct [Ru­(baptpy)­(DMSO-*d*_6_)]­Cl_2_ into [**4**]­Cl, characterized by peaks at 9.79, 8.60, and 8.50 ppm (marked with (*)) and changes in the whole area from 6.00 to 7.40 ppm. At this early stage, we hypothesized that this secondary photoreaction of producing [**4**]Cl has little biological relevance, as the main activity may come from the released drug. Furthermore, counterion association may not even occur in aqueous media due to strong solvation effects, as was shown for [Ru­(tpy)­(biq)­(H_2_O)]­Cl_2_.^33,59^

**Figure 4:**
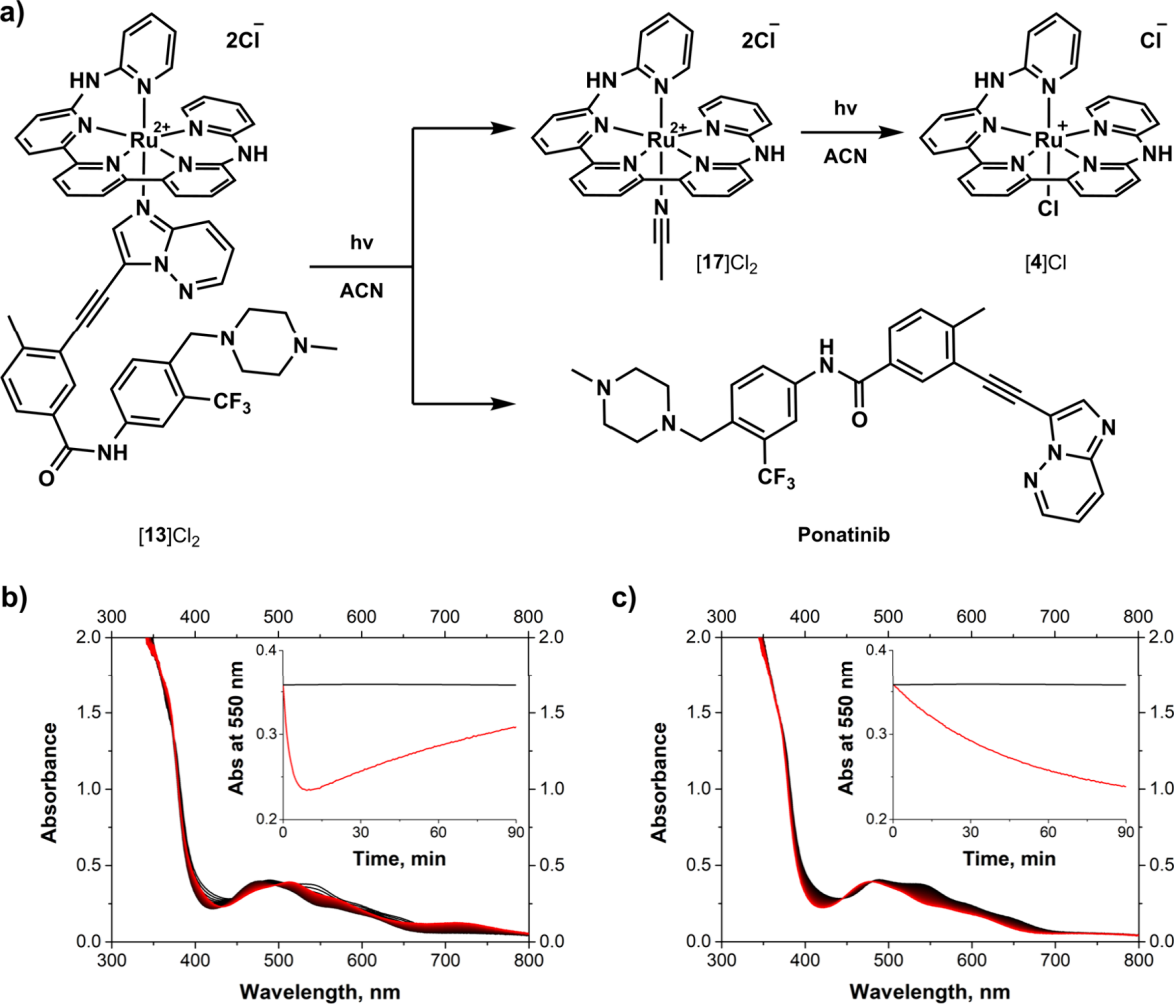
(a) Ligand photosubstitution observed for [**13**]­Cl_2_ is similar to all presented PACT compounds. Evolution of the absorption spectra of [**13**]­Cl_2_ in ACN (0.1 mM solution) upon irradiation with 625 nm red (b, 10.7 mW/cm^2^) and 730 nm far-red light (**c**, 8.9 mW/cm^2^) for 1.5 h at 25 °C. Spectra were measured every 0.5 min and evolved from black t_0_ → red t_90_, the insets show absorbance under light vs. dark control.

**Figure 5:**
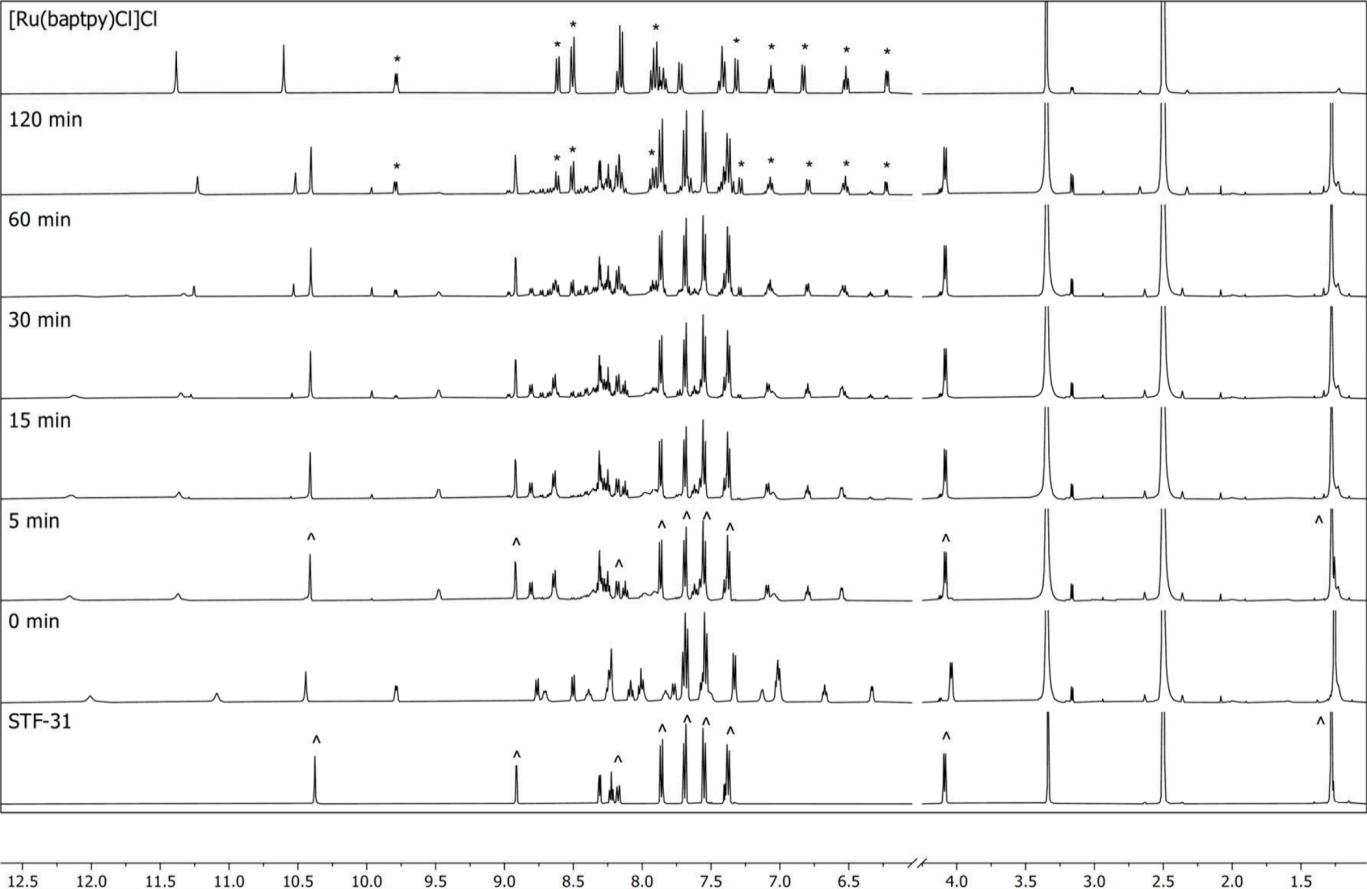
Evolution of the ^1^H NMR spectra of [**7**]­Cl_2_ in DMSO-*d*_6_ upon irradiation with 650 nm red light at 25 °C for 2 h. The data were collected at intervals of 0, 5, 15, 30, 60, and 120 min on Bruker Avance 500 MHz and compared to spectra of [Ru­(baptpy)­Cl]Cl and **STF-31** (characteristic peaks are marked with (*) and (^), respectively).

**Figure 6:**
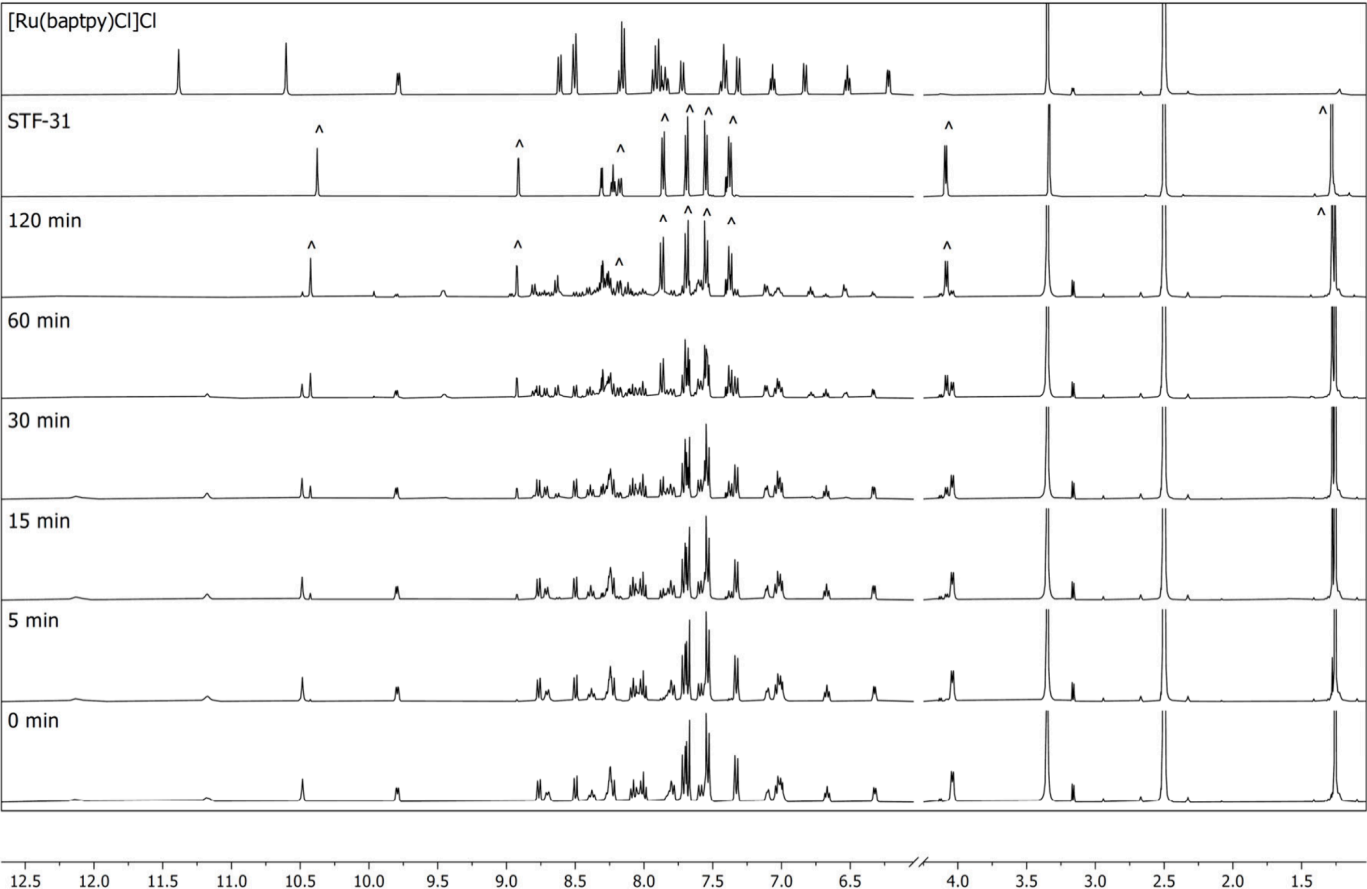
Evolution of ^1^H NMR spectra of [**7**]­Cl_2_ in DMSO-*d*_6_ upon irradiation with 730 nm far-red light at 25 °C for 2 h. The data were collected at intervals of 0, 5, 15, 30, 60, and 120 min on Bruker Avance 500 MHz and compared to the spectrum of [Ru­(baptpy)­Cl]Cl and **STF-31** (characteristic peaks are marked with (^)).

To sum up, compounds [**6**]­Cl_2_-[**16**]­Cl_2_ were all found to show very low phosphorescence and singlet oxygen generation quantum yields, with ligand photosubstitution being the dominant photochemical reactivity. Their thermal stability in ACN, water containing 2% v/v MeOH, and Opti-MEM complete was good, except for the **Gemcitabine** complex [**16**]­Cl_2_, which was found to slowly but fully decompose in acetonitrile over 24 h at 25 °C, marking the border of the caging capacity of [Ru­(baptpy)­(**L**)]^2+^. As a note, for this unstable complex [**16**]­Cl_2_ the photosubstitution with red and far-red light did work, but only in the sense that it led to a faster drug release under irradiation compared to the dark control. In water containing 2% v/v MeOH, [**16**]­Cl_2_ was found reasonably stable, in fact more stable than complexes [**11**]­Cl_2_ and [**13**]­Cl_2_ which showed signs of thermal cleavage over 24 h, up to 34% for [**13**]­Cl_2_ by HPLC. It is noteworthy that [**11**]­Cl_2_ was found three times less thermally stable in water than [**12**]­Cl_2_, despite the fact that both are similar nitrile complexes. Overall, thioether complexes [**14**]­(PF_6_)_2_ and [**15**]­(PF_6_)_2_ performed best in this series, showing the highest photoreactivity toward red and far-red light, while maintaining excellent thermal stability both in ACN and water, which is remarkable.^60^

The new pentadentate baptpy ligand generates a helical geometry upon coordinating transition metals like ruthenium, somewhat similar to previously reported 6,6″-di­(quinolin8-yl)-2,2′:6′,2″-terpyridine (dqtpy), except the baptpy is less rigid and does not have π-π stacking of side arms.^61^ While dqtpy was only coordinated to cobalt­(II) and iron­(II) to study light-driven CO_2_ reduction, there is no doubt it shares many similarities with baptpy and might as well be a good candidate for building new ruthenium­(II) photocages. Due to the larger geometry constrains of dqtpy, however, it might be capable of reversible axial side arm photodissociation, which was not observed for baptpy complexes, but can significantly impact toxicity.^62^

The comparison between [Ru­(baptpy)­(**STF-31**)]­Cl_2_ and the formerly reported [Ru­(tpy)­(biq)­(**STF-31**)]­Cl_2_ highlights the importance of geometry distortions in the first coordination sphere for achieving efficient ligand photosubstitution with low-energy light.^33,53,63^ While the previous complex was designed to increase the steric hindrance between the coordinated drug moiety and the cage itself, the latter makes use of the rigidity of the cage possessing a baptpy ligand, the strong chelating effect, and a higher degree of conjugation in the equatorial plane. Unlike [κ^3^+κ^2^+κ^1^] complexes, the [κ^5^+κ^1^] baptpy system provides plenty of space for coordinating various bulky monodentate drug payloads, even providing π-π stacking helipad as was seen in crystal structure of [**14**]­(PF_6_)_2_, yet it remains highly reactive to visible light. The photochemical response is especially pronounced when comparing [Ru­(baptpy)­(**MTI**)]­(PF_6_)_2_ to previously studied PACT prodrug [Ru­(Rtpy)­(bpy)­(**MTI**)]­(PF_6_)_2_ (Rtpy = 4′-(methylamido)-2,2′:6′,2″-terpyridine; bpy = 2,2′-bipyridine), with photoreactivity ζ_625_ = ε_625_Φ_625_ in ACN being 46 times higher for [**15**]­(PF_6_)_2_, and its quantum yield reaching nearly 10% (Table 1).^5^ At the same time, the new [Ru­(baptpy)­(**L**)]^2+^ photocage lacks the ability to produce singlet oxygen, and will hence require some structural modifications to perform well desired combination of PACT and PDT to combat both hypoxia and normoxia tumors at once.^20^

### DFT Studies

Quite often, red-shifting the absorption spectrum of ruthenium complex is accompanied by lower photosubstitution quantum yields.^38,64^ This trend is well-understood within the classical theory of photosubstitution reactions in ruthenium polypyridyl complexes. Photosubstitution typically occurs when photon absorption and intersystem crossing generate a triplet metal-to-ligand charge transfer (^3^MLCT) excited state, which is then thermally activated to a nearby triplet metal-centered (^3^MC) excited state with dissociative character.^65^ Usually, an energy barrier exists between the ^3^MLCT and ^3^MC state, necessitating thermal energy for the promotion of the electron in a ligand-based π* orbital to jump onto the nearest metal-based e_g_* orbital. Red-shifting the absorption spectrum requires stabilizing the ^3^MLCT states, which increases the gap between the ^3^MLCT and ^3^MC states and the energy barrier for their interconversion, thereby lowering the photosubstitution quantum yields. To red-shift the absorption while keeping appreciable photosubstitution efficiencies, this theory suggests also lowering the energy of the ^3^MC states. Here, given the obviously distorted helical structure of the baptpy complexes and the amine bridges extending the central terpyridine system toward two terminal pyridines, we performed a computational analysis of these compounds by static density functional theory (DFT) and time-dependent density functional theory (TDDFT) to identify how sterics and electronics explain their remarkable photochemical properties.

To do so we carried out a detailed analysis of complex [**6**]^2+^ *i*.*e*. [Ru­(baptpy)­(Py)]^2+^ as a typical example of the series, and compared it to its analogue [Ru­(tpy)­(Py)_3_]^2+^. The latter was reported to photosubstitute one of the axial pyridine ligands under blue light irradiation.^23^ Similar to [**6**]^2+^, it bears a terpyridine ligand, but it is deprived of any amine substituent, while three monodentate pyridine ligands complete the coordination sphere without additional distortion. The main structural distinction between these two compounds lies hence in the ligand strain generated by the pentadentate baptpy ligand vs. that generated by one tpy and two independent pyridines. Geometry optimization was first realized with the AMS 2023.101 engine^66^ at the B3LYP^67^/TZP^68,69^ /COSMO^70^/ZORA^71^ level, including the Grimme’s D3 dispersion corrections with BJ damping.^72^ At first glance, the helical structure of the minimized geometry of complex [**6**]^2+^ resulted in higher deviation from the perfect octahedral geometry, compared with [Ru­(tpy)­(Py)_3_]^2+^. The variance of the angles (σ^2^) in the first coordination sphere, measured relative to their ideal values of 90° and 180°, are summarized in Table 2. They were both larger in [**6**]^2+^, suggesting a more strongly distorted coordination sphere. To quantify the energy involved in this distortion, we decomposed the ligand strain energy (LSE) of both complexes in two components: the reorganization energy (RE), which is the total energy required to orient all nitrogen atoms toward the metal from a noncoordinated, relaxed conformation, and the internal strain energy (ISE), representing the energy expense arising from the strain that is applied on the ligand upon binding to the metal.^73^ To obtain the latter energy component, we performed a single point calculation of the ligand in the coordinated geometry (baptpy* or tpy*) at the same level of theory as that for the ruthenium complex. Subsequently, a new geometry optimization of each chelate was carried out, alleviating the strain that arose on them upon binding to ruthenium, to obtain a geometry named baptpy’ or tpy’ and the ISE (e.g., ISE = E­(baptpy*) – E­(baptpy′)). Finally, a conformer analysis was performed to identify the global minimum of each chelate, named baptpy or tpy, enabling us to determine the RE (e.g., RE = E­(baptpy′) – E­(baptpy)), and hence the LSE (Table 2). The RE and ISE were both found to be 3–4 times higher for [**6**]^2+^ than for [Ru­(tpy)­(Py)_3_]^2+^, with the total LSE being 4 times larger. Overall, [**6**]^2+^ was found to be much more strained than [Ru­(tpy)­(Py)_3_]^2+^, suggesting that its ligand field strength and ^3^MC energy level are lower.

**Table 2:** Calculated Deformation Energy^a^

**Compound**	**RE**, kJ/mol	**ISE**, kJ/mol	**LSE**, kJ/mol	**σ** ^**2**^ **90° angles**	**σ** ^**2**^ **180° angles**
[Ru(tpy)(Py)_3_]^2+^	15.16	39.45	54.61	46.36	238.35
[Ru(baptpy)(Py)]^2+^	64.42	141.76	206.18	105.76	378.41

a σ^2^ represents the variance of the angles Θ in the first coordination sphere of the octahedral complex, measured relative to their ideal values of 90° and 180°: $\sigma^{2}=\frac{1}{11}\overset{12}{\underset{i=1}{\sum }}{\left(\Theta -90/180\right)}^{2}$

In a second approach, TDDFT calculations were performed at the same level of theory as that of DFT (Table 3). The results indicated that the lowest-energy transition of [Ru­(baptpy)­(Py)]^2+^ was significantly lower (2.183 eV, 568.04 nm) than that of [Ru­(tpy)­(Py)_3_]^2+^ (2.414 eV, 513.63 nm). As is common in ruthenium­(II) octahedral complexes, both transitions were of MLCT character. The energies of the highest occupied molecular orbital (HOMO) and the lowest occupied molecular orbital (LUMO) of the complexes increased when going from [Ru­(tpy)­(Py)_3_]^2+^ to [**6**]^2+^, but the HOMO was more destabilized than the LUMO, leading to a much lower HOMO–LUMO gap for the latter (3.066 eV), compared with the former (3.341 eV). The increase in the energy of the HOMO can be explained partly by electronic effects resulting from the amine bridge. The HOMO in [**6**]^2+^ is metal-based, with all t_2g_ orbitals higher in energy due to the π-donor effect of the amine bridges. Additionally, part of the higher HOMO level can be attributed to distortion of the octahedral shape. The slight increase in the energy of the LUMO is due to the π* orbital of the terpyridine becoming more electron rich as a result of the electron-donating mesomeric effect of the amine bridges. Overall, the far-red absorption of complex [**6**]^2+^ seems to result from the electronic effect of the amine bridges, which destabilize the t_2g_ orbitals more than the e_g_* orbitals, thereby lowering the ^3^MLCT state and red-shifting the ^1^MLCT transitions in the absorption spectrum. In addition, the strong distortion of the coordination octahedron due to the pentadentate nature of the baptpy ligand keeps the ^3^MC low enough to be thermally promoted from such low-lying ^3^MLCT states, thereby allowing photosubstitution to take place with red- and far-red light irradiation.

**Table 3:** DFT and TDDFT Modeling^a^

**Compound**	**Orbital**	**Energy**, **eV**	**Transition energies**, **nm**	**Transition energies**, **eV**	***f***
[Ru(tpy)(Py)_3_]^2+^	LUMO	–2.603	513.63	2.414	0.0144
			512.14	2.421	0.0312
	HOMO	–5.944	469.72	2.640	0.0470
			445.71	2.782	0.0017
[Ru(baptpy)(Py)]^2+^	LUMO	–2.503	568.04	2.183	0.0583
			507.75	2.442	0.0123
	HOMO	–5.568	483.21	2.566	0.1141
			466.19	2.660	0.0242

a Franck–Condon transition energies were calculated by TDDFT on the ^1^GS geometry. Only transitions above 400 nm and with oscillator strength *f* > 0.02 are shown.

To further check these assumptions, we calculated the energies of the ^3^MLCT and ^3^MC states for both complexes and evaluated the interconversion energy barrier using nudged elastic band calculations (Table 4 and Table S19). The ^3^MLCT state for [**6**]^2+^ was calculated at a very low 158.3 kJ/mol energy level above the ground state, while for [Ru­(tpy)­(Py)_3_]^2+^, the ^3^MLCT state lay significantly higher at 189.8 kJ/mol. This much lower ^3^MLCT energy level aligns with the observation that [**6**]^2+^ has red-shifted absorption from the ground state to the ^1^MLCT state, compared with [Ru­(tpy)­(Py)_3_]^2+^.^23^ For [Ru­(tpy)­(Py)_3_]^2+^, two ^3^MC states were identified, which can be reached from the ^3^MLCT with an energy barrier of 21.6 and 11.2 kJ/mol, respectively (Figure S246). These barriers were determined by calculating the energy difference between the ^3^MLCT state and the highest-energy point (state X) along the nudged elastic band (NEB) path connecting the ^3^MLCT and ^3^MC states. The former corresponded to dissociation of one of the terminal pyridines of the terpyridine chelate, which is not experimentally observable, but the latter corresponded to the dissociation of an axial pyridine ligand, which is the photoreaction observed experimentally (Figure S247), and featured the lowest energy barrier. Notably, no ^3^MC states involving the dissociation of the equatorial ligand were found, which is consistent with experimental observations. For [**6**]^2+^, two ^3^MC states were identified as well (Figure S248), but both were found below the ^3^MLCT state in energy, as predicted by the high LSE (see above). The ^3^MC-1 state is only 2.41 kJ/mol lower, making it nearly isoenergetic with the ^3^MLCT state, while the ^3^MC-2 state is slightly more stable and 4.15 kJ/mol below the ^3^MLCT (Figure S249). The lower energy difference ΔE­(^3^MC – ^3^MLCT) found for [**6**]^2+^, compared with that for [Ru­(tpy)­(Py)_3_]^2+^, suggests that interconversion from ^3^MLCT to the ^3^MC state is more thermodynamically favorable in [**6**]^2+^. Two nearly isoenergetic dissociative reaction pathways were identified (Figure S248): the first pathway begins from the ^3^MLCT state and transitions to the ^3^MC-1 state, overcoming an energy barrier of 32.2 kJ/mol. From the ^3^MC-1 state, it can further transition to the ^3^MC-2 state, crossing a thermal barrier of 6.83 kJ/mol. The second pathway progresses from the ^3^MLCT state directly to the ^3^MC-2 state, requiring an energy barrier of 27.5 kJ/mol. Both barriers are relatively low compared to the ^3^MLCT – ^3^MC barrier of similar compounds.^53^ At this level of theory, the combined kinetic accessibility and thermodynamic stability of the ^3^MC states, together with the low energy of the ^3^MLCT states, are consistent with the far-red light-induced substitution reactivity of [**6**]^2+^. Of course, more fine-tuned theoretical studies would be needed to compare the photoreactivity of the pyridine-based baptpy compounds such as [**6**]^2+^ to that of their analogues bound to thioethers, imidazoles, or nitriles. However, the qualitatively comparable photoreactivities observed experimentally for [**6**]^2+^-[**16**]^2+^ makes us hypothesize that the modeling realized for [**6**]^2+^ gives a reasonable picture of the general causes behind the low-energy light photoreactivity of baptpy-based ruthenium compounds.

**Table 4:** ^3^MLCT-^3^MC Internal Energy Differences of [Ru­(baptpy)­(Py)]^2+^ ^a^

**State**	**ΔE(State –** ^**1**^ **GS)**, kJ/mol	**ΔE(State –** ^**3**^ **MLCT)**, kJ/mol	**ΔE(X –** ^**3**^ **MLCT)**, kJ/mol
^3^MLCT	158.3	0	–
^3^MC-1	155.9	–2.41	32.2
^3^MC-2	151.7	–4.15	27.5

a The highest energetic point found along the nudged elastic band pathway going from the ^3^MLCT state to state X.

### Photocytotoxicity

To obtain a glimpse of how the low-energy light-induced reactivity of [**6**]­Cl_2_-[**15**]­(PF_6_)_2_ would translate in a biological environment, and as a proof-of-concept of their potential for PACT, we measured their cytotoxicity and that of the free drugs **L** in 2D monolayers of normoxic skin melanoma (A375) and glioblastoma (U-87MG) human cancer cell lines, in the dark and upon red light (630 nm) irradiation, using formerly developed metabolic activity assay with 3-(4,5-dimethylthiazol-2-yl)-2,5-diphenyltetrazolium bromide (MTT).^5,74^ Briefly, each experiment was conducted at 37 °C for 96 h and consisted of the following steps: cell seeding and culturing in 96-well plates for 24 h, incubation with the compound of interest for 24 h in the dark, irradiation with red (630 nm, 34.1 mW/cm^2^, 30 min, 61.4 J/cm^2^) or far-red (730 nm, 63.2 mW/cm^2^, 30 min, 114 J/cm^2^) light followed by incubation for another 47.5 h, and finally an MTT end-point cell viability assay. The resulting dose–response curves, independent *t* tests, and detailed information about cell culturing and the cytotoxicity assay can be found in the Supporting Information. The cell-growth inhibition half maximal effective concentration (EC_50_ in μM) and photoindexes (PI = EC_50,D_/EC_50,RL_) were determined and are summarized in Table 5 and Table 6.

**Table 5:** *In Vitro* Anticancer Efficacy of the Free Drugs in A375 and U-87MG Cancer Cell Lines under Normoxia^a^

**Compound**	**EC**_**50**_ **(±95% CI)** **in A375,** **μM**	**EC**_**50**_ **(±95% CI)** **in U-87MG,** **μM**
**STF-31**	0.69 (+0.11/–0.10)	24.0 (+6.38/–5.38)
RAD-51-IN-1	5.13 (+0.38/–0.36)	96.8 (+25.7/–18.1)
**QC-82**	93.3 (+25.6/–14.8)	>200
**Norharmane**	>100	>200
**Neratinib**	0.16 (+0.01/–0.01)	14.2 (+1.50)
**Bosutinib**	4.10 (+0.26/–0.25)	13.1 (+1.61)
**Ponatinib**	1.00 (+0.07/–0.06)	3.14 (+0.87)
**Albendazole**	0.51 (+0.10/–0.09)	87.5 (+21.2/–15.3)
**MTI**	0.16 (+0.02/–0.02)	1.22 (+4.06/–1.18)

a For U-87MG experiments the cell medium was refreshed with drug-free medium just before irradiation.

**Table 6:** *In Vitro* Anticancer Efficacy of [**6**]­Cl_2_-[**15**]­(PF_6_)_2_ in A375 and U-87MG Cancer Cell Lines in the Dark (D) and upon Red Light Activation (RL) under Normoxia^a^

**Prodrug**	**Caged moiety**	**Light**	**EC**_**50**_ **(±95% CI) in A375**, **μM**	**PI**	**EC**_**50**_ **(±95% CI) in U-87MG**, **μM**	**PI**
[**6**]Cl_2_	**Pyridine**	D	>100	–	>200	–
		RL	>100		>200	
[**7**]Cl_2_	**STF-31**	D	9.08 (+1.08/–0.97)	**7**.**5**	>200	**>1**.**4**
		RL	1.21 (+0.26/–0.22)		148 (+87.9/–41.3)	
[**8**]Cl_2_	RAD-51-IN-1	D	7.65 (+0.87/–0.78)	**1**.**6**	>200	**>4**.**5**
		RL	4.87 (+0.57/–0.51)		43.9 (+8.09/–7.00)	
[**9**]Cl_2_	**QC-82**	D	47.7 (+15.3/–10.2)	**1**.**0**	>200	**>1**.**2**
		RL	48.9 (+24.1/–13.7)		164 (+50.9/–29.2)	
[**10**]Cl_2_	**Norharmane**	D	12.2 (+2.63/–2.18)	**1**.**5**	>200	**>3**.**6**
		RL	8.04 (+2.28/–1.77)		55.9 (+16.5/–13.2)	
[**11**]Cl_2_	**Neratinib**	D	1.28 (+0.08/–0.07)	**2**.**3**	15.2 (+1.69)	**1**.**0**
		RL	0.55 (+0.04/–0.04)		15.2	
[**12**]Cl_2_	**Bosutinib**	D	11.9 (+1.49/–1.32)	**5**.**7**	84.6 (+16.3/–13.1)	**4**.**4**
		RL	2.08 (+0.51/–0.41)		19.3 (+3.99/–3.42)	
[**13**]Cl_2_	**Ponatinib**	D	1.04 (+0.06/–0.06)	**0**.**9**	7.25 (+2.27/–2.11)	**>2**.**2**
		RL	1.16 (+0.07/–0.06)		3.34	
[**14**](PF_6_)_2_	**Albendazole**	D	7.45 (+2.07/–1.63)	**2**.**8**	>200	**>1**.**6**
		RL	2.68 (+0.66/–0.53)		129 (+91.9/–40.4)	
[**15**](PF_6_)_2_	**MTI**	D	5.22 (+1.31/–1.04)	**6**.**1**	>200	**–**
		RL	0.86 (+0.16/–0.14)		>200	

a Photoindexes calculated as PI = EC_50 D_/EC_50 RL_. Irradiation was performed using 630 nm red light LED array, 34.1 mW/cm^2^, 30 min, light dose 61.4 J/cm^2^. For U-87MG experiments the cell medium was refreshed with drug-free medium just before irradiation.

Most of the tested free drugs (**L**) exhibited cytotoxicity ranging from micro- to nanomolar concentrations in the cancer cells used. Among them, **Neratinib**, **Ponatinib**, and **MTI** were the most potent, whereas **Norharmane** and **QC-82** were the least potent (Table 5). As for the ruthenium-based complexes (Table 6), [**6**]­Cl_2_ showed reasonably low toxicity in both A375 and U-87MG cancer cell lines, suggesting that the new [Ru­(baptpy)­(**L**)]^2+^ photocage is a safe drug carrier. Among the tested compounds, a few were found to have very good PACT properties, such as complexes [**7**]­Cl_2_, [**12**]­Cl_2_, and [**15**]­(PF_6_)_2_, whose photoindexes (PI) under red light were up to 7.5, with EC_50,RL_ values that were nearly as low as the EC_50_ of the corresponding free inhibitors. Importantly, complex [**7**]­Cl_2_ was also able to demonstrate far-red light activation at 730 nm in A375 skin cancer cells with EC_50,FRL_ = 1.5 μM for EC_50,D_ = 9.3 μM, which corresponded to a PI value of 6.1 (Figure 7). As a note, this molecule is incapable of generating reactive oxygen species (ROS) such as singlet oxygen; hence, we see far-red light-activated PACT here without contribution of any photodynamic effect, which is rare.

**Figure 7:**
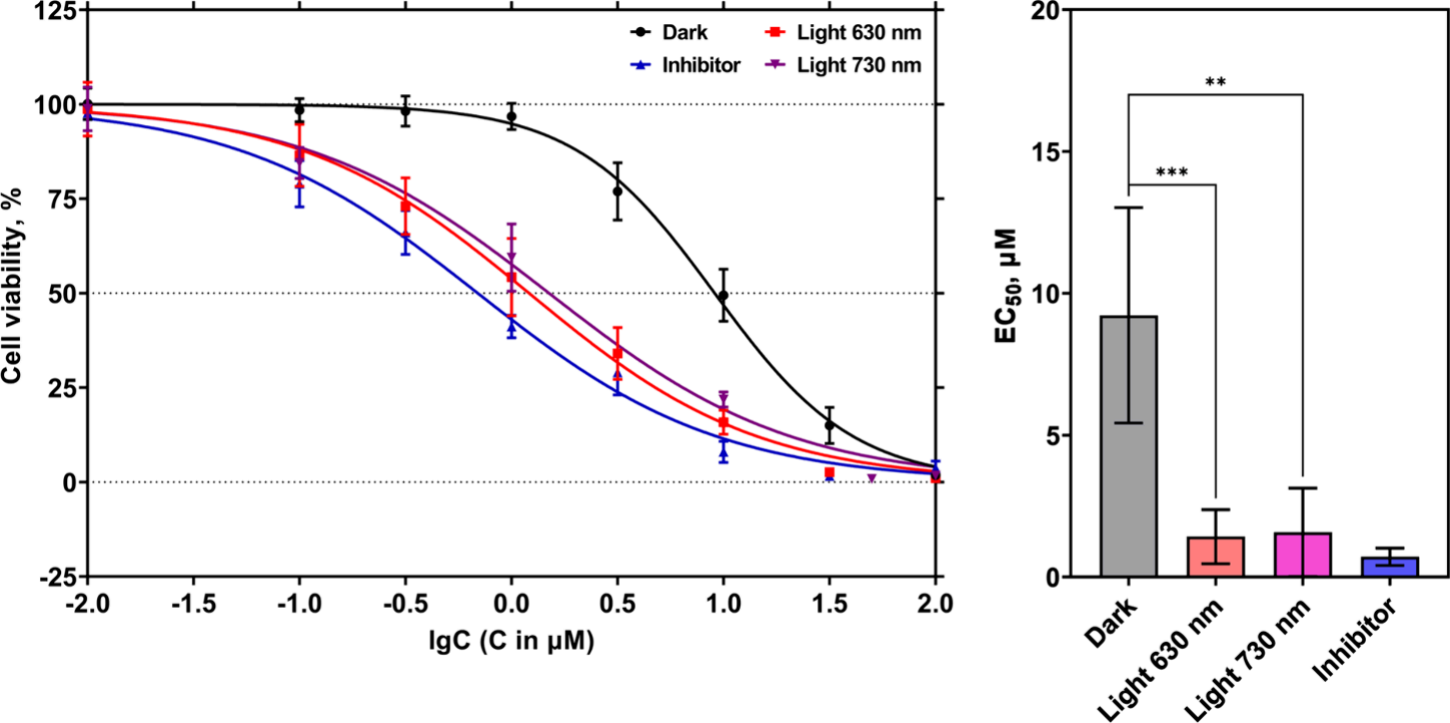
Dose response curves (left) and respective EC_50_ values (right) for **STF-31** itself (blue curve) and [**7**]­Cl_2_ in the dark (black curve) and upon 630 nm (red curve; 34.1 mW/cm^2^, 30 min, 61.4 J/cm^2^) or 730 nm (purple curve; 63.2 mW/cm^2^, 30 min, 114 J/cm^2^) light irradiation in the A375 skin melanoma cell line. The values are expressed as the mean ± standard error of the mean (SEM). Statistical significance was evaluated by the two-tailed unpaired Student’s *t-*test and expressed as follows: (ns) *p* > 0.05, (*) *p* ≤ 0.05, (**) *p* ≤ 0.01, (***) *p* ≤ 0.001, (****) *p* ≤ 0.0001.

For four cytotoxicity experiments in a series of eleven, i.e., for **Neratinib** (0.55 vs 0.16 μM), **Albendazole** (2.68 vs 0.51 μM), and **MTI** (0.86 vs 0.16 μM) with red light activation and for **STF-31** with far-red light activation (1.5 vs 0.69 μM), the EC_50_ value of the light-activated prodrug in A375 cells was higher than that of the free inhibitor they release. Though the differences were not massive, two hypotheses may be proposed to explain these observations. Possibly, the light dose chosen for this study may have been too low to activate 100% of the complex. For far-red light irradiation of [**7**]­Cl_2_ at 114 J/cm^2^ for example, HPLC indeed demonstrated that the light dose led to only partial release of **STF-31** (Figure S244): some of the starting reagent was still visible at the end of the 30 min irradiation time (t = 12.1 min), together with the peak of the free **STF-31** ligand (11.0 min). At such high wavelengths, there is hence room for improvement of the light dose (or of the photoreactivity) if we want to activate 100% of the complex. For red light activation, our HPLC studies showed in all three cases highlighted above that the light dose of 61.4 J/cm^2^ was high enough to release 100% of the caged inhibitor (see Figures S238, S241, and S242, respectively). Another hypothesis for the lowest activity of the red light-activated prodrug compared with that of the free inhibitor can be made: that the ruthenium cage exerts, after splitting of the molecule in two, an antagonistic effect on the biological action of the uncaged inhibitor. Characterizing the biological action of a photocage and its influence on the biological properties of an inhibitor is nontrivial; we have studied it in a recent paper dedicated to the photocaged **STF-31** compound [Ru­(tpy)­(biq)­(**STF-31**)]^2+^ using cytotoxicity studies, metabolomics, and rescue experiments.^48^ The differences in EC_50_ values between the red light-activated prodrugs [**11**]­Cl_2_, [**14**]­(PF_6_)_2_ and [**15**]­(PF_6_)_2_ and their free inhibitor were not high enough to justify such detailed studies here; however, such studies would be necessary as soon as more detailed biological studies of a single compound in a particular form of cancer would be undertaken.

Two additional comments about the cytotoxicity results should be made here. On the one hand, the light dose was kept identical in the whole cytotoxicity study to compare different compounds in identical conditions. This is justified on the biological point of view, but not photochemically: the photosubstitution quantum yields of the different compounds in this series of PACT prodrugs vary very much, from low (0.0008 for [**9**]­Cl_2_ with red light) to high values (0.100 for [**15**]­(PF_6_)_2_). Another possible approach to a cytotoxicity experiment would be to apply a higher light dose for the slowest compounds and a lower light dose for the fastest compounds; then of course, at the cost of comparability. These differences in photoreactivity can be retrieved from our HPLC analysis of the photosubstitution reaction in cell growing media, which was performed in 96-well plates using the same light irradiation conditions as the one used for cytotoxicity assays (61.4 J/cm^2^ for 630 nm, and 114 J/cm^2^ for 730 nm). According to Figure S234 for example, [**7**]­Cl_2_ after red light irradiation showed full disappearance of the reagent and appearance of the free ligand **STF-31** at 11.0 min. In the red-light cytotoxicity experiment for this compound, full photoconversion was hence achieved, while for far-red activation it was not (see above). Not all prodrugs released 100% of their loading with the red-light irradiation conditions used, however: according to HPLC [**9**]­Cl_2_, [**10**]­Cl_2_, [**13**]­Cl_2_ and [**16**]­Cl_2_ were found to be only partially cleaved (Figure S236, S237, S240 and S243), which fits with the low photosubstitution quantum yields of these compounds (Table 1). As noted, for [**9**]­Cl_2_ and [**10**]­Cl_2_ the low photoindexes found in cancer cells are probably independent from the fraction of released ligand, as free **QC-82** and **Norharmane** are poorly toxic anyhow.

On the other hand, the toxicity of several ruthenium conjugates in the series was surprisingly high in the dark, sometimes reaching the same value as that of the free drug (e.g., [**13**]­Cl_2_ in A375 or [**11**]­Cl_2_ in U-87MG cells). These results align surprisingly well with the poor thermal stability recorded by HPLC for [**13**]­Cl_2_ and [**11**]­Cl_2_ in aqueous solutions, which suggests that these compounds might either release their ligand thermally or transform into cytotoxic secondary metabolites. A second hypothesis for such high dark toxicity is that changing the delicate chemical architecture of an organic inhibitor with a big, charged ruthenium end-cap does not necessarily provide sufficient protection toward the biological targets of the ligand, in particular if it binds to its target protein far away from the heteroatom coordinated to ruthenium.^75^ In such a case, although the ligand is technically “caged”, the prodrug may still be able to bind to the target protein of the free ligand and hence be quite toxic. Finally, unexpected off-targets might be hit, such as the negatively charged mitochondrial membrane that may be sensitive to greasy, positively charged ruthenium­(II) prodrugs.^76,77^ Considering the large number of Ru compounds prepared in this study, it is impossible at this stage to provide an extensive mode-of-action study for all PACT compounds made. Better understanding of how they work or why some do not work in different cell lines will come from future, more in-depth biological studies. Overall, and despite some irregularities the photobiological effects of the prodrugs [**7**]­Cl_2_-[**15**]­(PF_6_)_2_ in A375 and U-87MG cell lines mostly mirrored their photochemical reactivity, with a higher toxicity following low-energy light activation.

## Conclusions

In this work, we presented a new photoresponsive, ruthenium-based scaffold [Ru­(baptpy)­(**L**)]^2+^ based on the pentapyridyl ligand baptpy and its application for the photocaging of a wide range of anticancer drugs. This motif was shown to bind multiple chemical functionalities such as primary amines, thioethers, nitriles, pyridines, imidazoles, and to retain them until light exposure. Their release was triggered by red (625 nm) and far-red (730 nm) light with good to excellent quantum yields, which is a highly desired property in PACT prodrugs. Naturally, it is too labor- and resource-intensive to investigate the biological properties of all 10 light-activated compounds in detail. We are therefore concentrating on a selected subset, and the results of these ongoing studies will be reported in due course. However, based on preliminary cytotoxicity studies performed in this work, several important lessons were learned. First, in several cases, the biological activity of the drug **L** was not entirely suppressed by coordination to the ruthenium cage. One obvious possibility to alleviate this problem is to introduce alternative metal-binding sites on the drug to change the position of the ruthenium fragment and improve photocaging; however, this strategy requires changing the drug and hence rerunning the whole drug development path. Another strategy is to run a wider screening of drug analogues targeted at the same protein target but that lead to better photocaging and higher photoindexes. Finally, functionalization of the spectator baptpy ligand with water-solubilizing, cancer-targeting, or electronically active substituents may also tune the photocaging ability of [Ru­(baptpy)­(L)]^2+^ by lowering the dark toxicity of the prodrug without having to modify the chosen drug. Another challenge to overcome in PACT is to maintain a good thermal stability of the prodrug, which varies considerably across different coordinating functionalities. For example, the inability of [Ru­(baptpy)­(**Gemcitabine**)]­Cl_2_ to retain its molecular integrity in the dark in acetonitrile calls for more research efforts into photocaging of primary amines, which so far was only convincingly achieved for the green light-activated complexes [Ru­(bpy)_2_(PR_3_)­(**L**)]^2+^ (R=Me or Ph) and [Ru­(bpy)_2_(**L**)_2_]^2+^.^78,79^ Such advancement may allow to photocage **Lenalidomide** and **Ibrutinib**, which are other examples of essential anticancer medicines possessing an amine group. Altogether, the new photocage presented in this work demonstrates that potent photoreactive compounds can be developed that are activated by low-energy light toward targeted cancer treatment with a wide range of structurally diverse anticancer agents.

## Supplementary Material





## Notes

Materials, synthesis and characterization data including NMR, mass spectrometry, elemental analysis, molar absorption coefficients and single-crystal X-ray diffraction; photochemistry data such as photosubstitution, phosphorescence and singlet oxygen quantum yields photocytotoxicity data and DFT calculations (PDF)
XRD data (ZIP)
