# Supplementary material for: A Sterically Open Ruthenium-Based Photocage Activated by Red and Far-Red Light for a Wide Range of Drugs
Source: J Am Chem Soc. 2025 Nov 18;147(48):44356–71. doi: 10.1021/jacs.5c14772 (PMC12679631; doi:10.1021/jacs.5c14772)
Supplement: Supplementary file 1 [file ja5c14772_si_001.pdf]

Supporting Information

for

A sterically open ruthenium-based photocage activated by red and far-red light for a wide range of drugs

Yurii Husiev,[a] Sina Katharina Götzfried,[a] Matthijs L. A. Hakkennes,[a] Daria Kotova,[a] Isabelle Tutein,[a] Corjan van de Griend,[a] Andrew C. Johns,[c] Selda Abyar,[a] Maxime A. Siegler,[b] Alexander Kornienko,[c] and Sylvestre Bonnet[a]*

[a] Leiden Institute of Chemistry, Universiteit Leiden, Einsteinweg 55, 2333 CC, Leiden, Netherlands.
[b] Department of Chemistry, Johns Hopkins University, 3400 N Charles St., Baltimore, MD, 21218, USA.
[c] Department of Chemistry and Biochemistry, Texas State University, 601 University Dr., San Marcos, TX, 78666, USA.

*Corresponding author email: bonnet@chem.leidenuniv.nl

Table of Contents

1 SYNTHESIS AND CHARACTERIZATION 3

1.1 General information 3

1.2 Synthesis of ligand 3

1.3 Synthesis of complexes 4

1.4 NMR data 12

1.5 Mass spectrometry data 60

1.6 Single crystal X-ray crystallography 74

2 PHOTOCHEMISTRY 90

2.1 General information 90

2.2 Molar absorption coefficients 90

2.3 Ligand photosubstitution monitored by UV-Vis 95

2.4 Stability in water monitored by UV-Vis and HPLC 106

2.5 Emission spectra of the light sources used 117

2.6 Ligand photosubstitution monitored by mass spectrometry 117

2.7 Phosphorescence data 121

2.8 Singlet oxygen measurement 122

3 PHOTOCYTOTOXICITY 124

3.1 General information 124

3.2 Cell culturing 124

3.3 Cytotoxicity assay 124

4 PHOTOSUBSTITUTION IN OPTI-MEM COMPLETE 135

5 DFT STUDIES 148

6 REFERENCES 151

1 Synthesis and characterization

1.1 General information

Unless otherwise noted, the reactions were carried under air at room temperature (r.t.) using common laboratory glass equipment. All reagents and solvents were purchased from commercial suppliers (BLDPharm, AmBeed, Fluorochem, Sigma-Aldrich, VWR and TCI) and used as obtained. The standard Schenk technique was used to carry reactions under N_2 atmosphere when needed. Thin layer chromatography (TLC) was performed using Supelco analytical silica gel on aluminum foil with fluorescent indicator 254 nm. Column chromatography was carried out using either silica gel (40-63 μm) or Sephadex LH-20 for size exclusion (SEC), and driven by pressurized air. The columns were packed using the slurry method and the compounds were introduced in form of either a solution or as a solid mixture with silica gel. The filtrations were done using 25 mm glass vacuum filter from Sartorius equipped with RC60 membrane from Cytiva. Nuclear magnetic resonance (NMR) spectra were recorded on Bruker Avance 300, 400 or 500 MHz and processed with MestReNova software. The chemical shifts are given relative to the residual signal of the solvent ($CDCl_3$: δ(^{1}H) = 7.26; δ(^{13}C) = 77.16; DMSO-*d*$_6$: δ(^{1}H) = 2.50; δ(^{13}C) = 39.52; CD_3OD: δ(^{1}H) = 3.31; δ(^{13}C) = 49.00; Acetone-*d*$_6$: δ(^{1}H) = 2.05; δ(^{13}C) = 29.84 and 206.26; CD_3CN: δ(^{1}H) = 1.94; δ(^{13}C) = 1.32 and 118.26).[1] The regular mass spectra (ESI-MS) were recorded on Shimadzu LCMS-2020 (ESI-Q). The high-resolution mass spectra (HRMS) were recorded on Thermo Finnigan LTQ Orbitrap. Elemental analyses were performed by Mikroanalytisches Laboratorium Kolbe (MIKROLAB). The **STF-31**,[2] **QC-82**,[3] **RAD-51-IN-1**,[4] **MTI**[5], **Entinostat**[6] and **NSC745885**[7] were synthesized by formerly reported procedures, while the other drugs were purchased from mentioned commercial suppliers, NMR characterization data is provided.

1.2 Synthesis of ligand

*Synthesis of [2,2':6',2''-terpyridine]-1,1''-dioxide (**1**)*

2,2':6',2''-Terpyridine (10.00 g, 41.60 mmol, 1 eq) and *m*-CPBA (22.55 g (70% pure), 91.00 mmol, 2.2 eq) were mixed in DCM (416 mL) and stirred for 24 h at r.t. The resulting suspension was quenched with saturated aqueous Na_2CO_3 (50 mL) and concentrated *in vacuo*. The obtained residue was then crushed into powder and refluxed for 15 min with $CHCl_3$ (250 mL) and a few spoons of anhydrous Na_2SO_4. The precipitate was then filtered, washed with hot $CHCl_3$ (250 mL) and the filtrate was concentrated *in vacuo* to afford target compound as white powder (11.03 g, 41.60 mmol, 100%). Characterization was consistent with previous reports.[8] **TLC** ($CHCl_3$/MeOH = 8/1, product R_f = 0.51). **^{1}H NMR** (500 MHz, $CDCl_3$) δ 8.94 (d, *J* = 8.0 Hz, 2H), 8.33 (ddd, *J* = 6.5, 1.3, 0.6 Hz, 2H), 8.19 (ddd, *J* = 8.0, 2.2, 0.6 Hz, 2H), 7.97 (t, *J* = 8.0 Hz, 1H), 7.37 (ddd, *J* = 8.0, 7.5, 1.3 Hz, 2H), 7.29 (ddd, *J* = 7.5, 6.5, 2.2 Hz, 2H). **^{13}C NMR** (126 MHz, $CDCl_3$) δ 149.51, 147.31, 140.88, 136.78, 128.09, 126.20, 125.76, 125.50. **ESI-MS**: exact m/z calculated for $[C_{15}H_{11}N_3O_2+H]^+$: 266.1, found: 266.1. **HRMS**: exact m/z calculated for $[C_{15}H_{11}N_3O_2+H]^+$: 266.0924, found: 266.0923.

*Synthesis of [2,2':6',2''-terpyridine]-6,6''-diamine (**2**)*

[2,2':6',2''-Terpyridine]-1,1''-dioxide (11.03 g, 41.60 mmol, 1 eq), potassium phthalimide (38.50 g, 208.00 mmol, 5 eq) and triethylamine (29.00 mL, d = 0.73 g/mL, 208.00 mmol, 5 eq) were mixed together in ACN (832 mL) followed by careful addition of solid TsCl

(39.60 g, 208.00 mmol, 5 eq). The obtained mixture was stirred for 24 h at r.t. until reaction completion. The resulting suspension was filtered and the filter cake was washed with ACN (100 mL) and hot demi water (400 mL). The solid residue was then mixed with aqueous 80% hydrazine hydrate solution (25.67 mL, d = 1.02 g/ml, 416.0 mmol, 10 eq) diluted with water (208 mL) and the mixture was stirred at 80 °C for another 24 h. The obtained suspension was further diluted with demi water (200 mL) followed by hot filtration. The filter cake was washed with plenty of hot water (200 mL) until all the side products were gone. Drying *in vacuo* afforded target compound as white powder (10.28 g, 39.00 mmol, 94%). Additionally, the compound can be purified through recrystallization from acetone/hexane. Characterization was consistent with previous reports.[8] **TLC** ($CHCl_3$/MeOH = 2/1, product R_f = 0.75, blue fluorescent spot under UV lamp). **^{1}H NMR** (300 MHz, DMSO-d_6) δ 8.23 (d, J = 7.8 Hz, 2H), 7.97 (t, J = 7.8 Hz, 1H), 7.72 (dd, J = 7.4, 0.9 Hz, 2H), 7.55 (t, J = 7.8 Hz, 2H), 6.53 (dd, J = 8.1, 0.9 Hz, 2H), 6.05 (s, 4H). **^{13}C NMR** (75 MHz, DMSO-d_6) δ 159.34, 155.23, 153.48, 137.96, 137.59, 119.66, 108.89, 108.79. **ESI-MS**: exact m/z calculated for $[C_{15}H_{13}N_5+H]^+$: 264.1, found: 264.2; exact m/z calculated for $[2C_{15}H_{13}N_5+Na]^+$: 549.2, found: 549.2. **HRMS**: exact m/z calculated for $[C_{15}H_{13}N_5+H]^+$: 264.1244, found: 264.1241.

*Synthesis of N^6,$N^{6''}$-di(pyridin-2-yl)-[2,2':6',2''-terpyridine]-6,6''-diamine (**3**; baptpy)*

[2,2':6',2''-Terpyridine]-6,6''-diamine (3.00 g, 11.39 mmol, 1 eq), 2-bromopyridine (2.28 mL, d = 1.66 g/mL, 23.93 mmol, 2.1 eq), $Pd(dba)_2$ (0.33 g, 0.57 mmol, 0.05 eq), rac-BINAP (0.36 g, 0.57 mmol, 0.05 eq), and *t*-BuOK (3.84 g, 34.20 mmol, 3 eq) were mixed together in dried and deoxygenated toluene (142 mL) and stirred for 48 h at 110 °C under N_2 atmosphere. Upon reaction completion the mixture was concentrated *in vacuo* and the residue was refluxed for 30 min in MeOH/H_2O = 4/1 (300 mL) followed by hot filtration. The filter cake was washed with acetone (50 mL) and Et_2O (50 mL) until all the side products were gone. Drying *in vacuo* afforded target compound as beige powder (3.93 g, 9.41 mmol, 83%). **TLC** ($CHCl_3$/MeOH = 8/1, product R_f = 0.05). **^{1}H NMR** (400 MHz, DMSO-d_6) δ 9.83 (s, 2H), 8.39 (d, J = 7.8 Hz, 2H), 8.27 (ddd, J = 4.9, 2.0, 0.8 Hz, 2H), 8.16 (t, J = 7.8 Hz, 1H), 8.11 (dd, J = 7.4, 0.9 Hz, 2H), 7.98 (d, J = 8.4 Hz, 2H), 7.87 (t, J = 7.9 Hz, 2H), 7.79 – 7.71 (m, 4H), 6.92 (ddd, J = 7.2, 4.9, 1.0 Hz, 2H). **^{13}C NMR** (101 MHz, DMSO-d_6) δ 155.04, 154.31, 154.03, 153.18, 147.61, 138.70, 138.47, 137.79, 120.39, 116.20, 112.87, 112.58, 111.79. **ESI-MS**: exact m/z calculated for $[C_{25}H_{19}N_7+H]^+$: 418.2, found: 418.1; exact m/z calculated for $[C_{25}H_{19}N_7+Na]^+$: 440.2, found: 440.1. **HRMS**: exact m/z calculated for $[C_{25}H_{19}N_7+H]^+$: 418.1775, found: 418.1769; exact m/z calculated for $[C_{25}H_{19}N_7+2H]^{2+}$: 209.5924, found: 209.5922. **Elemental analysis** calculated for $C_{25}H_{19}N_7$ (%): C, 71.93; H, 4.59; N, 23.49; found: C, 70.46; H, 4.53; N, 22.66.

1.3 Synthesis of complexes

*Synthesis of [Ru(baptpy)Cl]Cl ([**4**]Cl)*

[Ru(*p*-cymene)Cl_2]$_2$ (1.47 g, 2.40 mmol, 1 eq) and baptpy (2.00 g, 4.79 mmol, 2 eq) were mixed together in deoxygenated MeOH (96 mL) and stirred for 24 h at 65 °C under N_2 atmosphere. Upon reaction completion the violet solution was slightly concentrated *in vacuo* and then quenched with Et_2O (300 mL) to afford fine precipitate. The resulting solid was filtered, washed with $CHCl_3$ (50 mL) and Et_2O (50 mL) until all the side products were gone. Drying *in vacuo* afforded target compound as violet powder (2.58 g, 4.38 mmol, 91%). **^{1}H NMR** (500 MHz, DMSO-d_6) δ 11.46 (s, 1H), 10.65 (s, 1H), 9.79 (d, J = 5.1 Hz, 1H), 8.60 (dd, J = 8.1, 0.8 Hz, 1H), 8.50 (ddd, J = 7.8, 2.8, 0.9 Hz, 2H), 8.19 – 8.10 (m, 2H), 7.93 – 7.86

(m, 2H), 7.84 (ddd, *J* = 8.6, 7.1, 1.5 Hz, 1H), 7.76 (d, *J* = 8.3 Hz, 1H), 7.47 – 7.39 (m, 2H), 7.34 (dd, *J* = 8.0, 0.9 Hz, 1H), 7.06 (ddd, *J* = 7.1, 5.8, 1.2 Hz, 1H), 6.85 (dd, *J* = 8.3, 1.3 Hz, 1H), 6.52 (ddd, *J* = 7.1, 5.7, 1.3 Hz, 1H), 6.26 – 6.18 (m, 1H). **^{13}C NMR** (126 MHz, DMSO-*d$_6$*) δ 159.74, 159.59, 158.79, 157.43, 157.06, 156.82, 155.35, 151.08, 149.87, 149.53, 138.83, 137.71, 137.65, 135.50, 131.98, 121.48, 120.90, 119.20, 118.18, 118.02, 115.06, 114.57, 113.66, 112.60. **ESI-MS**: exact m/z calculated for [$C_{25}H_{19}Cl_2N_7Ru$–Cl]$^+$: 554.0, found: 554.0. **HRMS**: exact m/z calculated for [$C_{25}H_{19}Cl_2N_7Ru$–Cl]$^+$: 554.0432, found: 554.0427. **Elemental analysis** calculated for $C_{25}H_{19}Cl_2N_7Ru$ (%): C, 50.94; H, 3.25; N, 16.63; found: C, 50.35; H, 3.23; N, 16.41.

*Synthesis of [Ru(baptpy)(H$_2$O)](PF$_6$)$_2$ ([**5**](PF$_6$)$_2$)*

[Ru(baptpy)Cl]Cl (0.30 g, 0.51 mmol, 1 eq) and $AgPF_6$ (0.27 g, 1.07 mmol, 2.1 eq) were mixed together in deoxygenated acetone/H_2O = 1/1 (5 mL) and stirred for 24 h at 50 °C under N_2 atmosphere in dark. Upon reaction completion the violet mixture was filtered, the filter cake was washed with acetone (20 mL) and the filtrate was concentrated *in vacuo*. Purification by SEC in Acetone afforded target compound as violet powder (0.24 g, 0.29 mmol, 57%). Due to poor water solubility and quick ligand exchange it undergoes with most solvents the NMR characterization was done for CD_3CN complex. **^{1}H NMR** (400 MHz, CD_3CN) δ 9.49 (dd, *J* = 5.9, 1.7 Hz, 1H), 9.28 (s, 1H), 8.64 (s, 1H), 8.44 (dd, *J* = 8.1, 0.8 Hz, 1H), 8.33 (td, *J* = 7.8, 0.8 Hz, 2H), 8.21 (dd, *J* = 8.4, 7.7 Hz, 1H), 8.07 (t, *J* = 8.1 Hz, 1H), 8.03 – 7.94 (m, 2H), 7.85 (ddd, *J* = 8.7, 7.2, 1.8 Hz, 1H), 7.55 (dd, *J* = 8.5, 0.9 Hz, 1H), 7.47 (ddd, *J* = 8.3, 7.3, 1.7 Hz, 1H), 7.43 – 7.36 (m, 1H), 7.19 (dt, *J* = 8.5, 1.0 Hz, 1H), 7.07 (ddd, *J* = 7.1, 5.9, 1.2 Hz, 1H), 6.81 (dt, *J* = 8.4, 1.0 Hz, 1H), 6.59 (ddd, *J* = 7.3, 5.8, 1.2 Hz, 1H), 6.47 (dd, *J* = 5.8, 1.7 Hz, 1H). **^{13}C NMR** (101 MHz, CH_3CN) δ 159.74, 159.68, 159.07, 158.77, 157.61, 157.22, 156.10, 151.68, 151.10, 140.67, 140.31, 139.87, 138.95, 135.98, 123.52, 123.24, 120.97, 119.89, 119.68, 117.32, 117.12, 116.18, 115.98, 115.61. **^{31}P NMR** (162 MHz, CD_3CN) δ -146.76 (hept, 2P, *J* = 707.5 Hz). **^{19}F NMR** (376 MHz, CD_3CN) δ -72.77 (d, *J* = 707.4 Hz, 12F). **ESI-MS**: exact m/z calculated for [$C_{25}H_{21}F_{12}N_7OP_2Ru$–$H_2O$+MeOH–$HPF_6$–$PF_6$]$^+$: 550.1, found: 550.2; exact m/z calculated for [$C_{25}H_{21}F_{12}N_7OP_2Ru$–$H_2O$+MeOH–2$PF_6$]$^{2+}$: 275.5, found: 275.1. **HRMS**: exact m/z calculated for [$C_{25}H_{21}F_{12}N_7OP_2Ru$–$H_2O$+ACN–$HPF_6$–$PF_6$]$^+$: 559.0934, found: 559.0935. **Elemental analysis** calculated for $C_{37}H_{34}F_{12}N_{10}O_2P_2RuS$ (%): C, 41.39; H, 3.19; N, 13.04; found: C, 39.14; H, 3.01; N, 12.71.

*Synthesis of [Ru(baptpy)(Py)]Cl$_2$ ([**6**]Cl$_2$)*

[Ru(baptpy)Cl]Cl (0.30 g, 0.51 mmol, 1 eq), pyridine (0.21 mL, d = 0.98 g/mL, 2.54 mmol, 5 eq), and $AgPF_6$ (0.27 g, 1.07 mmol, 2.1 eq) were mixed together in deoxygenated acetone/H_2O = 1/1 (5 mL) and stirred for 24 h at 50 °C under N_2 atmosphere in dark. Upon reaction completion the violet mixture was filtered, the filter cake was washed with acetone (20 mL) and the filtrate was concentrated *in vacuo*. The obtained residue was dissolved in minimal quantity of acetone and saturated tetrabutylammonium chloride (1.41 g, 5.09 mmol, 10 eq) acetone solution was added dropwise to form fine precipitate. The resulting solid was filtered, washed with acetone (5 mL) and Et_2O (50 mL) until all the side products are gone. Reprecipitation from MeOH/Et_2O followed by filtration and drying *in vacuo* afforded target compound as violet powder (0.25 g, 0.37 mmol, 74%). **^{1}H NMR** (300 MHz, CD_3OD) δ 9.80 (dd, *J* = 6.0, 1.7 Hz, 1H), 8.60 (dd, *J* = 8.1, 0.8 Hz, 1H), 8.56 (dd, *J* = 7.7, 1.0 Hz, 1H), 8.40 – 8.30 (m, 2H), 8.16 – 8.05 (m, 2H), 7.96 (t, *J* = 8.1 Hz, 1H), 7.82 (ddd, *J* = 8.8, 7.2, 1.8 Hz, 1H), 7.74 (dd, *J* = 8.5, 0.9 Hz, 1H), 7.62 – 7.40 (m, 5H), 7.29 – 7.20 (m, 1H), 7.02 (dddd, *J* = 9.5, 6.6, 5.6, 1.4 Hz, 3H), 6.93 – 6.83 (m, 1H), 6.66 (ddd, *J* = 7.2, 5.8, 1.3 Hz, 1H), 6.52 – 6.40 (m,

1H). **^{13}C NMR** (75 MHz, CD_3OD) δ 159.63, 159.41, 159.12, 157.87, 156.45, 154.08, 153.47, 152.04, 151.51, 141.01, 140.64, 139.97, 139.05, 138.82, 135.45, 127.43, 126.93, 123.72, 123.60, 121.19, 120.56, 117.95, 117.47, 116.58, 116.15, 115.54. **ESI-MS**: exact m/z calculated for $[C_{30}H_{24}Cl_2N_8Ru–HCl–Cl]^+$: 597.1, found: 597.0; exact m/z calculated for $[C_{30}H_{24}Cl_2N_8Ru–2Cl]^{2+}$: 299.0, found: 298.5. **HRMS**: exact m/z calculated for $[C_{30}H_{24}Cl_2N_8Ru–HCl–Cl]^+$: 597.1092, found: 597.1084. **Elemental analysis** calculated for $C_{30}H_{24}Cl_2N_8Ru$ (%): C, 53.90; H, 3.62; N, 16.76; found: C, 52.78; H, 3.59; N, 16.48.

*Synthesis of [Ru(baptpy)(**STF-31**)]Cl_2 ([**7**]Cl_2)*

[Ru(baptpy)Cl]Cl (0.30 g, 0.51 mmol, 1 eq), **STF-31** (0.24 g, 0.56 mmol, 1.1 eq), and $AgPF_6$ (0.27 g, 1.07 mmol, 2.1 eq) were mixed together in deoxygenated acetone/H_2O = 1/1 (5 mL) and stirred for 24 h at 50 °C under N_2 atmosphere in dark. Upon reaction completion the purple mixture was filtered, the filter cake was washed with acetone (20 mL) and the filtrate was concentrated *in vacuo*. The obtained residue was dissolved in minimal quantity of acetone and saturated tetrabutylammonium chloride (1.41 g, 5.09 mmol, 10 eq) acetone solution was added dropwise to form fine precipitate. The resulting solid was filtered, washed with acetone (5 mL) and Et_2O (50 mL) until all the side products are gone. Reprecipitation from MeOH/Et_2O followed by filtration and drying *in vacuo* afforded target compound as purple powder (0.40 g, 0.40 mmol, 78%). **^{1}H NMR** (500 MHz, CD_3OD) δ 9.79 (ddd, *J* = 6.0, 1.8, 0.6 Hz, 1H), 8.61 (dd, *J* = 8.1, 0.8 Hz, 1H), 8.56 (dd, *J* = 7.8, 0.9 Hz, 1H), 8.40 (dd, *J* = 8.1, 0.8 Hz, 1H), 8.35 (dd, *J* = 8.5, 7.7 Hz, 1H), 8.31 (dd, *J* = 1.8, 1.1 Hz, 1H), 8.16 (dd, *J* = 7.7, 1.0 Hz, 1H), 8.08 (t, *J* = 7.9 Hz, 1H), 7.96 (t, *J* = 8.0 Hz, 1H), 7.82 (ddd, *J* = 8.7, 7.2, 1.8 Hz, 1H), 7.77 (dd, *J* = 8.5, 0.9 Hz, 1H), 7.74 – 7.70 (m, 2H), 7.69 – 7.64 (m, 2H), 7.62 (ddd, *J* = 8.4, 2.3, 1.2 Hz, 1H), 7.57 – 7.51 (m, 3H), 7.49 (dd, *J* = 8.1, 1.0 Hz, 1H), 7.37 – 7.24 (m, 4H), 7.05 (ddd, *J* = 7.2, 6.0, 1.3 Hz, 1H), 6.98 (ddd, *J* = 8.4, 5.7, 0.6 Hz, 1H), 6.94 – 6.84 (m, 1H), 6.66 (ddd, *J* = 7.2, 5.9, 1.3 Hz, 1H), 6.50 (ddd, *J* = 5.8, 1.8, 0.7 Hz, 1H), 4.11 (s, 2H), 1.32 (s, 9H). **^{13}C NMR** (126 MHz, CD_3OD) δ 167.99, 159.72, 159.31, 159.23, 157.67, 157.48, 157.38, 156.42, 152.10, 151.52, 148.49, 144.95, 143.86, 140.99, 140.67, 139.94, 139.05, 138.62, 135.43, 133.89, 129.13, 128.94, 128.77, 127.88, 127.16, 126.50, 123.81, 123.65, 121.18, 120.75, 120.62, 118.00, 117.45, 116.64, 116.04, 115.54, 47.30, 35.98, 31.47. **ESI-MS**: exact m/z calculated for $[C_{48}H_{44}Cl_2N_{10}O_3RuS–HCl–Cl]^+$: 941.2, found: 941.3; exact m/z calculated for $[C_{48}H_{44}Cl_2N_{10}O_3RuS–2Cl]^{2+}$: 471.1, found: 471.0. **HRMS**: exact m/z calculated for $[C_{48}H_{44}Cl_2N_{10}O_3RuS–HCl–Cl]^+$: 941.2290, found: 941.2275; exact m/z calculated for $[C_{48}H_{44}Cl_2N_{10}O_3RuS–2Cl]^{2+}$: 471.1182, found: 471.1175. **Elemental analysis** calculated for $C_{48}H_{44}Cl_2N_{10}O_3RuS$ (%): C, 56.91; H, 4.38; N, 13.83; found: C, 56.27; H, 4.33; N, 13.62.

*Synthesis of [Ru(baptpy)(**RAD-51-IN-1**)]Cl_2 ([**8**]Cl_2)*

[Ru(baptpy)Cl]Cl (0.15 g, 0.25 mmol, 1 eq), **RAD-51-IN-1** (0.11 g, 0.28 mmol, 1.1 eq), and $AgPF_6$ (0.14 g, 0.53 mmol, 2.1 eq) were mixed together in deoxygenated acetone/H_2O = 1/1 (2.5 mL) and stirred for 24 h at 50 °C under N_2 atmosphere in dark. Upon reaction completion the purple mixture was filtered, the filter cake was washed with acetone (10 mL) and the filtrate was concentrated *in vacuo*. The obtained residue was dissolved in minimal quantity of acetone and saturated tetrabutylammonium chloride (0.71 g, 2.54 mmol, 10 eq) acetone solution was added dropwise to form fine precipitate. The resulting solid was filtered, washed with acetone (2.5 mL) and Et_2O (25 mL) until all the side products are gone. Reprecipitation from MeOH/Et_2O followed by filtration and drying *in vacuo* afforded target compound as purple powder (0.17 g, 0.18 mmol, 69%). **^{1}H NMR** (400 MHz, CD_3OD) δ 9.90 – 9.84 (m, 1H), 8.60 (dd, *J* = 8.2, 0.8 Hz, 1H), 8.56 (dd, *J* = 7.7, 0.9 Hz, 1H), 8.38 – 8.32 (m, 2H),

8.21 (dd, *J* = 8.0, 1.4 Hz, 1H), 8.12 – 8.04 (m, 2H), 7.96 (t, *J* = 8.0 Hz, 1H), 7.89 – 7.79 (m, 3H), 7.75 (dd, *J* = 8.5, 0.9 Hz, 1H), 7.73 – 7.69 (m, 1H), 7.60 (d, *J* = 1.9 Hz, 1H), 7.58 – 7.49 (m, 3H), 7.43 – 7.35 (m, 2H), 7.31 – 7.23 (m, 3H), 7.18 – 7.11 (m, 2H), 7.09 – 6.99 (m, 3H), 6.90 (dt, *J* = 8.2, 1.1 Hz, 1H), 6.66 (ddd, *J* = 7.2, 5.9, 1.3 Hz, 1H), 6.48 – 6.42 (m, 1H), 5.47 (s, 2H). **^{13}C NMR** (101 MHz, CD_3OD) δ 163.55, 159.57, 159.53, 159.50, 159.05, 157.53, 157.35, 156.46, 153.74, 153.55, 153.03, 152.00, 151.60, 151.46, 148.49, 141.05, 140.71, 140.00, 139.14, 136.78, 136.11, 135.79, 135.63, 135.31, 134.81, 134.52, 130.07, 129.35, 128.57, 128.30, 127.96, 126.95, 125.21, 123.74, 123.69, 121.61, 121.21, 120.62, 118.09, 117.57, 116.66, 116.38, 115.59, 46.60. **ESI-MS**: exact m/z calculated for $[C_{47}H_{35}Cl_3N_{10}ORu–HCl–Cl]^+$: 891.2, found: 891.2; exact m/z calculated for $[C_{47}H_{35}Cl_3N_{10}ORu–2Cl]^{2+}$: 446.1, found: 445.8. **HRMS**: exact m/z calculated for $[C_{47}H_{35}Cl_3N_{10}ORu–Cl]^+$: 927.1412, found: 927.1403. **Elemental analysis** calculated for $C_{47}H_{35}Cl_3N_{10}ORu$ (%): C, 58.60; H, 3.66; N, 14.54; found: C, 58.19; H, 3.73; N, 14.43.

*Synthesis of [Ru(baptpy)(**QC-82**)]Cl₂ ([**9**]Cl₂)*

[Ru(baptpy)Cl]Cl (0.20 g, 0.34 mmol, 1 eq), **QC-82** (0.09 g, 0.37 mmol, 1.1 eq), and $AgPF_6$ (0.18 g, 0.71 mmol, 2.1 eq) were mixed together in deoxygenated acetone/H_2O = 1/1 (3.5 mL) and stirred for 24 h at 50 °C under N_2 atmosphere in dark. Upon reaction completion the violet mixture was filtered, the filter cake was washed with acetone (14 mL) and the filtrate was concentrated *in vacuo*. The obtained residue was dissolved in minimal quantity of acetone and saturated tetrabutylammonium chloride (0.94 g, 3.39 mmol, 10 eq) acetone solution was added dropwise to form fine precipitate. The resulting solid was filtered, washed with acetone (3.5 mL) and Et_2O (35 mL). Purification by SEC in MeOH afforded target compound as violet powder (0.10 g, 0.12 mmol, 35%). **^{1}H NMR** (400 MHz, CD_3OD) δ 9.69 (ddd, *J* = 5.9, 1.8, 0.7 Hz, 1H), 8.53 (dd, *J* = 8.1, 0.9 Hz, 1H), 8.48 (dd, *J* = 7.8, 0.9 Hz, 1H), 8.37 (dd, *J* = 8.1, 0.8 Hz, 1H), 8.29 (dd, *J* = 8.5, 7.7 Hz, 1H), 8.10 (dd, *J* = 7.7, 1.1 Hz, 1H), 8.03 (t, *J* = 7.8 Hz, 1H), 7.91 (t, *J* = 8.0 Hz, 1H), 7.81 (ddd, *J* = 8.7, 7.2, 1.8 Hz, 1H), 7.69 (dd, *J* = 8.5, 0.9 Hz, 1H), 7.51 (ddd, *J* = 8.3, 7.3, 1.8 Hz, 1H), 7.43 (dd, *J* = 7.9, 1.0 Hz, 1H), 7.24 (dt, *J* = 8.5, 1.1 Hz, 1H), 7.02 (ddd, *J* = 7.2, 5.9, 1.3 Hz, 1H), 6.84 (dt, *J* = 8.2, 1.1 Hz, 1H), 6.78 (t, *J* = 1.4 Hz, 1H), 6.63 (ddd, *J* = 7.2, 5.8, 1.3 Hz, 1H), 6.57 (t, *J* = 1.6 Hz, 1H), 6.52 (ddd, *J* = 5.8, 1.7, 0.6 Hz, 1H), 5.83 (t, *J* = 1.5 Hz, 1H), 4.83 (s, 2H), 1.97 (s, 3H), 1.80 – 1.64 (m, 12H). **^{13}C NMR** (126 MHz, CD_3OD) δ 208.12, 160.26, 159.98, 159.36, 159.29, 158.12, 157.17, 156.79, 151.91, 151.52, 151.44, 141.00, 140.67, 140.15, 139.67, 138.49, 134.61, 128.15, 123.54, 123.43, 123.16, 120.96, 120.09, 119.89, 117.40, 117.21, 116.32, 115.50, 115.32, 52.87, 46.55, 38.76, 37.34, 29.20. **ESI-MS**: exact m/z calculated for $[C_{40}H_{39}Cl_2N_9ORu–HCl–Cl]^+$: 762.2, found: 762.3; exact m/z calculated for $[C_{40}H_{39}Cl_2N_9ORu–2Cl]^{2+}$: 381.6, found: 381.5. **HRMS**: exact m/z calculated for $[C_{40}H_{39}Cl_2N_9ORu–HCl–Cl]^+$: 762.2248, found: 762.2229; exact m/z calculated for $[C_{40}H_{39}Cl_2N_9ORu–2Cl]^{2+}$: 381.6160, found: 381.6154. **Elemental analysis** calculated for $C_{40}H_{39}Cl_2N_9ORu$ (%): C, 57.62; H, 4.71; N, 15.12; found: C, 56.51; H, 4.71; N, 14.78.

*Synthesis of [Ru(baptpy)(**Norharmane**)]Cl₂ ([**10**]Cl₂)*

[Ru(baptpy)Cl]Cl (0.15 g, 0.25 mmol, 1 eq), **Norharmane** (0.05 g, 0.28 mmol, 1.1 eq), and $AgPF_6$ (0.14 g, 0.53 mmol, 2.1 eq) were mixed together in deoxygenated acetone/H_2O = 1/1 (2.5 mL) and stirred for 24 h at 50 °C under N_2 atmosphere in dark. Upon reaction completion the violet mixture was filtered, the filter cake was washed with acetone (10 mL) and the filtrate was concentrated *in vacuo*. The obtained residue was dissolved in minimal quantity of acetone and saturated tetrabutylammonium chloride (0.71 g, 2.54 mmol, 10 eq) acetone solution was added dropwise to form fine precipitate. The resulting solid was filtered,

washed with acetone (2.5 mL) and Et_2O (25 mL). Purification by SEC in MeOH afforded target compound as violet powder (0.08 g, 0.11 mmol, 42%). **^{1}H NMR** (400 MHz, CD_3OD) δ 9.86 (dd, J = 6.1, 1.7 Hz, 1H), 8.60 (ddd, J = 8.1, 2.2, 0.9 Hz, 2H), 8.40 (dd, J = 8.5, 7.7 Hz, 1H), 8.34 (dd, J = 8.1, 0.8 Hz, 1H), 8.19 – 8.09 (m, 2H), 7.97 (dt, J = 8.0, 1.0 Hz, 1H), 7.89 (t, J = 8.0 Hz, 1H), 7.83 – 7.73 (m, 2H), 7.63 (dd, J = 6.2, 0.9 Hz, 1H), 7.60 – 7.46 (m, 4H), 7.42 (dt, J = 8.4, 0.9 Hz, 1H), 7.29 – 7.23 (m, 1H), 7.23 – 7.15 (m, 2H), 7.01 (ddd, J = 7.2, 5.9, 1.3 Hz, 1H), 6.94 – 6.86 (m, 1H), 6.68 (ddd, J = 7.3, 5.8, 1.3 Hz, 1H), 6.52 (dd, J = 6.1, 1.7 Hz, 1H). **^{13}C NMR** (101 MHz, CD_3OD) δ 159.93, 159.77, 159.37, 159.23, 157.87, 157.39, 156.44, 152.13, 151.55, 151.43, 142.99, 141.40, 140.96, 140.60, 139.92, 138.96, 137.48, 136.75, 135.19, 131.01, 130.13, 123.66, 123.43, 122.98, 121.69, 121.19, 121.04, 120.51, 120.44, 117.88, 117.39, 117.10, 116.54, 115.95, 115.52, 113.00. **ESI-MS**: exact m/z calculated for $[C_{36}H_{27}Cl_2N_9Ru–HCl–Cl]^+$: 686.1, found: 686.2; exact m/z calculated for $[C_{36}H_{27}Cl_2N_9Ru–2Cl]^{2+}$: 343.6, found: 343.5. **HRMS**: exact m/z calculated for $[C_{36}H_{27}Cl_2N_9Ru–2Cl]^{2+}$: 343.5716, found: 343.5709. **Elemental analysis** calculated for $C_{36}H_{27}Cl_2N_9Ru$ (%): C, 57.07; H, 3.59; N, 16.64; found: C, 56.12; H, 3.73; N, 16.46.

*Synthesis of [Ru(baptpy)(**Neratinib**)]Cl_2 ([**11**]Cl_2)*

[Ru(baptpy)Cl]Cl (0.20 g, 0.34 mmol, 1 eq), **Neratinib** (0.21 g, 0.37 mmol, 1.1 eq), and $AgPF_6$ (0.18 g, 0.71 mmol, 2.1 eq) were mixed together in deoxygenated acetone/H_2O = 1/1 (3.5 mL) and stirred for 24 h at 50 °C under N_2 atmosphere in dark. Upon reaction completion the brownish red mixture was filtered, the filter cake was washed with acetone (14 mL) and the filtrate was concentrated *in vacuo*. The obtained residue was dissolved in minimal quantity of acetone and saturated tetrabutylammonium chloride (0.94 g, 3.39 mmol, 10 eq) acetone solution was added dropwise to form fine precipitate. The resulting solid was filtered, washed with acetone (3.5 mL) and Et_2O (35 mL). Purification by SEC in MeOH afforded target compound as brownish red powder (0.17 g, 0.15 mmol, 44%). **^{1}H NMR** (400 MHz, CD_3OD) δ 9.34 (dd, J = 5.9, 1.8 Hz, 1H), 8.76 (s, 1H), 8.71 (ddd, J = 4.9, 1.7, 0.9 Hz, 1H), 8.61 (d, J = 8.1 Hz, 1H), 8.53 (d, J = 8.0 Hz, 1H), 8.47 (dd, J = 7.7, 0.9 Hz, 1H), 8.26 (dd, J = 8.5, 7.7 Hz, 1H), 8.20 (dd, J = 7.7, 1.0 Hz, 1H), 8.15 – 8.07 (m, 2H), 8.01 (td, J = 7.7, 1.8 Hz, 1H), 7.90 (ddd, J = 8.7, 7.2, 1.8 Hz, 1H), 7.80 – 7.75 (m, 1H), 7.69 – 7.62 (m, 2H), 7.56 – 7.48 (m, 2H), 7.46 (dd, J = 8.1, 0.9 Hz, 1H), 7.32 – 7.26 (m, 2H), 7.15 (ddd, J = 7.2, 5.9, 1.3 Hz, 1H), 6.94 – 6.84 (m, 3H), 6.82 – 6.74 (m, 1H), 6.73 – 6.62 (m, 3H), 6.47 (dd, J = 5.9, 1.7 Hz, 1H), 5.28 – 5.17 (m, 2H), 4.32 (q, J = 7.0 Hz, 2H), 4.07 – 3.92 (m, 2H), 2.91 (s, 6H), 1.52 (t, J = 7.0 Hz, 3H). **^{13}C NMR** (101 MHz, CD_3OD) δ 164.49, 159.96, 159.69, 159.66, 159.19, 157.67, 157.24, 156.97, 156.92, 155.48, 153.89, 152.56, 152.35, 151.43, 151.41, 151.37, 150.19, 148.76, 141.09, 140.11, 139.45, 139.36, 136.68, 134.94, 133.70, 133.28, 129.46, 125.38, 125.03, 124.59, 124.00, 123.70, 123.55, 122.97, 122.78, 121.14, 120.40, 120.11, 117.80, 117.57, 116.65, 116.34, 115.84, 115.71, 115.01, 114.38, 109.13, 87.05, 72.86, 66.49, 58.78, 43.30, 14.59. **ESI-MS**: exact m/z calculated for $[C_{55}H_{48}Cl_3N_{13}O_3Ru–HCl–Cl]^+$: 1074.3, found: 1074.2; exact m/z calculated for $[C_{55}H_{48}Cl_3N_{13}O_3Ru–2Cl]^{2+}$: 537.6, found: 537.9. **HRMS**: exact m/z calculated for $[C_{55}H_{48}Cl_3N_{13}O_3Ru–HCl–Cl]^+$: 1074.2663, found: 1074.2659; exact m/z calculated for $[C_{55}H_{48}Cl_3N_{13}O_3Ru–Cl]^+$: 1110.2427, found: 1110.2424. **Elemental analysis** calculated for $C_{55}H_{48}Cl_3N_{13}O_3Ru$ (%): C, 57.62; H, 4.22; N, 15.88; found: C, 56.61; H, 4.37; N, 15.46.

*Synthesis of [Ru(baptpy)(**Bosutinib**)]Cl_2 ([**12**]Cl_2)*

[Ru(baptpy)Cl]Cl (0.20 g, 0.34 mmol, 1 eq), **Bosutinib** (0.20 g, 0.37 mmol, 1.1 eq), and $AgPF_6$ (0.18 g, 0.71 mmol, 2.1 eq) were mixed together in deoxygenated acetone/H_2O = 1/1 (3.5 mL) and stirred for 24 h at 50 °C under N_2 atmosphere in dark. Upon reaction completion

the brownish red mixture was filtered, the filter cake was washed with acetone (14 mL) and the filtrate was concentrated *in vacuo*. The obtained residue was dissolved in minimal quantity of acetone and saturated tetrabutylammonium chloride (0.94 g, 3.39 mmol, 10 eq) acetone solution was added dropwise to form fine precipitate. The resulting solid was filtered, washed with acetone (3.5 mL) and Et_2O (35 mL). Purification by SEC in MeOH afforded target compound as brownish red powder (0.16 g, 0.14 mmol, 42%). **^{1}H NMR** (400 MHz, CD_3OD) δ 9.20 (s, 1H), 8.66 (d, *J* = 8.2 Hz, 1H), 8.55 (d, *J* = 7.7 Hz, 2H), 8.46 – 8.17 (m, 3H), 8.15 – 8.08 (m, 1H), 7.93 (ddd, *J* = 8.8, 7.2, 1.8 Hz, 1H), 7.89 – 7.40 (m, 5H), 7.31 (dt, *J* = 8.4, 1.0 Hz, 1H), 7.25 – 7.15 (m, 2H), 7.05 – 6.39 (m, 5H), 4.22 (s, 2H), 3.92 (s, 3H), 3.72 – 3.34 (m, 5H), 2.79 (s, 11H), 2.13 (s, 2H). **^{13}C NMR** (101 MHz, CD_3OD) δ 157.50, 152.65, 151.89, 151.48, 141.16, 140.11, 139.54, 136.76, 130.82, 123.36, 121.11, 119.95, 117.96, 117.49, 115.31, 109.51, 102.51, 67.95, 57.14, 56.94, 55.15, 54.51, 51.54, 43.87, 26.80. **ESI-MS**: exact m/z calculated for $[C_{51}H_{48}Cl_4N_{12}O_3Ru–HCl–Cl]^+$: 1049.2, found: 1049.3; exact m/z calculated for $[C_{51}H_{48}Cl_4N_{12}O_3Ru–2Cl]^{2+}$: 524.1, found: 524.6; exact m/z calculated for $[C_{51}H_{48}Cl_4N_{12}O_3Ru–2Cl+H]^{3+}$: 350.4, found: 350.0. **HRMS**: exact m/z calculated for $[C_{51}H_{48}Cl_4N_{12}O_3Ru–HCl–Cl]^+$: 1047.2317, found: 1047.2308; exact m/z calculated for $[C_{51}H_{48}Cl_4N_{12}O_3Ru–2Cl+H]^{3+}$: 349.7487, found: 349.7485. **Elemental analysis** calculated for $C_{51}H_{48}Cl_4N_{12}O_3Ru$ (%): C, 54.70; H, 4.32; N, 15.01; found: C, 53.75; H, 4.43; N, 14.67.

*Synthesis of [Ru(baptpy)(**Ponatinib**)]Cl_2 ([**13**]Cl_2)*

[Ru(baptpy)Cl]Cl (0.10 g, 0.17 mmol, 1 eq), **Ponatinib** (0.10 g, 0.19 mmol, 1.1 eq), and $AgPF_6$ (0.09 g, 0.36 mmol, 2.1 eq) were mixed together in deoxygenated acetone/H_2O = 1/1 (1.7 mL) and stirred for 24 h at 50 °C under N_2 atmosphere in dark. Upon reaction completion the violet mixture was filtered, the filter cake was washed with acetone (7 mL) and the filtrate was concentrated *in vacuo*. The obtained residue was dissolved in minimal quantity of acetone and saturated tetrabutylammonium chloride (0.47 g, 1.70 mmol, 10 eq) acetone solution was added dropwise to form fine precipitate. The resulting solid was filtered, washed with acetone (1.7 mL) and Et_2O (17 mL) until all the side products are gone. Reprecipitation from MeOH/Et_2O followed by filtration and drying *in vacuo* afforded target compound as violet powder (0.14 g, 0.12 mmol, 74%). **^{1}H NMR** (400 MHz, CD_3OD) δ 9.95 (dd, *J* = 6.0, 1.7 Hz, 1H), 8.64 (ddd, *J* = 8.1, 2.1, 0.9 Hz, 2H), 8.54 (dd, *J* = 4.5, 1.5 Hz, 1H), 8.42 (dd, *J* = 8.5, 7.6 Hz, 1H), 8.26 (dd, *J* = 8.1, 0.8 Hz, 1H), 8.15 – 8.09 (m, 2H), 8.05 (dd, *J* = 7.7, 1.2 Hz, 1H), 7.99 – 7.89 (m, 3H), 7.88 – 7.82 (m, 2H), 7.81 – 7.74 (m, 2H), 7.60 (dd, *J* = 8.0, 1.1 Hz, 1H), 7.55 (ddd, *J* = 8.4, 7.3, 1.7 Hz, 1H), 7.40 (d, *J* = 8.1 Hz, 1H), 7.32 – 7.22 (m, 2H), 7.07 (dd, *J* = 9.5, 1.5 Hz, 1H), 7.01 (ddd, *J* = 7.2, 6.0, 1.3 Hz, 1H), 6.96 (dt, *J* = 8.2, 1.1 Hz, 1H), 6.68 (ddd, *J* = 7.3, 5.9, 1.3 Hz, 1H), 6.52 – 6.46 (m, 1H), 6.44 (s, 1H), 3.76 (s, 2H), 3.47 – 3.33 (m, 1H), 3.29 – 2.26 (m, 13H). **^{13}C NMR** (101 MHz, CD_3OD) δ 167.48, 160.11, 159.95, 159.84, 159.21, 157.87, 157.09, 151.91, 151.78, 151.45, 147.30, 145.78, 142.38, 140.91, 140.64, 139.88, 139.61, 139.32, 136.06, 135.41, 133.65, 132.95, 132.74, 132.18, 131.12, 130.33, 130.03, 129.52, 127.05, 125.12, 124.34, 124.14, 123.77, 123.55, 122.99, 122.77, 121.37, 120.61, 120.40, 119.29, 119.23, 118.19, 117.49, 116.76, 116.21, 115.62, 115.60, 98.68, 78.88, 58.21, 55.06, 51.18, 43.64, 20.76. **^{19}F NMR** (376 MHz, CD_3OD) δ -60.39 (s, 3F). **ESI-MS**: exact m/z calculated for $[C_{54}H_{46}Cl_2F_3N_{13}ORu–HCl–Cl]^+$: 1050.3, found: 1050.3; exact m/z calculated for $[C_{54}H_{46}Cl_2F_3N_{13}ORu–2Cl]^{2+}$: 525.6, found: 525.8; exact m/z calculated for $[C_{54}H_{46}Cl_2F_3N_{13}ORu–2Cl+H]^{3+}$: 350.8, found: 350.8. **HRMS**: exact m/z calculated for $[C_{54}H_{46}Cl_2F_3N_{13}ORu–HCl–Cl]^+$: 1050.2874, found: 1050.2862; exact m/z calculated for $[C_{54}H_{46}Cl_2F_3N_{13}ORu–2Cl+H]^{3+}$: 350.7673, found: 350.7671. **Elemental analysis** calculated for $C_{54}H_{46}Cl_2F_3N_{13}ORu$ (%): C, 57.81; H, 4.13; N, 16.23; found: C, 56.72; H, 4.25; N, 15.84.

*Synthesis of [Ru(baptpy)(**Albendazole**)](PF_6)$_2$ ([**14**](PF_6)$_2$)*

[Ru(baptpy)Cl]Cl (0.10 g, 0.17 mmol, 1 eq), **Albendazole** (0.09 g, 0.34 mmol, 2 eq), and $AgPF_6$ (0.09 g, 0.36 mmol, 2.1 eq) were mixed together in deoxygenated acetone/H_2O = 1/1 (1.7 mL) and stirred for 24 h at 50 °C under N_2 atmosphere in dark. Upon reaction completion the reddish mixture was filtered, the filter cake was washed with acetone (7 mL) and the filtrate was concentrated *in vacuo*. Purification by column chromatography on silica gel (DCM/MeOH = 10/1) afforded target compound as dark red powder (0.14 g, 0.13 mmol, 77%). **TLC** (DCM/MeOH = 10/1, product R_f = 0.40, dark red spot). **^{1}H NMR** (500 MHz, Acetone-d_6) δ 11.62 (s, 2H), 10.36 (s, 1H), 10.15 – 10.02 (m, 1H), 9.91 (s, 1H), 8.50 (dd, J = 8.1, 4.5 Hz, 2H), 8.30 (d, J = 7.6 Hz, 1H), 8.18 (t, J = 8.0 Hz, 1H), 8.15 – 8.05 (m, 3H), 7.99 (t, J = 7.8 Hz, 1H), 7.64 – 7.59 (m, 1H), 7.56 (t, J = 8.0 Hz, 2H), 7.28 (dd, J = 7.5, 4.9 Hz, 2H), 7.10 (d, J = 8.3 Hz, 1H), 7.05 (d, J = 8.3 Hz, 1H), 6.81 – 6.68 (m, 2H), 6.49 (s, 1H), 6.09 (dd, J = 8.2, 1.7 Hz, 1H), 3.92 (s, 3H), 2.49 – 2.25 (m, 2H), 1.19 – 1.09 (m, 2H), 0.62 (t, J = 7.3 Hz, 3H). **^{13}C NMR** (126 MHz, Acetone-d_6) δ 159.21, 158.17, 157.48, 156.73, 155.63, 151.23, 150.71, 149.92, 141.04, 140.38, 140.15, 138.63, 135.55, 123.63, 123.38, 121.37, 119.76, 117.59, 117.42, 116.60, 116.47, 115.84, 53.64, 35.60, 20.89, 12.93. **^{31}P NMR** (162 MHz, Acetone-d_6) δ -144.64 (hept, J = 706.6 Hz, 2P). **^{19}F NMR** (376 MHz, Acetone-d_6) δ -72.85 (d, J = 707.3 Hz, 12F). **ESI-MS**: exact m/z calculated for $[C_{37}H_{34}F_{12}N_{10}O_2P_2RuS–HPF_6–PF_6]^+$: 783.2, found: 783.2; exact m/z calculated for $[C_{37}H_{34}F_{12}N_{10}O_2P_2RuS–2PF_6]^{2+}$: 392.1, found: 392.1. **HRMS**: exact m/z calculated for $[C_{37}H_{34}F_{12}N_{10}O_2P_2RuS–2PF_6]^{2+}$: 392.0814, found: 392.0804. **Elemental analysis** calculated for $C_{37}H_{34}F_{12}N_{10}O_2P_2RuS$ (%): C, 41.39; H, 3.19; N, 13.04; found: C, 39.14; H, 3.01; N, 12.71.

*Synthesis of [Ru(baptpy)(**MTI**)](PF_6)$_2$ ([**15**](PF_6)$_2$)*

[Ru(baptpy)(H_2O)](PF_6)$_2$ (0.10 g, 0.12 mmol, 1 eq) and **MTI** (0.05 g, 0.13 mmol, 1.1 eq) were mixed together in deoxygenated DMF (1.2 mL) and stirred for 24 h at 50 °C under N_2 atmosphere in dark. Upon reaction completion the cherry mixture was quenched with Et_2O (10 mL) and filtered, the filter cake was washed with Et_2O (20 mL) and dried *in vacuo*. Purification by column chromatography on silica gel (Acetone with 3% of sat. aq. KPF_6) followed by washing with water afforded target compound as dark red powder (0.07 g, 0.06 mmol, 48%). **TLC** ($CHCl_3$/MeOH = 8/1, product R_f = 0.30, cherry spot). **^{1}H NMR** (400 MHz, Acetone-d_6) δ 11.44 (s, 1H), 10.71 (s, 1H), 10.41 (s, 1H), 9.85 (dd, J = 5.9, 1.7 Hz, 1H), 9.80 (s, 1H), 8.71 (dd, J = 8.2, 0.8 Hz, 1H), 8.57 – 8.54 (m, 1H), 8.53 (dd, J = 7.8, 0.9 Hz, 1H), 8.28 – 8.23 (m, 1H), 8.19 – 8.12 (m, 2H), 8.06 (t, J = 7.9 Hz, 1H), 7.89 (ddd, J = 8.7, 7.2, 1.7 Hz, 1H), 7.61 (dd, J = 8.1, 5.8 Hz, 2H), 7.55 – 7.49 (m, 3H), 7.33 – 7.27 (m, 3H), 7.26 – 7.22 (m, 1H), 7.17 – 7.10 (m, 3H), 7.00 (qd, J = 4.6, 4.0, 2.3 Hz, 4H), 6.78 (ddd, J = 7.3, 5.9, 1.3 Hz, 1H), 6.68 (dd, J = 5.8, 1.7 Hz, 1H), 2.51 (t, J = 6.6 Hz, 2H), 1.85 – 1.65 (m, 4H), 1.32 (s, 3H). **^{13}C NMR** (101 MHz, Acetone-d_6) δ 188.64, 159.69, 159.28, 159.25, 159.01, 158.67, 158.41, 157.22, 156.80, 155.54, 151.00, 150.83, 150.52, 141.11, 140.53, 140.22, 139.10, 138.55, 135.90, 133.52, 132.63, 132.39, 130.16, 128.52, 128.46, 128.37, 127.83, 123.85, 123.65, 121.45, 120.14, 120.00, 117.70, 117.60, 116.96, 116.61, 115.89, 105.96, 34.17, 32.78, 23.48, 15.54. **^{31}P NMR** (162 MHz, Acetone-d_6) δ -144.30 (hept, 2P, J = 707.5 Hz). **^{19}F NMR** (376 MHz, Acetone-d_6) δ -73.52 (d, J = 707.4 Hz, 12F). **ESI-MS**: exact m/z calculated for $[C_{48}H_{40}F_{12}N_{10}O_2P_2RuS–HPF_6–PF_6]^+$: 921.2, found: 921.2; exact m/z calculated for $[C_{48}H_{40}F_{12}N_{10}O_2P_2RuS–2PF_6]^{2+}$: 461.1, found: 460.8. **HRMS**: exact m/z calculated for $[C_{48}H_{40}F_{12}N_{10}O_2P_2RuS–HPF_6–PF_6]^+$: 921.2028, found: 921.2014; exact m/z calculated for $[C_{48}H_{40}F_{12}N_{10}O_2P_2RuS–2PF_6]^{2+}$: 461.1050, found: 461.1047. **Elemental analysis** calculated for $C_{48}H_{40}F_{12}N_{10}O_2P_2RuS$ (%): C, 47.57; H, 3.33; N, 11.56; found: C, 46.27; H, 3.41; N, 11.19.

*Synthesis of [Ru(baptpy)(**Gemcitabine**)]Cl_2 ([**16**]Cl_2)*

[Ru(baptpy)(H_2O)](PF_6)$_2$ (0.15 g, 0.18 mmol, 1 eq) and **Gemcitabine** (0.24 g, 0.91 mmol, 5 eq) were mixed together in deoxygenated acetone (1.8 mL) and stirred for 24 h at 50 °C under N_2 atmosphere in dark. Upon reaction completion the violet mixture was filtered, the filter cake was washed with acetone (10 mL) and the filtrate was concentrated *in vacuo*. The obtained residue was dissolved in minimal quantity of acetone and saturated tetrabutylammonium chloride (0.50 g, 1.80 mmol, 10 eq) acetone solution was added dropwise to form fine precipitate. The resulting solid was filtered, washed with acetone (1.8 mL) and Et_2O (18 mL) until all the side products are gone. Reprecipitation from MeOH/Et_2O followed by filtration and drying *in vacuo* afforded target compound as violet powder (0.10 g, 0.12 mmol, 65%). The compound was characterized as equimolar mixture of diastereomers. **^{1}H NMR** (400 MHz, DMSO-d_6) δ 11.95 (d, J = 6.1 Hz, 1H), 11.02 (d, J = 8.9 Hz, 1H), 10.86 (s, 1H), 9.72 – 9.62 (m, 1H), 8.73 (d, J = 8.1 Hz, 1H), 8.66 – 8.54 (m, 2H), 8.32 (t, J = 8.0 Hz, 1H), 8.22 (dd, J = 7.7, 3.0 Hz, 1H), 8.04 (t, J = 7.9 Hz, 2H), 7.94 (dd, J = 8.5, 2.8 Hz, 1H), 7.87 (t, J = 8.0 Hz, 1H), 7.59 – 7.47 (m, 3H), 7.25 (dd, J = 8.4, 4.6 Hz, 1H), 7.08 – 6.97 (m, 2H), 6.62 (t, J = 6.6 Hz, 1H), 6.36 (d, J = 5.8 Hz, 1H), 6.27 (d, J = 6.5 Hz, 1H), 5.93 (s, 1H), 5.75 (p, J = 7.8, 7.3 Hz, 1H), 5.18 (q, J = 6.2 Hz, 1H), 4.88 – 4.78 (m, 1H), 4.07 – 3.94 (m, 1H), 3.75 – 3.62 (m, 2H), 3.58 – 3.50 (m, 1H). **^{13}C NMR** (126 MHz, DMSO-d_6) δ 165.64, 158.16, 157.96, 157.77, 156.20, 155.58, 155.03, 154.59, 150.41, 149.78, 149.66, 138.64, 122.82, 121.88, 119.60, 118.79, 116.43, 115.38, 114.10, 96.63, 93.91, 79.65, 69.24, 68.51, 67.50, 60.02, 13.17. **^{19}F NMR** (471 MHz, DMSO-d_6) δ -115.22 – -117.79 (m, 2F). **ESI-MS**: exact m/z calculated for [$C_{34}H_{30}Cl_2F_2N_{10}O_4$Ru–HCl–Cl]$^+$: 781.1, found: 781.2; exact m/z calculated for [$C_{34}H_{30}Cl_2F_2N_{10}O_4$Ru–2Cl]$^{2+}$: 391.0, found: 390.8. **HRMS**: exact m/z calculated for [$C_{34}H_{30}Cl_2F_2N_{10}O_4$Ru–HCl–Cl]$^+$: 781.1388, found: 781.1387. **Elemental analysis** calculated for $C_{34}H_{30}Cl_2F_2N_{10}O_4$Ru (%): C, 47.89; H, 3.55; N, 16.43; found: C, 47.79; H, 3.54; N, 16.32.

1.4 NMR data

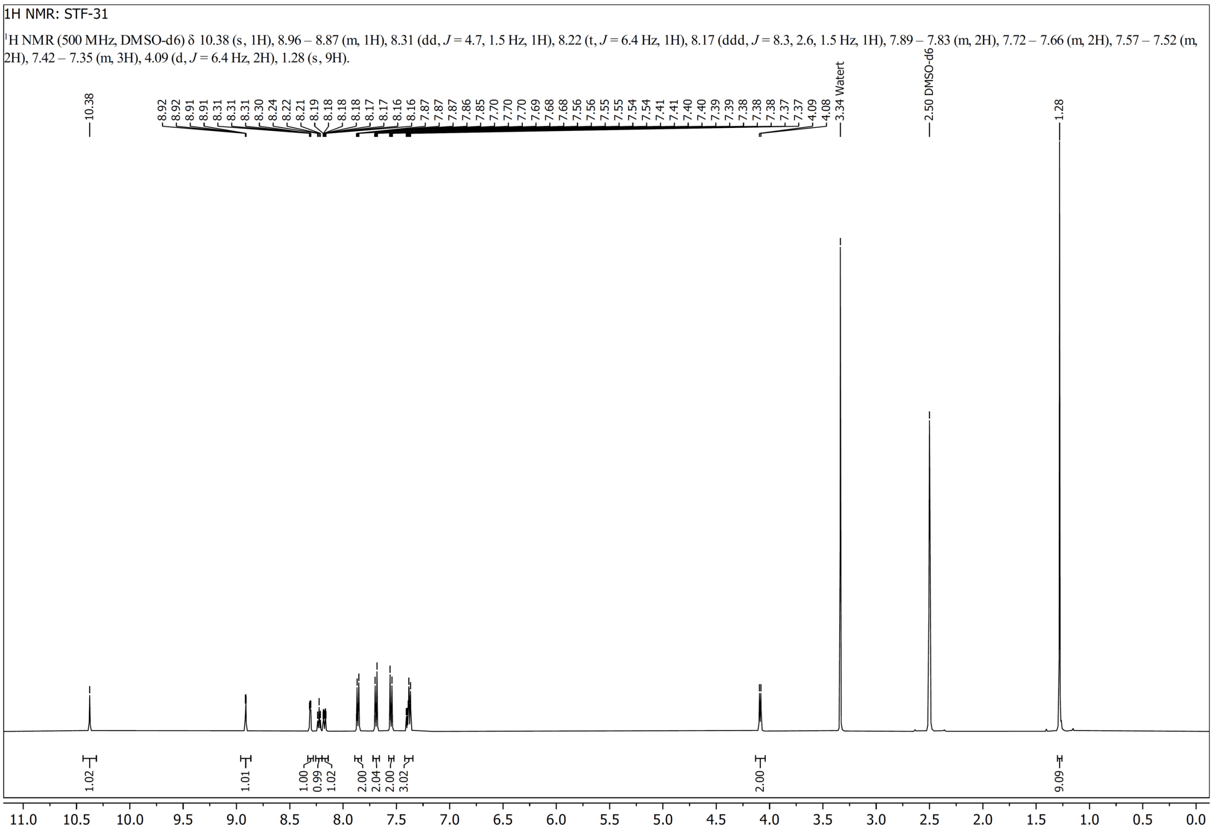

Figure S1. ^{1}H NMR of **STF-31** in DMSO-d_6.

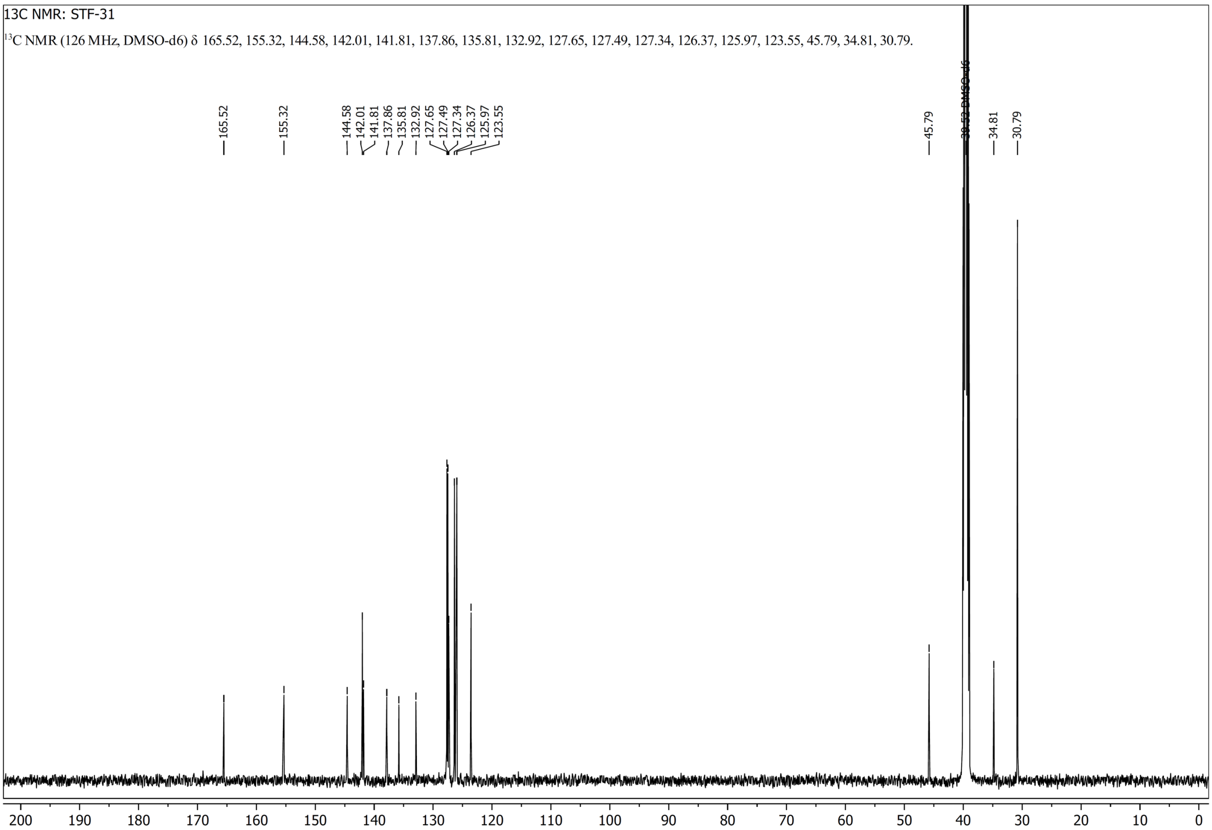

Figure S2. ^{13}C NMR of **STF-31** in DMSO-d_6.

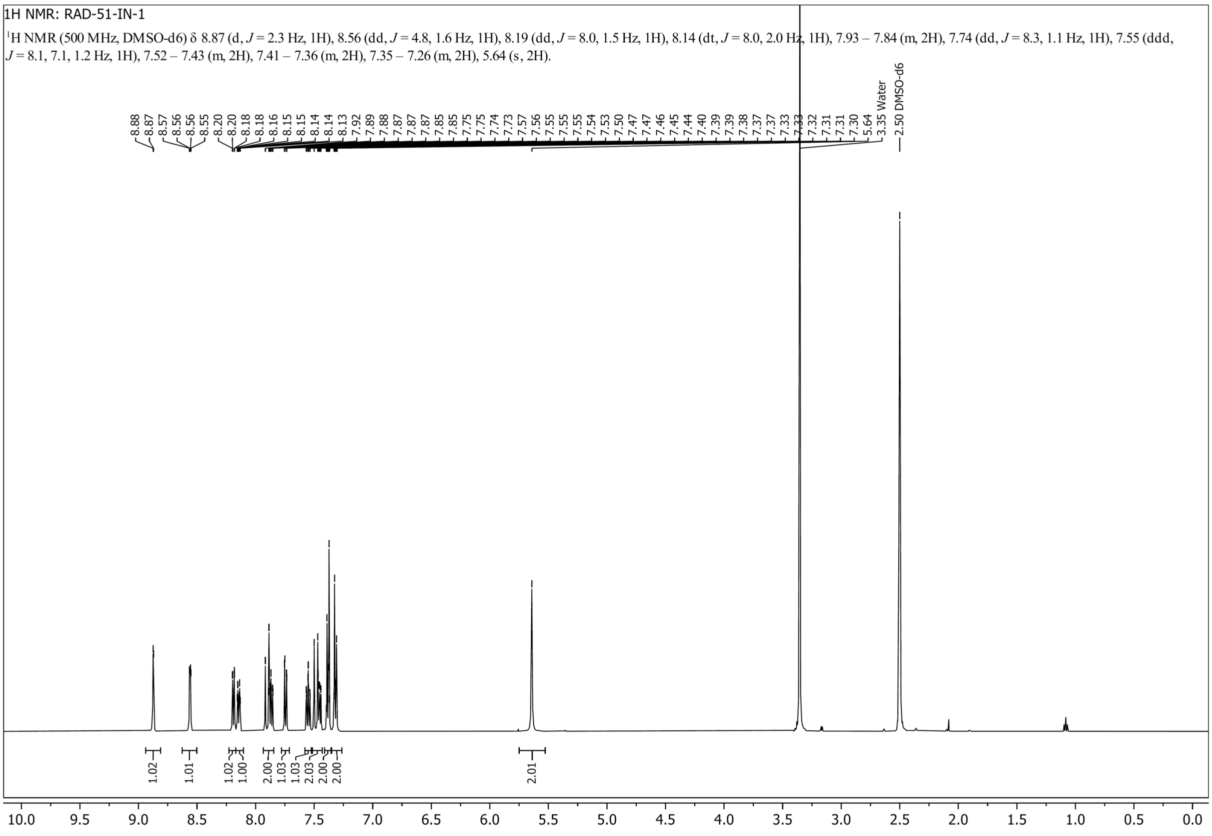

Figure S3. ^{1}H NMR of **RAD-51-IN-1** in DMSO-*d*$_6$.

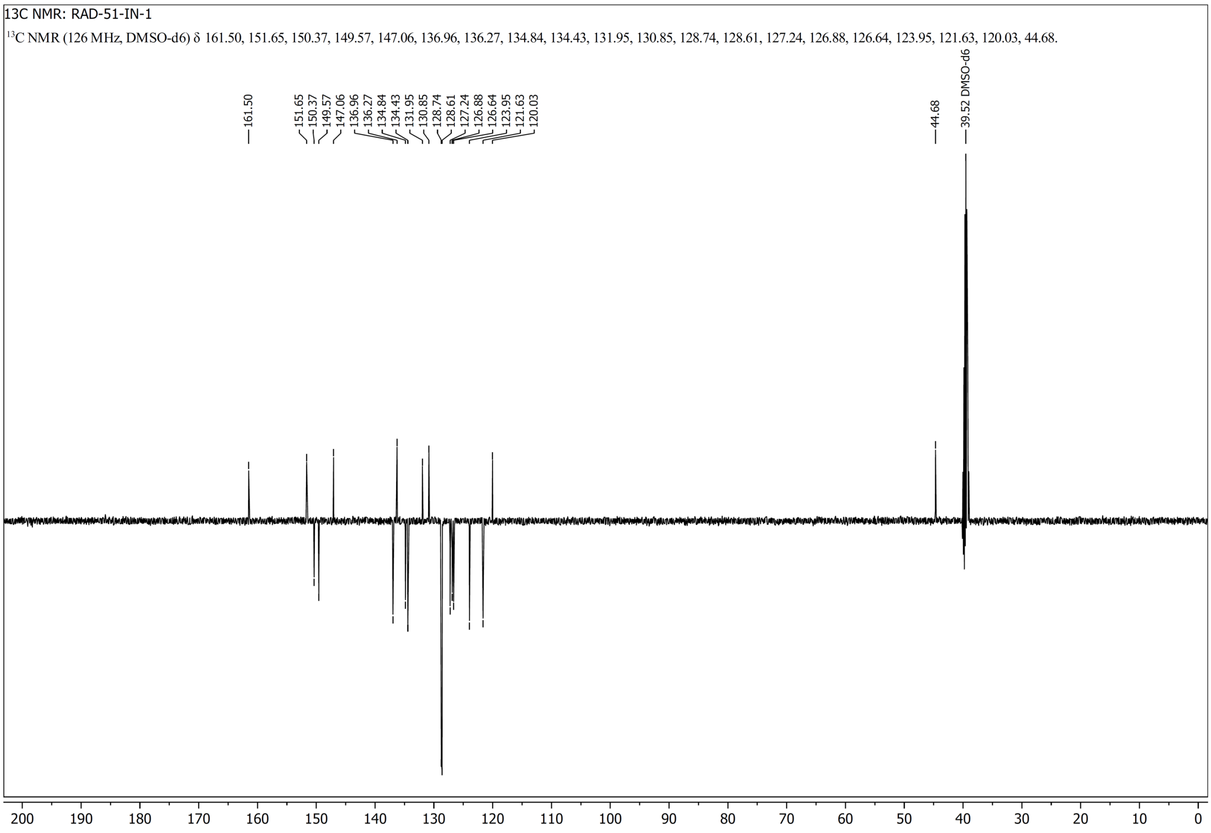

Figure S4. ^{13}C NMR of **RAD-51-IN-1** in DMSO-*d*$_6$.

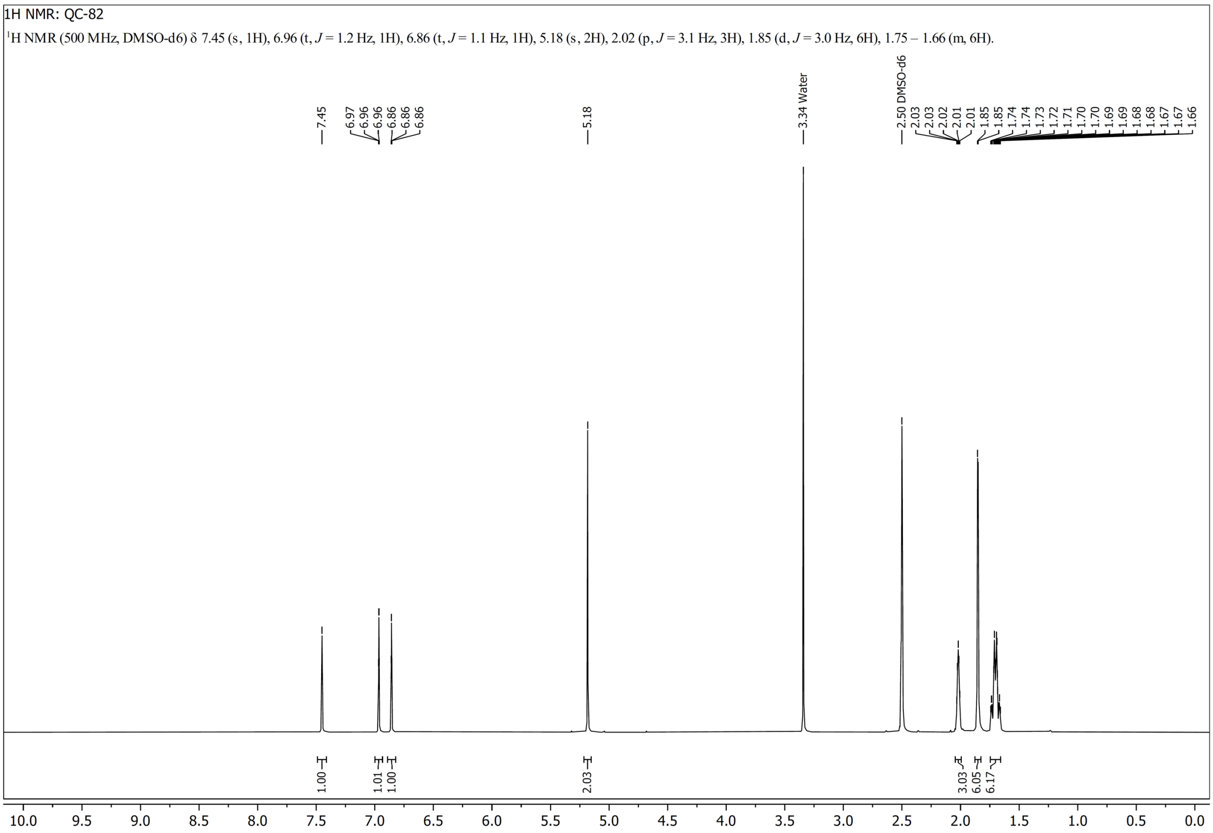

Figure S5. ^{1}H NMR of **QC-82** in DMSO-d_6.

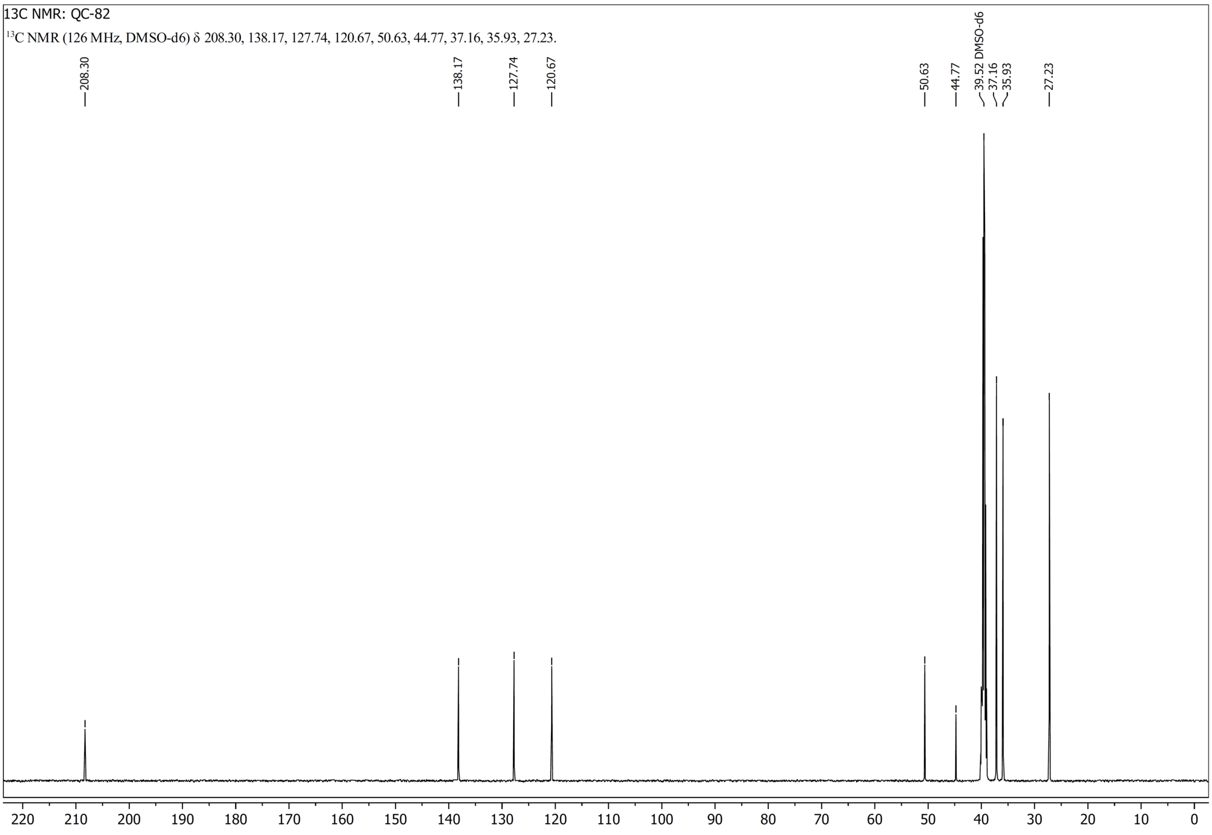

Figure S6. ^{13}C NMR of **QC-82** in DMSO-d_6.

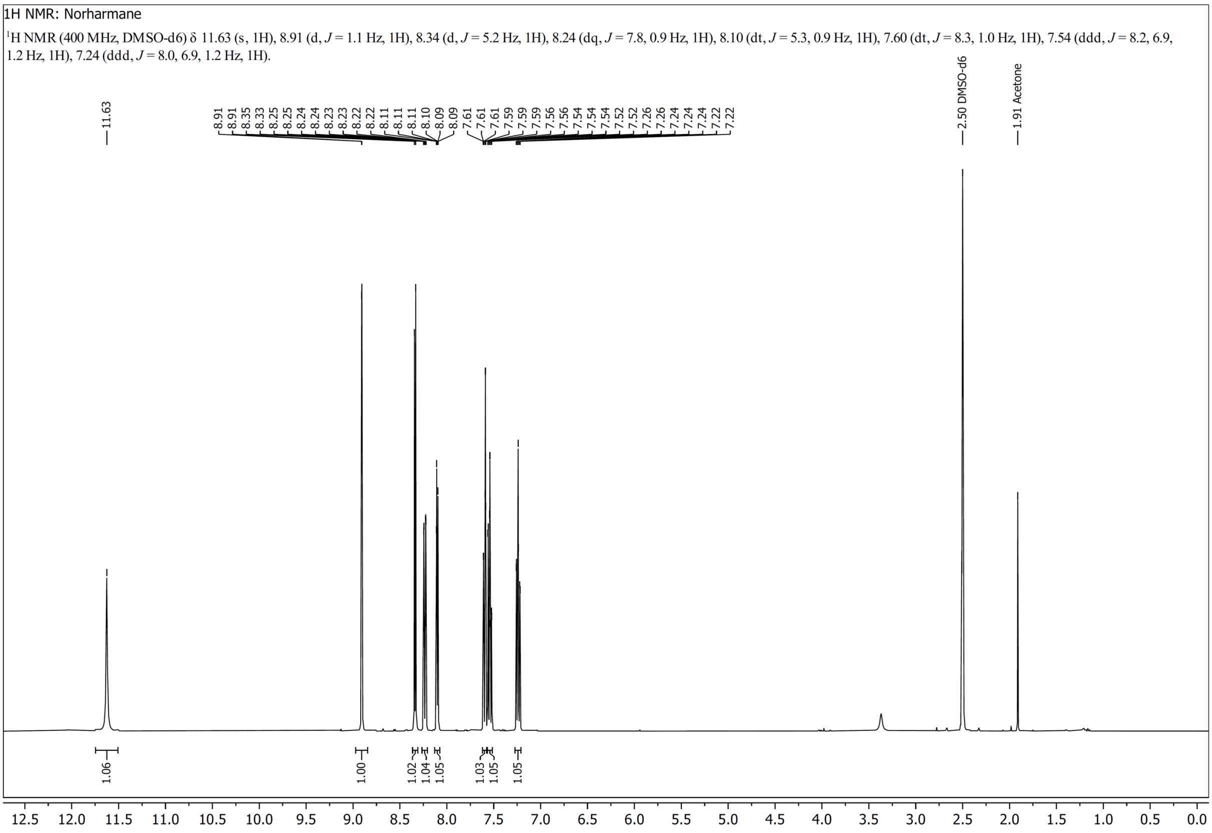

Figure S7. ^{1}H NMR of **Norharmane** in DMSO-d_6.

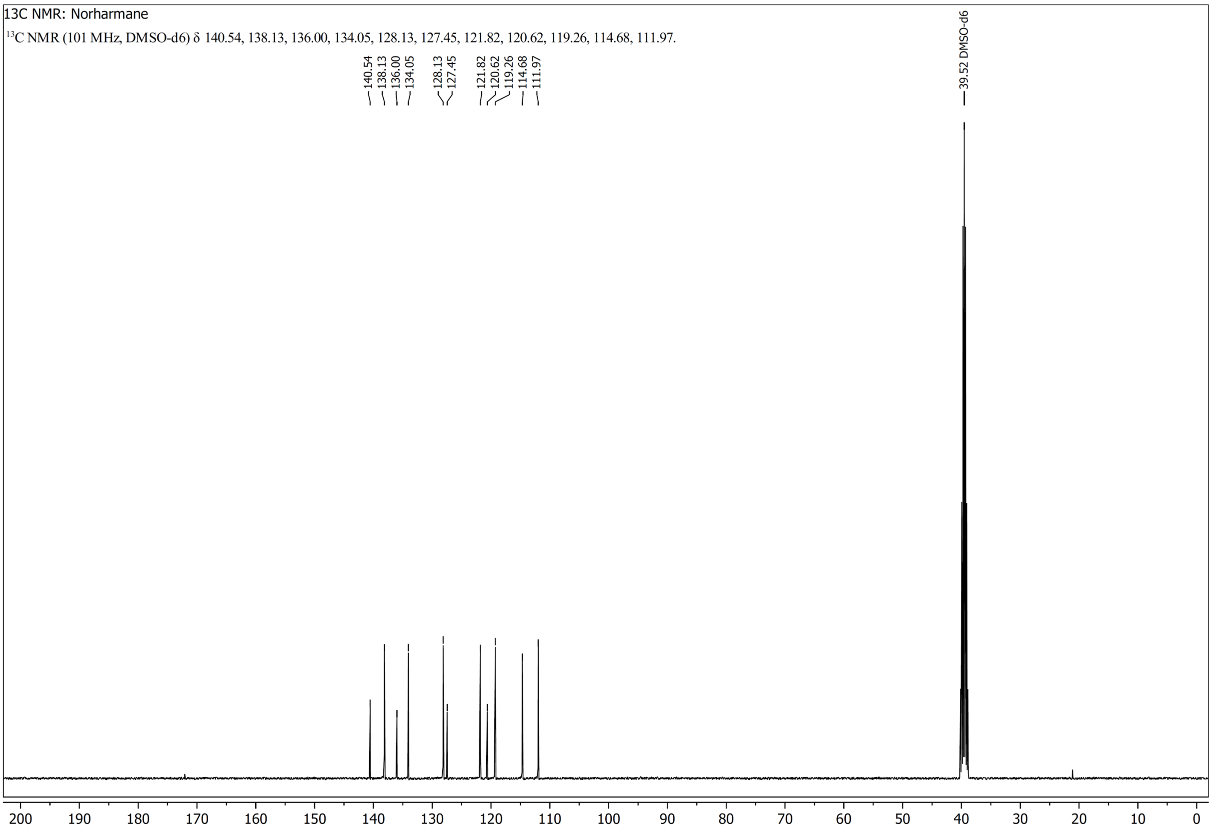

Figure S8. ^{13}C NMR of **Norharmane** in DMSO-d_6.

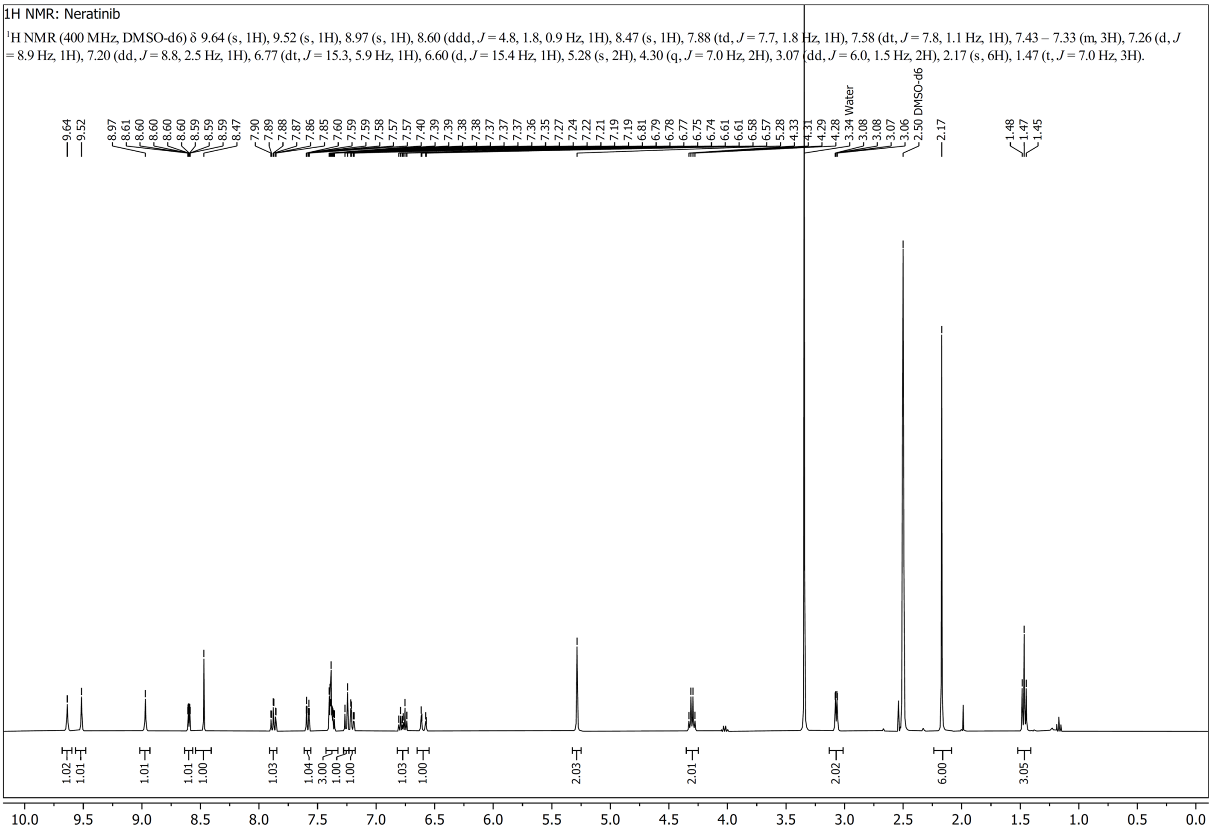

Figure S9. ^{1}H NMR of **Neratinib** in DMSO-d_6.

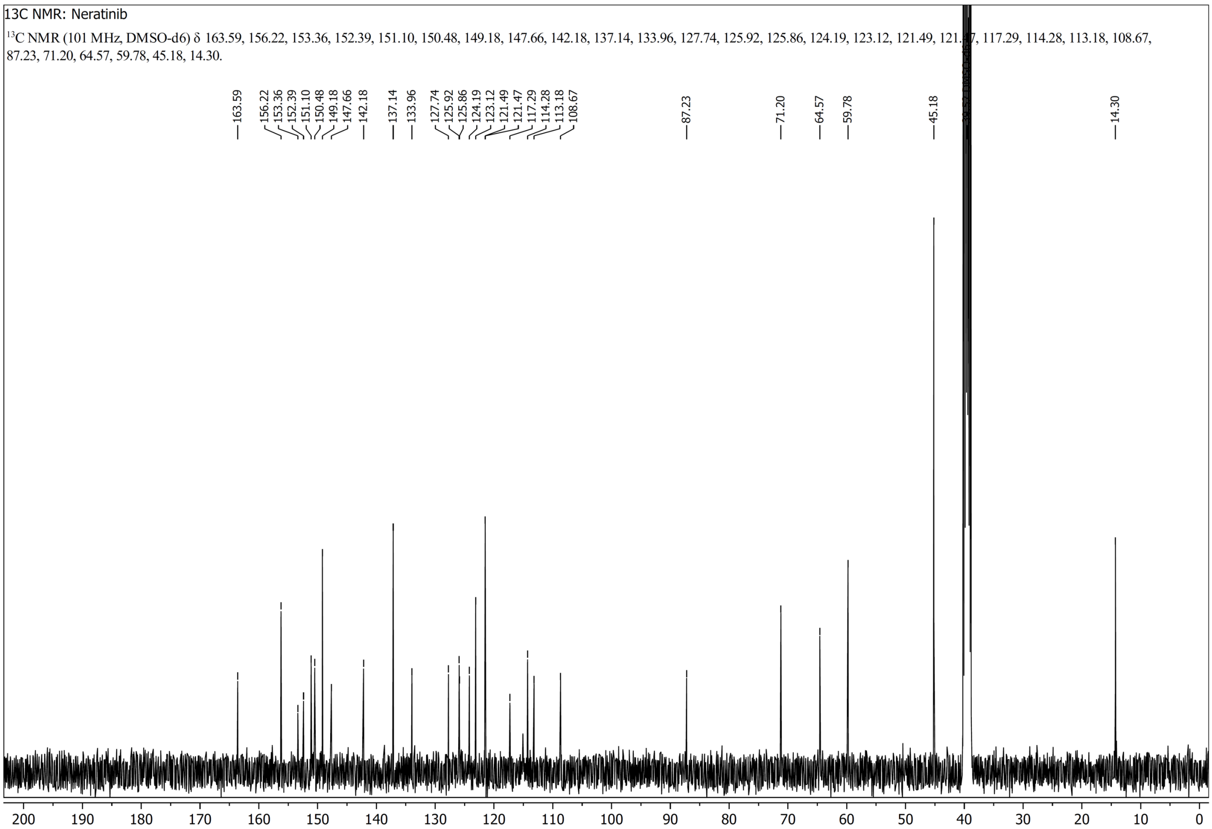

Figure S10. ^{13}C NMR of **Neratinib** in DMSO-d_6.

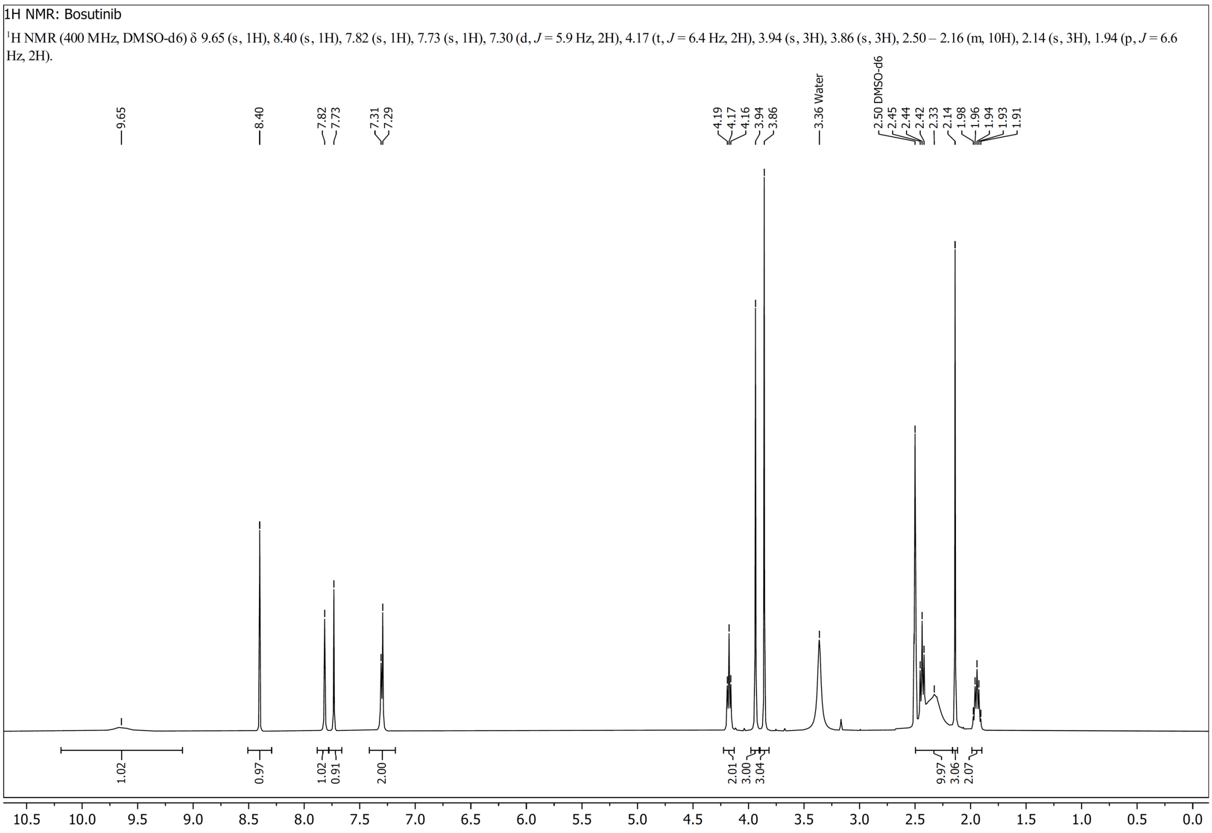

Figure S11. ^{1}H NMR of **Bosutinib** in DMSO-d_6.

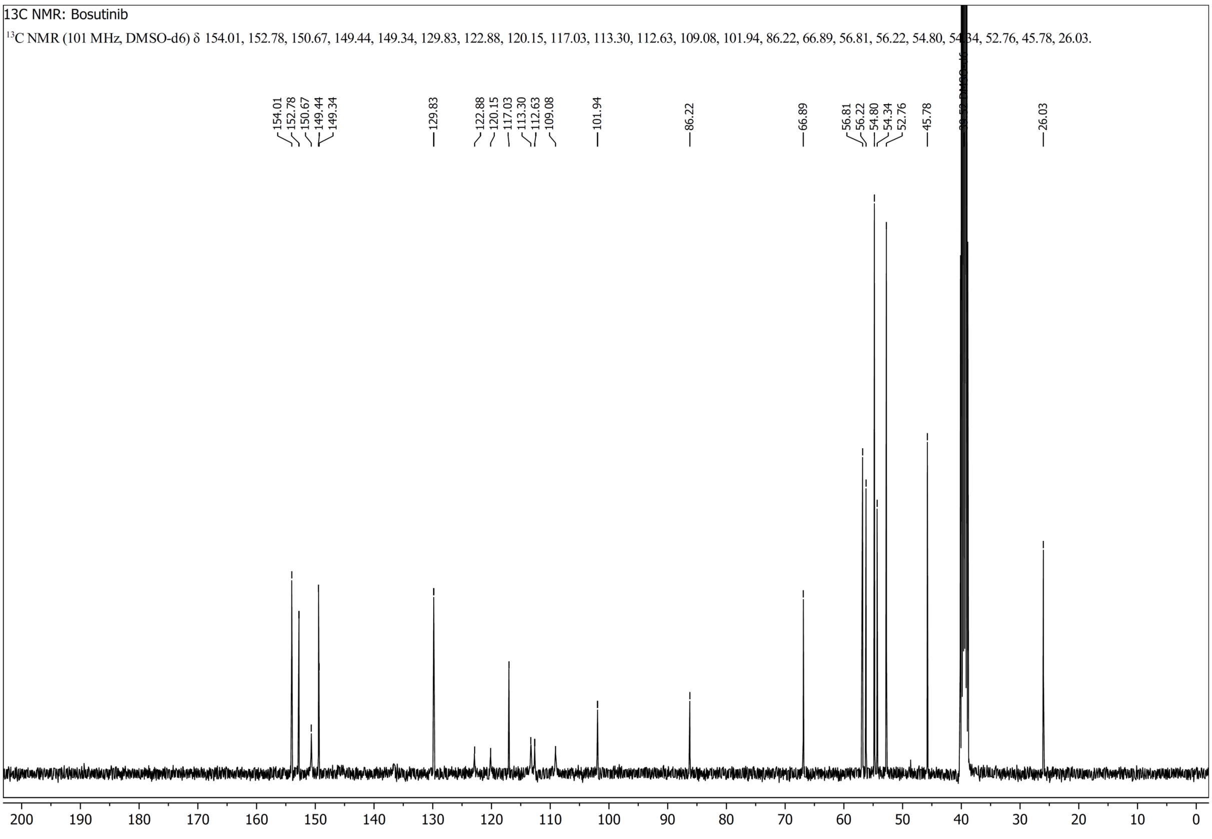

Figure S12. ^{13}C NMR of **Bosutinib** in DMSO-d_6.

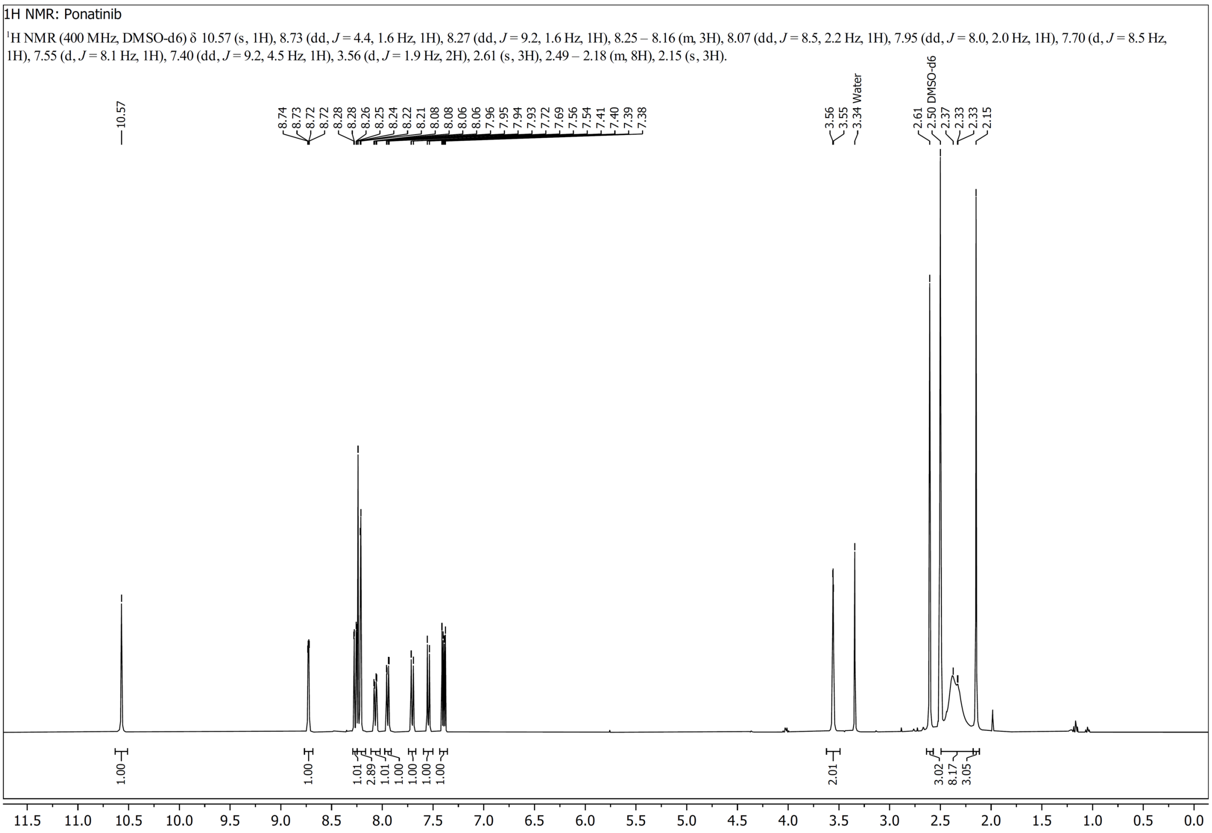

Figure S13. ^{1}H NMR of **Ponatinib** in DMSO-d_6.

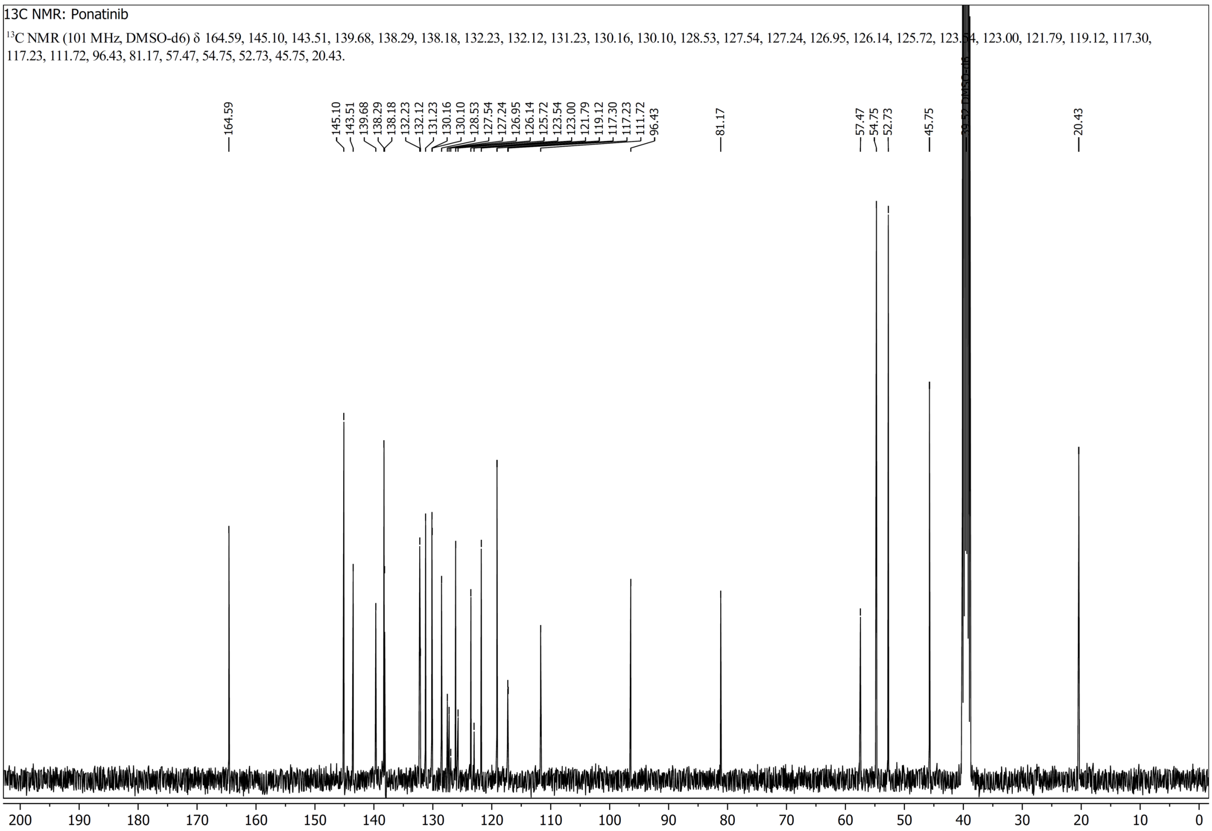

Figure S14. ^{13}C NMR of **Ponatinib** in DMSO-d_6.

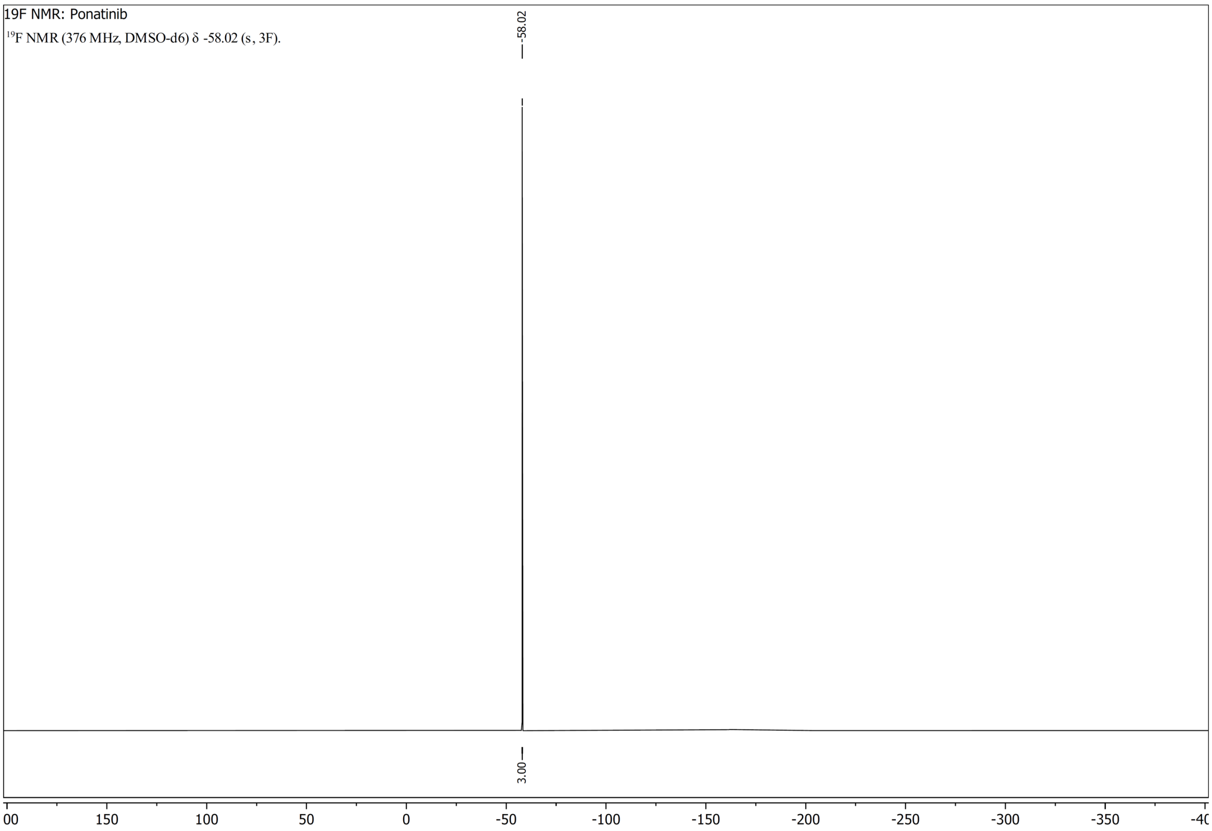

Figure S15. ^{19}F NMR of **Ponatinib** in DMSO-*d*$_6$.

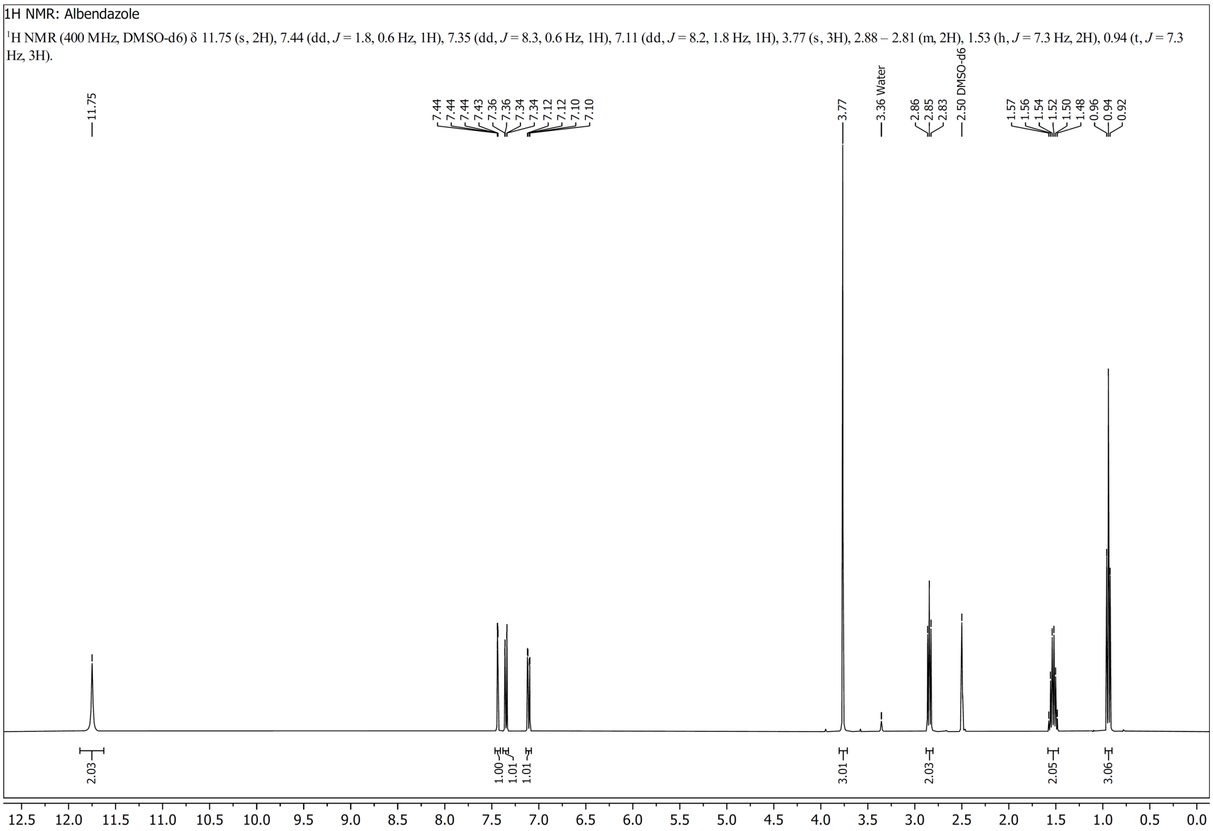

Figure S16. ^{1}H NMR of **Albendazole** in DMSO-*d*$_6$.

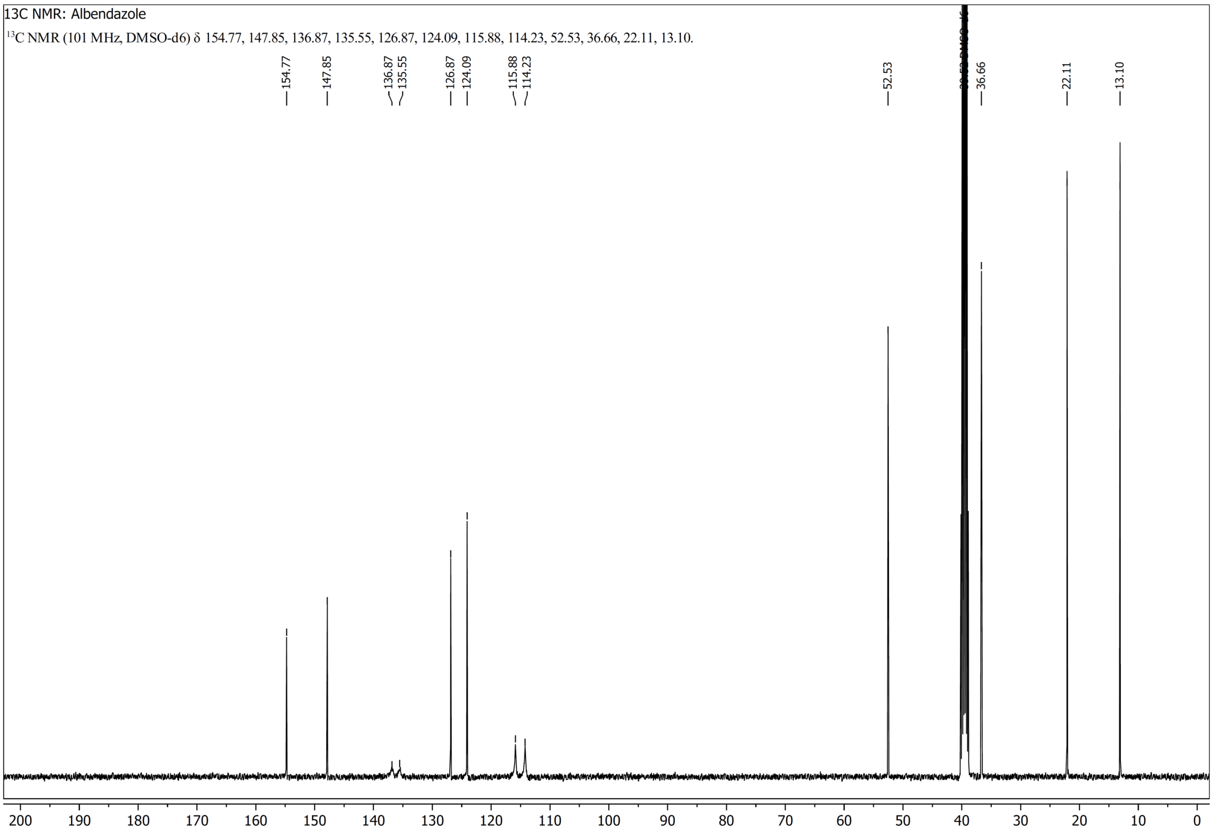

Figure S17. ^{13}C NMR of **Albendazole** in DMSO-*d*$_6$.

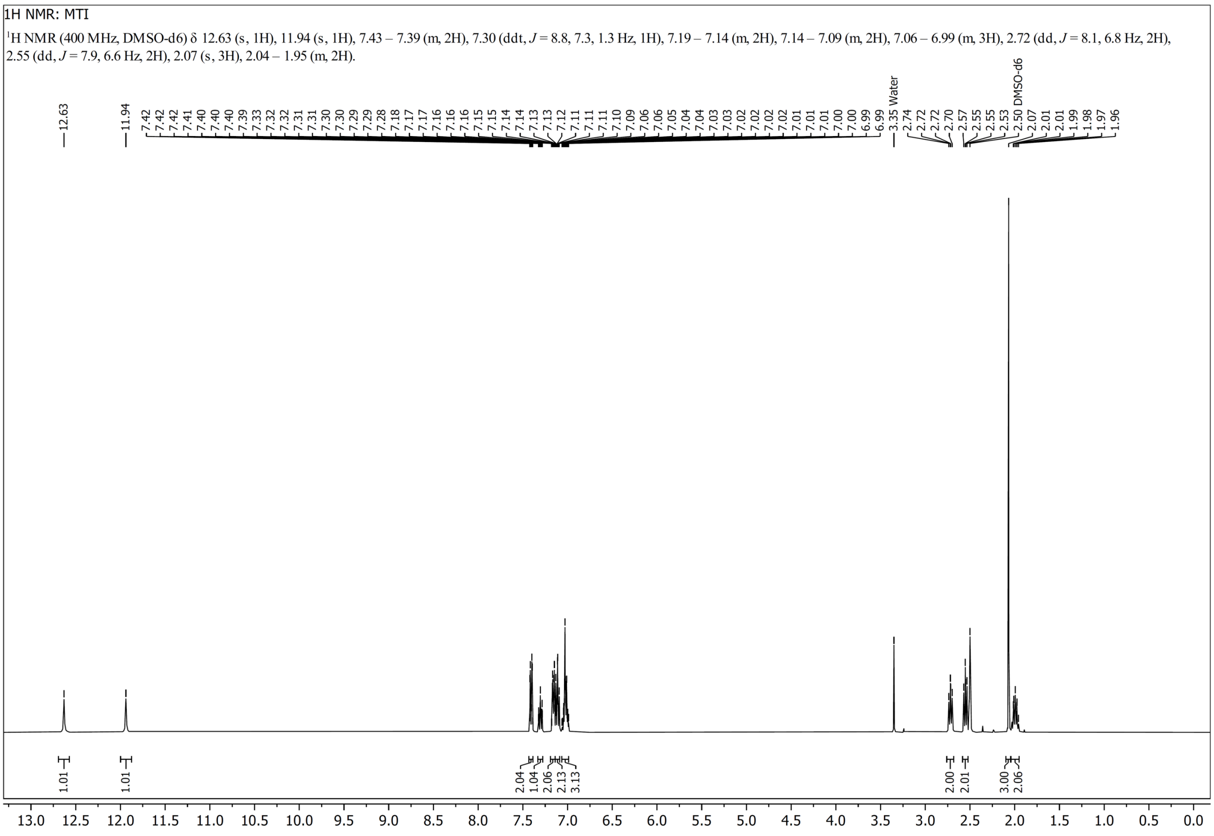

Figure S18. ^{1}H NMR of **MTI** in DMSO-*d*$_6$.

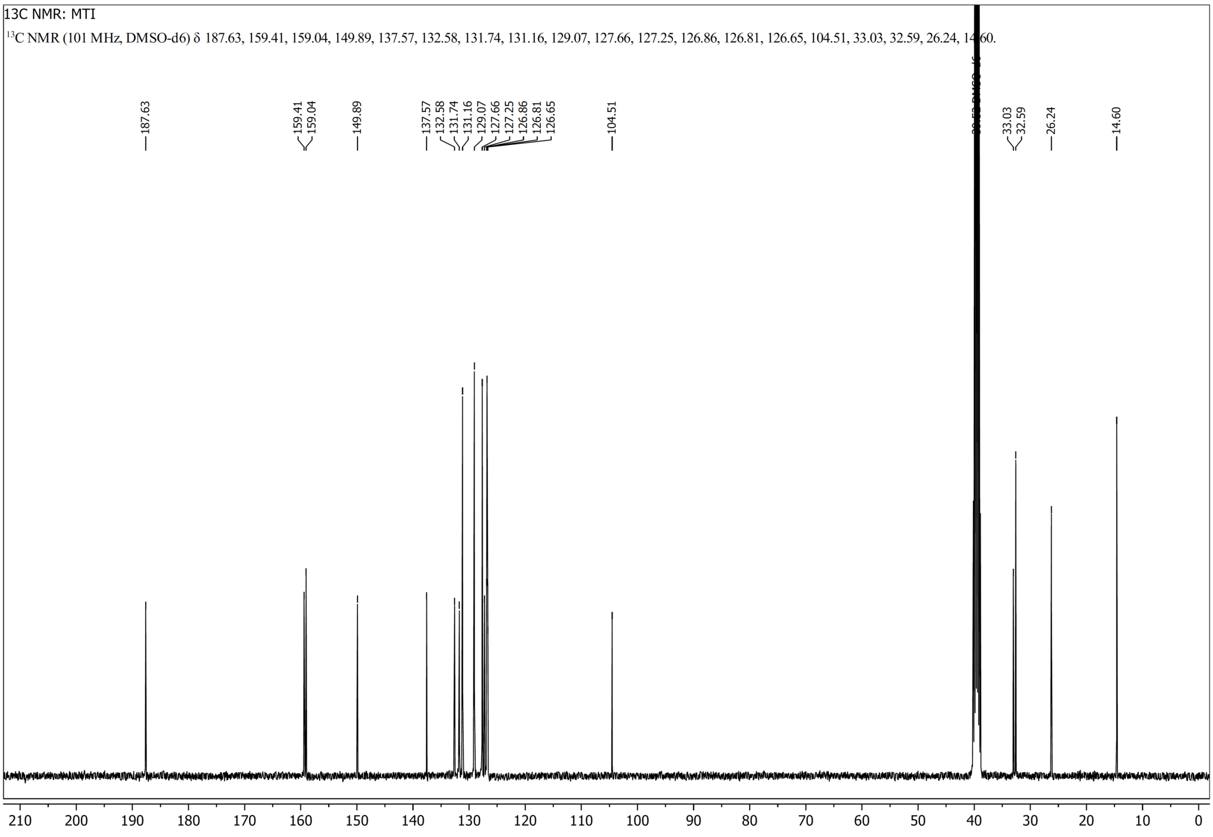

Figure S19. ^{13}C NMR of **MTI** in DMSO-d_6.

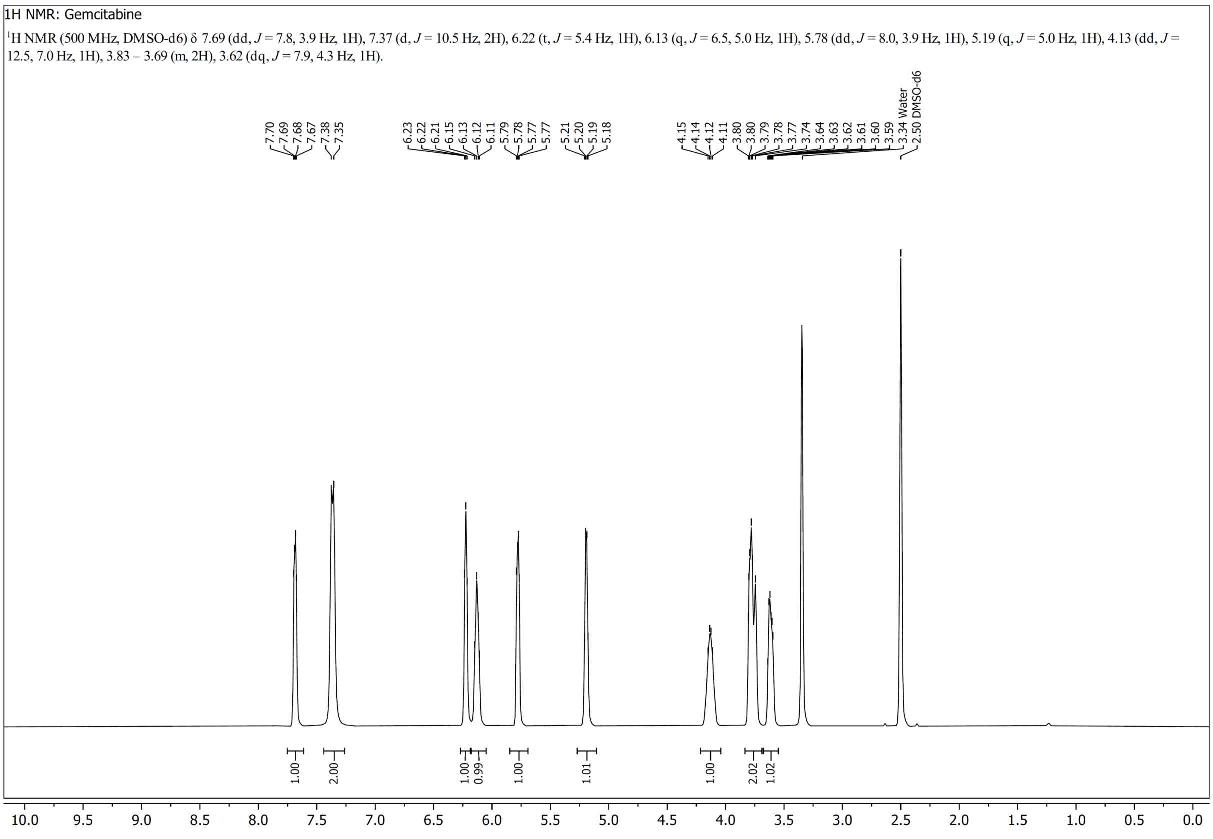

Figure S20. ^{1}H NMR of **Gemcitabine** in DMSO-d_6.

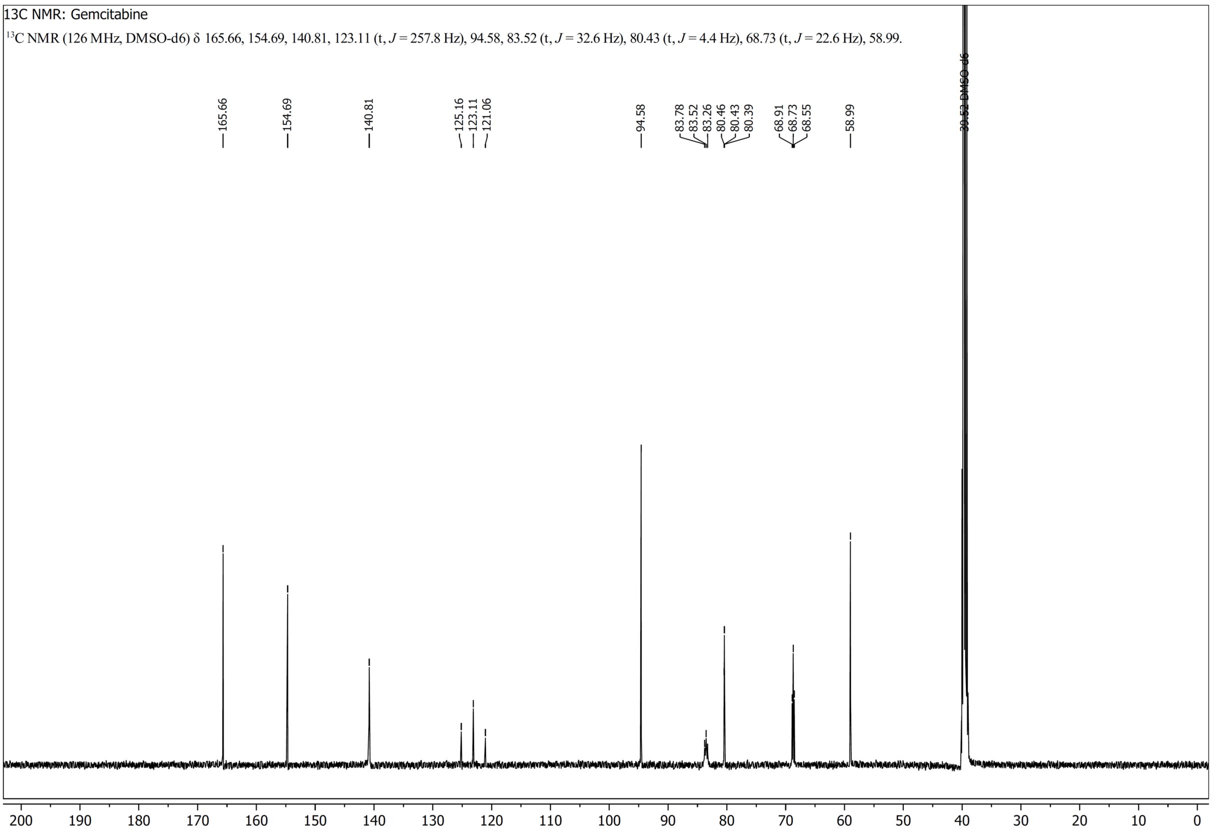

Figure S21. ^{13}C NMR of **Gemcitabine** in DMSO-d_6.

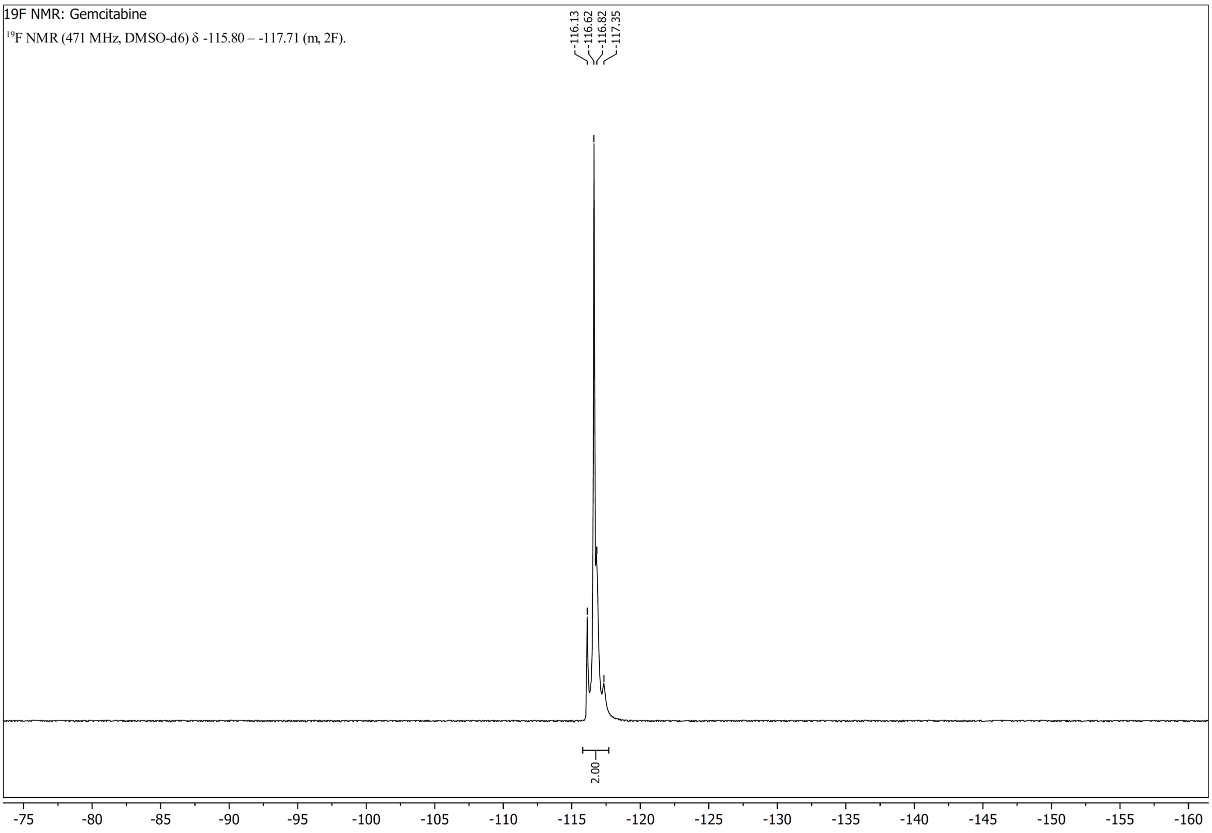

Figure S22. ^{19}F NMR of **Gemcitabine** in DMSO-d_6.

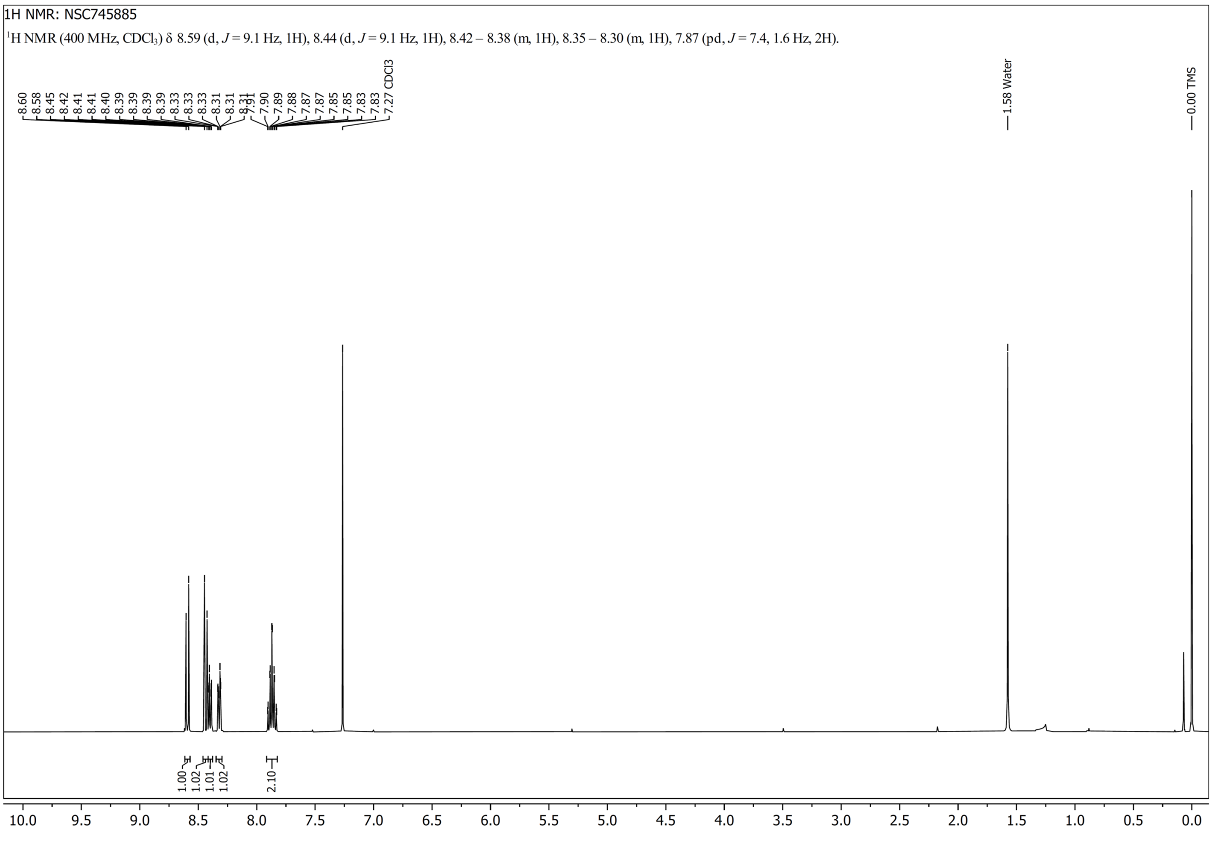

Figure S23. ^{1}H NMR of **NSC745885** in $CDCl_3$.

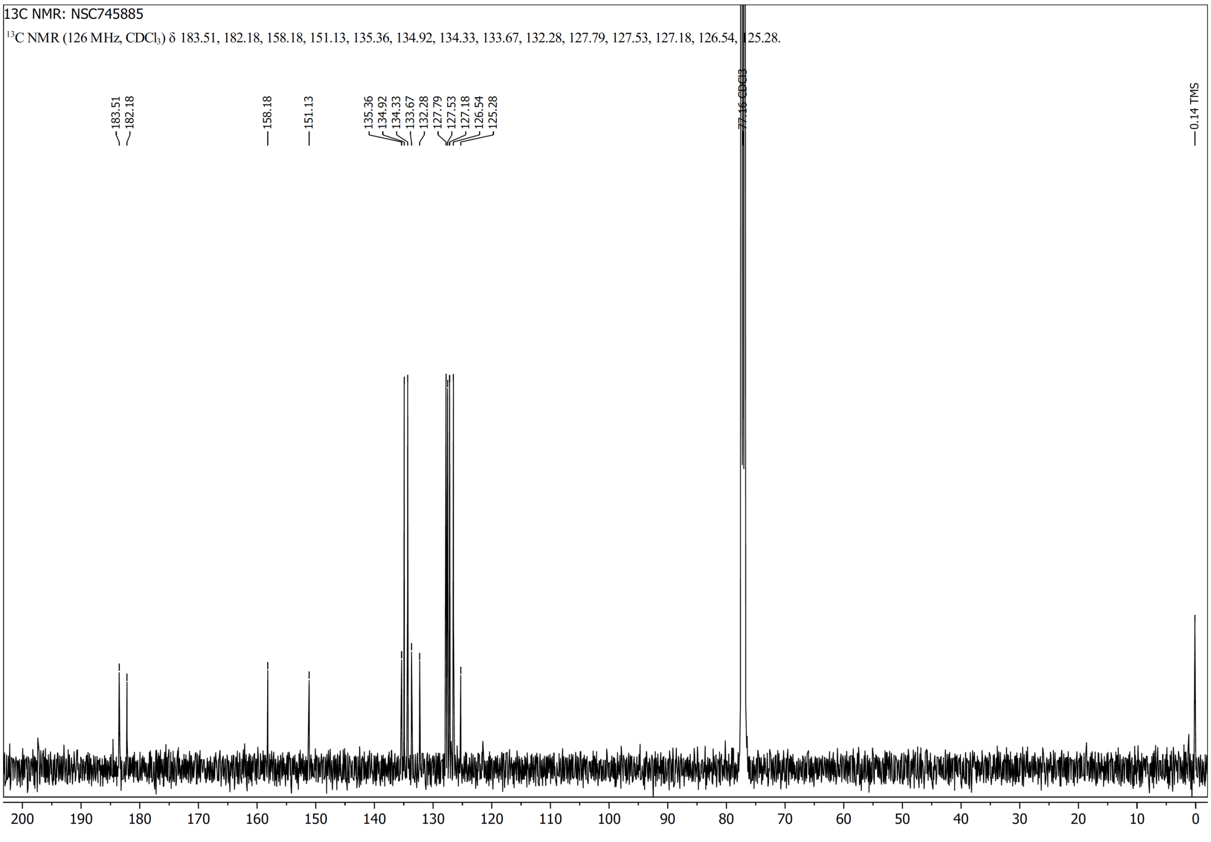

Figure S24. ^{13}C NMR of **NSC745885** in $CDCl_3$.

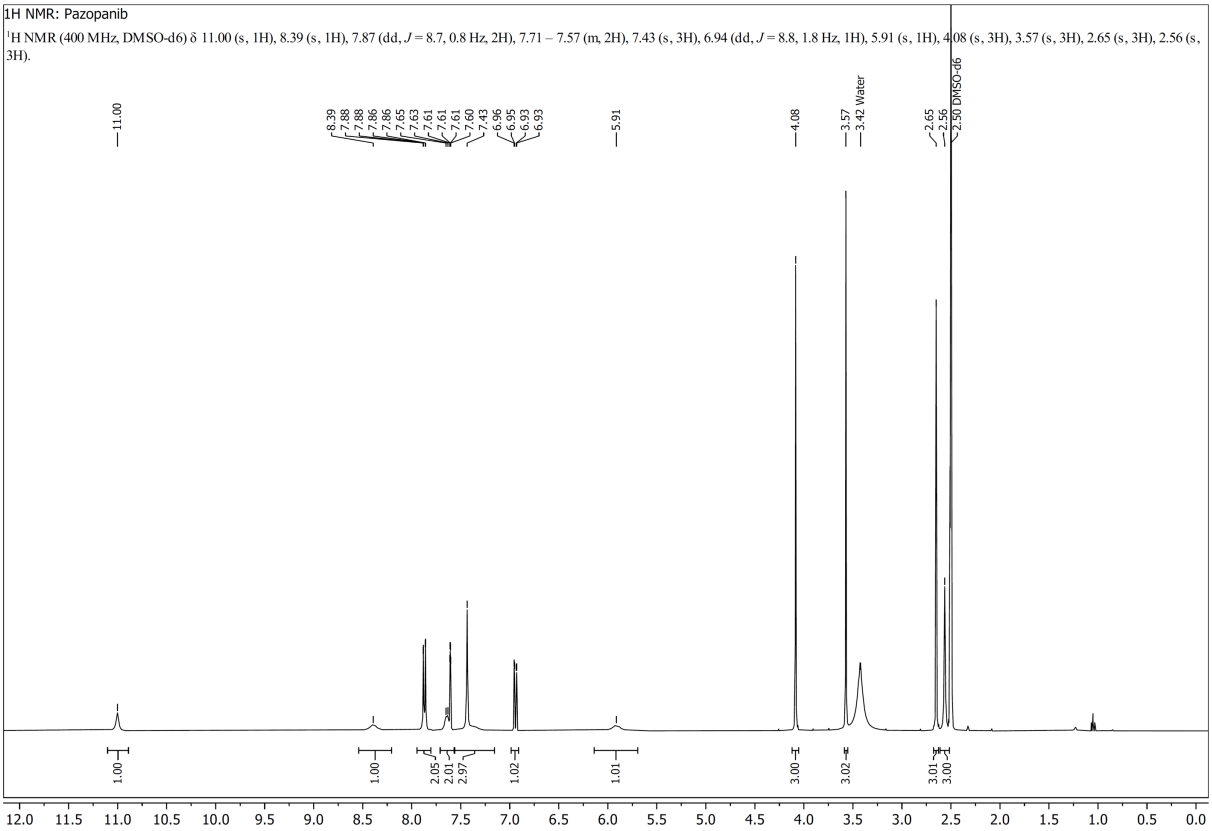

Figure S25. ^{1}H NMR of **Pazopanib** in DMSO-d_6.

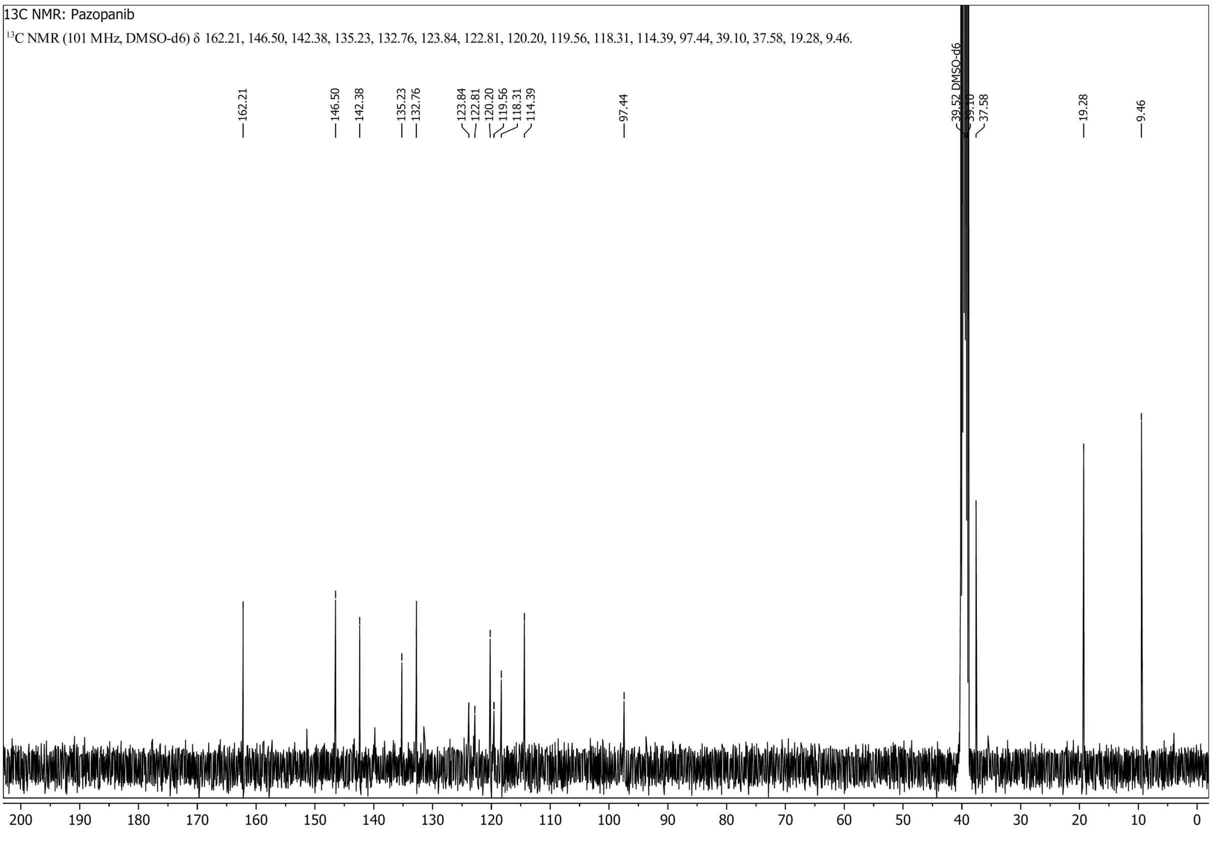

Figure S26. ^{13}C NMR of **Pazopanib** in DMSO-d_6.

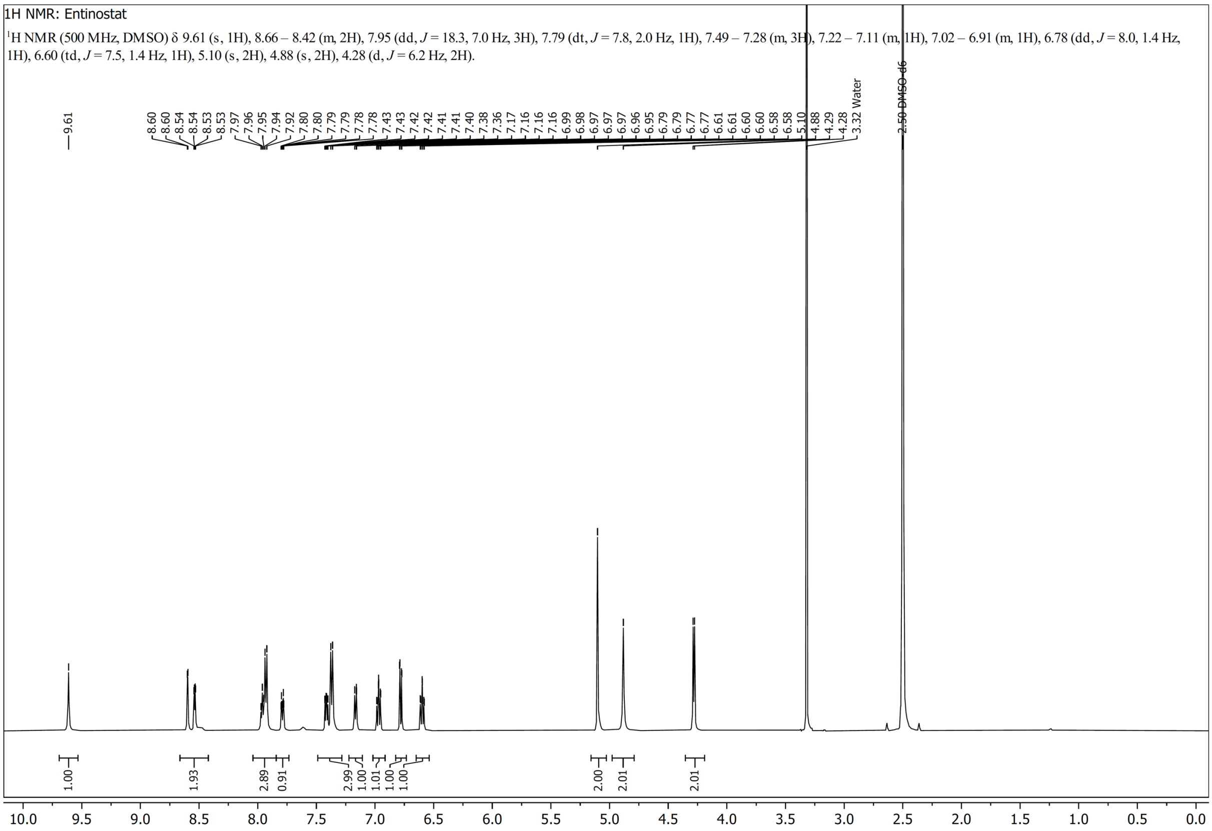

Figure S27. ^{1}H NMR of **Entinostat** in DMSO-d_6.

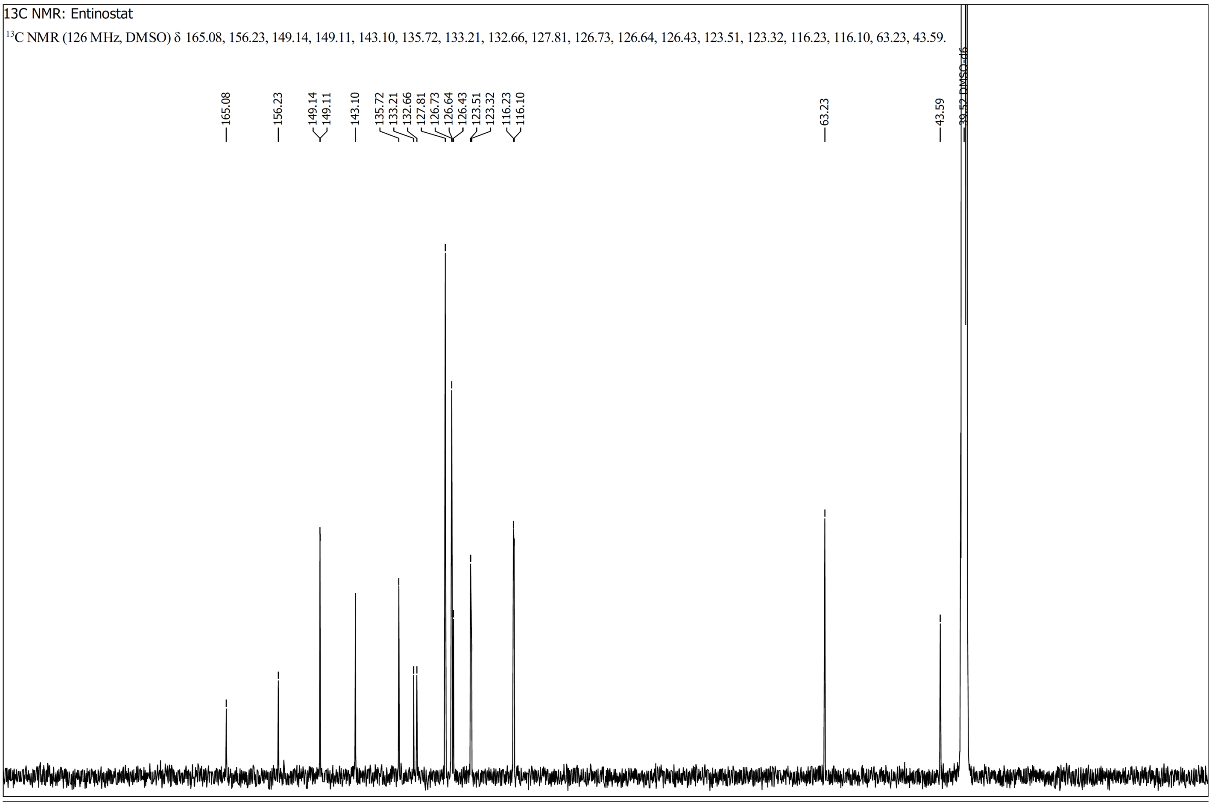

Figure S28. ^{13}C NMR of **Entinostat** in DMSO-d_6.

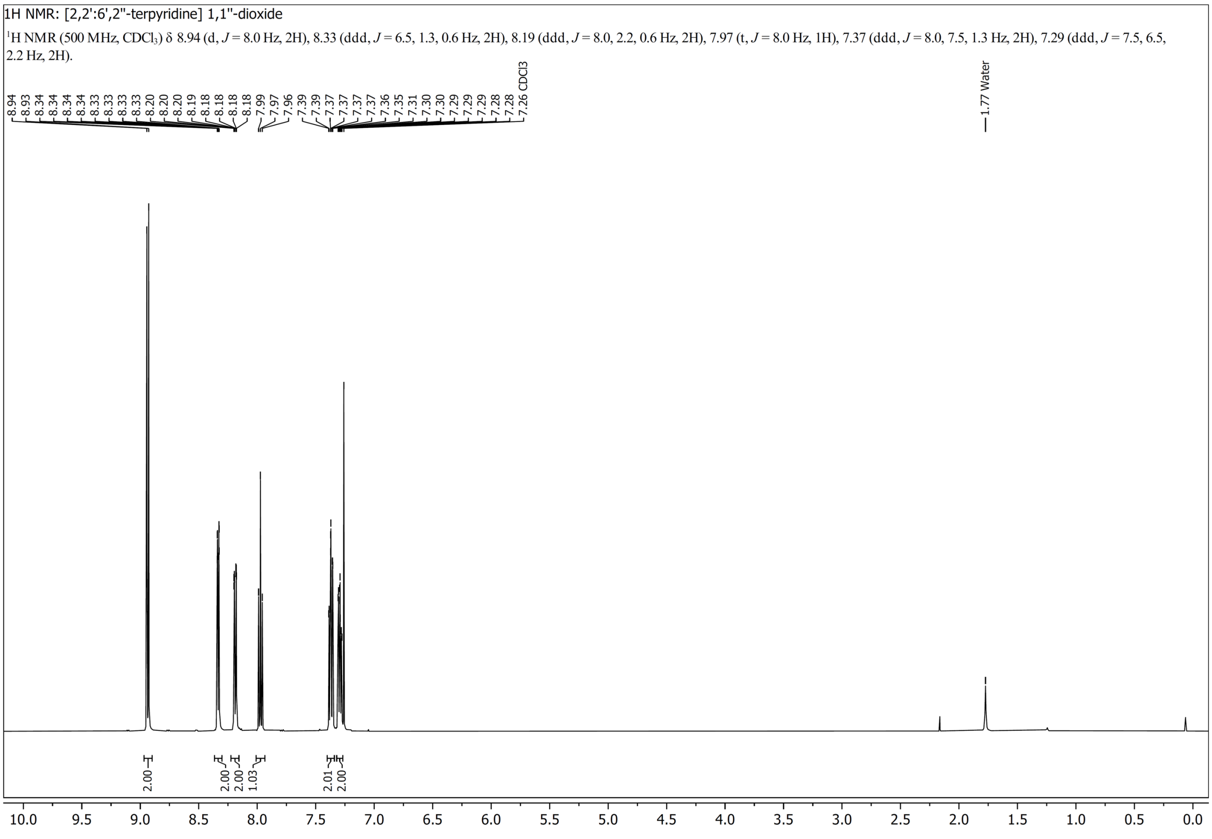

Figure S29. ^{1}H NMR of **1** in $CDCl_3$.

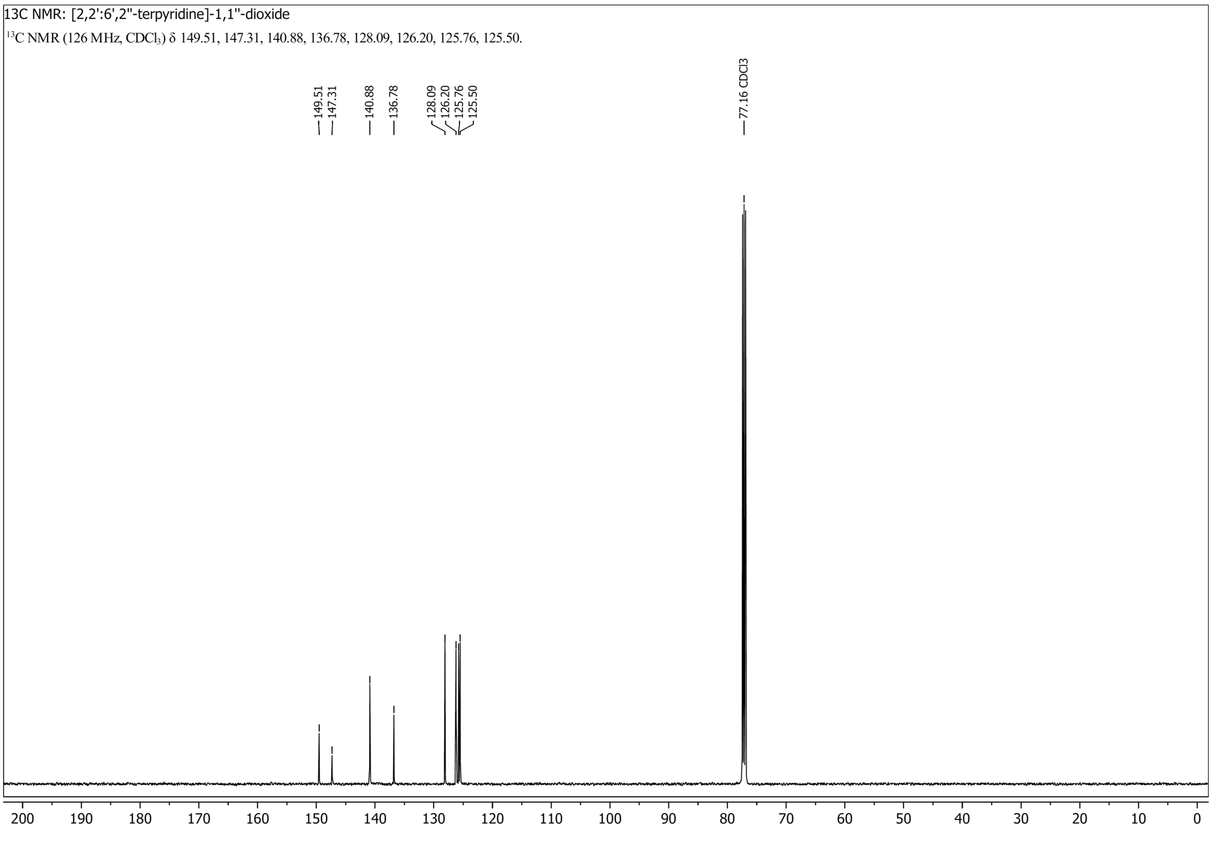

Figure S30. ^{13}C NMR of **1** in $CDCl_3$.

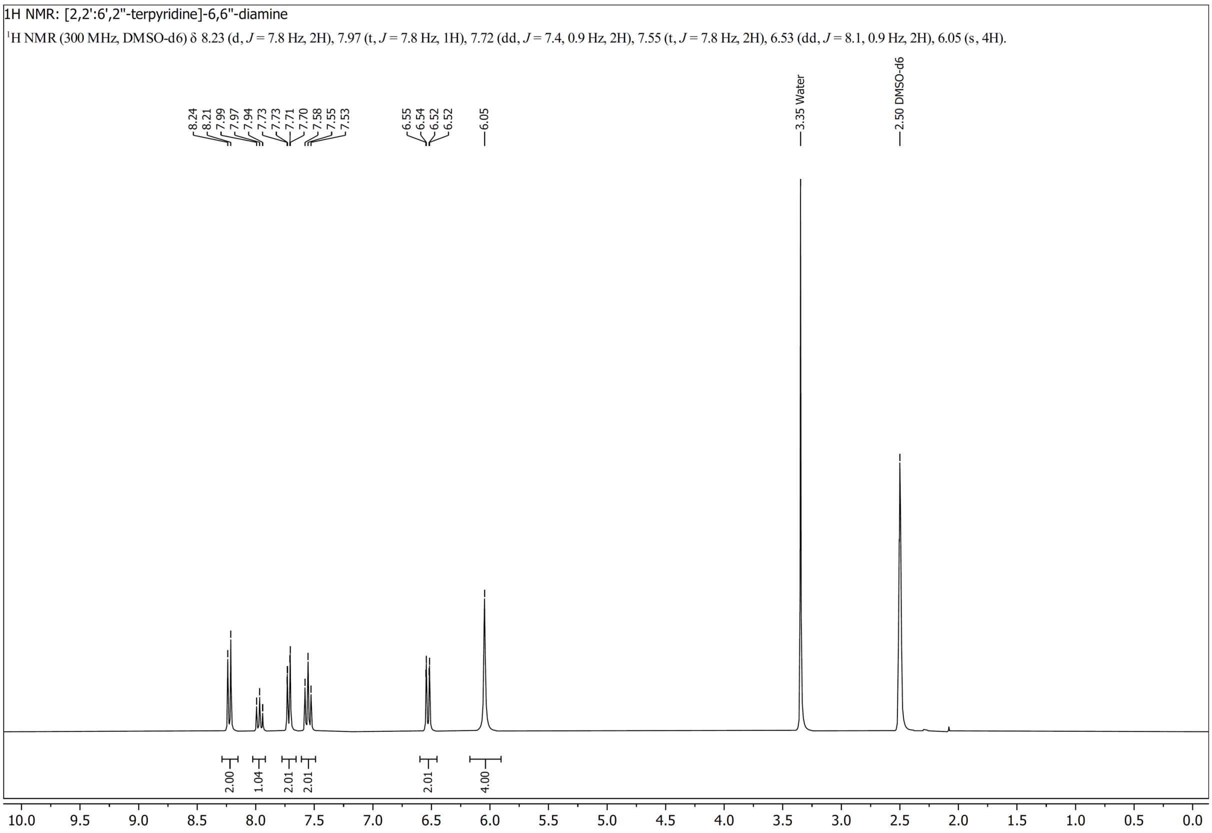

Figure S31. ^{1}H NMR of **2** in DMSO-d_6.

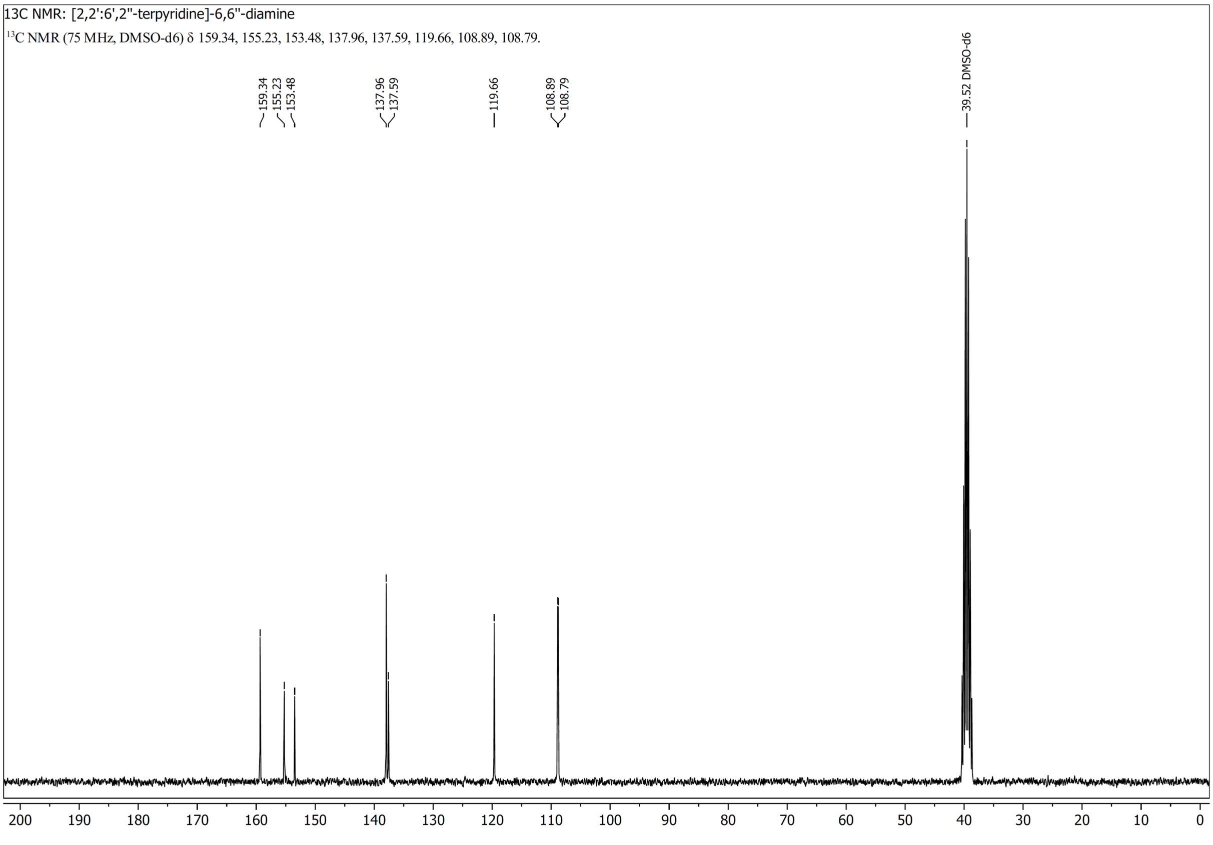

Figure S32. ^{13}C NMR of **2** in DMSO-d_6.

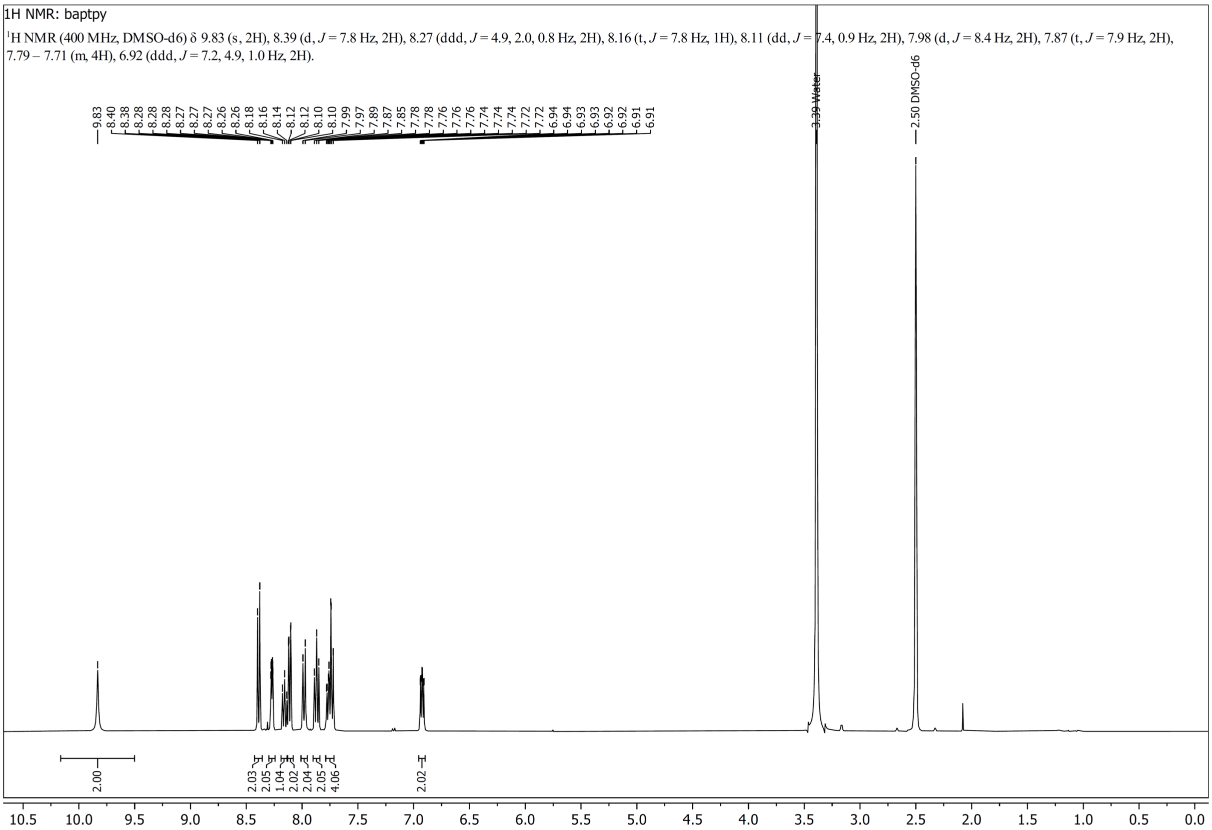

Figure S33. ^{1}H NMR of **3** in DMSO-d_6.

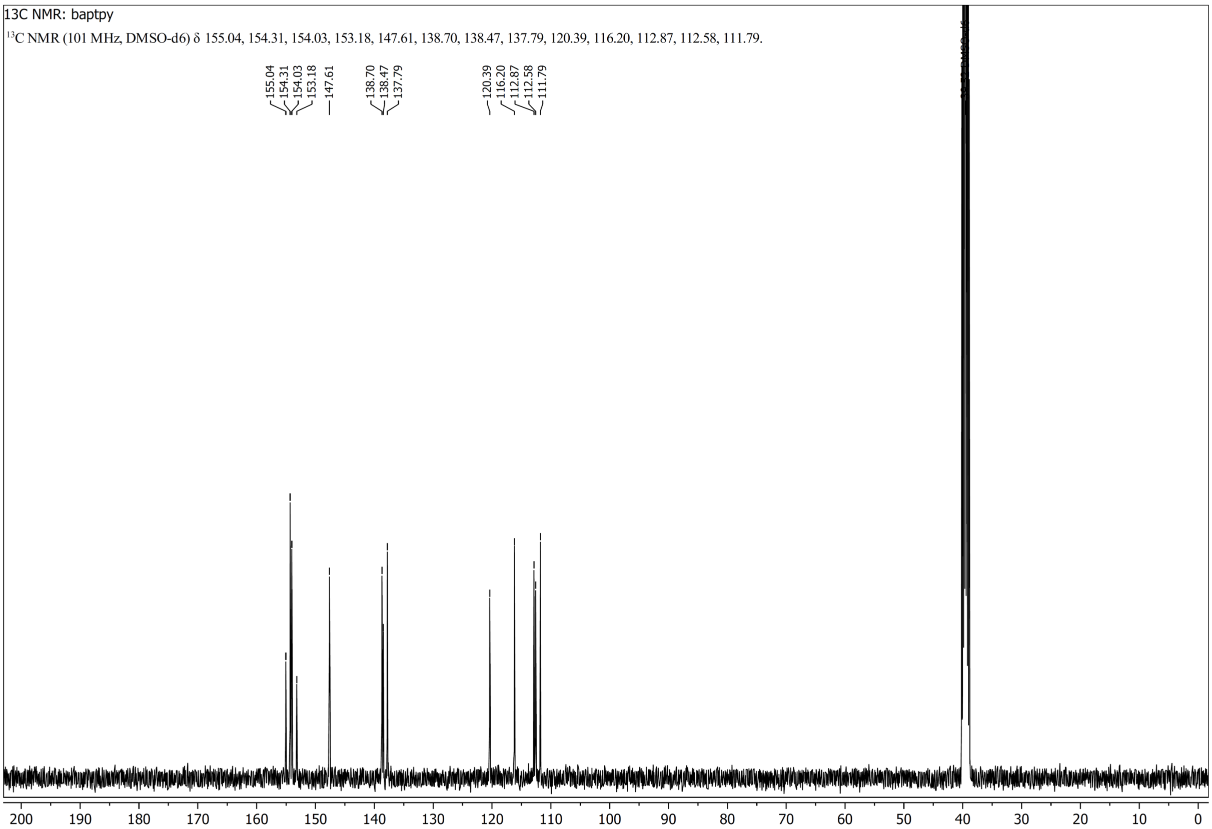

Figure S34. ^{13}C NMR of **3** in DMSO-d_6.

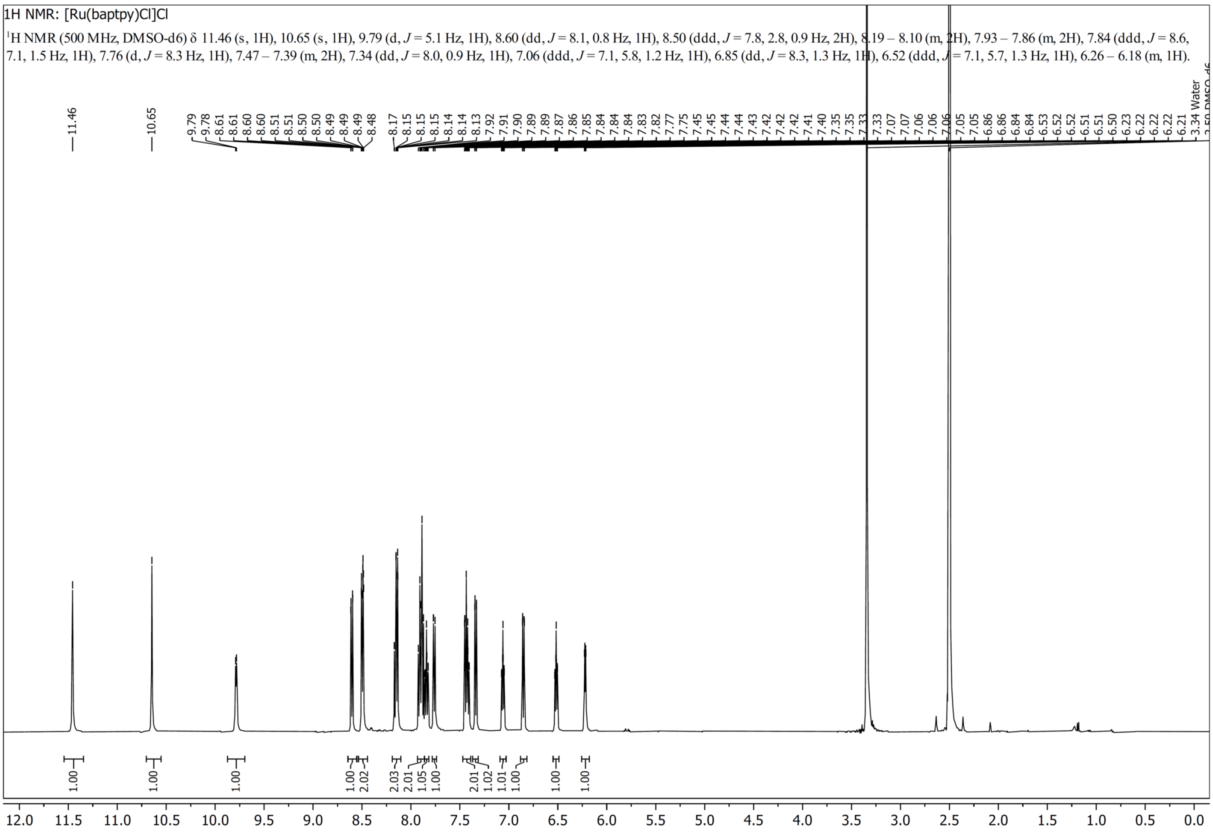

Figure S35. ^{1}H NMR of [**4**]Cl in DMSO-d_6.

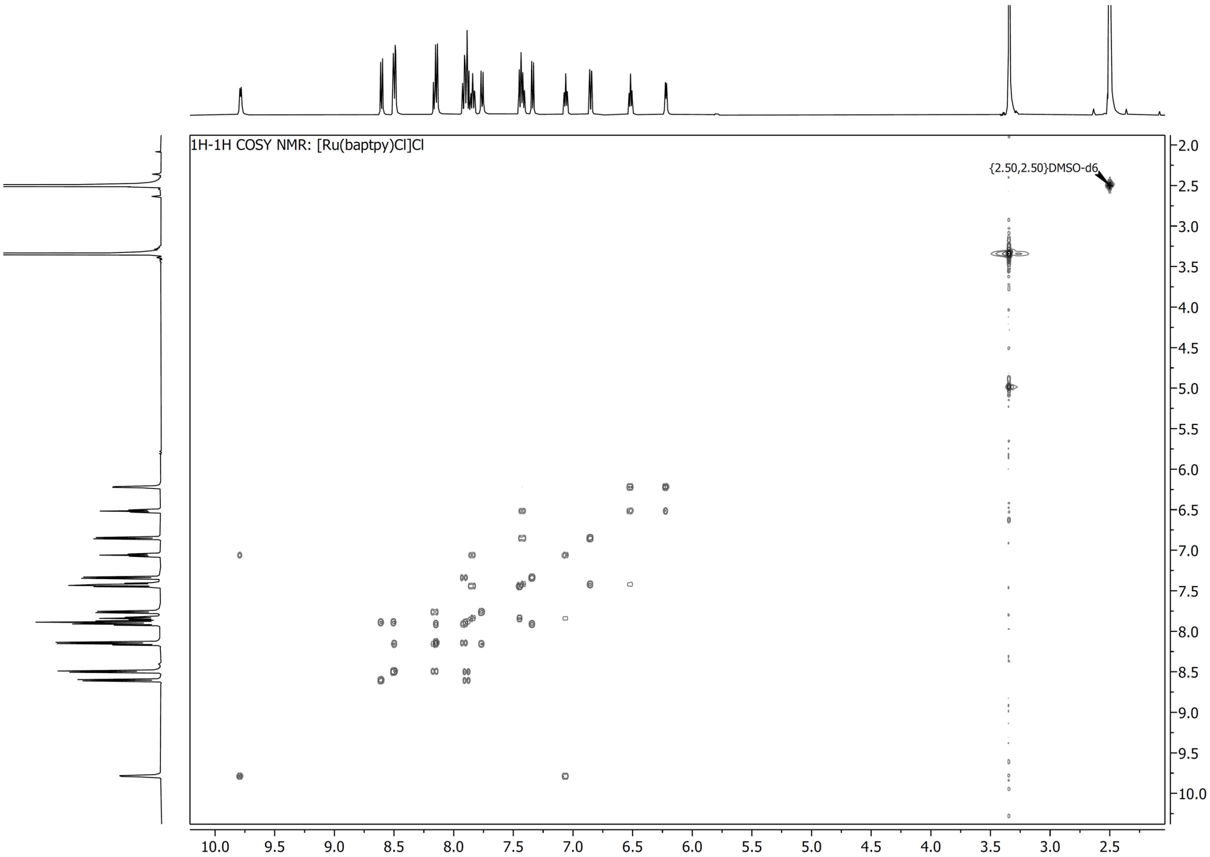

Figure S36. ^{1}H-^{1}H COSY NMR of [**4**]Cl in DMSO-d_6.

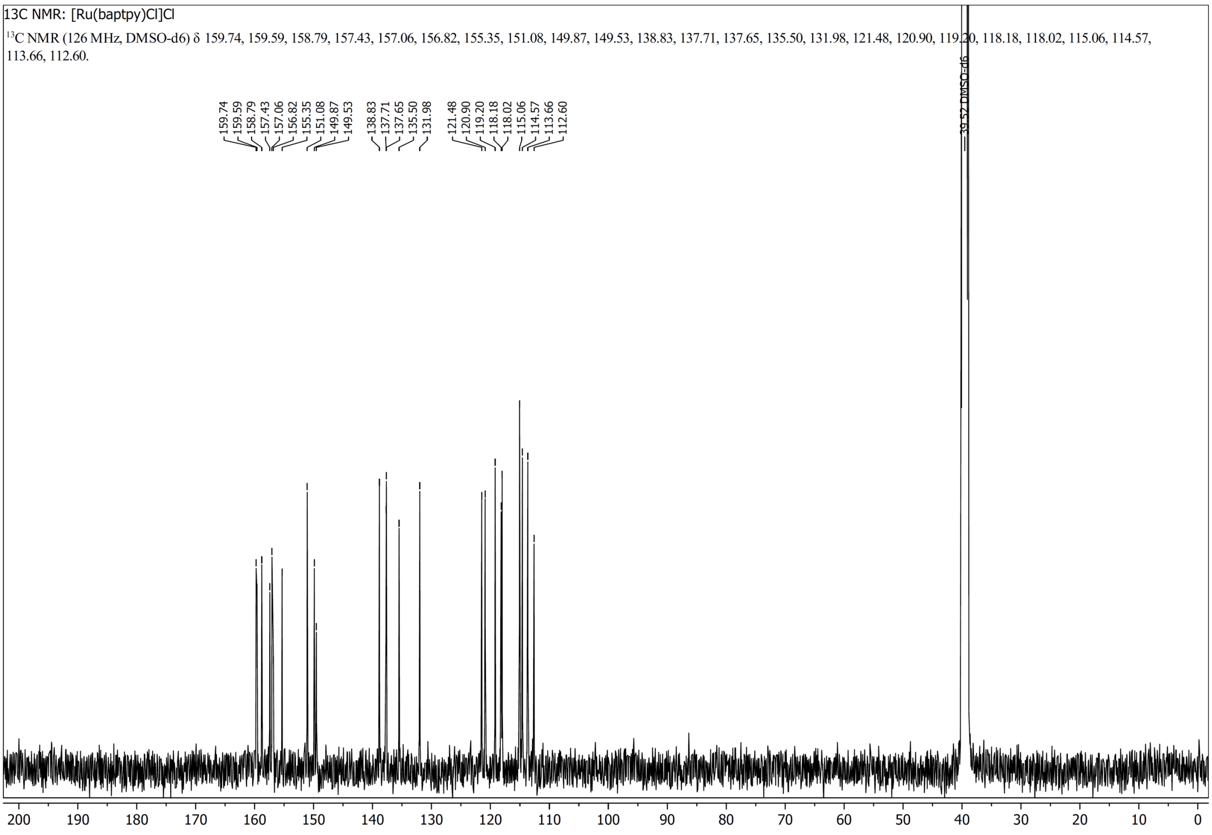

Figure S37. ^{13}C NMR of [**4**]Cl in DMSO-d_6.

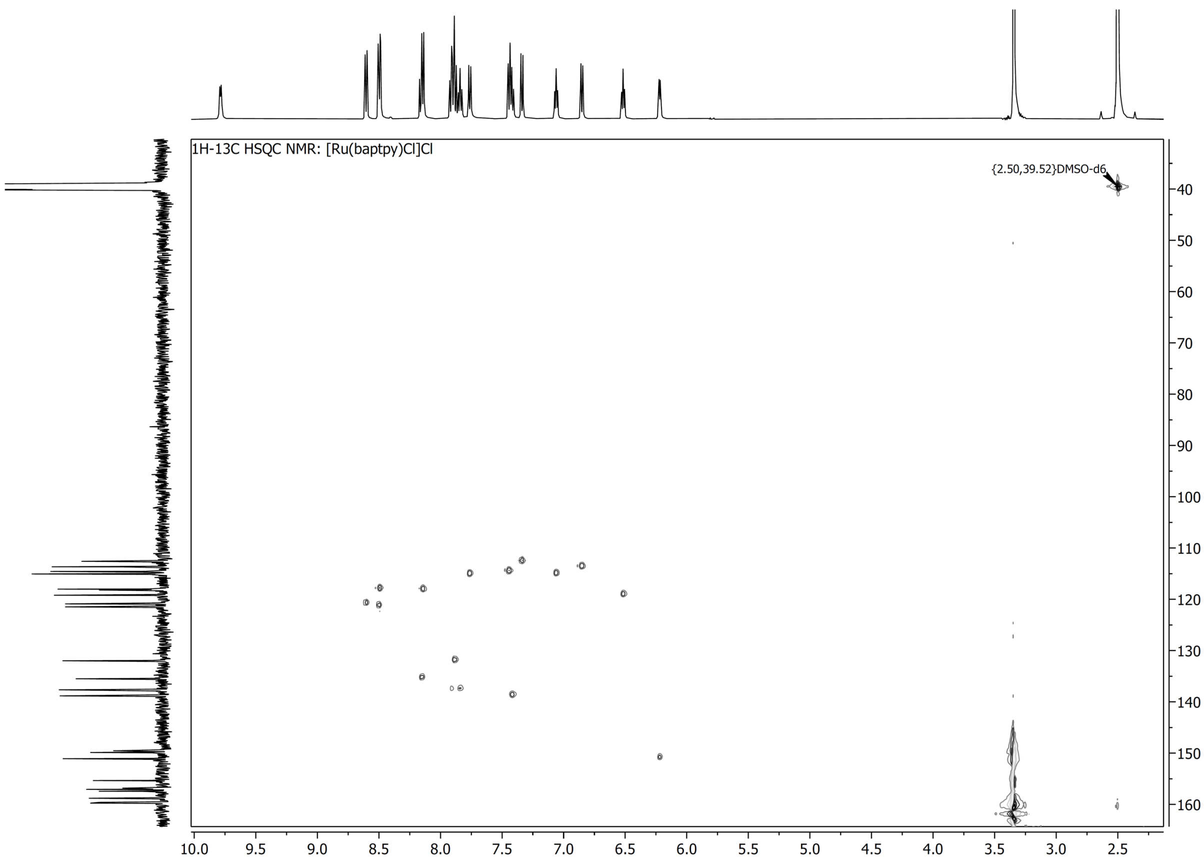

Figure S38. ^{1}H-^{13}C HSQC NMR of [**4**]Cl in DMSO-d_6.

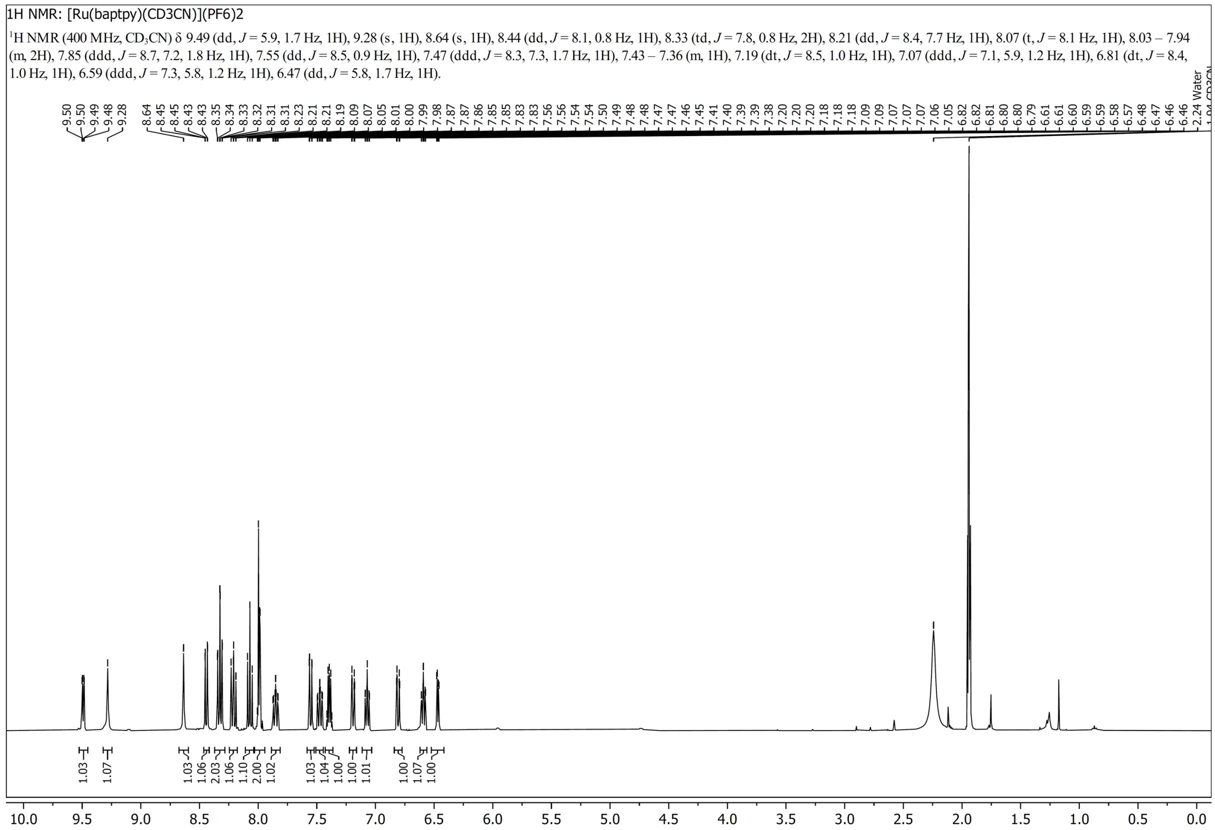

Figure S39. ^{1}H NMR of [**5**]$(PF_6)_2$ in CD_3CN.

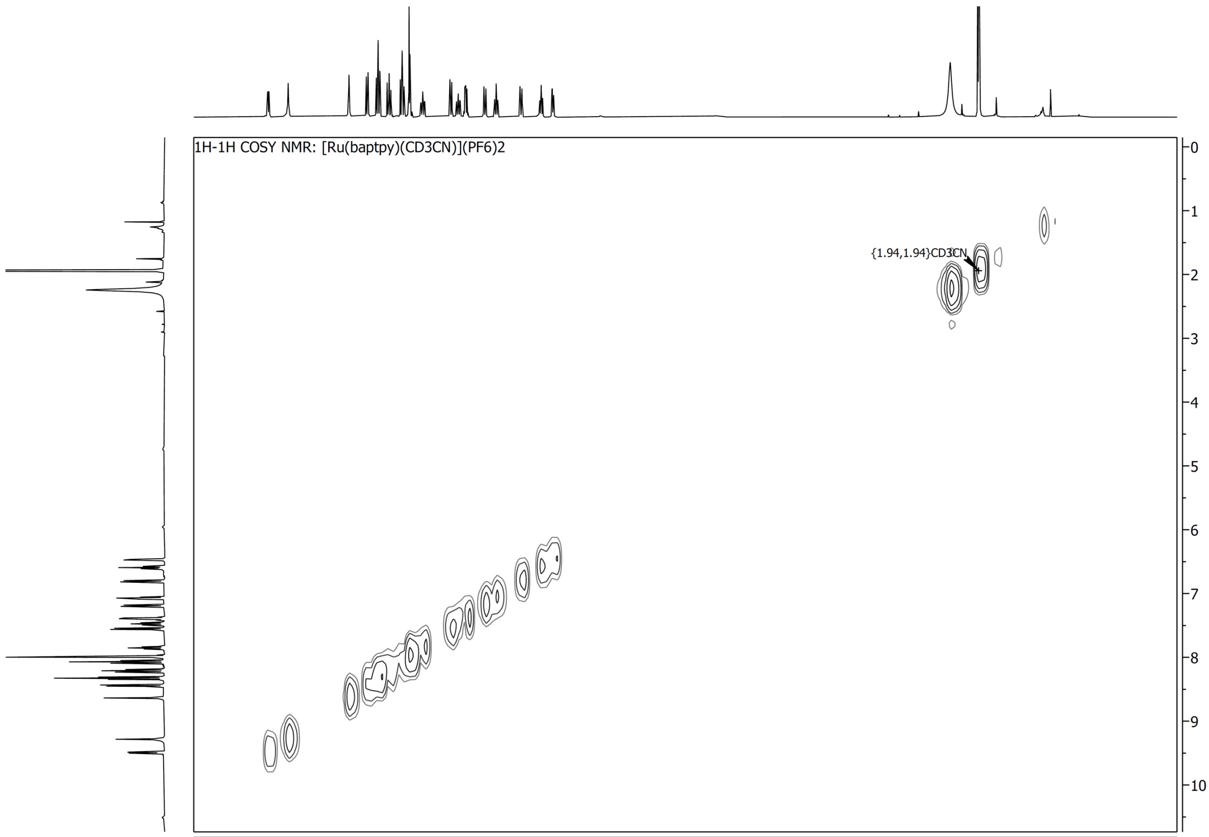

Figure S40. ^{1}H-^{1}H COSY NMR of [**5**]$(PF_6)_2$ in CD_3CN.

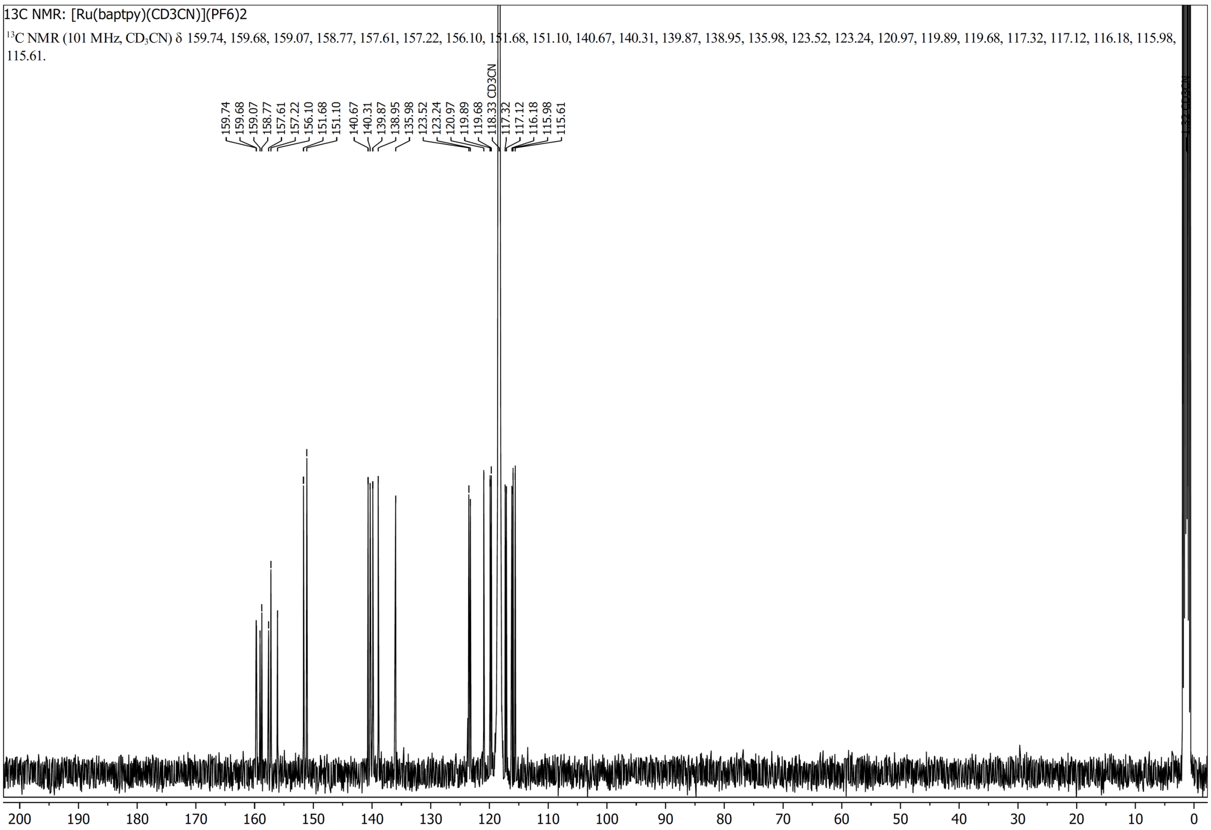

Figure S41. ^{13}C NMR of [**5**]$(PF_6)_2$ in CD_3CN.

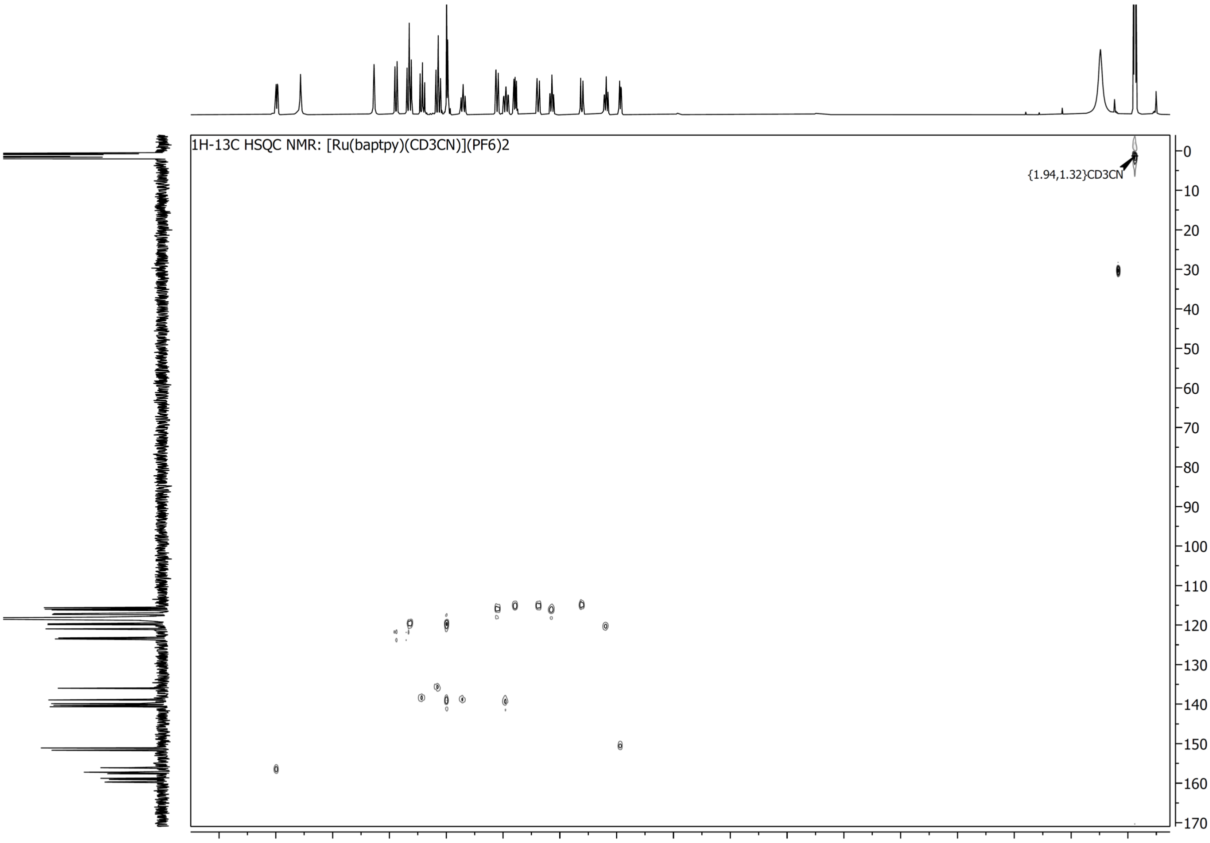

Figure S42. ^{1}H-^{13}C HSQC NMR of [**5**]$(PF_6)_2$ in CD_3CN.

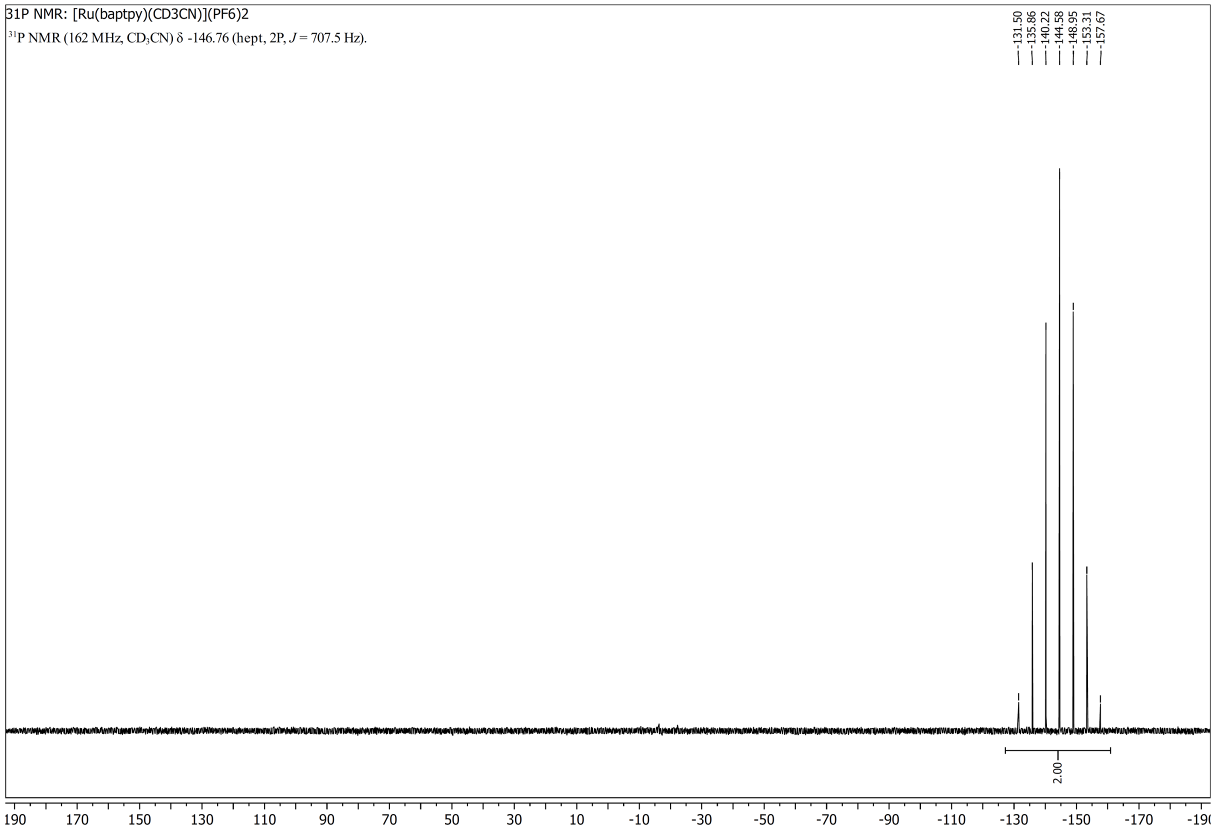

Figure S43. ^{31}P NMR of [**5**]$(PF_6)_2$ in CD_3CN.

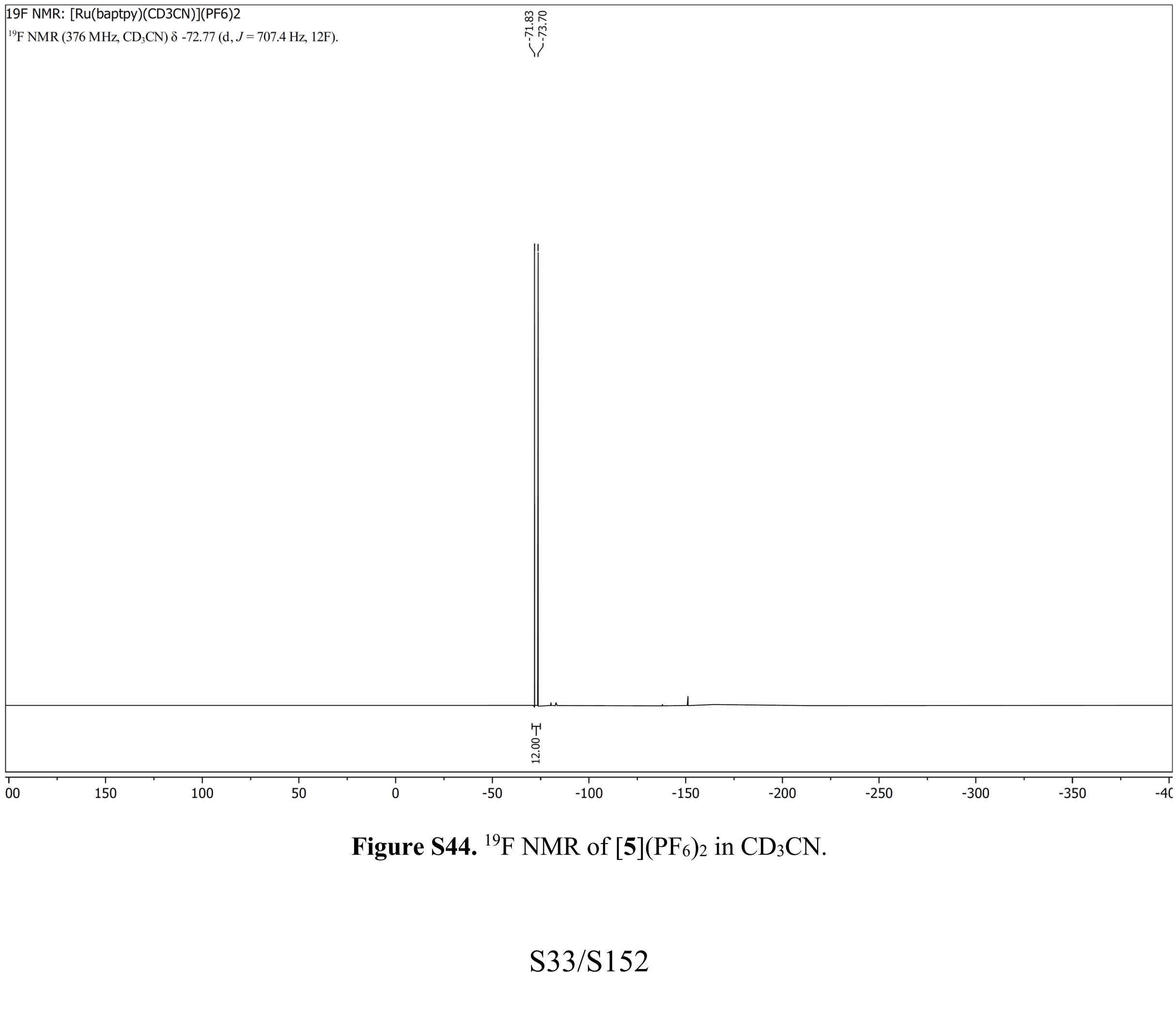

Figure S44. ^{19}F NMR of [**5**]$(PF_6)_2$ in CD_3CN.

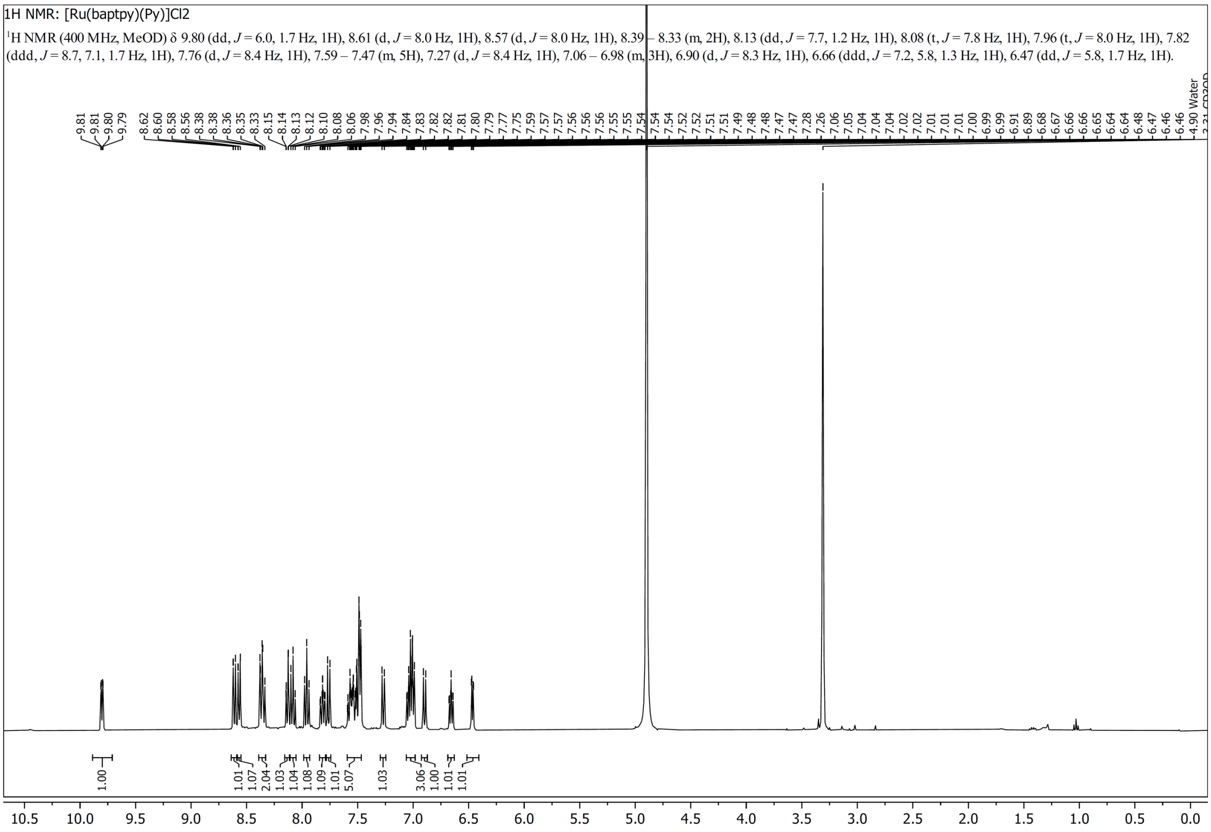

Figure S45. ^{1}H NMR of [**6**]Cl_2 in CD_3OD.

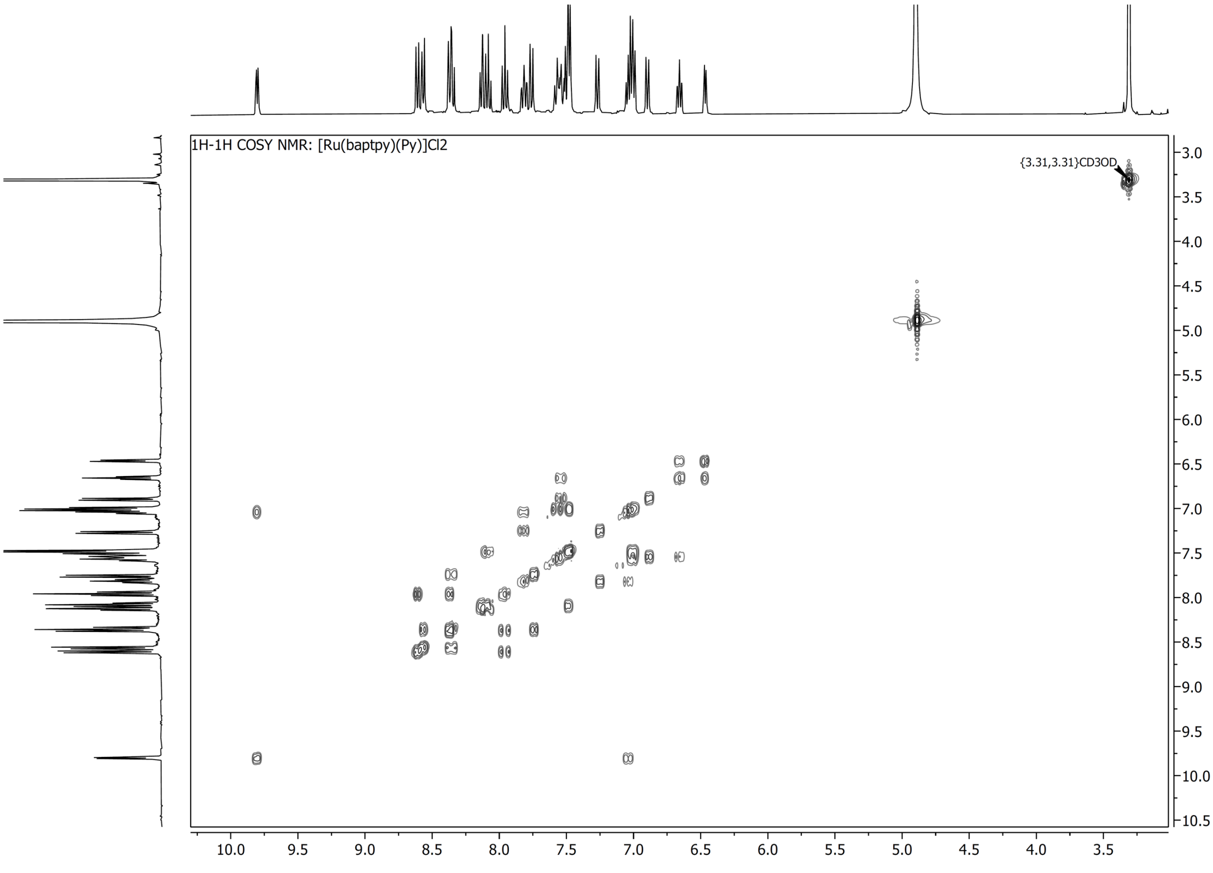

Figure S46. ^{1}H-^{1}H COSY NMR of [**6**]Cl_2 in CD_3OD.

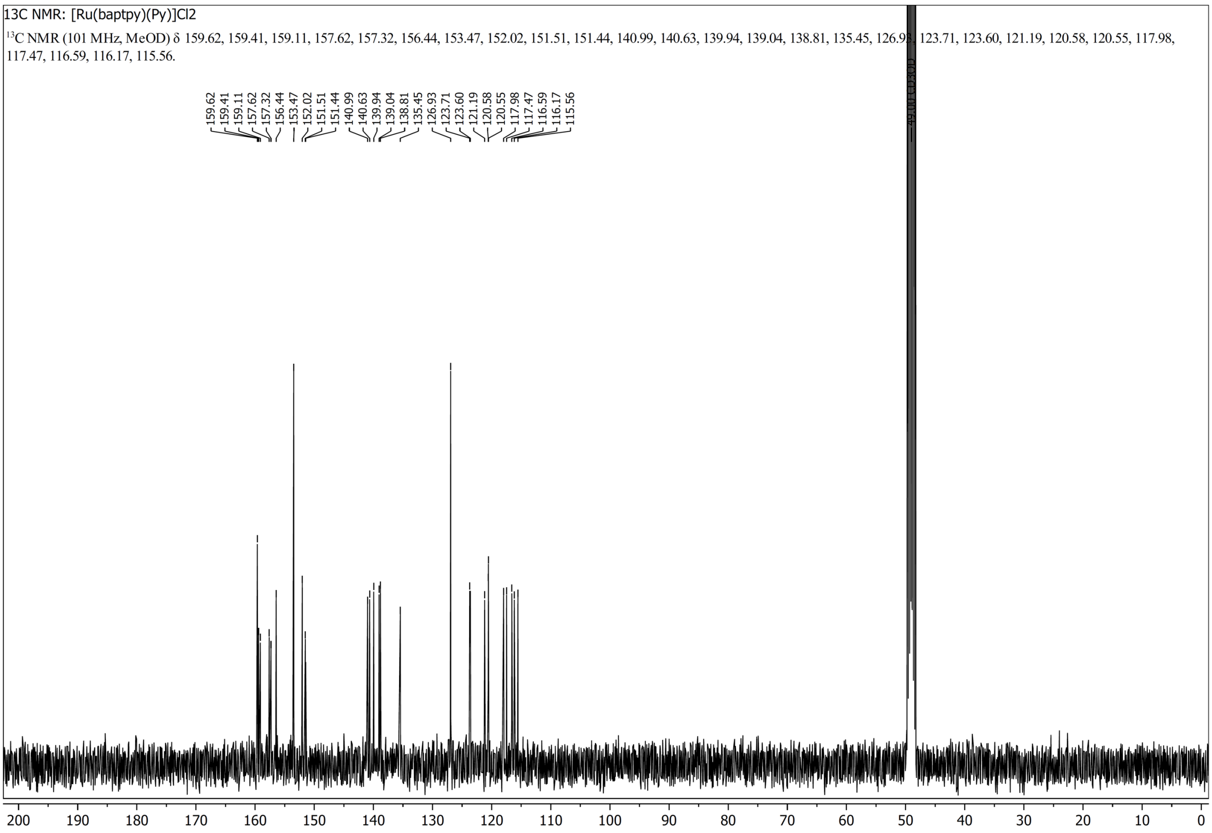

Figure S47. ^{13}C NMR of [**6**]Cl_2 in CD_3OD.

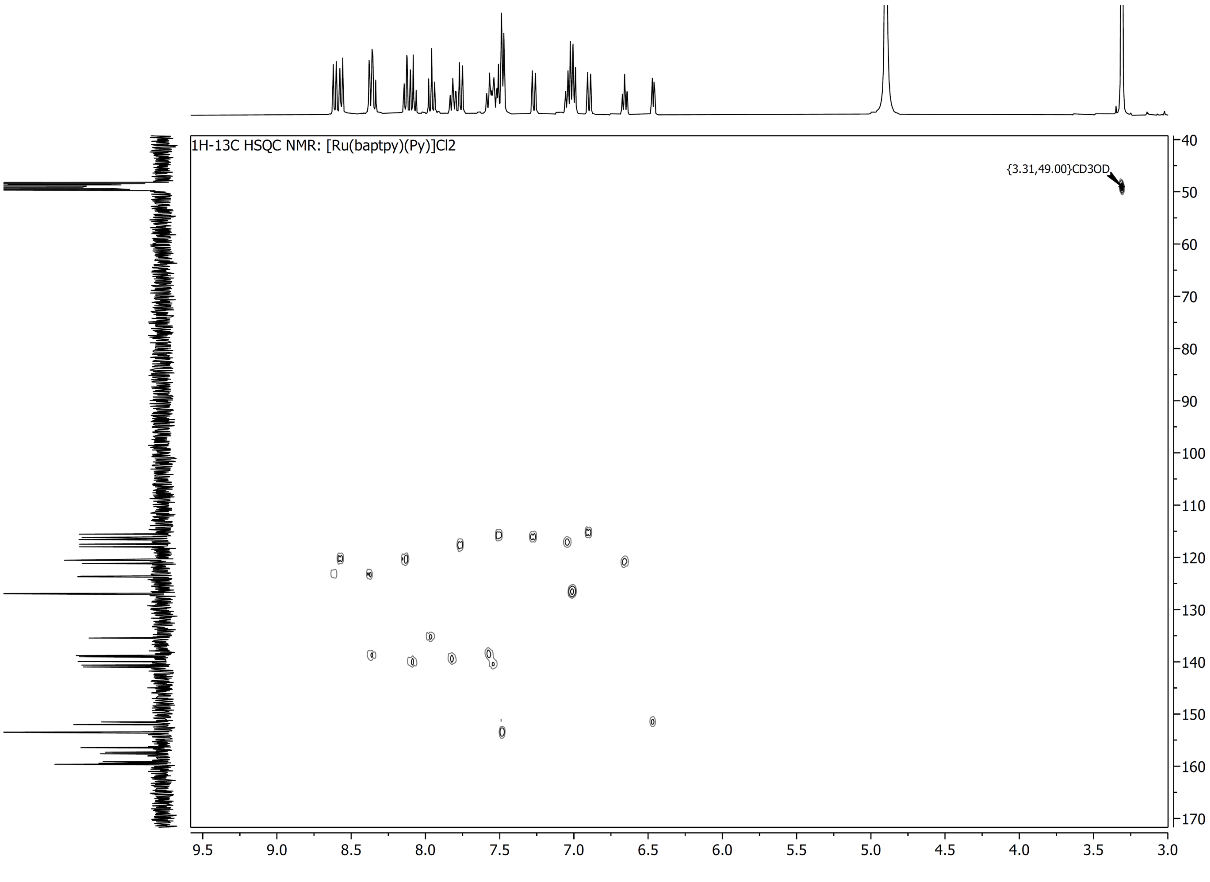

Figure S48. ^{1}H-^{13}C HSQC NMR of [**6**]Cl_2 in CD_3OD.

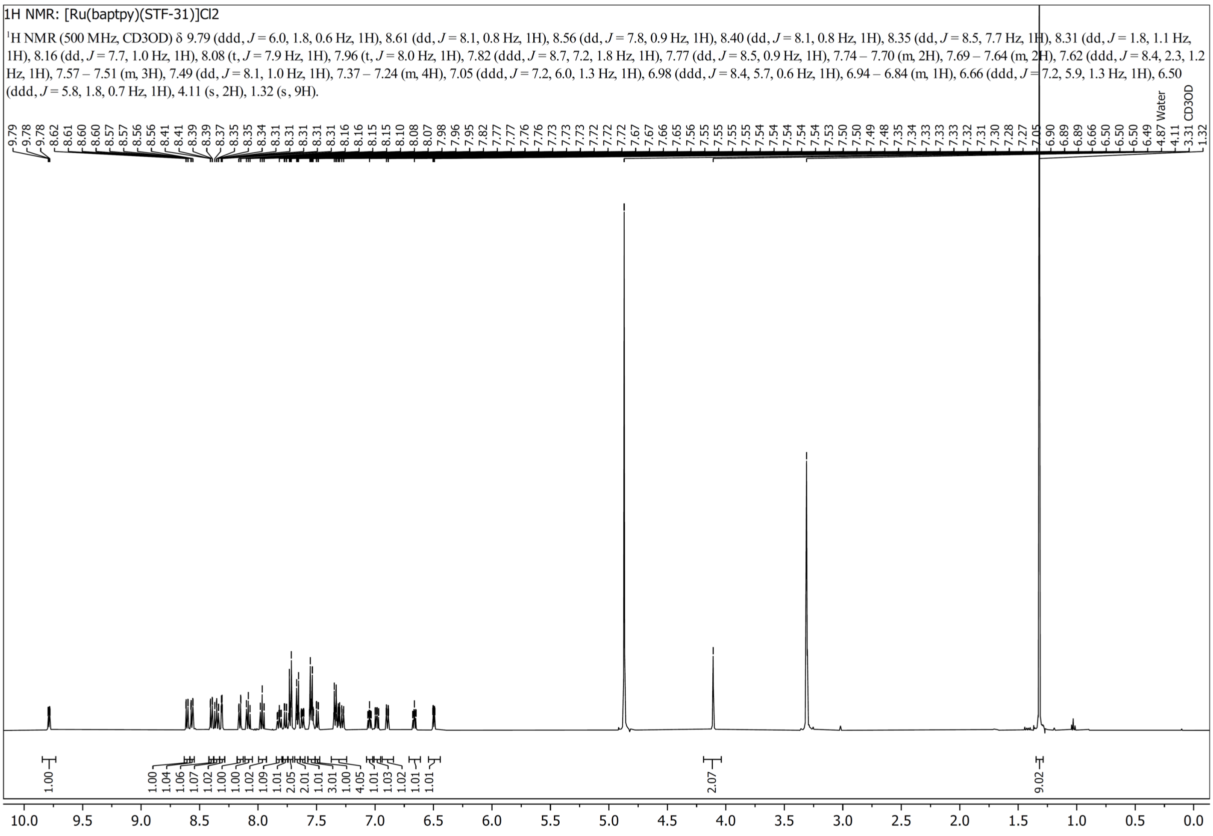

Figure S49. ^{1}H NMR of [**7**]Cl_2 in CD_3OD.

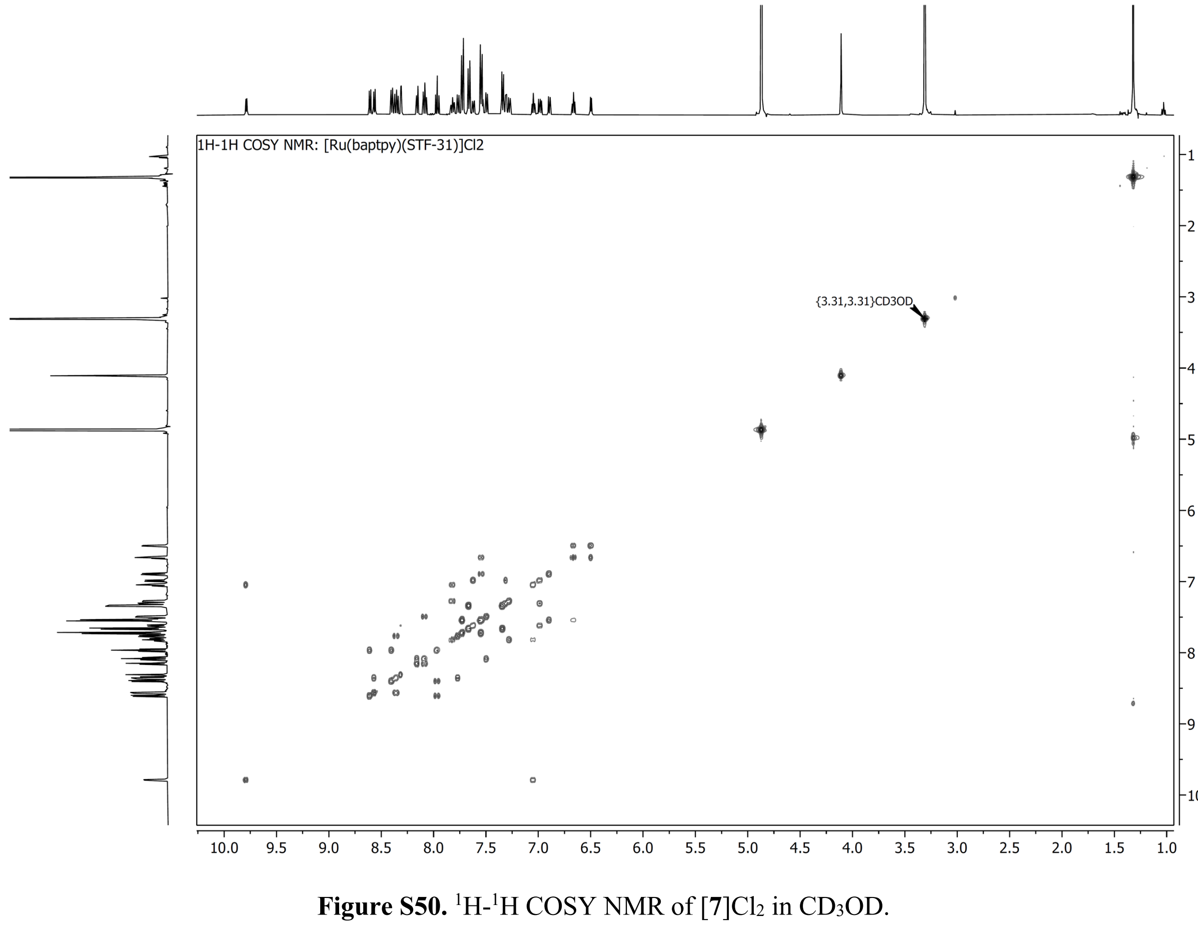

Figure S50. ^{1}H-^{1}H COSY NMR of [**7**]Cl_2 in CD_3OD.

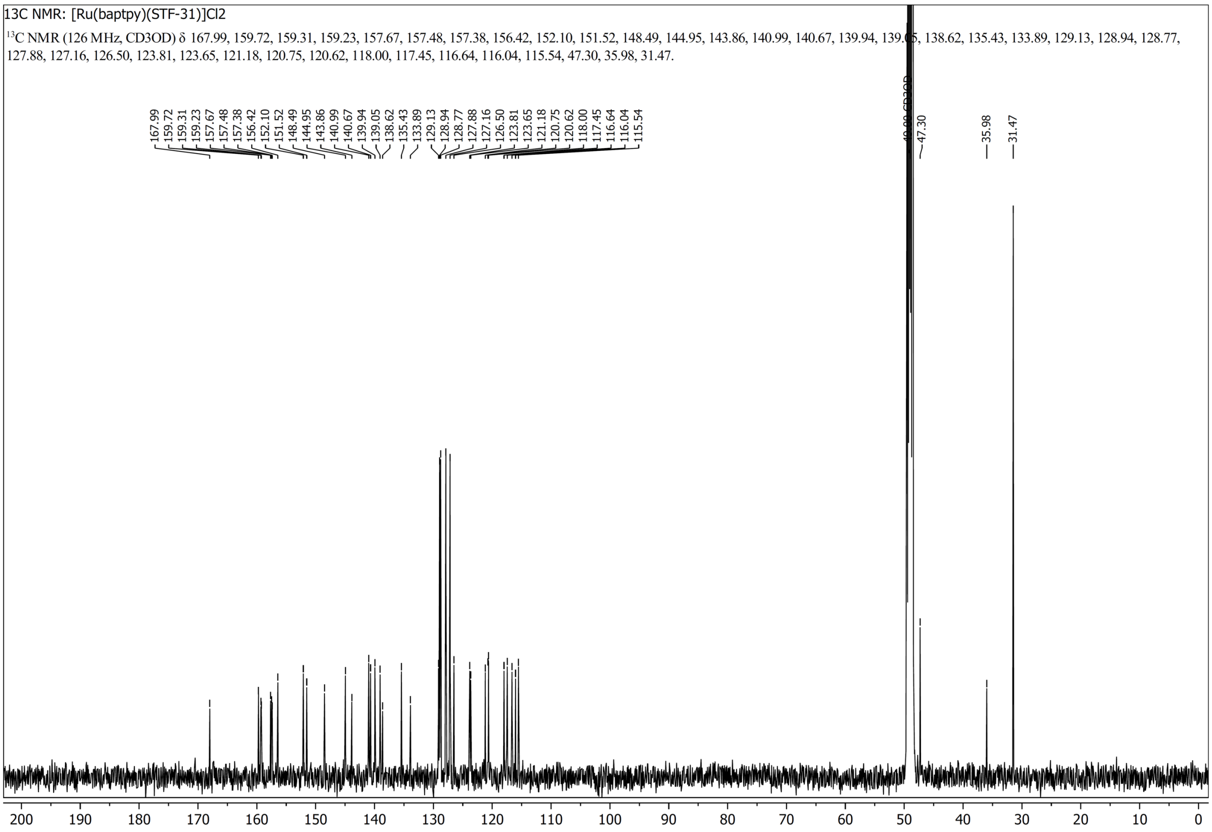

Figure S51. ^{13}C NMR of [**7**]Cl_2 in CD_3OD.

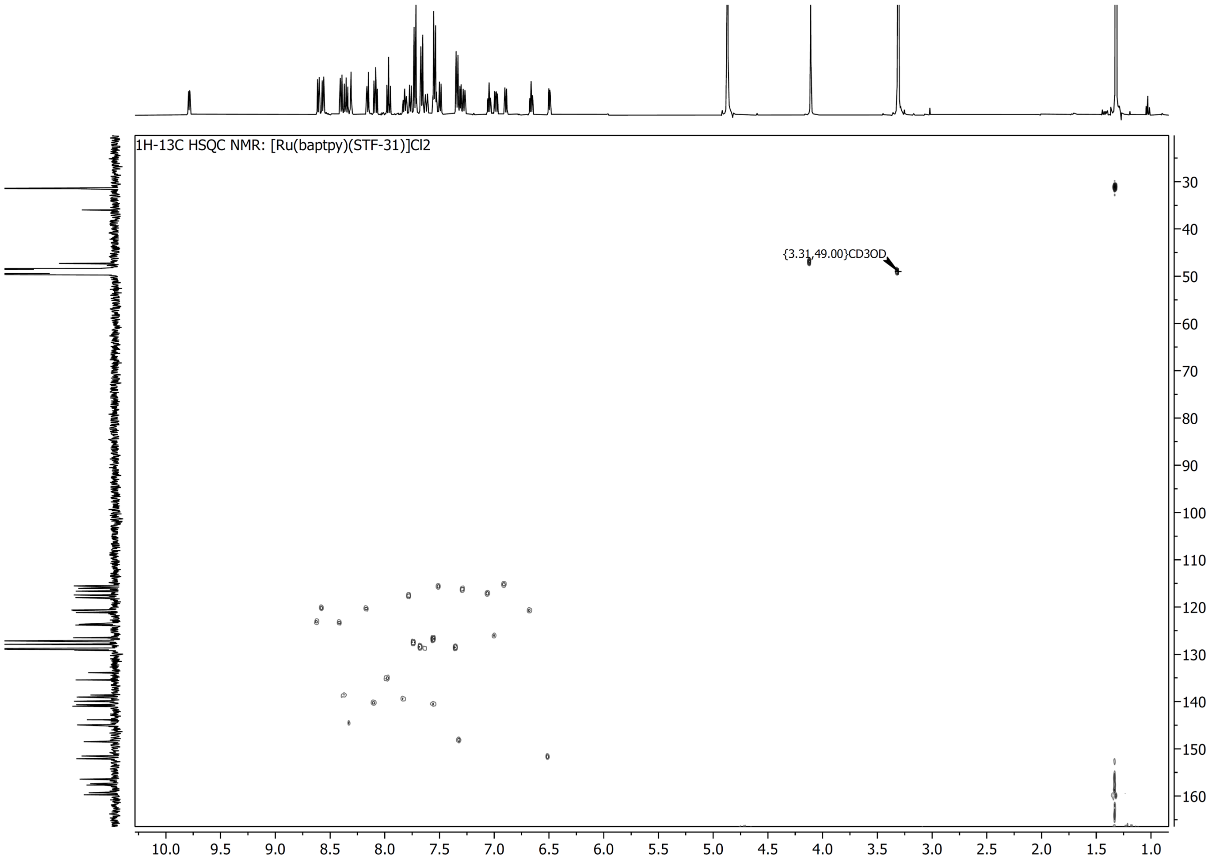

Figure S52. ^{1}H-^{13}C HSQC NMR of [**7**]Cl_2 in CD_3OD.

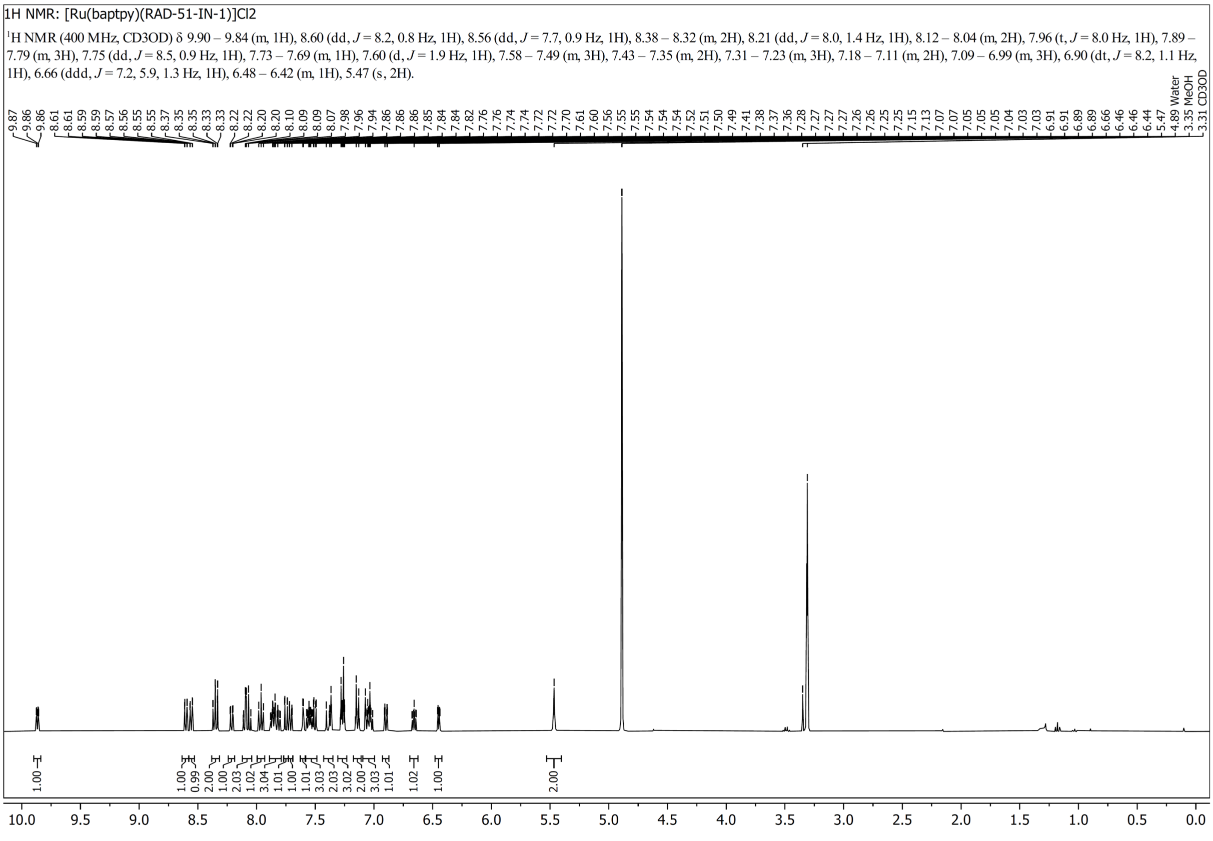

Figure S53. ^{1}H NMR of [**8**]Cl_2 in CD_3OD.

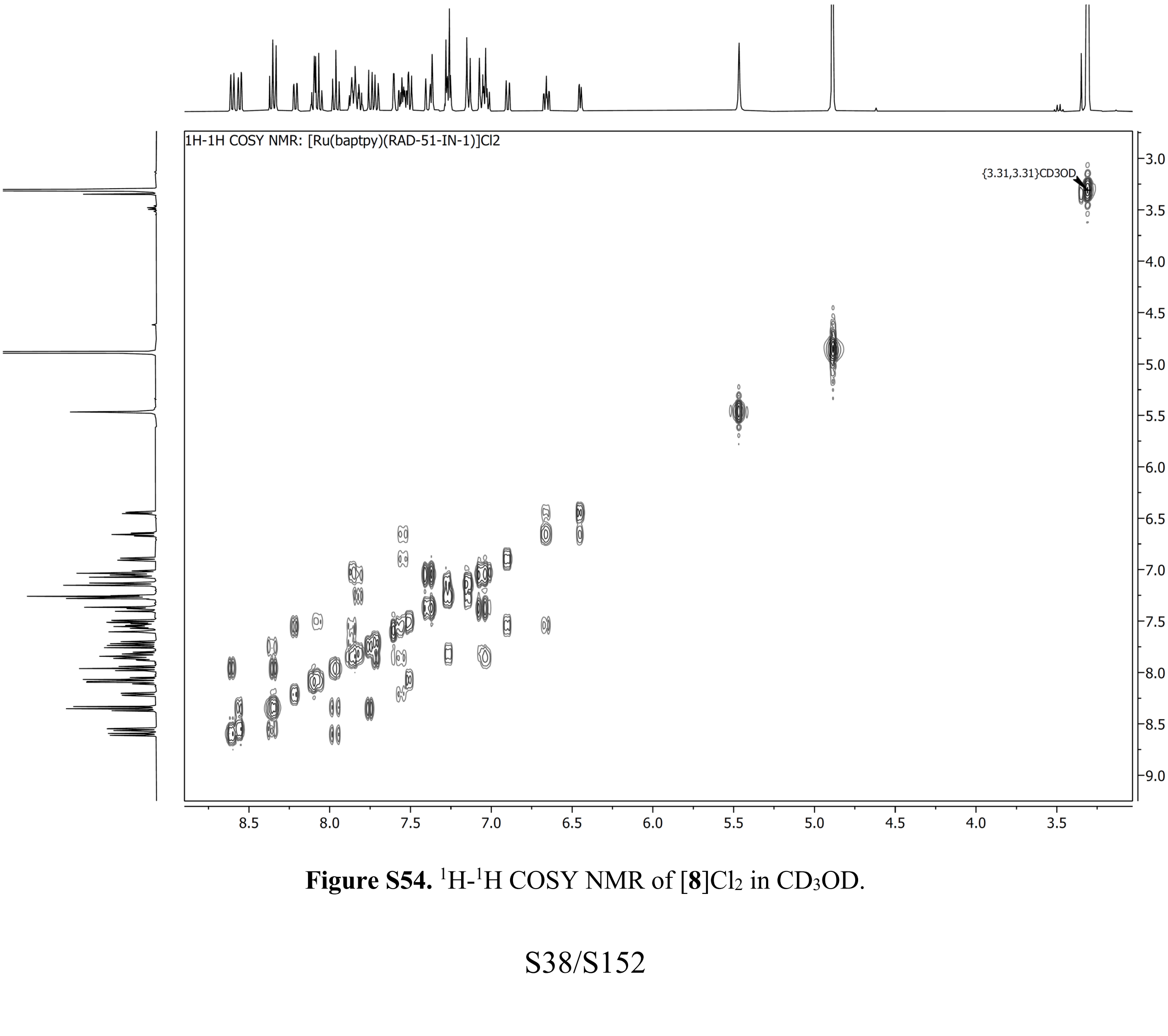

Figure S54. ^{1}H-^{1}H COSY NMR of [**8**]Cl_2 in CD_3OD.

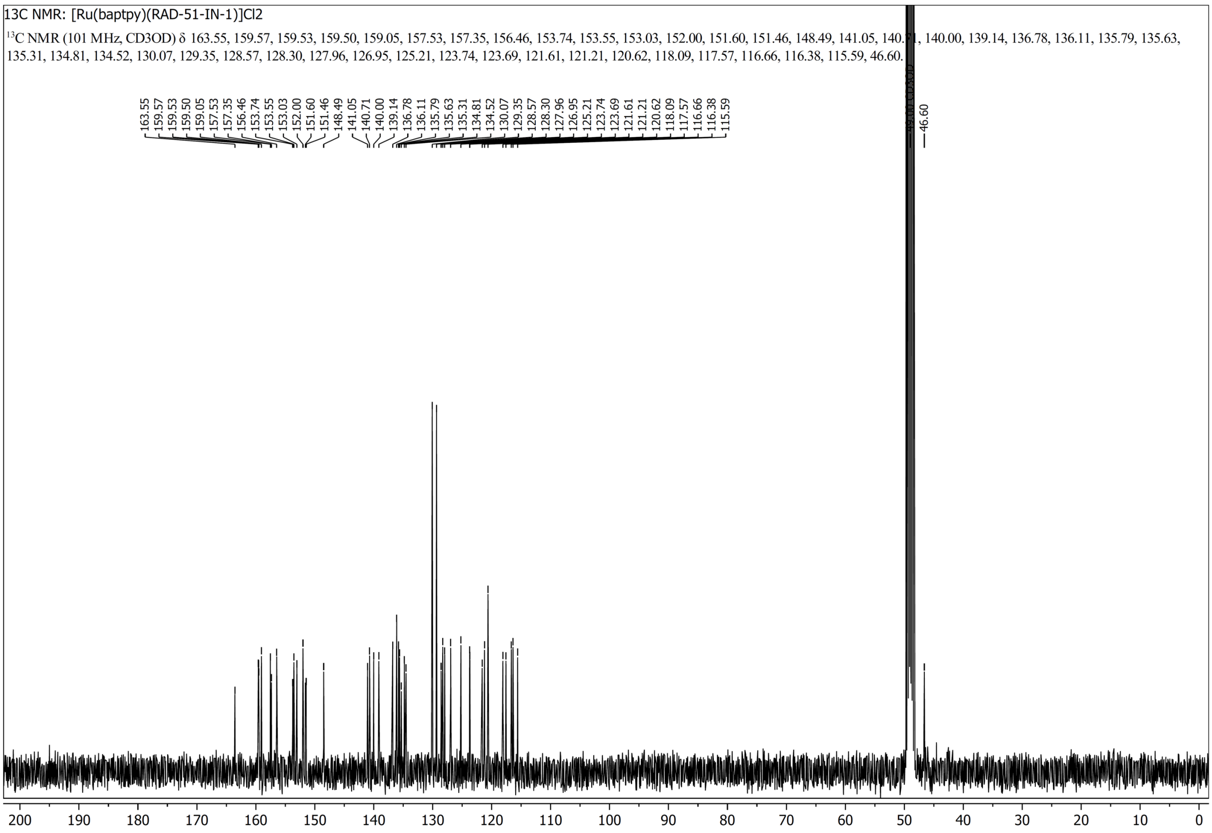

Figure S55. ^{13}C NMR of [**8**]Cl_2 in CD_3OD.

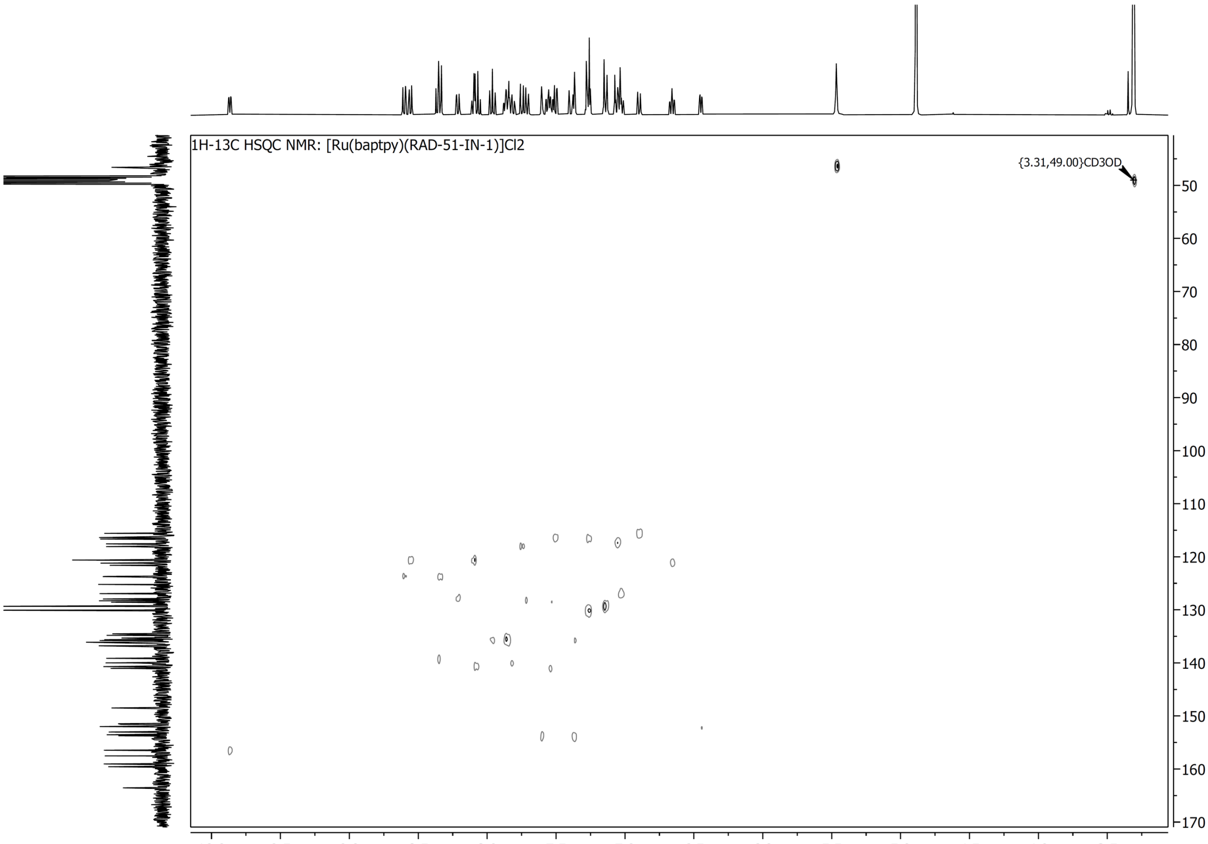

Figure S56. ^{1}H-^{13}C HSQC NMR of [**8**]Cl_2 in CD_3OD.

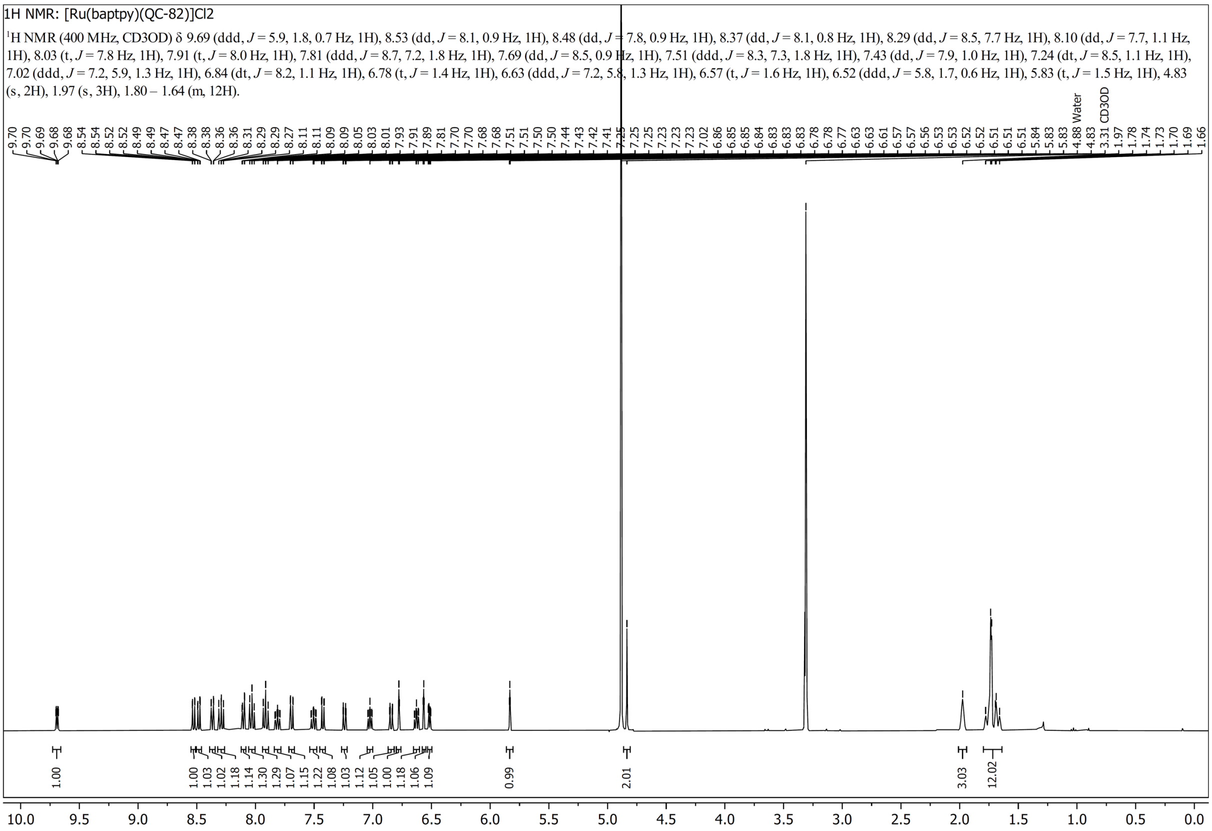

Figure S57. ^{1}H NMR of [**9**]Cl_2 in CD_3OD.

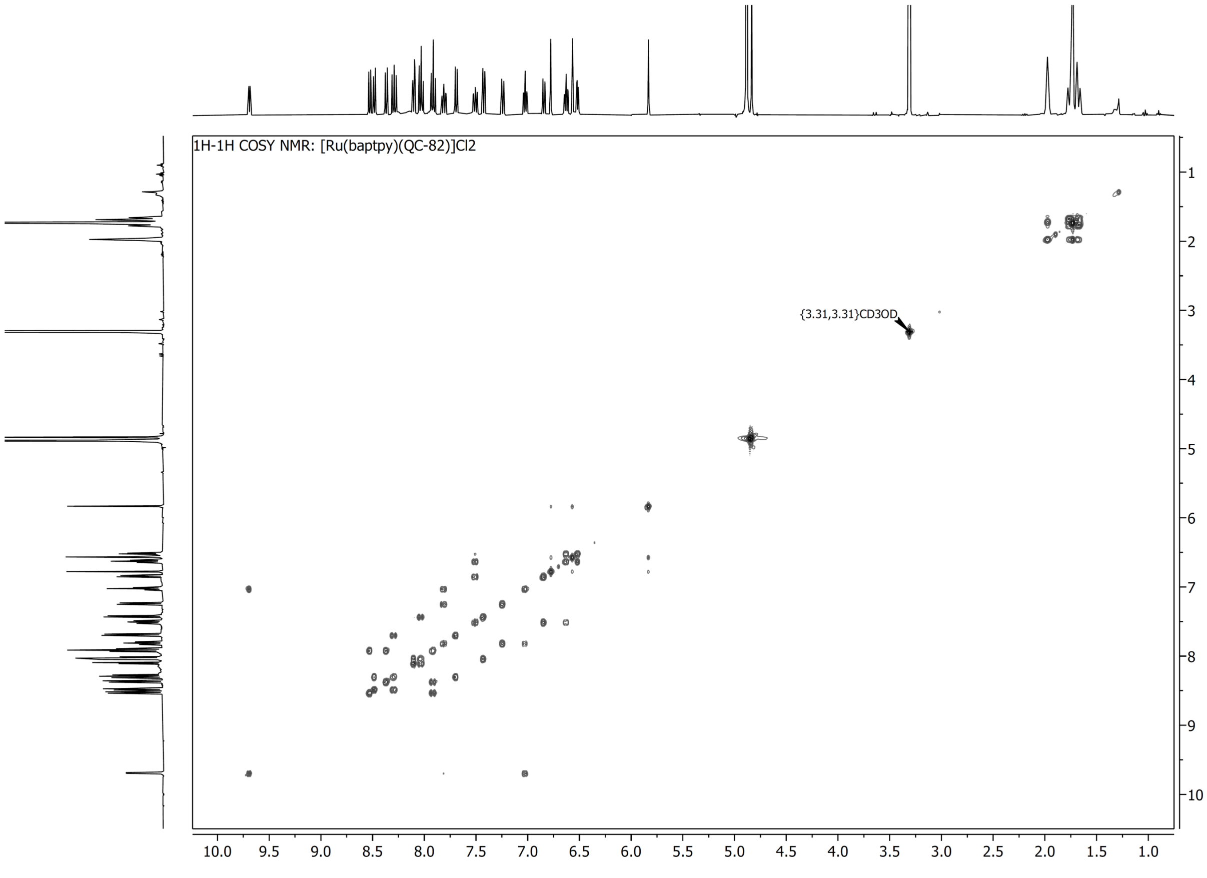

Figure S58. ^{1}H-^{1}H COSY NMR of [**9**]Cl_2 in CD_3OD.

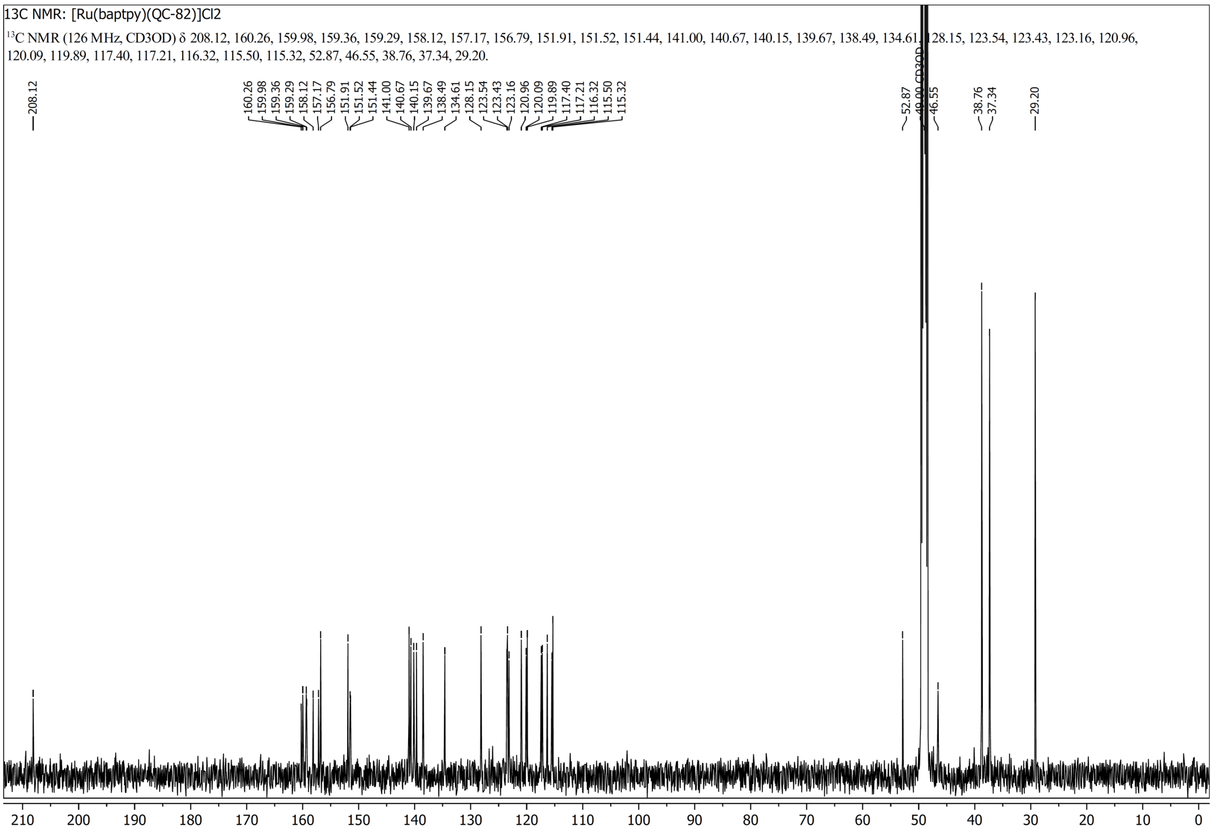

Figure S59. ^{13}C NMR of [**9**]Cl_2 in CD_3OD.

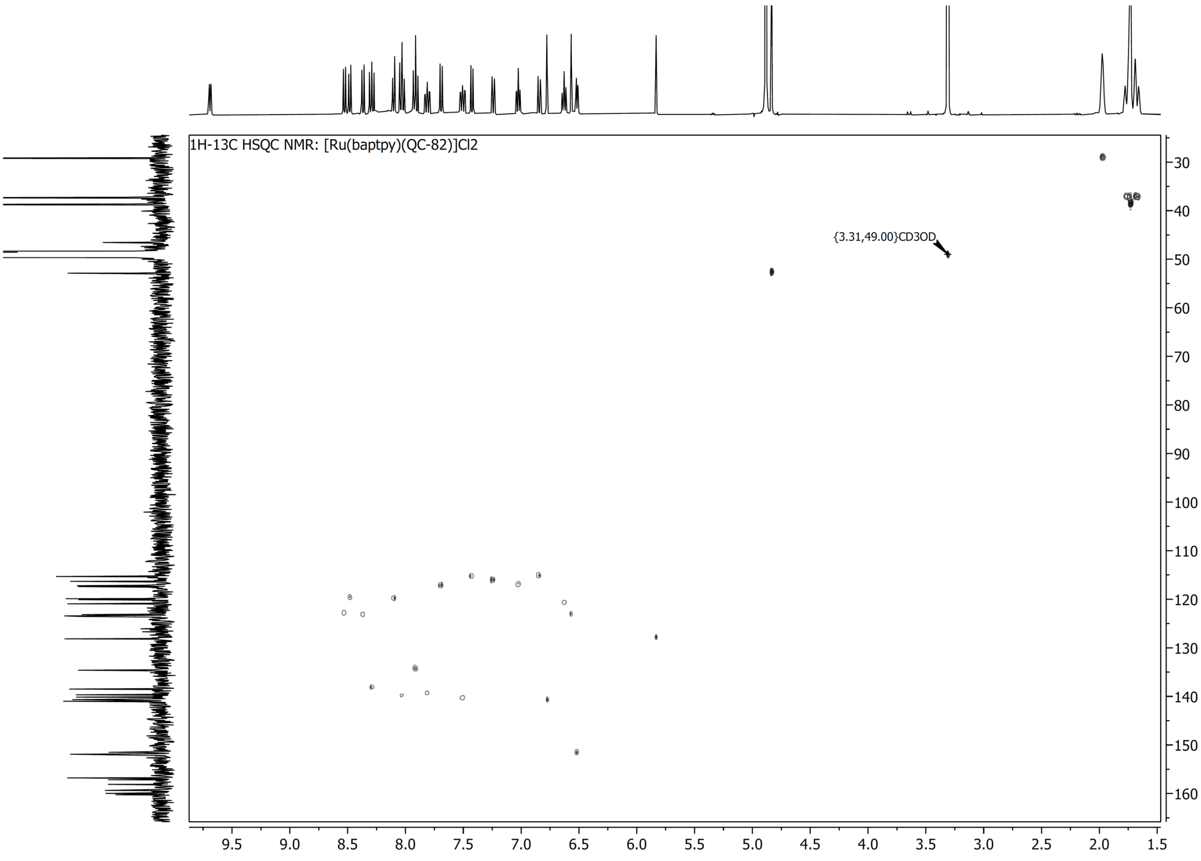

Figure S60. ^{1}H-^{13}C HSQC NMR of [**9**]Cl_2 in CD_3OD.

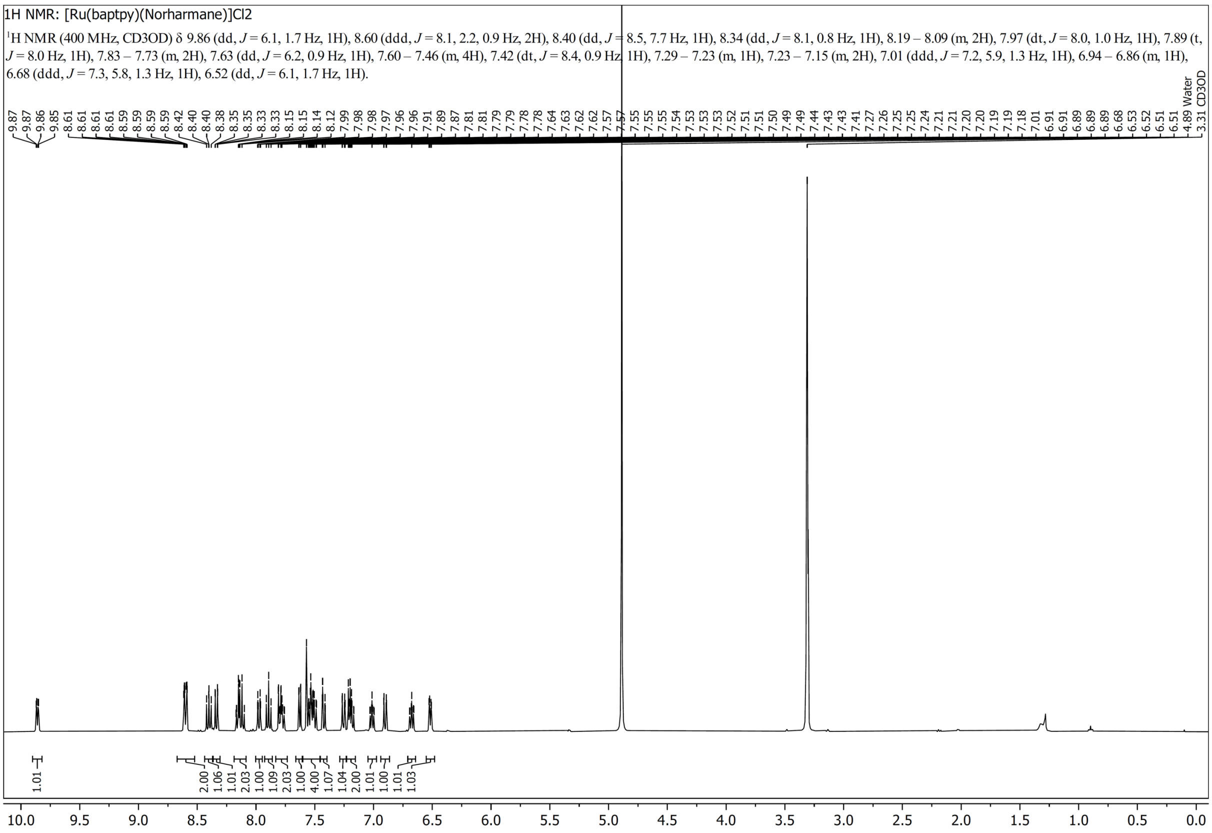

Figure S61. ^{1}H NMR of [**10**]Cl_2 in CD_3OD.

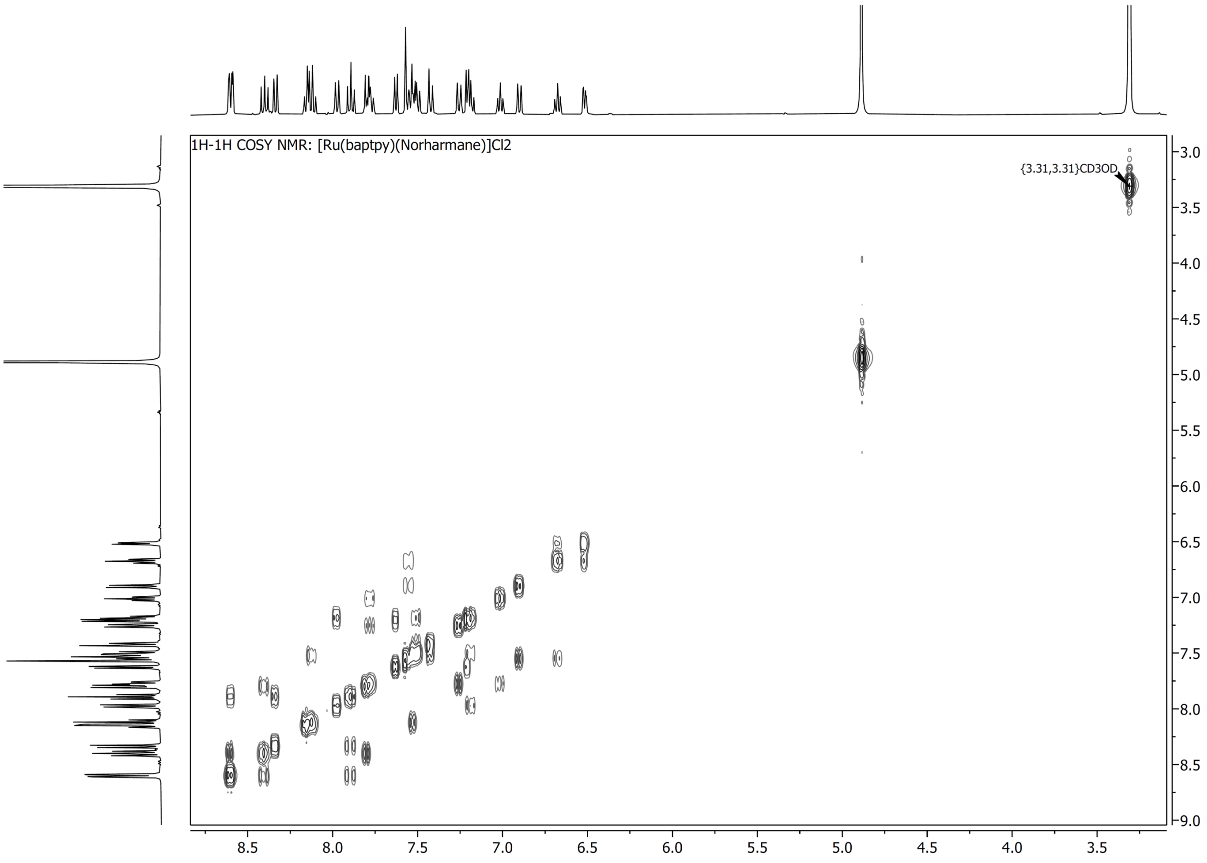

Figure S62. ^{1}H-^{1}H COSY NMR of [**10**]Cl_2 in CD_3OD.

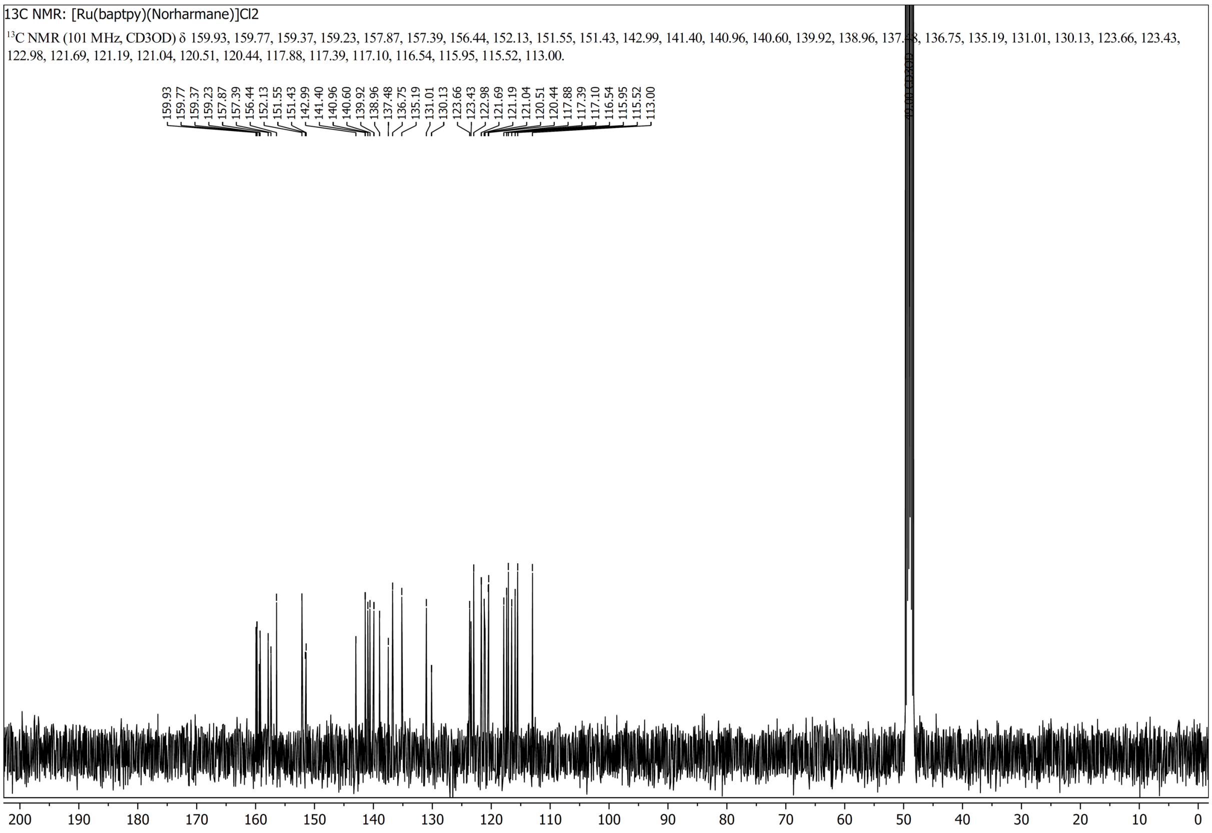

Figure S63. ^{13}C NMR of [**10**]Cl_2 in CD_3OD.

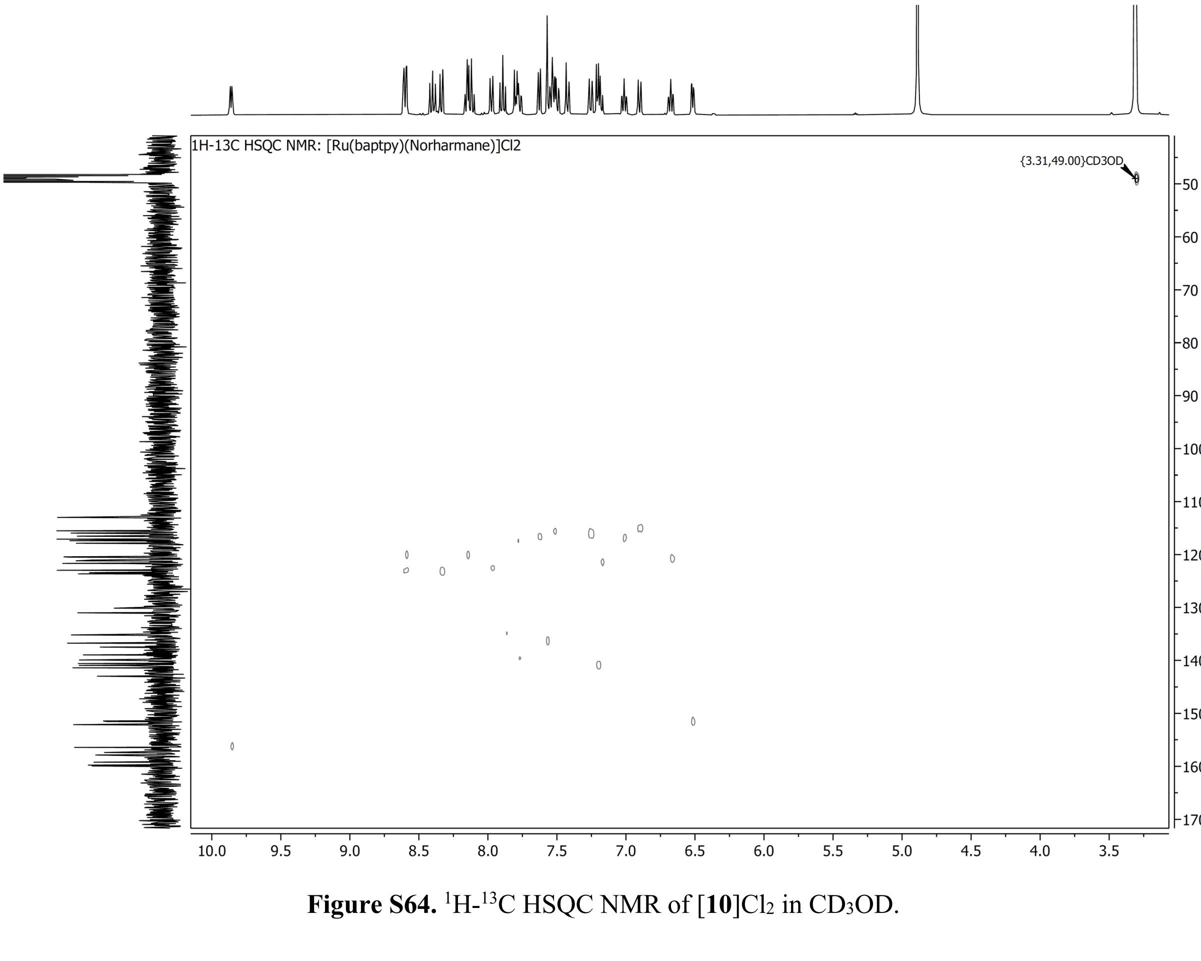

Figure S64. ^{1}H-^{13}C HSQC NMR of [**10**]Cl_2 in CD_3OD.

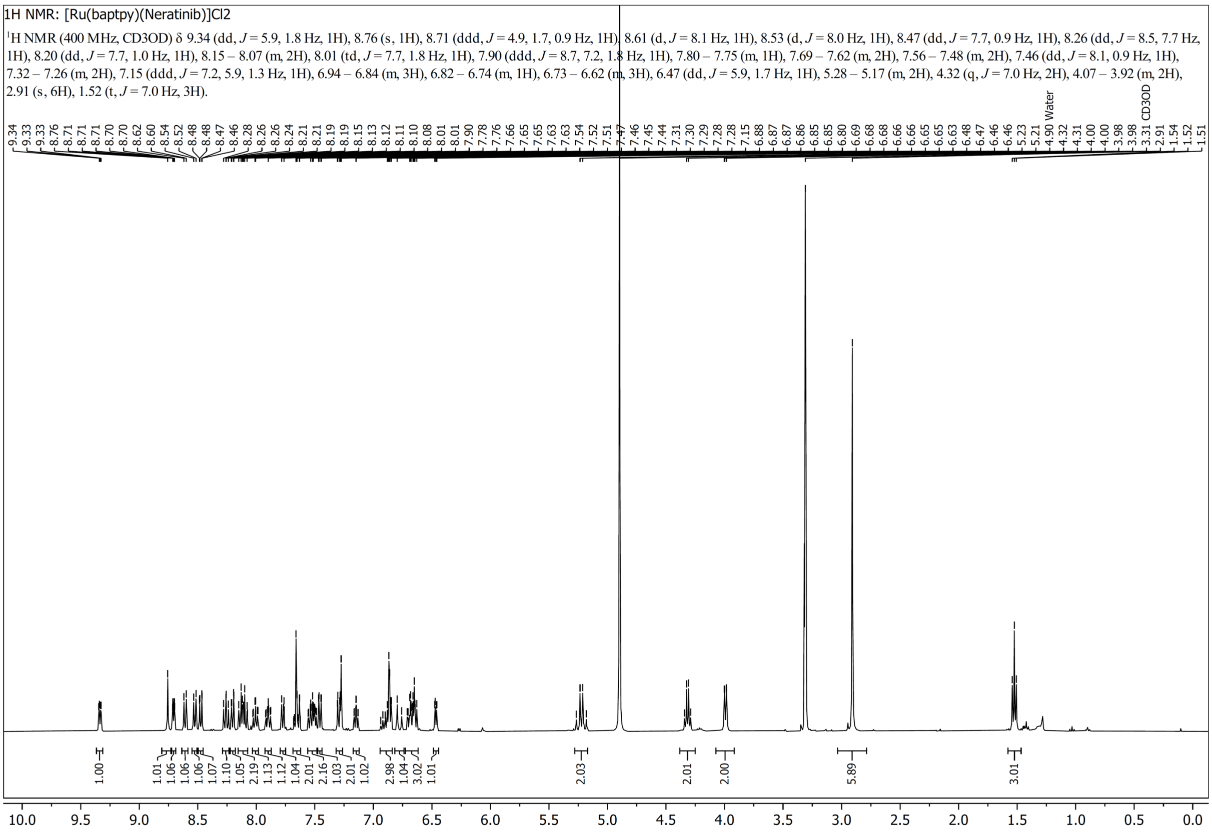

Figure S65. ^{1}H NMR of [**11**]Cl_2 in CD_3OD.

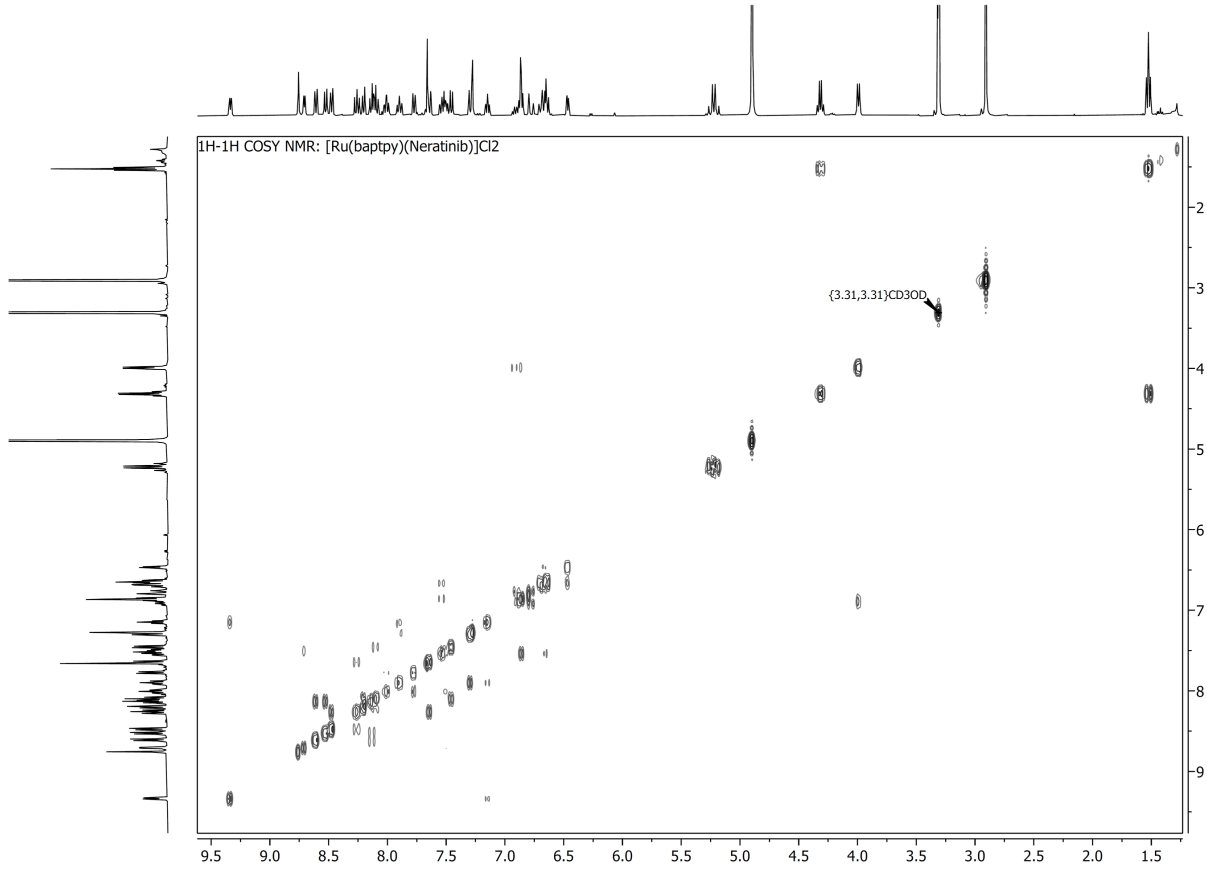

Figure S66. ^{1}H-^{1}H COSY NMR of [**11**]Cl_2 in CD_3OD.

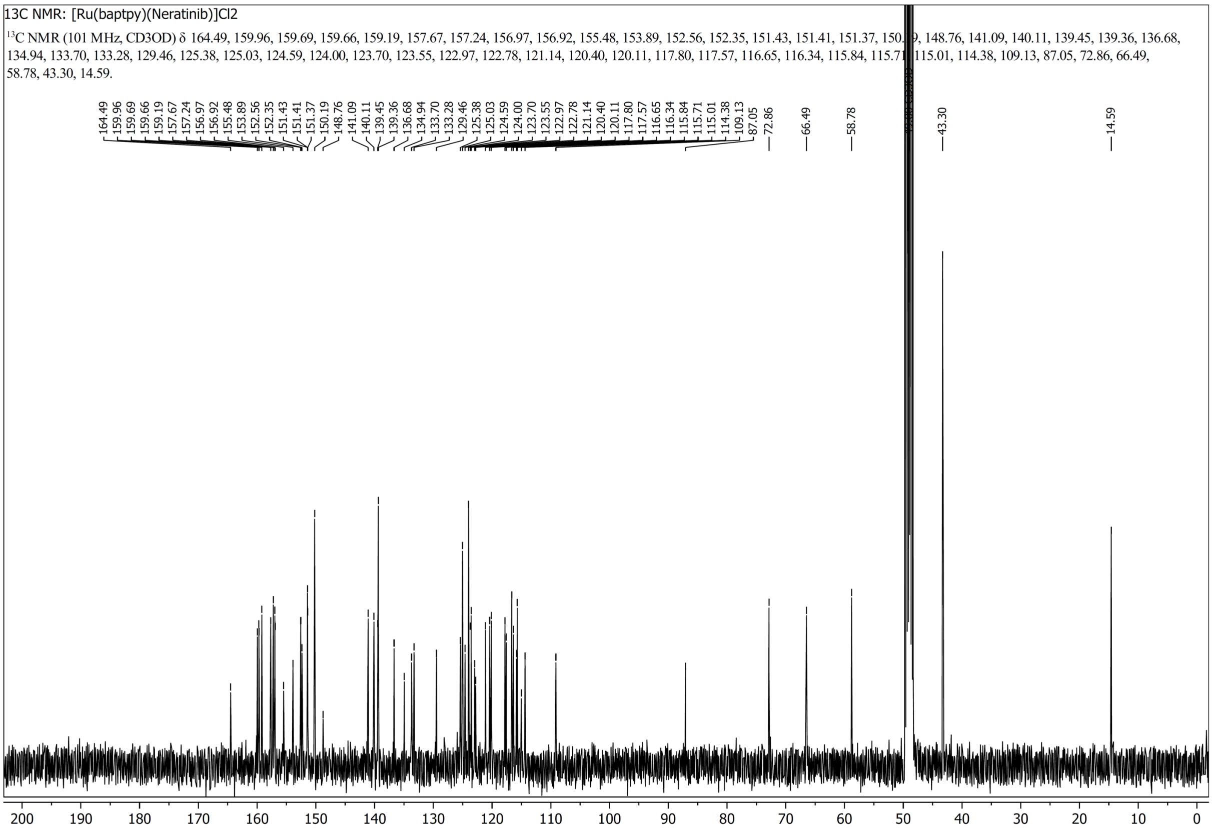

Figure S67. ^{13}C NMR of [**11**]Cl_2 in CD_3OD.

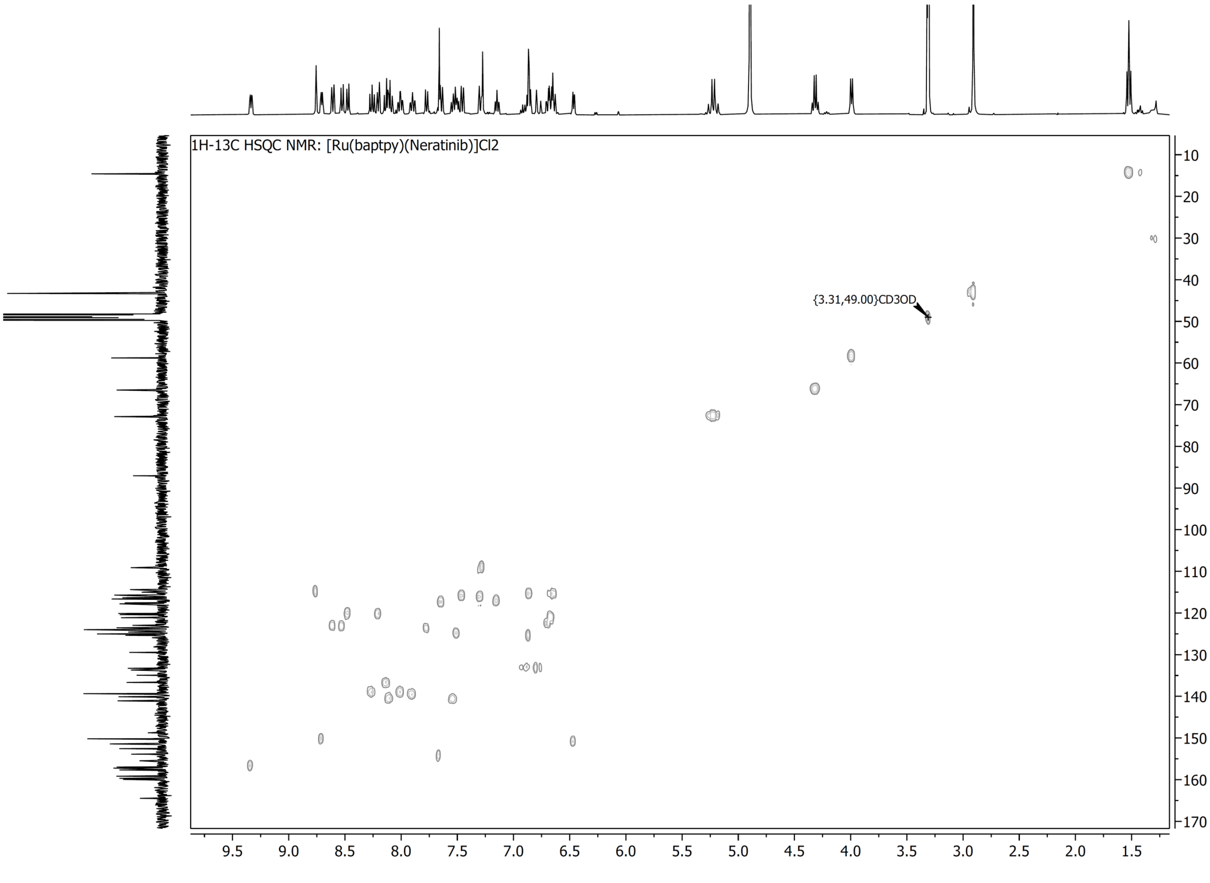

Figure S68. ^{1}H-^{13}C HSQC NMR of [**11**]Cl_2 in CD_3OD.

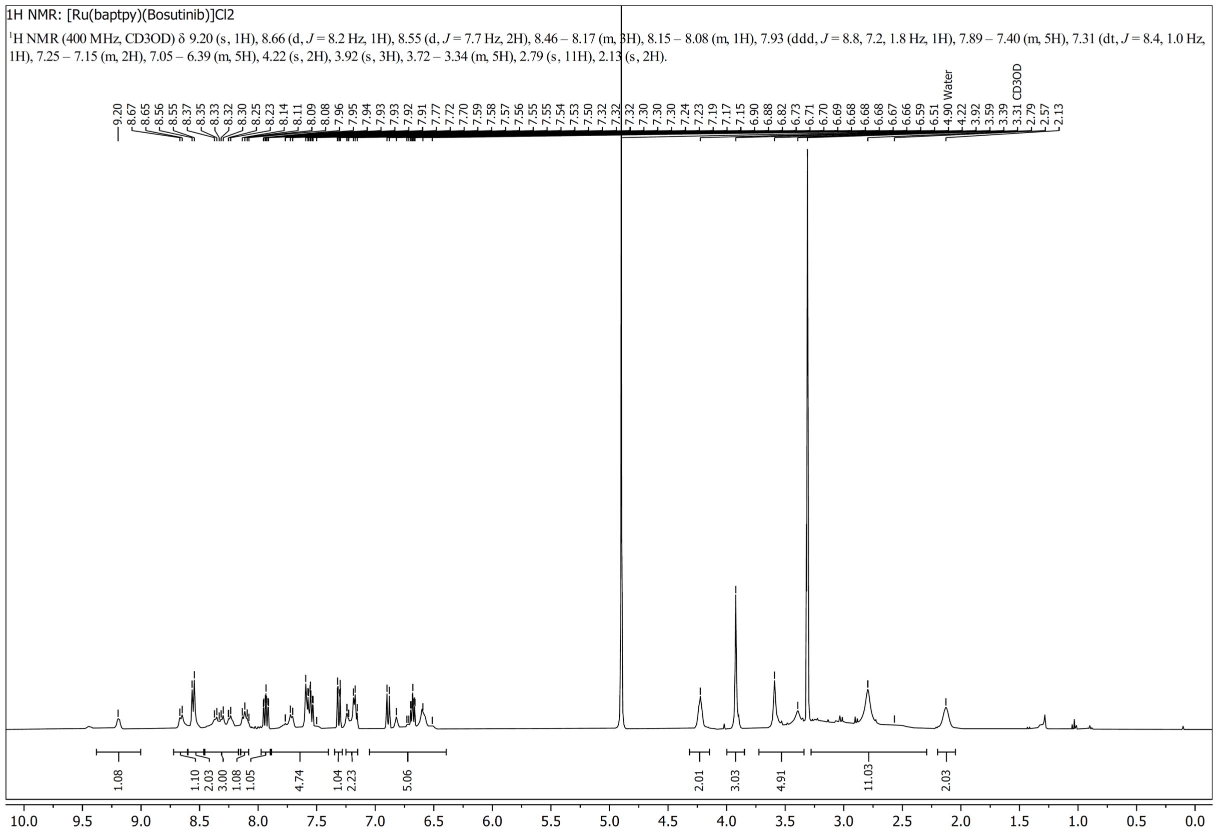

Figure S69. ^{1}H NMR of [**12**]Cl_2 in CD_3OD.

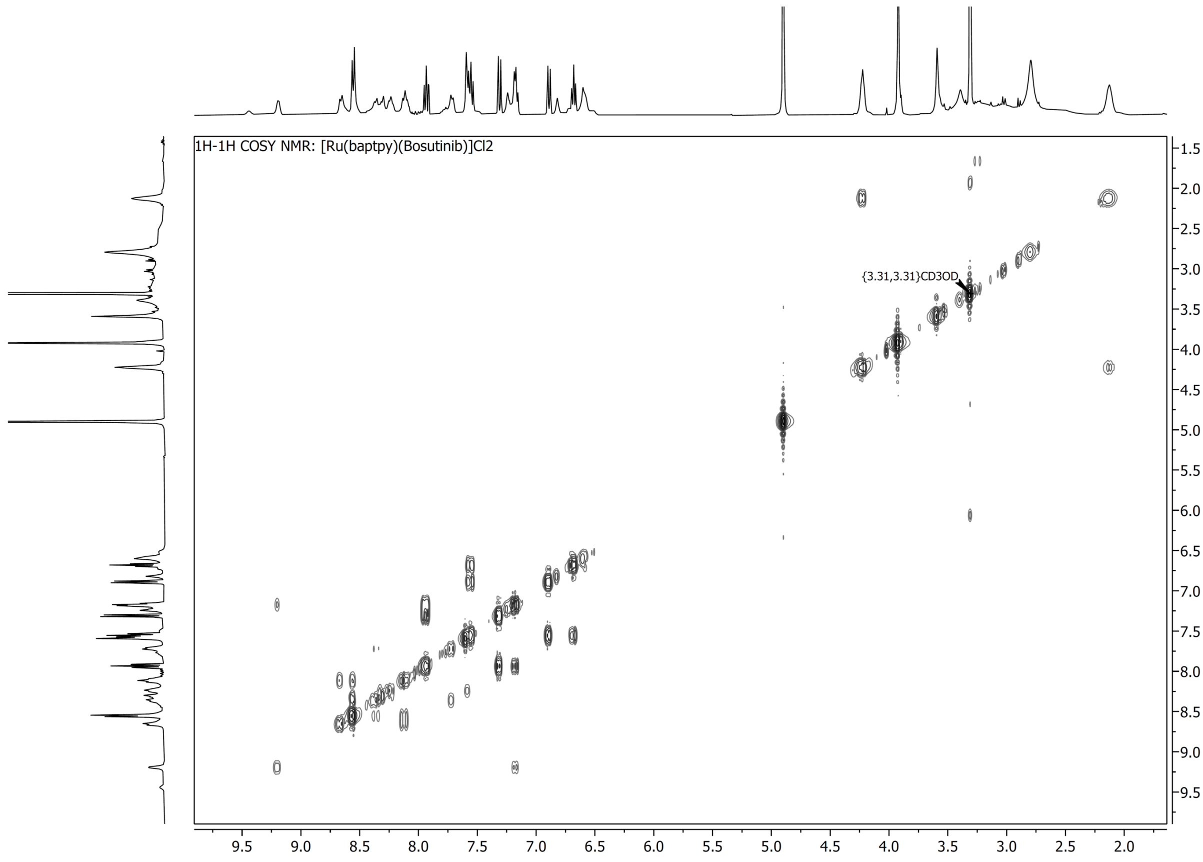

Figure S70. ^{1}H-^{1}H COSY NMR of [**12**]Cl_2 in CD_3OD.

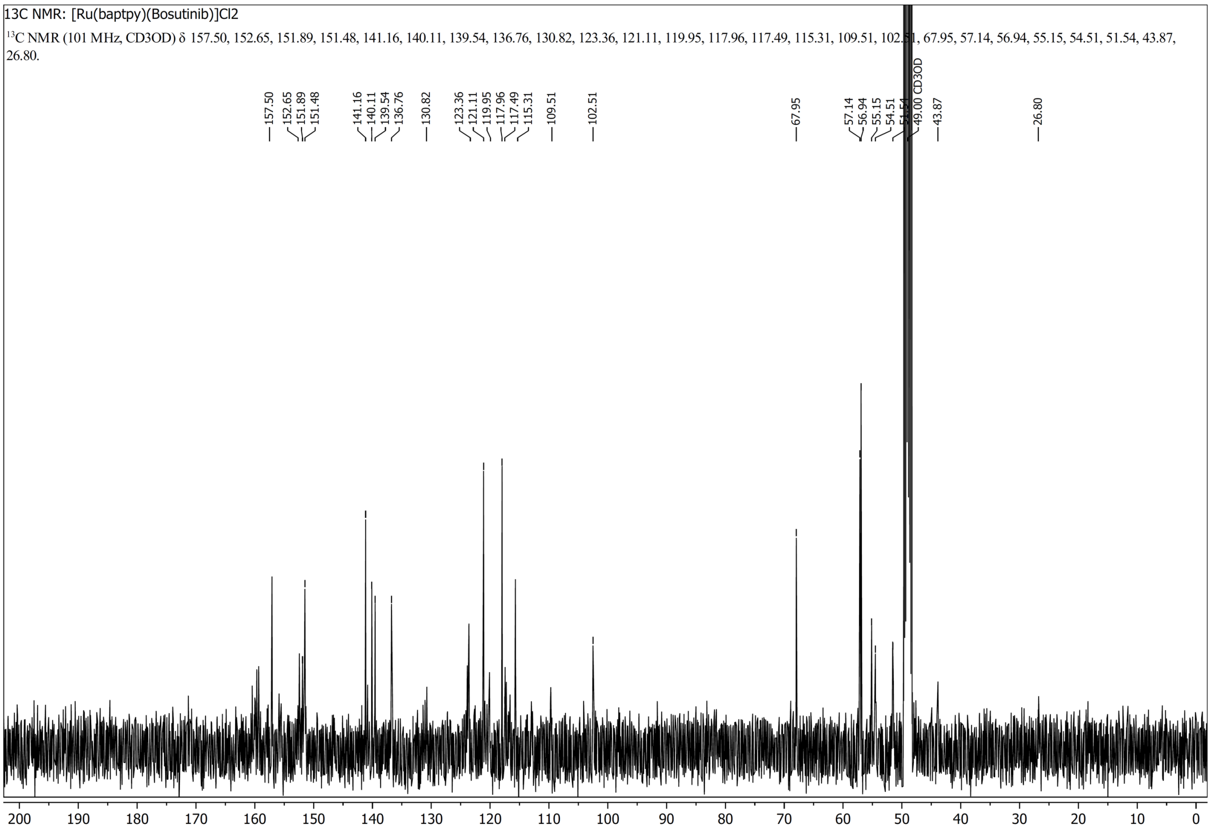

Figure S71. ^{13}C NMR of [**12**]Cl_2 in CD_3OD.

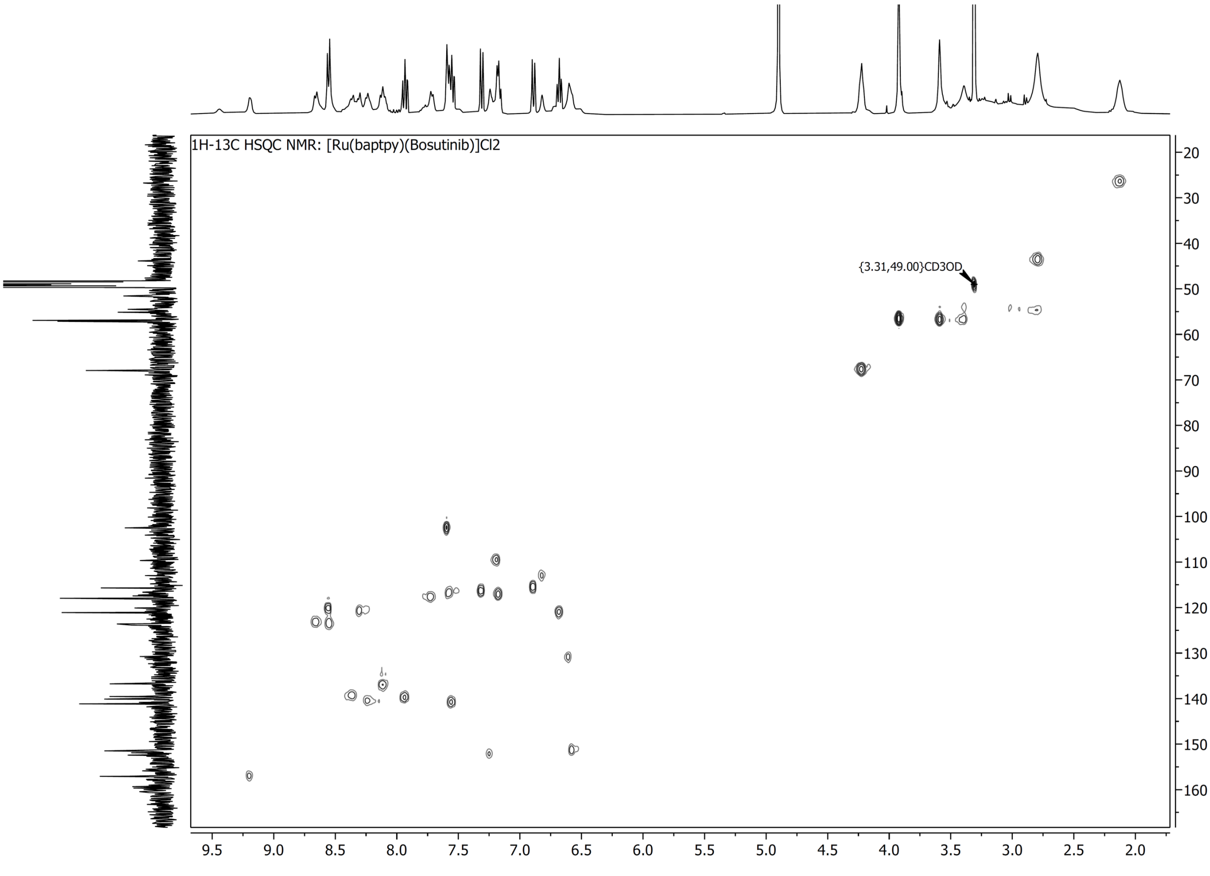

Figure S72. ^{1}H-^{13}C HSQC NMR of [**12**]Cl_2 in CD_3OD.

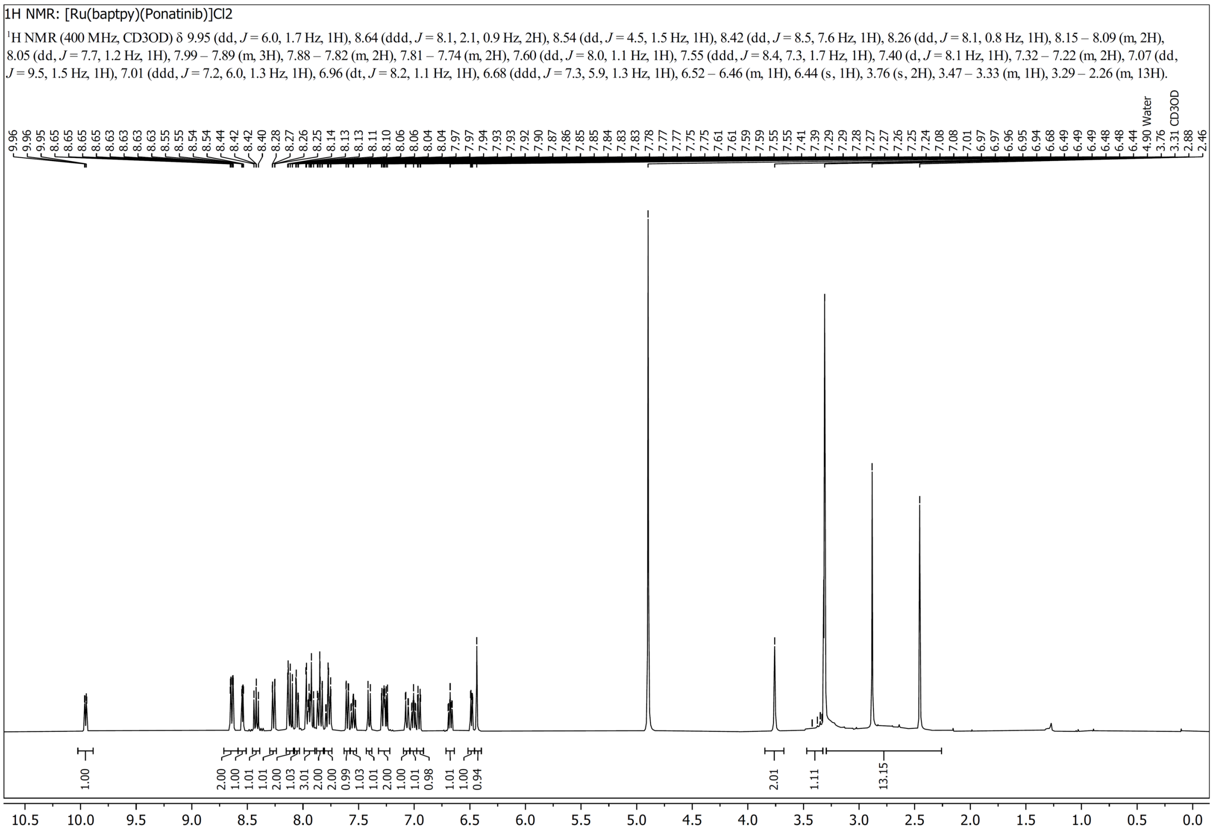

Figure S73. ^{1}H NMR of [**13**]Cl_2 in CD_3OD.

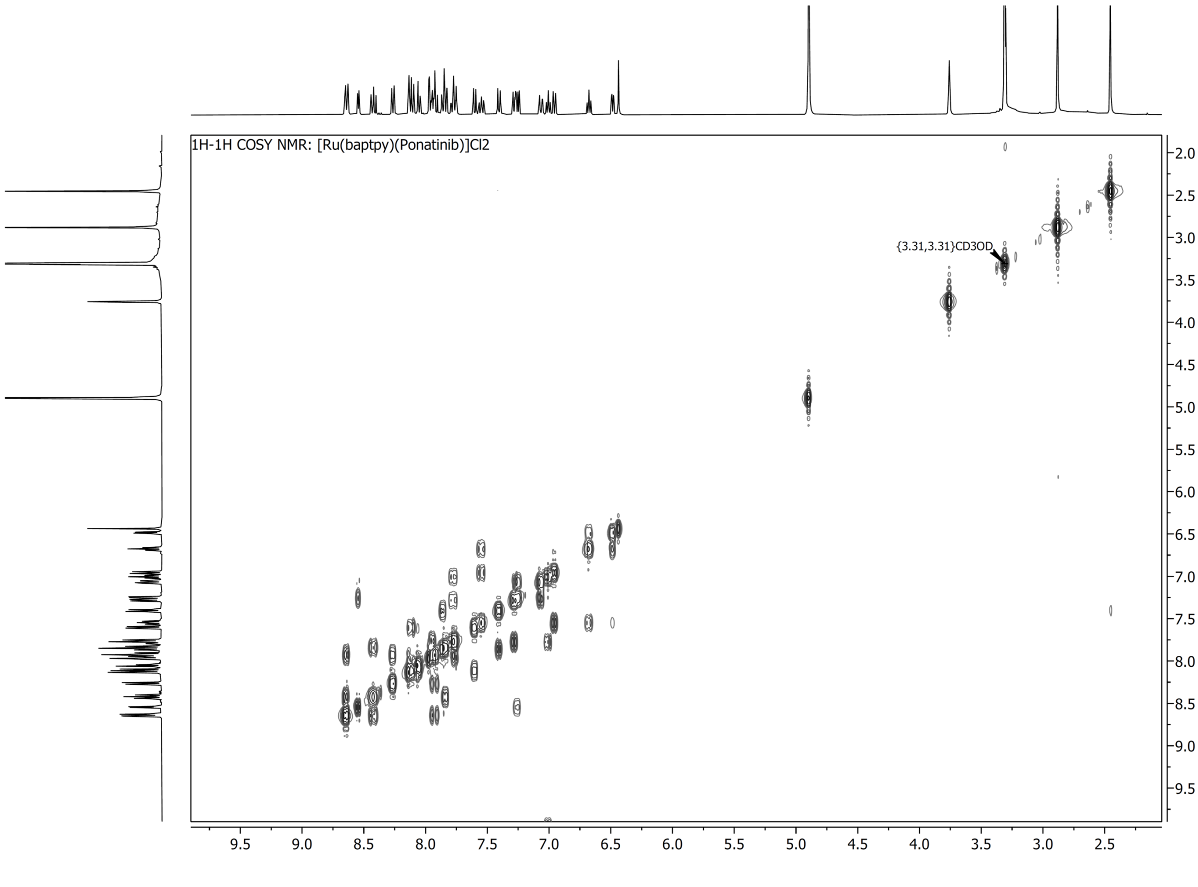

Figure S74. ^{1}H-^{1}H COSY NMR of [**13**]Cl_2 in CD_3OD.

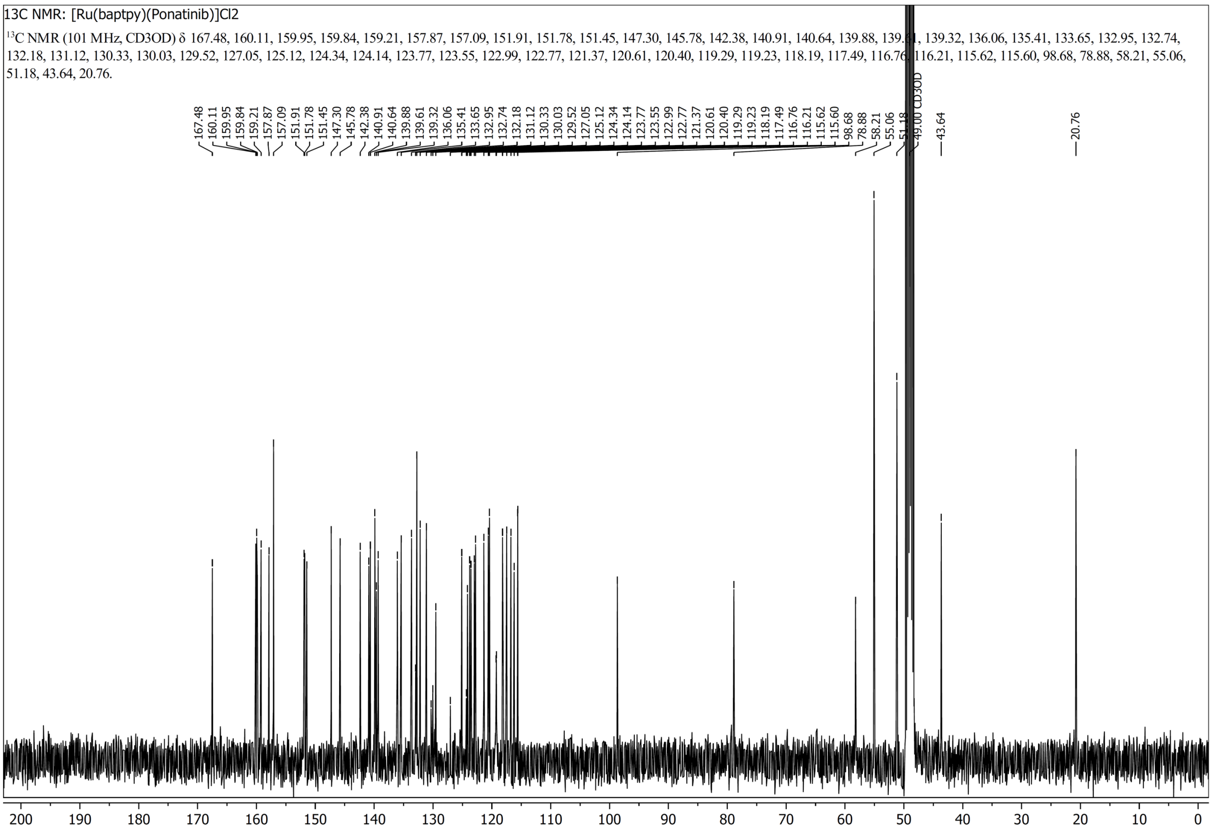

Figure S75. ^{13}C NMR of [**13**]Cl_2 in CD_3OD.

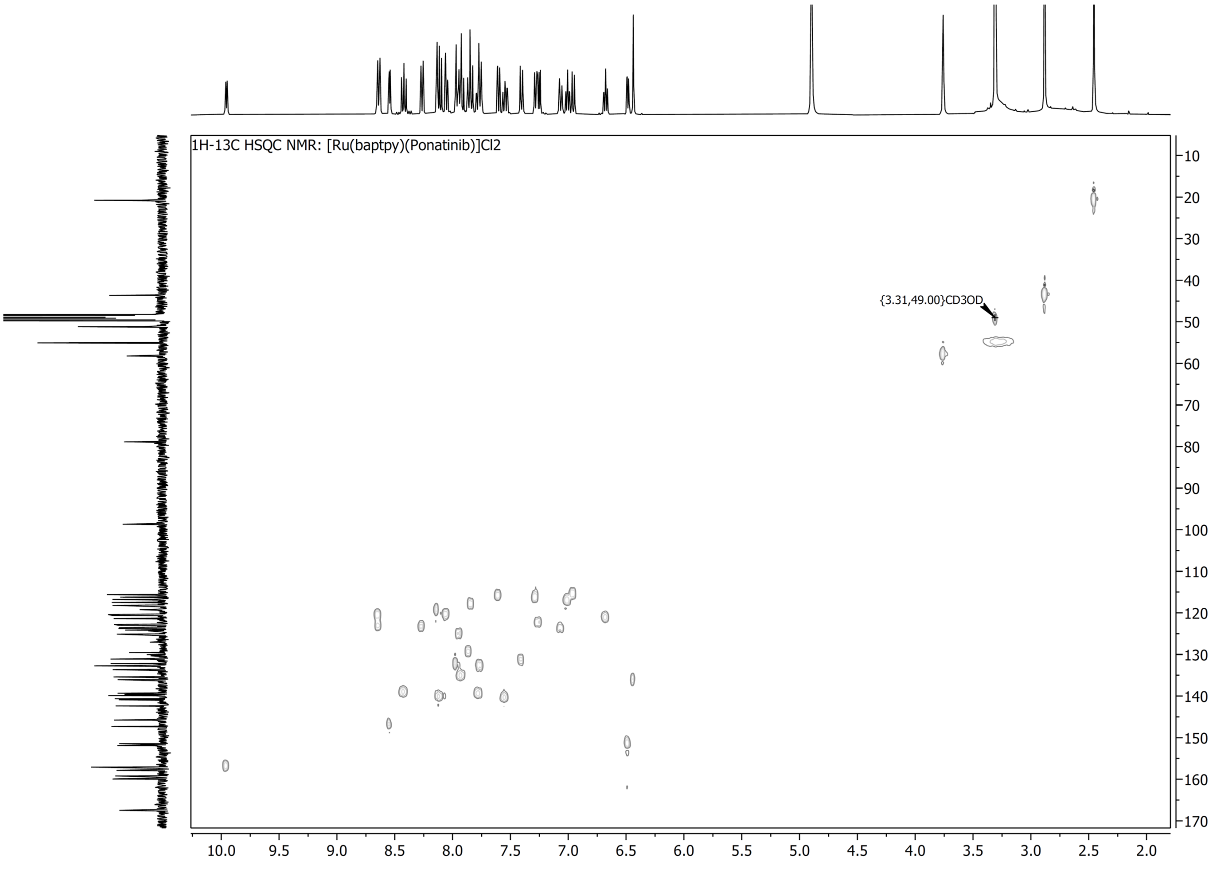

Figure S76. ^{1}H-^{13}C HSQC NMR of [**13**]Cl_2 in CD_3OD.

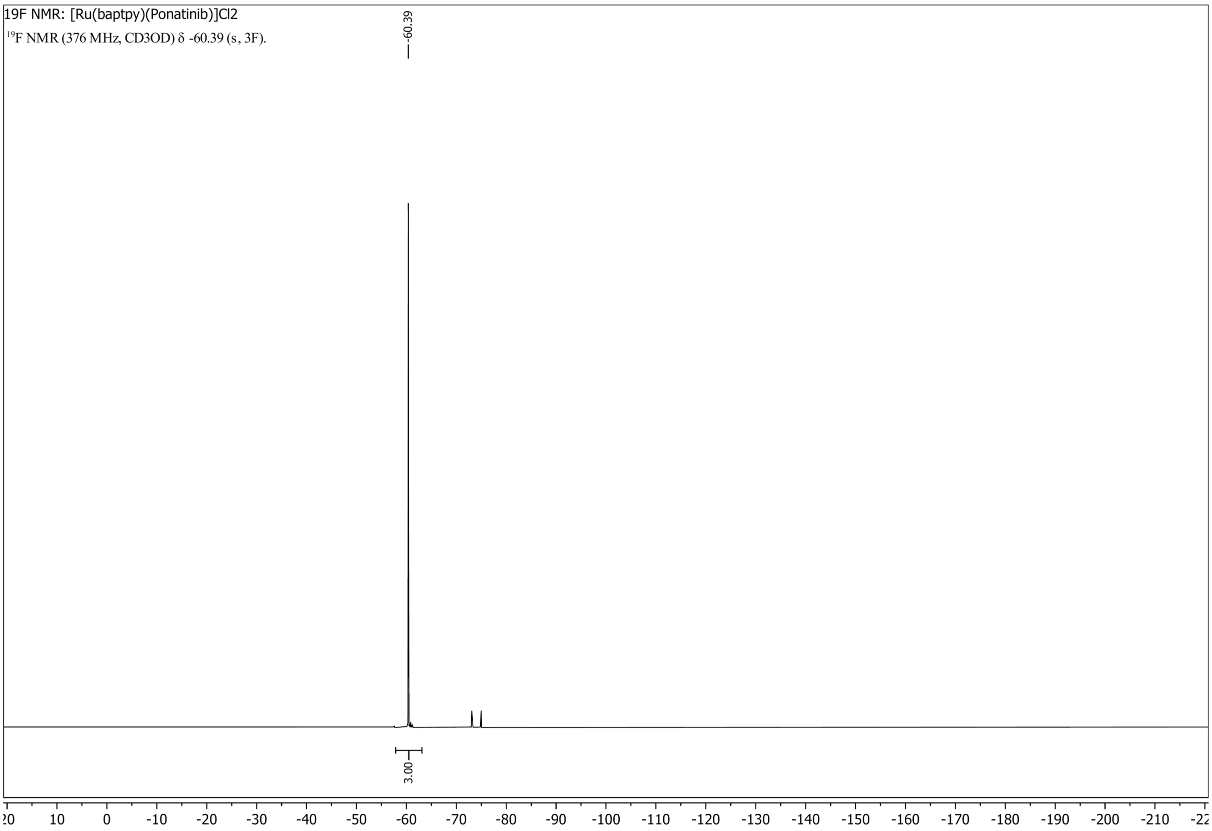

Figure S77. ^{19}F NMR of [**13**]Cl_2 in CD_3OD.

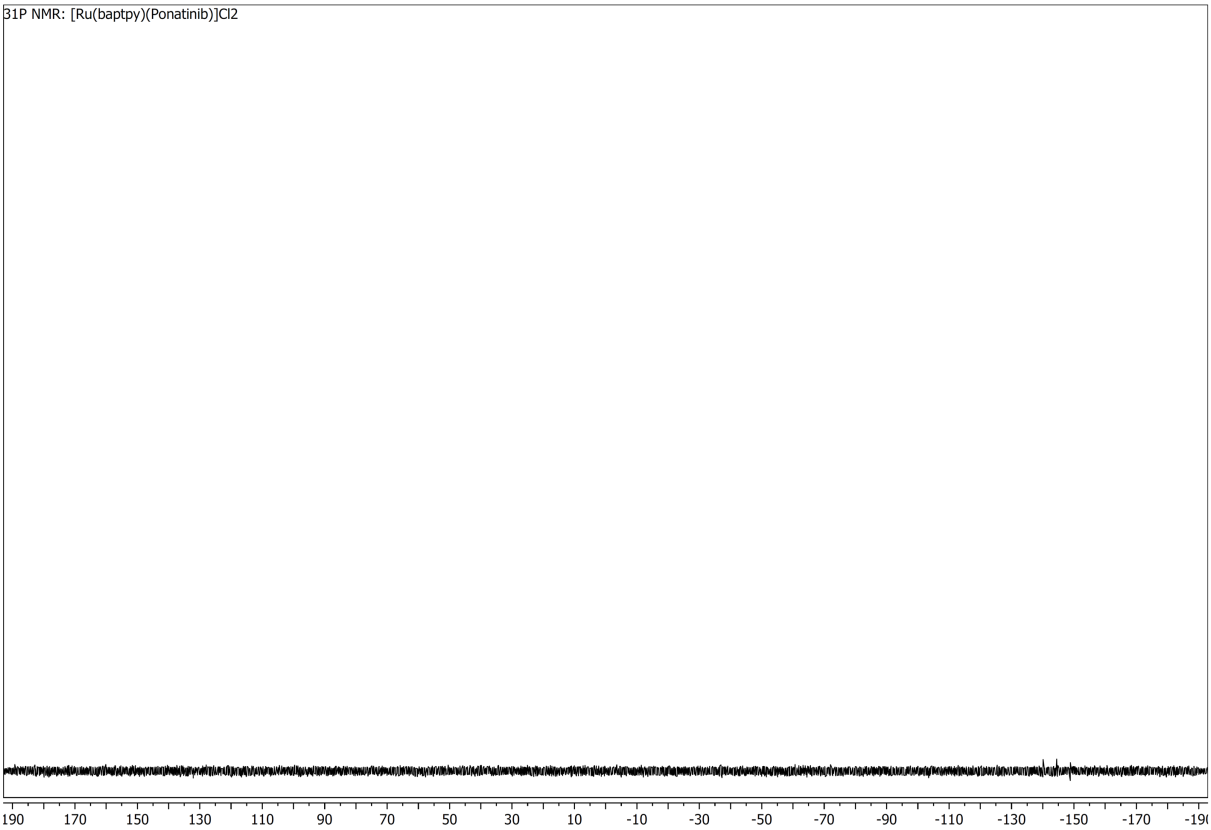

Figure S78. ^{31}P NMR of [**13**]Cl_2 in CD_3OD.

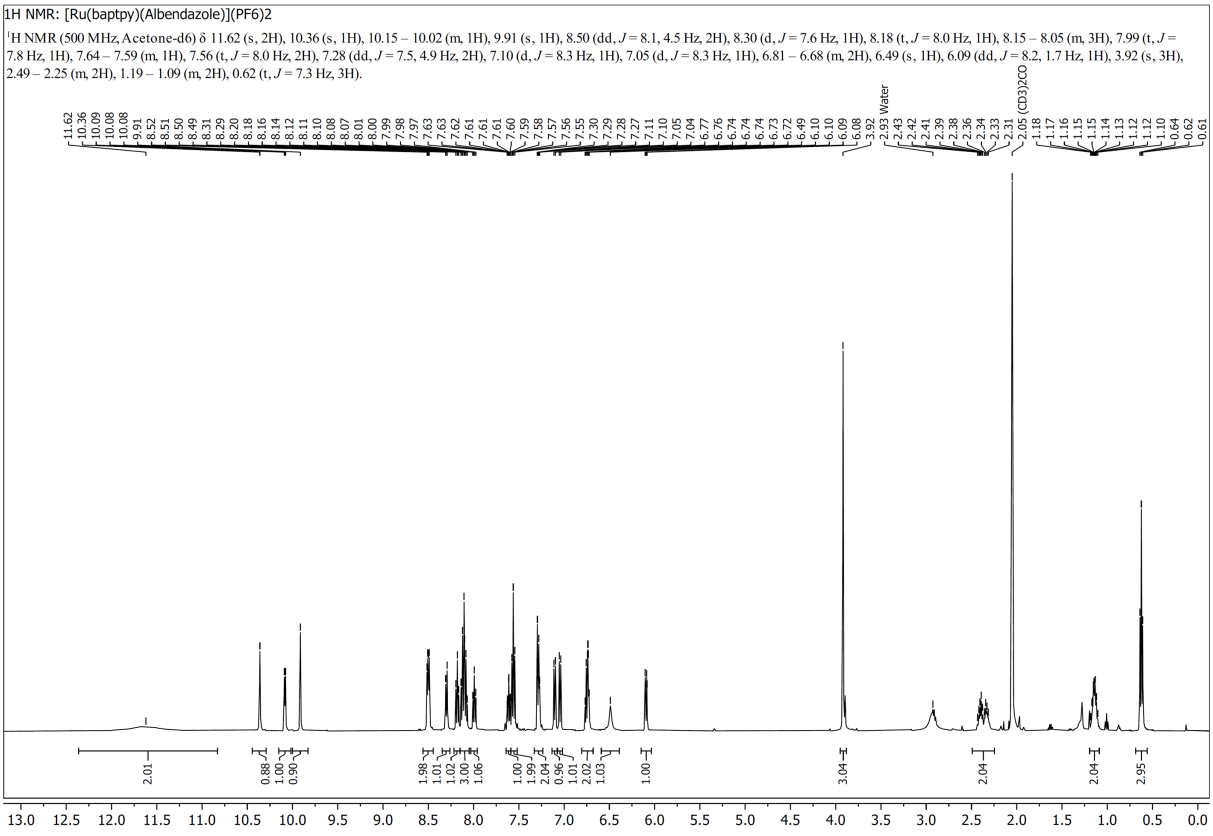

Figure S79. ^{1}H NMR of [**14**](PF_6)$_2$ in Acetone-d_6.

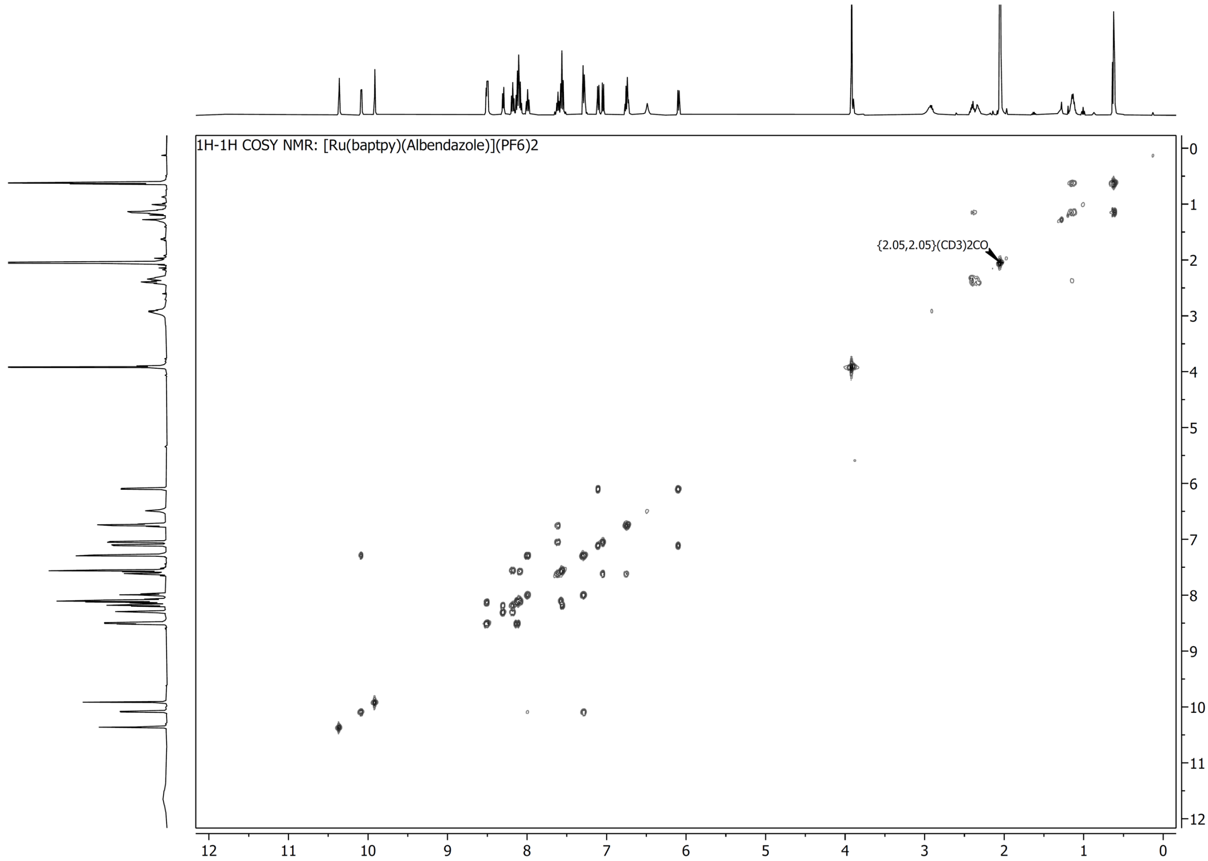

Figure S80. ^{1}H-^{1}H COSY NMR of [**14**](PF_6)$_2$ in Acetone-d_6.

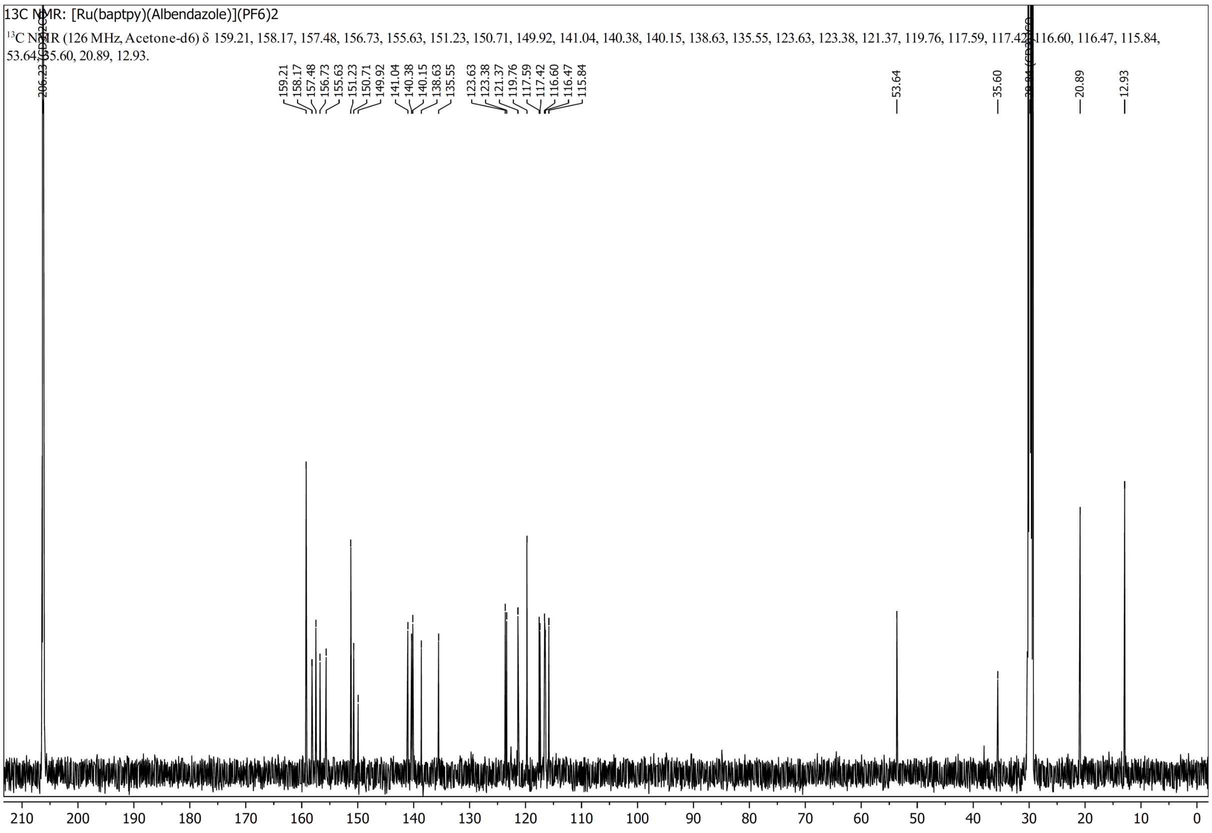

Figure S81. ^{13}C NMR of [**14**]$(PF_6)_2$ in Acetone-*d6*.

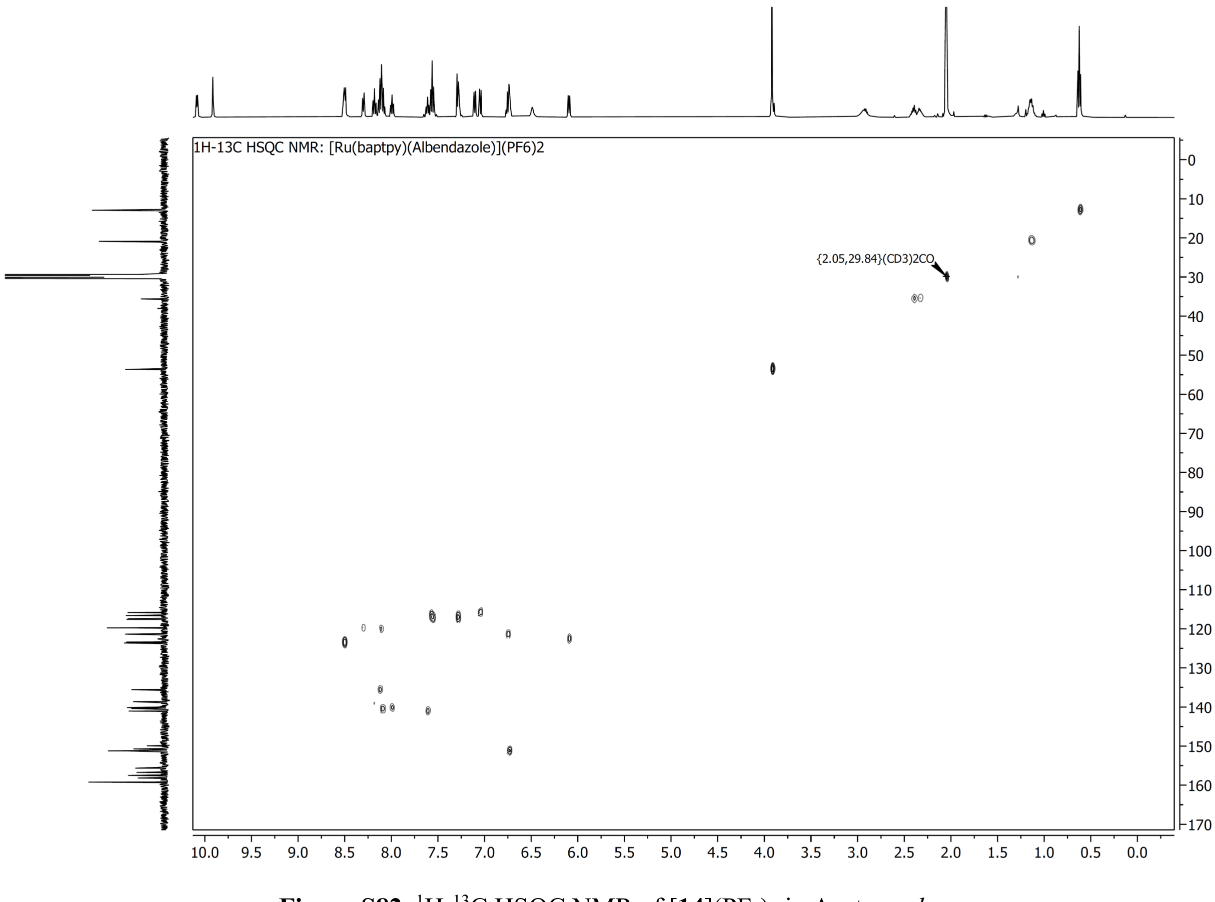

Figure S82. ^1H-^{13}C HSQC NMR of [**14**]$(PF_6)_2$ in Acetone-*d6*.

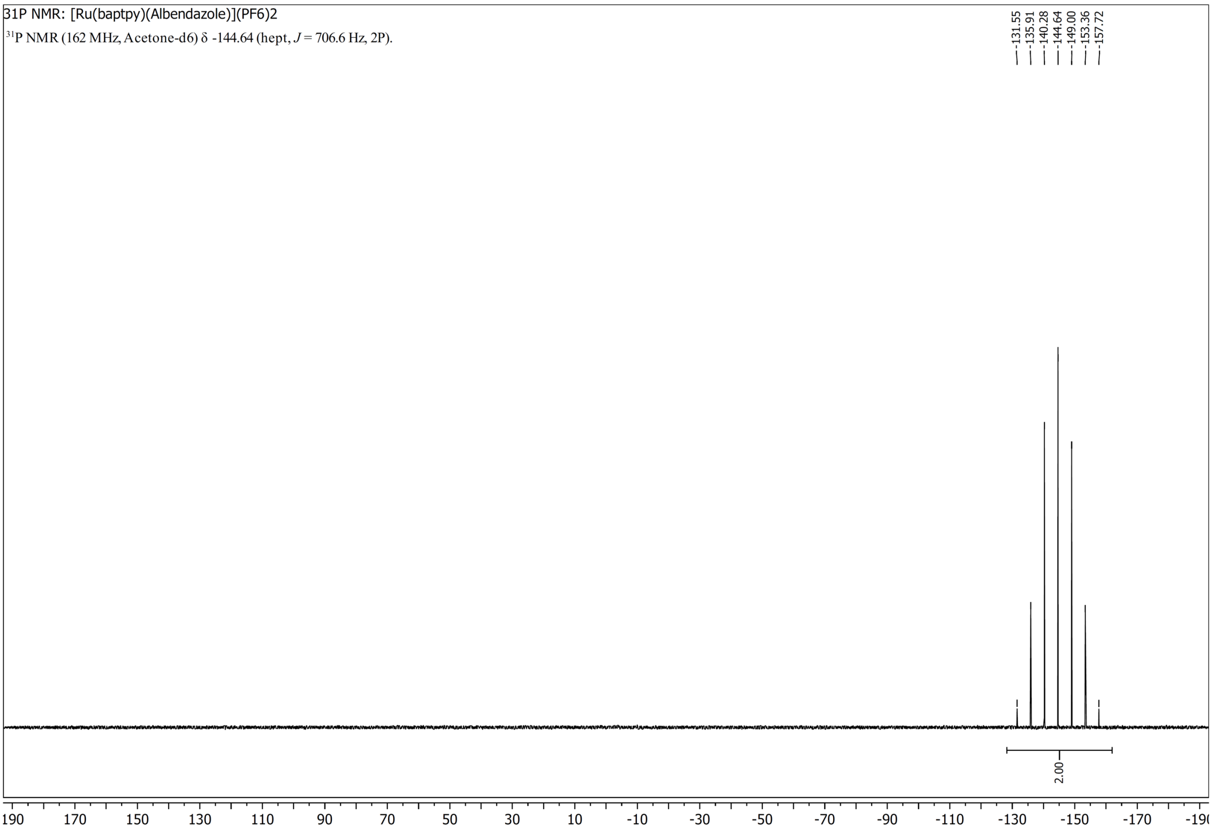

Figure S83. ^{31}P NMR of [**14**](PF_6)$_2$ in Acetone-*d*$_6$.

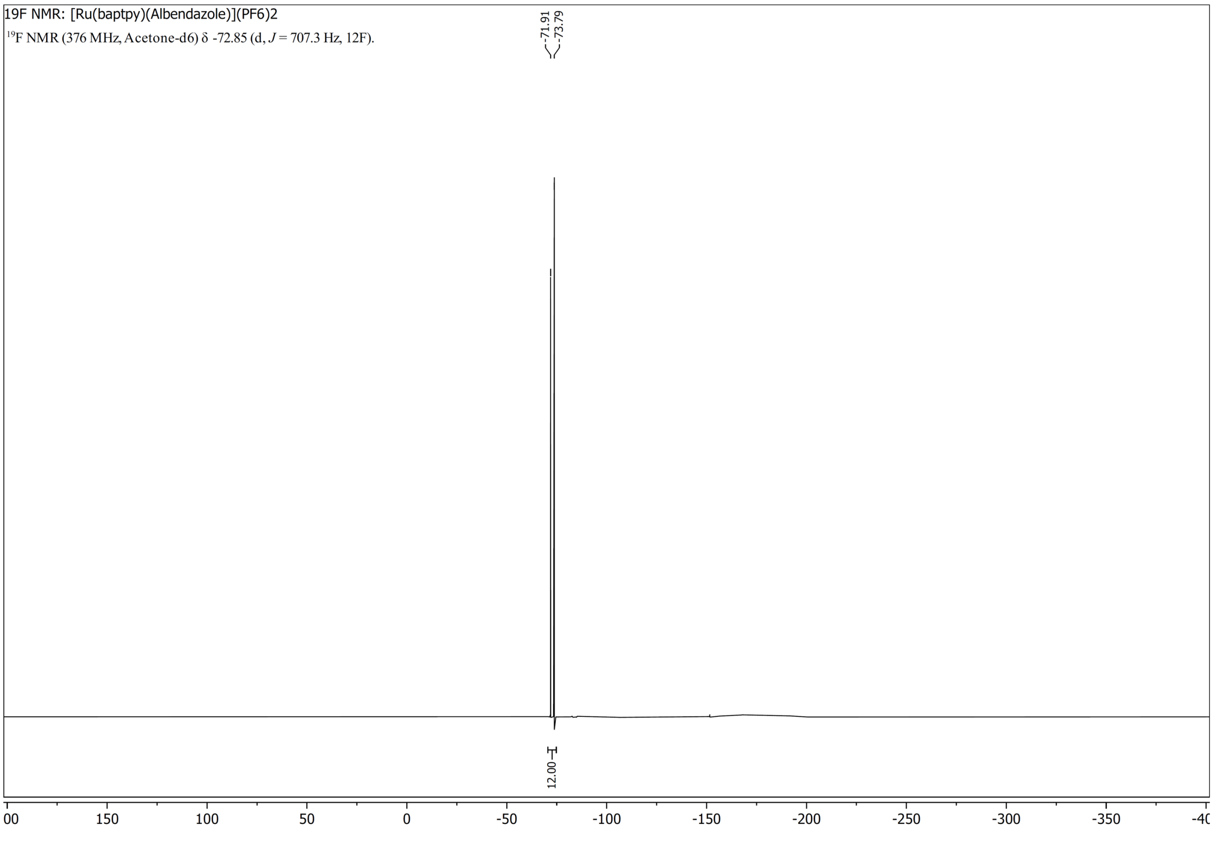

Figure S84. ^{19}F NMR of [**14**](PF_6)$_2$ in Acetone-*d*$_6$.

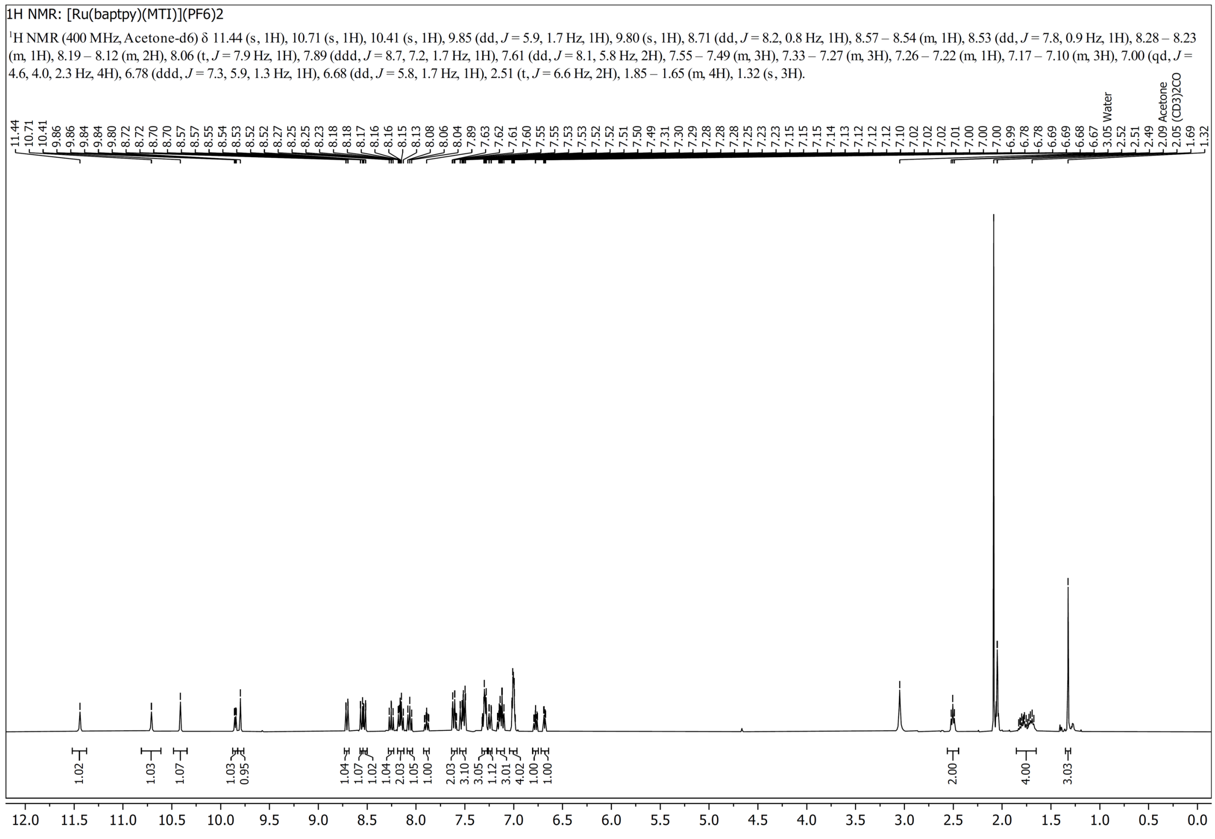

Figure S85. ^{1}H NMR of [**15**](PF_6)$_2$ in Acetone-d_6.

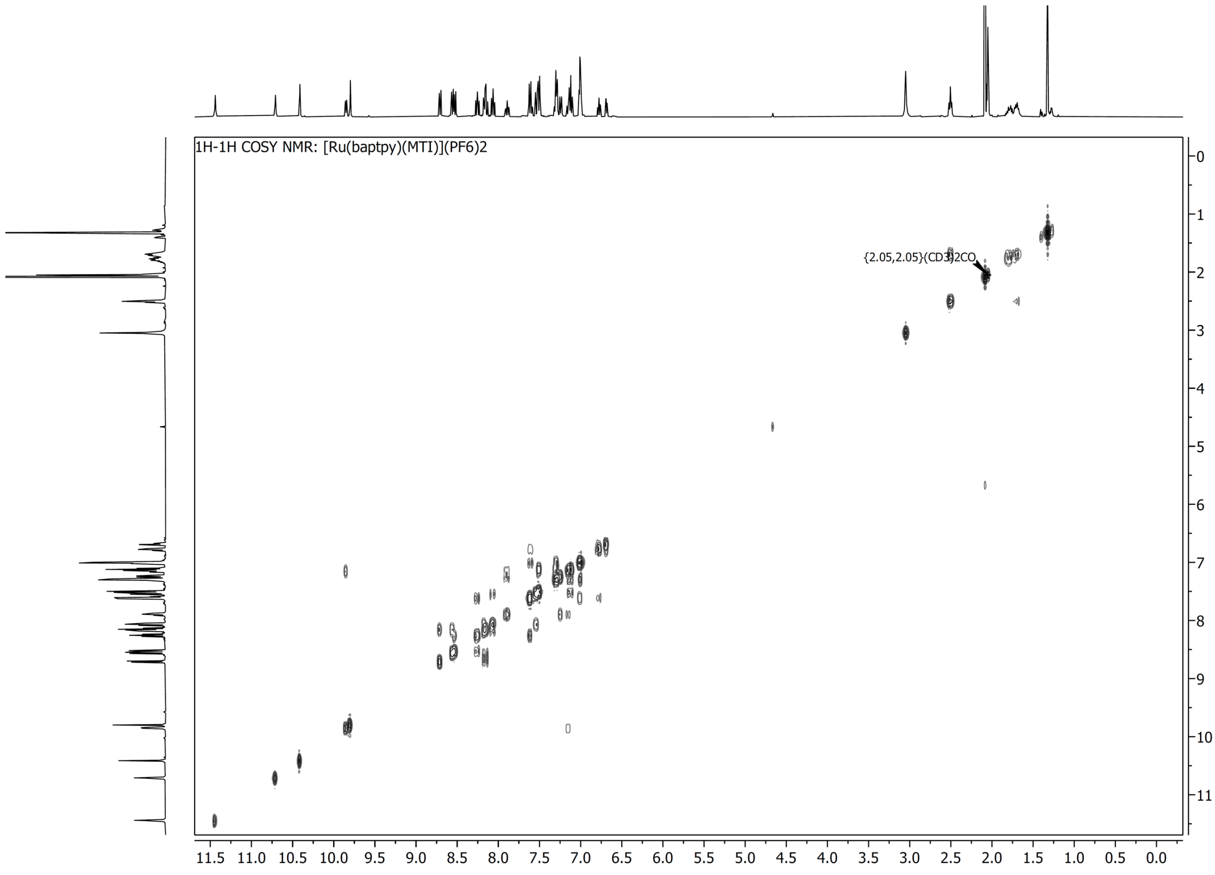

Figure S86. ^{1}H-^{1}H COSY NMR of [1**5**](PF_6)$_2$ in Acetone-d_6.

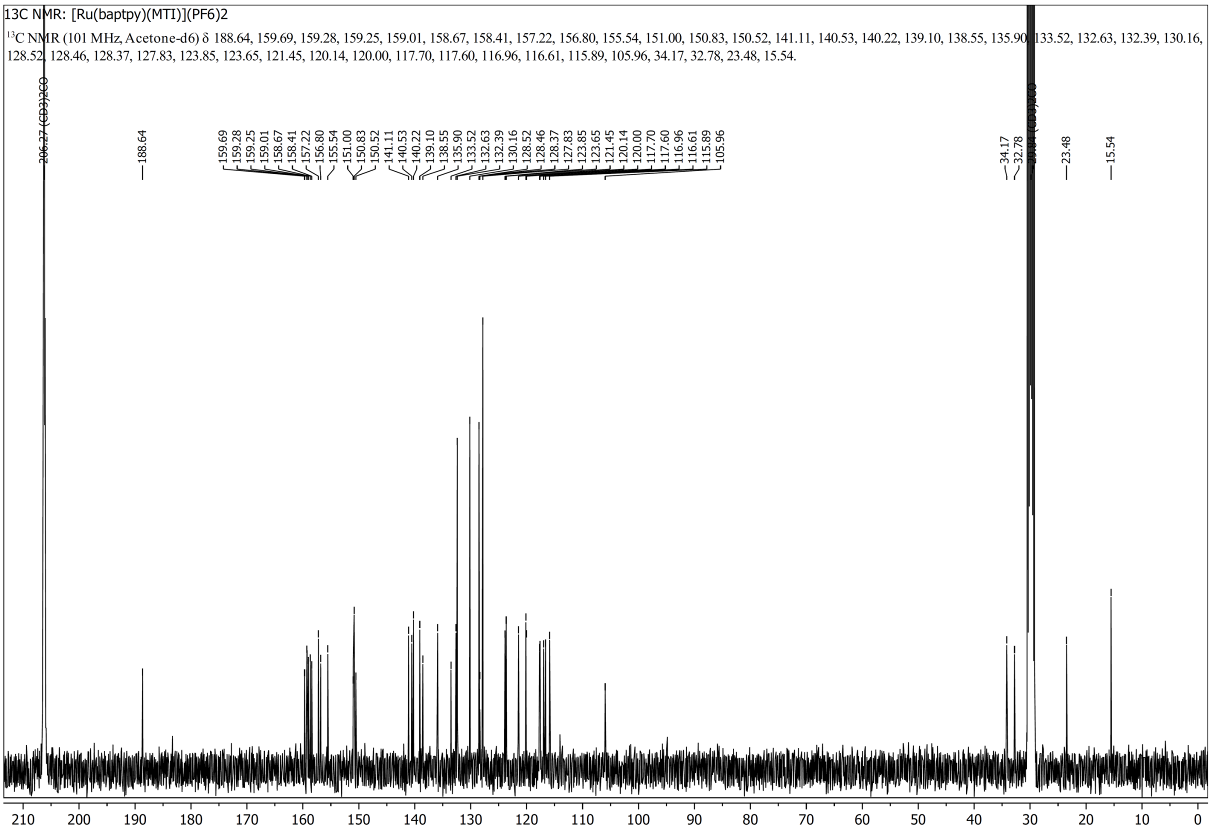

Figure S87. ^{13}C NMR of [1**5**](PF_6)$_2$ in Acetone-d_6.

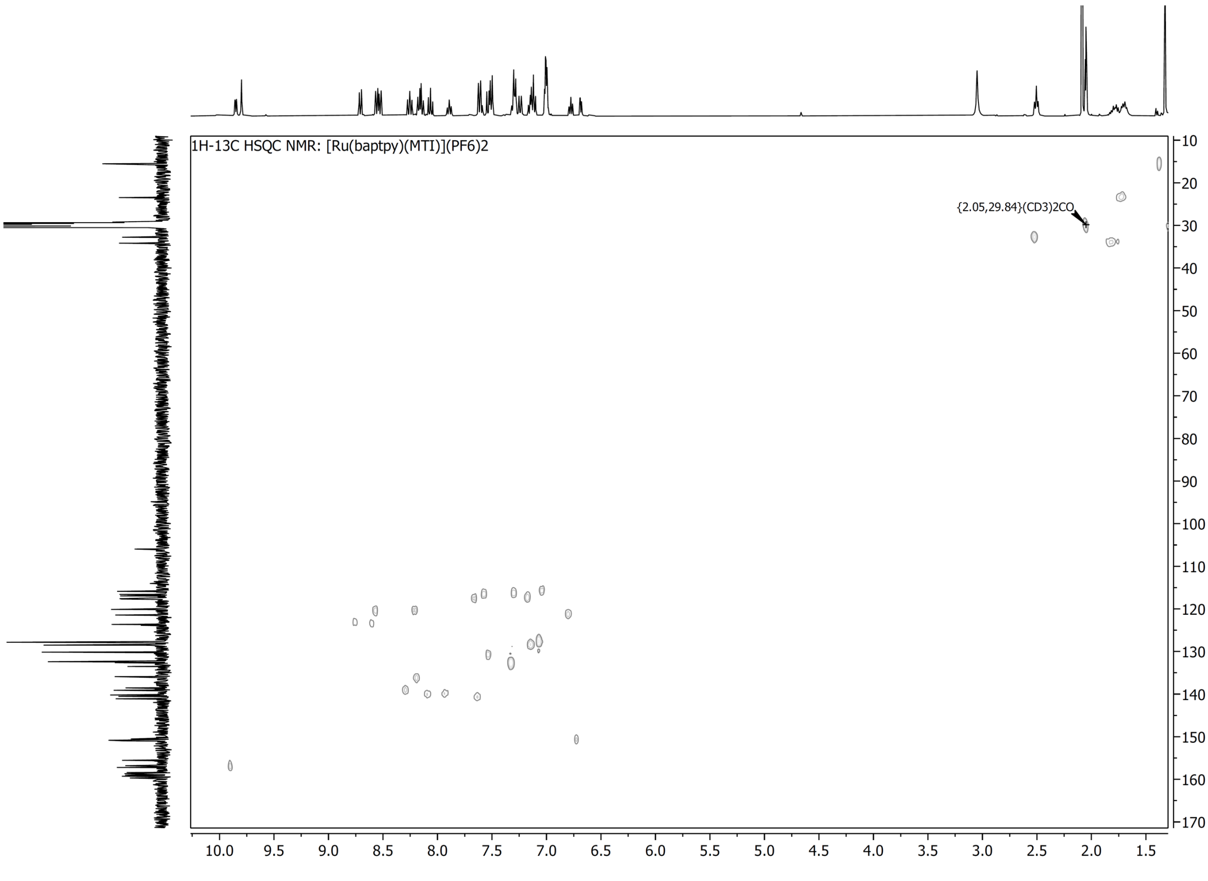

Figure S88. ^{1}H-^{13}C HSQC NMR of [1**5**](PF_6)$_2$ in Acetone-d_6.

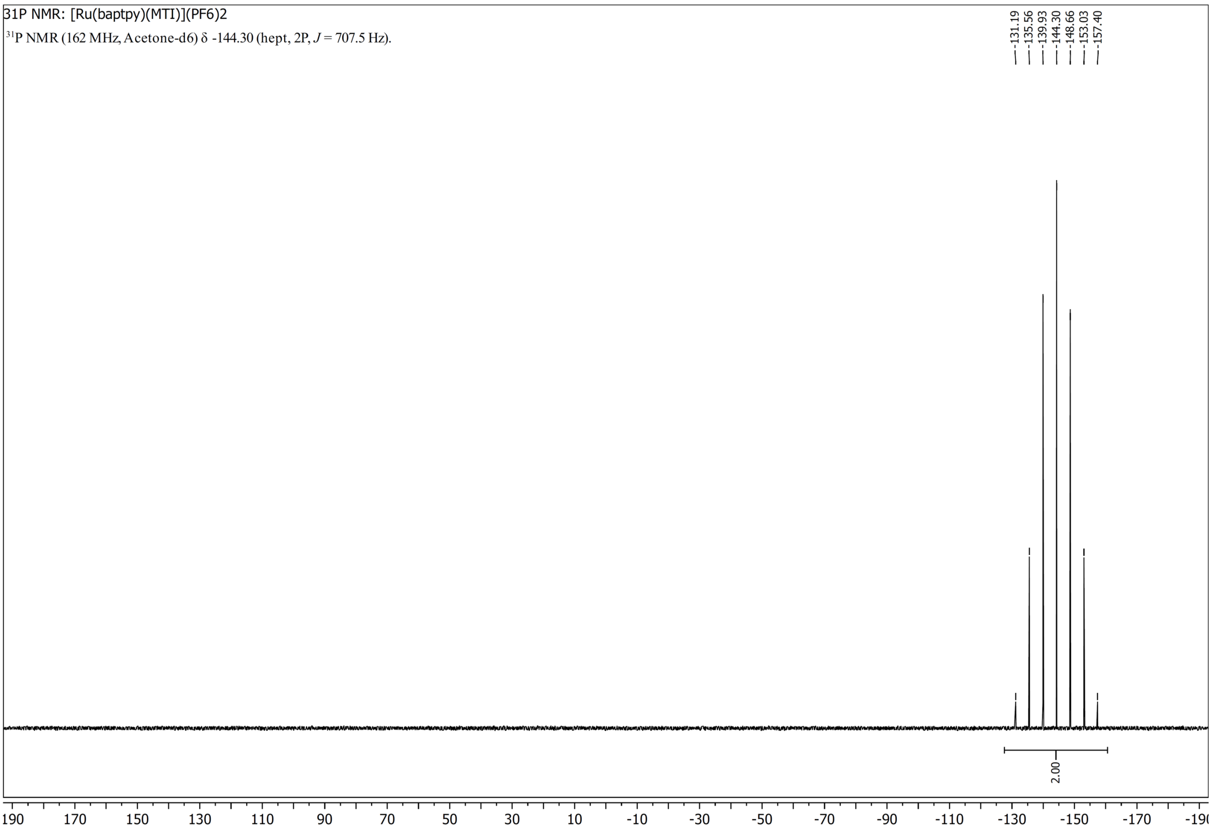

Figure S89. ^{31}P NMR of [1**5**](PF_6)$_2$ in Acetone-d_6.

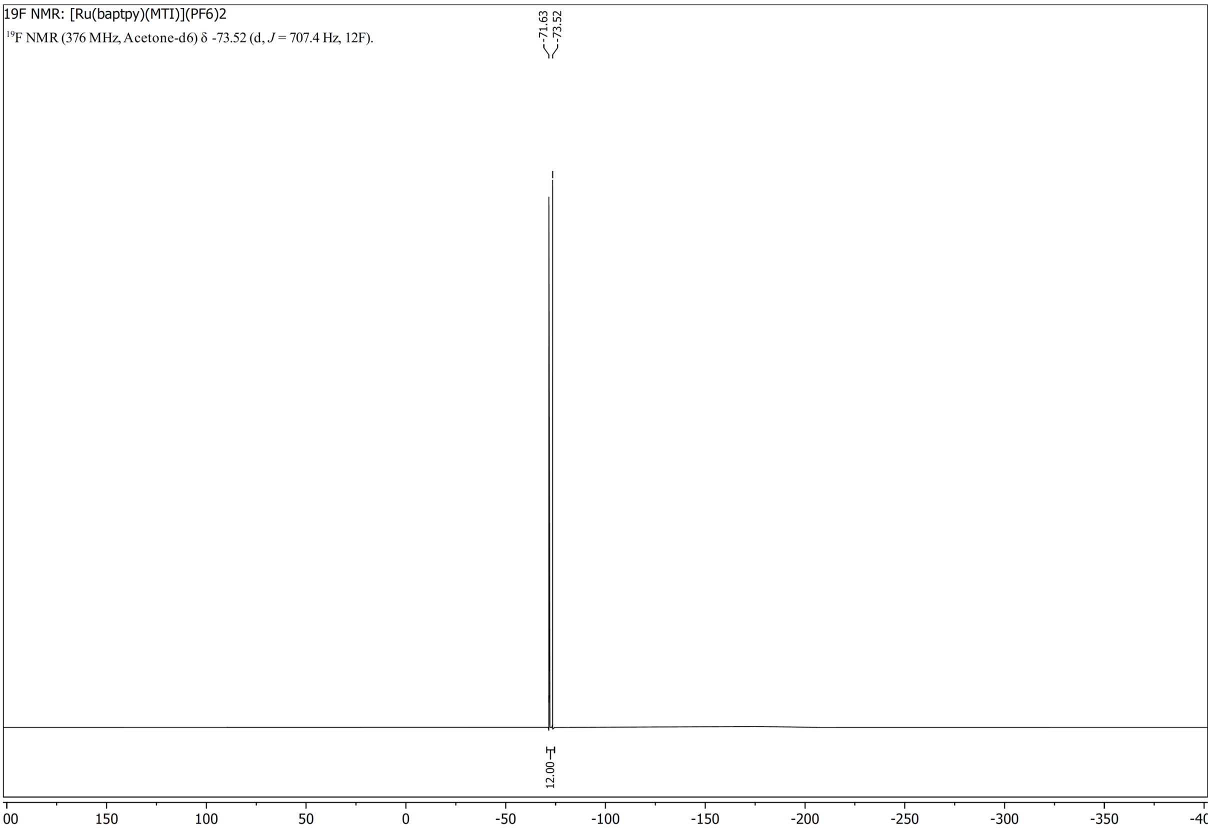

Figure S90. ^{19}F NMR of [1**5**](PF_6)$_2$ in Acetone-d_6.

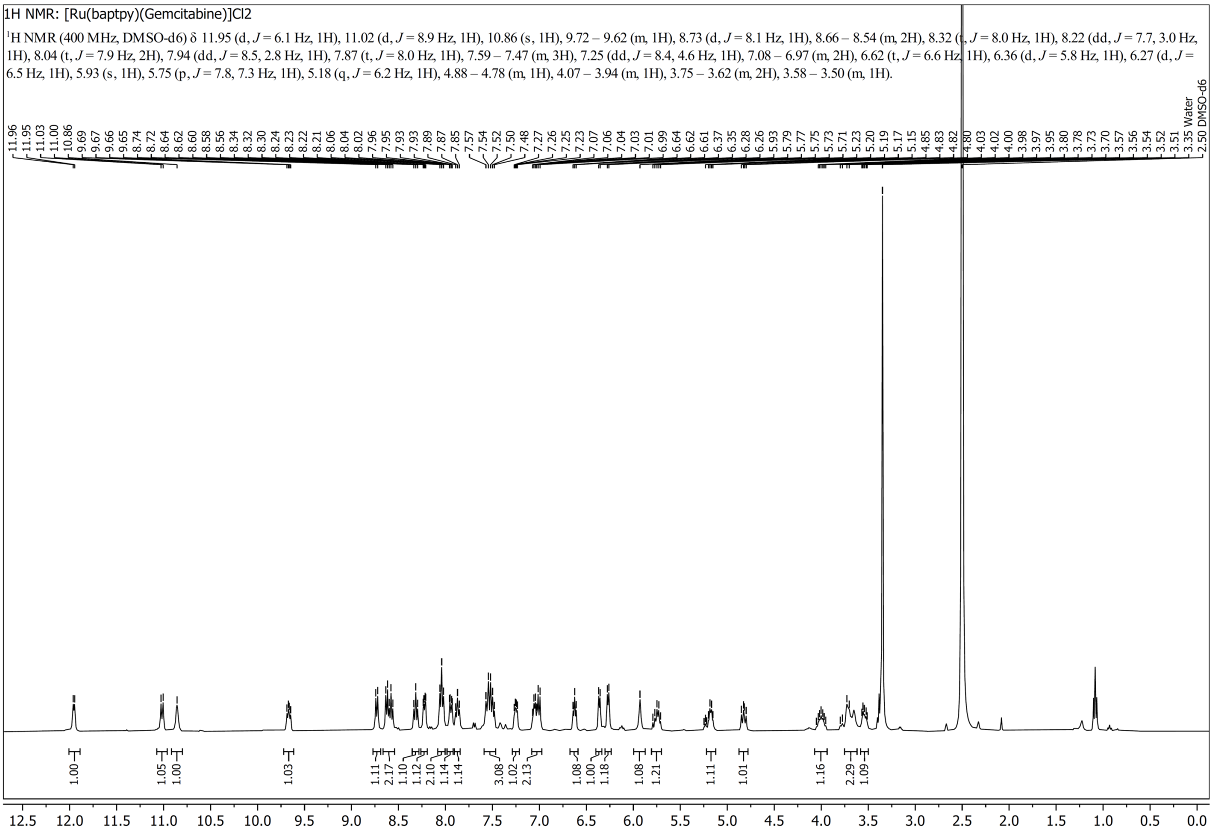

Figure S91. ^{1}H NMR of [**16**]Cl_2 in DMSO-d_6.

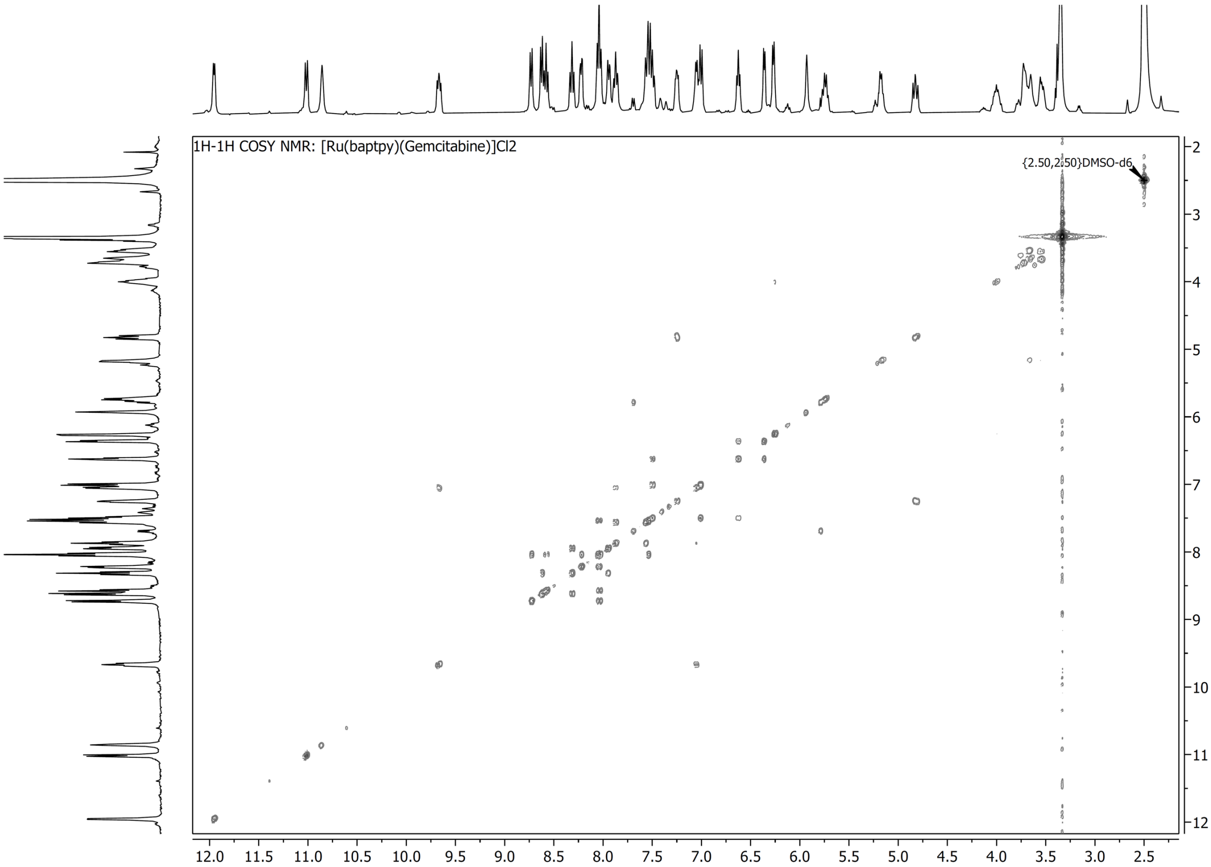

Figure S92. ^{1}H-^{1}H COSY NMR of [**16**]Cl_2 in DMSO-d_6.

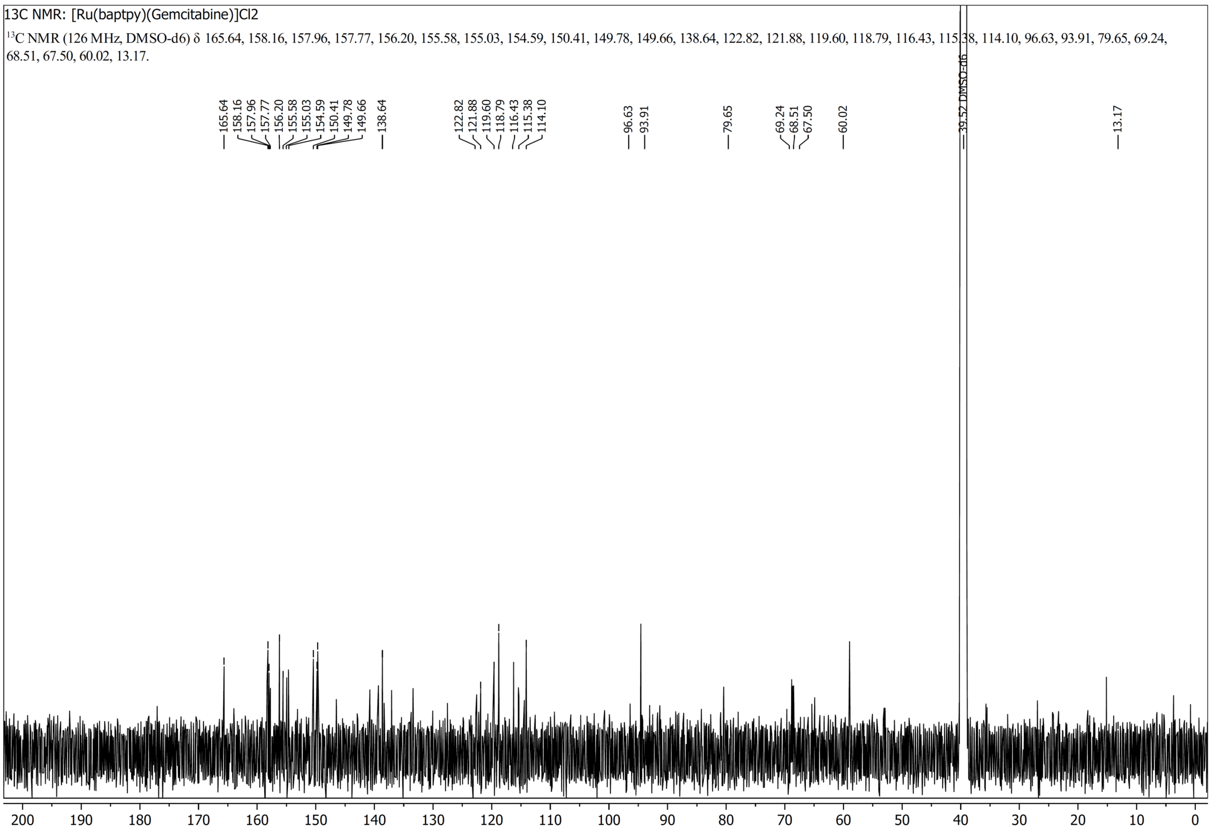

Figure S93. ^{13}C NMR of [**16**]Cl_2 in DMSO-*d6*.

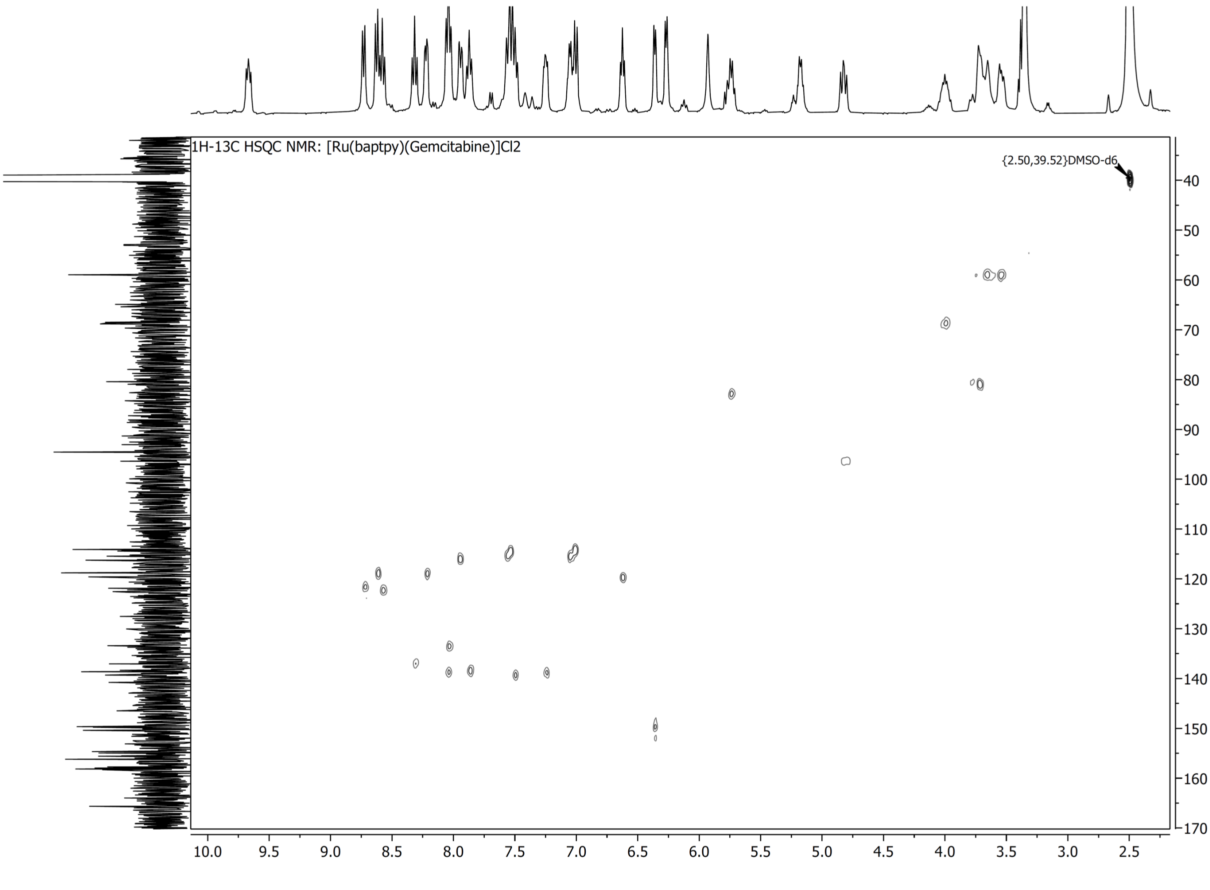

Figure S94. ^{1}H-^{13}C HSQC NMR of [**16**]Cl_2 in DMSO-*d6*.

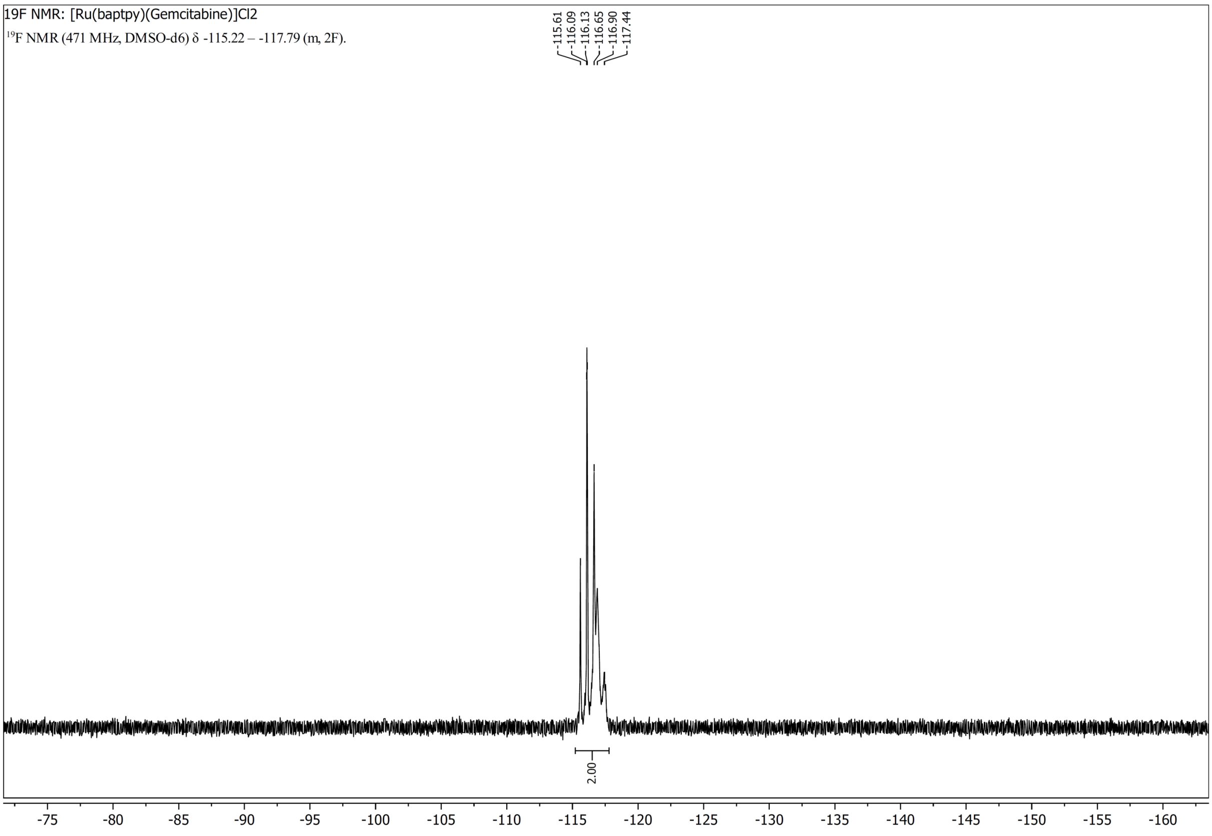

Figure S95. ^{19}F NMR of [**16**]Cl_2 in DMSO-d_6.

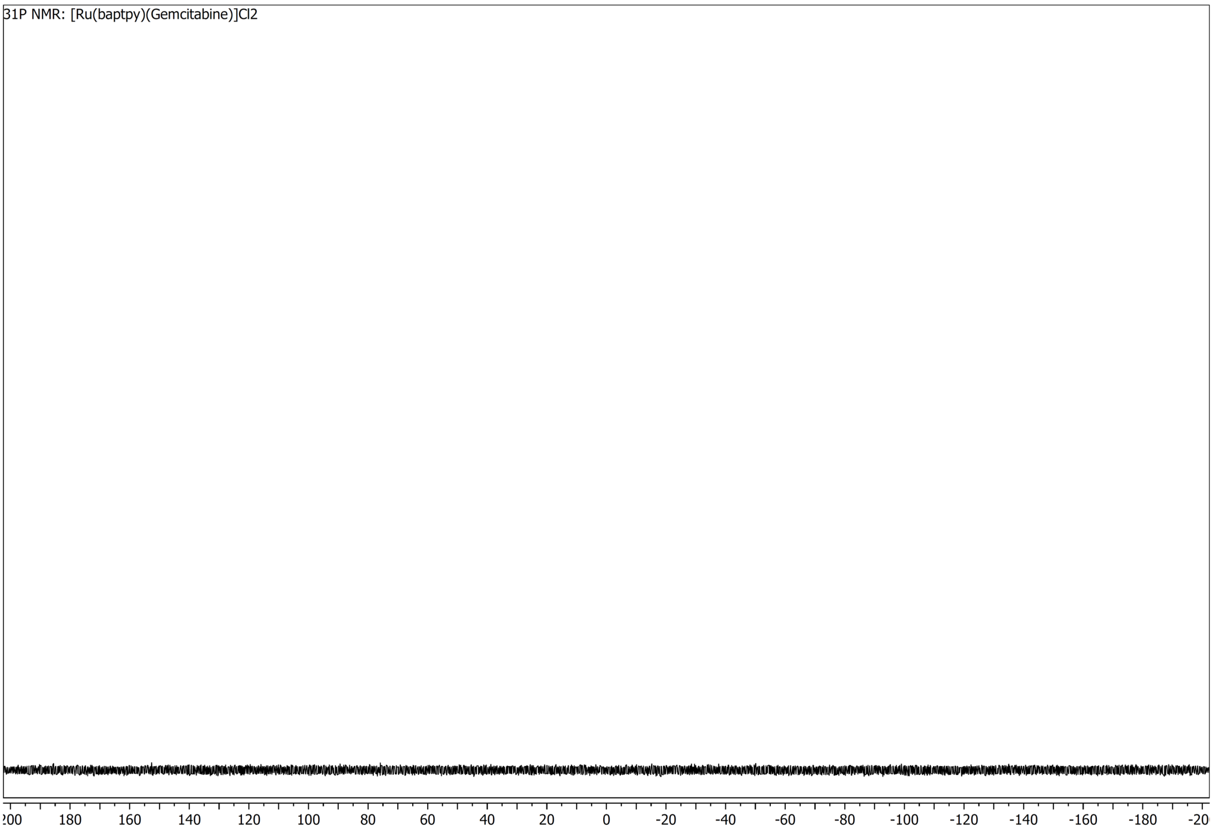

Figure S96. ^{31}P NMR of [**16**]Cl_2 in DMSO-d_6.

1.5 Mass spectrometry data

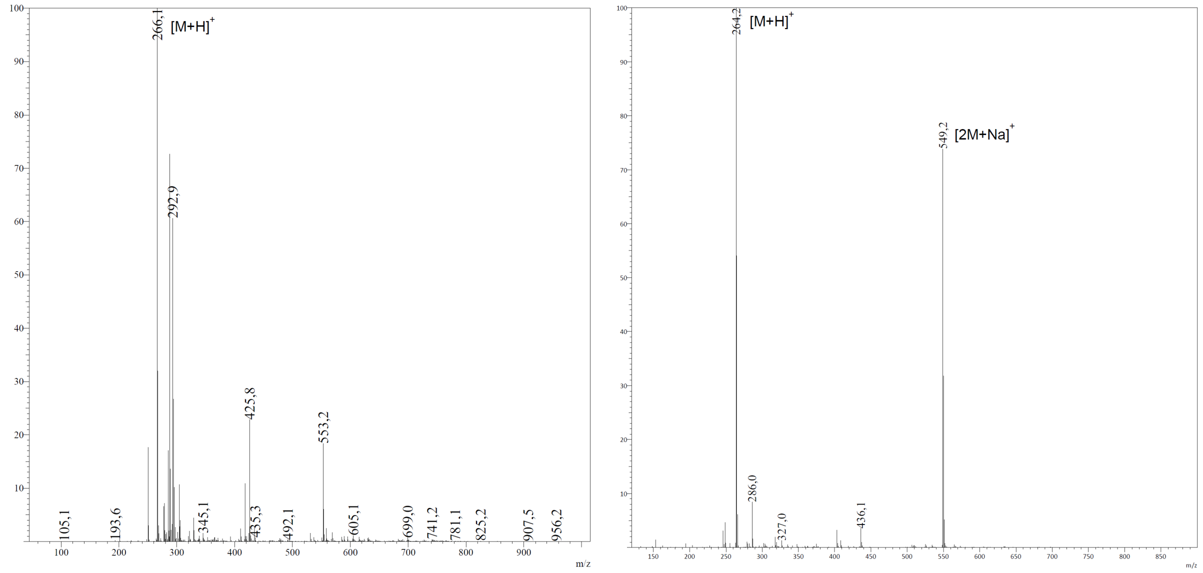

Figure S97. ESI-MS of **1** (left) and **2** (right) in MeOH.

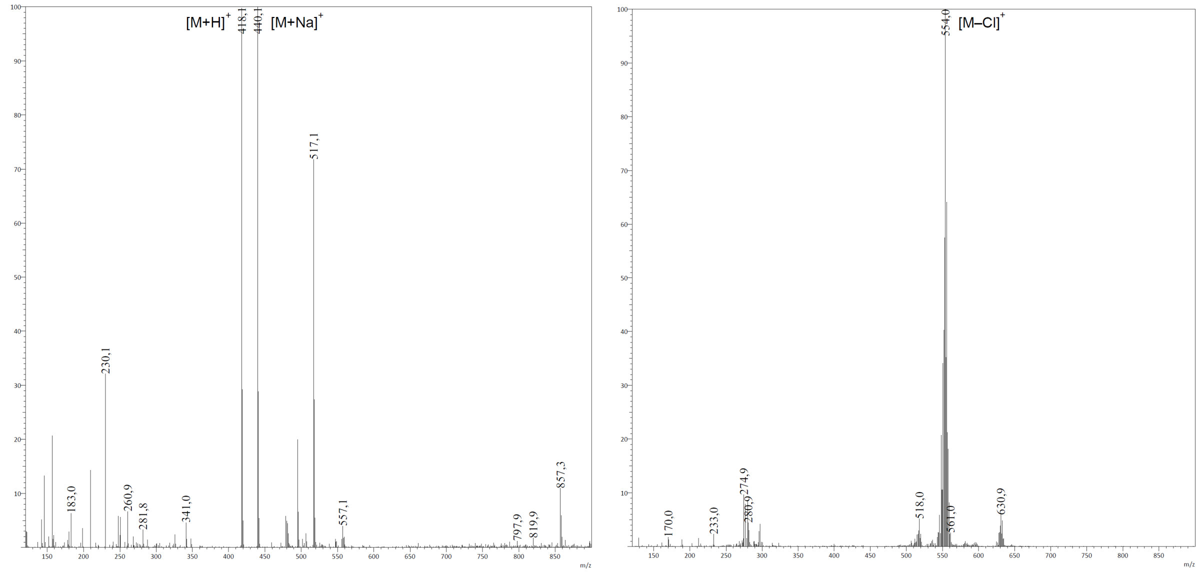

Figure S98. ESI-MS of **3** (left) and [**4**]Cl (right) in MeOH.

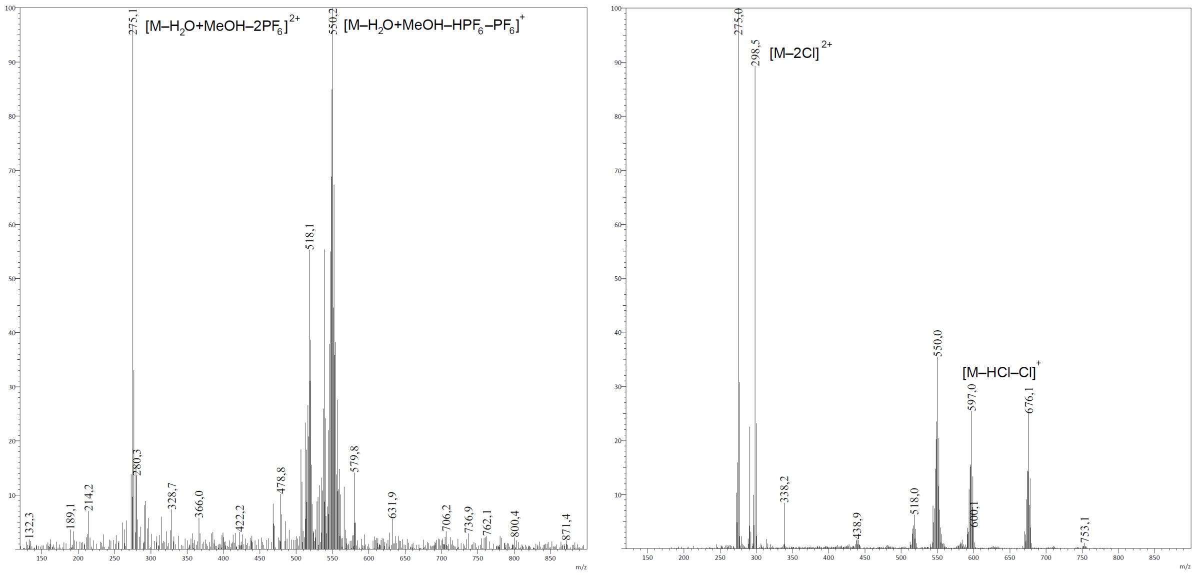

Figure S99. ESI-MS of [**5**](PF_6)$_2$ (left) and [**6**]Cl_2 (right) in MeOH.

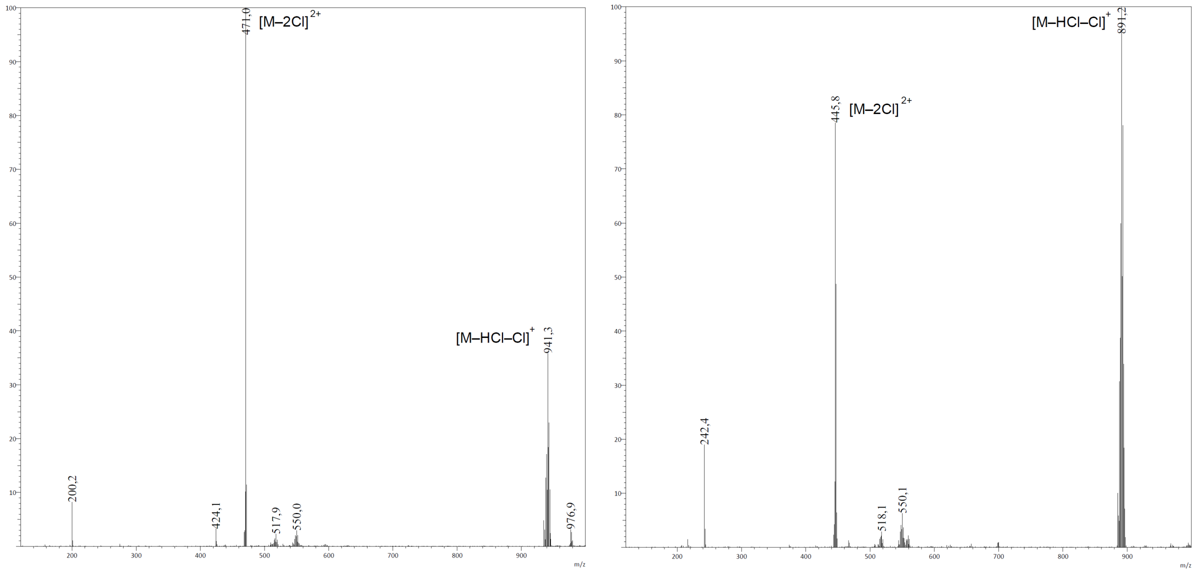

Figure S100. ESI-MS of [**7**]Cl_2 (left) and [**8**]Cl_2 (right) in MeOH.

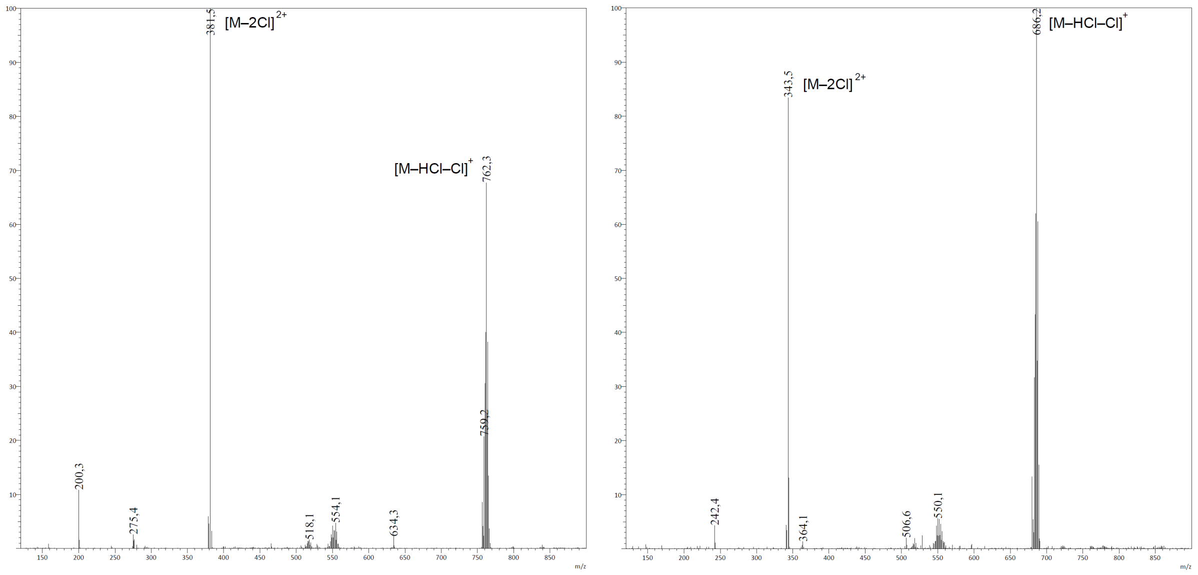

Figure S101. ESI-MS of [**9**]Cl_2 (left) and [**10**]Cl_2 (right) in MeOH.

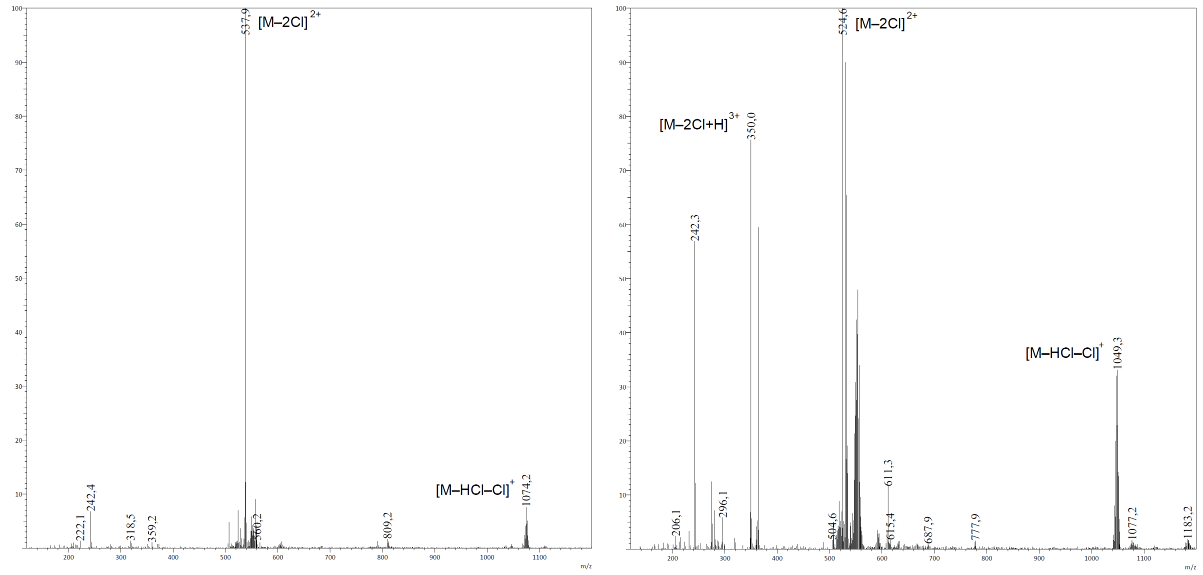

Figure S102. ESI-MS of [**11**]Cl_2 (left) and [**12**]Cl_2 (right) in MeOH.

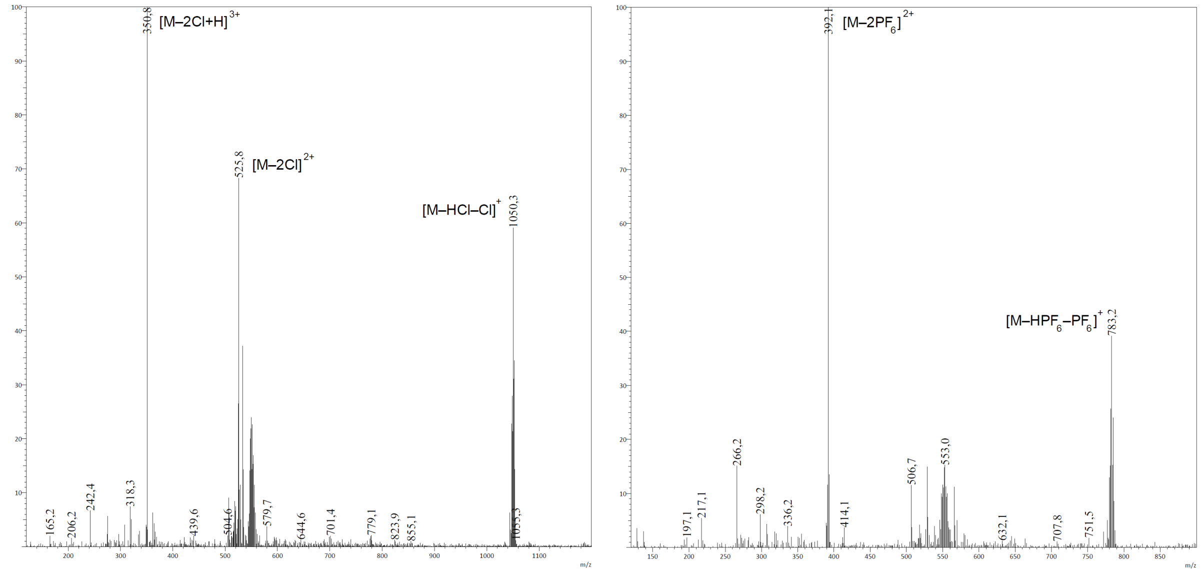

Figure S103. ESI-MS of [**13**]Cl_2 (left) and [**14**]$(PF_6)_2$ (right) in MeOH.

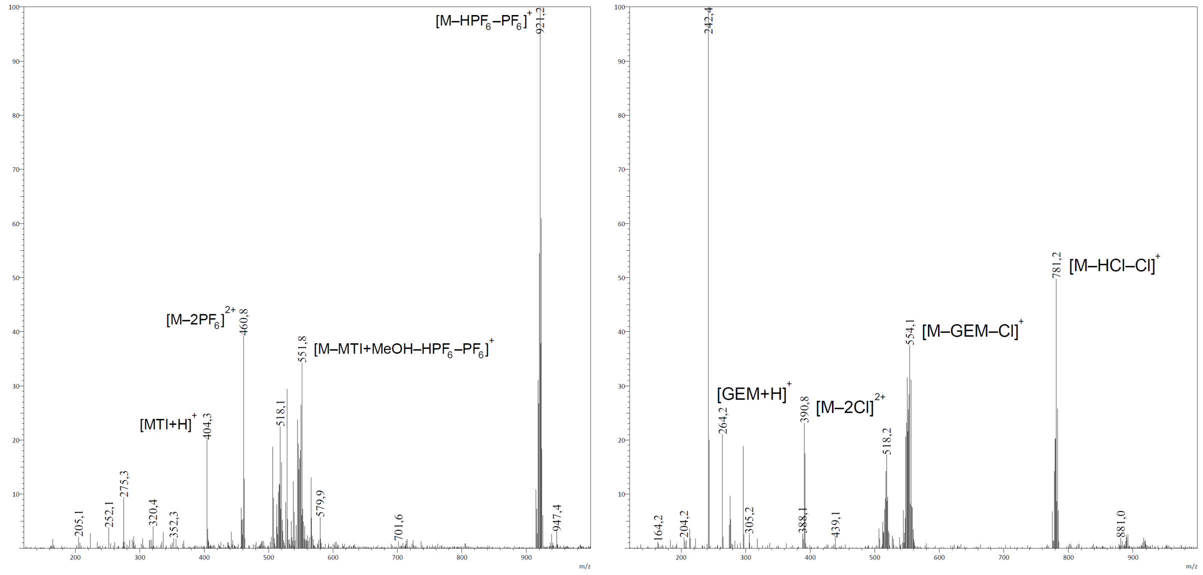

Figure S104. ESI-MS of [**15**]$(PF_6)_2$ (left) and [**16**]Cl_2 (right) in MeOH.

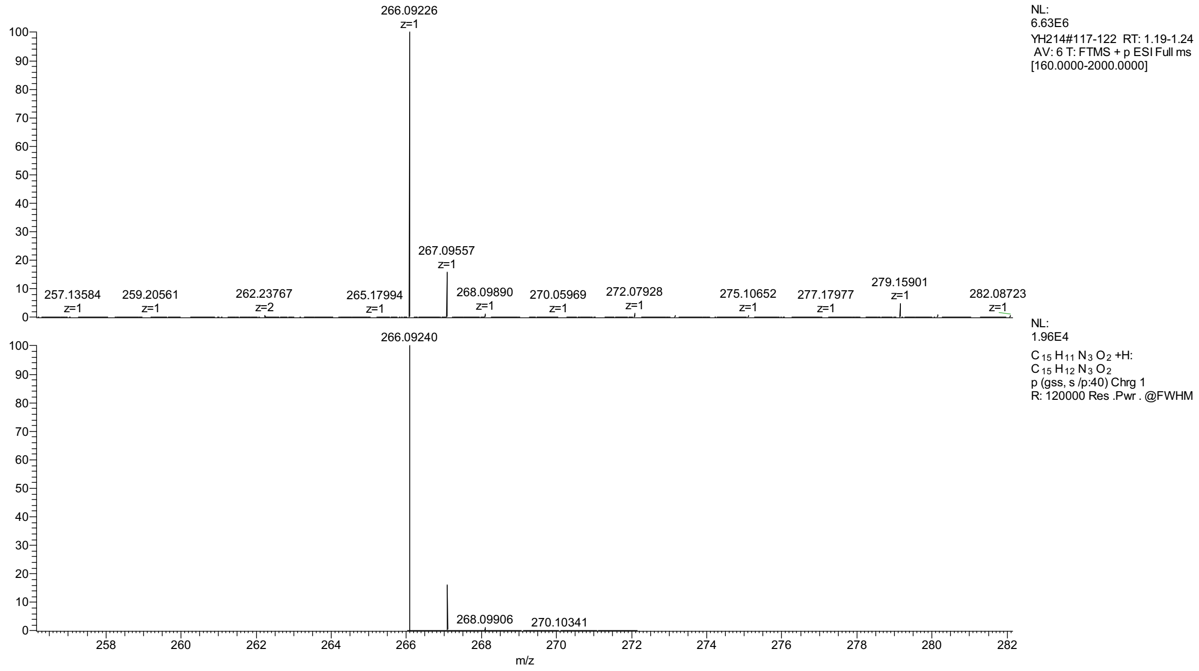

Figure S105. HRMS of **1** in MeOH, measured (top) and calculated (bottom).

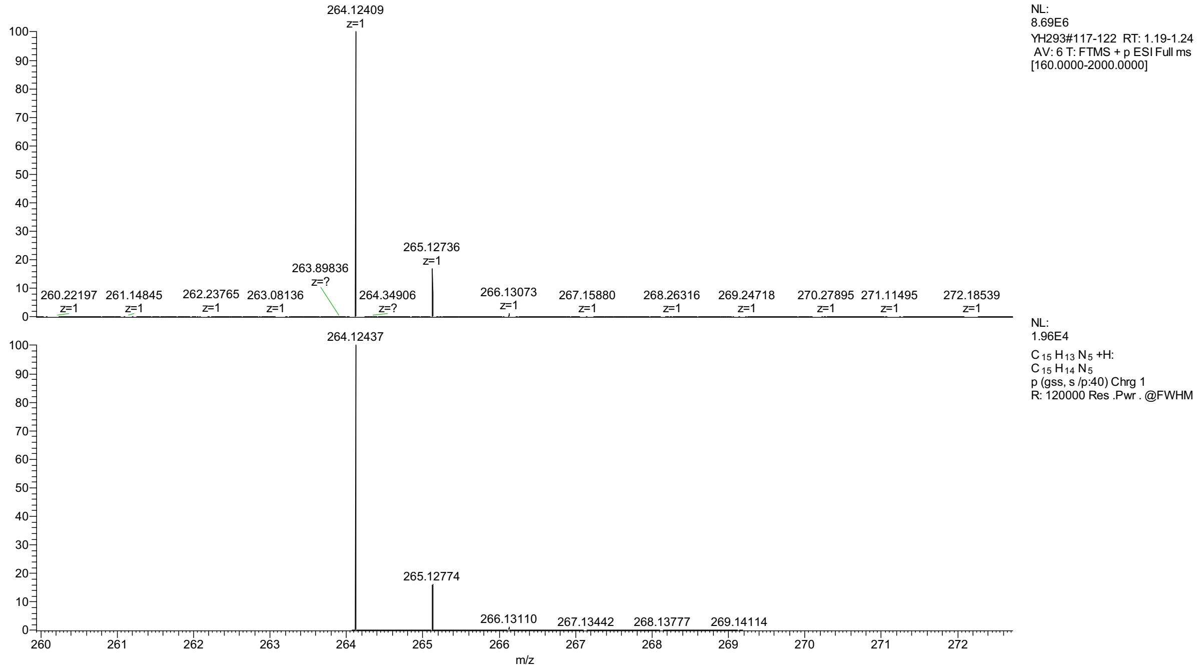

Figure S106. HRMS of **2** in MeOH, measured (top) and calculated (bottom).

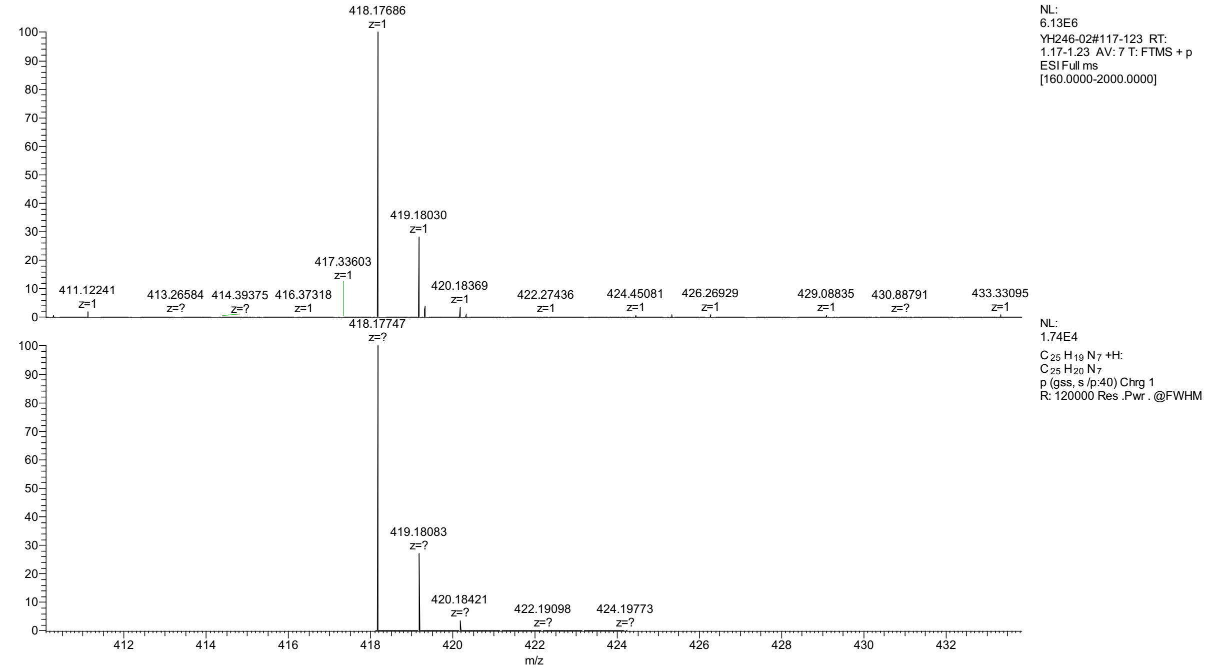

Figure S107. HRMS of **3** in MeOH, measured (top) and calculated (bottom).

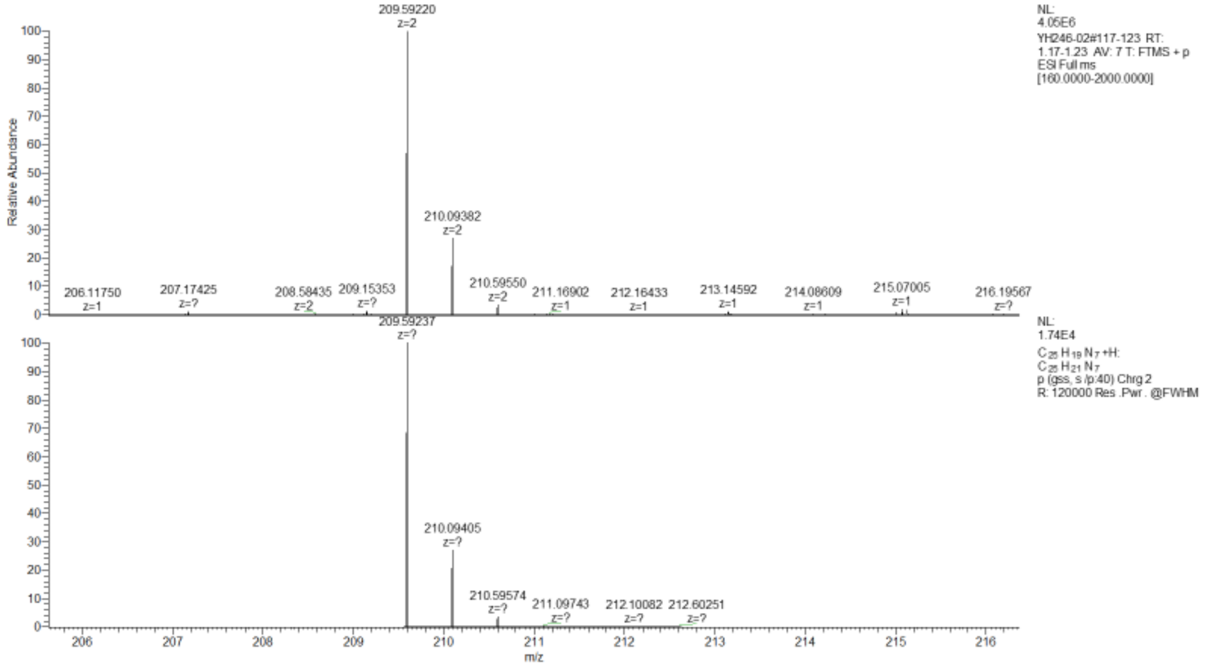

Figure S108. HRMS of **3** in MeOH, measured (top) and calculated (bottom).

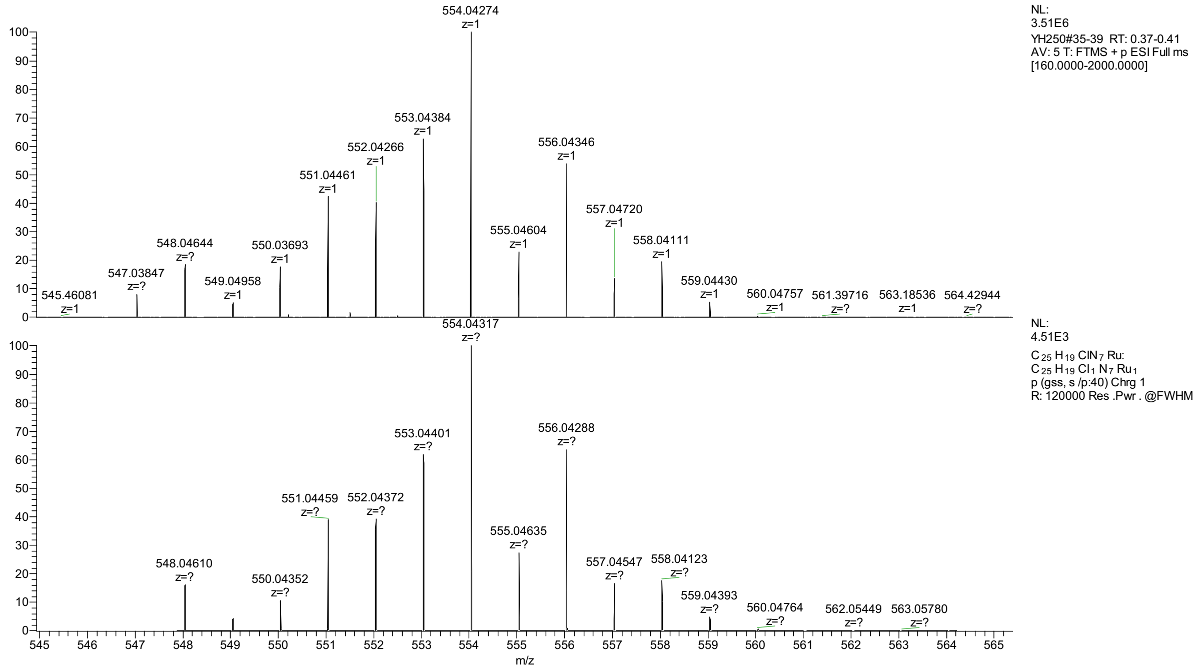

Figure S109. HRMS of [**4**]Cl in MeOH, measured (top) and calculated (bottom).

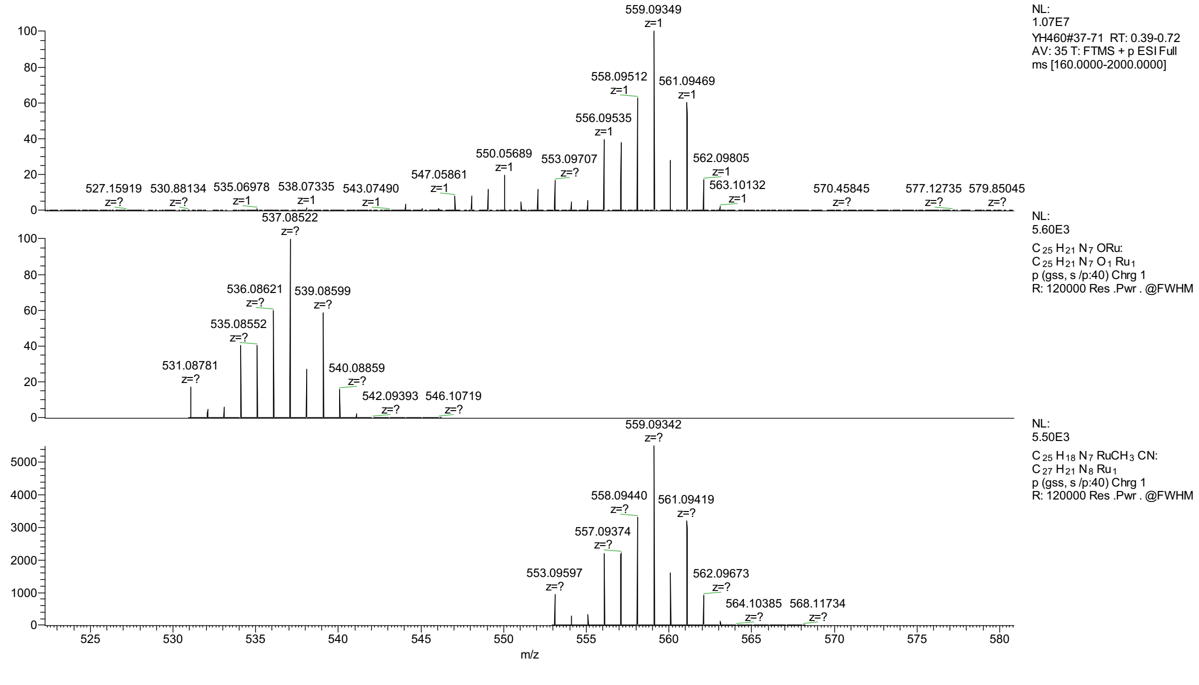

Figure S110. HRMS of [**5**]$(PF_6)_2$ in MeOH, measured (top) and calculated (bottom).

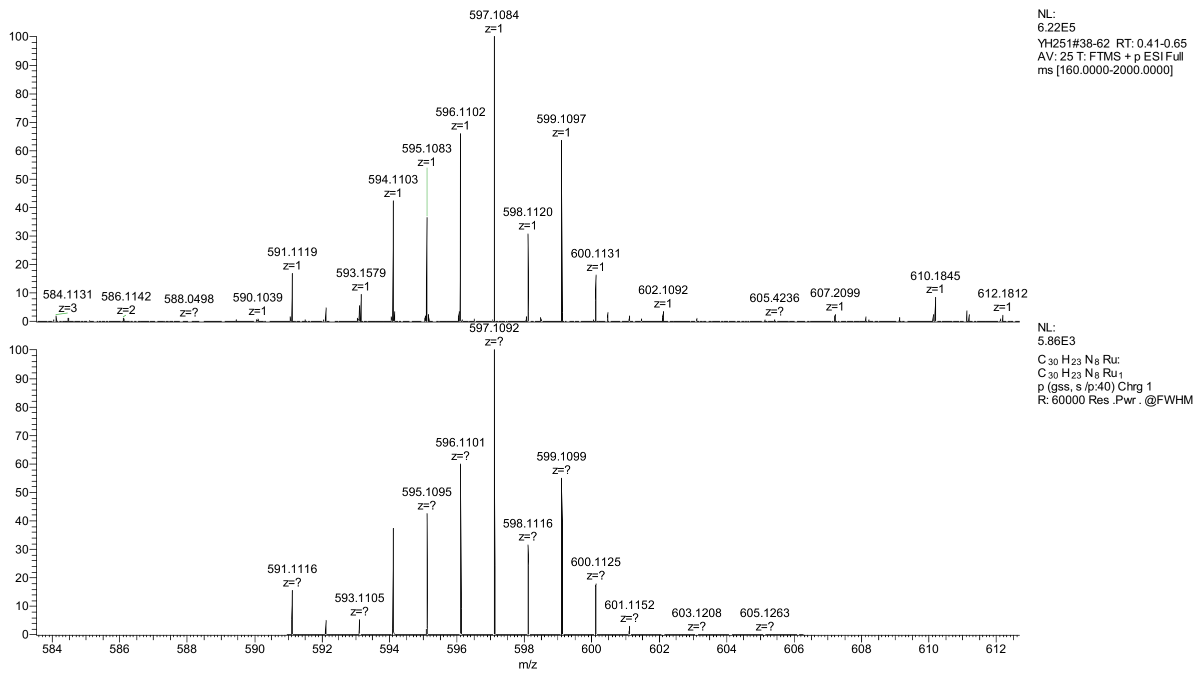

Figure S111. HRMS of [**6**]Cl_2 in MeOH, measured (top) and calculated (bottom).

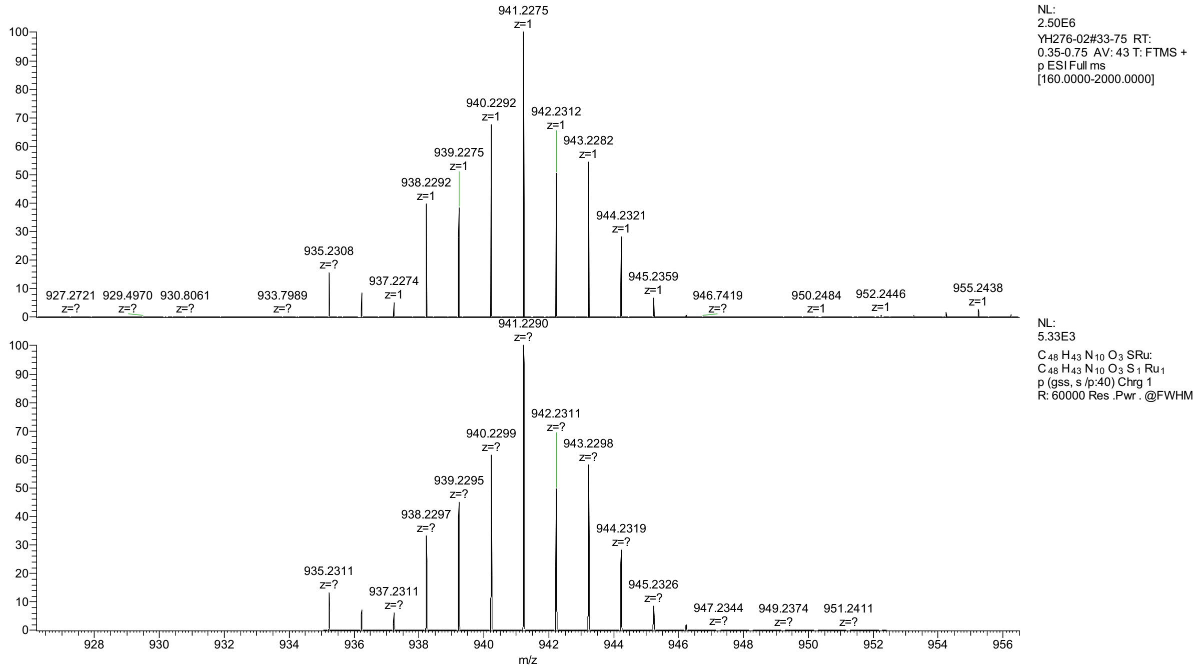

Figure S112. HRMS of [**7**]Cl_2 in MeOH, measured (top) and calculated (bottom).

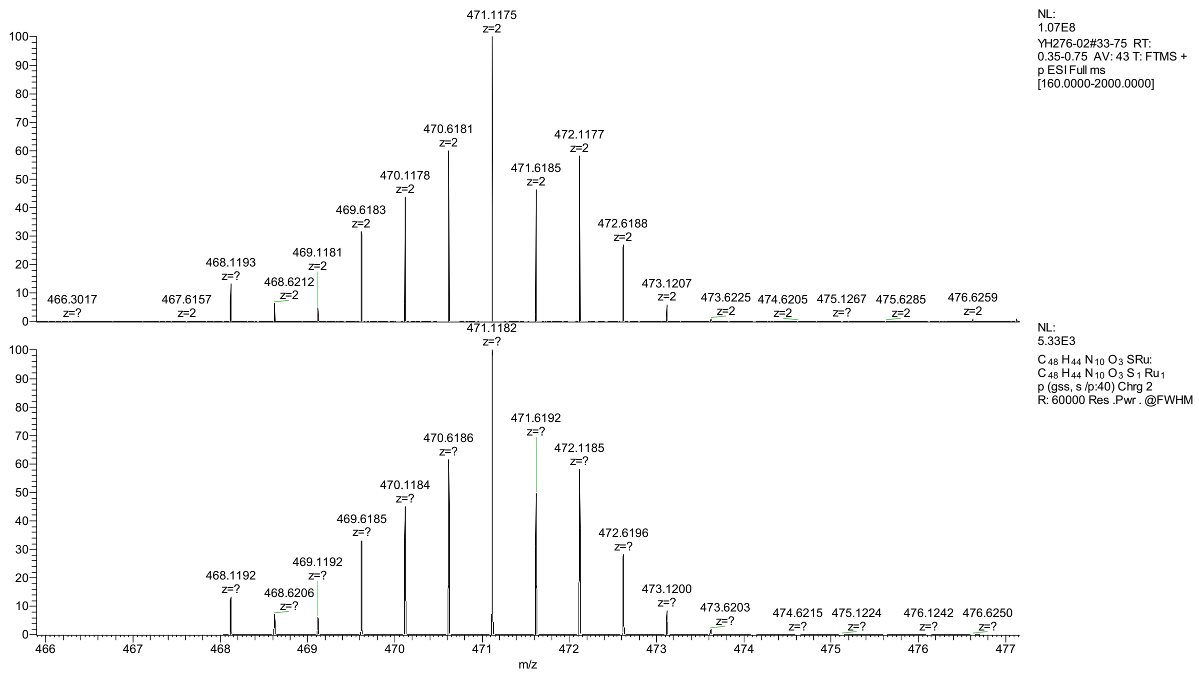

Figure S113. HRMS of [**7**]Cl2 in MeOH, measured (top) and calculated (bottom).

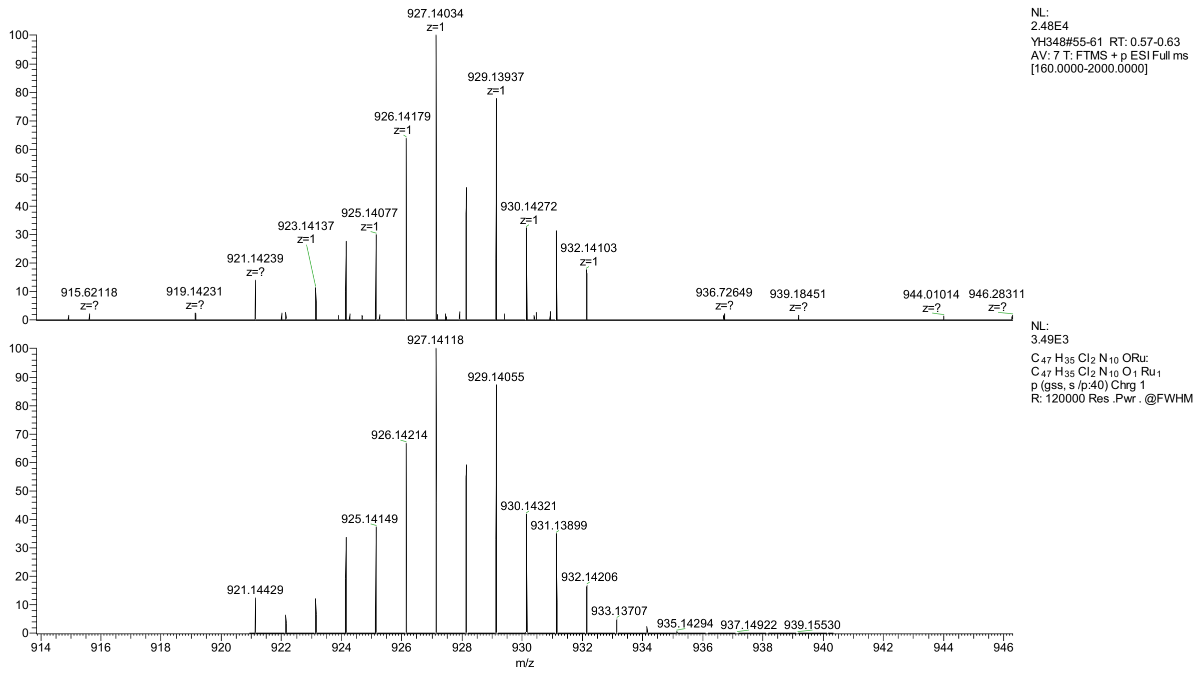

Figure S114. HRMS of [**8**]Cl2 in MeOH, measured (top) and calculated (bottom).

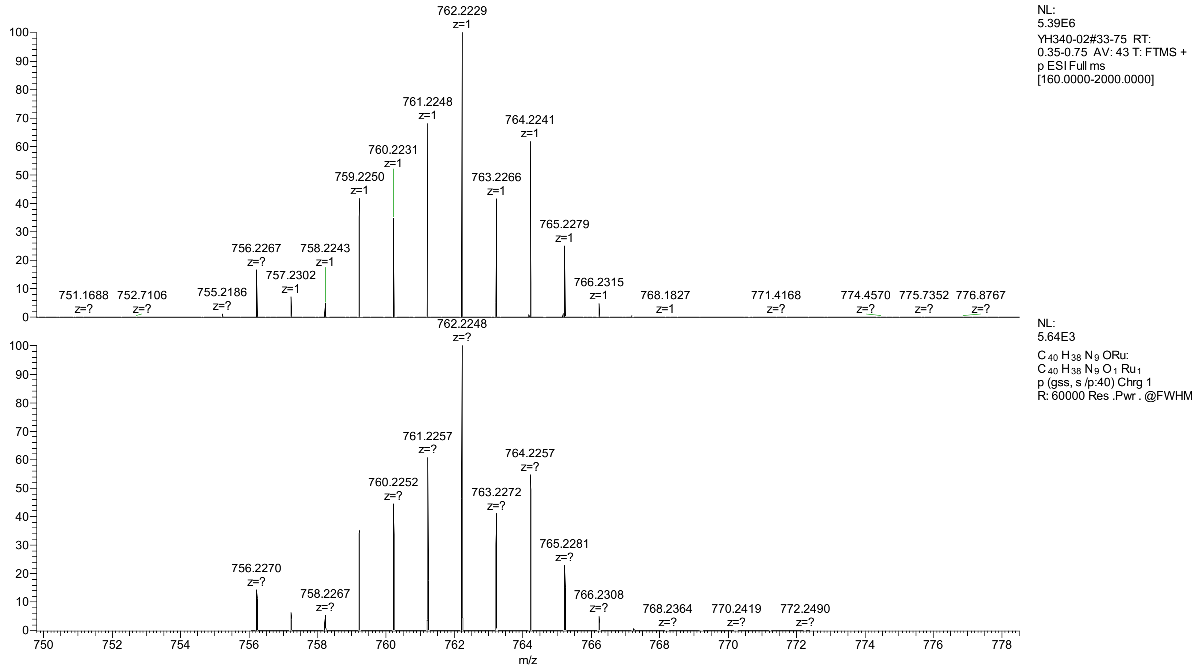

Figure S115. HRMS of [**9**]Cl_2 in MeOH, measured (top) and calculated (bottom).

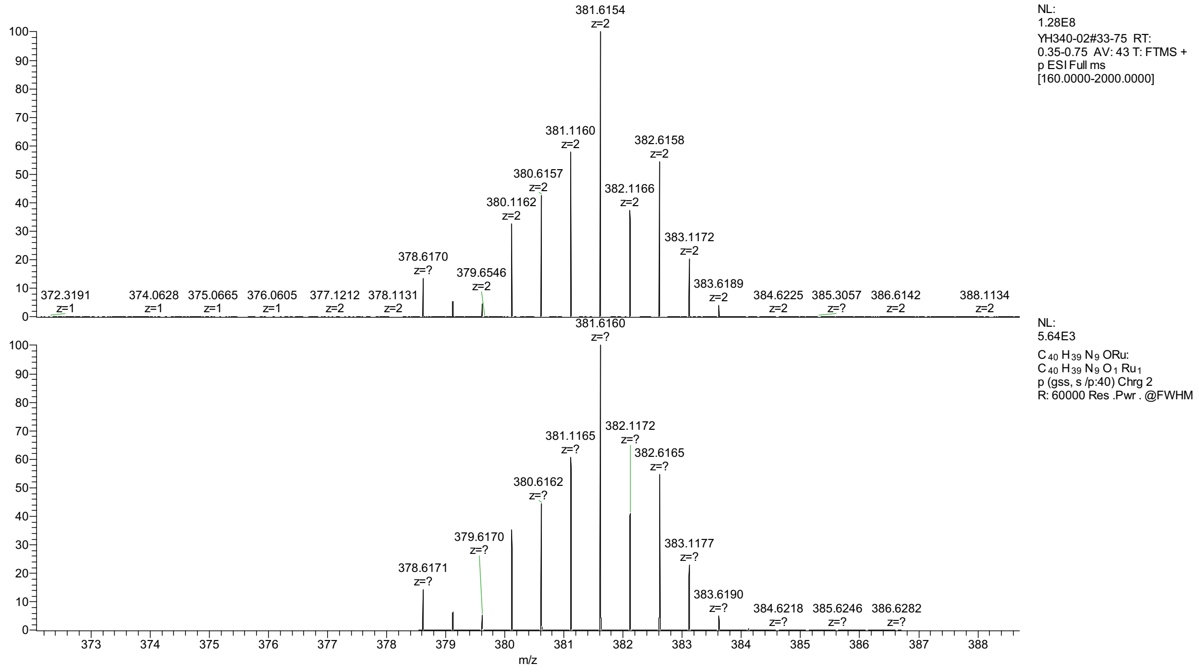

Figure S116. HRMS of [**9**]Cl_2 in MeOH, measured (top) and calculated (bottom).

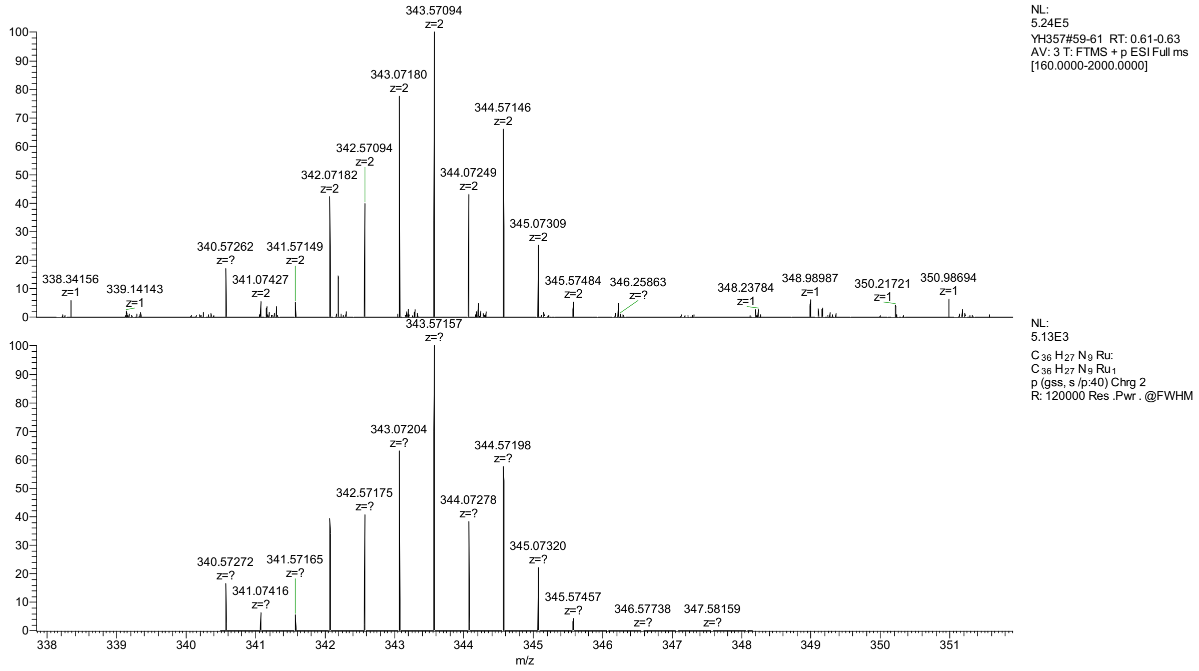

Figure S117. HRMS of [**10**]Cl_2 in MeOH, measured (top) and calculated (bottom).

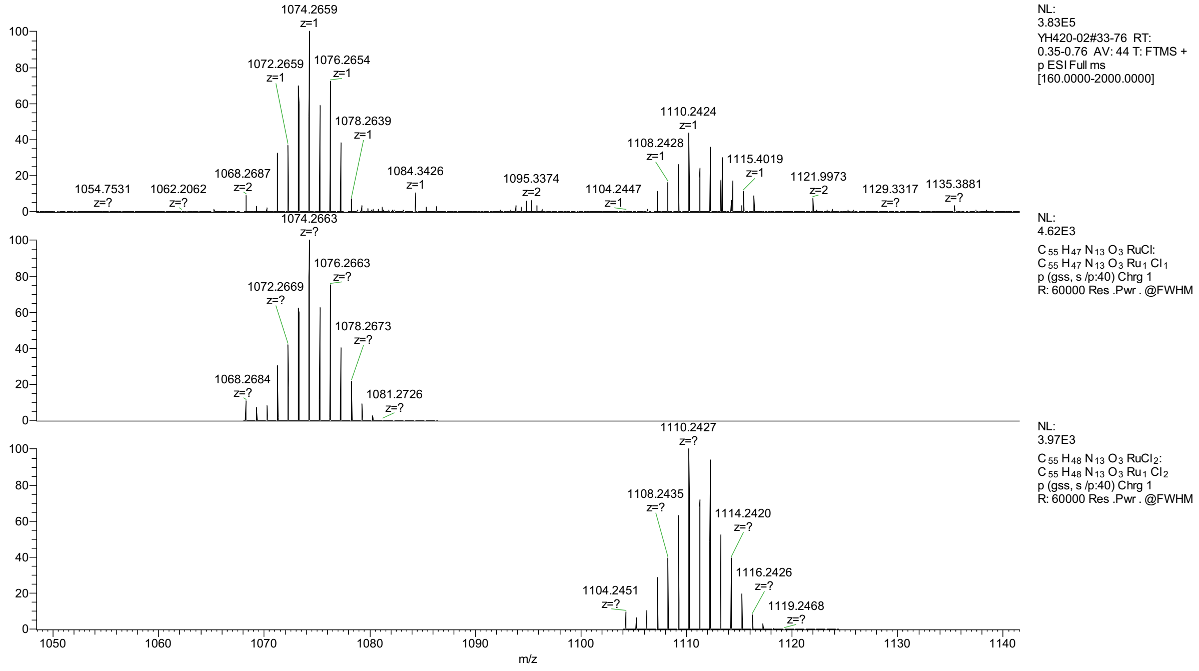

Figure S118. HRMS of [**11**]Cl_2 in MeOH, measured (top) and calculated (bottom).

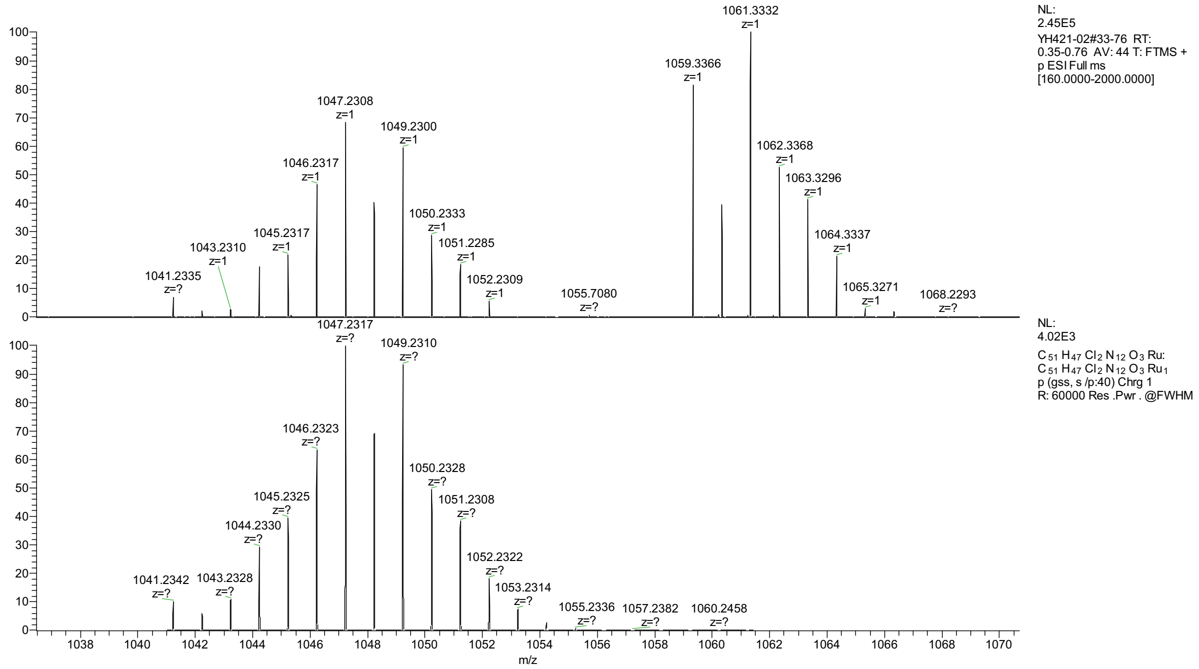

Figure S119. HRMS of [**12**]Cl_2 in MeOH, measured (top) and calculated (bottom).

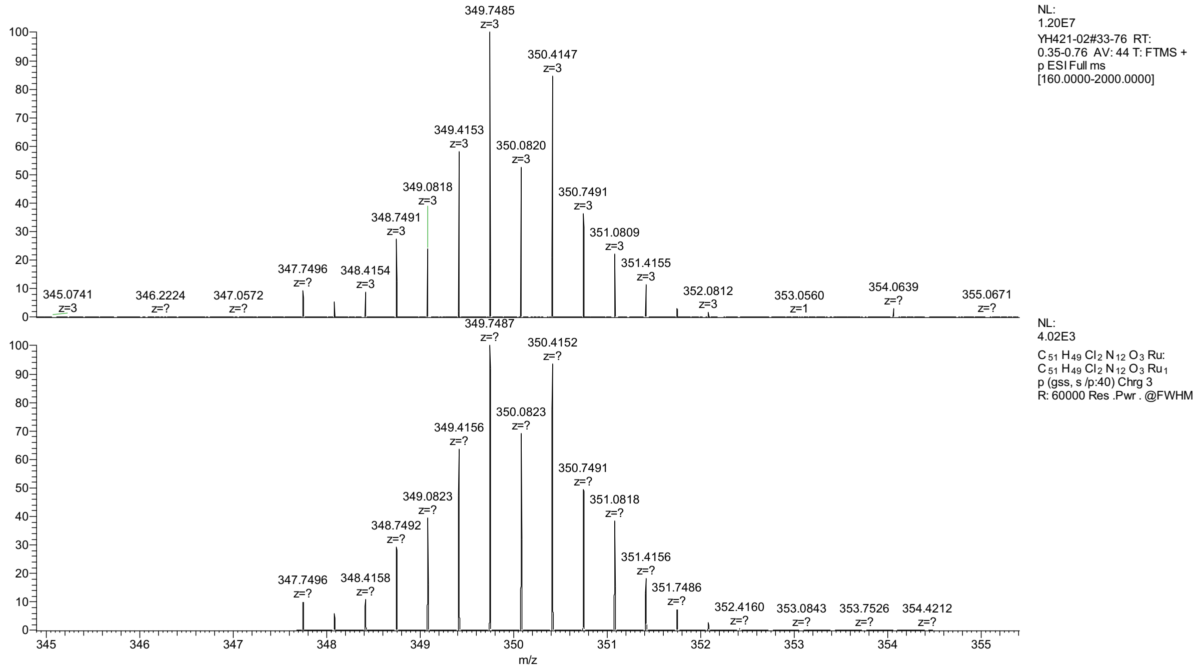

Figure S120. HRMS of [**12**]Cl_2 in MeOH, measured (top) and calculated (bottom).

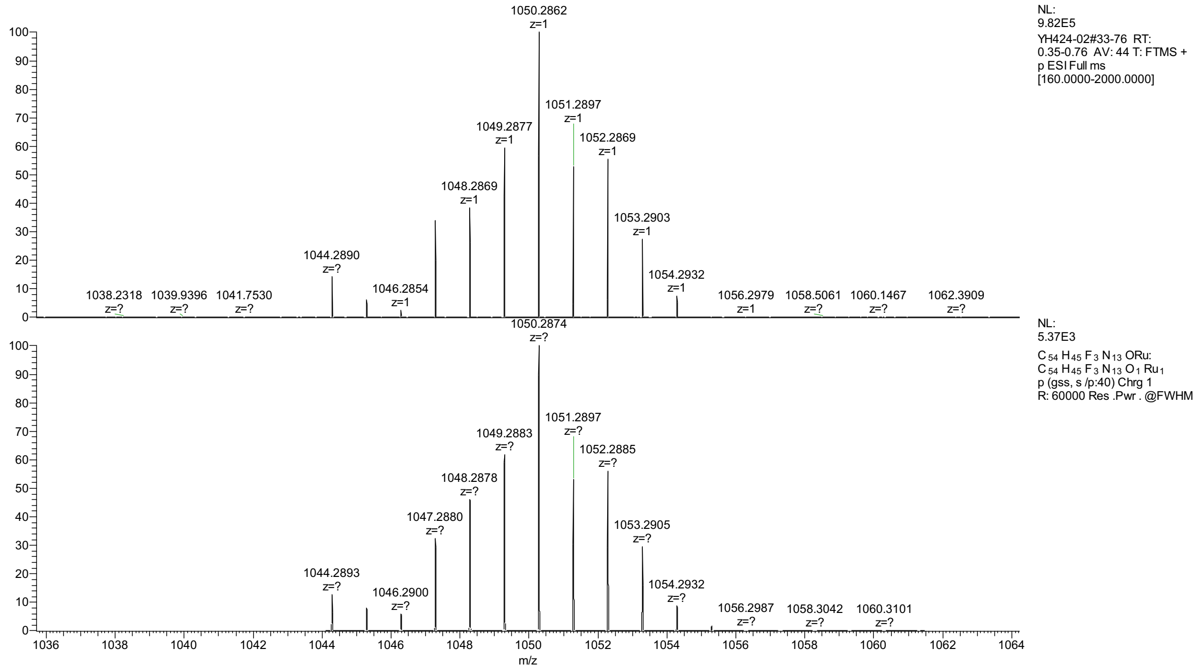

Figure S121. HRMS of [**13**]Cl_2 in MeOH, measured (top) and calculated (bottom).

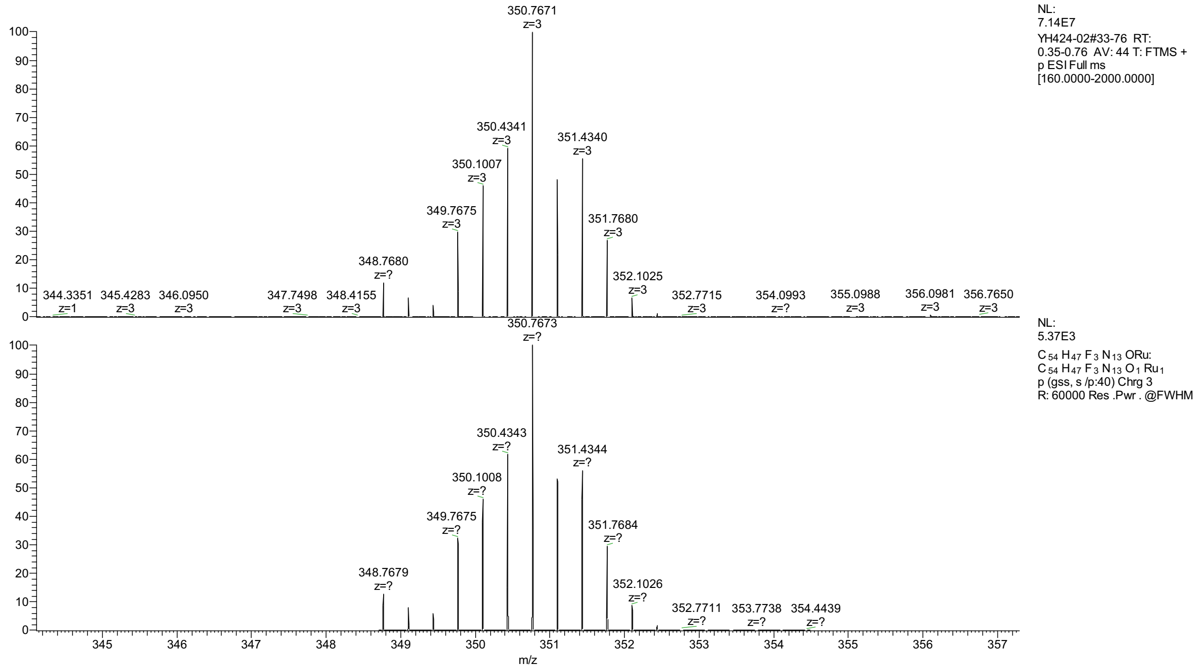

Figure S122. HRMS of [**13**]Cl_2 in MeOH, measured (top) and calculated (bottom).

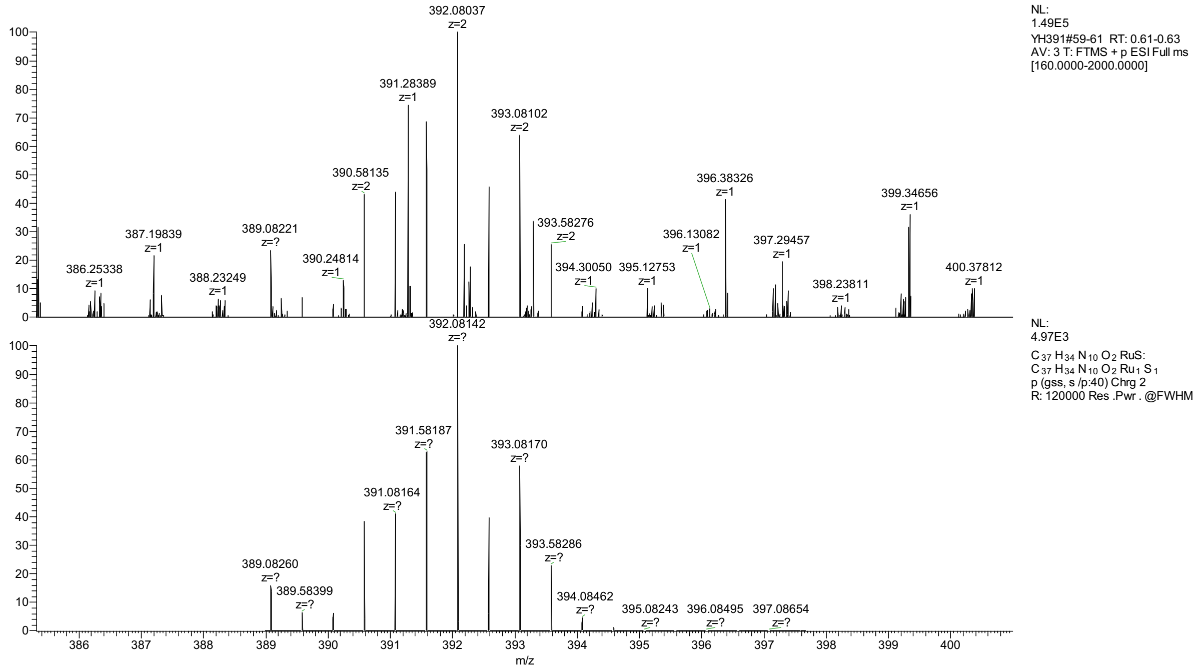

Figure S123. HRMS of [**14**](PF_6)$_2$ in MeOH, measured (top) and calculated (bottom).

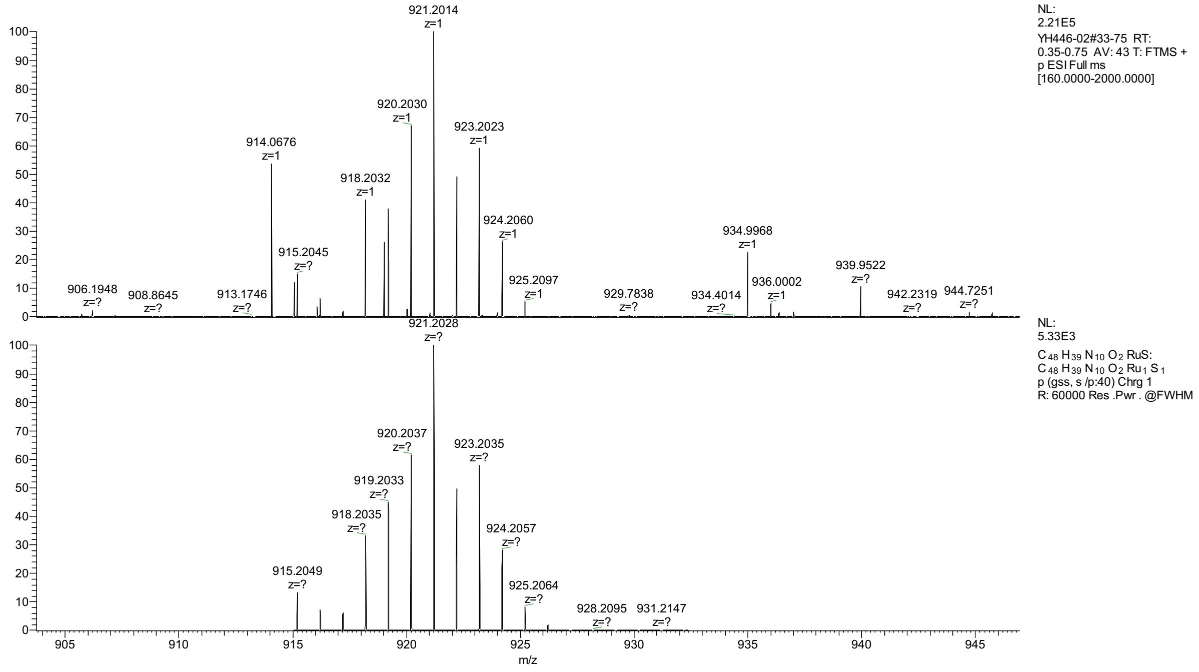

Figure S124. HRMS of [**15**](PF_6)$_2$ in MeOH, measured (top) and calculated (bottom).

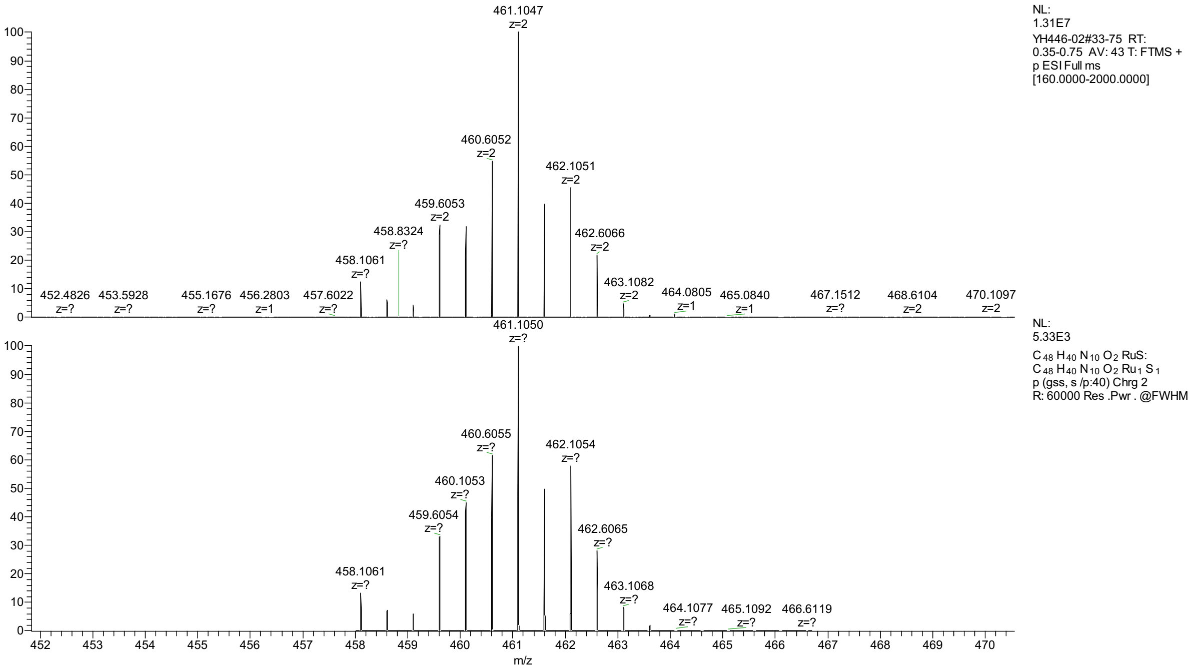

Figure S125. HRMS of [**15**](PF_6)$_2$ in MeOH, measured (top) and calculated (bottom).

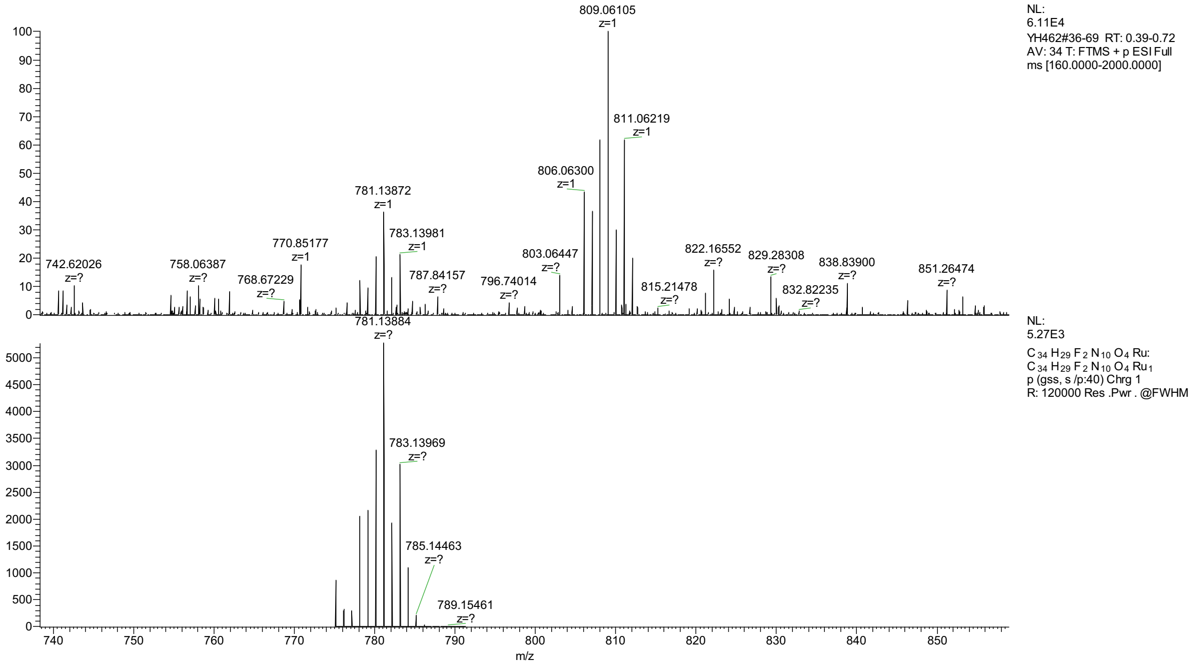

Figure S126. HRMS of [**16**]Cl_2 in MeOH, measured (top) and calculated (bottom).

1.6 Single crystal X-ray crystallography

[4]PF₆

All reflection intensities were measured at 253(2) K using a SuperNova diffractometer (equipped with Atlas detector) with Mo *K*α radiation (λ = 0.71073 Å) under the program CrysAlisPro (Version CrysAlisPro 1.171.42.49, Rigaku OD, 2022). The same program was used to refine the cell dimensions and for data reduction. The structure was solved with the program SHELXS-2018/3 (Sheldrick, 2018) and was refined on F^2 with SHELXL-2018/3 (Sheldrick, 2018).[9] Numerical absorption correction based on gaussian integration over a multifaceted crystal model was performed using CrysAlisPro. The temperature of the data collection was controlled using the system Cryojet (manufactured by Oxford Instruments). The H atoms were placed at calculated positions (unless otherwise specified) using the instruction AFIX 43 with isotropic displacement parameters having values 1.2 U_{eq} of the attached C atoms. The H atoms attached to N2 and N6 were found from difference Fourier maps, and their coordinates were refined pseudofreely using the DFIX instruction in order to keep the N–H distances within an acceptable range.

The structure exhibits partial disorder. One PF_6^- (P1→P6) counterion is disordered over two orientations, with the major component refining to an occupancy of 0.709(7). Additionally, the asymmetric unit includes a partially occupied and disordered HPF_6 moiety (an excess of HPF_6 was added to favor crystallization) with refined occupancies of 0.321(8) and 0.351(8) for the two components. The position of the associated hydrogen atom could not be determined with certainty. A small amount of disordered solvent is also present in the asymmetric unit; its contribution to the diffraction data was removed using the SQUEEZE procedure implemented in Platon (Spek, 2009).[10]

Computer programs: *CrysAlis PRO* 1.171.42.49 (Rigaku OD, 2022), *SHELXS2018*/3 (Sheldrick, 2018), *SHELXL2018*/3 (Sheldrick, 2018), *SHELXTL* v6.10 (Sheldrick, 2008).[9]

Table S1. Selected bond lengths and angles for [**4**]PF_6.

**Bonds, Å**		**Angles, °**	
Ru1–Cl1	2.3158(9)	Cl1–Ru1–N1	86.44(8)
Ru1–N1	2.067(3)	Cl1–Ru1–N3	96.17(9)
Ru1–N3	1.999(3)	Cl1–Ru1–N4	84.81(9)
Ru1–N4	1.970(3)	Cl1–Ru1–N5	94.34(8)
Ru1–N5	2.019(3)	N1–Ru1–N3	92.98(11)
Ru1–N7	2.103(3)	N1–Ru1–N5	100.28(11)
		N1–Ru1–N7	89.29(11)
		N3–Ru1–N4	81.13(12)
		N3–Ru1–N7	91.83(11)
		N4–Ru1–N5	77.29(12)
		N4–Ru1–N7	100.28(11)
		N5–Ru1–N7	79.83(11)

Table S2. Crystallographic data summary for [**4**]PF_6.

**Crystal data**	
Chemical formula	$C_{25}H_{19}ClN_7Ru \cdot 1.672(F_6P)$
M_r	796.43
Crystal system, space group	Monoclinic, *I*2/*a*
Temperature (K)	253
a, *b*, *c* (Å)	22.230 (1), 12.1936 (4), 22.8833 (12)
β (°)	109.167 (5)
V (Å^3)	5859.0 (5)
Z	8
Radiation type	Mo *K*α
μ (mm^{-1})	0.81
Crystal size (mm)	0.31 × 0.10 × 0.05
**Data collection**	
Diffractometer	SuperNova, Dual, Cu at zero, Atlas
Absorption correction	Gaussian<br>*CrysAlis PRO* 1.171.42.49 (Rigaku Oxford Diffraction, 2022) Numerical absorption correction based on gaussian integration over a multifaceted crystal model Empirical absorption correction using spherical harmonics, implemented in SCALE3 ABSPACK scaling algorithm.
T_{min}, T_{max}	0.740, 1.000
No. of measured, independent and observed [$I > 2\sigma(I)$] reflections	31230, 5746, 4650
R_{int}	0.065
$(\sin \theta/\lambda)_{max}$ (Å^{-1})	0.617
**Refinement**	
$R[F^2 > 2\sigma(F^2)]$, $wR(F^2)$, S	0.040, 0.105, 1.07
No. of reflections	5746
No. of parameters	563
No. of restraints	524
H-atom treatment	H atoms treated by a mixture of independent and constrained refinement
	$w = 1/[\sigma^2(F_o^2) + (0.0446P)^2 + 12.0267P]$<br>where $P = (F_o^2 + 2F_c^2)/3$
$\Delta\rho_{max}$, $\Delta\rho_{min}$ (e Å^{-3})	0.78, -0.73

[7](BF$_4$)$_2$

All reflection intensities were measured at 110(2) K using a SuperNova diffractometer (equipped with Atlas detector) with Mo *K*α radiation (λ = 0.71073 Å) under the program CrysAlisPro (Version CrysAlisPro 1.171.42.49, Rigaku OD, 2022). The same program was used to refine the cell dimensions and for data reduction. The structure was solved with the program SHELXS-2018/3 (Sheldrick, 2018) and was refined on F^2 with SHELXL-2018/3 (Sheldrick, 2018).[9] Numerical absorption correction based on gaussian integration over a multifaceted crystal model was performed using CrysAlisPro. The temperature of the data collection was controlled using the system Cryojet (manufactured by Oxford Instruments). The H atoms were placed at calculated positions (unless otherwise specified) using the instructions AFIX 23, AFIX 43 or AFIX 137 with isotropic displacement parameters having values 1.2 or 1.5 U_{eq} of the attached C or N atoms. The H atoms attached to N9, N10, O1W and O2W were found from difference Fourier maps, and their coordinates were refined pseudofreely using the DFIX instructions in order to keep the N–H, O–H bond and H…H distances within some acceptable ranges.

The structure is party disordered. The moiety Ru1→C25 and the two BF_4^- counterions are disordered over either two or three orientations, and all occupancy factors of the major/minor components of the disorder can be retrieved from the final .cif file. The asymmetric unit also contains two lattice water solvent molecules (O1W is fully occupied but O2W is only partially occupied, and the occupancy factor refines to 0.624(11)).

Computer programs: *CrysAlis PRO* 1.171.42.49 (Rigaku OD, 2022), *SHELXS2018*/3 (Sheldrick, 2018), *SHELXL2018*/3 (Sheldrick, 2018), *SHELXTL* v6.10 (Sheldrick, 2008).[9]

Table S3. Selected bond lengths and angles for [**7**](BF$_4$)$_2$.

**Bonds, Å**		**Angles, °**	
Ru1–N1	2.098(4)	N1–Ru1–N3	92.55(16)
Ru1–N3	2.027(4)	N1–Ru1–N5	110.42(17)
Ru1–N4	1.961(3)	N1–Ru1–N7	81.09(17)
Ru1–N5	2.055(4	N1–Ru1–N8	87.98(15)
Ru1–N7	2.119(4)	N3–Ru1–N4	80.46(16)
Ru1–N8	2.114(4)	N3–Ru1–N7	95.27(18)
		N3–Ru1–N8	92.96(17)
		N4–Ru1–N5	77.46(17)
		N4–Ru1–N7	106.77(19)
		N4–Ru1–N8	85.04(17)
		N5–Ru1–N7	82.9(2)
		N5–Ru1–N8	93.76(18)

Table S4. Crystallographic data summary for [**7**]$(BF_4)_2$.

**Crystal data**	
Chemical formula	$C_{48}H_{44}N_{10}O_3RuS\cdot 2(BF_4)\cdot 1.624(H_2O)$
M_r	1144.88
Crystal system, space group	Triclinic, *P*-1
Temperature (K)	110
a, *b*, *c* (Å)	13.0211 (4), 13.4609 (5), 14.2536 (2)
α, β, γ (°)	80.732 (2), 82.5504 (19), 82.385 (2)
V ($Å^3$)	2428.93 (12)
Z	2
Radiation type	Mo *K*α
μ (mm^{-1})	0.46
Crystal size (mm)	0.33 × 0.24 × 0.20
**Data collection**	
Diffractometer	SuperNova, Dual, Cu at zero, Atlas
Absorption correction	Gaussian<br>*CrysAlis PRO* 1.171.42.49 (Rigaku Oxford Diffraction, 2022) Numerical absorption correction based on gaussian integration over a multifaceted crystal model Empirical absorption correction using spherical harmonics, implemented in SCALE3 ABSPACK scaling algorithm.
T_{min}, T_{max}	0.465, 1.000
No. of measured, independent and observed [$I > 2\sigma(I)$] reflections	51058, 9543, 8850
R_{int}	0.037
$(\sin \theta/\lambda)_{max}$ ($Å^{-1}$)	0.617
**Refinement**	
$R[F^2 > 2\sigma(F^2)]$, $wR(F^2)$, S	0.047, 0.110, 1.20
No. of reflections	9543
No. of parameters	1123
No. of restraints	1935
H-atom treatment	H atoms treated by a mixture of independent and constrained refinement
$\Delta\rho_{max}$, $\Delta\rho_{min}$ (e $Å^{-3}$)	0.93, -0.62

*[**8**]Cl₂*

All reflection intensities were measured at 110.00(10) K using a Rigaku XtaLAB Synergy R (equipped with a rotating-anode X-ray source and HyPix-6000HE detector) with Cu *K*α radiation (λ = 1.54178 Å) under the program CrysAlisPro (Version CrysAlisPro 1.171.42.49, Rigaku OD, 2022). The same program was used to refine the cell dimensions and for data reduction. The structure was solved with the program SHELXT-2018/2 (Sheldrick, 2018) and was refined on F^2 with SHELXL-2019/3 (Sheldrick, 2018).[9] Analytical numeric absorption correction using a multifaceted crystal was applied using CrysAlisPro. The temperature of the data collection was controlled using the system Cryostream 1000 from Oxford Cryosystems. The H atoms were placed at calculated positions (unless otherwise specified) using the instructions AFIX 23 or AFIX 43 with isotropic displacement parameters having values 1.2 U_{eq} of the attached C atoms. The H atoms attached to N2 and N6 were found from difference Fourier maps, and their coordinates were refined pseudofreely using the DFIX instruction in order to keep the N−H bond distances within an acceptable range.

The asymmetric unit contains one ordered Ru complex, two Cl^- counterions, and some amount of partially occupied and very disordered lattice solvent molecules (most likely DMF, THF). The latter contribution was removed from the final refinement using the SQUEEZE procedure in Platon (Spek, 2009).[10]

Computer programs: *CrysAlis PRO* 1.171.42.49 (Rigaku OD, 2022), *SHELXT2018*/2 (Sheldrick, 2018), *SHELXL2019*/3 (Sheldrick, 2018), *SHELXTL* v6.10 (Sheldrick, 2008).[9]

Table S5. Selected bond lengths and angles for [**8**]Cl_2.

**Bonds, Å**		**Angles, °**	
Ru1–N1	2.090(2)	N1–Ru1–N3	91.74(8)
Ru1–N3	2.0260(19)	N1–Ru1–N5	110.93(8)
Ru1–N4	1.9615(19)	N1–Ru1–N7	83.81(8)
Ru1–N5	2.0662(19)	N1–Ru1–N8	88.23(8)
Ru1–N7	2.104(2)	N3–Ru1–N4	81.07(8)
Ru1–N8	2.106(2)	N3–Ru1–N7	95.36(8)
		N3–Ru1–N8	91.66(8)
		N4–Ru1–N5	77.46(8)
		N4–Ru1–N7	106.18(8)
		N4–Ru1–N8	82.55(8)
		N5–Ru1–N7	81.92(8)
		N5–Ru1–N8	94.66(8)

Table S6. Crystallographic data summary for [**8**]Cl_2.

**Crystal data**	
Chemical formula	$C_{47}H_{35}ClN_{10}ORu·2(Cl)$
M_r	963.27
Crystal system, space group	Monoclinic, *I*2/*a*
Temperature (K)	110
a, *b*, *c* (Å)	25.5407 (5), 13.4994 (2), 40.0473 (7)
β (°)	106.2548 (19)
V (Å^3)	13255.7 (4)
Z	8
Radiation type	Cu *K*α
μ (mm^{-1})	3.29
Crystal size (mm)	0.12 × 0.05 × 0.04
**Data collection**	
Diffractometer	XtaLAB Synergy R, HyPix
Absorption correction	Analytical<br>*CrysAlis PRO* 1.171.42.95a (Rigaku Oxford Diffraction, 2023)<br>Analytical numeric absorption correction using a multifaceted crystal model based on expressions derived by R.C. Clark & J.S. Reid. (Clark, R. C. & Reid, J. S. (1995). Acta Cryst. A51, 887-897)<br>Empirical absorption correction using spherical harmonics, implemented in SCALE3 ABSPACK scaling algorithm.[11]
T_{min}, T_{max}	0.814, 0.930
No. of measured, independent and observed [$I > 2\sigma(I)$] reflections	86469, 13014, 12013
R_{int}	0.047
$(\sin \theta/\lambda)_{max}$ (Å^{-1})	0.616
**Refinement**	
$R[F^2 > 2\sigma(F^2)]$, $wR(F^2)$, S	0.040, 0.106, 1.07
No. of reflections	13014
No. of parameters	565
No. of restraints	2
H-atom treatment	H atoms treated by a mixture of independent and constrained refinement
	$w = 1/[\sigma^2(F_o^2) + (0.0464P)^2 + 30.680P]$<br>where $P = (F_o^2 + 2F_c^2)/3$
$\Delta\rho_{max}$, $\Delta\rho_{min}$ (e Å^{-3})	1.22, -0.67

*[**9**](PF$_6$)$_2$*

All reflection intensities were measured at 110(2) K using a SuperNova diffractometer (equipped with Atlas detector) with Mo *K*α radiation (λ = 0.71073 Å) under the program CrysAlisPro (Version CrysAlisPro 1.171.42.49, Rigaku OD, 2022). The same program was used to refine the cell dimensions and for data reduction. The structure was solved with the program SHELXS-2018/3 (Sheldrick, 2018) and was refined on F^2 with SHELXL-2018/3 (Sheldrick, 2018).[9] Empirical absorption correction using spherical harmonics was performed using CrysAlisPro. The temperature of the data collection was controlled using the system Cryojet (manufactured by Oxford Instruments). The H atoms were placed at calculated positions (unless otherwise specified) using the instructions AFIX 13, AFIX 23, AFIX 43 or AFIX 137 with isotropic displacement parameters having values 1.2 or 1.5 U_{eq} of the attached C atoms. The H atoms attached to N2 and N6 were found from difference Fourier maps, and their coordinates were refined pseudofreely using the DFIX instruction in order to keep the N–H bond distances within an acceptable range.

The structure is ordered. The absolute configuration has been established by anomalous-dispersion effects in diffraction measurements on the crystal, and the Flack and Hooft parameters refine to -0.02(2) and -0.018(19), respectively.

Computer programs: *CrysAlis PRO* 1.171.42.49 (Rigaku OD, 2022), *SHELXS2018*/3 (Sheldrick, 2018), *SHELXL2018*/3 (Sheldrick, 2018), *SHELXTL* v6.10 (Sheldrick, 2008).[9]

Table S7. Selected bond lengths and angles for [**9**](PF$_6$)$_2$.

**Bonds, Å**		**Angles, °**	
Ru1–N1	2.100(5)	N1–Ru1–N3	92.4(2)
Ru1–N3	2.018(5)	N1–Ru1–N5	110.2(2)
Ru1–N4	1.955(5)	N1–Ru1–N7	84.5(2)
Ru1–N5	2.061(5)	N1–Ru1–N8	87.3(2)
Ru1–N7	2.100(6)	N3–Ru1–N4	80.9(2)
Ru1–N8	2.072(6)	N3–Ru1–N7	94.8(2)
		N3–Ru1–N8	91.8(2)
		N4–Ru1–N5	77.6(2)
		N4–Ru1–N7	105.1(2)
		N4–Ru1–N8	83.8(2)
		N5–Ru1–N7	82.4(2)
		N5–Ru1–N8	94.6(2)

Table S8. Crystallographic data summary for [**9**]$(PF_6)_2$.

**Crystal data**	
Chemical formula	$C_{40}H_{39}N_9ORu \cdot 2(F_6P) \cdot C_3H_6O$
M_r	1110.89
Crystal system, space group	Triclinic, *P*1
Temperature (K)	110
a, *b*, *c* (Å)	9.7515 (4), 11.2867 (6), 11.9061 (6)
α, β, γ (°)	66.599 (5), 82.851 (4), 71.056 (4)
V (Å^3)	1137.47 (11)
Z	1
Radiation type	Mo *K*α
μ (mm^{-1})	0.51
Crystal size (mm)	0.10 × 0.10 × 0.06
**Data collection**	
Diffractometer	SuperNova, Dual, Cu at zero, Atlas
Absorption correction	Multi-scan<br>*CrysAlis PRO* 1.171.42.49 (Rigaku Oxford Diffraction, 2022)<br>Empirical absorption correction using spherical harmonics, implemented in SCALE3 ABSPACK scaling algorithm.
T_{min}, T_{max}	0.614, 1.000
No. of measured, independent and observed [$I > 2\sigma(I)$] reflections	19120, 8793, 8072
R_{int}	0.065
$(\sin \theta/\lambda)_{max}$ (Å^{-1})	0.617
**Refinement**	
$R[F^2 > 2\sigma(F^2)]$, $wR(F^2)$, S	0.047, 0.084, 1.02
No. of reflections	8793
No. of parameters	630
No. of restraints	5
H-atom treatment	H atoms treated by a mixture of independent and constrained refinement
$\Delta\rho_{max}$, $\Delta\rho_{min}$ (e Å^{-3})	0.79, -0.42
Absolute structure	Flack x determined using 3110 quotients [(I+)-(I-)]/[(I+)+(I-)] (Parsons, Flack and Wagner, Acta Cryst. B69 (2013) 249-259).
Absolute structure parameter	-0.02 (2)

*[**9**](BF$_4$)$_2$*

All reflection intensities were measured at 110(2) K using a SuperNova diffractometer (equipped with Atlas detector) with Mo *K*α radiation (λ = 0.71073 Å) under the program CrysAlisPro (Version CrysAlisPro 1.171.42.49, Rigaku OD, 2022). The same program was used to refine the cell dimensions and for data reduction. The structure was solved with the program SHELXS-2018/3 (Sheldrick, 2018) and was refined on F^2 with SHELXL-2018/3 (Sheldrick, 2018).[9] Analytical numeric absorption correction using a multifaceted crystal model was performed using CrysAlisPro. The temperature of the data collection was controlled using the system Cryojet (manufactured by Oxford Instruments). The H atoms were placed at calculated positions (unless otherwise specified) using the instructions AFIX 13, AFIX 23, AFIX 43 or AFIX 137 with isotropic displacement parameters having values 1.2 or 1.5 U_{eq} of the attached C atoms. The H atoms attached to N2 and N6 were found from difference Fourier maps, and their coordinates were refined pseudofreely using the DFIX instruction in order to keep the N–H bond distances within an acceptable range.

The structure is partly disordered. One of the two BF_4^- counterions and the lattice MeOH solvent molecule were found to be disordered over two orientations, and the occupancy factors of the major components of the disorder refine to 0.838(9) and 0.694(10). The absolute configuration has been established by anomalous-dispersion effects in diffraction measurements on the crystal, and the Flack and Hooft parameters refine to -0.020(9) and -0.015(8), respectively.

Computer programs: *CrysAlis PRO* 1.171.42.49 (Rigaku OD, 2022), *SHELXS2018*/3 (Sheldrick, 2018), *SHELXL2018*/3 (Sheldrick, 2018), *SHELXTL* v6.10 (Sheldrick, 2008).[9]

Table S9. Selected bond lengths and angles for [**9**](BF$_4$)$_2$.

**Bonds, Å**		**Angles, °**	
Ru1–N1	2.095(3)	N1–Ru1–N3	92.00(13)
Ru1–N3	2.021(3)	N1–Ru1–N5	109.73(13)
Ru1–N4	1.946(4)	N1–Ru1–N7	86.91(14)
Ru1–N5	2.049(3)	N1–Ru1–N8	87.73(14)
Ru1–N7	2.087(4)	N3–Ru1–N4	81.45(15)
Ru1–N8	2.095(4)	N3–Ru1–N7	93.55(13)
		N3–Ru1–N8	91.99(13)
		N4–Ru1–N5	77.89(14)
		N4–Ru1–N7	101.97(14)
		N4–Ru1–N8	83.97(14)
		N5–Ru1–N7	82.08(13)
		N5–Ru1–N8	94.71(13)

Table S10. Crystallographic data summary for [**9**](BF_4)$_2$.

**Crystal data**	
Chemical formula	$C_{40}H_{39}N_9ORu·2(BF_4)·CH_4O$
M_r	968.53
Crystal system, space group	Triclinic, *P*1
Temperature (K)	110
a, *b*, *c* (Å)	9.6576 (2), 10.4002 (2), 12.2969 (3)
α, β, γ (°)	69.155 (2), 77.481 (2), 64.622 (2)
V (Å^3)	1039.86 (4)
Z	1
Radiation type	Mo *K*α
μ (mm^{-1})	0.46
Crystal size (mm)	0.34 × 0.21 × 0.10
**Data collection**	
Diffractometer	SuperNova, Dual, Cu at zero, Atlas
Absorption correction	Analytical<br>*CrysAlis PRO* 1.171.42.49 (Rigaku Oxford Diffraction, 2022) Analytical numeric absorption correction using a multifaceted crystal model based on expressions derived by R.C. Clark & J.S. Reid. (Clark, R. C. & Reid, J. S. (1995). Acta Cryst. A51, 887-897) Empirical absorption correction using spherical harmonics, implemented in SCALE3 ABSPACK scaling algorithm.[11]
T_{min}, T_{max}	0.903, 0.961
No. of measured, independent and observed [$I > 2\sigma(I)$] reflections	24148, 9463, 9350
R_{int}	0.029
$(\sin \theta/\lambda)_{max}$ (Å^{-1})	0.650
**Refinement**	
$R[F^2 > 2\sigma(F^2)]$, $wR(F^2)$, S	0.029, 0.066, 1.03
No. of reflections	9463
No. of parameters	637
No. of restraints	184
H-atom treatment	H atoms treated by a mixture of independent and constrained refinement
$\Delta\rho_{max}$, $\Delta\rho_{min}$ (e Å^{-3})	0.60, -0.26
Absolute structure	Flack x determined using 4480 quotients [(I+)-(I-)]/[(I+)+(I-)] (Parsons, Flack and Wagner, Acta Cryst. B69 (2013) 249-259).
Absolute structure parameter	-0.020 (9)

*[**10**]Cl$_2$*

All reflection intensities were measured at 110.00(10) K using a Rigaku XtaLAB Synergy R (equipped with a rotating-anode X-ray source and HyPix-6000HE detector) with Cu *K*α radiation (λ = 1.54178 Å) under the program CrysAlisPro (Version CrysAlisPro 1.171.42.49, Rigaku OD, 2022). The same program was used to refine the cell dimensions and for data reduction. The structure was solved with the program SHELXS-2018/3 (Sheldrick, 2018) and was refined on F^2 with SHELXL-2018/3 (Sheldrick, 2018).[9] Analytical numeric absorption correction using a multifaceted crystal was applied using CrysAlisPro. Crystals were initially deposited on a microscope slide in some Parabar 10312 and were quickly cooled under a cold N_2(g) stream in order to prevent any decomposition of the crystals. One single crystal was then quickly picked and mounted on the diffractometer while being flash-cooled at 110 K. The temperature of the data collection was controlled using the system Cryostream 1000 from Oxford Cryosystems. The H atoms were placed at calculated positions using the instructions AFIX 43 with isotropic displacement parameters having values 1.2 U_{eq} of the attached C or N atoms.

The structure is disordered. The Ru complex and one of the two Cl^- counterions are disordered over two and three orientations, respectively. All occupancy factors can be retrieved from the final .cif file. The crystal lattice also contains some amount of very disordered lattice solvent molecules, and their contribution was removed from the final refinement using the SQUEEZE procedure in Platon (Spek, 2009).[10]

Computer programs: *CrysAlis PRO* 1.171.42.49 (Rigaku OD, 2022), *SHELXS2018*/3 (Sheldrick, 2018), *SHELXL2018*/3 (Sheldrick, 2018), *SHELXTL* v6.10 (Sheldrick, 2008).[9]

Table S11. Selected bond lengths and angles for [**10**]Cl$_2$.

**Bonds, Å**		**Angles, °**	
Ru1–N1	2.099(7)	N1–Ru1–N3	95.8(3)
Ru1–N3	2.017(6)	N1–Ru1–N5	107.9(3)
Ru1–N4	1.973(6)	N1–Ru1–N7	88.5(3)
Ru1–N5	2.033(7)	N1–Ru1–N8	84.0(3)
Ru1–N7	2.047(8)	N3–Ru1–N4	79.3(3)
Ru1–N8	2.118(7)	N3–Ru1–N7	97.1(4)
		N3–Ru1–N8	94.4(4)
		N4–Ru1–N5	78.3(3)
		N4–Ru1–N7	105.0(4)
		N4–Ru1–N8	83.3(4)
		N5–Ru1–N7	81.1(3)
		N5–Ru1–N8	90.9(4)

Table S12. Crystallographic data summary for [**10**]Cl_2.

**Crystal data**	
Chemical formula	$C_{36}H_{27}N_9Ru·2(Cl)$
M_r	757.63
Crystal system, space group	Triclinic, *P*-1
Temperature (K)	110
a, *b*, *c* (Å)	9.5729 (5), 17.3947 (15), 17.8473 (10)
α, β, γ (°)	63.221 (7), 75.085 (5), 83.549 (5)
V ($Å^3$)	2563.7 (3)
Z	2
Radiation type	Cu *K*α
μ (mm^{-1})	3.65
Crystal size (mm)	0.30 × 0.15 × 0.02
**Data collection**	
Diffractometer	XtaLAB Synergy R, HyPix
Absorption correction	Analytical<br>*CrysAlis PRO* 1.171.42.80a (Rigaku Oxford Diffraction, 2023)<br>Analytical numeric absorption correction using a multifaceted crystal model based on expressions derived by R.C. Clark & J.S. Reid. (Clark, R. C. & Reid, J. S. (1995). Acta Cryst. A51, 887-897)<br>Empirical absorption correction using spherical harmonics, implemented in SCALE3 ABSPACK scaling algorithm.[11]
T_{min}, T_{max}	0.560, 0.915
No. of measured, independent and observed [$I > 2\sigma(I)$] reflections	38374, 10035, 7703
R_{int}	0.035
$(\sin \theta/\lambda)_{max}$ ($Å^{-1}$)	0.616
**Refinement**	
$R[F^2 > 2\sigma(F^2)]$, $wR(F^2)$, S	0.076, 0.244, 1.06
No. of reflections	10035
No. of parameters	870
No. of restraints	1811
H-atom treatment	H-atom parameters constrained
$\Delta\rho_{max}$, $\Delta\rho_{min}$ (e $Å^{-3}$)	1.35, -0.69

[14](PF$_6$)$_2$

All reflection intensities were measured at 250.00(10) K* using a Rigaku XtaLAB Synergy R (equipped with a rotating-anode X-ray source and HyPix-6000HE detector) with Cu $K\alpha$ radiation (λ = 1.54178 Å) under the program CrysAlisPro (Version CrysAlisPro 1.171.42.49, Rigaku OD, 2022). The same program was used to refine the cell dimensions and for data reduction. The structure was solved with the program SHELXS-2018/3 (Sheldrick, 2018) and was refined on F^2 with SHELXL-2018/3 (Sheldrick, 2018).[9] Analytical numeric absorption correction using a multifaceted crystal was applied using CrysAlisPro. The temperature of the data collection was controlled using the system Cryostream 1000 from Oxford Cryosystems). The H (or D) atoms were placed at calculated positions using the instructions AFIX 23, AFIX 43 or AFIX 137 with isotropic displacement parameters having values 1.2 or 1.5 U_{eq} of the attached C or N atoms. The H atom attached to O1S (partially occupied MeOH) could not be retrieved from difference Fourier map.

The structure exhibits partial disorder. The N1→C5 moiety and both PF_6^- counterions are disordered over two orientations, with the major components refining to occupancy factors of 0.504(13), 0.61(2), and 0.555(6), respectively. The asymmetric unit also contains one site occupied by a mixture of lattice MeOH (occupancy: 0.42(2)) and deuterated acetone (occupancy: 0.58(2)) solvent molecules. In addition, the asymmetric unit includes unresolved, disordered (and likely partially occupied) lattice solvent molecules, whose contribution was removed from the final refinement using the SQUEEZE procedure in Platon (Spek, 2009).[10]

*Data were collected at 250 K due to a malfunction in the Cryostream 1000's shield flow, which caused icing and prevented data collection at 110 K.

Computer programs: *CrysAlis PRO* 1.171.42.49 (Rigaku OD, 2022), *SHELXS2018*/3 (Sheldrick, 2018), *SHELXL2018*/3 (Sheldrick, 2018), *SHELXTL* v6.10 (Sheldrick, 2008).[9]

Table S13. Selected bond lengths and angles for [**14**](PF$_6$)$_2$.

**Bonds, Å**		**Angles, °**	
Ru1–S1	2.3547(12)	S1–Ru1–N1	80.8(5)
Ru1–N1	2.098(18)	S1–Ru1–N3	93.37(12)
Ru1–N3	2.033(4)	S1–Ru1–N4	89.02(11)
Ru1–N4	1.959(4)	S1–Ru1–N5	95.90(11)
Ru1–N5	2.063(4)	N1–Ru1–N3	92.1(7)
Ru1–N7	2.125(5)	N1–Ru1–N5	111.6(7)
		N1–Ru1–N7	86.7(5)
		N3–Ru1–N4	80.70(16)
		N3–Ru1–N7	95.96(19)
		N4–Ru1–N5	77.05(16)
		N4–Ru1–N7	104.53(16)
		N5–Ru1–N7	80.42(19)

Table S14. Crystallographic data summary for [**14**]$(PF_6)_2$.

**Crystal data**	
Chemical formula	$C_{37}H_{34}N_{10}O_2RuS{\cdot}2(F_6P){\cdot}0.58(C_3D_6O){\cdot}0.42(CH_3O)$
M_r	1123.95
Crystal system, space group	Triclinic, *P*-1
Temperature (K)	250
a, *b*, *c* (Å)	9.2424 (5), 14.0980 (5), 20.5291 (8)
α, β, γ (°)	87.006 (3), 79.279 (4), 70.961 (4)
V (Å^3)	2484.4 (2)
Z	2
Radiation type	Cu *K*α
μ (mm^{-1})	4.38
Crystal size (mm)	0.23 × 0.02 × 0.02
**Data collection**	
Diffractometer	XtaLAB Synergy R, HyPix
Absorption correction	Analytical<br>*CrysAlis PRO* 1.171.42.80a (Rigaku Oxford Diffraction, 2023) Analytical numeric absorption correction using a multifaceted crystal model based on expressions derived by R.C. Clark & J.S. Reid. (Clark, R. C. & Reid, J. S. (1995). Acta Cryst. A51, 887-897) Empirical absorption correction using spherical harmonics, implemented in SCALE3 ABSPACK scaling algorithm.[11]
T_{min}, T_{max}	0.555, 0.934
No. of measured, independent and observed [$I > 2\sigma(I)$] reflections	36290, 8852, 6825
R_{int}	0.068
$(\sin \theta/\lambda)_{max}$ (Å^{-1})	0.598
**Refinement**	
$R[F^2 > 2\sigma(F^2)]$, $wR(F^2)$, S	0.060, 0.169, 1.04
No. of reflections	8852
No. of parameters	811
No. of restraints	846
H-atom treatment	H-atom parameters constrained
$\Delta\rho_{max}$, $\Delta\rho_{min}$ (e Å^{-3})	1.83, -0.50

[17](PF₆)(ClO₄)

All reflection intensities were measured at 110.00(10) K using a Rigaku XtaLAB Synergy R (equipped with a rotating-anode X-ray source and HyPix-6000HE detector) with Cu *K*α radiation (λ = 1.54178 Å) under the program CrysAlisPro (Version CrysAlisPro 1.171.42.49, Rigaku OD, 2022). The same program was used to refine the cell dimensions and for data reduction. The structure was solved with the program SHELXT-2018/2 (Sheldrick, 2018) and was refined on F^2 with SHELXL-2019/3 (Sheldrick, 2018).[9] Analytical numeric absorption correction using a multifaceted crystal model was applied using CrysAlisPro. The temperature of the data collection was controlled using the system Cryostream 1000 from Oxford Cryosystems. The H atoms were placed at calculated positions (unless otherwise specified) using the instructions AFIX 43 or AFIX 137 with isotropic displacement parameters having values 1.2 or 1.5 U_{eq} of the attached C atoms. The H atoms attached to N2 and N6 were found from difference Fourier maps, and their coordinates were refined pseudofreely using the DFIX instruction in order to keep the N–H bond distances within an acceptable range.

The structure exhibits partial disorder. One site in the asymmetric unit contains a disordered mixture of ClO_4^- and PF_6^- counterions, with the ClO_4^- component being dominant (occupancy 0.721(7)) and the PF_6^- component minor (occupancy 0.279(7)). The second PF_6^- counterion is fully ordered and occupies its site without disorder.

Computer programs: *CrysAlis PRO* 1.171.42.49 (Rigaku OD, 2022), *SHELXT2018*/2 (Sheldrick, 2018), *SHELXL2019*/3 (Sheldrick, 2018), *SHELXTL* v6.10 (Sheldrick, 2008).[9]

Table S15. Selected bond lengths and angles for [**17**](PF_6)(ClO_4).

**Bonds, Å**		**Angles, °**	
Ru1–N1	2.088(4	N1–Ru1–N3	92.24(18)
Ru1–N3	2.017(4)	N1–Ru1–N5	110.13(17)
Ru1–N4	1.957(5)	N1–Ru1–N7	83.10(18)
Ru1–N5	2.059(4)	N1–Ru1–N8	88.64(18)
Ru1–N7	2.098(5)	N3–Ru1–N4	80.96(18)
Ru1–N8	2.023(5)	N3–Ru1–N7	96.91(18)
		N3–Ru1–N8	92.74(18)
		N4–Ru1–N5	77.32(17)
		N4–Ru1–N7	103.69(18)
		N4–Ru1–N8	85.60(18)
		N5–Ru1–N7	81.05(18)
		N5–Ru1–N8	93.11(18)

Table S16. Crystallographic data summary for [**17**](PF_6)(ClO_4).

**Crystal data**	
Chemical formula	$C_{27}H_{22}N_8Ru\cdot1.279(F_6P)\cdot0.721(ClO_4)\cdot C_2H_3N$
M_r	857.62
Crystal system, space group	Triclinic, *P*-1
Temperature (K)	110
a, *b*, *c* (Å)	8.2958 (6), 11.5374 (8), 18.2115 (13)
α, β, γ (°)	83.572 (6), 78.365 (6), 71.903 (6)
V (Å^3)	1620.5 (2)
Z	2
Radiation type	Cu *K*α
μ (mm^{-1})	5.90
Crystal size (mm)	0.06 × 0.03 × 0.01
**Data collection**	
Diffractometer	XtaLAB Synergy R, HyPix
Absorption correction	Analytical<br>*CrysAlis PRO* 1.171.42.95a (Rigaku Oxford Diffraction, 2023)<br>Analytical numeric absorption correction using a multifaceted crystal model based on expressions derived by R.C. Clark & J.S. Reid. (Clark, R. C. & Reid, J. S. (1995). Acta Cryst. A51, 887-897)<br>Empirical absorption correction using spherical harmonics, implemented in SCALE3 ABSPACK scaling algorithm.[11]
T_{min}, T_{max}	0.772, 0.931
No. of measured, independent and observed [$I > 2\sigma(I)$] reflections	28015, 5792, 5356
R_{int}	0.041
$(\sin \theta/\lambda)_{max}$ (Å^{-1})	0.598
**Refinement**	
$R[F^2 > 2\sigma(F^2)]$, $wR(F^2)$, S	0.057, 0.139, 1.10
No. of reflections	5792
No. of parameters	532
No. of restraints	234
H-atom treatment	H atoms treated by a mixture of independent and constrained refinement
	$w = 1/[\sigma^2(F_o^2) + (0.0448P)^2 + 11.1431P]$<br>where $P = (F_o^2 + 2F_c^2)/3$
$\Delta\rho_{max}$, $\Delta\rho_{min}$ (e Å^{-3})	1.95, -2.21

The structures of the eight complexes have been deposited to the CCDC with the deposition number 2477004-2477011.

2 Photochemistry

2.1 General information

Unless otherwise noted, all the absorption spectra were recorded in acetonitrile (ACN) at 298 K under air using macro spectrophotometer cuvette for 3 mL from Hellma Analytics (lightpath: 1 cm) and Agilent Technologies Cary 60 UV-Vis spectrometer equipped with Cary Single Cell Peltier Accessory for temperature control. The small NewEnergy LEDs to drive the photosubstitution reactions in the cuvette were purchased from Mouser Electronics while more powerful EvoluChem LED spotlights were obtained from HepatoChem. The surface power densities at the irradiation wavelength were measured using Nova Power Meter from Ophir Photonics and the respective photon fluxes were determined by formerly described actinometry protocol.[2] In short, as the ferrioxalate actinometer does not absorb red or far-red light, it was used to measure the photon flux of a blue LED (450 nm). The power of the light beam of that 450 nm LED, as well as that of a 625 and 730 nm LED of interest, were measured using the optical Nova Power Meter, using home-made setup to ensure that the geometries were identical. Finally, the photon flux at 625 or 730 nm was calculated from that at 450 nm corrected by the ratio of optical powers at two wavelengths, and the ratio of photon energies at 625 vs. 450 (or 730 vs. 450) nm. The 625 nm LED had an average power of 11.1 mW/cm^2 and an average photon flux of 1.30×10^{-7} mol/s. The 730 nm LED had an average power of 8.84 mW/cm^2 and an average photon flux of 1.21×10^{-7} mol/s. The modeling of photosubstitution reaction kinetics was done using Glotaran 1.5.1 and R 4.2.2. The calculations of the photosubstitution quantum yields were done by formerly described method.[2] Q_{total} stands for total amount of photons absorbed by compound during photosubstitution reaction. HPLC chromatograms were recorded by Thermo Scientific Dionex UltiMate 3000 UHPLC: gradient elution 10 to 90% v/v ACN in H_2O (+0.1% v/v TFA); UV detector: 272 nm; run time: 23.5 min.

2.2 Molar absorption coefficients

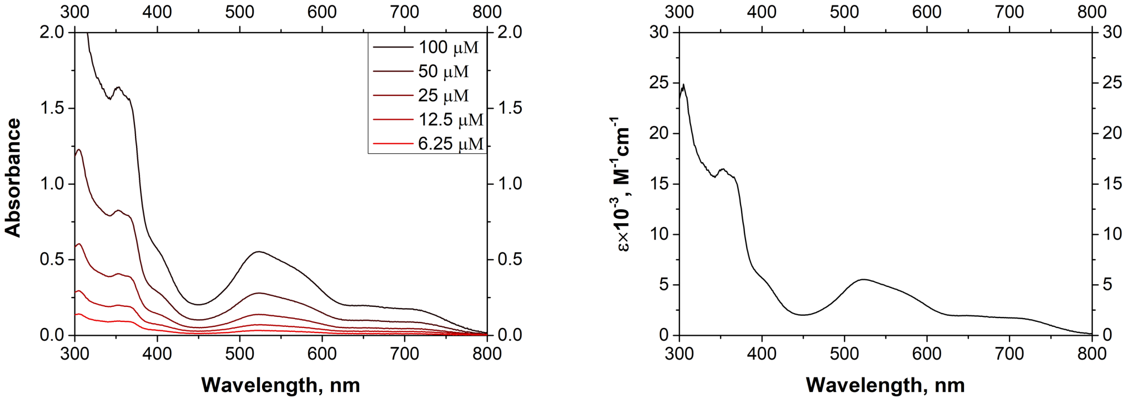

Figure S127. Absorption spectra of [**4**]Cl in ACN at different concentrations (left) and respective molar absorption coefficients (right) calculated as the slopes of A = f(C) linear regressions at every wavelength from 300 to 800 nm.

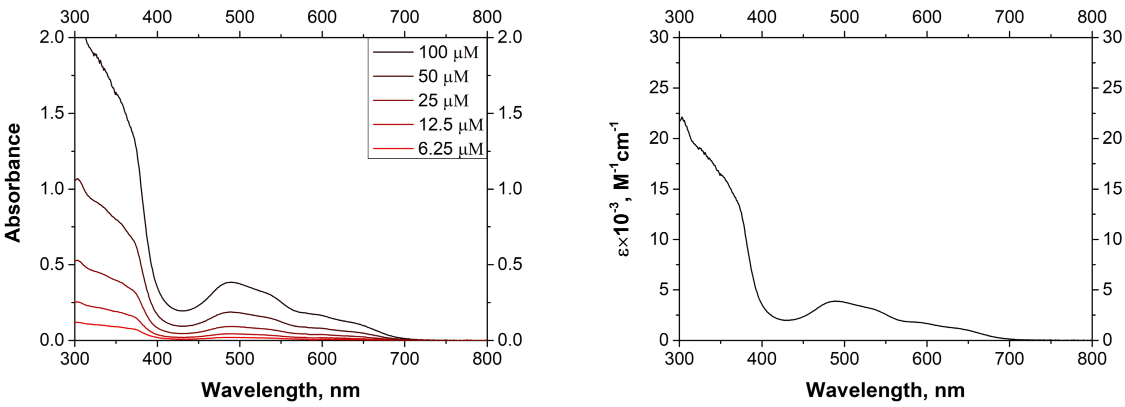

Figure S128. Absorption spectra of [**6**]Cl_2 in ACN at different concentrations (left) and respective molar absorption coefficients (right) calculated as the slopes of A = f(C) linear regressions at every wavelength from 300 to 800 nm.

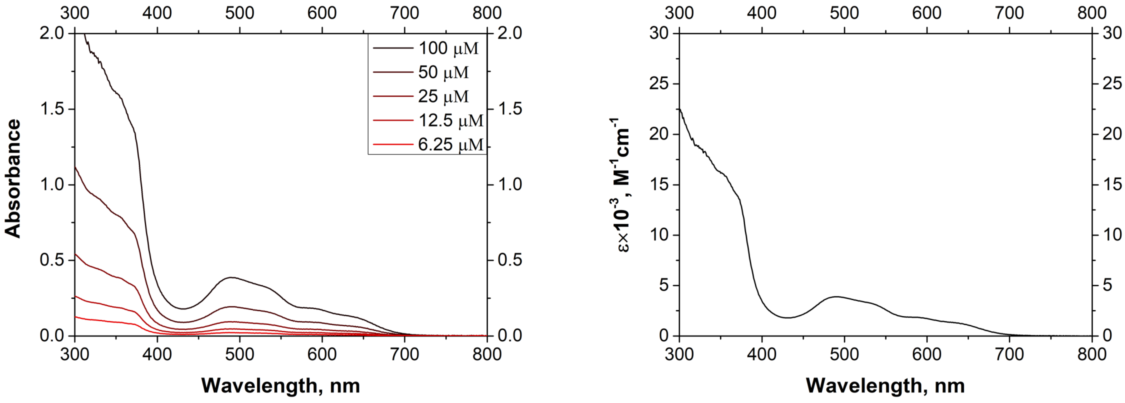

Figure S129. Absorption spectra of [**7**]Cl_2 in ACN at different concentrations (left) and respective molar absorption coefficients (right) calculated as the slopes of A = f(C) linear regressions at every wavelength from 300 to 800 nm.

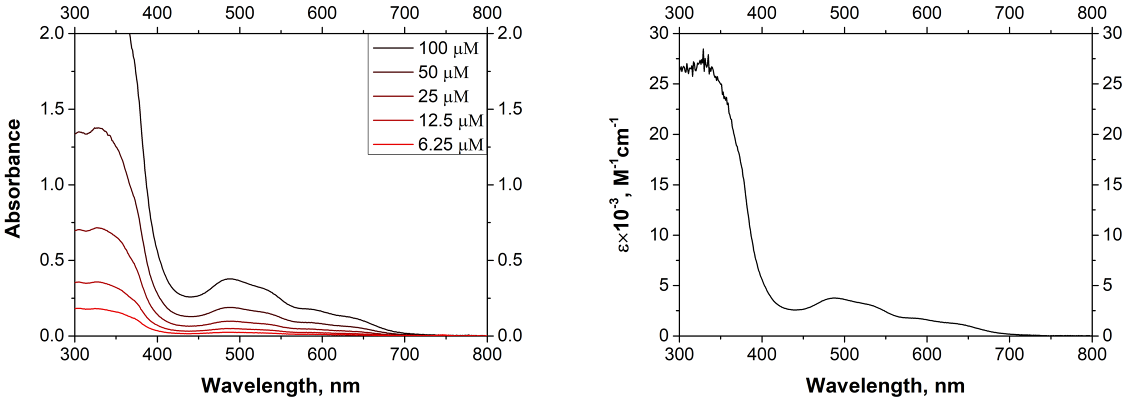

Figure S130. Absorption spectra of [**8**]Cl_2 in ACN at different concentrations (left) and respective molar absorption coefficients (right) calculated as the slopes of A = f(C) linear regressions at every wavelength from 300 to 800 nm.

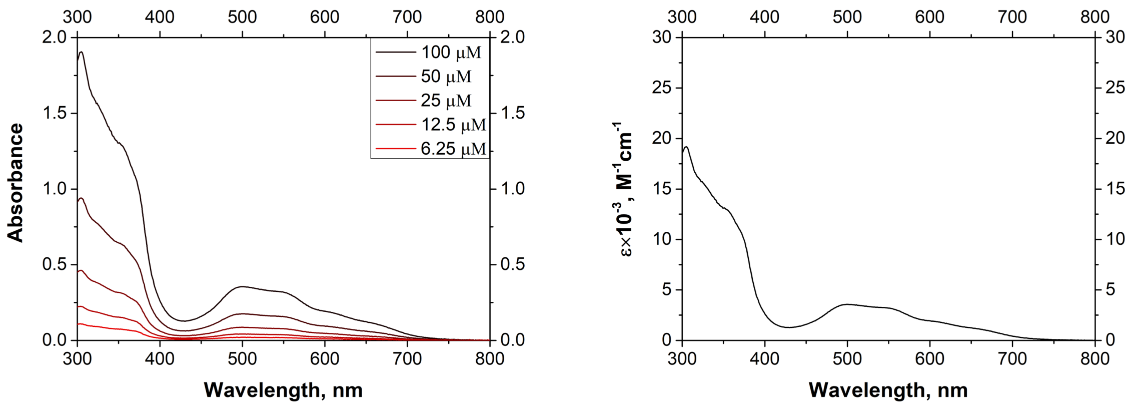

Figure S131. Absorption spectra of [**9**]Cl_2 in ACN at different concentrations (left) and respective molar absorption coefficients (right) calculated as the slopes of A = f(C) linear regressions at every wavelength from 300 to 800 nm.

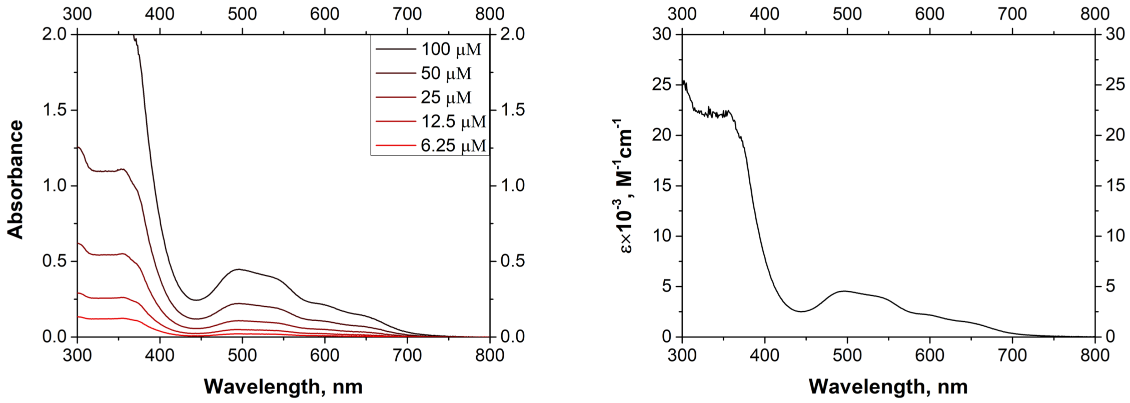

Figure S132. Absorption spectra of [**10**]Cl_2 in ACN at different concentrations (left) and respective molar absorption coefficients (right) calculated as the slopes of A = f(C) linear regressions at every wavelength from 300 to 800 nm.

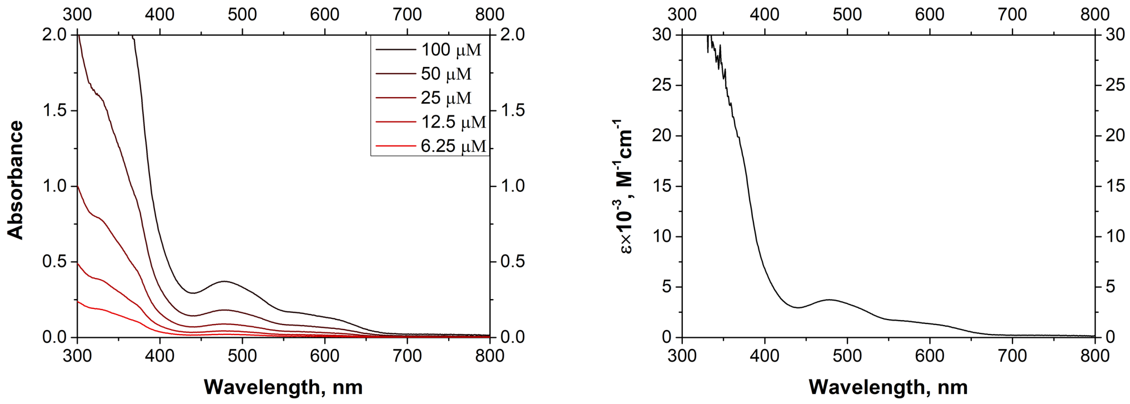

Figure S133. Absorption spectra of [**11**]Cl_2 in ACN at different concentrations (left) and respective molar absorption coefficients (right) calculated as the slopes of A = f(C) linear regressions at every wavelength from 300 to 800 nm.

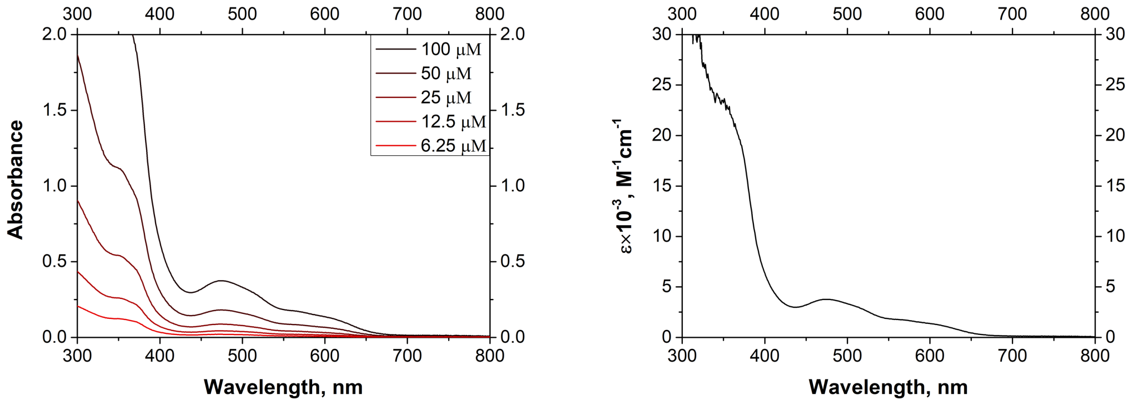

Figure S134. Absorption spectra of [**12**]Cl_2 in ACN at different concentrations (left) and respective molar absorption coefficients (right) calculated as the slopes of A = f(C) linear regressions at every wavelength from 300 to 800 nm.

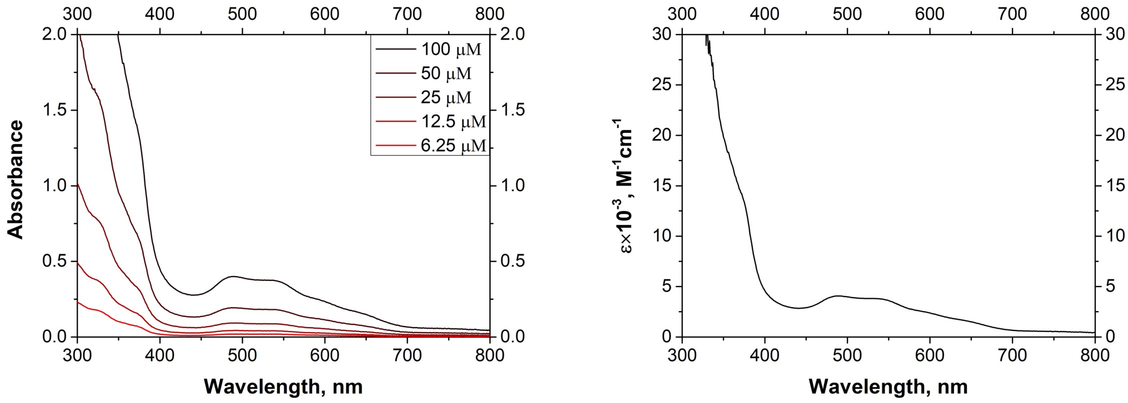

Figure S135. Absorption spectra of [**13**]Cl_2 in ACN at different concentrations (left) and respective molar absorption coefficients (right) calculated as the slopes of A = f(C) linear regressions at every wavelength from 300 to 800 nm.

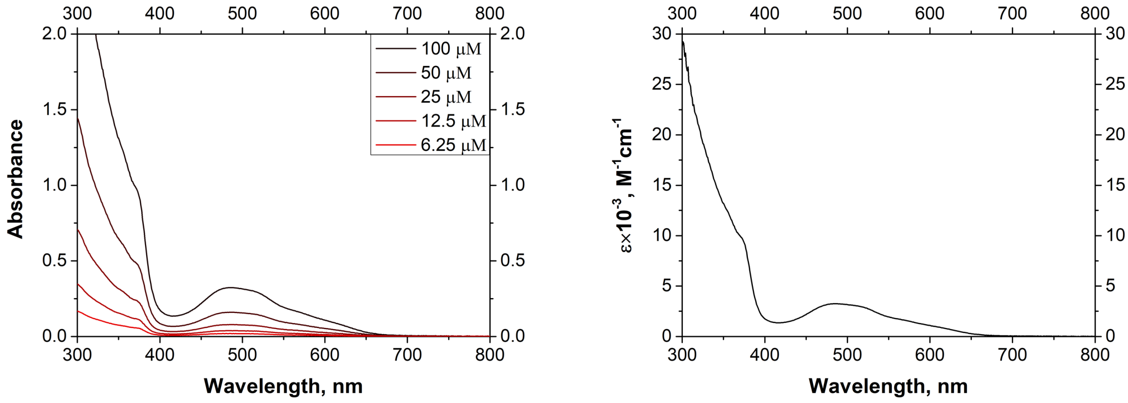

Figure S136. Absorption spectra of [**14**]$(PF_6)_2$ in ACN at different concentrations (left) and respective molar absorption coefficients (right) calculated as the slopes of A = f(C) linear regressions at every wavelength from 300 to 800 nm.

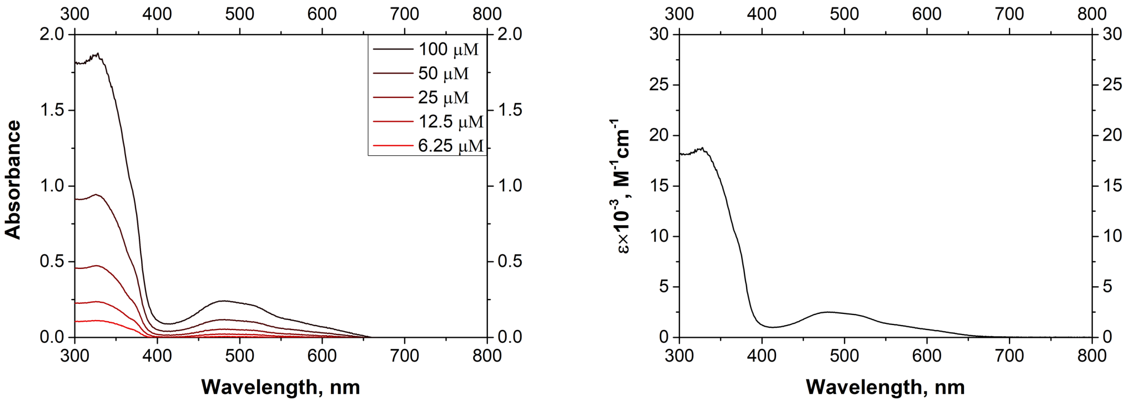

Figure S137. Absorption spectra of [**15**]$(PF_6)_2$ in ACN at different concentrations (left) and respective molar absorption coefficients (right) calculated as the slopes of A = f(C) linear regressions at every wavelength from 300 to 800 nm.

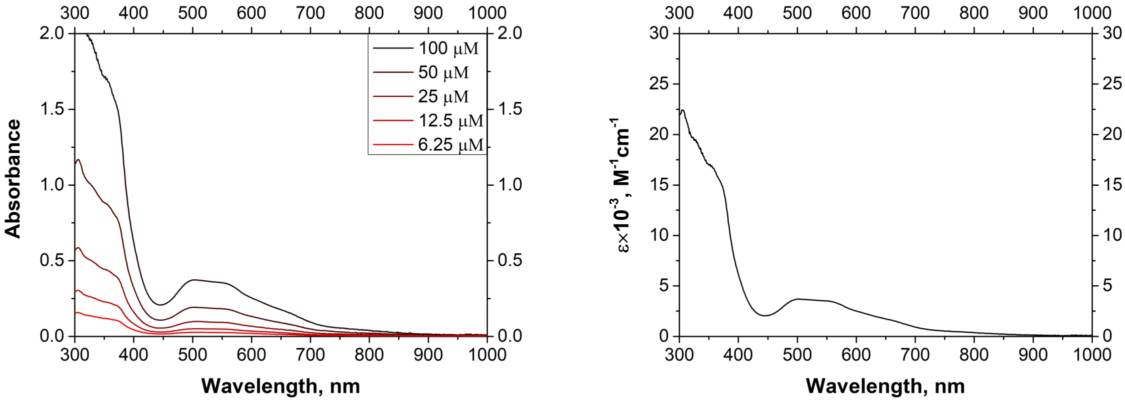

Figure S138. Absorption spectra of [**16**]Cl_2 in ACN at different concentrations (left) and respective molar absorption coefficients (right) calculated as the slopes of A = f(C) linear regressions at every wavelength from 300 to 1000 nm.

2.3 Ligand photosubstitution monitored by UV-Vis

*[Ru(baptpy)(Py)]Cl₂ ([**6**]Cl₂)*

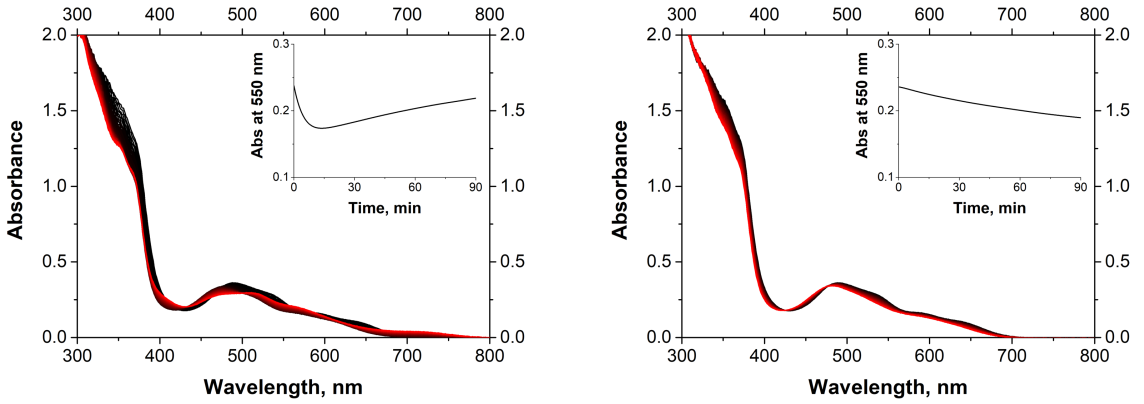

Figure S139. Evolution of the absorption spectra of [**6**]Cl_2 in ACN (0.1 mM solution) upon irradiation with 625 nm red (left) and 730 nm near-infrared (right) light for 1.5 h. Spectra were measured every 0.5 min and evolved from black $t_0 \rightarrow$ red t_{90}, the insets show absorbance evolution under light.

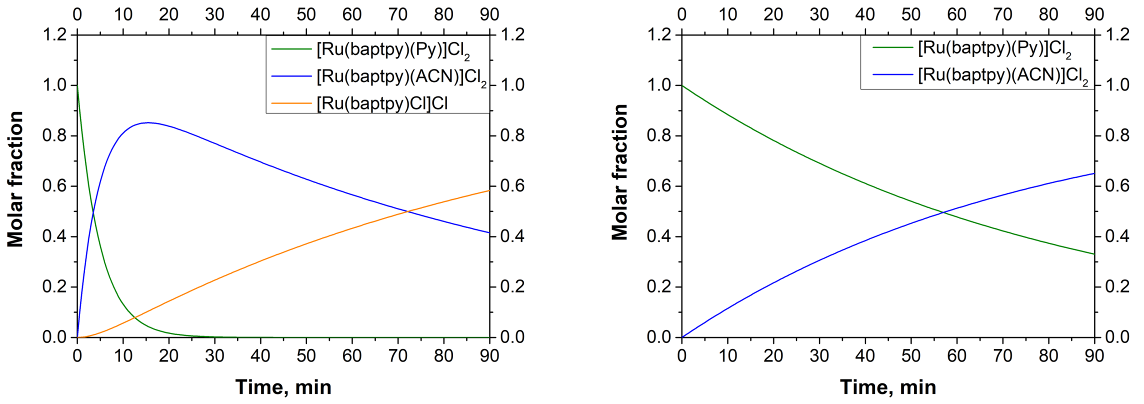

Figure S140. Molar fraction evolution of [**6**]Cl_2 in ACN (0.1 mM solution) upon irradiation with 625 nm red (left) and 730 nm near-infrared (right) light for 1.5 h.

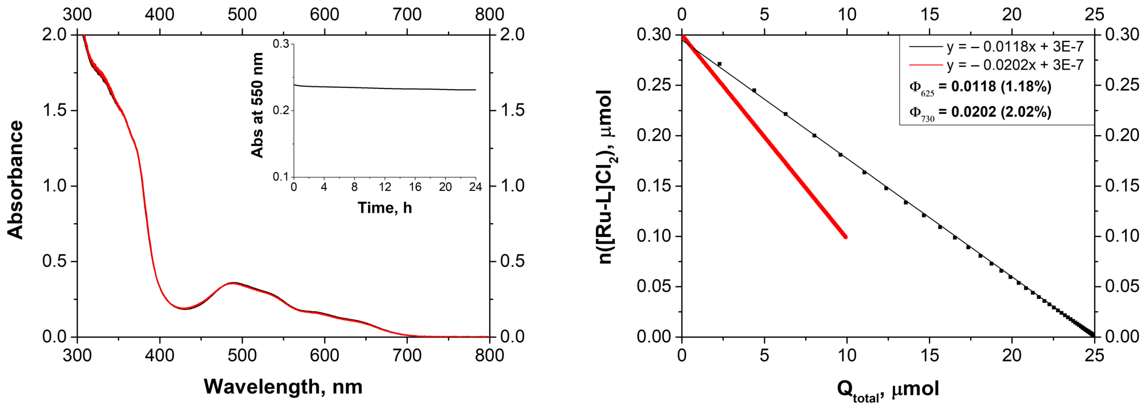

Figure S141. Evolution of the absorption spectra of [**6**]Cl_2 in ACN (0.1 mM solution) over 24 h in dark (left) and photosubstitution quantum yields calculated as negative slopes of n = f(Q_{total}) linear regressions (right).

*[Ru(baptpy)(**STF-31**)]Cl_2 ([7]Cl_2)*

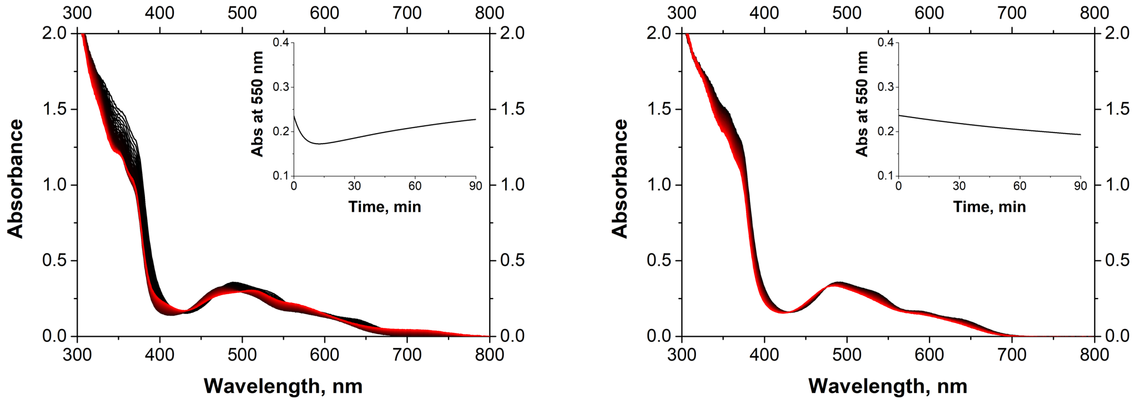

Figure S142. Evolution of the absorption spectra of [**7**]Cl_2 in ACN (0.1 mM solution) upon irradiation with 625 nm red (left) and 730 nm near-infrared (right) light for 1.5 h. Spectra were measured every 0.5 min and evolved from black $t_0 \rightarrow$ red t_{90}, the insets show absorbance evolution under light.

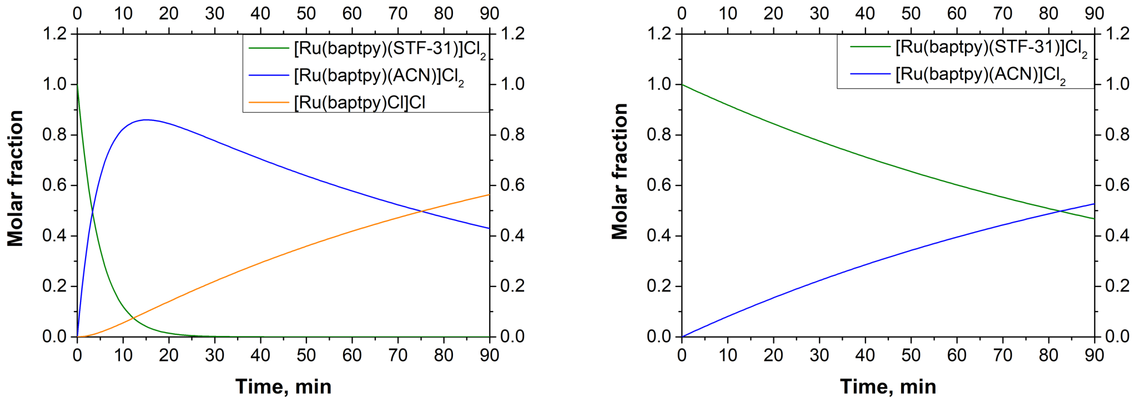

Figure S143. Molar fraction evolution of [**7**]Cl_2 in ACN (0.1 mM solution) upon irradiation with 625 nm red (left) and 730 nm near-infrared (right) light for 1.5 h.

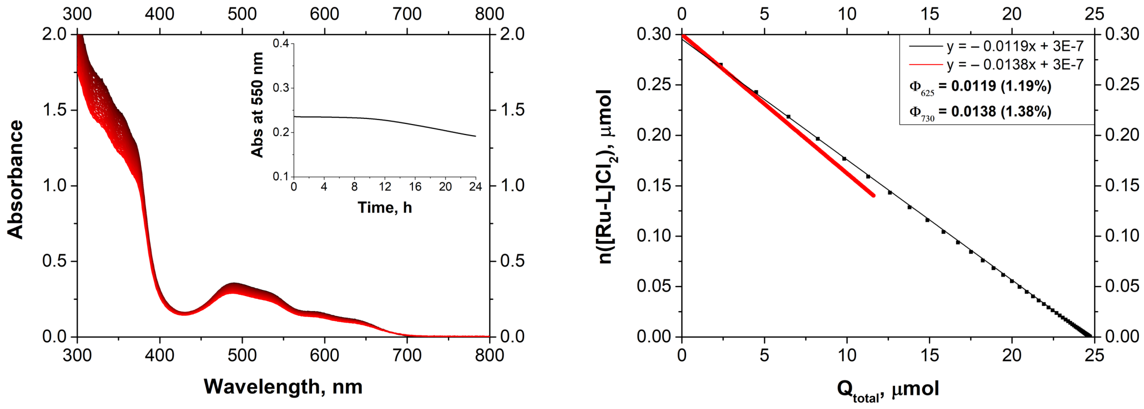

Figure S144. Evolution of the absorption spectra of [**7**]Cl_2 in ACN (0.1 mM solution) over 24 h in dark (left) and photosubstitution quantum yields calculated as negative slopes of $n = f(Q_{total})$ linear regressions (right).

*[Ru(baptpy)(**RAD-51-IN-1**)]Cl*$_2$ *([**8**]Cl*$_2$*)*

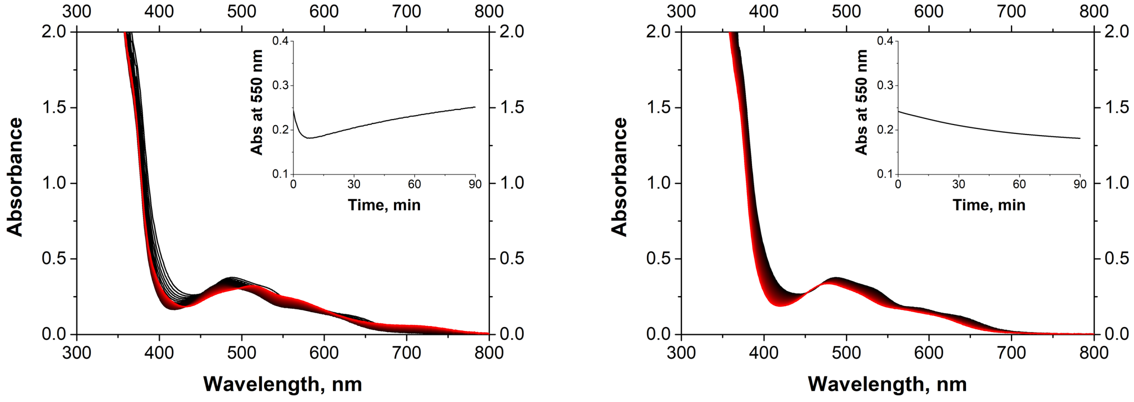

Figure S145. Evolution of the absorption spectra of [**8**]Cl$_2$ in ACN (0.1 mM solution) upon irradiation with 625 nm red (left) and 730 nm near-infrared (right) light for 1.5 h. Spectra were measured every 0.5 min and evolved from black $t_0 \rightarrow$ red t_{90}, the insets show absorbance evolution under light.

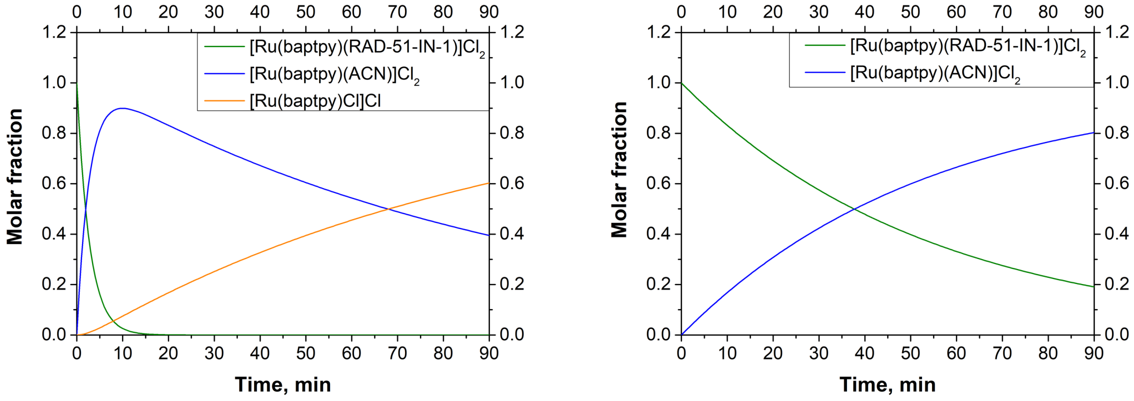

Figure S146. Molar fraction evolution of [**8**]Cl$_2$ in ACN (0.1 mM solution) upon irradiation with 625 nm red (left) and 730 nm near-infrared (right) light for 1.5 h.

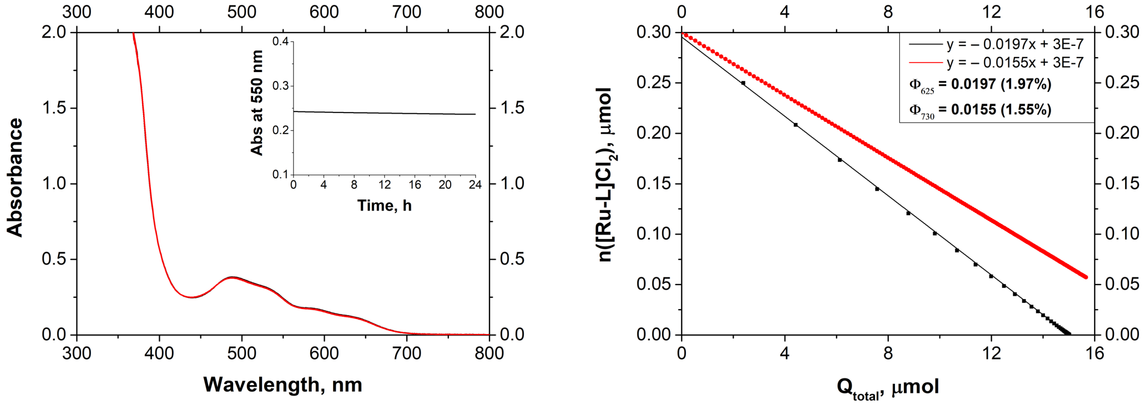

Figure S147. Evolution of the absorption spectra of [**8**]Cl$_2$ in ACN (0.1 mM solution) over 24 h in dark (left) and photosubstitution quantum yields calculated as negative slopes of n = f(Q_{total}) linear regressions (right).

*[Ru(baptpy)(**QC-82**)]Cl_2 ([**9**]Cl_2)*

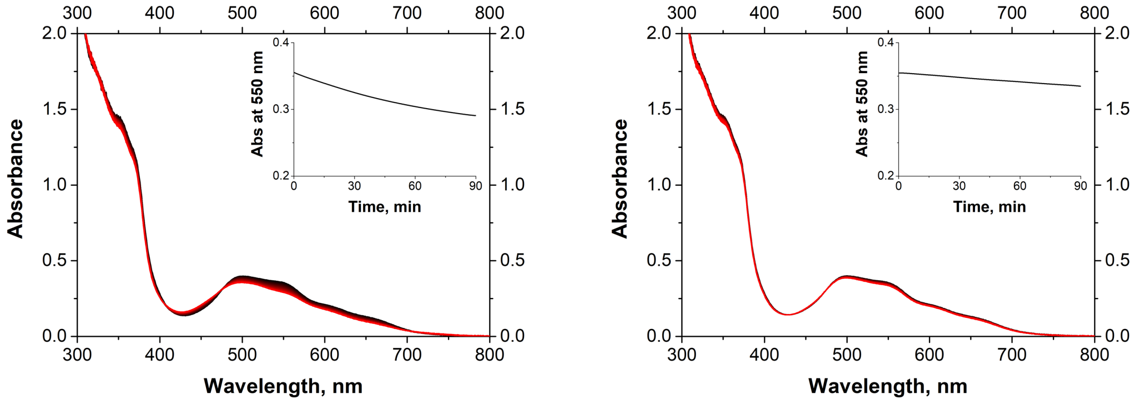

Figure S148. Evolution of the absorption spectra of [**9**]Cl_2 in ACN (0.1 mM solution) upon irradiation with 625 nm red (left) and 730 nm near-infrared (right) light for 1.5 h. Spectra were measured every 0.5 min and evolved from black $t_0 \rightarrow$ red t_{90}, the insets show absorbance evolution under light.

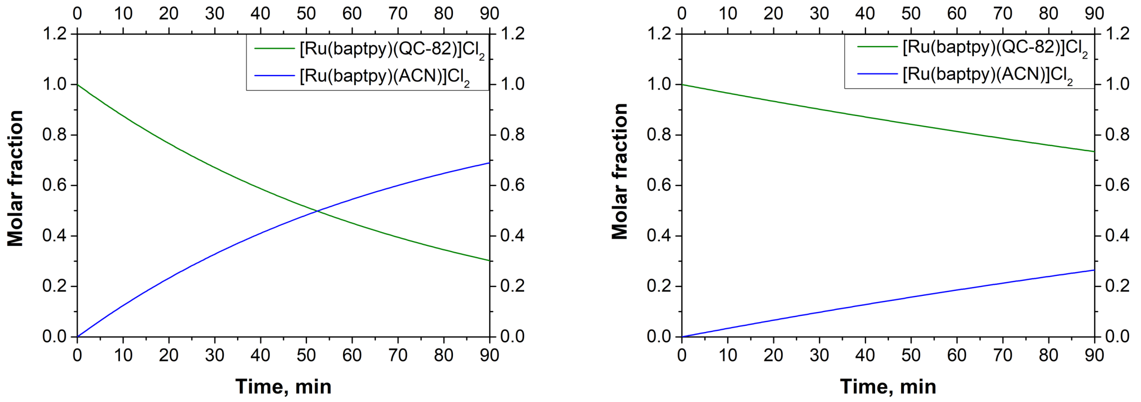

Figure S149. Molar fraction evolution of [**9**]Cl_2 in ACN (0.1 mM solution) upon irradiation with 625 nm red (left) and 730 nm near-infrared (right) light for 1.5 h.

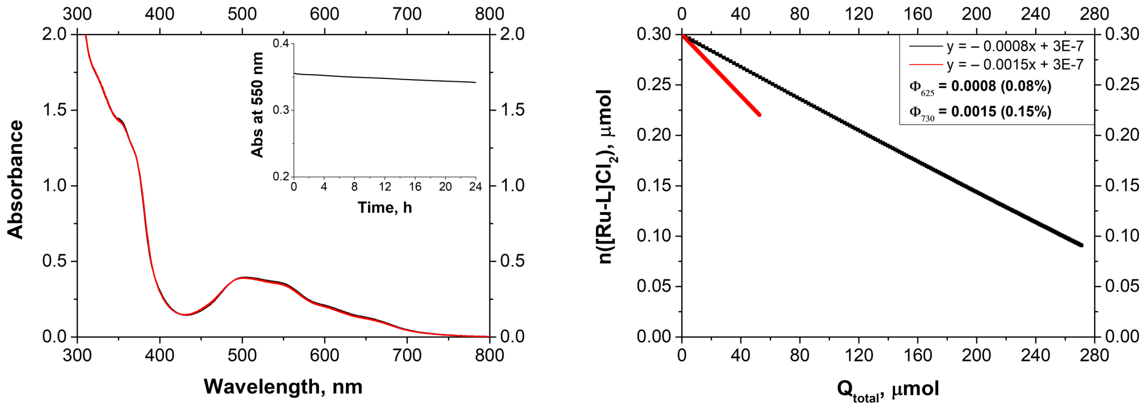

Figure S150. Evolution of the absorption spectra of [**9**]Cl_2 in ACN (0.1 mM solution) over 24 h in dark (left) and photosubstitution quantum yields calculated as negative slopes of n = f(Q_{total}) linear regressions (right).

*[Ru(baptpy)(**Norharmane**)]Cl_2 ([**10**]Cl_2)*

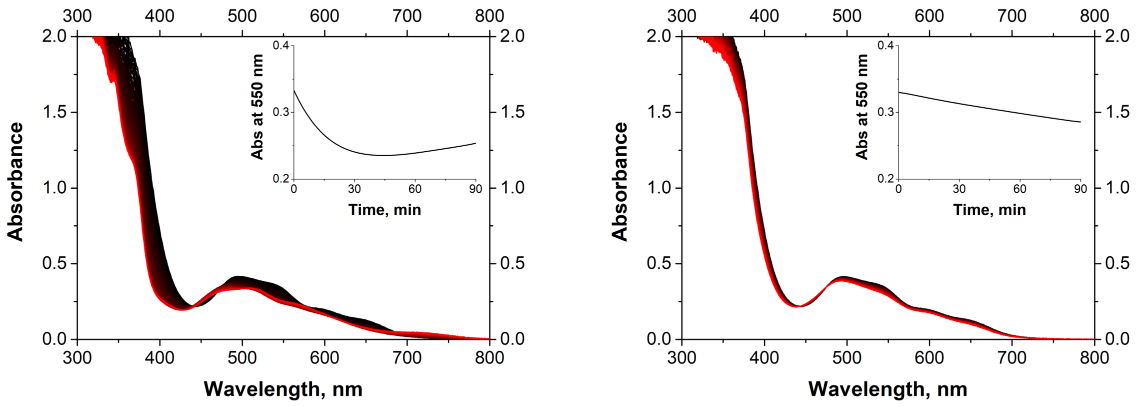

Figure S151. Evolution of the absorption spectra of [**10**]Cl_2 in ACN (0.1 mM solution) upon irradiation with 625 nm red (left) and 730 nm near-infrared (right) light for 1.5 h. Spectra were measured every 0.5 min and evolved from black $t_0 \rightarrow$ red t_{90}, the insets show absorbance evolution under light.

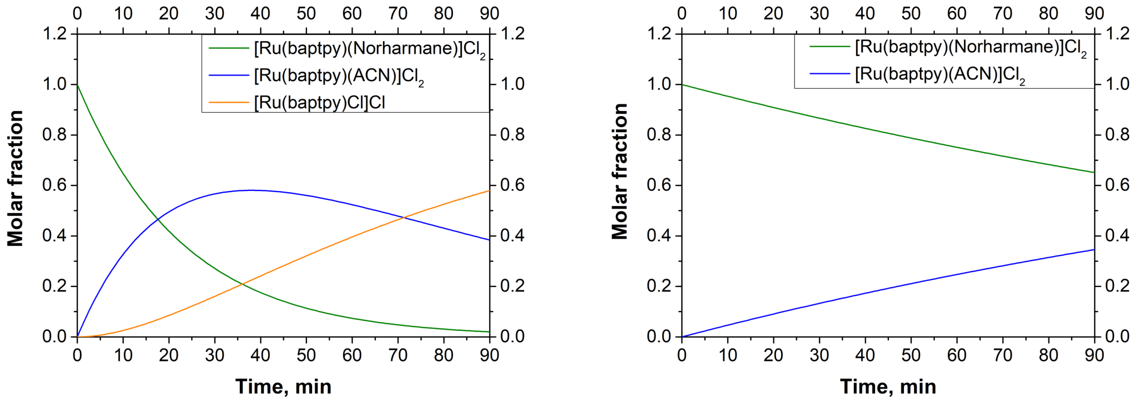

Figure S152. Molar fraction evolution of [**10**]Cl_2 in ACN (0.1 mM solution) upon irradiation with 625 nm red (left) and 730 nm near-infrared (right) light for 1.5 h.

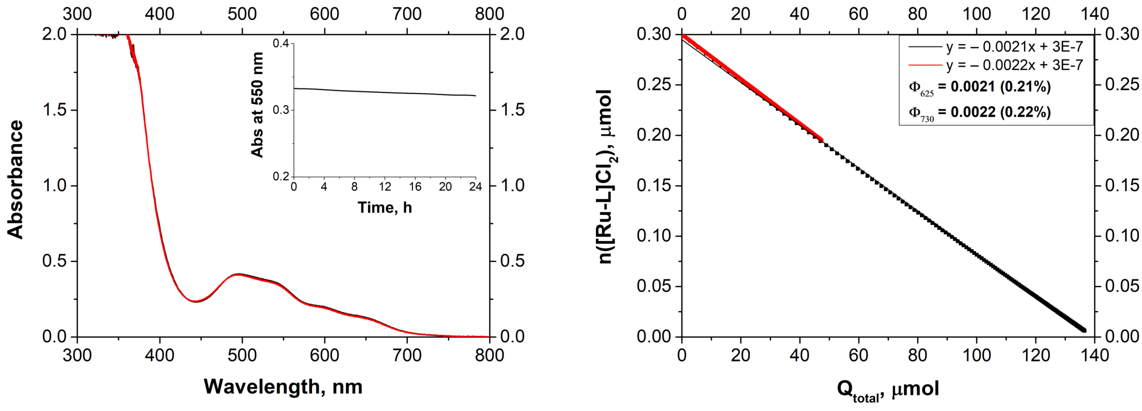

Figure S153. Evolution of the absorption spectra of [**10**]Cl_2 in ACN (0.1 mM solution) over 24 h in dark (left) and photosubstitution quantum yields calculated as negative slopes of n = f(Q_{total}) linear regressions (right).

*[Ru(baptpy)(**Neratinib**)]Cl_2 ([**11**]Cl_2)*

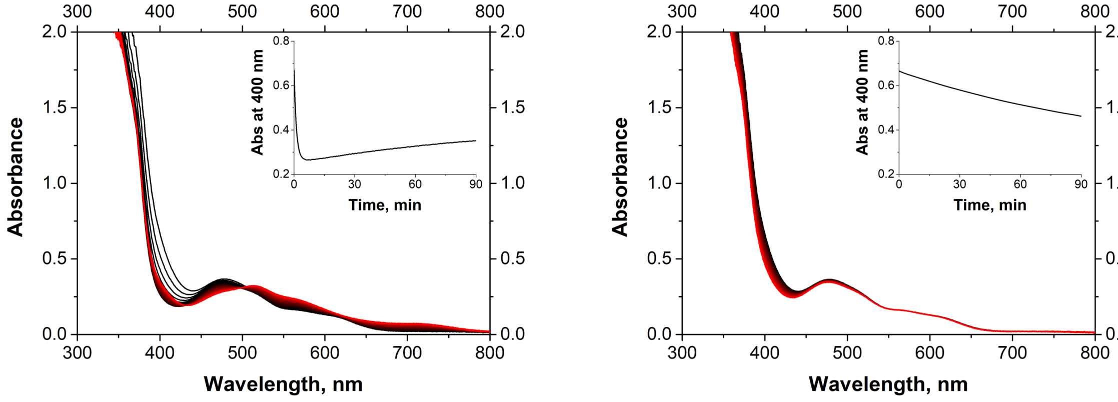

Figure S154. Evolution of the absorption spectra of [**11**]Cl_2 in ACN (0.1 mM solution) upon irradiation with 625 nm red (left) and 730 nm near-infrared (right) light for 1.5 h . Spectra were measured every 0.5 min and evolved from black $t_0 \rightarrow$ red t_{90}, the insets show absorbance evolution under light.

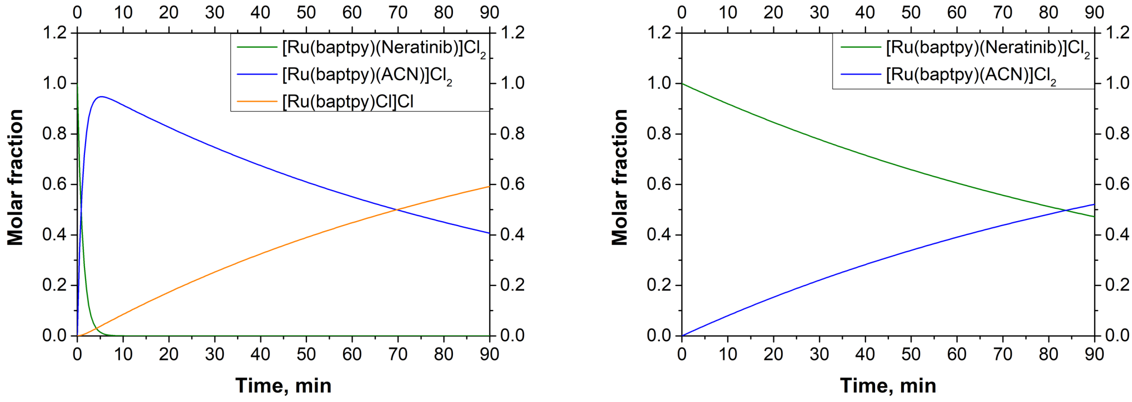

Figure S155. Molar fraction evolution of [**11**]Cl_2 in ACN (0.1 mM solution) upon irradiation with 625 nm red (left) and 730 nm near-infrared (right) light for 1.5 h.

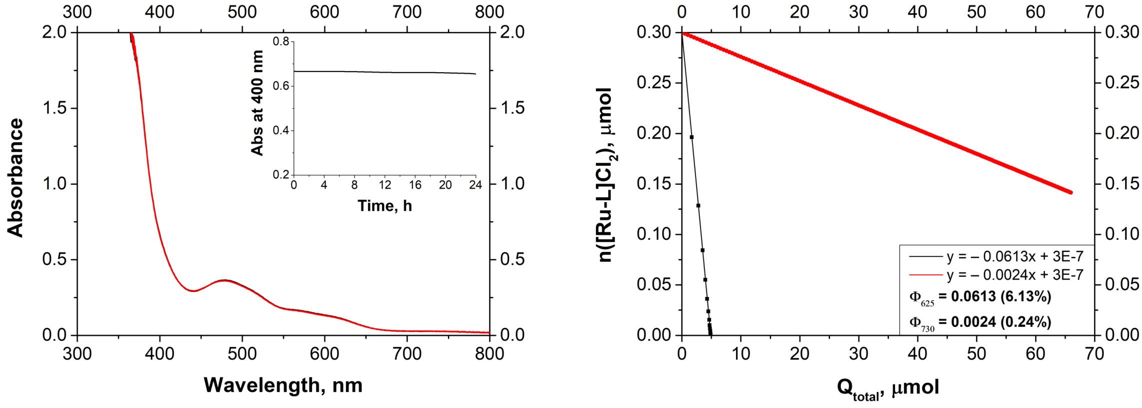

Figure S156. Evolution of the absorption spectra of [**11**]Cl_2 in ACN (0.1 mM solution) over 24 h in dark (left) and photosubstitution quantum yields calculated as negative slopes of n = f(Q_{total}) linear regressions (right).

*[Ru(baptpy)(**Bosutinib**)]Cl₂ ([**12**]Cl₂)*

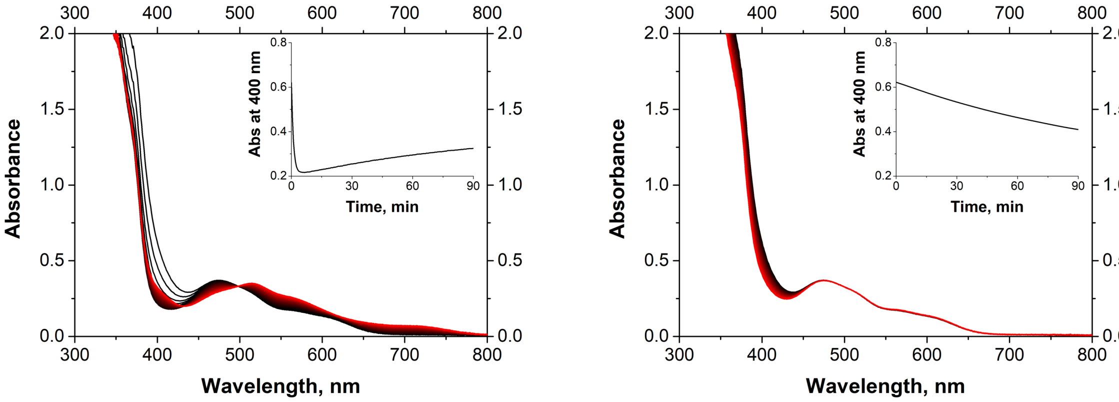

Figure S157. Evolution of the absorption spectra of [**12**]Cl_2 in ACN (0.1 mM solution) upon irradiation with 625 nm red (left) and 730 nm near-infrared (right) light for 1.5 h. Spectra were measured every 0.5 min and evolved from black $t_0 \rightarrow$ red t_{90}, the insets show absorbance evolution under light.

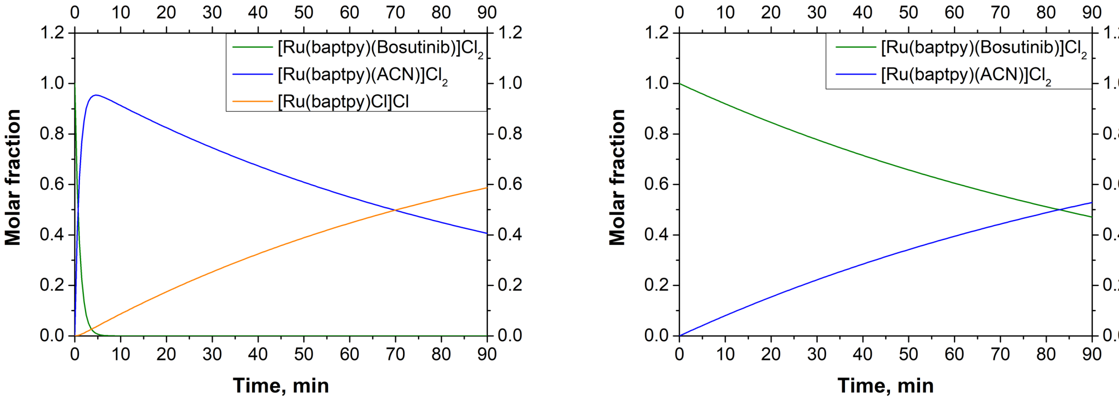

Figure S158. Molar fraction evolution of [**12**]Cl_2 in ACN (0.1 mM solution) upon irradiation with 625 nm red (left) and 730 nm near-infrared (right) light for 1.5 h.

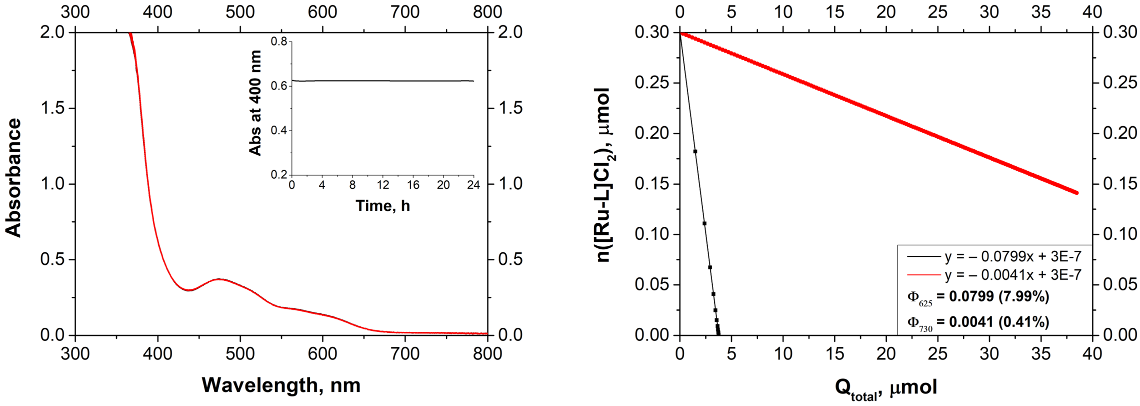

Figure S159. Evolution of the absorption spectra of [**12**]Cl_2 in ACN (0.1 mM solution) over 24 h in dark (left) and photosubstitution quantum yields calculated as negative slopes of n = f(Q_{total}) linear regressions (right).

*[Ru(baptpy)(**Ponatinib**)]Cl₂ ([**13**]Cl₂)*

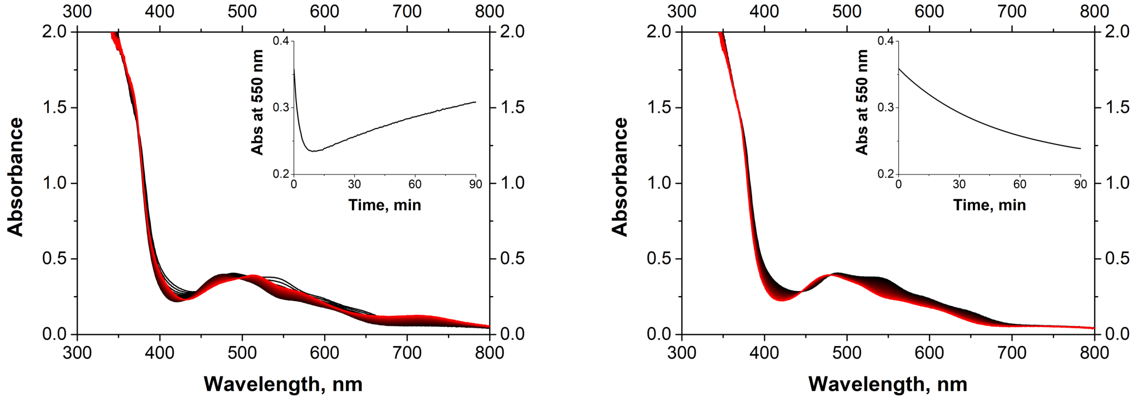

Figure S160. Evolution of the absorption spectra of [**13**]Cl_2 in ACN (0.1 mM solution) upon irradiation with 625 nm red (left) and 730 nm near-infrared (right) light for 1.5 h. Spectra were measured every 0.5 min and evolved from black $t_0 \rightarrow$ red t_{90}, the insets show absorbance evolution under light.

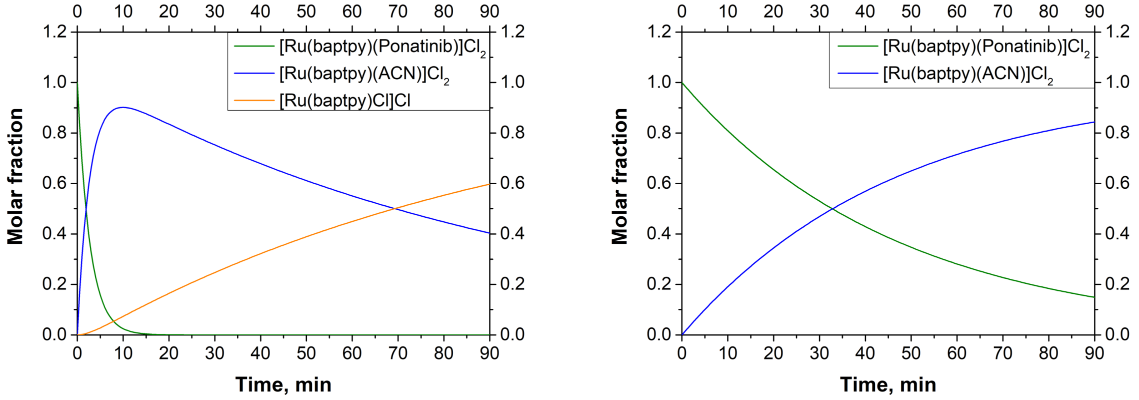

Figure S161. Molar fraction evolution of [**13**]Cl_2 in ACN (0.1 mM solution) upon irradiation with 625 nm red (left) and 730 nm near-infrared (right) light for 1.5 h.

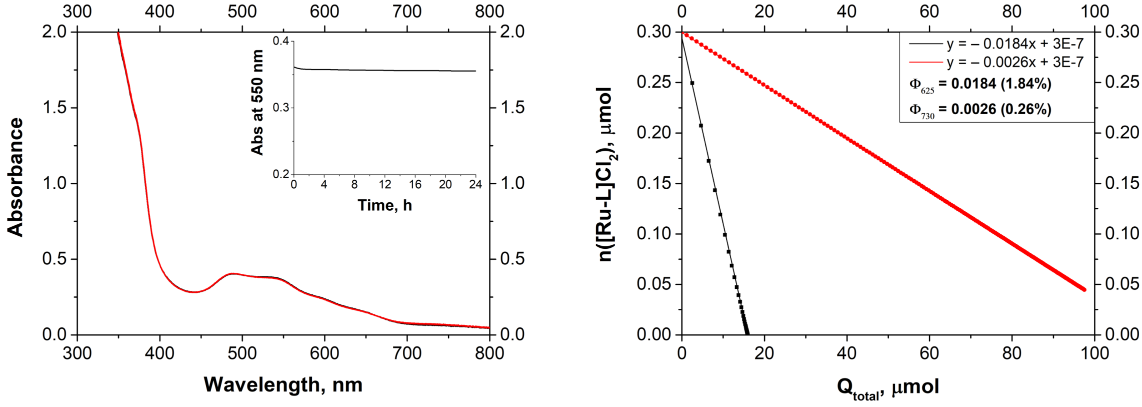

Figure S162. Evolution of the absorption spectra of [**13**]Cl_2 in ACN (0.1 mM solution) over 24 h in dark (left) and photosubstitution quantum yields calculated as negative slopes of $n = f(Q_{total})$ linear regressions (right).

*[Ru(baptpy)(**Albendazole**)](PF_6)$_2$ ([**14**](PF_6)$_2$)*

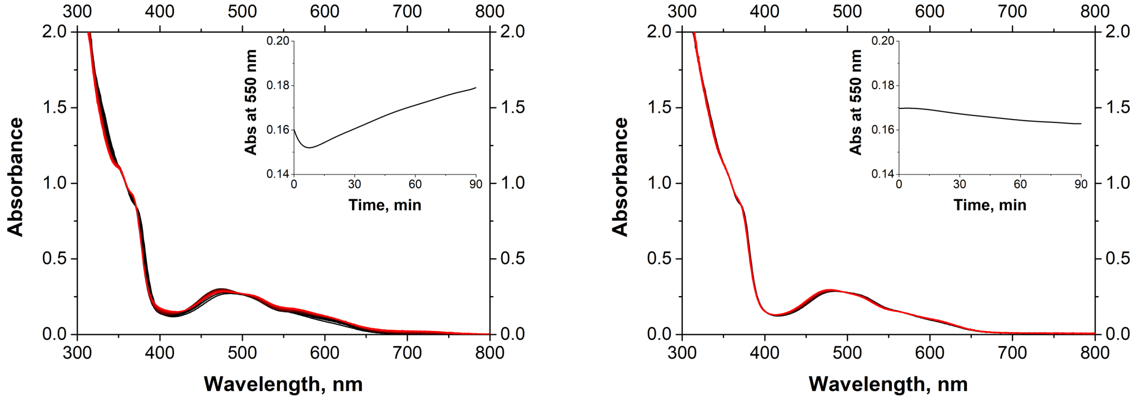

Figure S163. Evolution of the absorption spectra of [**14**](PF_6)$_2$ in ACN (0.1 mM solution) upon irradiation with 625 nm red (left) and 730 nm near-infrared (right) light for 1.5 h. Spectra were measured every 0.5 min and evolved from black $t_0 \rightarrow$ red t_{90}, the insets show absorbance evolution under light.

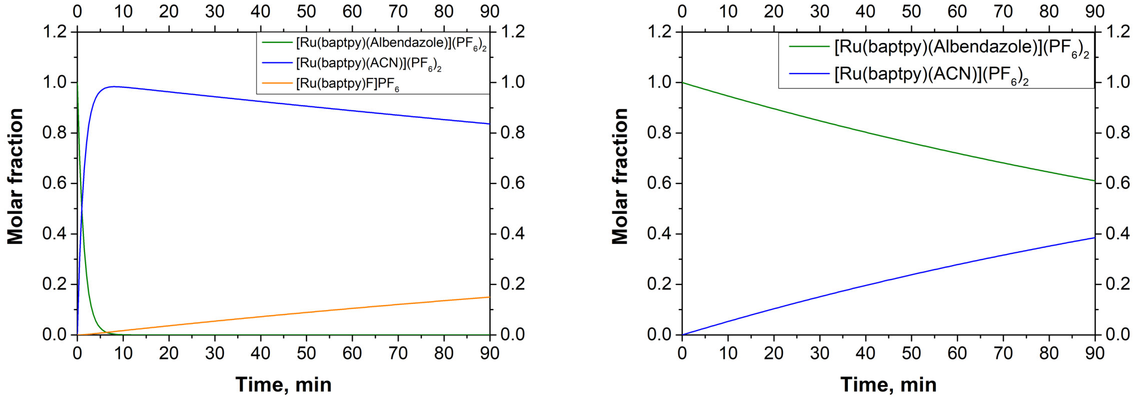

Figure S164. Molar fraction evolution of [**14**](PF_6)$_2$ in ACN (0.1 mM solution) upon irradiation with 625 nm red (left) and 730 nm near-infrared (right) light for 1.5 h.

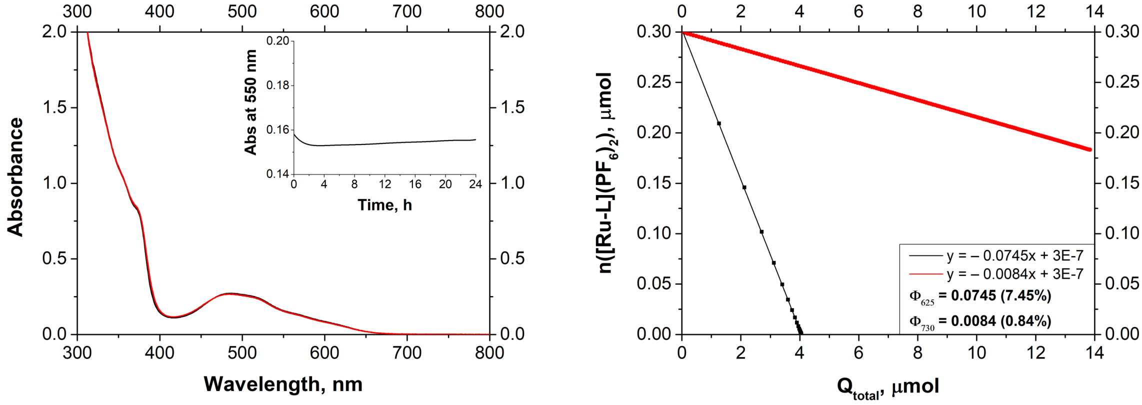

Figure S165. Evolution of the absorption spectra of [**14**](PF_6)$_2$ in ACN (0.1 mM solution) over 24 h in dark (left) and photosubstitution quantum yields calculated as negative slopes of n = f(Q_{total}) linear regressions (right).

*[Ru(baptpy)(**MTI**)](PF₆)₂ ([**15**](PF₆)₂)*

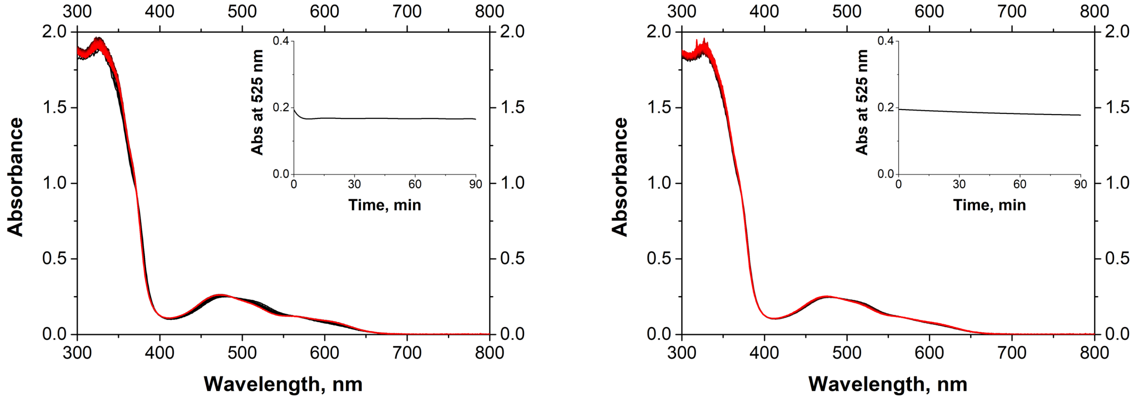

Figure S166. Evolution of the absorption spectra of [**15**](PF_6)$_2$ in ACN (0.1 mM solution) upon irradiation with 625 nm red (left) and 730 nm near-infrared (right) light for 1.5 h. Spectra were measured every 0.5 min and evolved from black $t_0 \rightarrow$ red t_{90}, the insets show absorbance evolution under light.

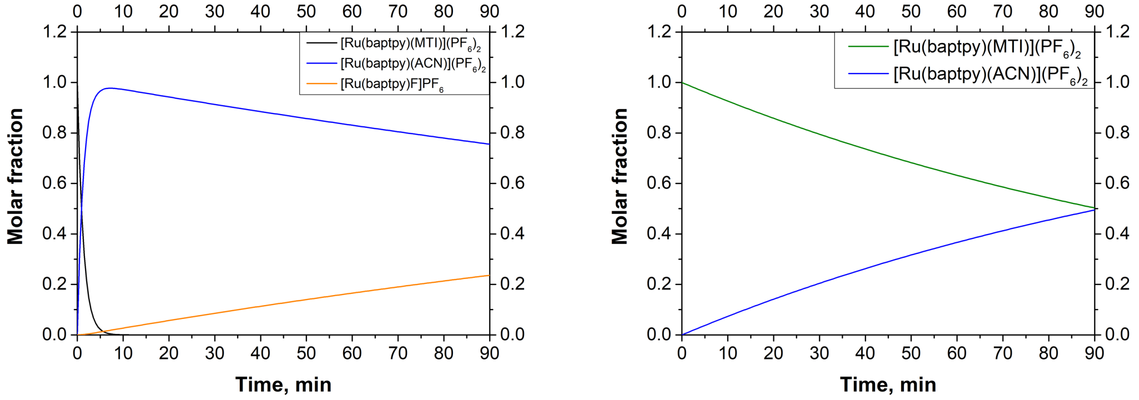

Figure S167. Molar fraction evolution of [**15**](PF_6)$_2$ in ACN (0.1 mM solution) upon irradiation with 625 nm red (left) and 730 nm near-infrared (right) light for 1.5 h.

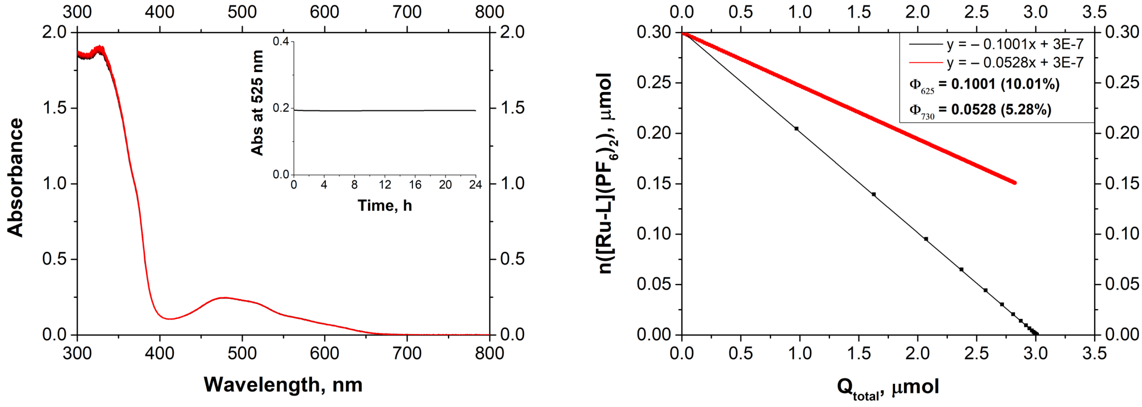

Figure S168. Evolution of the absorption spectra of [**15**](PF_6)$_2$ in ACN (0.1 mM solution) over 24 h in dark (left) and photosubstitution quantum yields calculated as negative slopes of n = f(Q_{total}) linear regressions (right).

*[Ru(baptpy)(**Gemcitabine**)]Cl₂ ([**16**]Cl₂)*

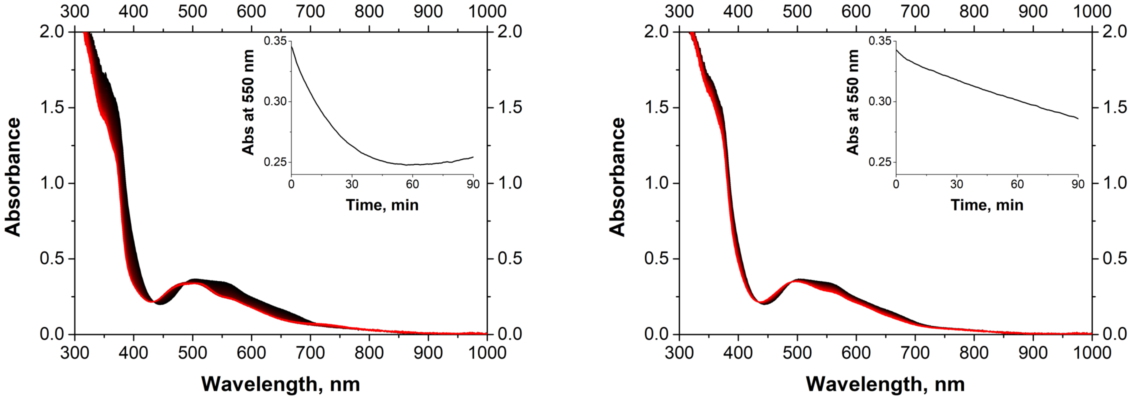

Figure S169. Evolution of the absorption spectra of [**16**]Cl_2 in ACN (0.1 mM solution) upon irradiation with 625 nm red (left) and 730 nm near-infrared (right) light for 1.5 h. Spectra were measured every 0.5 min and evolved from black $t_0 \rightarrow$ red t_{90}, the insets show absorbance evolution under light.

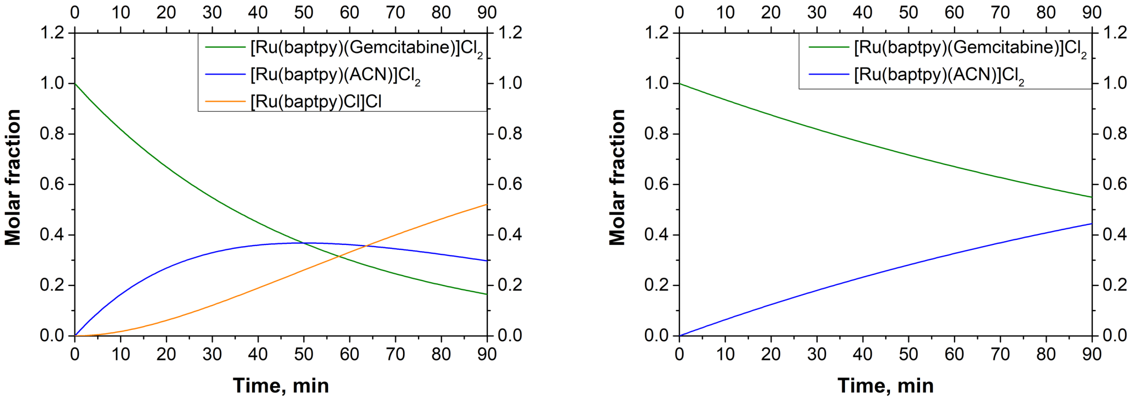

Figure S170. Molar fraction evolution of [**16**]Cl_2 in ACN (0.1 mM solution) upon irradiation with 625 nm red (left) and 730 nm near-infrared (right) light for 1.5 h.

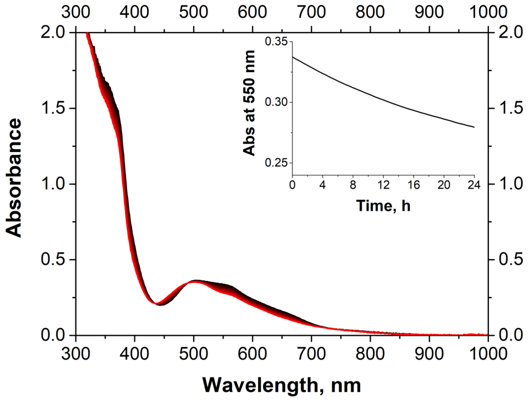

Figure S171. Evolution of the absorption spectra of [**16**]Cl_2 in ACN (0.1 mM solution) over 24 h in dark.

2.4 Stability in water monitored by UV-Vis and HPLC

Solutions of each complex (0.1 mM, 5 mL) in milli-Q water with 2% v/v MeOH were prepared and at t = 0 h the first HPLC chromatograms were measured. The absobtion evolution for every compound was then measured by UV-Vis spectrophotometer over 24 h in dark at 25 °C, scan every 30 min. At t = 24 h, the final HPLC chromatograms were measured.

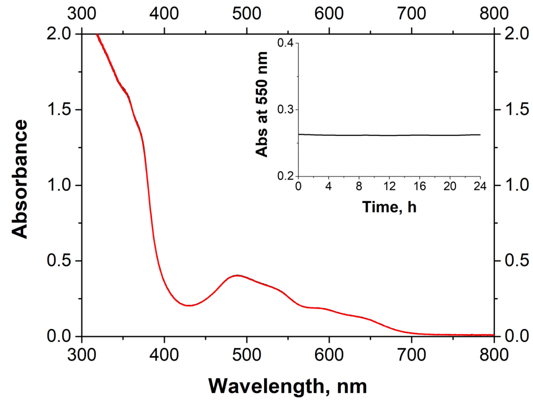

Figure S172. Evolution of the absorption spectra of [**6**]Cl_2 in H_2O with 2% v/v MeOH (0.1 mM solution) over 24 h in dark at 298 K.

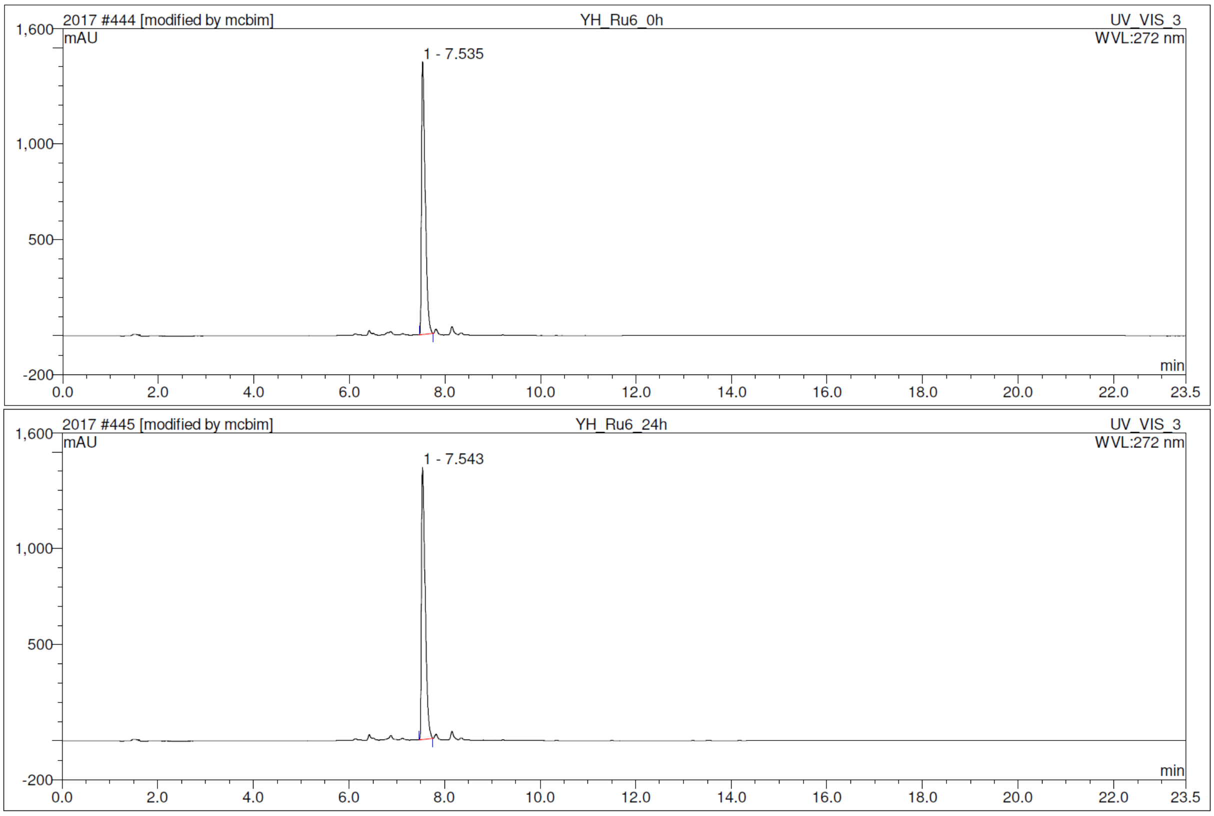

Figure S173. HPLC traces of [**6**]Cl_2 (t_R = 7.5 min) at the beginning (top) and at the end (bottom) of UV-Vis stability test indicating 1.69% reduction in relative peak area over 24 h in H_2O with 2% v/v MeOH (0.1 mM solution).

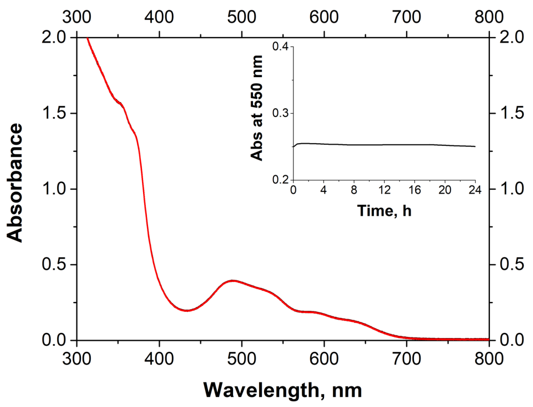

Figure S174. Evolution of the absorption spectra of [**7**]Cl_2 in H_2O with 2% v/v MeOH (0.1 mM solution) over 24 h in dark at 298 K.

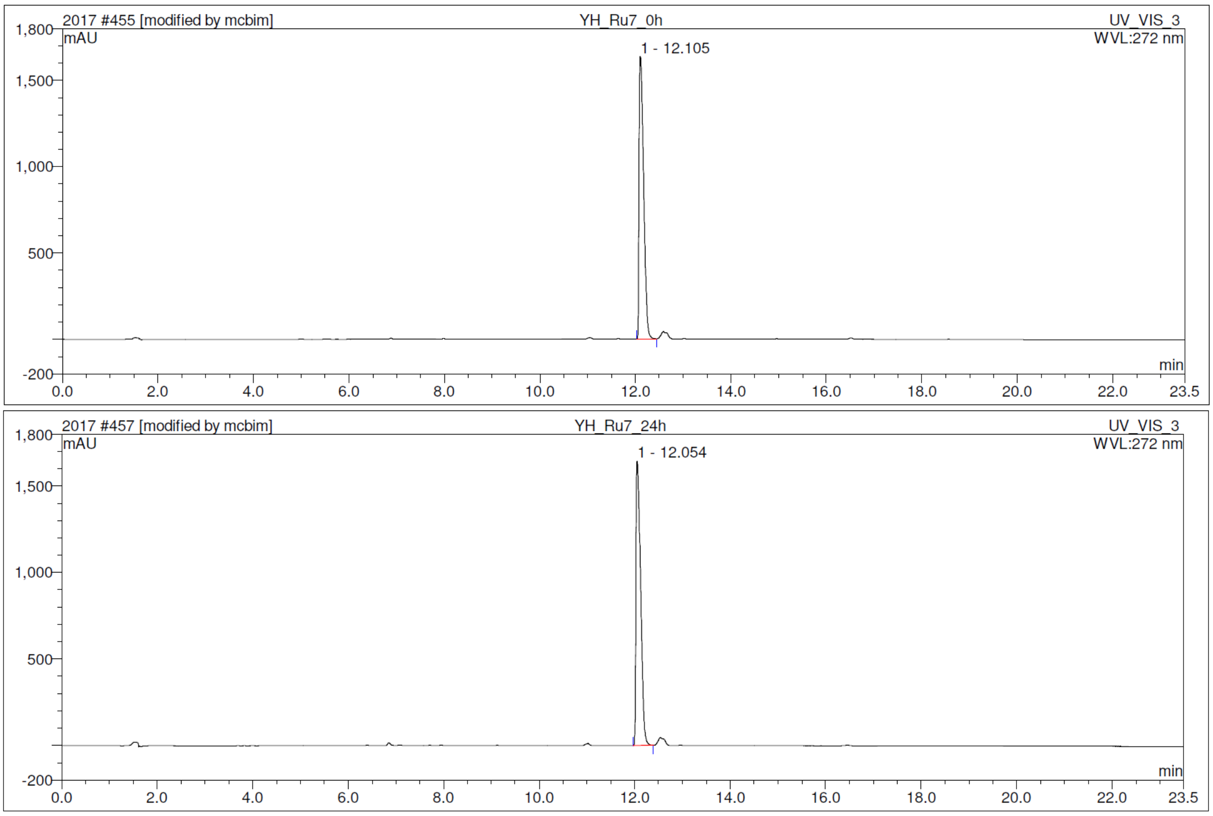

Figure S175. HPLC traces of [**7**]Cl_2 (t_R = 12.1 min) at the beginning (top) and at the end (bottom) of UV-Vis stability test indicating 1.80% reduction in relative peak area over 24 h in H_2O with 2% v/v MeOH (0.1 mM solution).

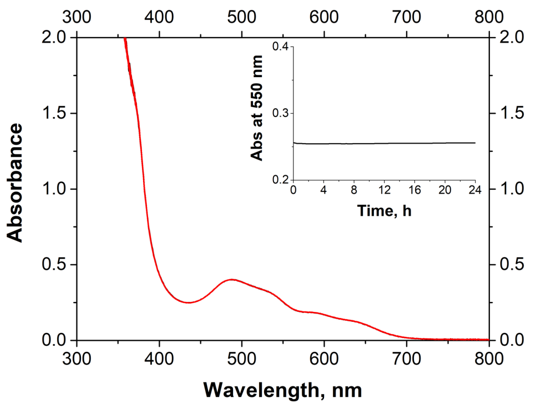

Figure S176. Evolution of the absorption spectra of [**8**]Cl_2 in H_2O with 2% v/v MeOH (0.1 mM solution) over 24 h in dark at 298 K.

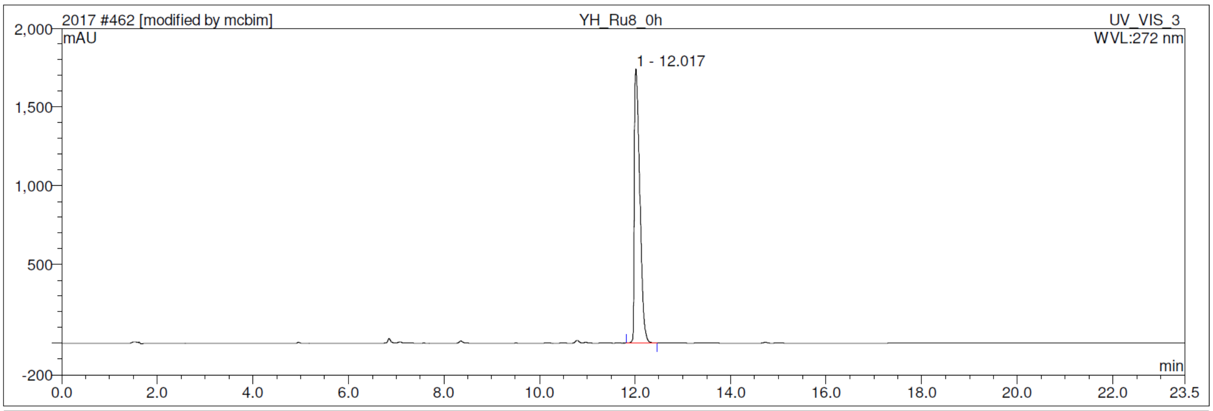

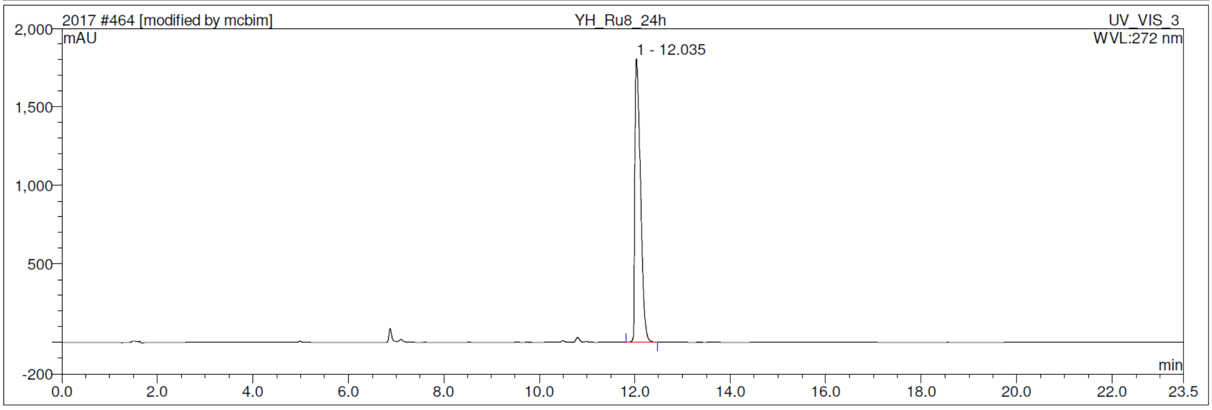

Figure S177. HPLC traces of [**8**]Cl_2 (t_R = 12.0 min) at the beginning (top) and at the end (bottom) of UV-Vis stability test indicating 1.91% reduction in relative peak area over 24 h in H_2O with 2% v/v MeOH (0.1 mM solution).

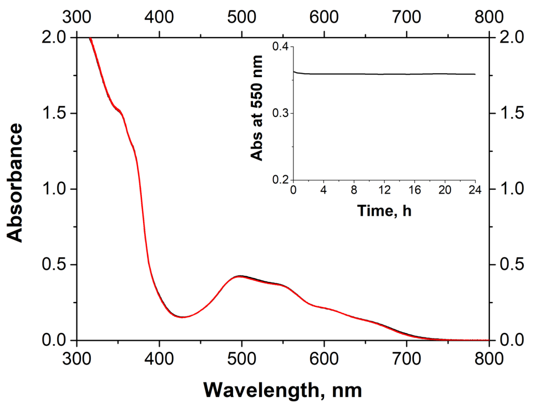

Figure S178. Evolution of the absorption spectra of [**9**]Cl_2 in H_2O with 2% v/v MeOH (0.1 mM solution) over 24 h in dark at 298 K.

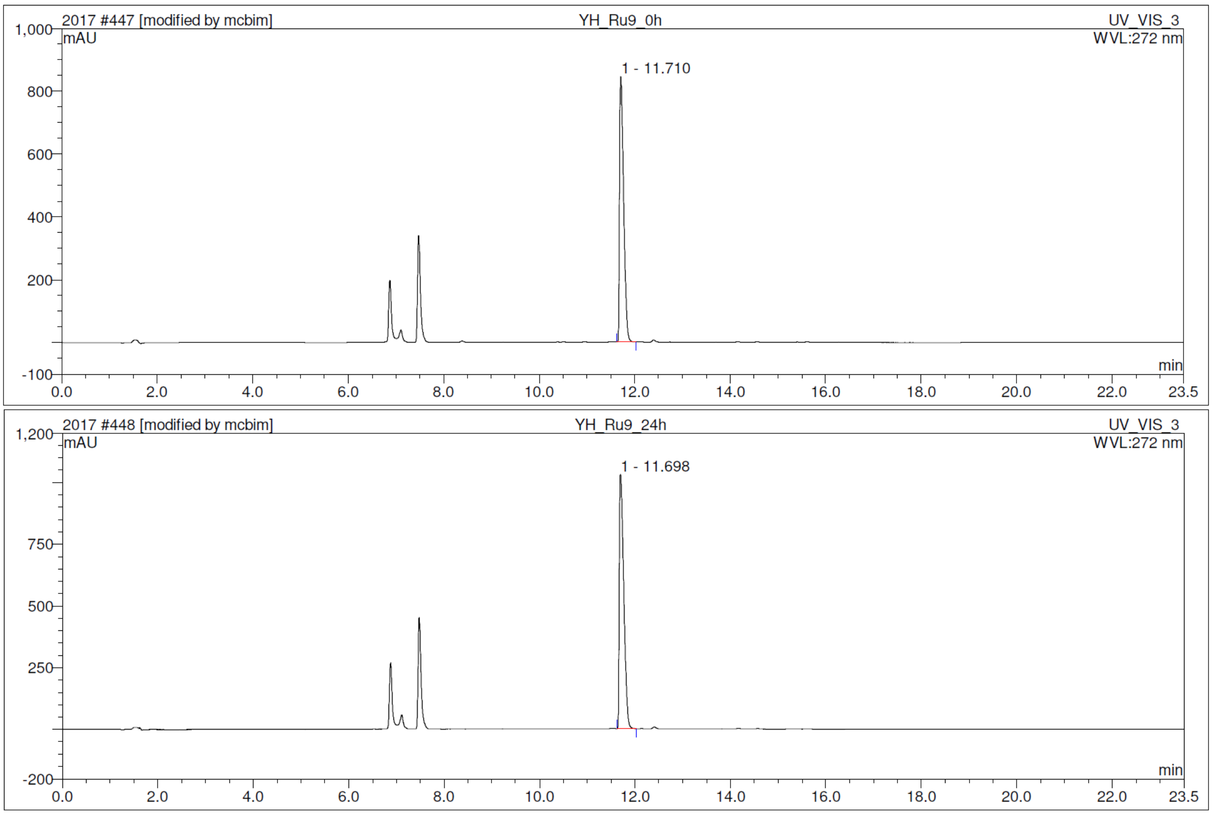

Figure S179. HPLC traces of [**9**]Cl_2 (t_R = 11.7 min) at the beginning (top) and at the end (bottom) of UV-Vis stability test indicating 0.99% reduction in relative peak area over 24 h in H_2O with 2% v/v MeOH (0.1 mM solution).

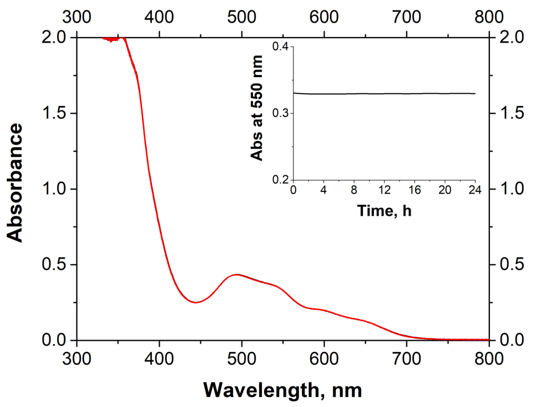

Figure S180. Evolution of the absorption spectra of [**10**]Cl_2 in H_2O with 2% v/v MeOH (0.1 mM solution) over 24 h in dark at 298 K.

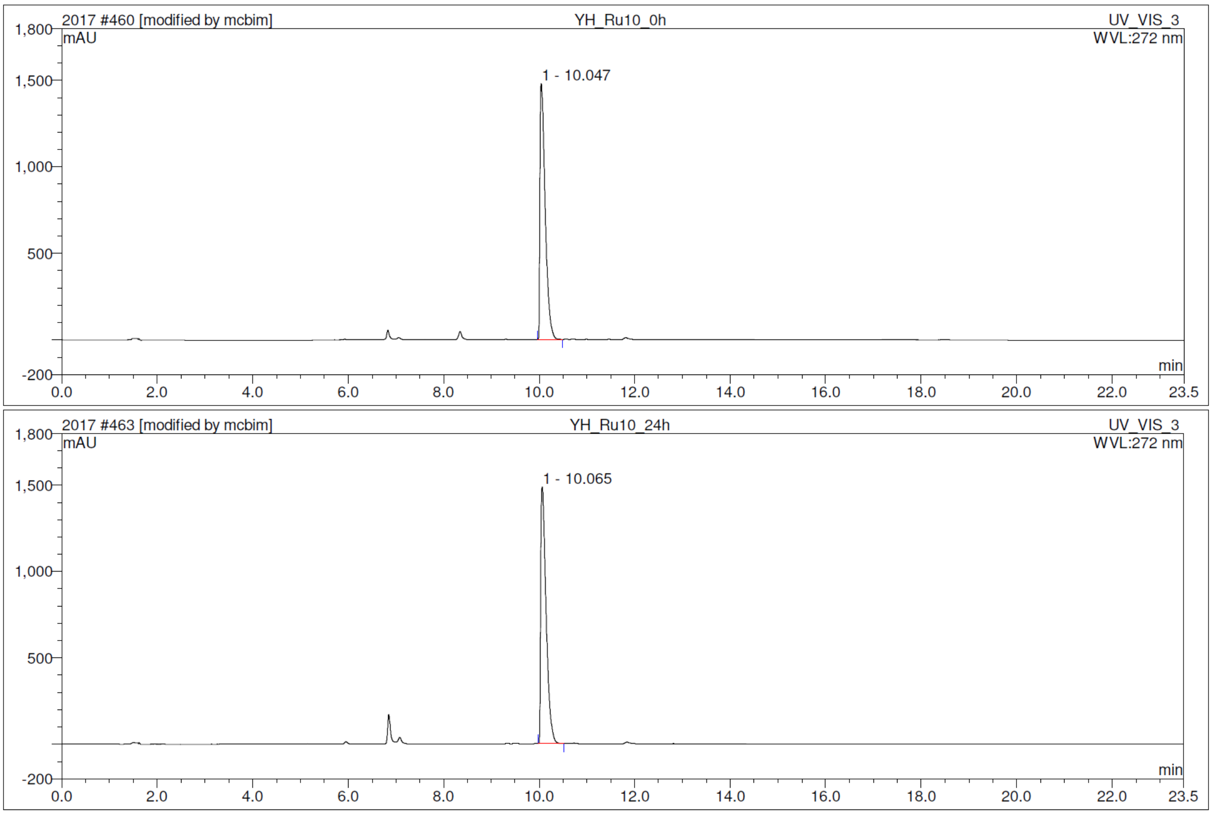

Figure S181. HPLC traces of [**10**]Cl_2 (t_R = 10.1 min) at the beginning (top) and at the end (bottom) of UV-Vis stability test indicating 3.05% reduction in relative peak area over 24 h in H_2O with 2% v/v MeOH (0.1 mM solution).

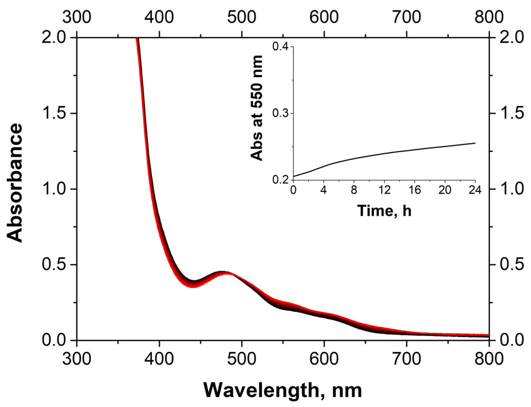

Figure S182. Evolution of the absorption spectra of [**11**]Cl_2 in H_2O with 2% v/v MeOH (0.1 mM solution) over 24 h in dark at 298 K.

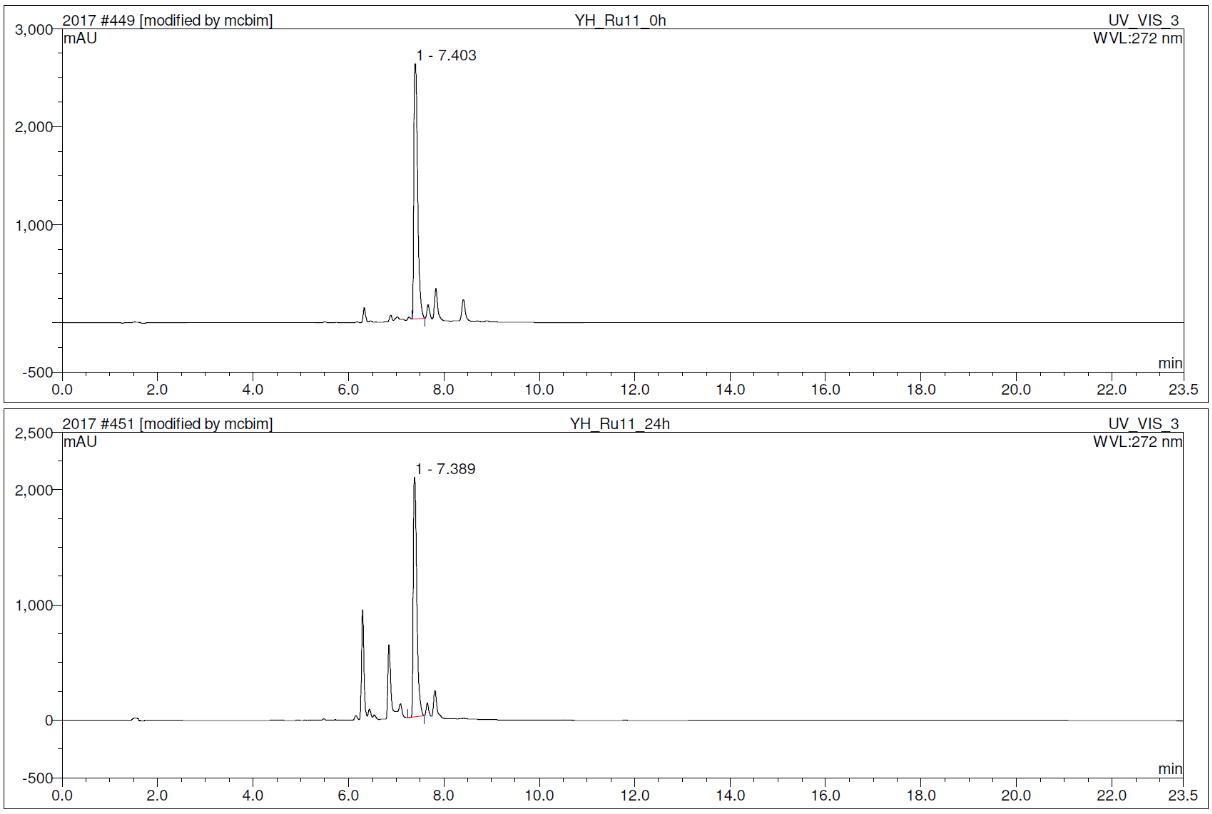

Figure S183. HPLC traces of [**11**]Cl_2 (t_R = 7.4 min) at the beginning (top) and at the end (bottom) of UV-Vis stability test indicating 21.21% reduction in relative peak area over 24 h in H_2O with 2% v/v MeOH (0.1 mM solution).

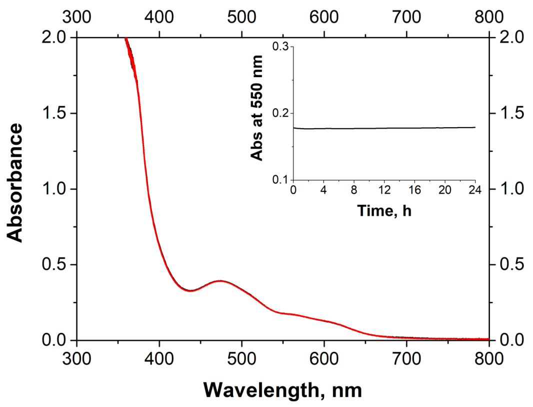

Figure S184. Evolution of the absorption spectra of [**12**]Cl_2 in H_2O with 2% v/v MeOH (0.1 mM solution) over 24 h in dark at 298 K.

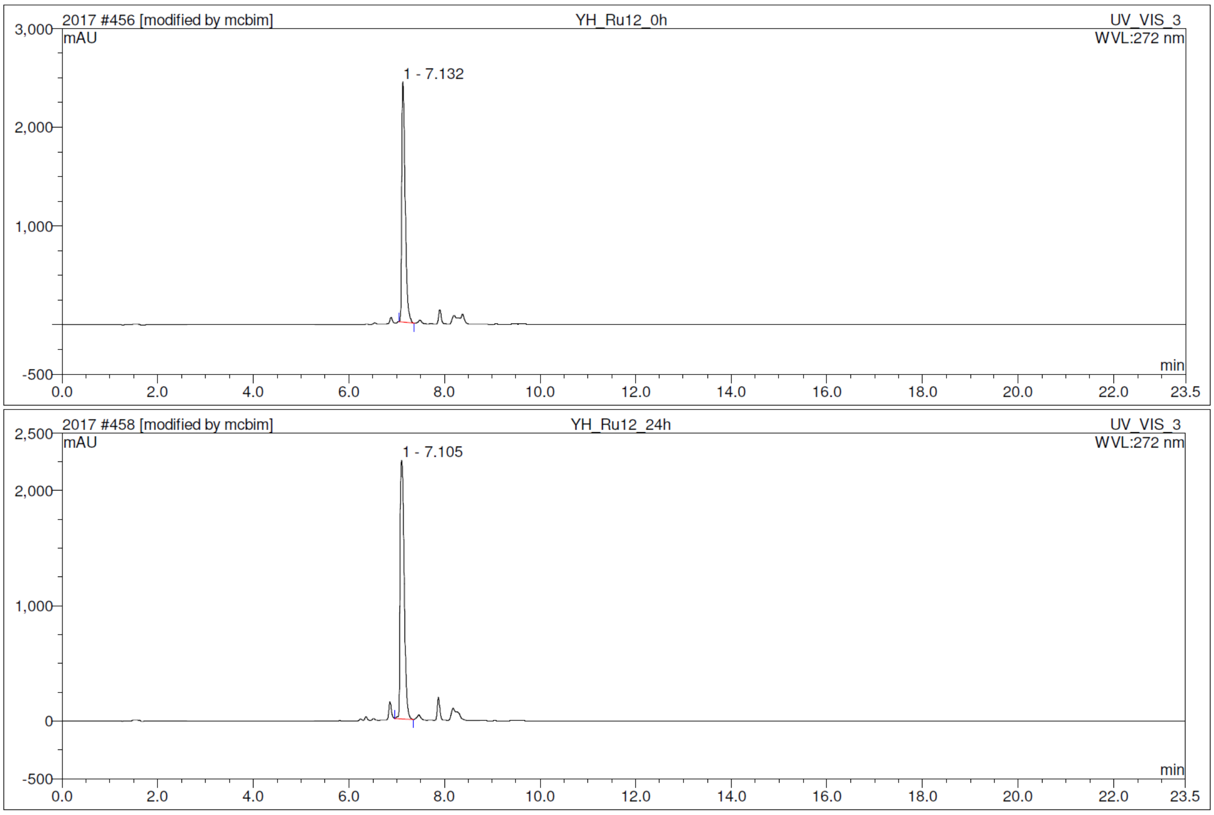

Figure S185. HPLC traces of [**12**]Cl_2 (t_R = 7.1 min) at the beginning (top) and at the end (bottom) of UV-Vis stability test indicating 7.34% reduction in relative peak area over 24 h in H_2O with 2% v/v MeOH (0.1 mM solution).

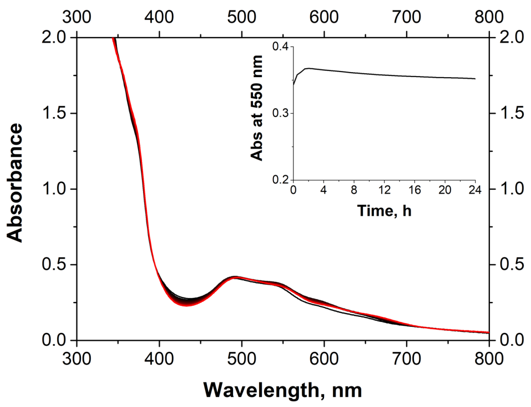

Figure S186. Evolution of the absorption spectra of [**13**]Cl_2 in H_2O with 2% v/v MeOH (0.1 mM solution) over 24 h in dark at 298 K.

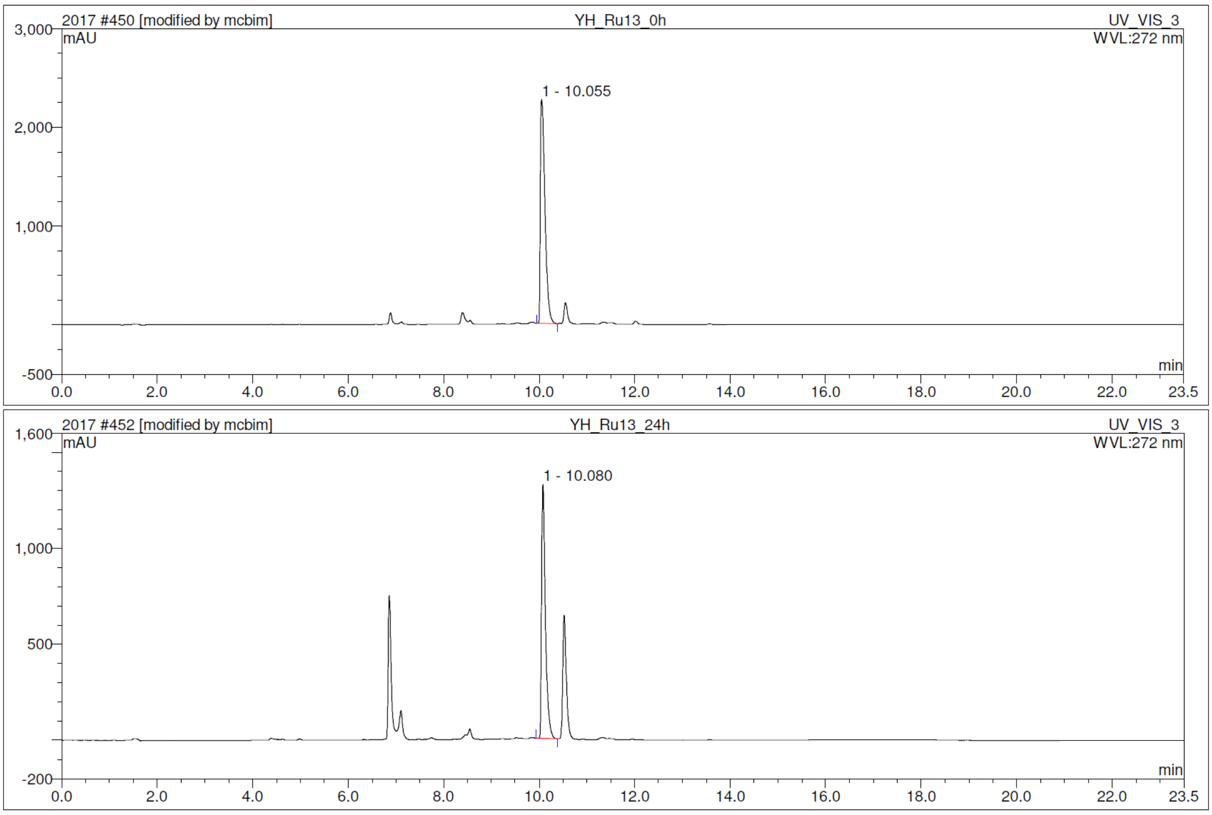

Figure S187. HPLC traces of [**13**]Cl_2 (t_R = 10.1 min) at the beginning (top) and at the end (bottom) of UV-Vis stability test indicating 34.18% reduction in relative peak area over 24 h in H_2O with 2% v/v MeOH (0.1 mM solution).

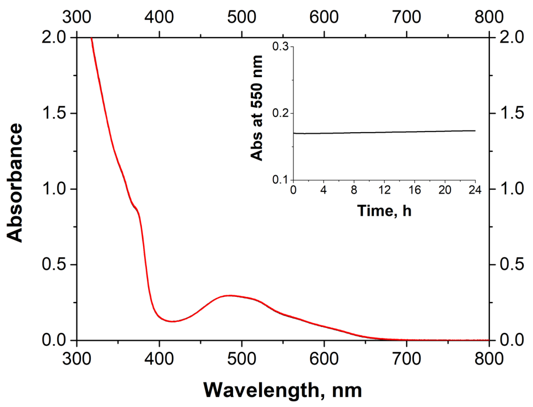

Figure S188. Evolution of the absorption spectra of [**14**]$(PF_6)_2$ in H_2O with 2% v/v MeOH (0.1 mM solution) over 24 h in dark at 298 K.

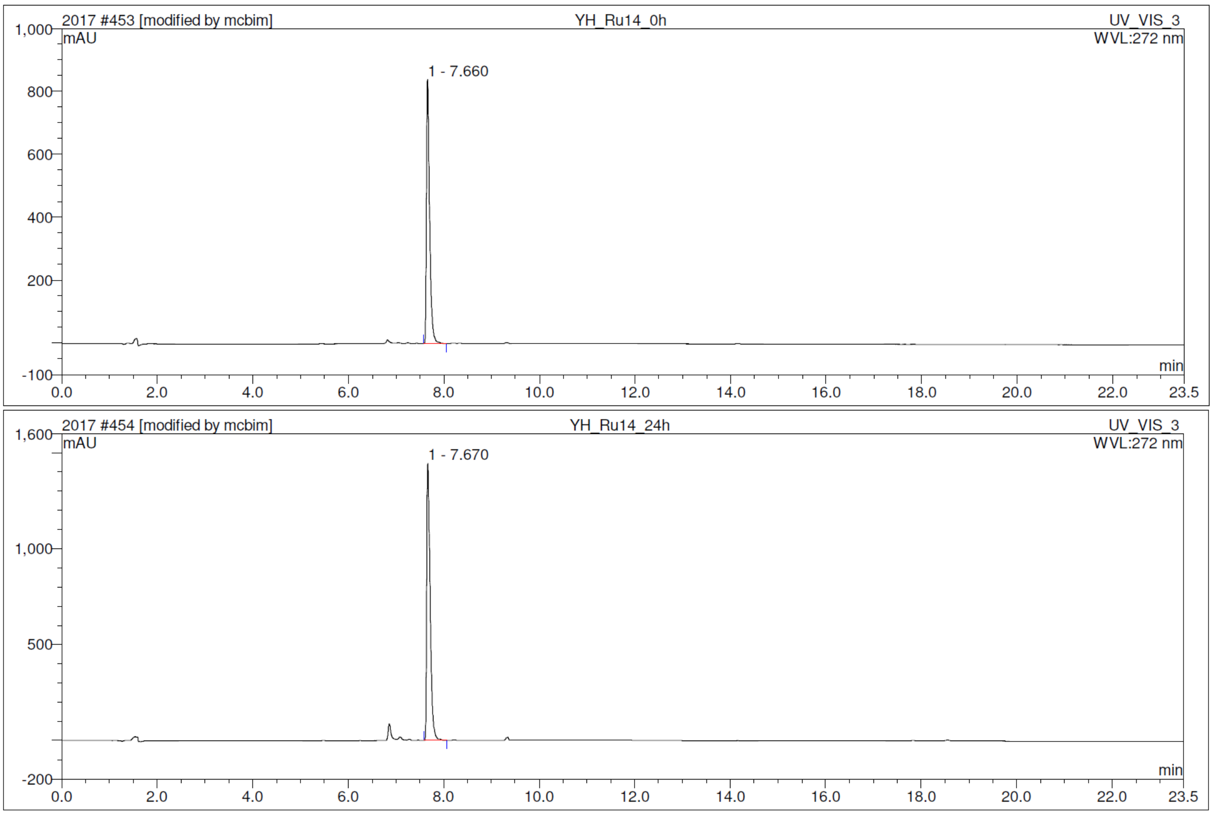

Figure S189. HPLC traces of [**14**]Cl_2 (t_R = 7.7 min) at the beginning (top) and at the end (bottom) of UV-Vis stability test indicating 5.08% reduction in relative peak area over 24 h in H_2O with 2% v/v MeOH (0.1 mM solution).

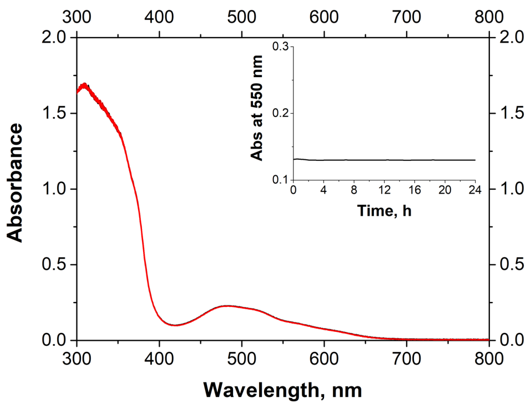

Figure S190. Evolution of the absorption spectra of [**15**]$(PF_6)_2$ in H_2O with 2% v/v MeOH (0.1 mM solution) over 24 h in dark at 298 K.

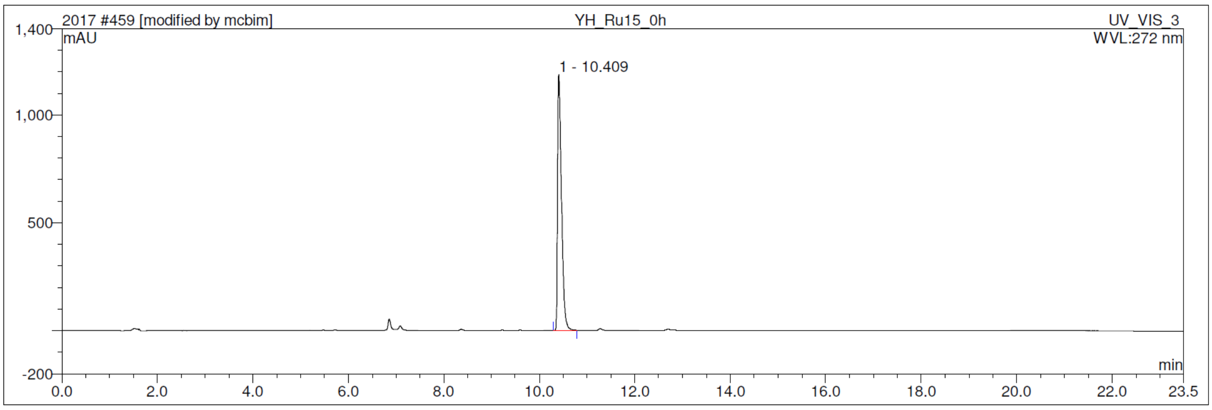

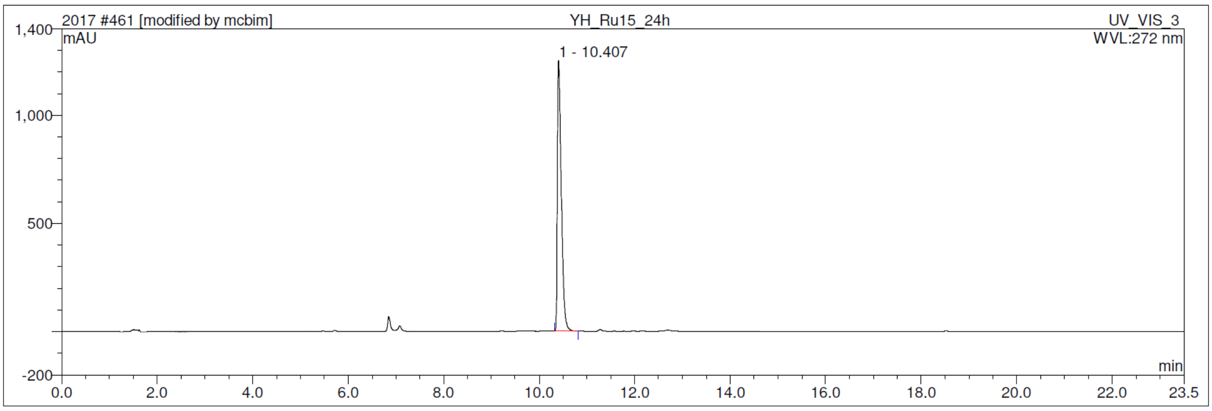

Figure S191. HPLC traces of [**15**]$(PF_6)_2$ (t_R = 10.4 min) at the beginning (top) and at the end (bottom) of UV-Vis stability test indicating 1.44% reduction in relative peak area over 24 h in H_2O with 2% v/v MeOH (0.1 mM solution).

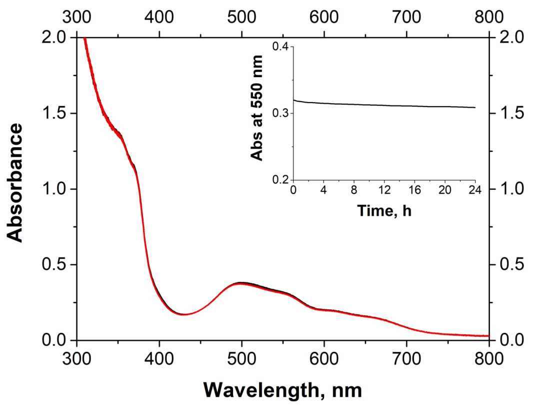

Figure S192. Evolution of the absorption spectra of [**16**]Cl_2 in H_2O with 2% v/v MeOH (0.1 mM solution) over 24 h in dark at 298 K.

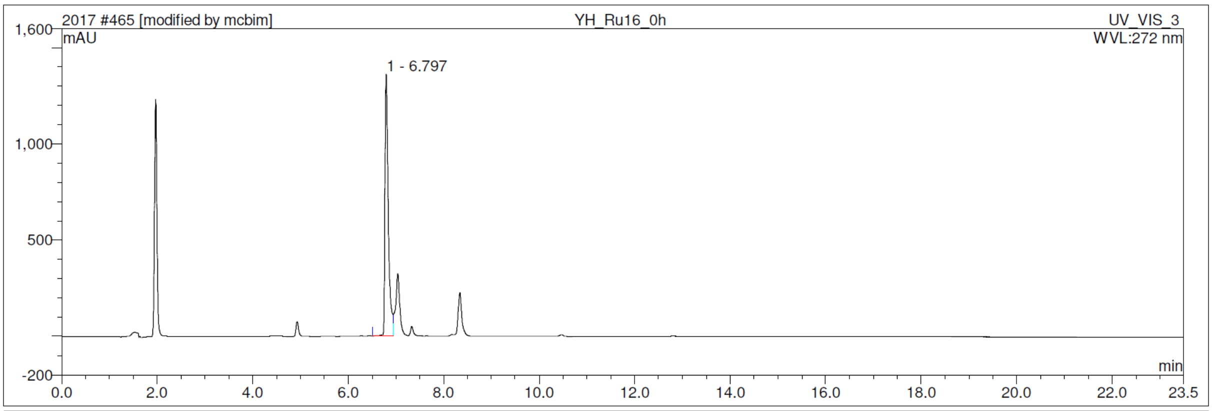

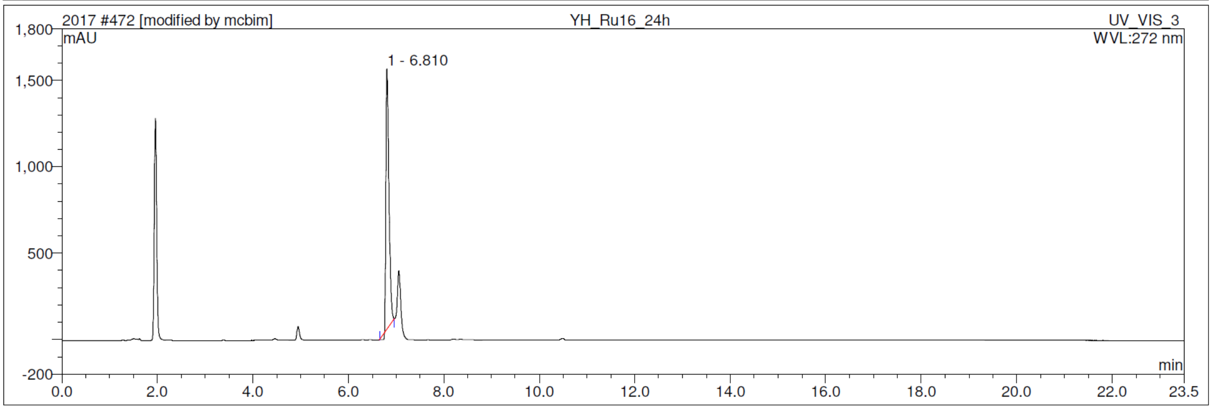

Figure S193. HPLC traces of [**16**]Cl_2 (t_R = 6.8 min) at the beginning (top) and at the end (bottom) of UV-Vis stability test indicating 6.00% reduction in relative peak area over 24 h in H_2O with 2% v/v MeOH (0.1 mM solution).

2.5 Emission spectra of the light sources used

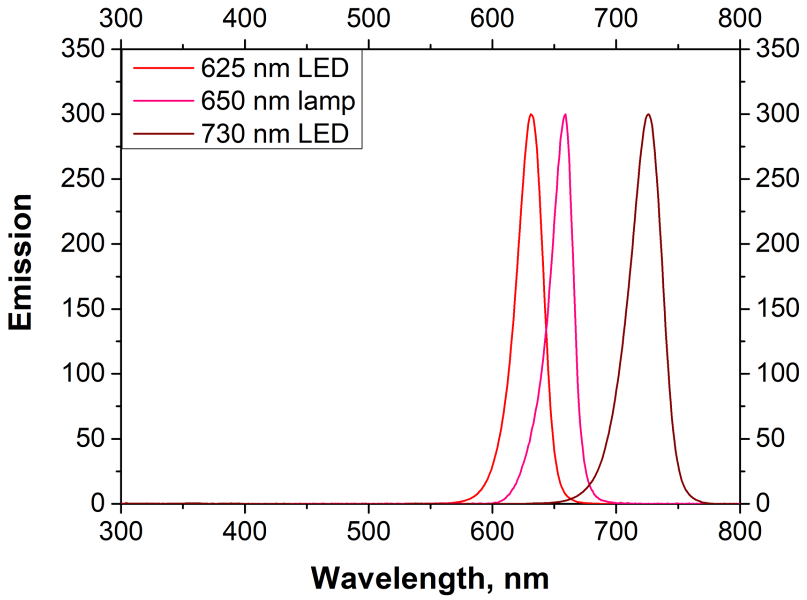

Figure S194. Normalized spectral irradiance of LEDs used for UV-Vis and NMR experiments.

2.6 Ligand photosubstitution monitored by mass spectrometry

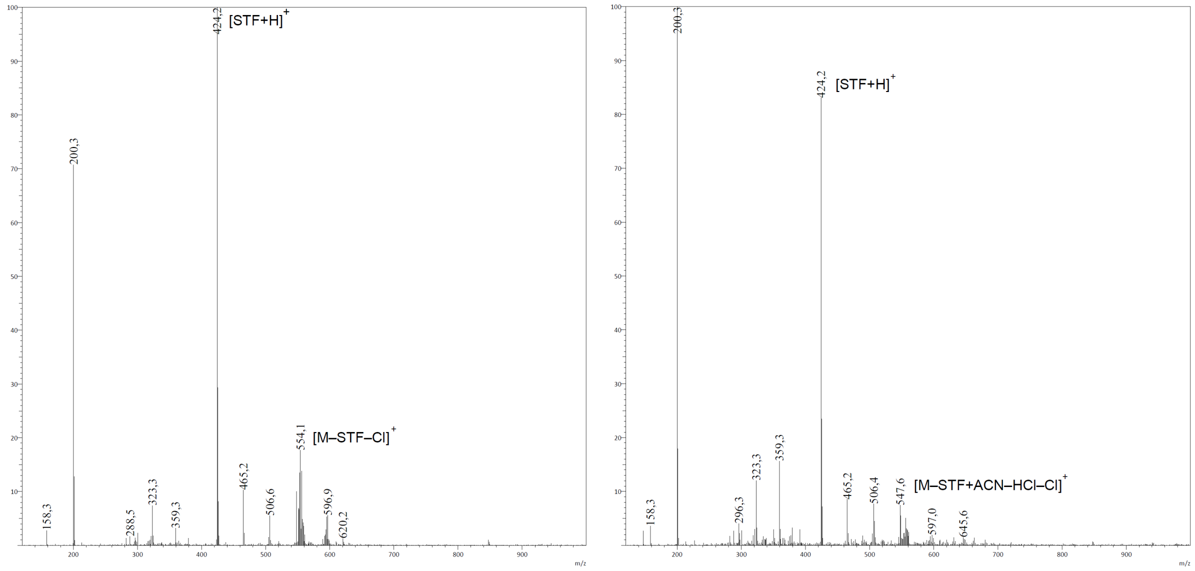

Figure S195. ESI-MS of [**7**]Cl_2 in ACN after irradiation with 625 nm (left) and 730 nm (right) light.

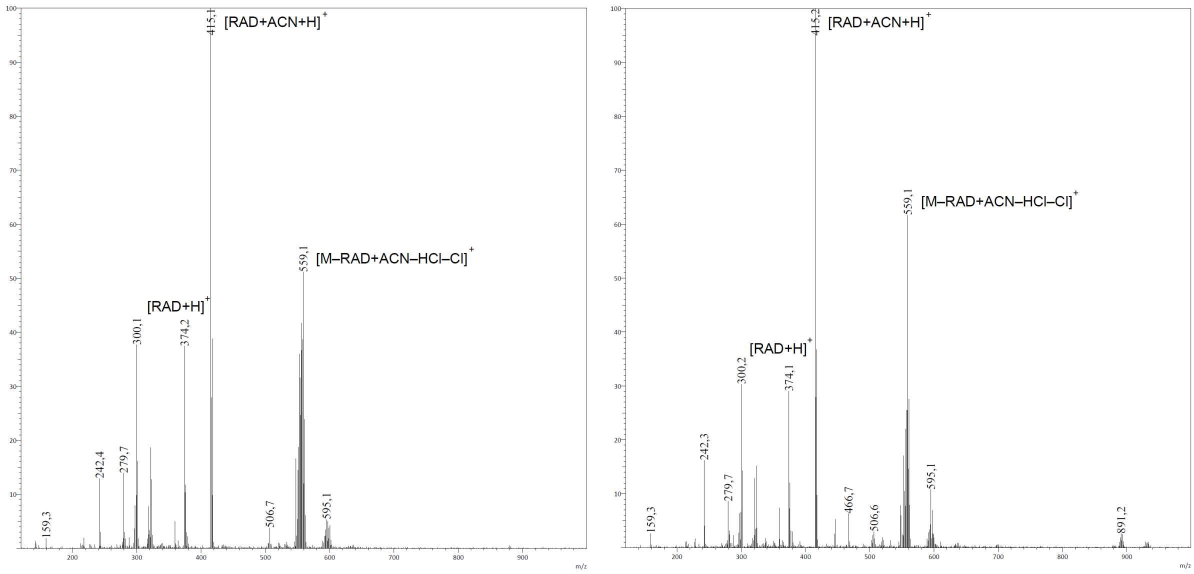

Figure S196. ESI-MS of [**8**]Cl_2 in ACN after irradiation with 625 nm (left) and 730 nm (right) light.

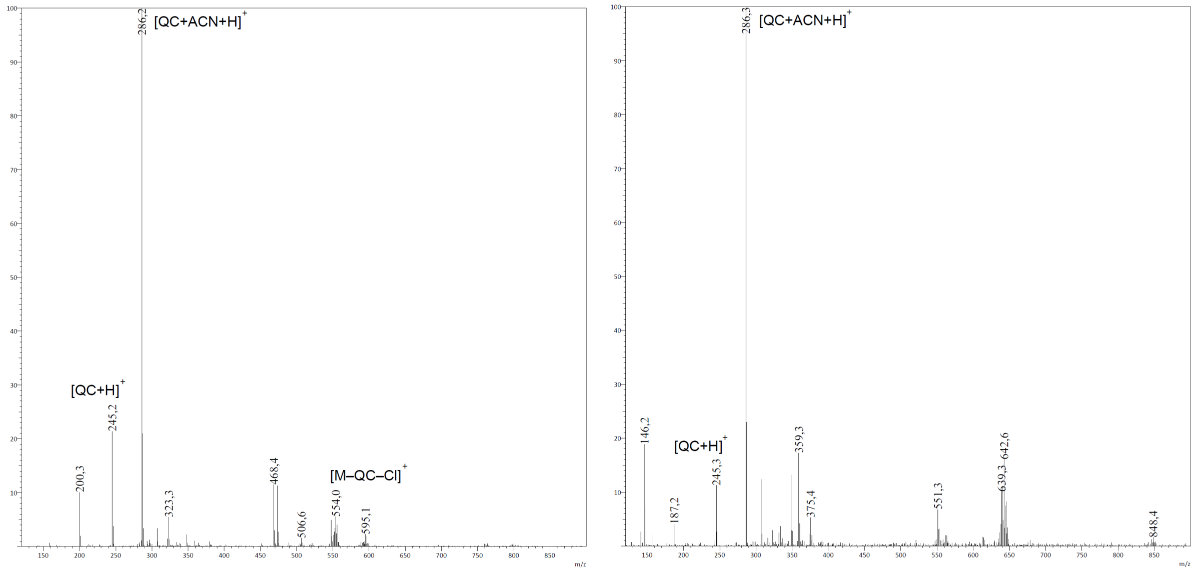

Figure S197. ESI-MS of [**9**]Cl_2 in ACN after irradiation with 625 nm (left) and 730 nm (right) light.

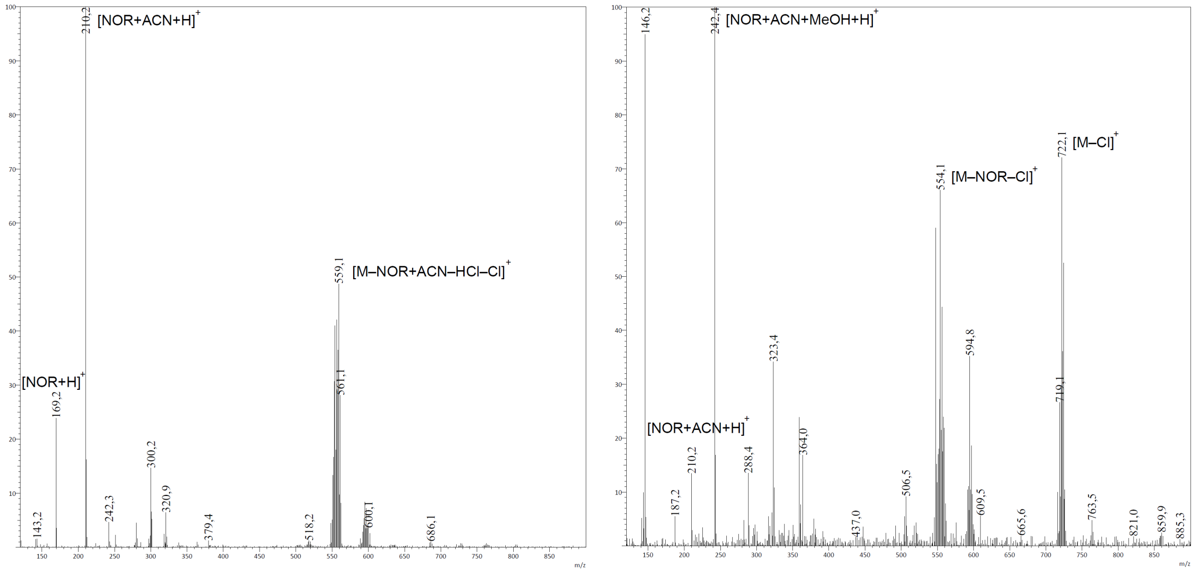

Figure S198. ESI-MS of [**10**]Cl_2 in ACN after irradiation with 625 nm (left) and 730 nm (right) light.

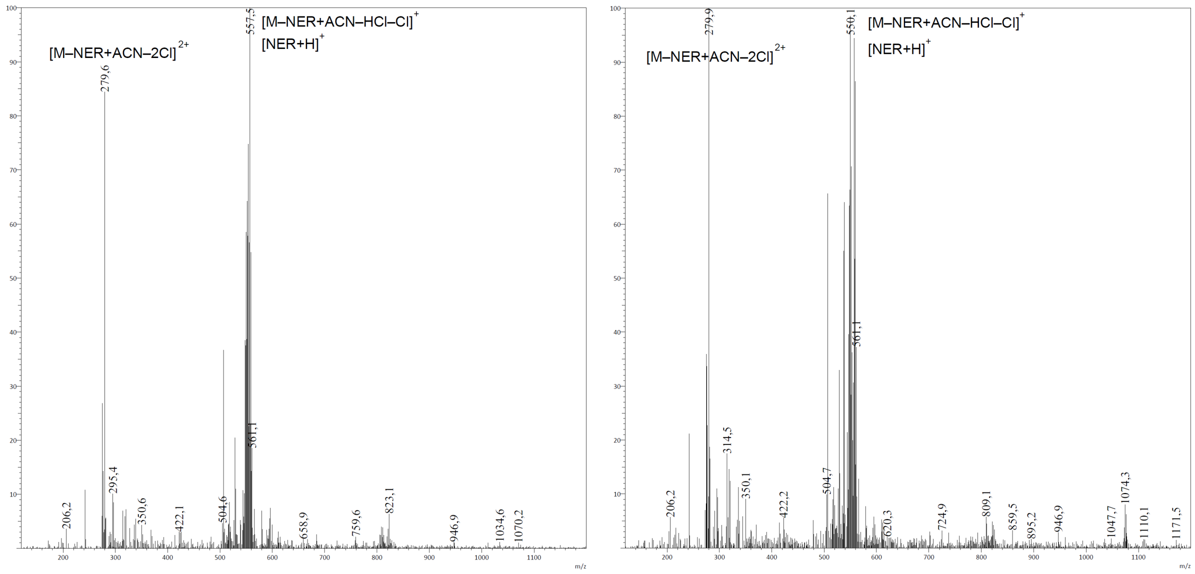

Figure S199. ESI-MS of [**11**]Cl_2 in ACN after irradiation with 625 nm (left) and 730 nm (right) light.

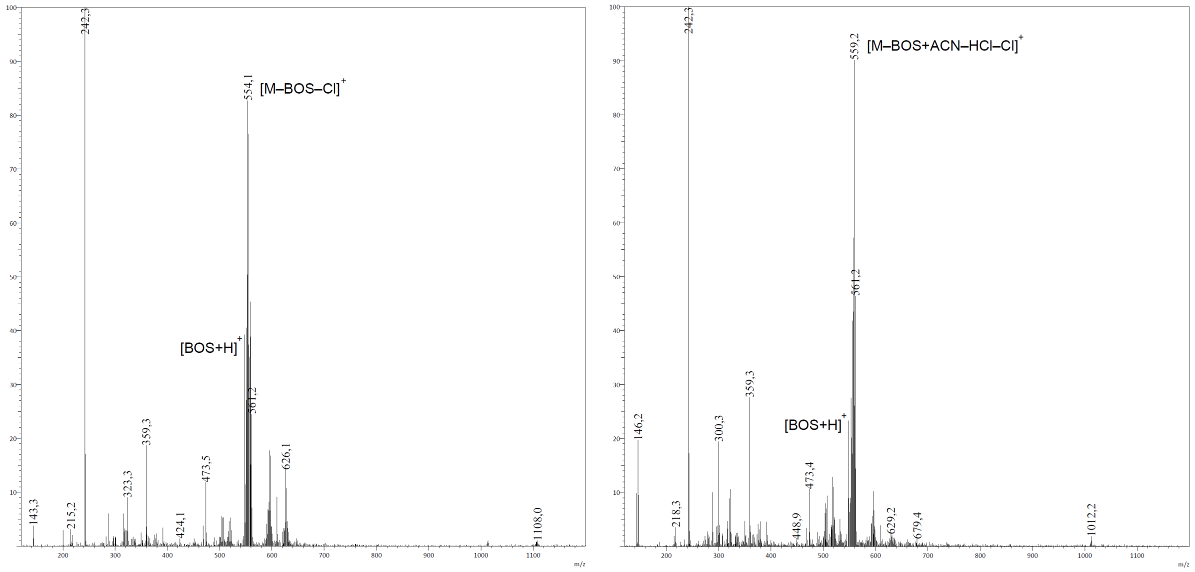

Figure S200. ESI-MS of [**12**]Cl_2 in ACN after irradiation with 625 nm (left) and 730 nm (right) light.

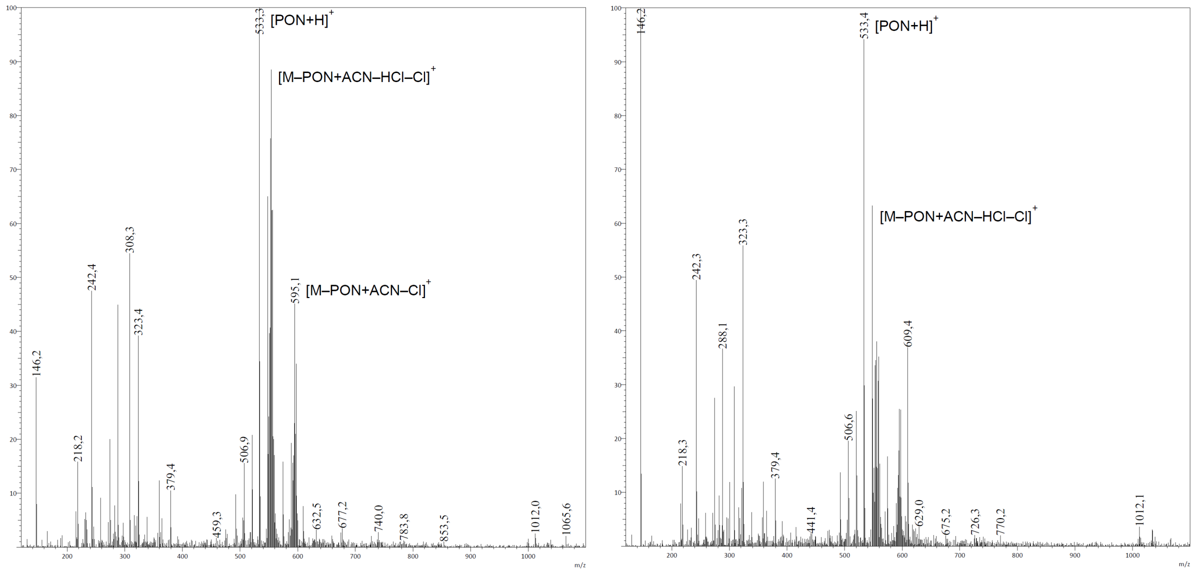

Figure S201. ESI-MS of [**13**]Cl_2 in ACN after irradiation with 625 nm (left) and 730 nm (right) light.

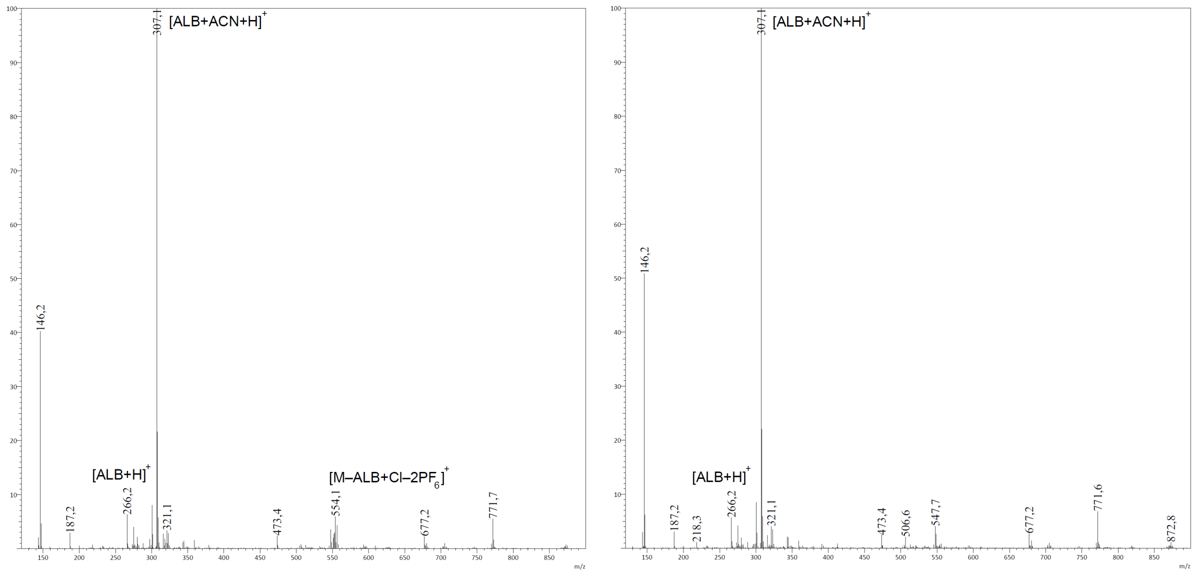

Figure S202. ESI-MS of [**14**](PF_6)$_2$ in ACN after irradiation with 625 nm (left) and 730 nm (right) light.

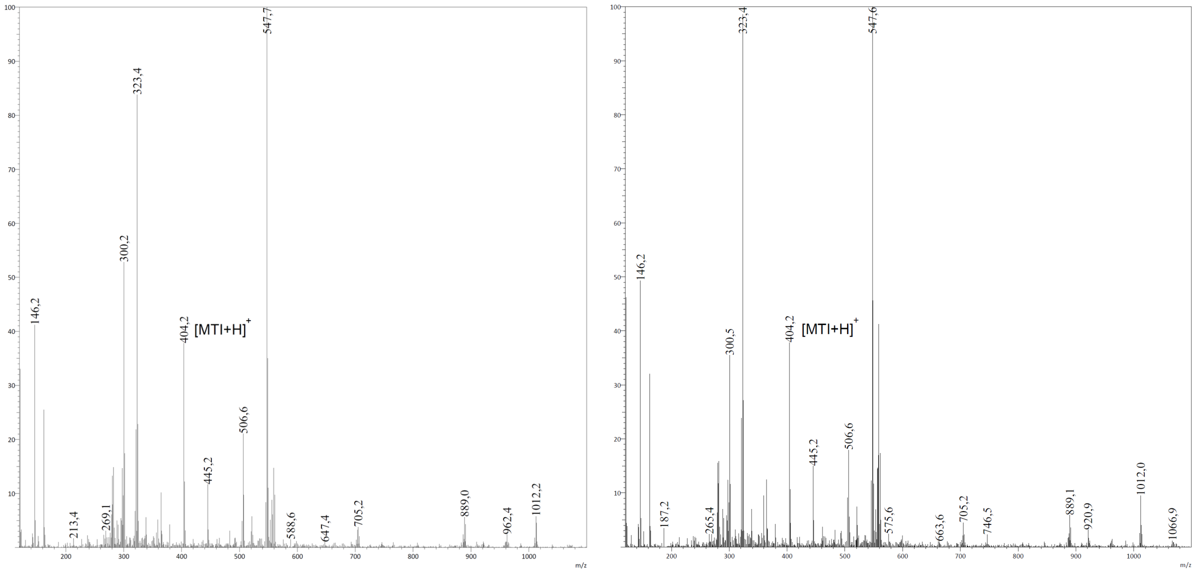

Figure S203. ESI-MS of [**15**](PF_6)$_2$ in ACN after irradiation with 625 nm (left) and 730 nm (right) light.

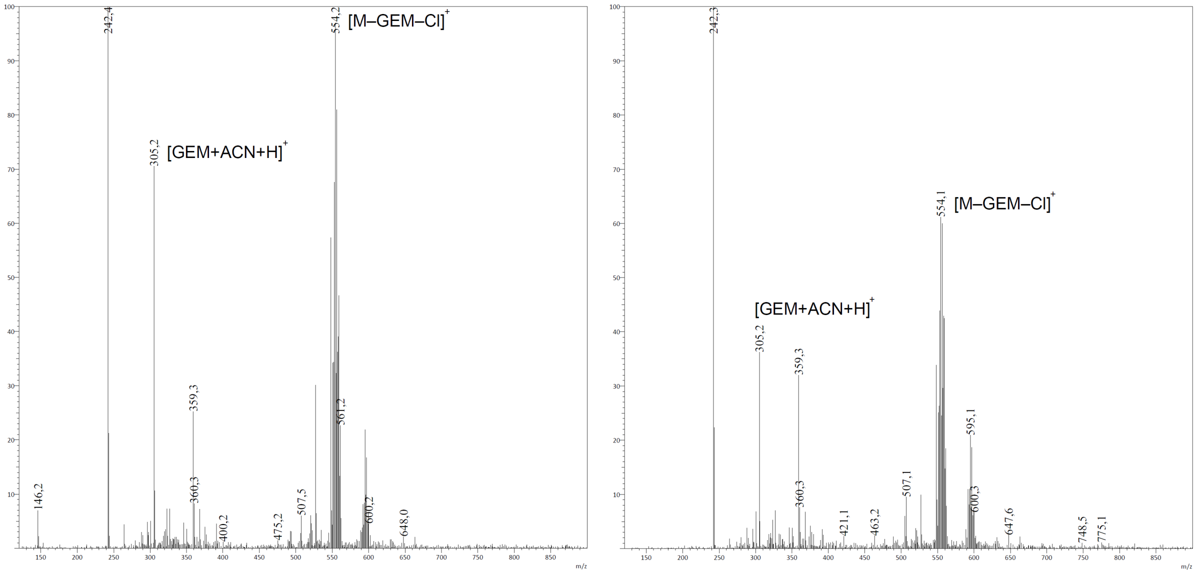

Figure S204. ESI-MS of [**16**]Cl_2 in ACN after irradiation with 625 nm (left) and 730 nm (right) light.

2.7 Phosphorescence data

The steady-state emission measurements were performed on a previously reported custom-built setup utilizing a slightly modified experimental procedure, results are presented in **Table S17** and **Figure S205** below.[12] $[Ru(bpy)_3]Cl_2$ was used as a reference with reported $\Phi_P = 0.018 \pm 0.002$ in air-saturated ACN.[13] All the compounds were dissolved in 3 mL of ACN and transferred into a macro fluorescence cuvette from Hellma Analytics (lightpaths: 1 cm x 1 cm). The irradiation of samples was done at 298 K using 450 nm LRD-0450 Laserglow fiber-coupled laser set to 80 mW at the cuvette with help of PM100USB Thorlabs power meter. The UV-Vis and emission spectra were recorded at 298 K with Agilent Cary 60 UV-Vis and Avantes 2048L StarLine spectrometers respectively. The emission spectrums were acquired within 100 ms. All the spectral data was processed with OriginPro 9.1 and MS Excel 2016.

Table S17. Determination of phosphorescence quantum yields in ACN.

Compound	Absorbance at 450 nm	Integrated emission	Φ_P
$[Ru(bpy)_3]Cl_2$	0.1022	938.9151	0.01800
[**4**]Cl	0.0960	0.7913	0.00002
[**6**]Cl_2	0.1049	2.2191	0.00004
[**7**]Cl_2	0.1039	1.2398	0.00002
[**8**]Cl_2	0.1047	2.2843	0.00004
[**9**]Cl_2	0.1058	1.5292	0.00003
[**10**]Cl_2	0.1035	3.2865	0.00006
[**11**]Cl_2	0.1048	5.7685	0.00011
[**12**]Cl_2	0.0979	0.8363	0.00002
[**13**]Cl_2	0.1023	8.1644	0.00016
[**14**]$(PF_6)_2$	0.1031	4.8433	0.00009
[**15**]$(PF_6)_2$	0.1037	3.9487	0.00007

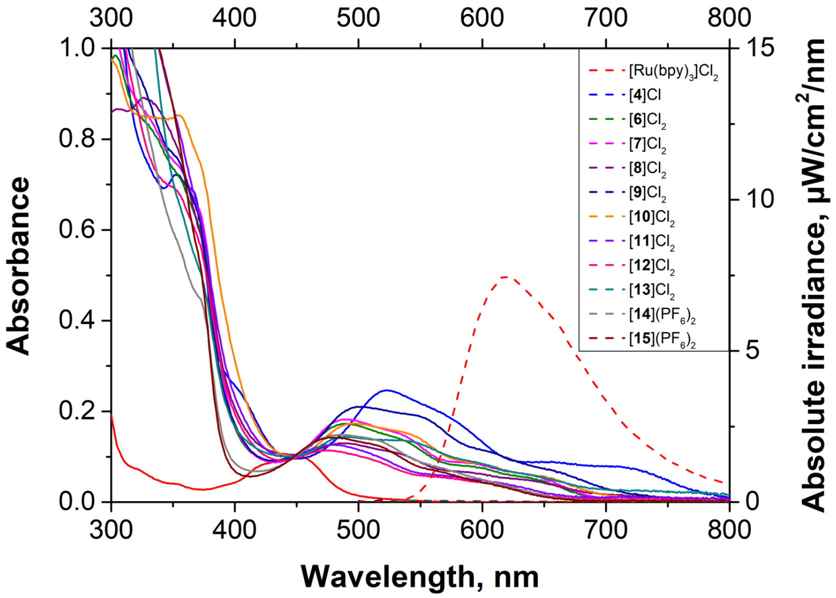

Figure S205. Absorption (solid) and emission (dash) spectrums of compounds from **Table S17**, the absorption at 450 nm was adjusted to 0.1 for all of them.

2.8 Singlet oxygen measurement

The steady-state singlet oxygen emission measurements were performed on a previously reported custom-built setup utilizing a slightly modified experimental procedure, results are presented in **Table S18** and **Figure S206-S208** below.[12,14,15] Perinaphthenone was used as a reference with reported Φ_Δ ($^1O_2$) = 0.98 ± 0.07 in air-saturated ACN.[16] $[Ru(bpy)_3]Cl_2$ was used as validation compound with reported Φ_Δ ($^1O_2$) = 0.57 ± 0.06 in air-saturated ACN.[17] The NIR spectra were acquired within 20 s at 298 K with Avantes NIR256-1.7TEC spectrometer. Other conditions were similar to those described in the phosphorescence section above.

Table S18. Determination of singlet oxygen quantum yields in ACN.

Compound	Absorbance at 450 nm	Integrated emission	Φ_Δ
Perinaphthenone	0.0957	0.2270	0.980
$[Ru(bpy)_3]Cl_2$	0.1045	0.1473	0.583
[**4**]Cl	0.1023	0.0007	0.003
[**6**]Cl_2	0.1014	0.0003	0.001
[**7**]Cl_2	0.1039	0.0007	0.003
[**8**]Cl_2	0.1079	0.0019	0.007
[**9**]Cl_2	0.1003	0.0009	0.004
[**10**]Cl_2	0.1032	0.0011	0.004
[**11**]Cl_2	0.1077	0.0010	0.004
[**12**]Cl_2	0.1032	0.0005	0.002
[**13**]Cl_2	0.0972	0.0013	0.006
[**14**]$(PF_6)_2$	0.1034	0.0012	0.005
[**15**]$(PF_6)_2$	0.1009	0.0008	0.003

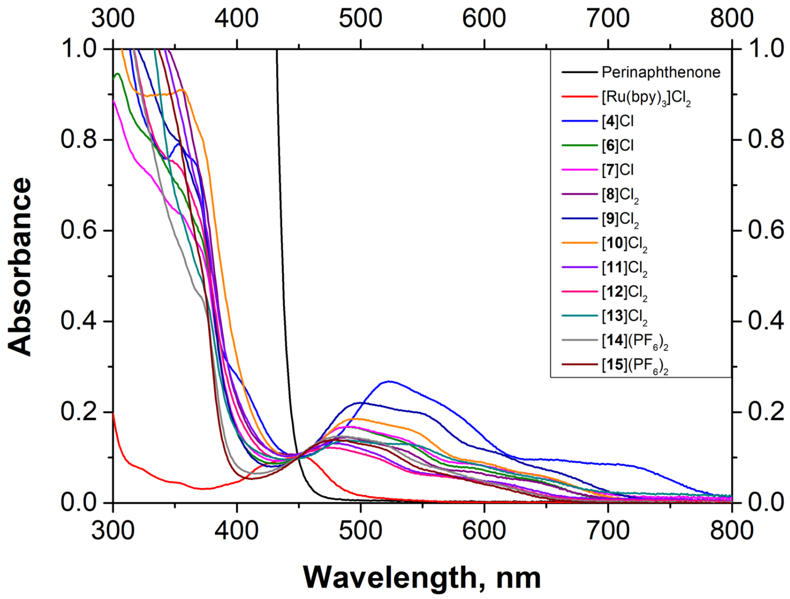

Figure S206. UV-Vis spectra of compounds from **Table S18** in ACN, the absorption at 450 nm was adjusted to 0.1 for all of them.

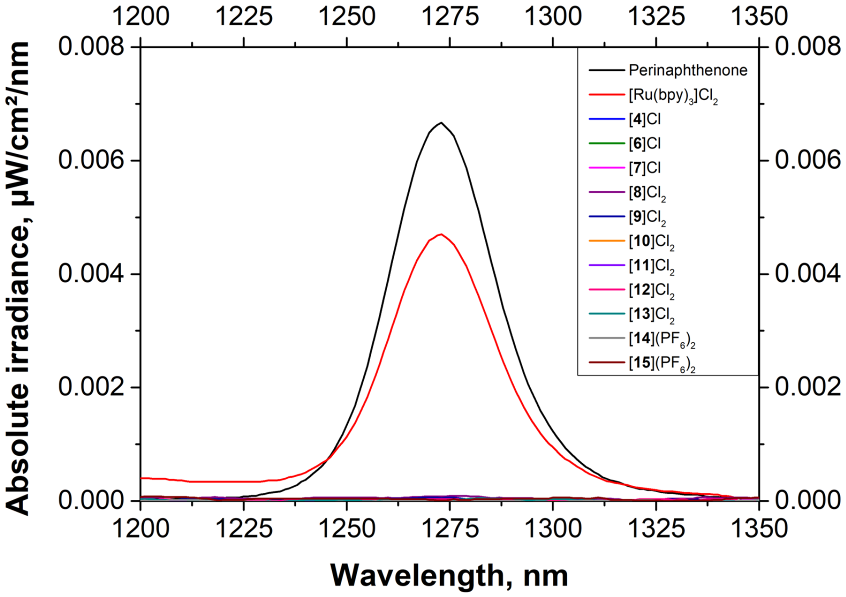

Figure S207. Raw NIR emission spectra of compounds from **Table S18** in ACN.

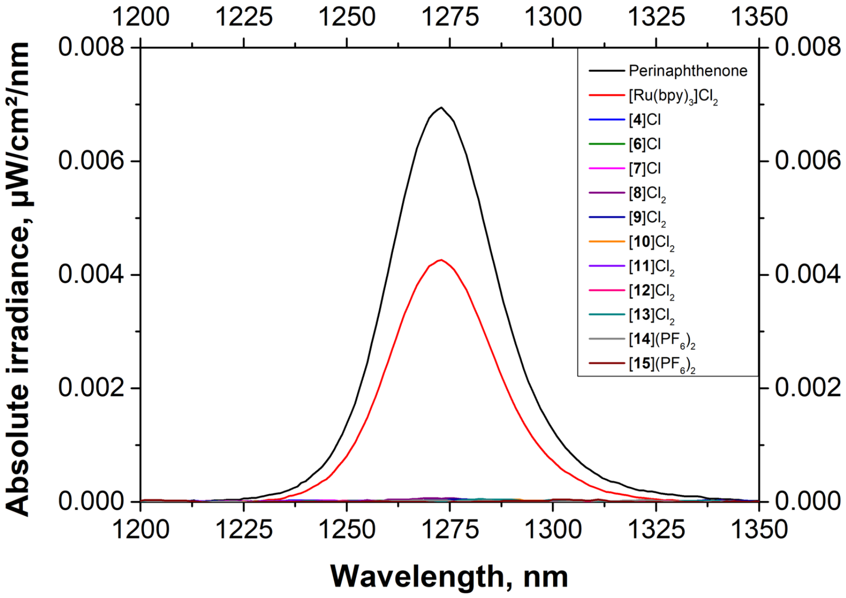

Figure S208. Normalized NIR emission spectra of compounds from **Table S18** in ACN for proper graphical comparison (backgrounds were subtracted and the emission intensities were adjusted as if compounds absorbance at 450 nm was exactly 0.1).

3 Photocytotoxicity

3.1 General information

DMEM (High Glucose (4.5 g/L), with phenol red and sodium pyruvate, without L-glutamine) was obtained from Capricorn Scientific, Gibco Opti-MEM Reduced Serum Medium (with L-glutamine and HEPES, without phenol red) from Thermo Fisher Scientific and DPBS Buffered Solution (without calcium and magnesium chloride) from Sigma-Aldrich. Human cancer cell line A375 was distributed by the European Collection of Cell Cultures (ECACC) and purchased from Sigma-Aldrich. The glioblastoma cells U-87MG cells were purchased from ATCC (American Type Culture Collection, Manassas, Virginia, US). Fetal calf serum (FCS), trypsin, D-mannitol, L-glutamine and **Cisplatin** were purchased from Sigma-Aldrich. Penicillin/streptomycin (P/S) were purchased from Duchefa Biochemie. Trypan blue (0.4% in 0.81% sodium chloride and 0.06% potassium phosphate dibasic solution) was purchased from Bio-Rad Laboratories. Thiazolyl blue tetrazolium bromide (MTT) was obtained from Bio-Connect. T-25 and T-75 cell culture flasks were purchased from Sarstedt and Cellstar-96-well plates from Greiner Bio-One and Sarstedt. Cells were counted using a BioRad TC20 automated cell counter with BioRad Cell Counting Slides. UV-Vis measurements for analysis of 96-well plates were performed on a M1000 Tecan Plate Reader. Cells were inspected with an Olympus IX81 microscope. All the compounds for cell experiments were weighted using Sartorius Cubis II (MCA) Micro Balance. The raw cytotoxicity data was processed using Microsoft Office Excel 2016 and GraphPad Prism 9. The values are expressed as the mean ± standard error of the mean (SEM) of separate experiments. Statistical significance was evaluated by the two-tailed unpaired Student's t-test and expressed as follows (ns): $p > 0.05$, (*): $p \leq 0.05$, (**): $p \leq 0.01$, (***): $p \leq 0.001$, (****): $p \leq 0.0001$.

3.2 Cell culturing

Cells were thawed and at least passaged twice before starting cytotoxicity experiments. The A375 cells were cultured in T-25 flasks in DMEM-complete (DMEM supplemented with 10% v/v FCS, 0.2% v/v P/S, and 1% v/v L-glutamine) and split at 70-80% confluence two times per week (seeding density: 40000 cells/mL, 6 mL per flask). The U-87MG cells were cultured in T-75 flasks in DMEM-complete and split at 70-80% confluence two times per week (seeding density: 35000 cells/mL, 15 mL per flask). The normoxic cells were incubated in the dark at 21% O_2, 5% CO_2 and 37 °C in PHCbi O_2/CO_2 MCO-170M Multigas Incubator. Cells used in all biological experiments were cultured for a maximum of eight weeks.

3.3 Cytotoxicity assay

The photocytotoxicity of prepared compounds in A375 and U-87MG cancer cell lines were assessed using formerly described assay.[18] In short, once at 70-80% confluence the medium from cultured A375 cells was removed and the residue was rinsed with 2 mL of PBS. The obtained cells were trypsinized, mixed with 5 mL of DMEM-complete and transferred to a centrifuge tube (4 min, 0.3 relative centrifugal force (RCF)). After centrifuging the pellet of cells was separated from the medium and resuspended in 5 mL of Opti-MEM complete (Opti-MEM supplemented with 2.5% v/v FCS, 0.2% v/v P/S, and 1% v/v L-glutamine). The cells were then counted using tpypan blue and BioRad TC20 automated cell counter with BioRad Cell Counting Slides (usual numbers were 10^6 cells/mL). The resulting suspension was then diluted to 40000 cells/mL (60000 cells/mL for U-87MG) with Opti-MEM complete and

96-well plates were seeded 4000 cells/well (6000 cells/well for U-87MG) using electronic multichannel pipette leaving outer layer cells-free filled with 0.2 mL of Opti-MEM complete alone. The obtained plates were placed inside the previously mentioned normoxic incubator for 24 h.

The next day (t = 24 h) compounds to be tested were weighted on micro balance in 1.5 mL Eppendorf tubes, then dissolved in sterile DMSO and diluted with Opti-MEM complete to form 0.2 mM stock solutions. The maximum amount of DMSO used per well was below 0.4% v/v. The experiments with A375 (U-87MG) were all conducted in range between 10 nM to 100 µM (1 µM to 200 µM) with 8 (6) concentrations tested in total (three technical for each of three biological replicates for both cell lines). Additionally, each plate contained wells with DMSO negative control (0.4% v/v per well) and **Cisplatin** positive control (10 µM per well), the respective solution in Opti-MEM complete was prepared from an aqueous stock based on clinical formulation (3.3 mM **Cisplatin**, 55 mM mannitol, 154 mM NaCl). For every plate to be irradiated a control dark plate was also prepared in parallel and treated identically but without irradiation (**Figure S209**). After all the compounds were added the plates were further incubated for 24 h in a dark.

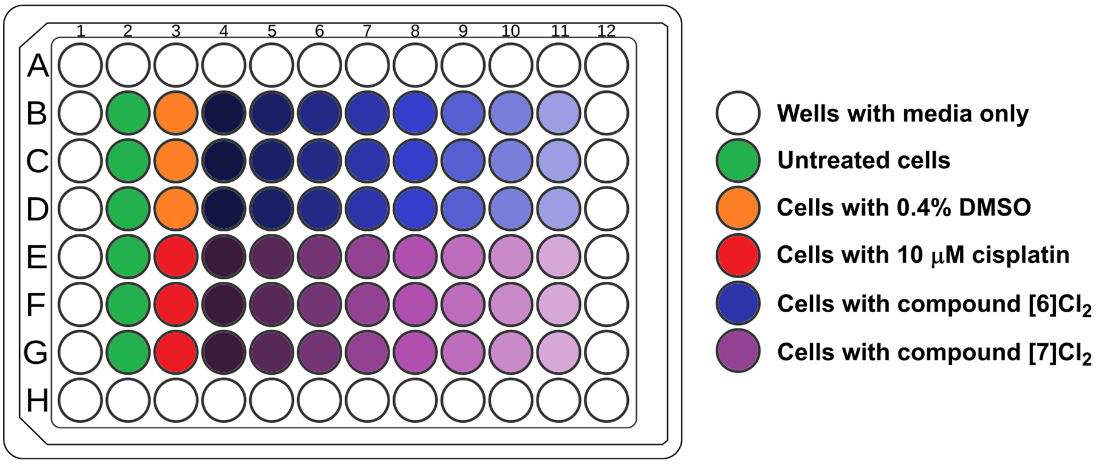

Figure S209. Example of 96-well plate design for measuring photocytotoxicity of [**6**]Cl_2 and [**7**]Cl_2. Two plates were prepared for each replicate with one being irradiated by red light and the other kept in dark.

On the third day (t = 48 h) the irradiation was performed on a previously reported custom-built setup utilizing the same experimental procedure.[18] The system consisted of a thermostat (Ditabis or Hettich) fitted with two flat-bottom microplate thermoblocks and a 96-LED array fitted to a standard 96-well plate. The LED array was powered by BK Precision BK1550 Digital Bench Power Supply (36 V, 3 A, 1-Output, 108 W). The irradiation time with 630 nm LED array (34.1 mW/cm^2) was 30 min and the respective light dose was determined to be 61.4 J/cm^2; with 730 nm LED array (63.2 mW/cm^2): 30 min, 114 J/cm^2.

At last, on the fifth day (t = 96 h) the MTT end-point assay was performed utilizing a common procedure.[19] In short, solution of 3-(4,5-dimethylthiazol-2-yl)-2,5-diphenyltetrazolium bromide in PBS (5 mg/mL) was added to all 96-well plates (20 µL per well, final concentration 0.5 mg/mL) followed by incubation for 3 h. Upon completion the media was gently removed by suction and replaced with DMSO (0.2 mL per well) to solubilize the formed formazan salt. The resulting samples were shaken for 10 min till complete dissolution and measured with a plate reader at 570 nm. The respective cells viabilities were calculated by former procedure[19,20] and the results are presented by dose-response curves (two parametric hill-slope evaluation) and independent T-tests below. For every experiment it was ensured that neither 0.4% DMSO or light itself causing any significant reduction in cells population.

630 nm red light irradiation in A375 cell line

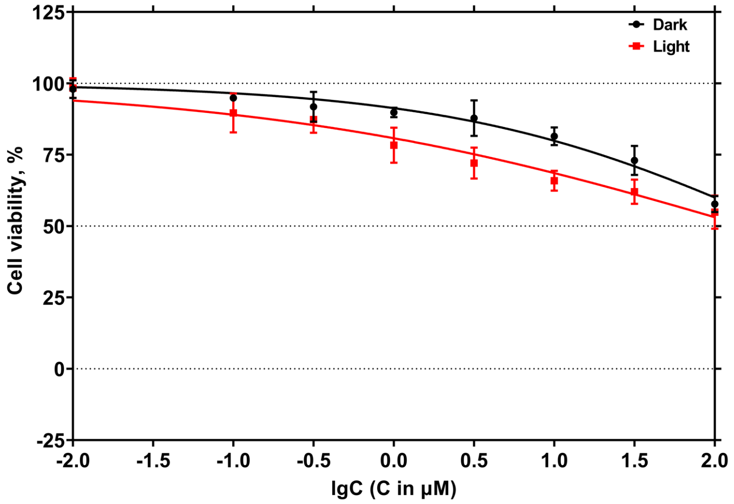

Figure S210. Dose response curves for [**6**]Cl_2 in the dark and upon 630 nm red-light irradiation in A375 cell line.

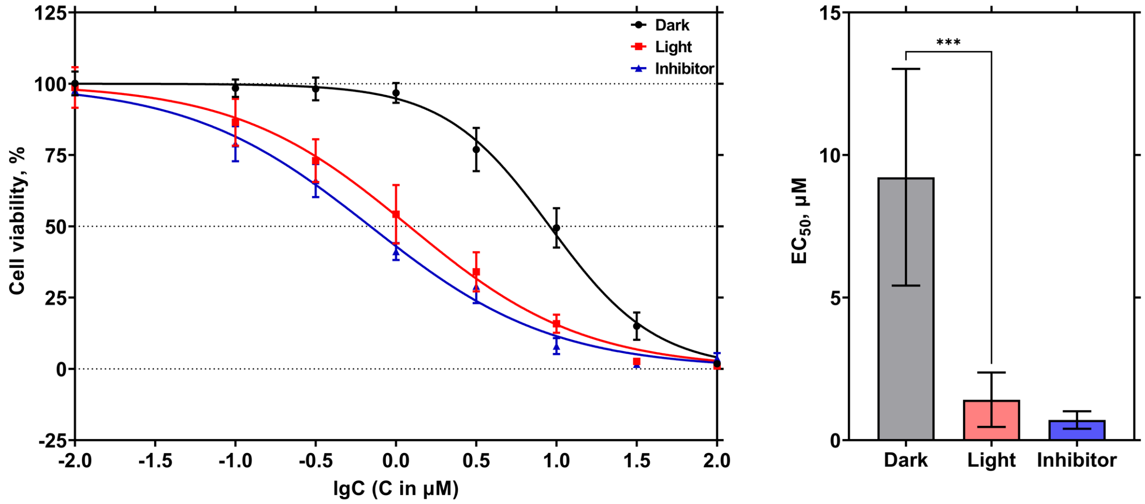

Figure S211. Dose response curves (left) and respective EC_{50} values (right) for **STF-31** itself and [**7**]Cl_2 in the dark and upon 630 nm red-light irradiation in A375 cell line.

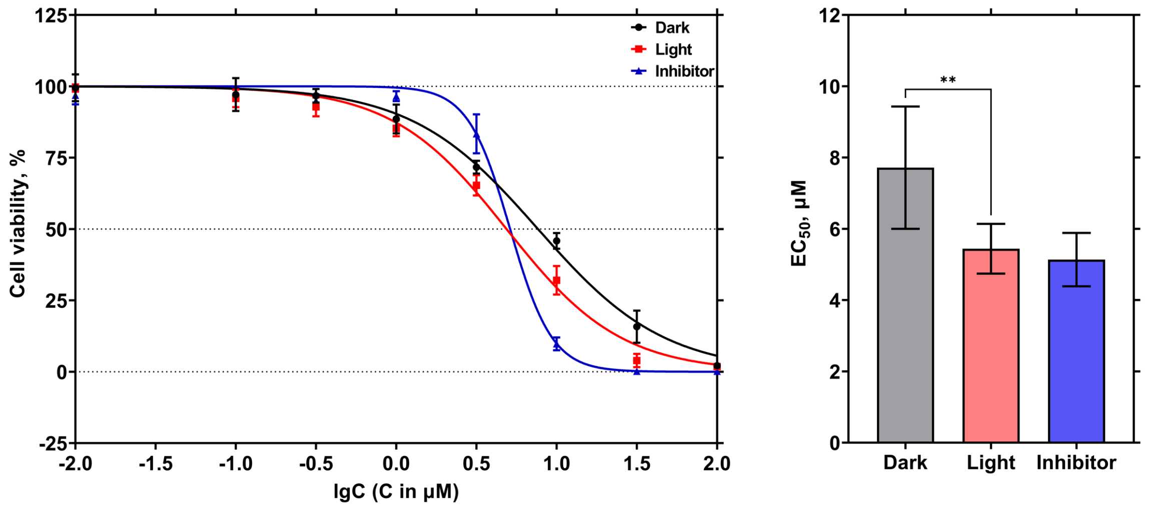

Figure S212. Dose response curves (left) and respective EC_{50} values (right) for **RAD-51-IN-1** itself and [**8**]Cl_2 in the dark and upon 630 nm red-light irradiation in A375 cell line.

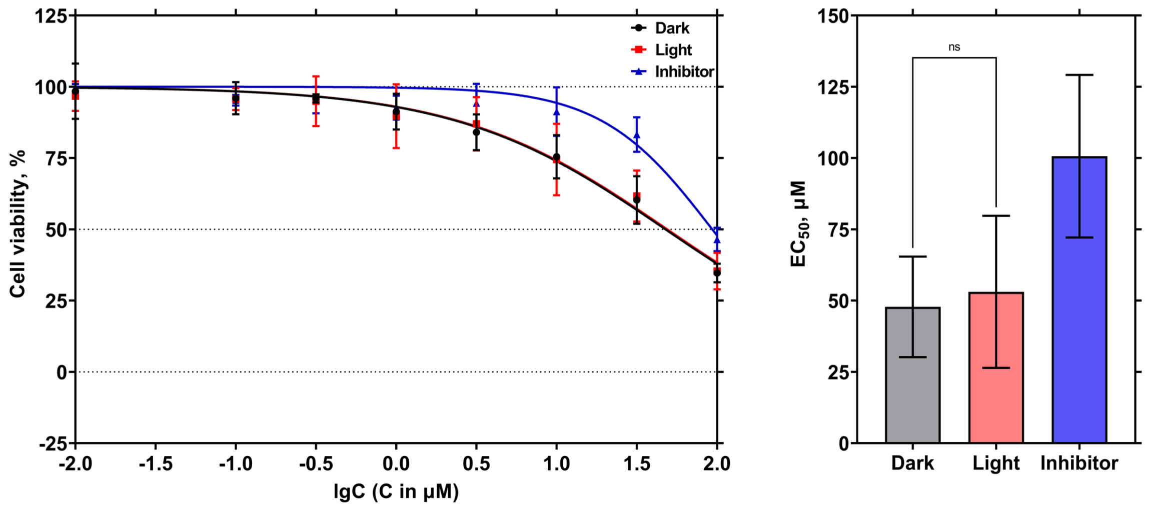

Figure S213. Dose response curves (left) and respective EC_{50} values (right) for **QC-82** itself and [**9**]Cl_2 in the dark and upon 630 nm red-light irradiation in A375 cell line.

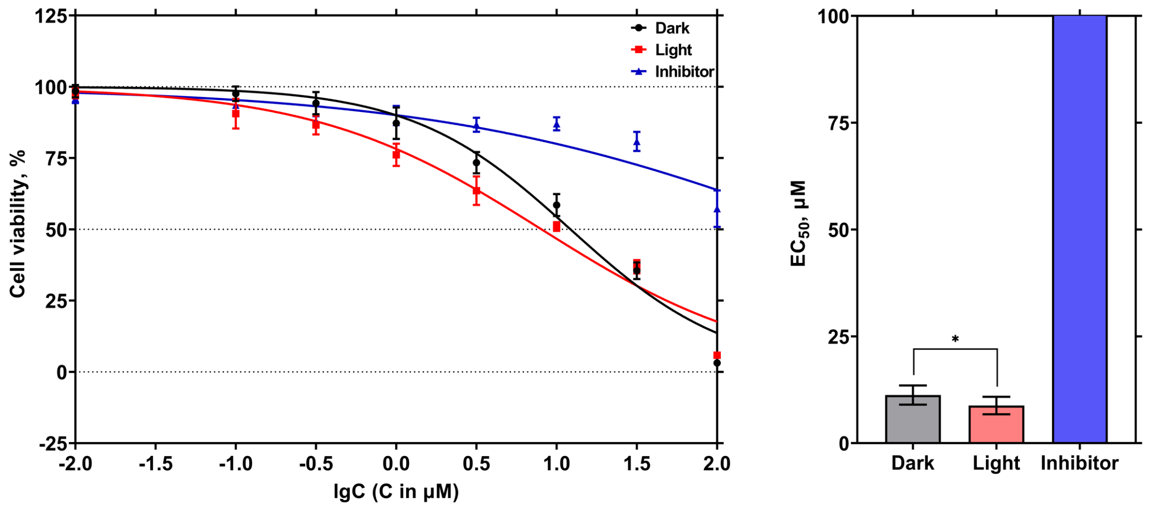

Figure S214. Dose response curves (left) and respective EC_{50} values (right) for **Norharmane** itself and [**10**]Cl_2 in the dark and upon 630 nm red-light irradiation in A375 cell line.

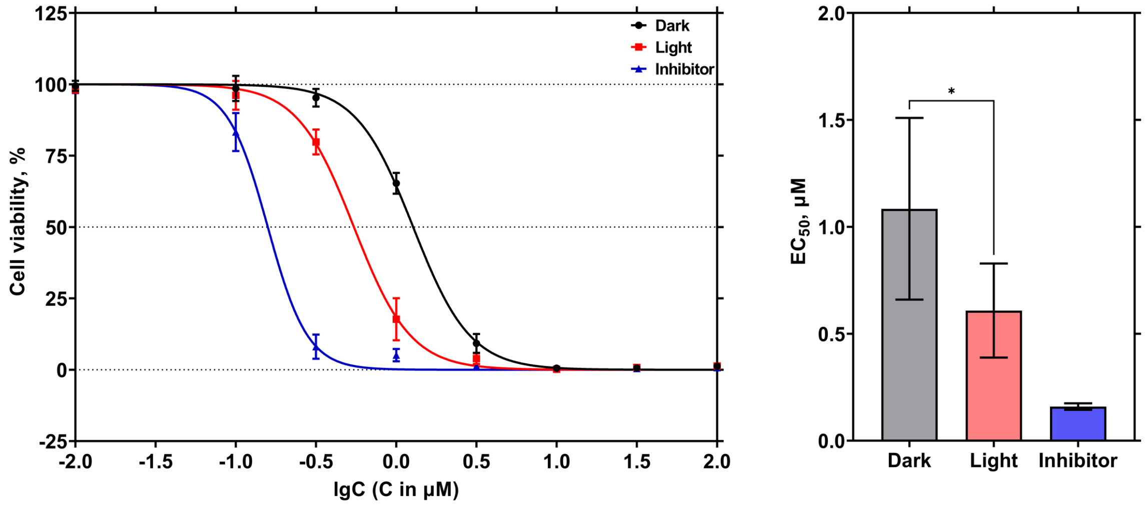

Figure S215. Dose response curves (left) and respective EC_{50} values (right) for **Neratinib** itself and [**11**]Cl_2 in the dark and upon 630 nm red-light irradiation in A375 cell line.

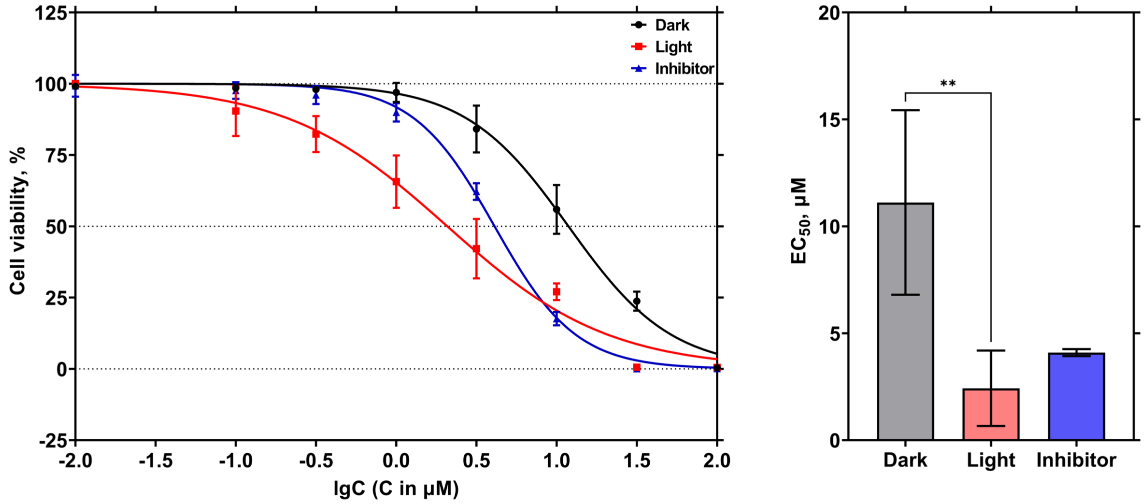

Figure S216. Dose response curves (left) and respective EC_{50} values (right) for **Bosutinib** itself and [**12**]Cl_2 in the dark and upon 630 nm red-light irradiation in A375 cell line.

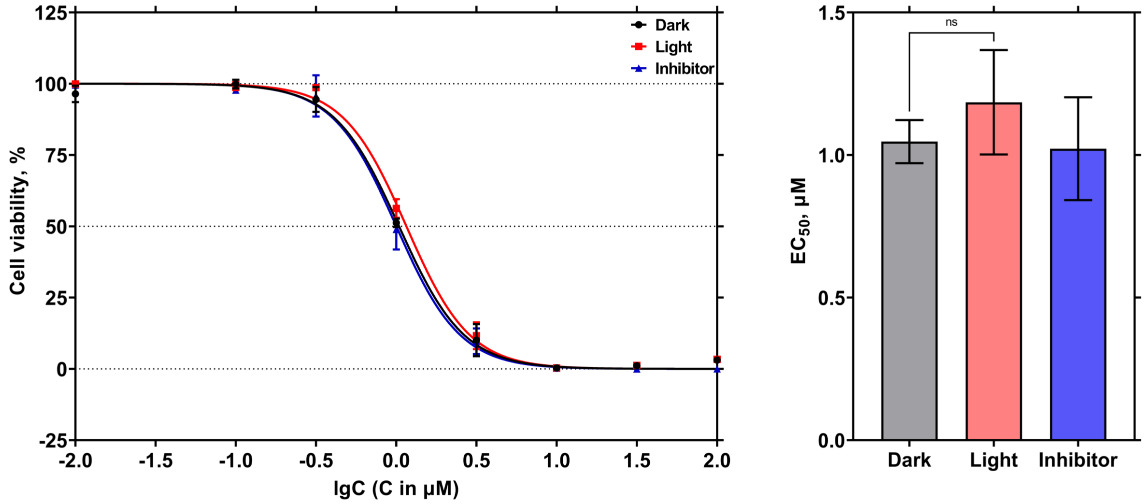

Figure S217. Dose response curves (left) and respective EC_{50} values (right) for **Ponatinib** itself and [**13**]Cl_2 in the dark and upon 630 nm red-light irradiation in A375 cell line.

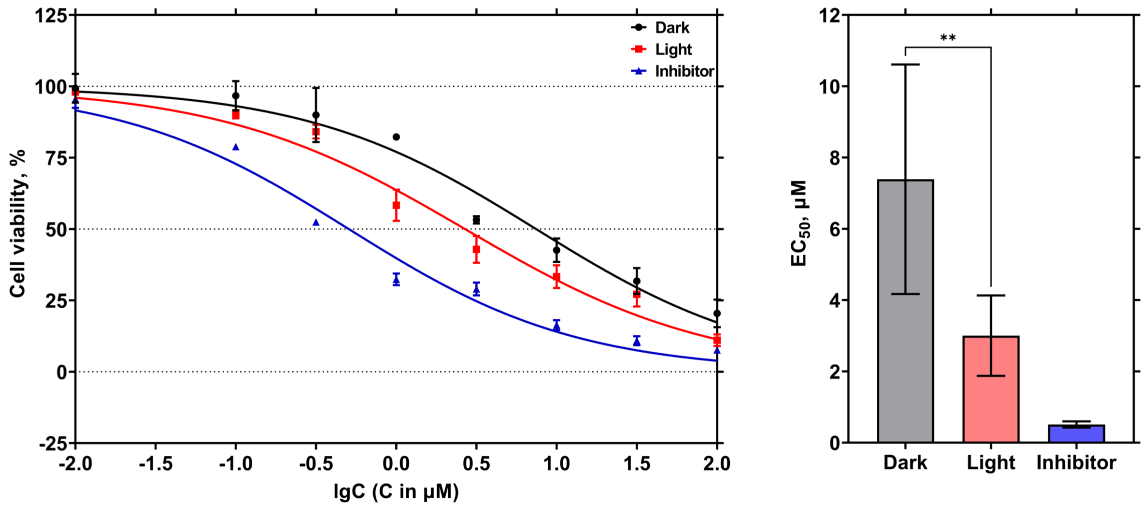

Figure S218. Dose response curves (left) and respective EC_{50} values (right) for **Albendazole** itself and [**14**]$(PF_6)_2$ in the dark and upon 630 nm red-light irradiation in A375 cell line.

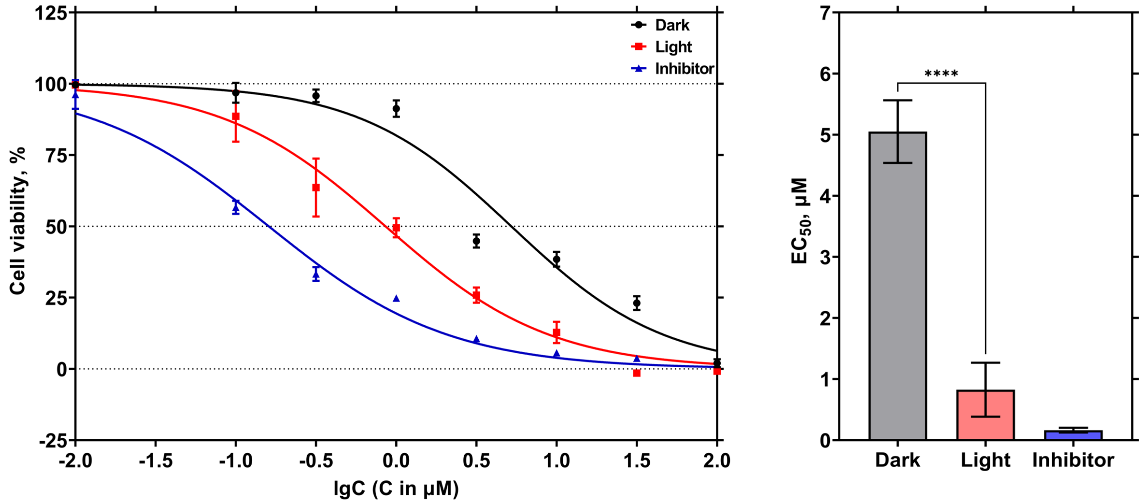

Figure S219. Dose response curves (left) and respective EC_{50} values (right) for **MTI** itself and [**15**]$(PF_6)_2$ in the dark and upon 630 nm red-light irradiation in A375 cell line.

630 nm red light irradiation in U-87MG cell line

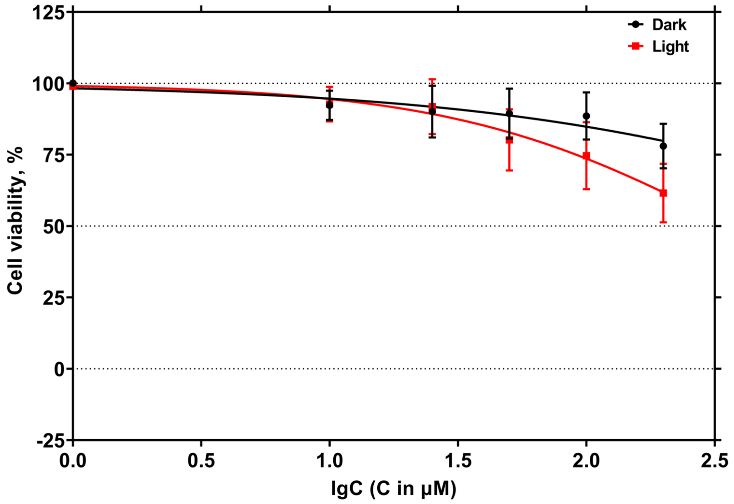

Figure S220. Dose response curves for [**6**]Cl_2 in the dark and upon 630 nm red-light irradiation in U-87MG cell line.

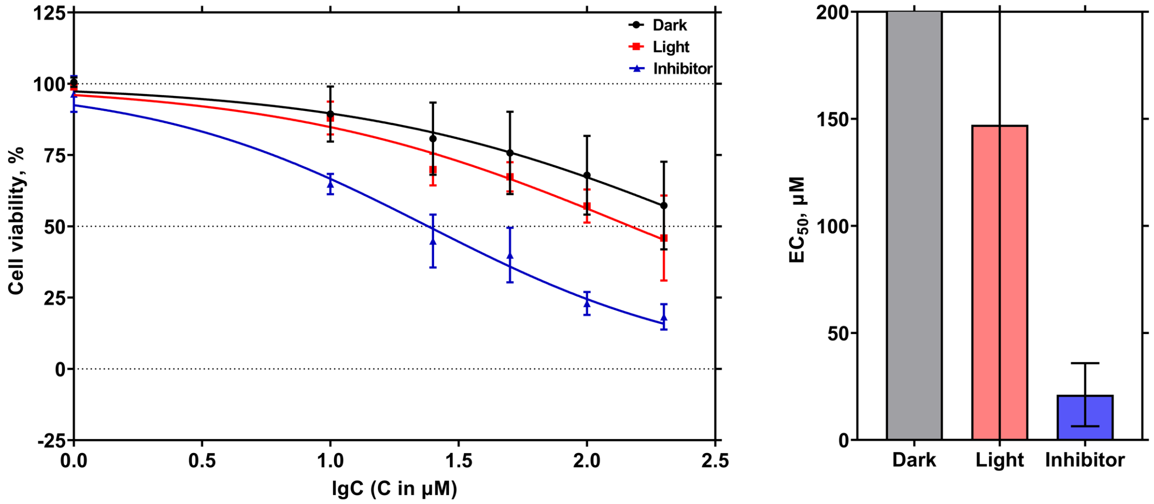

Figure S221. Dose response curves (left) and respective EC_{50} values (right) for **STF-31** itself and [**7**]Cl_2 in the dark and upon 630 nm red-light irradiation in U-87MG cell line.

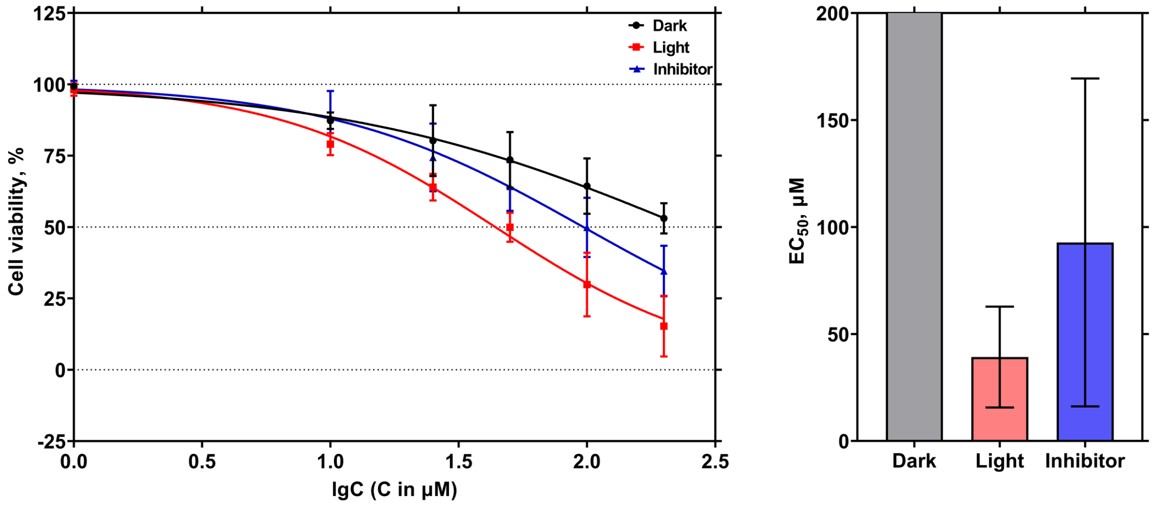

Figure S222. Dose response curves (left) and respective EC$_{50}$ values (right) for **RAD-51-IN-1** itself and [**8**]Cl$_2$ in the dark and upon 630 nm red-light irradiation in U-87MG cell line.

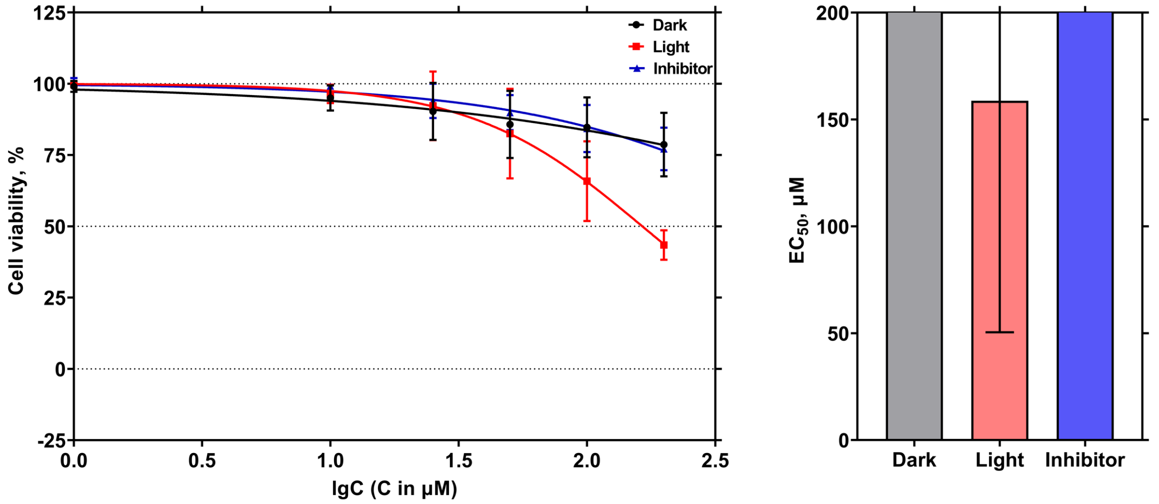

Figure S223. Dose response curves (left) and respective EC$_{50}$ values (right) for **QC-82** itself and [**9**]Cl$_2$ in the dark and upon 630 nm red-light irradiation in U-87MG cell line.

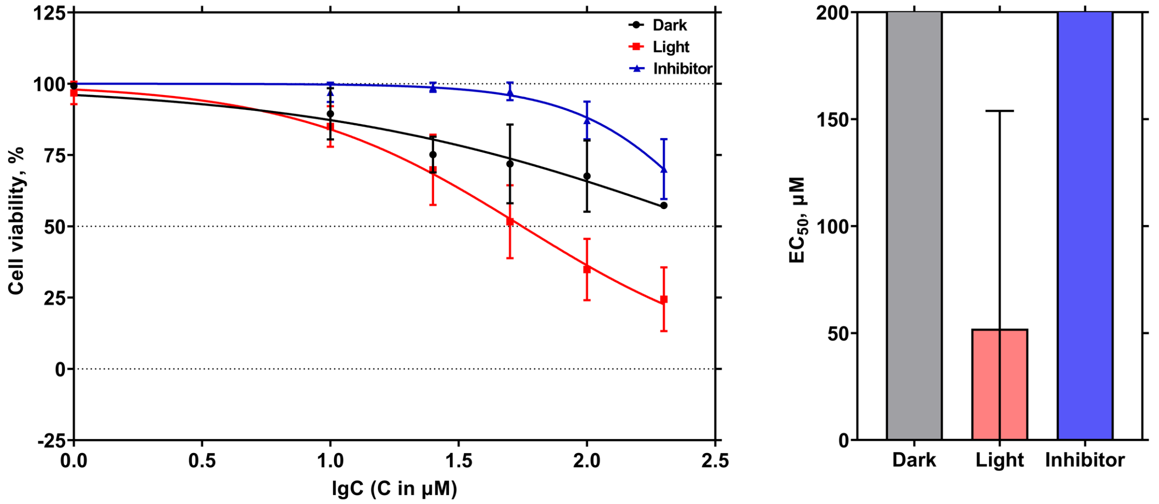

Figure S224. Dose response curves (left) and respective EC$_{50}$ values (right) for **Norharmane** itself and [**10**]Cl$_2$ in the dark and upon 630 nm red-light irradiation in U-87MG cell line.

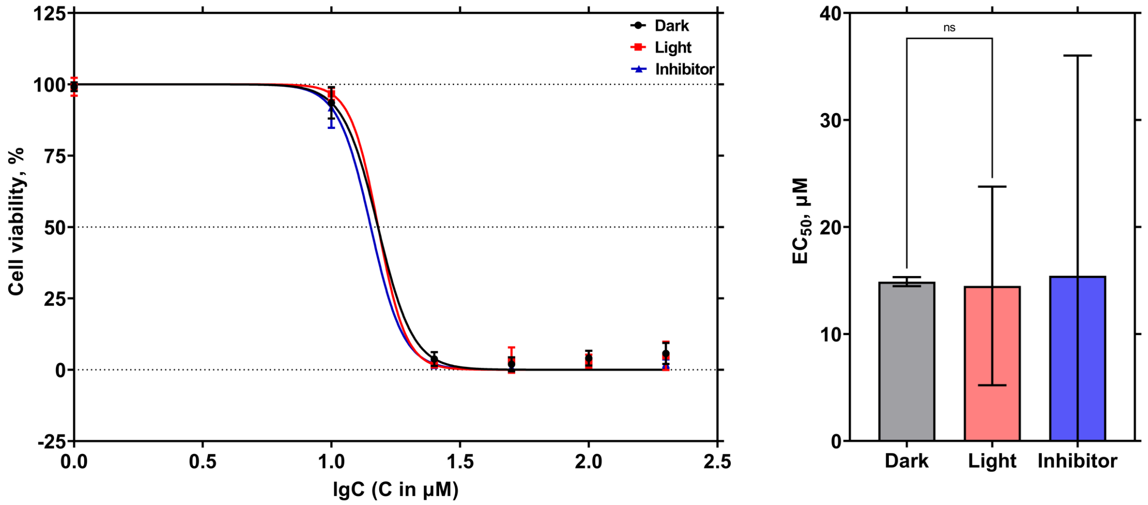

Figure S225. Dose response curves (left) and respective EC_{50} values (right) for **Neratinib** itself and [**11**]Cl_2 in the dark and upon 630 nm red-light irradiation in U-87MG cell line.

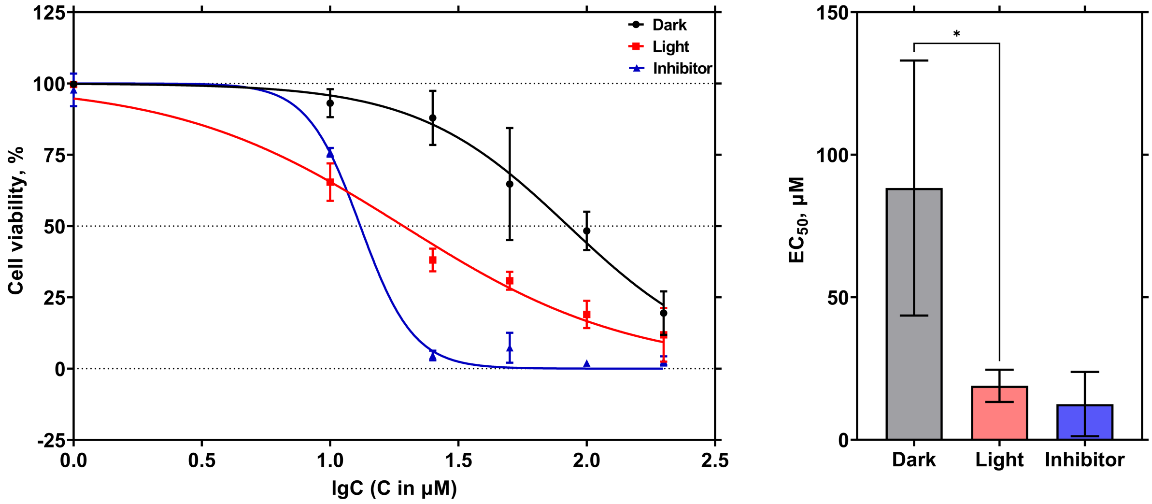

Figure S226. Dose response curves (left) and respective EC_{50} values (right) for **Bosutinib** itself and [**12**]Cl_2 in the dark and upon 630 nm red-light irradiation in U-87MG cell line.

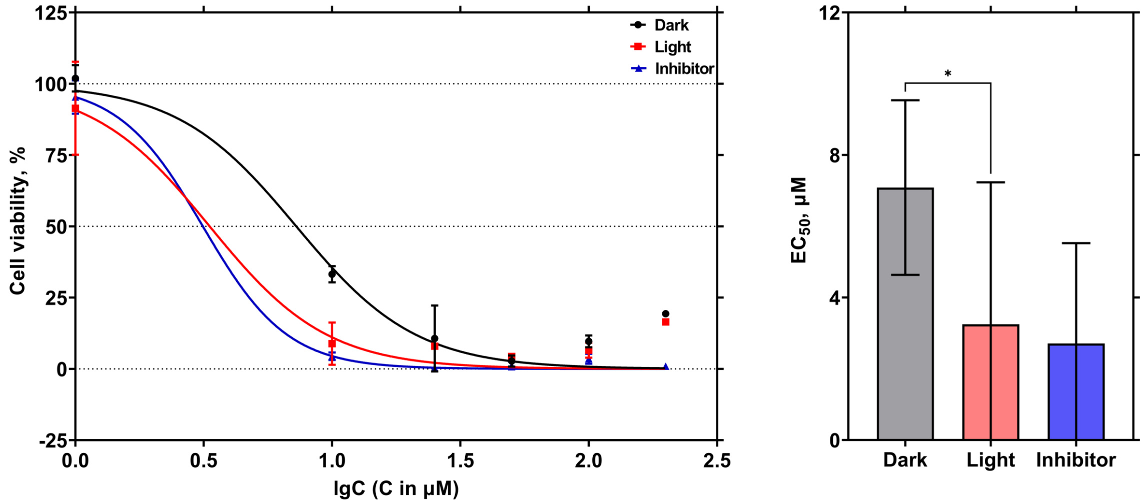

Figure S227. Dose response curves (left) and respective EC_{50} values (right) for **Ponatinib** itself and [**13**]Cl_2 in the dark and upon 630 nm red-light irradiation in U-87MG cell line.

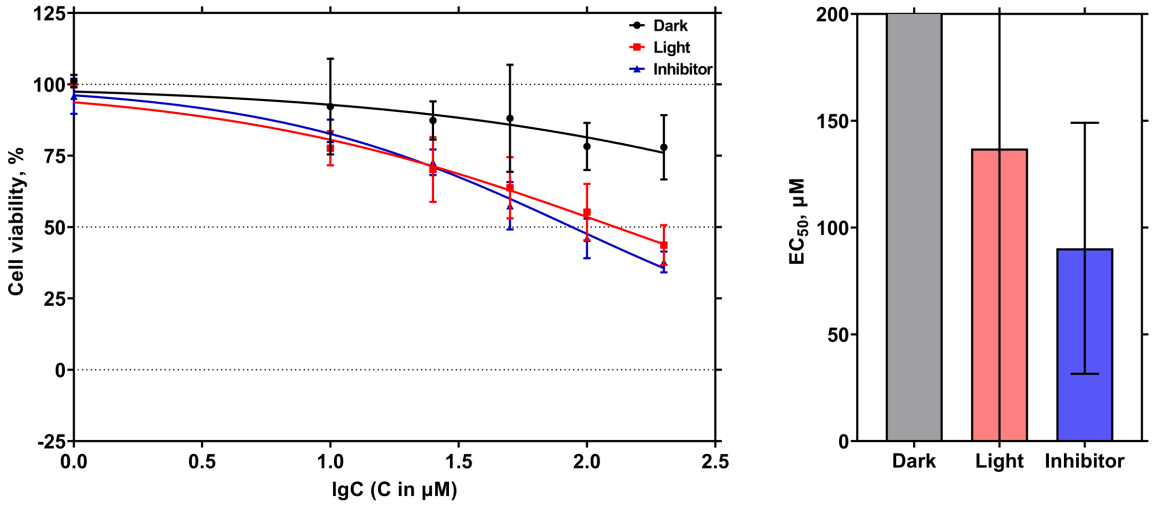

Figure S228. Dose response curves (left) and respective EC_{50} values (right) for **Albendazole** itself and [**14**]$(PF_6)_2$ in the dark and upon 630 nm red-light irradiation in U-87MG cell line.

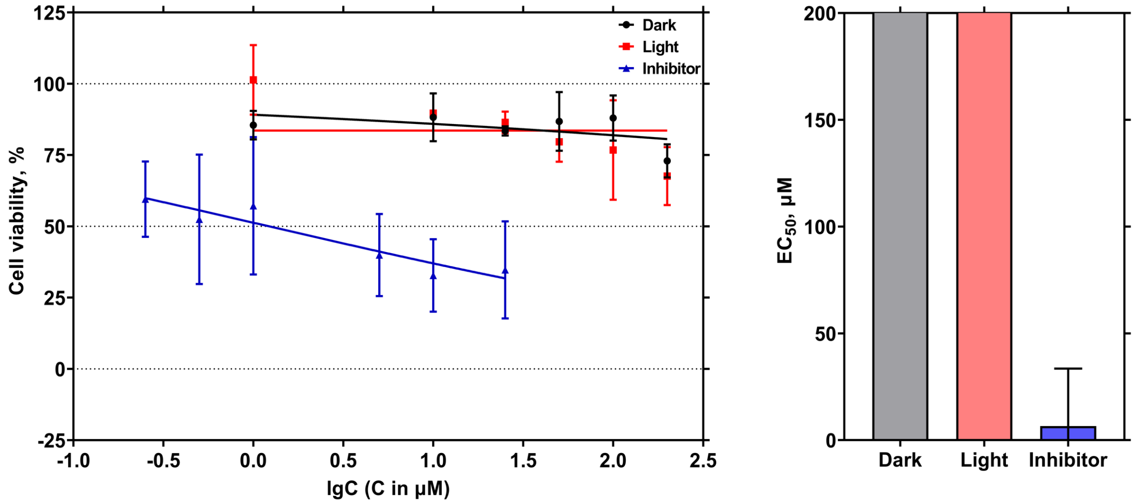

Figure S229. Dose response curves (left) and respective EC_{50} values (right) for **MTI** itself and [**15**]$(PF_6)_2$ in the dark and upon 630 nm red-light irradiation in U-87MG cell line. Due to poor **MTI** solubility the concentration range was changed to 0.25-25 μM.

730 nm far-red light irradiation in A375 cell line

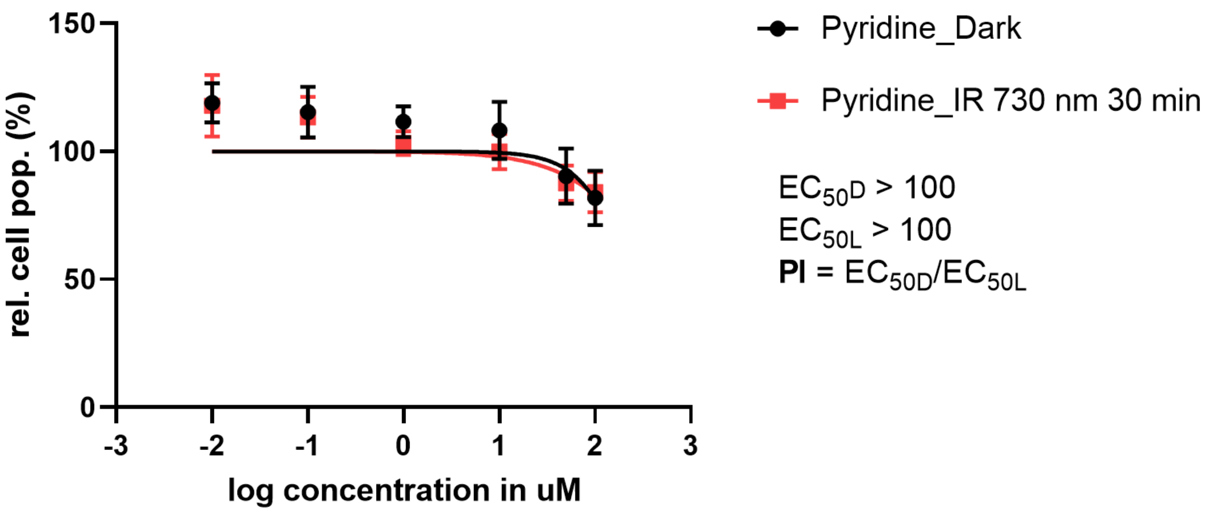

Figure S230. Dose response curves (left) and respective EC_{50} values (right) for [**6**]Cl_2 in the dark and upon 730 nm far-red light irradiation in A375 cell line.

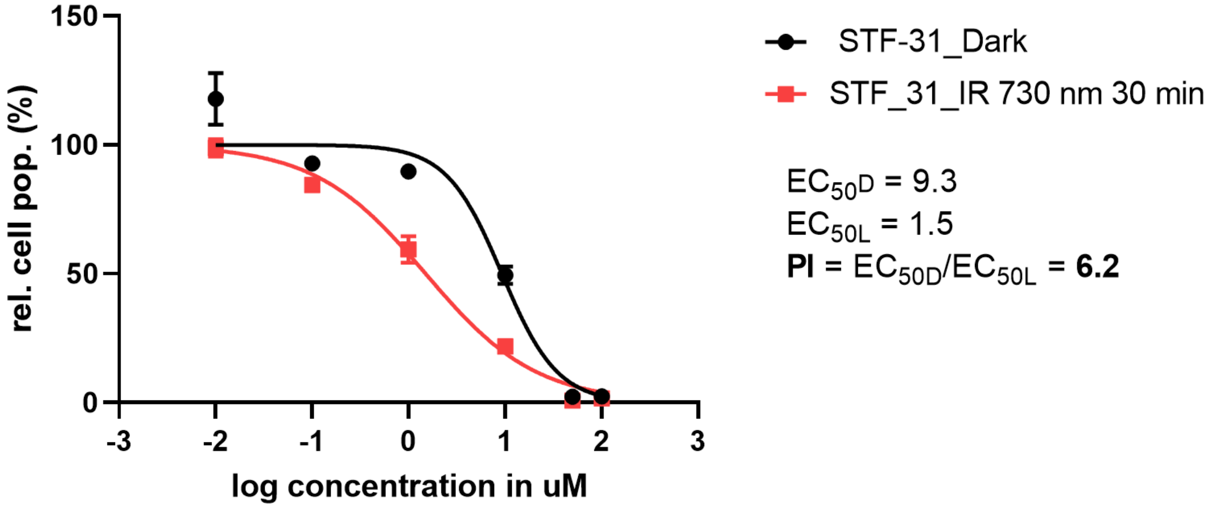

Figure S231. Dose response curves (left) and respective EC_{50} values (right) for [**7**]Cl_2 in the dark and upon 730 nm far-red light irradiation in A375 cell line.

4 Photosubstitution in Opti-MEM complete

In these experiments, 5 mL of 0.1 mM solution of each compound in Opti-MEM complete with 0.4% v/v DMSO was prepared, and an aliquot was analyzed by HPLC at the start. Then 0.2 mL of each solution of each compound was pipetted in the wells of a 96-well plate; this volume and concentration was identical to that used in cytotoxicity experiment in A375 cells at the highest concentration. A blank sample composed of only Opti-MEM complete and 0.4% v/v DMSO was also pipetted in a well and measured by HPLC, see **Figure S232**. The compound-containing samples were then irradiated with 630 nm red light LED array used for the cytotoxicity assay, using exactly the same light dose (61.4 J/cm^2 for red light, 114 J/cm^2 for far-red light) and temperature (37 °C). Then, an aliquot of each well was analyzed by HPLC to see how far the cleavage had gone. Finally, the reference HPLC trace of each free inhibitor was measured as well, so that the peaks on the chromatogram of the "irradiated" samples can be easily interpreted. Data are shown in **Figure S232-S244**.

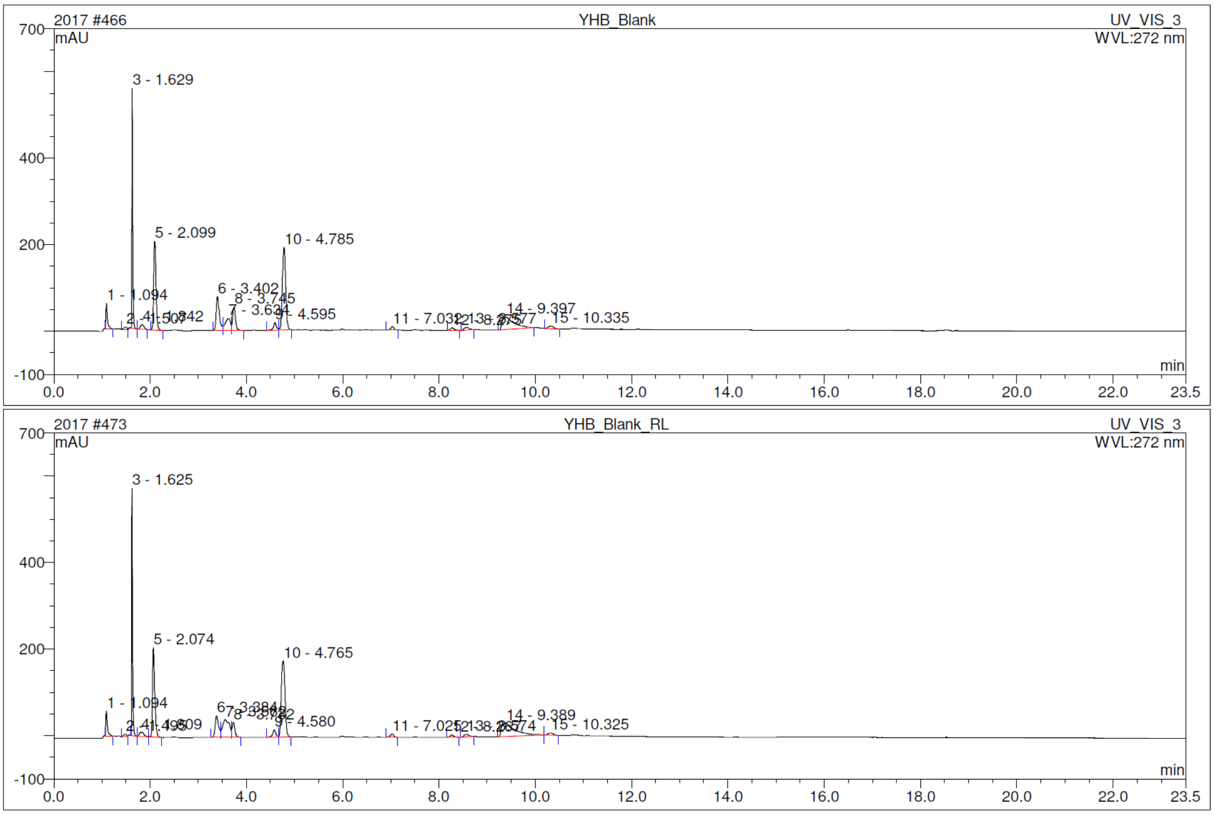

Figure S232. HPLC traces of Opti-MEM complete with 0.4% v/v DMSO (0.2 mL) at the beginning (top) and at the end (bottom) of 630 nm red-light irradiation (34.1 mW/cm^2, 30 min, 61.4 J/cm^2) at 37 °C. No significant changes were observed. These samples can be seen as vehicle control samples for comparison with **Figures S233-S244**.

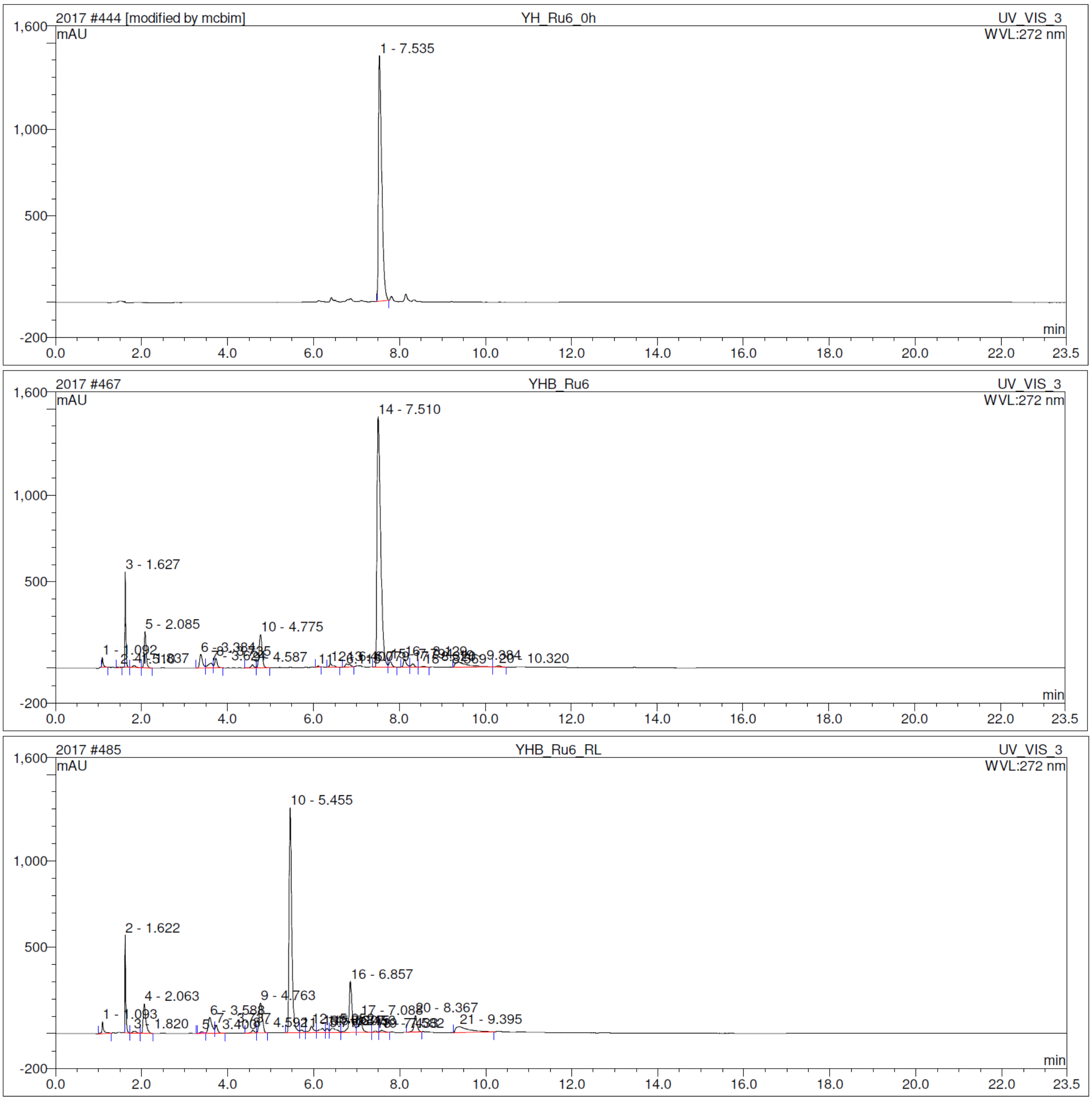

Figure S233. HPLC traces of [**6**]Cl_2 (0.1 mM) at the beginning (middle) and at the end (bottom) of 630 nm red-light irradiation (34.1 mW/cm^2, 30 min, 61.4 J/cm^2) in Opti-MEM complete with 0.4% v/v DMSO (0.2 mL) at 37 °C. Top shows the HPLC trace of free [**6**]Cl_2 (t_R = 7.5 min) for comparison. Complete photosubstitution was observed; the main cleavage product was found at t_R = 5.5 min (Ru residue).

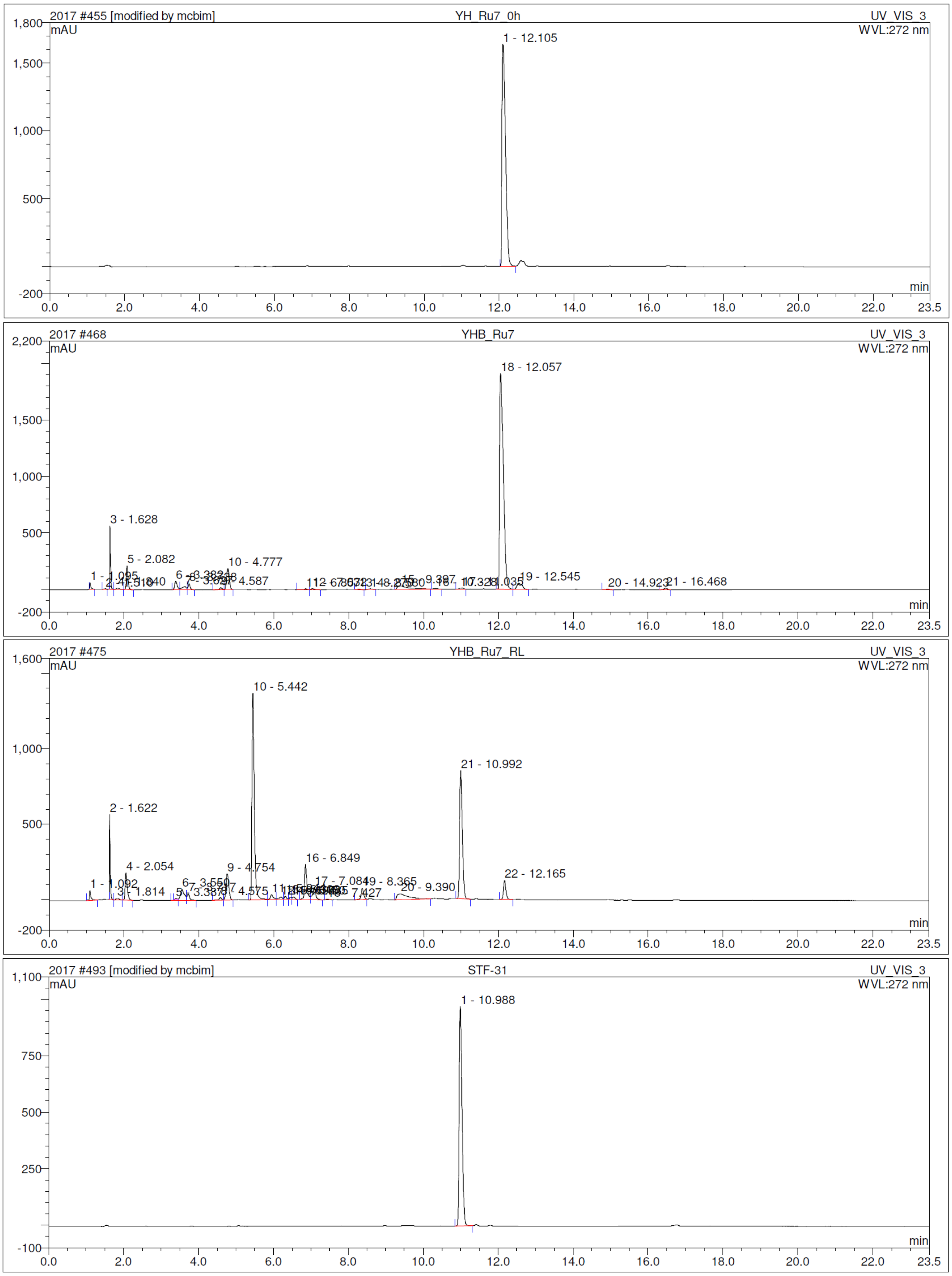

Figure S234. HPLC traces of [**7**]Cl_2 (0.1 mM) at the beginning (middle top) and at the end (middle bottom) of 630 nm red-light irradiation (34.1 mW/cm^2, 30 min, 61.4 J/cm^2) in Opti-MEM complete with 0.4% v/v DMSO (0.2 mL) at 37 °C. HPLC traces of pure [**7**]Cl_2 (top, t_R = 12.1 min) and free **STF-31** (bottom, t_R = 11.0 min) are added for comparison. In such conditions complete photosubstitution was observed; the main cleavage products were found at t_R = 5.5 min (Ru residue) and t_R = 11.0 min (free **STF-31**).

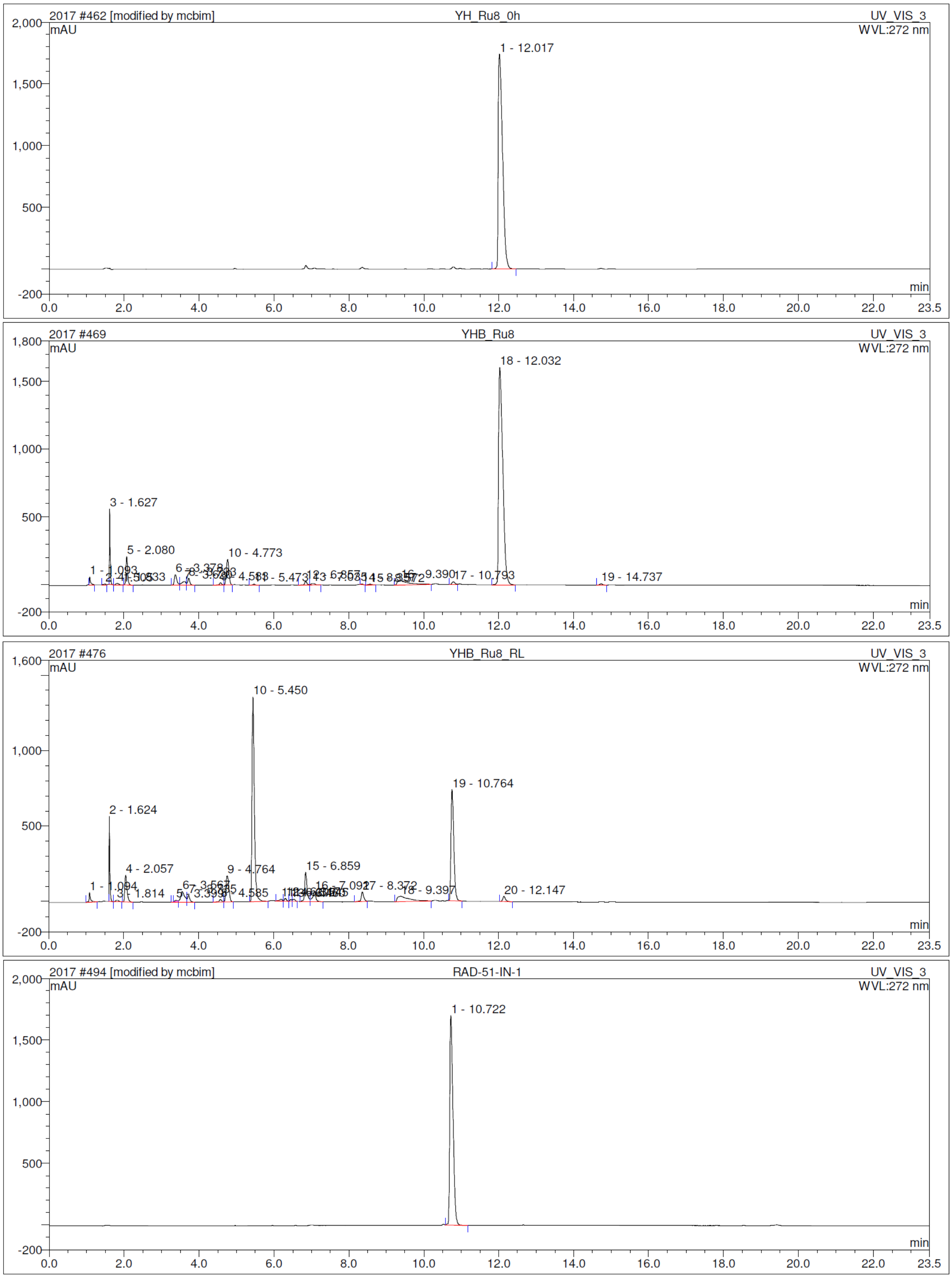

Figure S235. HPLC traces of [**8**]Cl$_2$ (0.1 mM) at the beginning (middle top) and at the end (middle bottom) of 630 nm red-light irradiation (34.1 mW/cm^2, 30 min, 61.4 J/cm^2) in Opti-MEM complete with 0.4% v/v DMSO (0.2 mL) at 37 °C. HPLC traces of pure [**8**]Cl$_2$ (top, t_R = 12.0 min) and of free **RAD-51-IN-1** (bottom, t_R = 10.7 min) are added for comparison, complete photosubstitution was observed; the main cleavage products were found at t_R = 5.5 min (Ru residue) and t_R = 10.7 min (free **RAD-51-IN-1**).

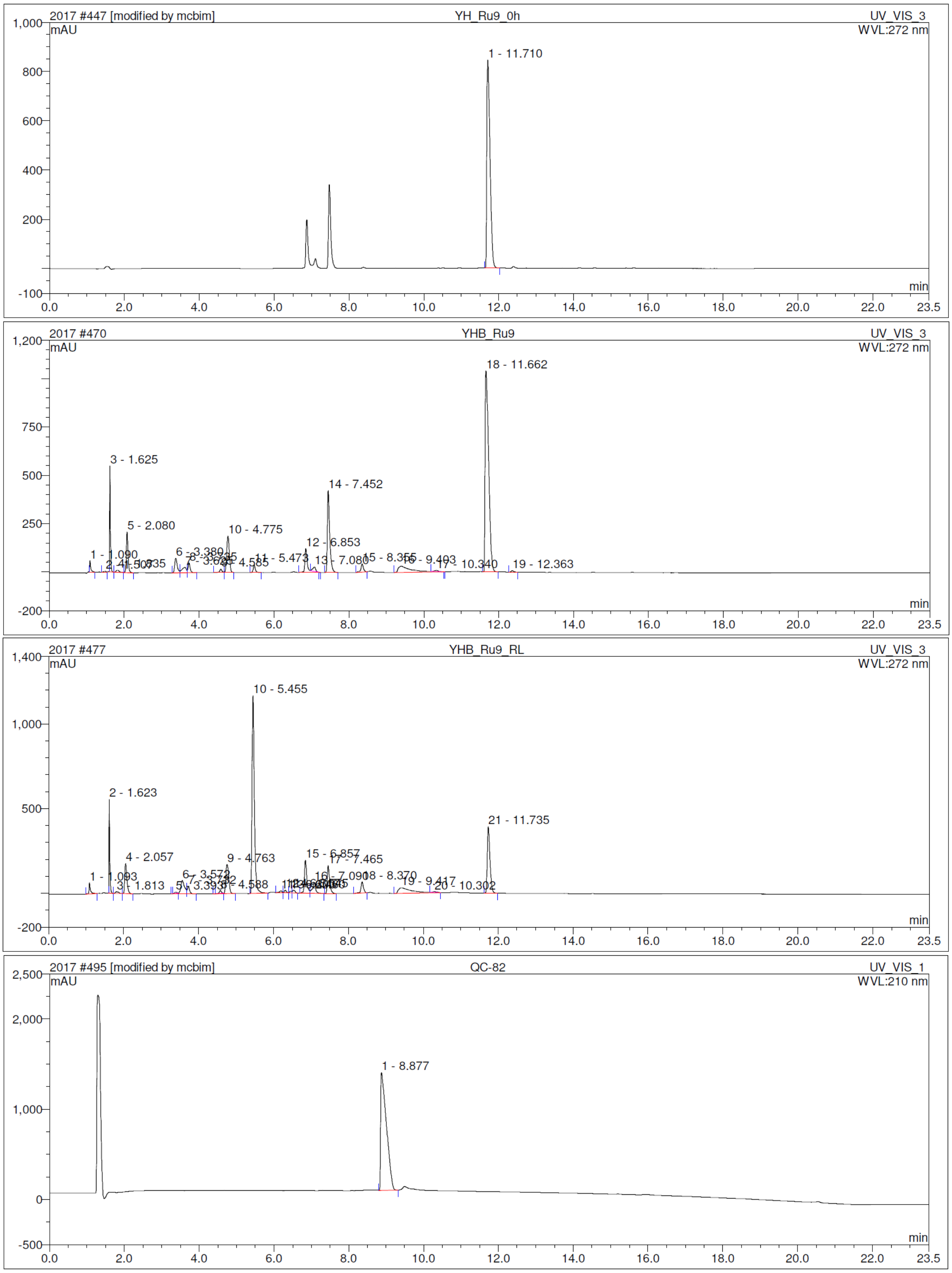

Figure S236. HPLC traces of [**9**]Cl_2 (0.1 mM) at the beginning (middle top) and at the end (middle bottom) of 630 nm red-light irradiation (34.1 mW/cm^2, 30 min, 61.4 J/cm^2) in Opti-MEM complete with 0.4% v/v DMSO (0.2 mL) at 37 °C. HPLC traces of pure [**9**]Cl_2 (top, t_R = 11.7 min) and **QC-82** (bottom, t_R = 8.9 min) are added for comparison, partial photosubstitution was observed, the main cleavage products were found at t_R = 5.5 min (Ru residue) and t_R = 8.9 min (**QC-82**, visible only in 210 nm UV channel).

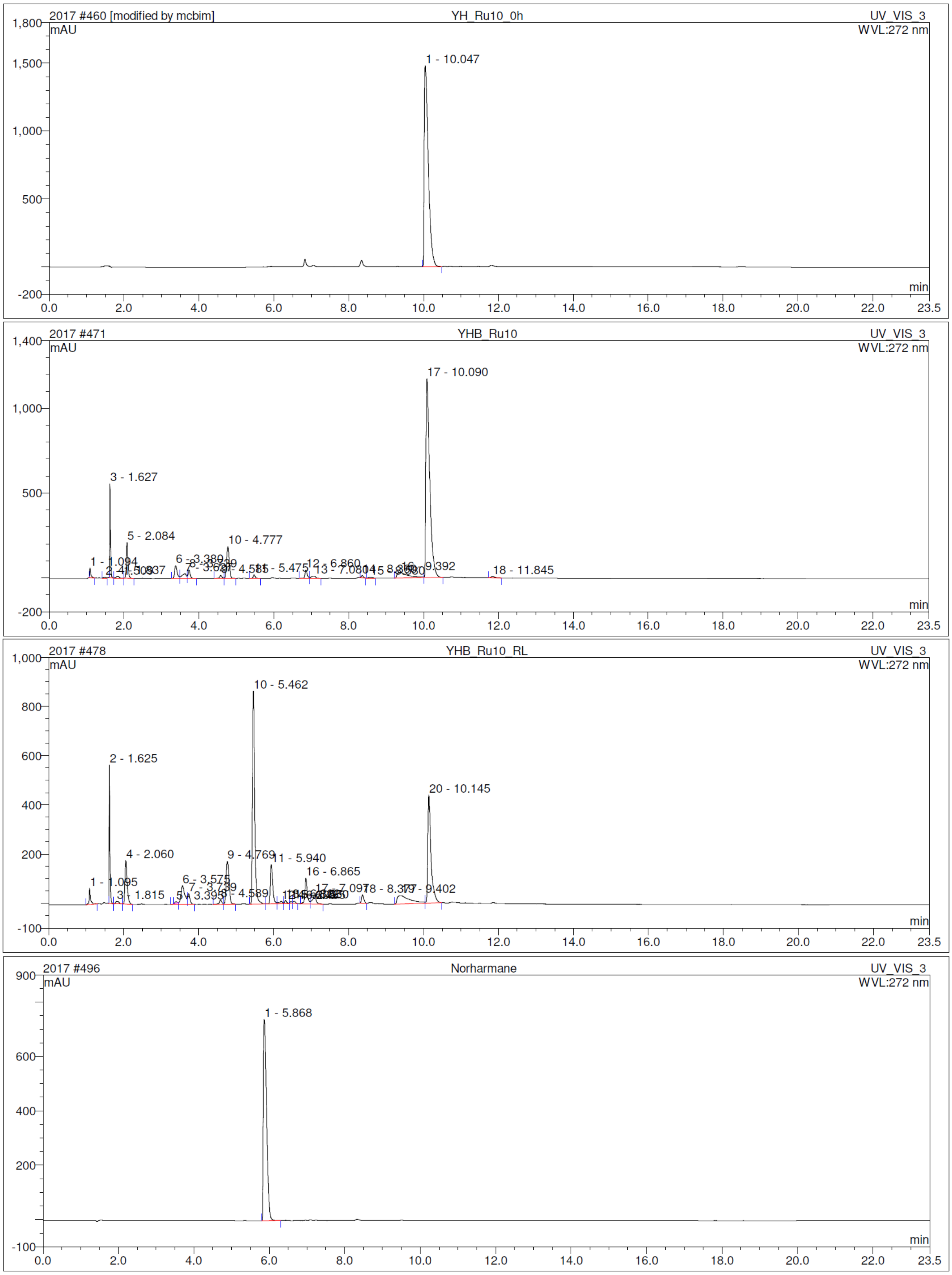

Figure S237. HPLC traces of [**10**]Cl_2 (0.1 mM) at the beginning (middle top) and at the end (middle bottom) of 630 nm red-light irradiation (34.1 mW/cm^2, 30 min, 61.4 J/cm^2) in Opti-MEM complete with 0.4% v/v DMSO (0.2 mL) at 37 °C. HPLC traces of pure [**10**]Cl_2 (top, t_R = 10.1 min) and free **Norharmane** (bottom, t_R = 5.9 min) are added for comparison, partial photosubstitution was observed, main cleavage products were found at t_R = 5.5 min (Ru residue) and t_R = 5.9 min (free **Norharmane**).

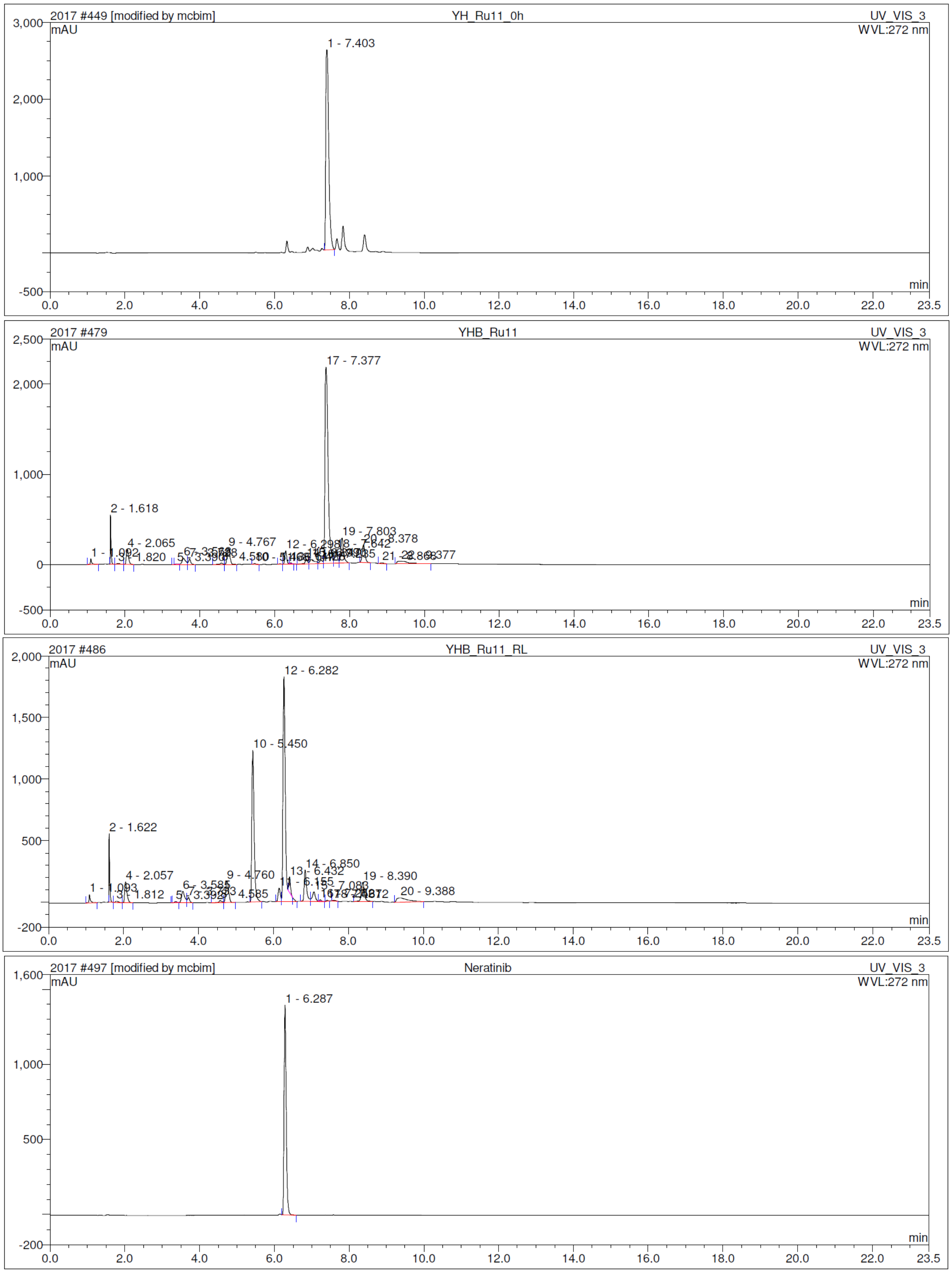

Figure S238. HPLC traces of [**11**]Cl_2 (0.1 mM) at the beginning (middle top) and at the end (middle bottom) of 630 nm red-light irradiation (34.1 mW/cm^2, 30 min, 61.4 J/cm^2) in Opti-MEM complete with 0.4% v/v DMSO (0.2 mL) at 37 °C. HPLC traces of pure [**11**]Cl_2 (top, t_R = 7.4 min) and free **Neratinib** (bottom, t_R = 6.3 min) are added for comparison, complete photosubstitution was observed, main cleavage products were found at t_R = 5.5 min (Ru residue) and t_R = 6.3 min (free **Neratinib**).

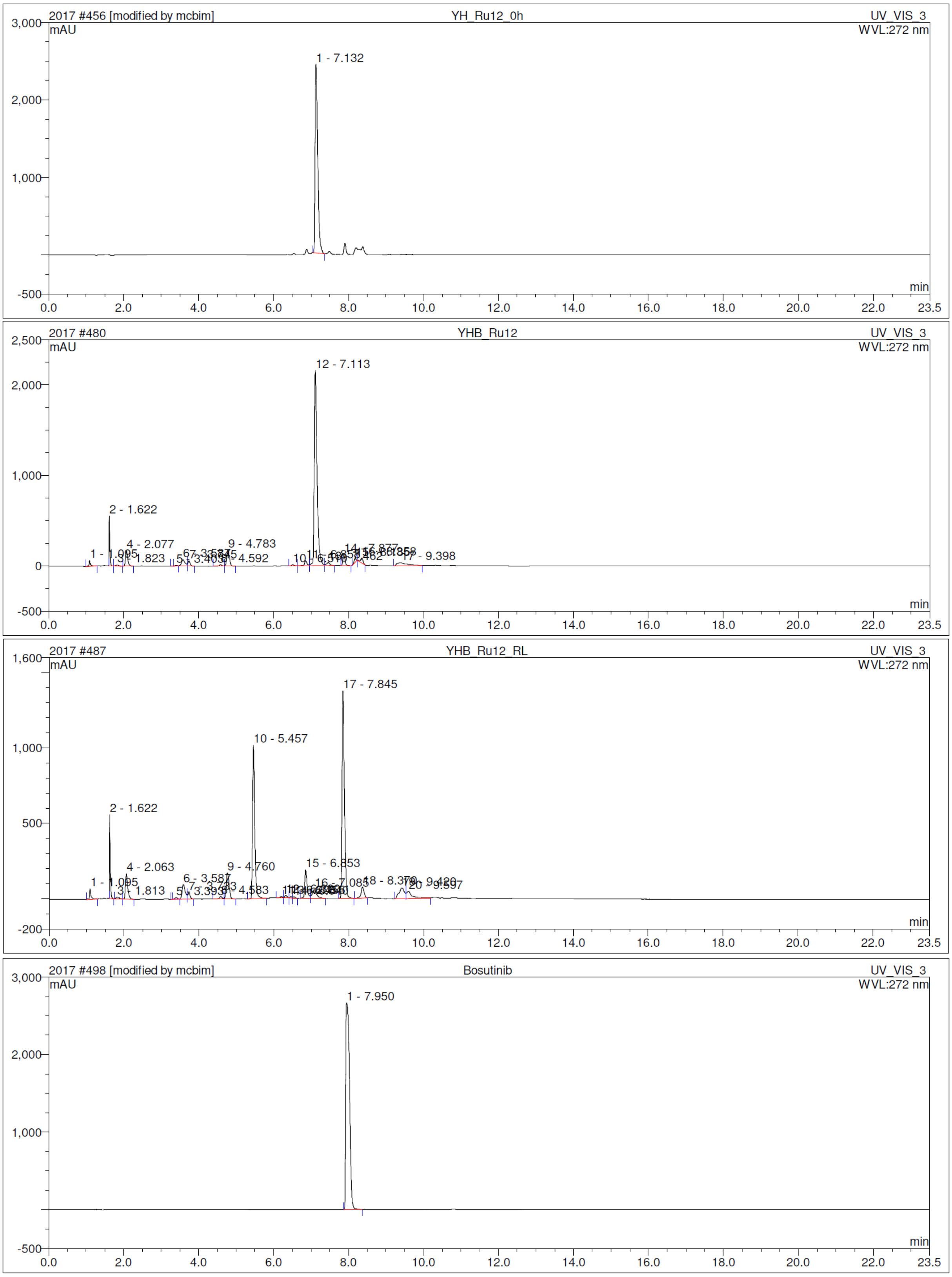

Figure S239. HPLC traces of [**12**]Cl_2 (0.1 mM) at the beginning (middle top) and at the end (middle bottom) of 630 nm red-light irradiation (34.1 mW/cm^2, 30 min, 61.4 J/cm^2) in Opti-MEM complete with 0.4% v/v DMSO (0.2 mL) at 37 °C. HPLC traces of pure [**12**]Cl_2 (top, t_R = 7.1 min) and free **Bosutinib** (bottom, t_R = 7.9 min) are added for comparison, complete photosubstitution was observed, main cleavage products were found at t_R = 5.5 min (Ru residue) and t_R = 7.9 min (free **Bosutinib**).

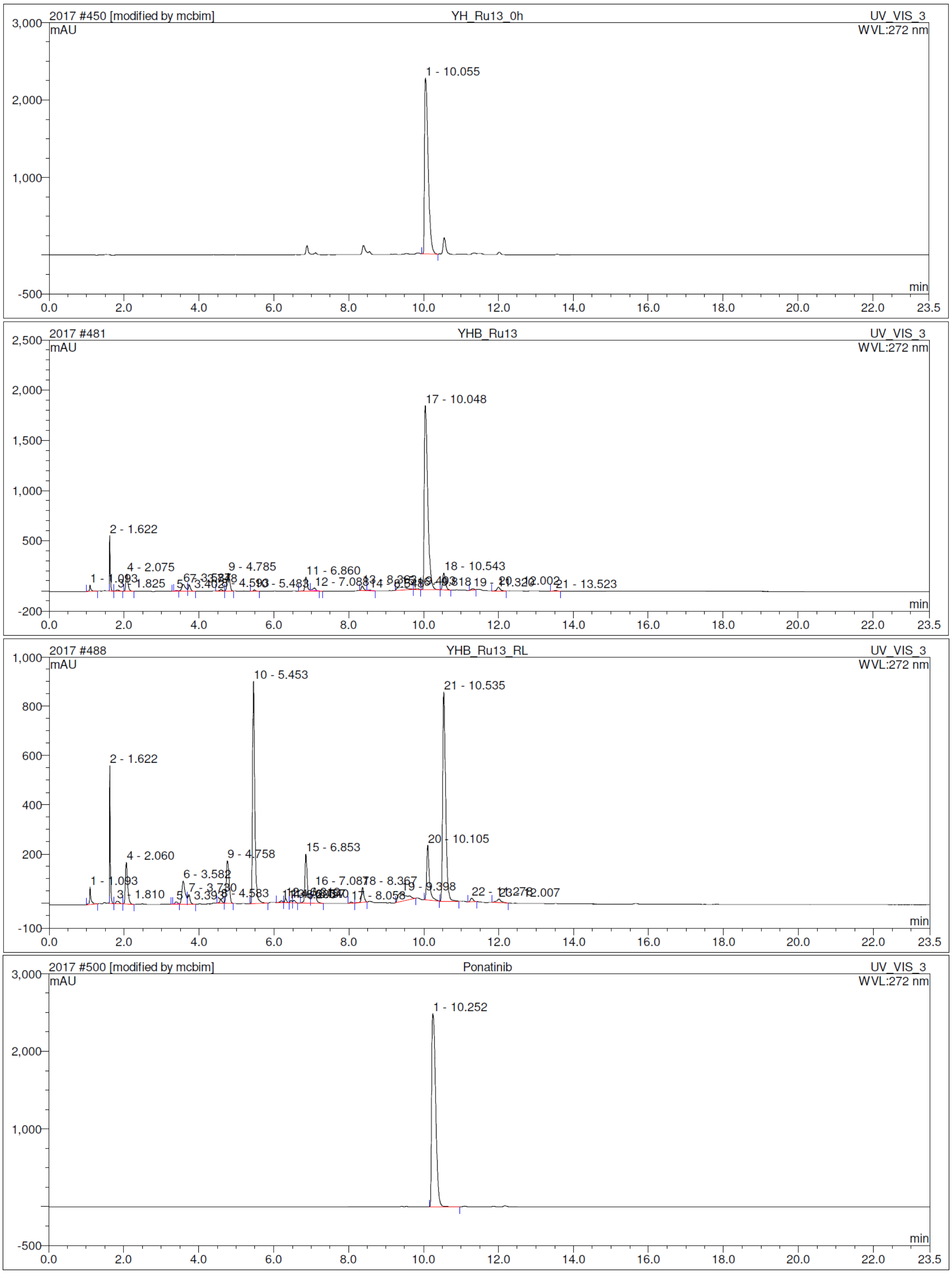

Figure S240. HPLC traces of [**13**]Cl_2 (0.1 mM) at the beginning (middle top) and at the end (middle bottom) of 630 nm red-light irradiation (34.1 mW/cm^2, 30 min, 61.4 J/cm^2) in Opti-MEM complete with 0.4% v/v DMSO (0.2 mL) at 37 °C. HPLC traces of pure [**13**]Cl_2 (top, t_R = 10.1 min) and free **Ponatinib** (bottom, t_R = 10.3 min) are added for comparison, partial photosubstitution was observed, main cleavage products were found at t_R = 5.5 min (Ru residue) and t_R = 10.5 min (free **Ponatinib**).

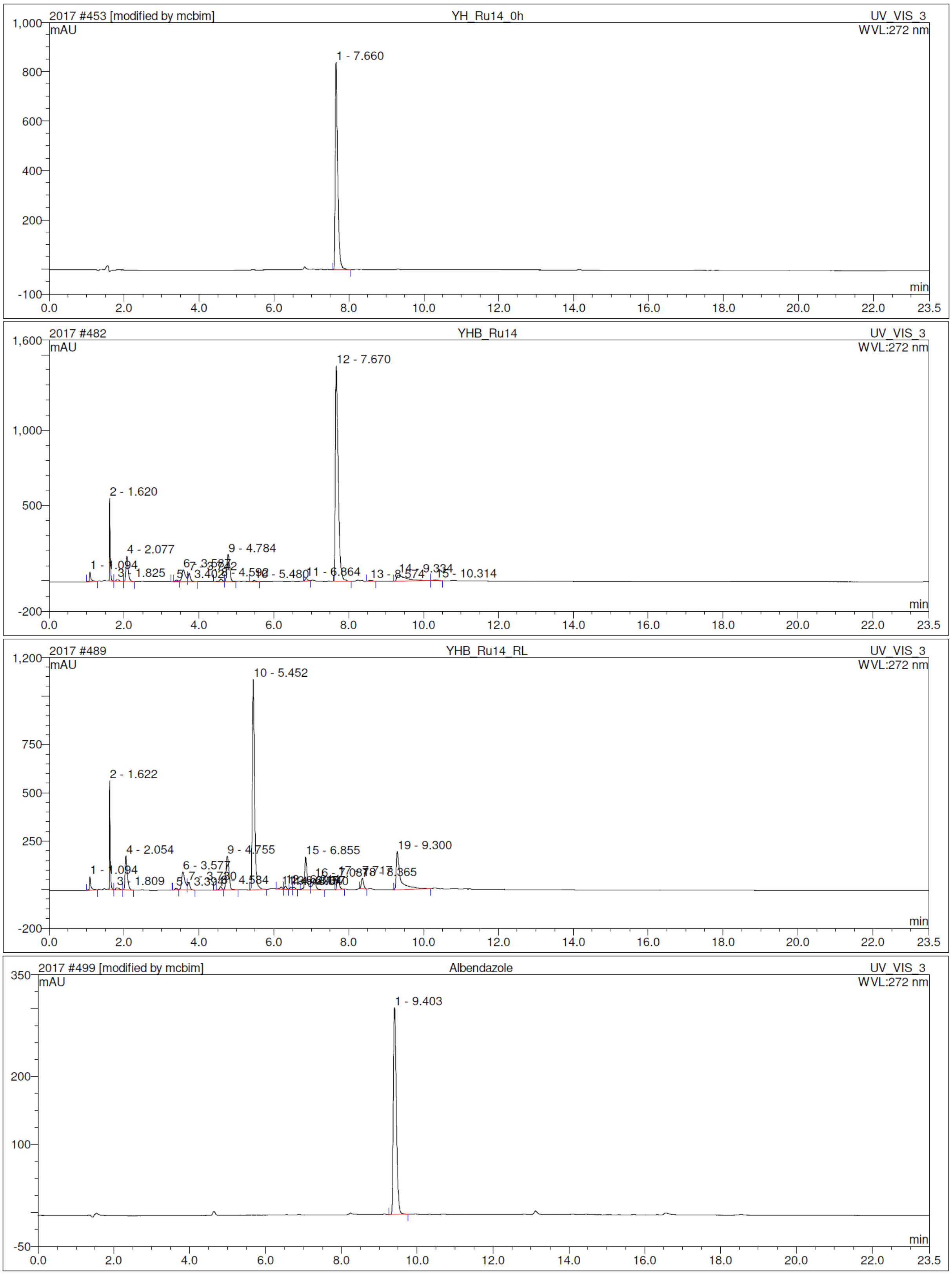

Figure S241. HPLC traces of [**14**](PF_6)$_2$ (0.1 mM) at the beginning (middle top) and at the end (middle bottom) of 630 nm red-light irradiation (34.1 mW/cm^2, 30 min, 61.4 J/cm^2) in Opti-MEM complete with 0.4% v/v DMSO (0.2 mL) at 37 °C. HPLC traces of pure [**14**](PF_6)$_2$ (top, t_R = 7.7 min) and free **Albendazole** (bottom, t_R = 9.4 min) are added for comparison, complete photosubstitution was observed, main cleavage products were found at t_R = 5.5 min (Ru residue) and t_R = 9.3 min (free **Albendazole**).

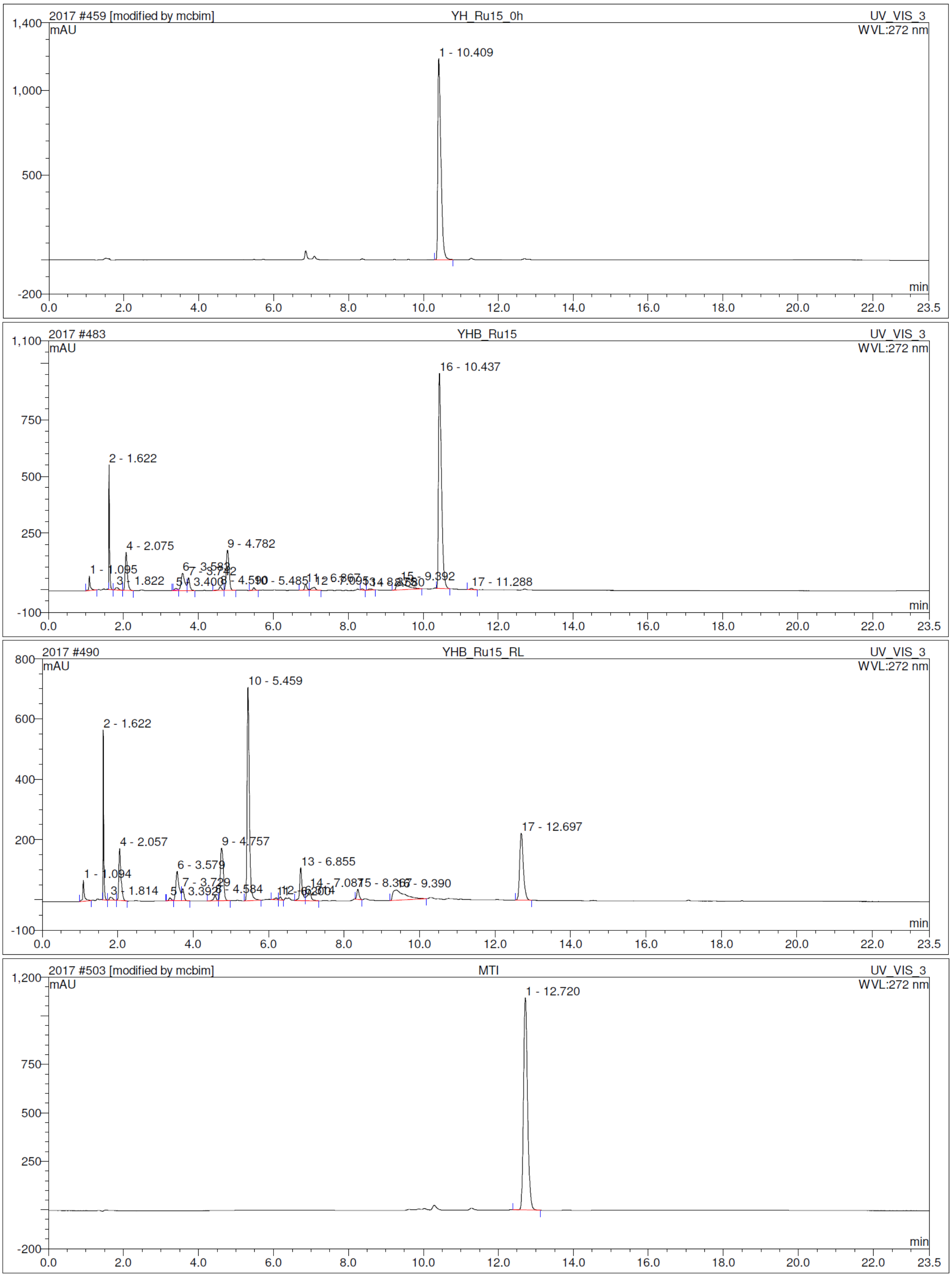

Figure S242. HPLC traces of [**15**]$(PF_6)_2$ (0.1 mM) at the beginning (middle top) and at the end (middle bottom) of 630 nm red-light irradiation (34.1 mW/cm^2, 30 min, 61.4 J/cm^2) in Opti-MEM complete with 0.4% v/v DMSO (0.2 mL) at 37 °C. HPLC traces of pure [**15**]$(PF_6)_2$ (top, t_R = 10.4 min) and free **MTI** (bottom, t_R = 12.7 min) are added for comparison, complete photosubstitution was observed, main cleavage products were found at t_R = 5.5 min (Ru residue) and t_R = 12.7 min (free **MTI**).

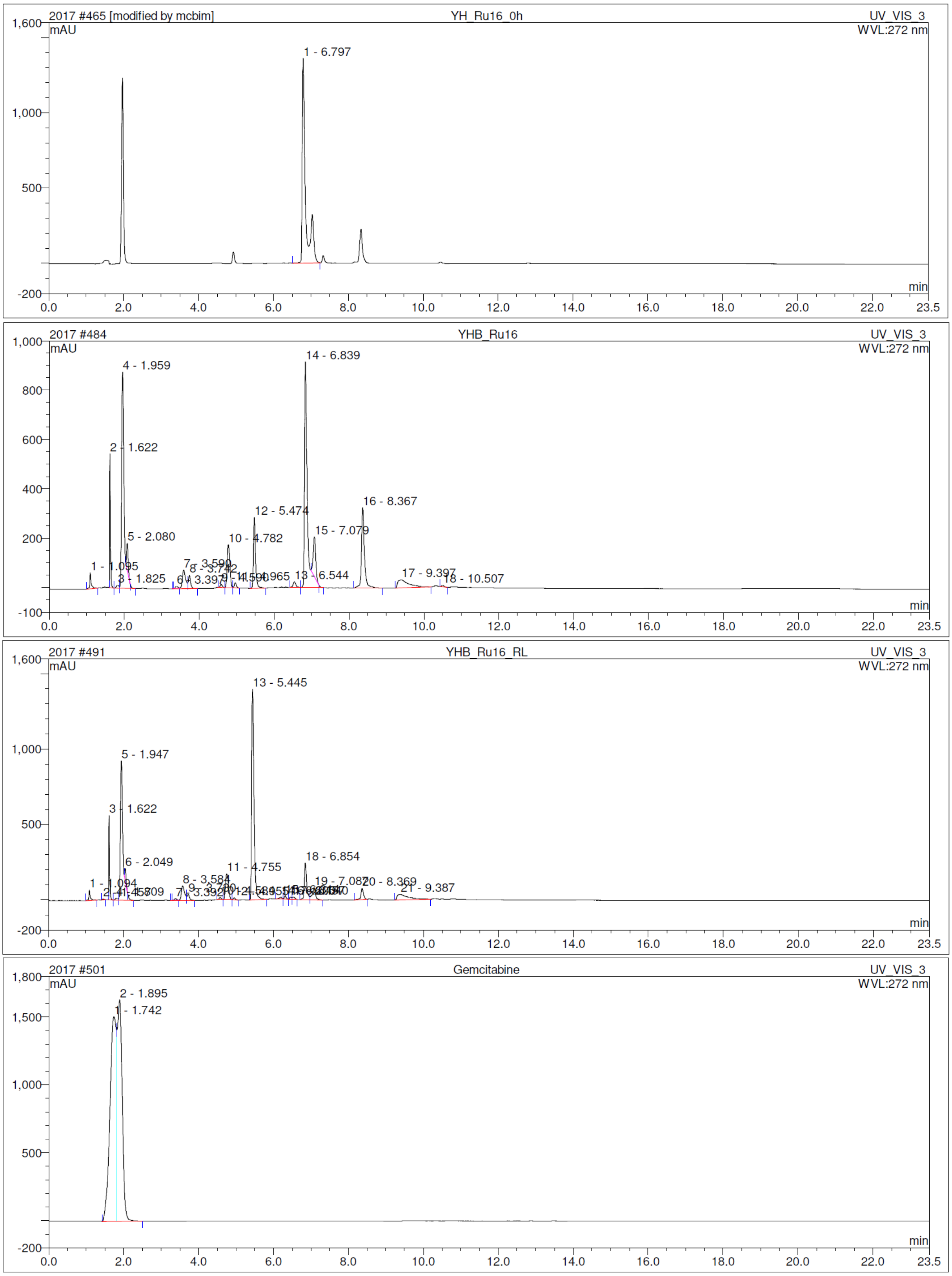

Figure S243. HPLC traces of [**16**]Cl_2 (0.1 mM) at the beginning (middle top) and at the end (middle bottom) of 630 nm red-light irradiation (34.1 mW/cm^2, 30 min, 61.4 J/cm^2) in Opti-MEM complete with 0.4% v/v DMSO (0.2 mL) at 37 °C. HPLC traces of pure [**16**]Cl_2 (top, t_R = 10.4 min) and free **Gemcitabine** (bottom, t_R = 1.9 min) are added for comparison, almost full photosubstitution was observed, main cleavage products were found at t_R = 5.5 min (Ru residue) and t_R = 1.9 min (free **Gemcitabine**).

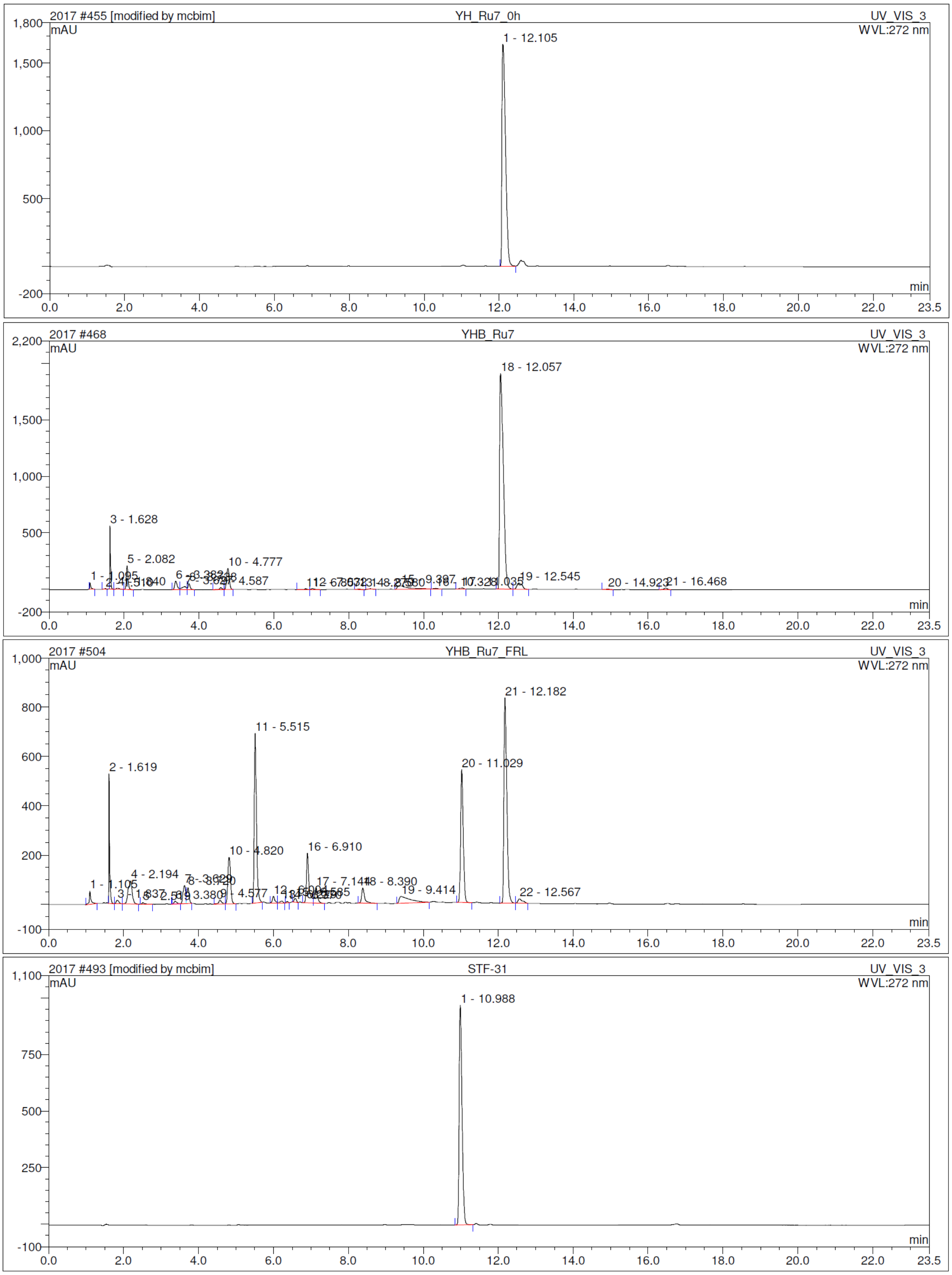

Figure S244. HPLC traces of [**7**]Cl_2 (0.1 mM) at the beginning (middle top) and at the end (middle bottom) of 730 nm far-red light irradiation (63.2 mW/cm^2, 30 min, 114 J/cm^2) in Opti-MEM complete with 0.4% v/v DMSO (0.2 mL) at 37 °C. HPLC traces of pure [**7**]Cl_2 (top, t_R = 12.1 min) and free **STF-31** (bottom, t_R = 11.0 min) are added for comparison, partial photosubstitution was observed, main cleavage products were found at t_R = 5.5 min (Ru residue) and t_R = 11.0 min (free **STF-31**).

5 DFT studies

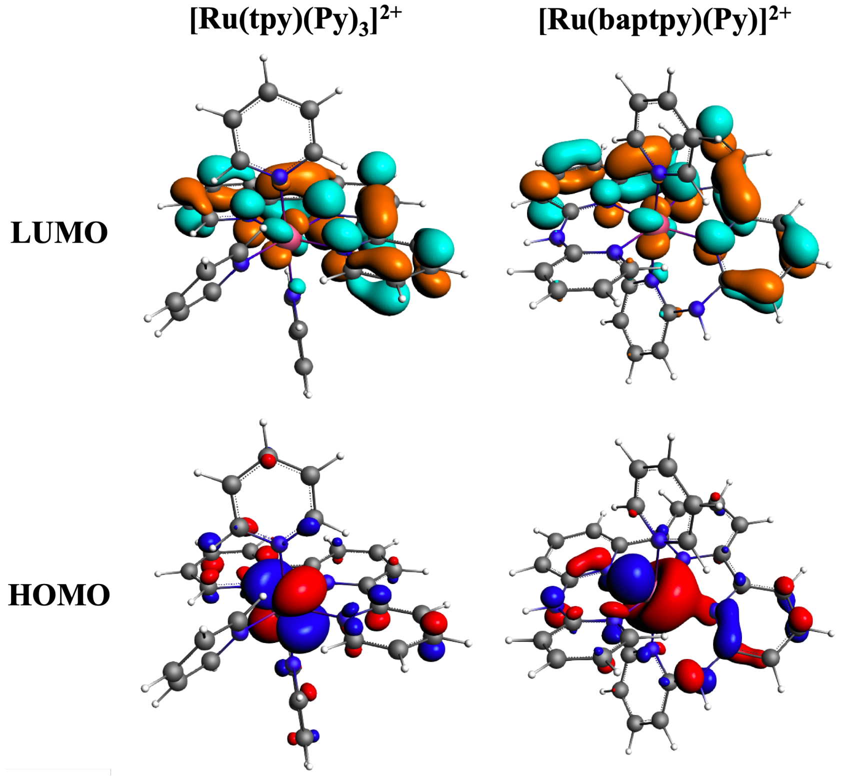

Figure S245. The HOMO and LUMO orbitals of $[Ru(tpy)(Py)_3]^{2+}$ and $[Ru(baptpy)(Py)]^{2+}$. Isopotential was set at 0.035.

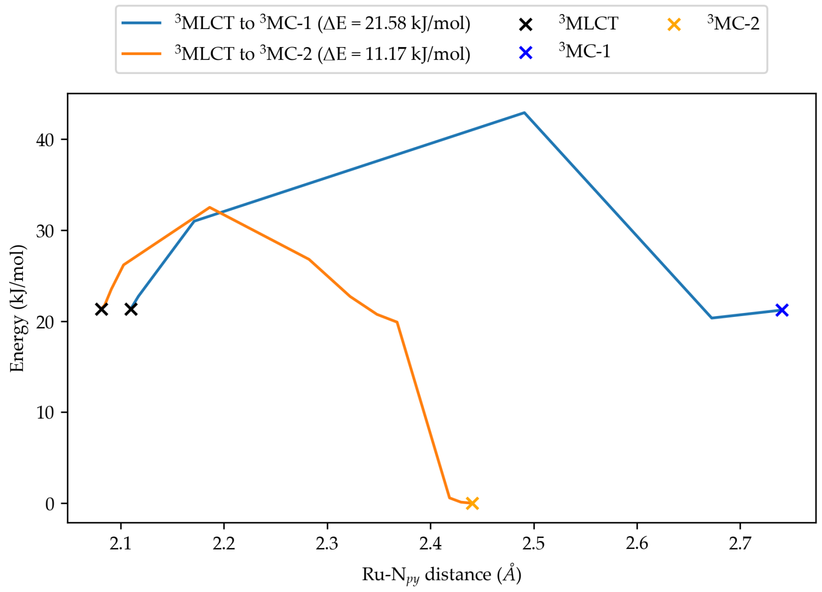

Figure S246. The minimum energy paths between the ^{3}MLCT and ^{3}MC states found for $[Ru(tpy)(Py)_3]^{2+}$. The horizontal axis plots the length of Ru-N bond that is broken upon accessing the dissociative ^{3}MC state.

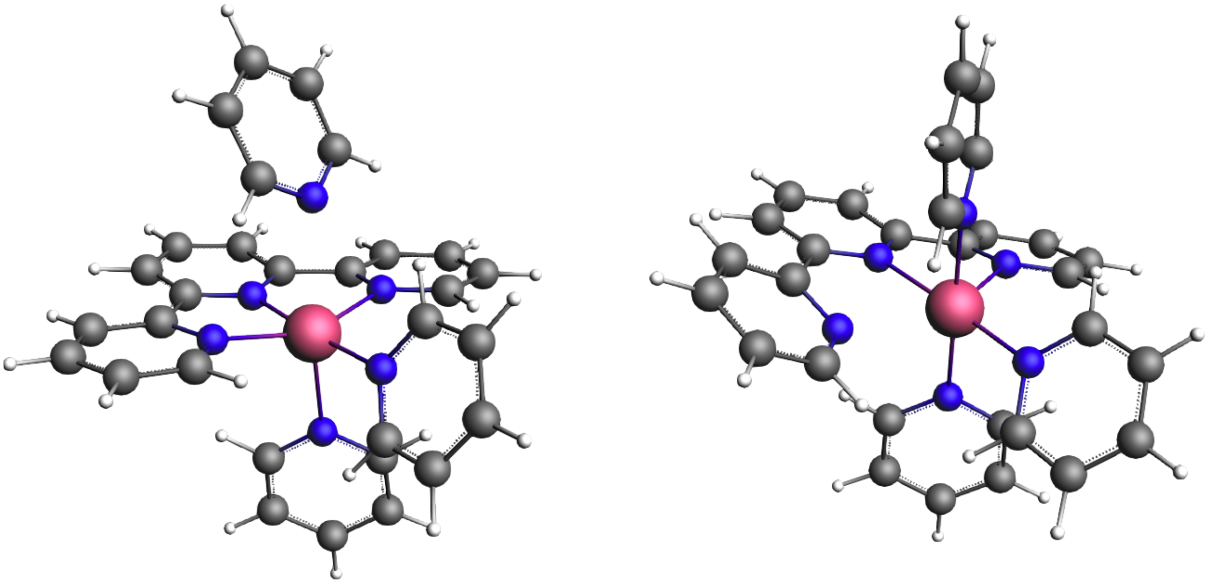

^{3}MC-1 **^{3}MC-2**

Figure S247. Geometries of the ^{3}MC states found for $[Ru(tpy)(Py)_3]^{2+}$.

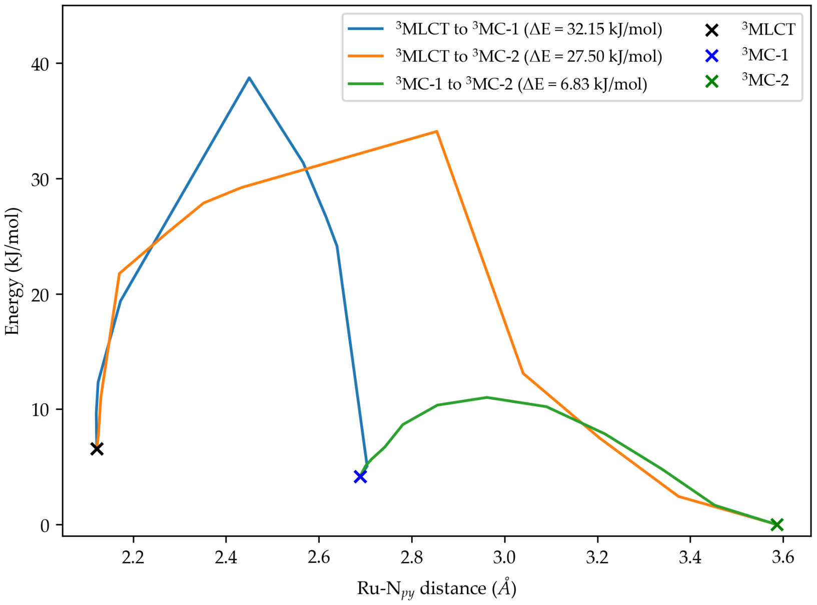

Figure S248. The minimum energy path between the ^{3}MLCT and ^{3}MC states found for $[Ru(baptpy)(Py)]^{2+}$. The horizontal axis plots the length of Ru-N bond that is broken upon accessing the dissociative ^{3}MC state.

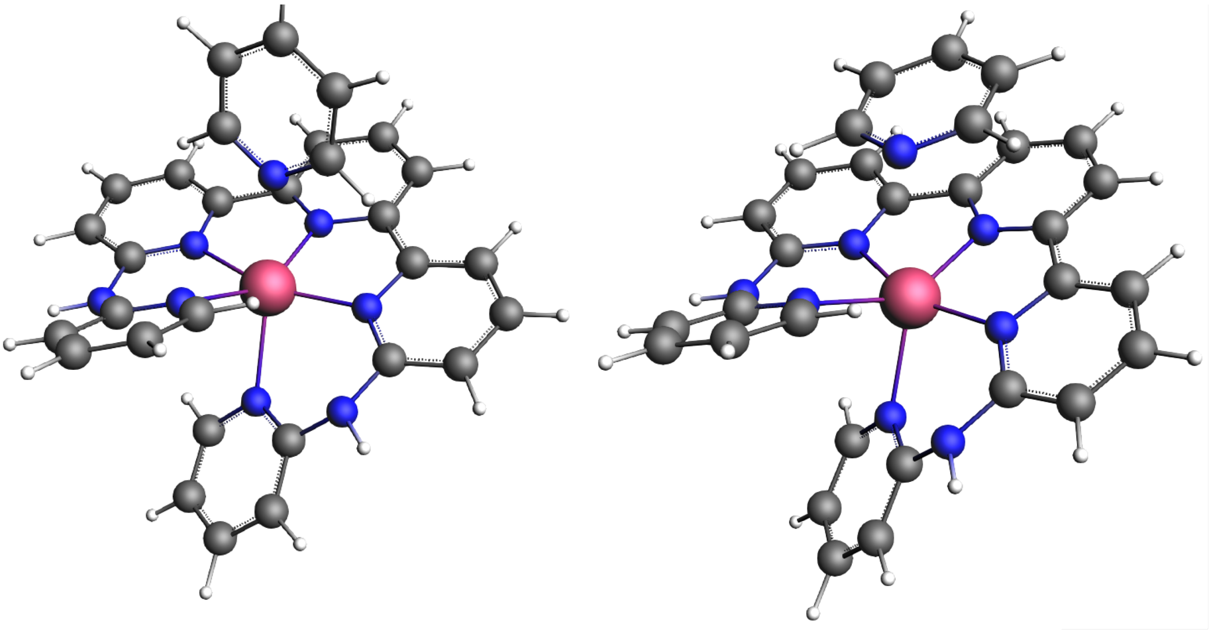

^{3}MC-1 ^{3}MC-2

Figure S249. Geometries of the ^{3}MC states found for $[Ru(baptpy)(Py)]^{2+}$.

Table S19. ^{3}MLCT-^{3}MC internal energy differences of $[Ru(tpy)(Py)_3]^{2+}$.

State	ΔE(State – ^{1}GS), kJ/mol	ΔE(State – ^{3}MLCT), kJ/mol	ΔE(X – ^{3}MLCT), kJ/mol
^{3}MLCT	189.8	0	–
^{3}MC-1	189.7	–0.02	21.6
^{3}MC-2	168.4	–21.3	11.2

^{a} The highest energetic point found along the nudged elastic band pathway going from the ^{3}MLCT state to state X.

6 References

(1) Fulmer, G. R.; Miller, A. J. M.; Sherden, N. H.; Gottlieb, H. E.; Nudelman, A.; Stoltz, B. M.; Bercaw, J. E.; Goldberg, K. I. NMR Chemical Shifts of Trace Impurities: Common Laboratory Solvents, Organics, and Gases in Deuterated Solvents Relevant to the Organometallic Chemist. *Organometallics* **2010**, *29* (9), 2176–2179. https://doi.org/10.1021/om100106e.

(2) Lameijer, L. N.; Ernst, D.; Hopkins, S. L.; Meijer, M. S.; Askes, S. H. C.; Le Dévédec, S. E.; Bonnet, S. A Red-Light-Activated Ruthenium-Caged NAMPT Inhibitor Remains Phototoxic in Hypoxic Cancer Cells. *Angew. Chem. Int. Ed.* **2017**, *56* (38), 11549–11553. https://doi.org/10.1002/anie.201703890.

(3) Rahman, M. N.; Vlahakis, J. Z.; Szarek, W. A.; Nakatsu, K.; Jia, Z. X-Ray Crystal Structure of Human Heme Oxygenase-1 in Complex with 1-(Adamantan-1-Yl)-2-(1 *H* -Imidazol-1-Yl)Ethanone: A Common Binding Mode for Imidazole-Based Heme Oxygenase-1 Inhibitors. *J. Med. Chem.* **2008**, *51* (19), 5943–5952. https://doi.org/10.1021/jm800505m.

(4) Ward, A.; Dong, L.; Harris, J. M.; Khanna, K. K.; Al-Ejeh, F.; Fairlie, D. P.; Wiegmans, A. P.; Liu, L. Quinazolinone Derivatives as Inhibitors of Homologous Recombinase RAD51. *Bioorganic & Medicinal Chemistry Letters* **2017**, *27* (14), 3096–3100. https://doi.org/10.1016/j.bmcl.2017.05.039.

(5) Medellin, D. C.; Zhou, Q.; Scott, R.; Hill, R. M.; Frail, S. K.; Dasari, R.; Ontiveros, S. J.; Pelly, S. C.; van Otterlo, W. A. L.; Betancourt, T.; Shuster, C. B.; Hamel, E.; Bai, R.; LaBarbera, D. V.; Rogelj, S.; Frolova, L. V.; Kornienko, A. Novel Microtubule-Targeting 7-Deazahypoxanthines Derived from Marine Alkaloid Rigidins with Potent in Vitro and in Vivo Anticancer Activities. *J. Med. Chem.* **2016**, *59* (1), 480–485. https://doi.org/10.1021/acs.jmedchem.5b01426.

(6) Yang, F.; Zhao, N.; Ge, D.; Chen, Y. Next-Generation of Selective Histone Deacetylase Inhibitors. *RSC Adv.* **2019**, *9* (34), 19571–19583. https://doi.org/10.1039/C9RA02985K.

(7) Dong, G.; Fang, Y.; Liu, Y.; Liu, N.; Wu, S.; Zhang, W.; Sheng, C. Design, Synthesis and Evaluation of 4-Substituted Anthra[2,1-c][1,2,5]Thiadiazole-6,11-Dione Derivatives as Novel Non-Camptothecin Topoisomerase I Inhibitors. *Bioorganic & Medicinal Chemistry Letters* **2017**, *27* (9), 1929–1933. https://doi.org/10.1016/j.bmcl.2017.03.039.

(8) Verbeet, W.; Husiev, Y.; Bonnet, S. Simple and Efficient Method for Mono- and Di-Amination of Polypyridine *N* -Oxides. *Eur J Org Chem* **2024**, *27* (14), e202400054. https://doi.org/10.1002/ejoc.202400054.

(9) Sheldrick, G. M. Crystal Structure Refinement with *SHELXL*. *Acta Crystallogr C Struct Chem* **2015**, *71* (1), 3–8. https://doi.org/10.1107/S2053229614024218.

(10) Spek, A. L. Structure Validation in Chemical Crystallography. *Acta Crystallogr D Biol Crystallogr* **2009**, *65* (2), 148–155. https://doi.org/10.1107/S090744490804362X.

(11) Clark, R. C.; Reid, J. S. The Analytical Calculation of Absorption in Multifaceted Crystals. *Acta Crystallogr A Found Crystallogr* **1995**, *51* (6), 887–897. https://doi.org/10.1107/S0108767395007367.

(12) Zhou, X.-Q.; Busemann, A.; Meijer, M. S.; Siegler, M. A.; Bonnet, S. The Two Isomers of a Cyclometallated Palladium Sensitizer Show Different Photodynamic Properties in Cancer Cells. *Chem. Commun.* **2019**, *55* (32), 4695–4698. https://doi.org/10.1039/C8CC10134E.

(13) Suzuki, K.; Kobayashi, A.; Kaneko, S.; Takehira, K.; Yoshihara, T.; Ishida, H.; Shiina, Y.; Oishi, S.; Tobita, S. Reevaluation of Absolute Luminescence Quantum Yields of Standard Solutions Using a Spectrometer with an Integrating Sphere and a Back-Thinned

CCD Detector. *Phys. Chem. Chem. Phys.* **2009**, *11* (42), 9850. https://doi.org/10.1039/b912178a.
(14) Ossola, R.; Jönsson, O. M.; Moor, K.; McNeill, K. Singlet Oxygen Quantum Yields in Environmental Waters. *Chem. Rev.* **2021**, *121* (7), 4100–4146. https://doi.org/10.1021/acs.chemrev.0c00781.
(15) Partanen, S. B.; Erickson, P. R.; Latch, D. E.; Moor, K. J.; McNeill, K. Dissolved Organic Matter Singlet Oxygen Quantum Yields: Evaluation Using Time-Resolved Singlet Oxygen Phosphorescence. *Environ. Sci. Technol.* **2020**, *54* (6), 3316–3324. https://doi.org/10.1021/acs.est.9b07246.
(16) Schmidt, R.; Tanielian, C.; Dunsbach, R.; Wolff, C. Phenalenone, a Universal Reference Compound for the Determination of Quantum Yields of Singlet Oxygen O2(1Δg) Sensitization. *Journal of Photochemistry and Photobiology A: Chemistry* **1994**, *79* (1–2), 11–17. https://doi.org/10.1016/1010-6030(93)03746-4.
(17) Abdel-Shafi, A. A.; Beer, P. D.; Mortimer, R. J.; Wilkinson, F. Photosensitized Generation of Singlet Oxygen from Vinyl Linked Benzo-Crown-Ether−Bipyridyl Ruthenium(II) Complexes. *J. Phys. Chem. A* **2000**, *104* (2), 192–202. https://doi.org/10.1021/jp991876z.
(18) Hopkins, S. L.; Siewert, B.; Askes, S. H. C.; Veldhuizen, P.; Zwier, R.; Heger, M.; Bonnet, S. An in Vitro Cell Irradiation Protocol for Testing Photopharmaceuticals and the Effect of Blue, Green, and Red Light on Human Cancer Cell Lines. *Photochem Photobiol Sci* **2016**, *15* (5), 644–653. https://doi.org/10.1039/c5pp00424a.
(19) Bretin, L.; Husiev, Y.; Ramu, V.; Zhang, L.; Hakkennes, M.; Abyar, S.; Johns, A. C.; Le Dévédec, S. E.; Betancourt, T.; Kornienko, A.; Bonnet, S. Red-Light Activation of a Microtubule Polymerization Inhibitor via Amide Functionalization of the Ruthenium Photocage. *Angew Chem Int Ed* **2024**, *63* (5), e202316425. https://doi.org/10.1002/anie.202316425.
(20) Abyar, S.; Huang, L.; Husiev, Y.; Bretin, L.; Chau, B.; Ramu, V.; Wildeman, J. H.; Belfor, K.; Wijaya, L. S.; Van Der Noord, V. E.; Harms, A. C.; Siegler, M. A.; Le Dévédec, S. E.; Bonnet, S. Oxygen-Dependent Interactions between the Ruthenium Cage and the Photoreleased Inhibitor in NAMPT-Targeted Photoactivated Chemotherapy. *J. Med. Chem.* **2024**, *67* (13), 11086–11102. https://doi.org/10.1021/acs.jmedchem.4c00589.
