# Supplementary material for: A Sterically Open Ruthenium-Based Photocage Activated by Red and Far-Red Light for a Wide Range of Drugs
Source: J Am Chem Soc. 2025 Nov 18;147(48):44356–71. doi: 10.1021/jacs.5c14772 (PMC12679631; doi:10.1021/jacs.5c14772)

checkCIF/PLATON report

Structure factors have been supplied for datablock(s) 9_BF4_2

THIS REPORT IS FOR GUIDANCE ONLY. IF USED AS PART OF A REVIEW PROCEDURE FOR PUBLICATION, IT SHOULD NOT REPLACE THE EXPERTISE OF AN EXPERIENCED CRYSTALLOGRAPHIC REFEREE.

No syntax errors found. CIF dictionary Interpreting this report

Datablock: 9_BF4_2

Bond precision: C-C = 0.0068 A Wavelength=0.71073

Cell:	a=9.6576(2)	b=10.4002(2)	c=12.2969(3)
	alpha=69.155(2)	beta=77.481(2)	gamma=64.622(2)
Temperature:	110 K		

	Calculated	Reported
Volume	1039.86(4)	1039.86(4)
Space group	P 1	P 1
Hall group	P 1	P 1
Moiety formula	C40 H39 N9 O Ru, 2(B F4), C H4 O	C40 H39 N9 O Ru, 2(B F4), C H4 O
Sum formula	C41 H43 B2 F8 N9 O2 Ru	C41 H43 B2 F8 N9 O2 Ru
Mr	968.53	968.53
Dx,g cm-3	1.547	1.547
Z	1	1
Mu (mm-1)	0.462	0.462
F000	494.0	494.0
F000'	492.99	
h,k,lmax	12,13,15	12,13,15
Nref	9546[4773]	9463
Tmin,Tmax	0.891,0.954	0.903,0.961
Tmin'	0.857	

Correction method= # Reported T Limits: Tmin=0.903 Tmax=0.961
AbsCorr = ANALYTICAL

Data completeness= 1.98/0.99 Theta(max)= 27.500

R(reflections)= 0.0294(9350) wR2(reflections)= 0.0663(9463)

S = 1.028 Npar= 637

The following ALERTS were generated. Each ALERT has the format
test-name_ALERT_alert-type_alert-level.
Click on the hyperlinks for more details of the test.

Alert level C

PLAT090_ALERT_3_C Poor Data / Parameter Ratio (Zmax > 18) .... 7.49 Note
PLAT094_ALERT_2_C Ratio of Maximum / Minimum Residual Density .... 2.30 Report

Alert level G

PLAT002_ALERT_2_G Number of Distance or Angle Restraints on AtSite 18 Note
PLAT003_ALERT_2_G Number of Uiso or U(i,j) Restrained non-H-Atoms 14 Report
PLAT007_ALERT_5_G Number of Unrefined Donor-H Atoms .... 2 Report
H1S H1S'
PLAT154_ALERT_1_G The s.u.'s on the Cell Angles are Equal ..(Note) 0.002 Degree
PLAT171_ALERT_4_G The CIF-Embedded .res File Contains EADP Records 1 Report
PLAT172_ALERT_4_G The CIF-Embedded .res File Contains DFIX Records 2 Report
PLAT175_ALERT_4_G The CIF-Embedded .res File Contains SAME Records 1 Report
PLAT176_ALERT_4_G The CIF-Embedded .res File Contains SADI Records 1 Report
PLAT178_ALERT_4_G The CIF-Embedded .res File Contains SIMU Records 2 Report
PLAT188_ALERT_3_G A Non-default SIMU Restraint Value has been used 0.0100 Report
PLAT188_ALERT_3_G A Non-default SIMU Restraint Value has been used 0.0100 Report
PLAT191_ALERT_3_G A Non-default SADI Restraint Value has been used 0.0100 Report
PLAT244_ALERT_4_G Low 'Solvent' Ueq as Compared to Neighbors of B2 Check
PLAT302_ALERT_4_G Anion/Solvent/Minor-Residue Disorder (Resd 2) 100% Note
PLAT302_ALERT_4_G Anion/Solvent/Minor-Residue Disorder (Resd 4) 100% Note
PLAT302_ALERT_4_G Anion/Solvent/Minor-Residue Disorder (Resd 5) 100% Note
PLAT302_ALERT_4_G Anion/Solvent/Minor-Residue Disorder (Resd 6) 100% Note
PLAT304_ALERT_4_G Non-Integer Number of Atoms in ..... (Resd 2) 4.19 Check
PLAT304_ALERT_4_G Non-Integer Number of Atoms in ..... (Resd 4) 0.81 Check
PLAT304_ALERT_4_G Non-Integer Number of Atoms in ..... (Resd 5) 4.16 Check
PLAT304_ALERT_4_G Non-Integer Number of Atoms in ..... (Resd 6) 1.84 Check
PLAT432_ALERT_2_G Short Inter X...Y Contact F1' ..C1 . 2.91 Ang.
x,y,z = 1_555 Check
PLAT720_ALERT_4_G Number of Unusual/Non-Standard Labels .... 9 Note
H1S1 H1S2 H1S3 C1S' H1S4 H1S5 H1S6 O1S'
H1S'
PLAT794_ALERT_5_G Tentative Bond Valency for Ru1 (III) . 3.16 Info
PLAT860_ALERT_3_G Number of Least-Squares Restraints .... 184 Note
PLAT899_ALERT_4_G SHELXL2018 is Outdated and Succeeded by SHELXL 2019/3 Note
PLAT910_ALERT_3_G Missing FCF Reflection(s) Below Theta(Min)[Deg]= 2.27 Note
0 0 1,
PLAT967_ALERT_5_G Note: Two-Theta Cutoff Value in Embedded .res .. 55.0 Degree
PLAT969_ALERT_5_G The 'Henn et al.' R-Factor-gap value .... 2.084 Note
Predicted wR2: Based on SigI**2 3.18 or SHELX Weight 6.45
PLAT978_ALERT_2_G Number C-C Bonds with Positive Residual Density. 3 Info

0 **ALERT level A** = Most likely a serious problem - resolve or explain
0 **ALERT level B** = A potentially serious problem, consider carefully
2 **ALERT level C** = Check. Ensure it is not caused by an omission or oversight
30 **ALERT level G** = General information/check it is not something unexpected

1 ALERT type 1 CIF construction/syntax error, inconsistent or missing data

```
 5 ALERT type 2 Indicator that the structure model may be wrong or deficient
 6 ALERT type 3 Indicator that the structure quality may be low
16 ALERT type 4 Improvement, methodology, query or suggestion
 4 ALERT type 5 Informative message, check
```

It is advisable to attempt to resolve as many as possible of the alerts in all categories. Often the minor alerts point to easily fixed oversights, errors and omissions in your CIF or refinement strategy, so attention to these fine details can be worthwhile. In order to resolve some of the more serious problems it may be necessary to carry out additional measurements or structure refinements. However, the purpose of your study may justify the reported deviations and the more serious of these should normally be commented upon in the discussion or experimental section of a paper or in the "special_details" fields of the CIF. checkCIF was carefully designed to identify outliers and unusual parameters, but every test has its limitations and alerts that are not important in a particular case may appear. Conversely, the absence of alerts does not guarantee there are no aspects of the results needing attention. It is up to the individual to critically assess their own results and, if necessary, seek expert advice.

Publication of your CIF in IUCr journals

A basic structural check has been run on your CIF. These basic checks will be run on all CIFs submitted for publication in IUCr journals (*Acta Crystallographica*, *Journal of Applied Crystallography*, *Journal of Synchrotron Radiation*); however, if you intend to submit to *Acta Crystallographica Section C* or *E* or *IUCrData*, you should make sure that full publication checks are run on the final version of your CIF prior to submission.

Publication of your CIF in other journals

Please refer to the *Notes for Authors* of the relevant journal for any special instructions relating to CIF submission.

PLATON version of 04/06/2025; check.def file version of 30/05/2025

Datablock 9_BF4_2 - ellipsoid plot

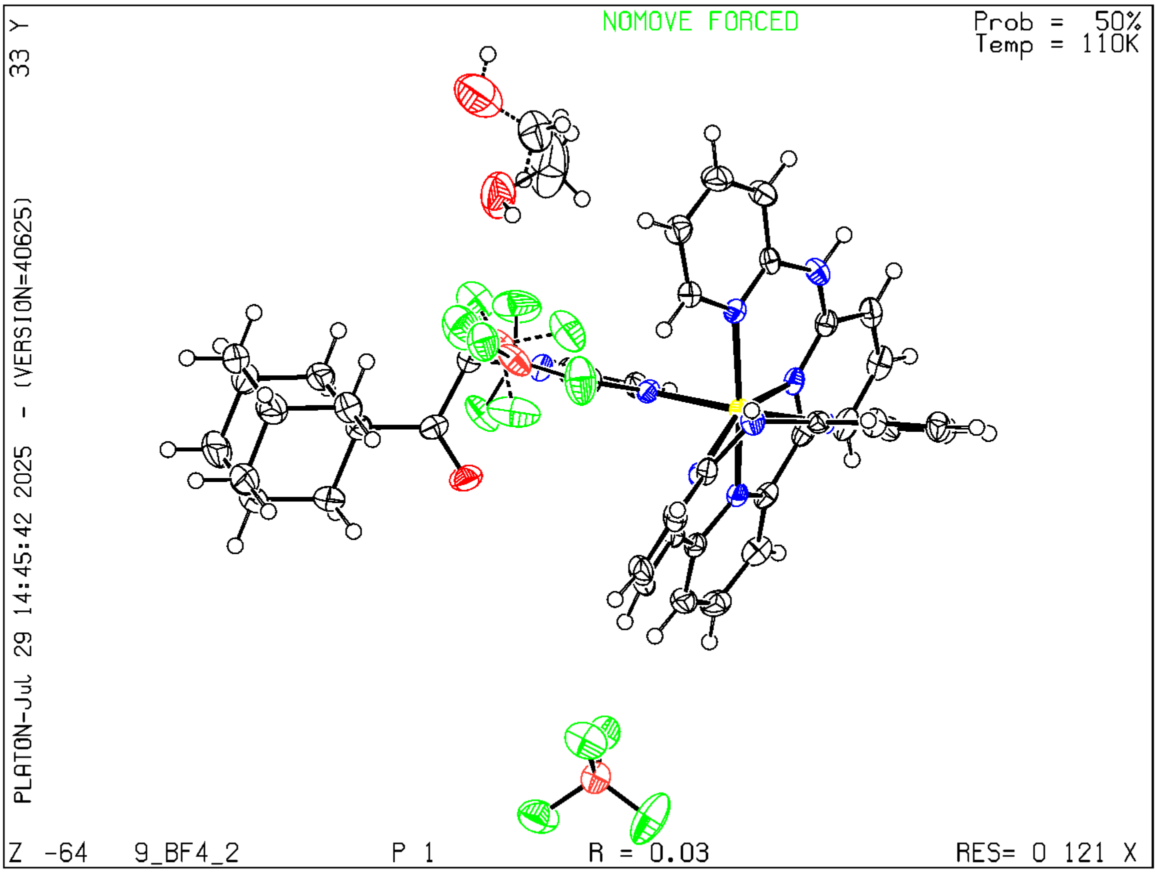

Supplement: Supplementary file 2 [file ja5c14772_si_002.zip › XRD/9_BF4_2_checkCIF_report.pdf]
