# Supplementary material for: A Sterically Open Ruthenium-Based Photocage Activated by Red and Far-Red Light for a Wide Range of Drugs
Source: J Am Chem Soc. 2025 Nov 18;147(48):44356–71. doi: 10.1021/jacs.5c14772 (PMC12679631; doi:10.1021/jacs.5c14772)

checkCIF/PLATON report

Structure factors have been supplied for datablock(s) 9_PF6_2

THIS REPORT IS FOR GUIDANCE ONLY. IF USED AS PART OF A REVIEW PROCEDURE FOR PUBLICATION, IT SHOULD NOT REPLACE THE EXPERTISE OF AN EXPERIENCED CRYSTALLOGRAPHIC REFEREE.

No syntax errors found. CIF dictionary Interpreting this report

Datablock: 9_PF6_2

Bond precision: C-C = 0.0108 A Wavelength=0.71073

Cell:	a=9.7515(4)	b=11.2867(6)	c=11.9061(6)
	alpha=66.599(5)	beta=82.851(4)	gamma=71.056(4)
Temperature:	110 K		

	Calculated	Reported
Volume	1137.47(11)	1137.47(11)
Space group	P 1	P 1
Hall group	P 1	P 1
Moiety formula	C40 H39 N9 O Ru, 2(F6 P), C3 H6 O	C40 H39 N9 O Ru, 2(F6 P), C3 H6 O
Sum formula	C43 H45 F12 N9 O2 P2 Ru	C43 H45 F12 N9 O2 P2 Ru
Mr	1110.89	1110.89
Dx,g cm-3	1.622	1.622
Z	1	1
Mu (mm-1)	0.514	0.514
F000	564.0	564.0
F000'	563.27	
h,k,lmax	12,13,14	12,13,14
Nref	8936[4468]	8793
Tmin,Tmax	0.948,0.969	0.614,1.000
Tmin'	0.948	

Correction method= # Reported T Limits: Tmin=0.614 Tmax=1.000
AbsCorr = MULTI-SCAN

Data completeness= 1.97/0.98 Theta(max)= 26.000

R(reflections)= 0.0470(8072) wR2(reflections)= 0.0836(8793)

S = 1.017 Npar= 630

The following ALERTS were generated. Each ALERT has the format
test-name_ALERT_alert-type_alert-level.
Click on the hyperlinks for more details of the test.

Alert level C

```
PLAT090_ALERT_3_C Poor Data / Parameter Ratio (Zmax > 18) ........       7.09 Note
PLAT250_ALERT_2_C Large U3/U1 Ratio for <U(i,j)> Tensor(Resd    3)        2.3 Note
PLAT342_ALERT_3_C Low Bond Precision on  C-C Bonds ...............    0.01085 Ang.
```

Alert level G

```
PLAT002_ALERT_2_G Number of Distance or Angle Restraints on AtSite          4 Note
PLAT172_ALERT_4_G The CIF-Embedded .res File Contains DFIX Records          1 Report
PLAT244_ALERT_4_G Low    'Solvent' Ueq as Compared to Neighbors of         P1 Check
PLAT244_ALERT_4_G Low    'Solvent' Ueq as Compared to Neighbors of         P2 Check
PLAT720_ALERT_4_G Number of Unusual/Non-Standard Labels ..........          6 Note
              H1S1    H1S2    H1S3    H3S1    H3S2    H3S3
PLAT794_ALERT_5_G Tentative Bond Valency for Ru1       (III)     .       3.13 Info
PLAT860_ALERT_3_G Number of Least-Squares Restraints .............          5 Note
PLAT899_ALERT_4_G SHELXL2018 is Outdated   and Succeeded by SHELXL     2019/3 Note
PLAT910_ALERT_3_G Missing FCF Reflection(s) Below Theta(Min)[Deg]=       2.06 Note
                0  0  1,
PLAT941_ALERT_3_G Average HKL Measurement Multiplicity ...........        4.3 Low
PLAT967_ALERT_5_G Note: Two-Theta Cutoff Value in Embedded .res ..       52.0 Degree
PLAT969_ALERT_5_G The 'Henn et al.' R-Factor-gap value ..........       1.103 Note
              Predicted wR2: Based on SigI**2  7.57 or SHELX Weight  8.22
PLAT978_ALERT_2_G Number C-C Bonds with Positive Residual Density.          2 Info
```

0 **ALERT level A** = Most likely a serious problem - resolve or explain
0 **ALERT level B** = A potentially serious problem, consider carefully
3 **ALERT level C** = Check. Ensure it is not caused by an omission or oversight
13 **ALERT level G** = General information/check it is not something unexpected

0 ALERT type 1 CIF construction/syntax error, inconsistent or missing data
3 ALERT type 2 Indicator that the structure model may be wrong or deficient
5 ALERT type 3 Indicator that the structure quality may be low
5 ALERT type 4 Improvement, methodology, query or suggestion
3 ALERT type 5 Informative message, check

It is advisable to attempt to resolve as many as possible of the alerts in all categories. Often the minor alerts point to easily fixed oversights, errors and omissions in your CIF or refinement strategy, so attention to these fine details can be worthwhile. In order to resolve some of the more serious problems it may be necessary to carry out additional measurements or structure refinements. However, the purpose of your study may justify the reported deviations and the more serious of these should normally be commented upon in the discussion or experimental section of a paper or in the "special_details" fields of the CIF. checkCIF was carefully designed to identify outliers and unusual parameters, but every test has its limitations and alerts that are not important in a particular case may appear. Conversely, the absence of alerts does not guarantee there are no aspects of the results needing attention. It is up to the individual to critically assess their own results and, if necessary, seek expert advice.

Publication of your CIF in IUCr journals

A basic structural check has been run on your CIF. These basic checks will be run on all CIFs submitted for publication in IUCr journals (*Acta Crystallographica*, *Journal of Applied Crystallography*, *Journal of Synchrotron Radiation*); however, if you intend to submit to *Acta Crystallographica Section C* or *E* or *IUCrData*, you should make sure that full publication checks are run on the final version of your CIF prior to submission.

Publication of your CIF in other journals

Please refer to the *Notes for Authors* of the relevant journal for any special instructions relating to CIF submission.

PLATON version of 04/06/2025; check.def file version of 30/05/2025

Datablock 9_PF6_2 - ellipsoid plot

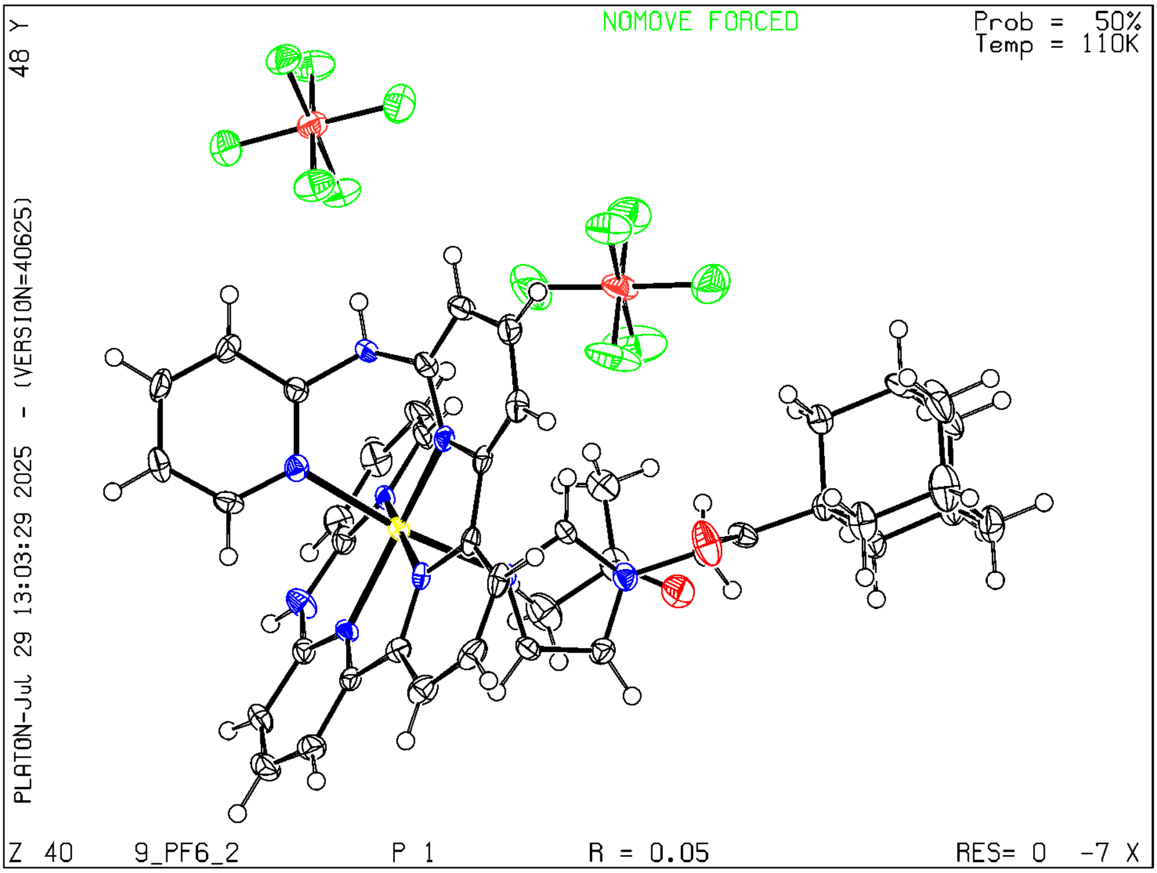

Supplement: Supplementary file 2 [file ja5c14772_si_002.zip › XRD/9_PF6_2_checkCIF_report.pdf]
