# Supplementary material for: A Sterically Open Ruthenium-Based Photocage Activated by Red and Far-Red Light for a Wide Range of Drugs
Source: J Am Chem Soc. 2025 Nov 18;147(48):44356–71. doi: 10.1021/jacs.5c14772 (PMC12679631; doi:10.1021/jacs.5c14772)

checkCIF/PLATON report

Structure factors have been supplied for datablock(s) 14_PF6_2

THIS REPORT IS FOR GUIDANCE ONLY. IF USED AS PART OF A REVIEW PROCEDURE FOR PUBLICATION, IT SHOULD NOT REPLACE THE EXPERTISE OF AN EXPERIENCED CRYSTALLOGRAPHIC REFEREE.

No syntax errors found. CIF dictionary Interpreting this report

Datablock: 14_PF6_2

Bond precision:	C-C = 0.0090 A		Wavelength=1.54178
Cell:	a=9.2424(5)	b=14.0980(5)	c=20.5291(8)
	alpha=87.006(3)	beta=79.279(4)	gamma=70.961(4)
Temperature:	250 K		

	Calculated	Reported
Volume	2484.4(2)	2484.4(2)
Space group	P -1	P -1
Hall group	-P 1	-P 1
Moiety formula	C37 H34 N10 O2 Ru S, 2(F6 P), 0.58(C3 D6 O), 0.42(C H3 O) [+ so	C37 H34 N10 O2 Ru S, 2(F6 P), 0.58(C3 D6 O), 0.42(C H3 O)
Sum formula	C39.16 H35.26 D3.48 F12 N10 O3 P2 Ru S [+ solvent]	C39.16 H35.26 D3.47 F12 N10 O3 P2 Ru S
Mr	1124.02	1123.95
Dx,g cm-3	1.503	1.502
Z	2	2
Mu (mm-1)	4.379	4.379
F000	1131.4	1131.0
F000'	1137.29	
h,k,lmax	11,16,24	11,16,24
Nref	8875	8852
Tmin,Tmax	0.896,0.916	0.555,0.934
Tmin'	0.362	

Correction method= # Reported T Limits: Tmin=0.555 Tmax=0.934
AbsCorr = ANALYTICAL

Data completeness= 0.997 Theta(max)= 67.250

R(reflections)= 0.0596(6825)

wR2(reflections)= 0.1693(8852)

S = 1.038 Npar= 811

The following ALERTS were generated. Each ALERT has the format
test-name_ALERT_alert-type_alert-level.
Click on the hyperlinks for more details of the test.

Alert level C

```
PLAT094_ALERT_2_C Ratio of Maximum / Minimum Residual Density ....       3.64 Report
PLAT220_ALERT_2_C NonSolvent   Resd 1   C   Ueq(max)/Ueq(min)  Range      3.1 Ratio
PLAT234_ALERT_4_C Large Hirshfeld Difference P2       --F10       .      0.18 Ang.
PLAT260_ALERT_2_C Large Average Ueq of Residue Including        P1      0.101 Check
PLAT260_ALERT_2_C Large Average Ueq of Residue Including        P2      0.142 Check
PLAT260_ALERT_2_C Large Average Ueq of Residue Including       P2'      0.145 Check
PLAT260_ALERT_2_C Large Average Ueq of Residue Including       O2S      0.197 Check
PLAT260_ALERT_2_C Large Average Ueq of Residue Including       O1S      0.183 Check
PLAT309_ALERT_2_C Single Bonded Oxygen (C-O > 1.3 Ang) ..........         O1S Check
PLAT342_ALERT_3_C Low Bond Precision on  C-C Bonds ...............    0.00896 Ang.
PLAT911_ALERT_3_C Missing FCF Refl Between Thmin & STh/L=    0.598         22 Report
                2  2  0,  -2  4  0,   1 10  0,  -1 15  0,   1-15  1,  -4  0  1,
                6  1  2,   9 13  4,  -2 -1  5,  10 -1  6,   0  5  8,  -7-11  9,
                0  2 10,   4  4 11,   4  5 11,   3  4 12,  -1 11 16,  -5  2 18,
                1 -5 19,   8  2 19,   1 -5 20,   2 -6 21,
PLAT971_ALERT_2_C Check Calcd Resid. Dens.  0.88Ang From Ru1             1.77 eA-3
```

Alert level G

```
PLAT002_ALERT_2_G Number of Distance or Angle Restraints on AtSite         46 Note
PLAT003_ALERT_2_G Number of Uiso or U(i,j)  Restrained non-H-Atoms         52 Report
PLAT007_ALERT_5_G Number of Unrefined Donor-H Atoms .............           4 Report
              H2A   H6    H8A   H10
PLAT041_ALERT_1_G Calc. and Reported SumFormula    Strings  Differ     Please Check
              Calc: C39.16 H35.26 D3.48 F12 N10 O3 P2 Ru S
              Rep.: C39.16 H35.26 D3.47 F12 N10 O3 P2 Ru S
PLAT168_ALERT_4_G The CIF-Embedded .res File Contains EXYZ Records          1 Report
PLAT171_ALERT_4_G The CIF-Embedded .res File Contains EADP Records          2 Report
PLAT172_ALERT_4_G The CIF-Embedded .res File Contains DFIX Records          3 Report
PLAT174_ALERT_4_G The CIF-Embedded .res File Contains FLAT Records          3 Report
PLAT175_ALERT_4_G The CIF-Embedded .res File Contains SAME Records          4 Report
PLAT176_ALERT_4_G The CIF-Embedded .res File Contains SADI Records          1 Report
PLAT178_ALERT_4_G The CIF-Embedded .res File Contains SIMU Records          5 Report
PLAT188_ALERT_3_G A Non-default SIMU Restraint Value has been used     0.0100 Report
PLAT188_ALERT_3_G A Non-default SIMU Restraint Value has been used     0.0100 Report
PLAT188_ALERT_3_G A Non-default SIMU Restraint Value has been used     0.0100 Report
PLAT188_ALERT_3_G A Non-default SIMU Restraint Value has been used     0.0100 Report
PLAT188_ALERT_3_G A Non-default SIMU Restraint Value has been used     0.0100 Report
PLAT191_ALERT_3_G A Non-default SADI Restraint Value has been used     0.0100 Report
PLAT231_ALERT_4_G Hirshfeld Test (Solvent)    P2       --F7       .       6.6 s.u.
PLAT301_ALERT_3_G Main Residue  Disorder .............(Resd    1)         12% Note
PLAT302_ALERT_4_G Anion/Solvent/Minor-Residue Disorder (Resd    2)       100% Note
PLAT302_ALERT_4_G Anion/Solvent/Minor-Residue Disorder (Resd    3)       100% Note
PLAT302_ALERT_4_G Anion/Solvent/Minor-Residue Disorder (Resd    4)       100% Note
PLAT302_ALERT_4_G Anion/Solvent/Minor-Residue Disorder (Resd    5)       100% Note
```

```
PLAT302_ALERT_4_G Anion/Solvent/Minor-Residue Disorder (Resd    6)       100% Note
PLAT302_ALERT_4_G Anion/Solvent/Minor-Residue Disorder (Resd    7)       100% Note
PLAT304_ALERT_4_G Non-Integer Number of Atoms in ..... (Resd    2)       4.27 Check
PLAT304_ALERT_4_G Non-Integer Number of Atoms in ..... (Resd    3)       3.89 Check
PLAT304_ALERT_4_G Non-Integer Number of Atoms in ..... (Resd    4)       2.73 Check
PLAT304_ALERT_4_G Non-Integer Number of Atoms in ..... (Resd    5)       3.11 Check
PLAT304_ALERT_4_G Non-Integer Number of Atoms in ..... (Resd    6)       5.80 Check
PLAT304_ALERT_4_G Non-Integer Number of Atoms in ..... (Resd    7)       2.10 Check
PLAT380_ALERT_4_G Incorrectly? Oriented X(sp2)-Methyl Moiety .....        C2S Check
PLAT380_ALERT_4_G Incorrectly? Oriented X(sp2)-Methyl Moiety .....        C4S Check
PLAT432_ALERT_2_G Short Inter X...Y Contact  F12'     ..C37      .       2.95 Ang.
                                               1-x,1-y,1-z  =      2_666 Check
PLAT432_ALERT_2_G Short Inter X...Y Contact  C13      ..C35      .       3.07 Ang.
                                               1-x,1-y,1-z  =      2_666 Check
PLAT605_ALERT_4_G Largest Solvent Accessible VOID in the Structure        257 A**3
PLAT720_ALERT_4_G Number of Unusual/Non-Standard Labels ..........          9 Note
              H1S1    H1S2    H1S3    D2S1    D2S2    D2S3    D4S1    D4S2
              D4S3
PLAT721_ALERT_1_G Bond    Calc     0.96000, Rep     0.97000 Dev...       0.01 Ang.
              C1S      -H1S2           1_555   1_555 ........     #  134 Check
PLAT790_ALERT_4_G Centre of Gravity not Within Unit Cell: Resd.  #          3 Note
              F6 P
PLAT790_ALERT_4_G Centre of Gravity not Within Unit Cell: Resd.  #          5 Note
              F6 P
PLAT790_ALERT_4_G Centre of Gravity not Within Unit Cell: Resd.  #          6 Note
              C3 D6 O
PLAT811_ALERT_5_G No ADDSYM Analysis: Too Many Excluded Atoms ....          ! Info
PLAT860_ALERT_3_G Number of Least-Squares Restraints ............         846 Note
PLAT869_ALERT_4_G ALERTS Related to the Use of SQUEEZE  Suppressed          ! Info
PLAT899_ALERT_4_G SHELXL2018 is Outdated   and Succeeded by SHELXL     2019/3 Note
PLAT909_ALERT_3_G Percentage of I>2sig(I) Data at Theta(Max) Still        54% Note
PLAT910_ALERT_3_G Missing FCF Reflection(s) Below Theta(Min)[Deg]=       3.32 Note
                0  0  1,
PLAT941_ALERT_3_G Average HKL Measurement Multiplicity ..........         4.1 Low
PLAT965_ALERT_2_G The SHELXL WEIGHT Optimisation has not Converged     Please Check
PLAT967_ALERT_5_G Note: Two-Theta Cutoff Value in Embedded .res ..      134.5 Degree
PLAT969_ALERT_5_G The 'Henn et al.' R-Factor-gap value ..........      3.001 Note
              Predicted wR2: Based on SigI**2  5.64 or SHELX Weight 16.31
PLAT978_ALERT_2_G Number C-C Bonds with Positive Residual Density.          1 Info
```

```
  0 ALERT level A = Most likely a serious problem - resolve or explain
  0 ALERT level B = A potentially serious problem, consider carefully
 12 ALERT level C = Check. Ensure it is not caused by an omission or oversight
 52 ALERT level G = General information/check it is not something unexpected

  2 ALERT type 1 CIF construction/syntax error, inconsistent or missing data
 15 ALERT type 2 Indicator that the structure model may be wrong or deficient
 13 ALERT type 3 Indicator that the structure quality may be low
 30 ALERT type 4 Improvement, methodology, query or suggestion
  4 ALERT type 5 Informative message, check
```

It is advisable to attempt to resolve as many as possible of the alerts in all categories. Often the minor alerts point to easily fixed oversights, errors and omissions in your CIF or refinement strategy, so attention to these fine details can be worthwhile. In order to resolve some of the more serious problems it may be necessary to carry out additional measurements or structure refinements. However, the purpose of your study may justify the reported deviations and the more serious of these should normally be commented upon in the discussion or experimental section of a paper or in the "special_details" fields of the CIF. checkCIF was carefully designed to identify outliers and unusual parameters, but every test has its limitations and alerts that are not important in a particular case may appear. Conversely, the absence of alerts does not guarantee there are no aspects of the results needing attention. It is up to the individual to critically assess their own results and, if necessary, seek expert advice.

Publication of your CIF in IUCr journals

A basic structural check has been run on your CIF. These basic checks will be run on all CIFs submitted for publication in IUCr journals (*Acta Crystallographica*, *Journal of Applied Crystallography*, *Journal of Synchrotron Radiation*); however, if you intend to submit to *Acta Crystallographica Section C* or *E* or *IUCrData*, you should make sure that full publication checks are run on the final version of your CIF prior to submission.

Publication of your CIF in other journals

Please refer to the *Notes for Authors* of the relevant journal for any special instructions relating to CIF submission.

PLATON version of 04/06/2025; check.def file version of 30/05/2025

Datablock 14_PF6_2 - ellipsoid plot

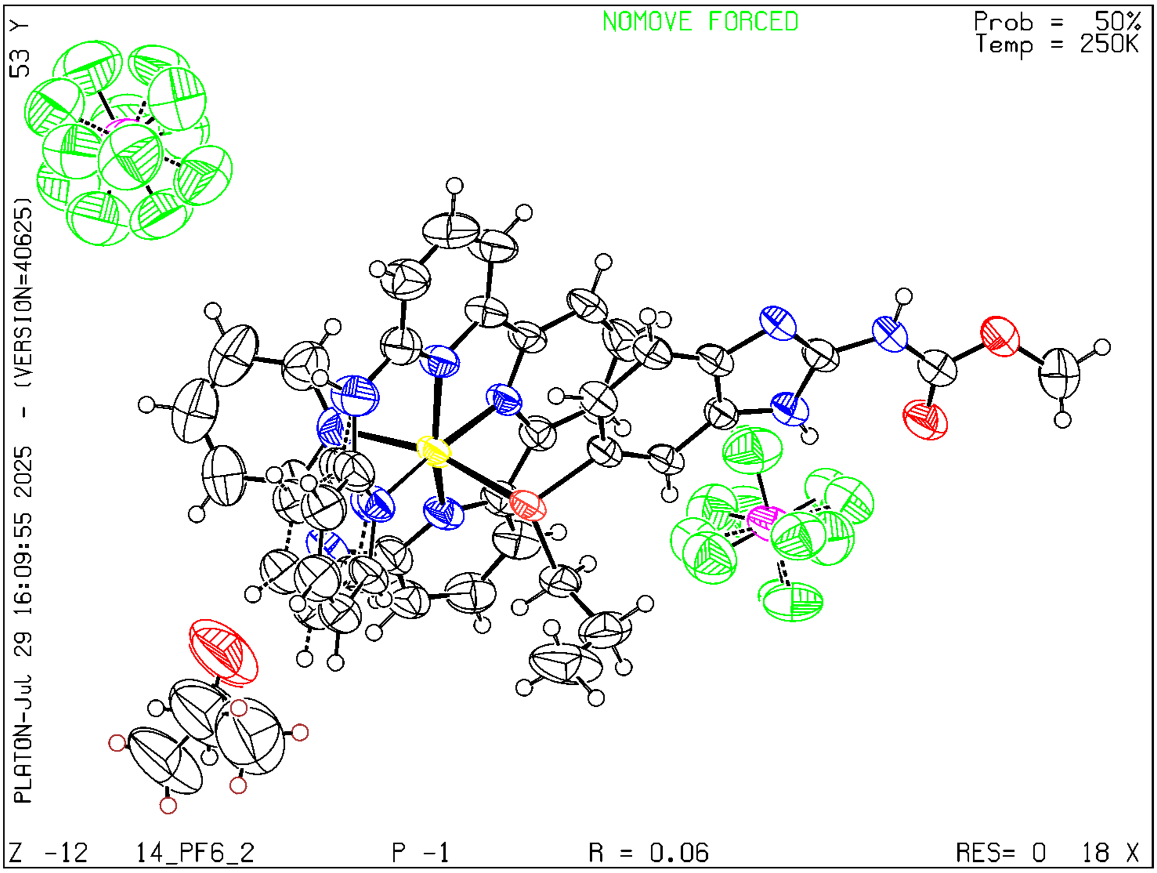

Supplement: Supplementary file 2 [file ja5c14772_si_002.zip › XRD/14_PF6_2_checkCIF_report.pdf]
