# Supplementary material for: A Sterically Open Ruthenium-Based Photocage Activated by Red and Far-Red Light for a Wide Range of Drugs
Source: J Am Chem Soc. 2025 Nov 18;147(48):44356–71. doi: 10.1021/jacs.5c14772 (PMC12679631; doi:10.1021/jacs.5c14772)

checkCIF/PLATON report

Structure factors have been supplied for datablock(s) 17_PF6_ClO4

THIS REPORT IS FOR GUIDANCE ONLY. IF USED AS PART OF A REVIEW PROCEDURE FOR PUBLICATION, IT SHOULD NOT REPLACE THE EXPERTISE OF AN EXPERIENCED CRYSTALLOGRAPHIC REFEREE.

No syntax errors found. CIF dictionary Interpreting this report

Datablock: 17_PF6_ClO4

Bond precision: C-C = 0.0088 A Wavelength=1.54178

Cell:	a=8.2958(6)	b=11.5374(8)	c=18.2115(13)
	alpha=83.572(6)	beta=78.365(6)	gamma=71.903(6)
Temperature:	110 K		

	Calculated	Reported
Volume	1620.5(2)	1620.5(2)
Space group	P -1	P -1
Hall group	-P 1	-P 1
Moiety formula	C27 H22 N8 Ru, 1.279(F6 P), 0.721(Cl O4), C2 H3 N	C27 H22 N8 Ru, 1.279(F6 P), 0.721(Cl O4), C2 H3 N
Sum formula	C29 H25 Cl0.72 F7.67 N9 O2.88 P1.28 Ru	C29 H25 Cl0.72 F7.67 N9 O2.88 P1.28 Ru
Mr	857.77	857.62
Dx,g cm-3	1.758	1.758
Z	2	2
Mu (mm-1)	5.904	5.904
F000	859.2	859.0
F000'	863.45	
h,k,lmax	9,13,21	9,13,21
Nref	5810	5792
Tmin,Tmax	0.809,0.921	0.772,0.931
Tmin'	0.685	

Correction method= # Reported T Limits: Tmin=0.772 Tmax=0.931
AbsCorr = ANALYTICAL

Data completeness= 0.997 Theta(max)= 67.242

R(reflections)= 0.0573(5356)

wR2(reflections)= 0.1390(5792)

S = 1.098   Npar= 532

The following ALERTS were generated. Each ALERT has the format
test-name_ALERT_alert-type_alert-level.
Click on the hyperlinks for more details of the test.

Alert level C

```
PLAT077_ALERT_4_C Unit Cell Contains Non-integer Number of Atoms .     Please Check
PLAT342_ALERT_3_C Low Bond Precision on  C-C Bonds ...............    0.00875 Ang.
PLAT906_ALERT_3_C Large K Value in the Analysis of Variance ......      3.350 Check
PLAT911_ALERT_3_C Missing FCF Refl Between Thmin & STh/L=    0.598         17 Report
               -9  1  0,   7 -5  3,   6 13  3,   6 -6  4,  -9 -4  4,  -9 -3  4,
                8 -4  5,   1 11  5,  -7 -6  6,  -7 -5  6,  -5  0 16,  -3 -5 17,
                7  3 18,   7  4 18,   6  3 19,   6  4 19,   2  5 21,
PLAT971_ALERT_2_C Check Calcd Resid. Dens.  0.81Ang From Ru1            1.93 eA-3
PLAT971_ALERT_2_C Check Calcd Resid. Dens.  0.82Ang From Ru1            1.70 eA-3
PLAT972_ALERT_2_C Check Calcd Resid. Dens.  0.84Ang From Ru1           -2.17 eA-3
PLAT972_ALERT_2_C Check Calcd Resid. Dens.  0.79Ang From Ru1           -1.98 eA-3
PLAT972_ALERT_2_C Check Calcd Resid. Dens.  0.75Ang From Ru1           -1.97 eA-3
```

Alert level G

```
PLAT002_ALERT_2_G Number of Distance or Angle Restraints on AtSite         18 Note
PLAT003_ALERT_2_G Number of Uiso or U(i,j)  Restrained non-H-Atoms         12 Report
PLAT068_ALERT_1_G Reported F000 Differs from Calcd (or Missing)...     Please Check
PLAT083_ALERT_2_G SHELXL Second Parameter in WGHT  Unusually Large      11.14 Why ?
PLAT154_ALERT_1_G The s.u.'s on the Cell Angles are Equal ..(Note)      0.006 Degree
PLAT172_ALERT_4_G The CIF-Embedded .res File Contains DFIX Records          2 Report
PLAT175_ALERT_4_G The CIF-Embedded .res File Contains SAME Records          1 Report
PLAT177_ALERT_4_G The CIF-Embedded .res File Contains DELU Records          1 Report
PLAT178_ALERT_4_G The CIF-Embedded .res File Contains SIMU Records          1 Report
PLAT188_ALERT_3_G A Non-default SIMU Restraint Value has been used     0.0100 Report
PLAT192_ALERT_3_G A Non-default DELU Restraint Value for First Par     0.0050 Report
PLAT244_ALERT_4_G Low    'Solvent' Ueq as Compared to Neighbors of         P1 Check
PLAT302_ALERT_4_G Anion/Solvent/Minor-Residue Disorder (Resd    3)       100% Note
PLAT302_ALERT_4_G Anion/Solvent/Minor-Residue Disorder (Resd    4)       100% Note
PLAT304_ALERT_4_G Non-Integer Number of Atoms in ..... (Resd    3)       1.95 Check
PLAT304_ALERT_4_G Non-Integer Number of Atoms in ..... (Resd    4)       3.61 Check
PLAT432_ALERT_2_G Short Inter X...Y Contact  O4       ..C4       .       2.99 Ang.
                                           1-x,1-y,-z  =      2_665 Check
PLAT720_ALERT_4_G Number of Unusual/Non-Standard Labels .........           3 Note
              H1S1    H1S2    H1S3
PLAT794_ALERT_5_G Tentative Bond Valency for Ru1       (III)     .       3.23 Info
PLAT860_ALERT_3_G Number of Least-Squares Restraints .............        234 Note
PLAT909_ALERT_3_G Percentage of I>2sig(I) Data at Theta(Max) Still        89% Note
PLAT910_ALERT_3_G Missing FCF Reflection(s) Below Theta(Min)[Deg]=       4.04 Note
                0  0  1,
PLAT941_ALERT_3_G Average HKL Measurement Multiplicity ..........         4.8 Low
PLAT967_ALERT_5_G Note: Two-Theta Cutoff Value in Embedded .res ..      134.5 Degree
PLAT969_ALERT_5_G The 'Henn et al.' R-Factor-gap value ..........       4.239 Note
              Predicted wR2: Based on SigI**2  3.28 or SHELX Weight 12.66
PLAT978_ALERT_2_G Number C-C Bonds with Positive Residual Density.          0 Info
```

```
 0 ALERT level A = Most likely a serious problem - resolve or explain
 0 ALERT level B = A potentially serious problem, consider carefully
 9 ALERT level C = Check. Ensure it is not caused by an omission or oversight
26 ALERT level G = General information/check it is not something unexpected

 2 ALERT type 1 CIF construction/syntax error, inconsistent or missing data
10 ALERT type 2 Indicator that the structure model may be wrong or deficient
 9 ALERT type 3 Indicator that the structure quality may be low
11 ALERT type 4 Improvement, methodology, query or suggestion
 3 ALERT type 5 Informative message, check
```

It is advisable to attempt to resolve as many as possible of the alerts in all categories. Often the minor alerts point to easily fixed oversights, errors and omissions in your CIF or refinement strategy, so attention to these fine details can be worthwhile. In order to resolve some of the more serious problems it may be necessary to carry out additional measurements or structure refinements. However, the purpose of your study may justify the reported deviations and the more serious of these should normally be commented upon in the discussion or experimental section of a paper or in the "special_details" fields of the CIF. checkCIF was carefully designed to identify outliers and unusual parameters, but every test has its limitations and alerts that are not important in a particular case may appear. Conversely, the absence of alerts does not guarantee there are no aspects of the results needing attention. It is up to the individual to critically assess their own results and, if necessary, seek expert advice.

Publication of your CIF in IUCr journals

A basic structural check has been run on your CIF. These basic checks will be run on all CIFs submitted for publication in IUCr journals (*Acta Crystallographica*, *Journal of Applied Crystallography*, *Journal of Synchrotron Radiation*); however, if you intend to submit to *Acta Crystallographica Section C* or *E* or *IUCrData*, you should make sure that full publication checks are run on the final version of your CIF prior to submission.

Publication of your CIF in other journals

Please refer to the *Notes for Authors* of the relevant journal for any special instructions relating to CIF submission.

PLATON version of 04/06/2025; check.def file version of 30/05/2025

Datablock 17_PF6_ClO4 - ellipsoid plot

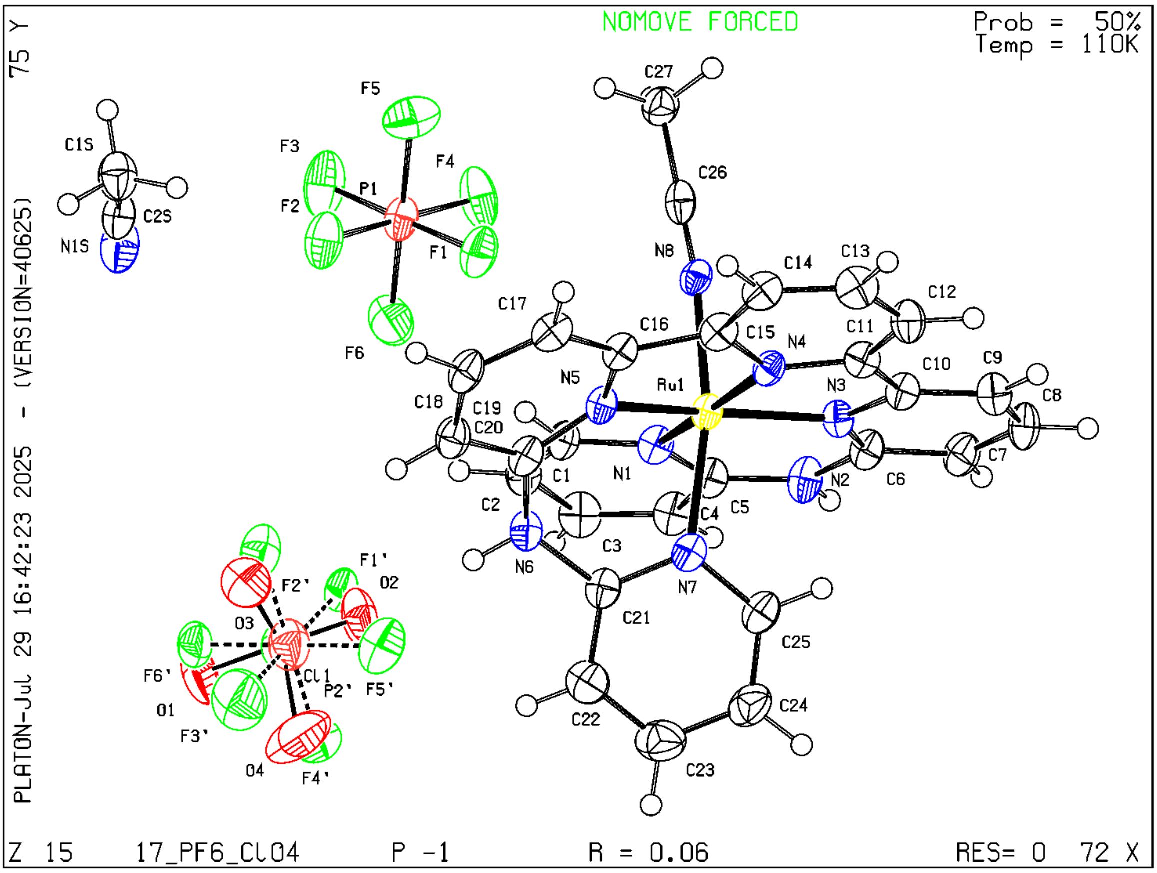

Supplement: Supplementary file 2 [file ja5c14772_si_002.zip › XRD/17_PF6_ClO4_checkCIF_report.pdf]
