# Supplementary material for: A Sterically Open Ruthenium-Based Photocage Activated by Red and Far-Red Light for a Wide Range of Drugs
Source: J Am Chem Soc. 2025 Nov 18;147(48):44356–71. doi: 10.1021/jacs.5c14772 (PMC12679631; doi:10.1021/jacs.5c14772)

# checkCIF/PLATON report

Structure factors have been supplied for datablock(s) 10_Cl_2

THIS REPORT IS FOR GUIDANCE ONLY. IF USED AS PART OF A REVIEW PROCEDURE FOR PUBLICATION, IT SHOULD NOT REPLACE THE EXPERTISE OF AN EXPERIENCED CRYSTALLOGRAPHIC REFEREE.

No syntax errors found.    CIF dictionary    Interpreting this report

# Datablock: 10_Cl_2

---

Bond precision: = 0.0000 A    Wavelength=1.54178

| | | | |
|---|---|---|---|
| Cell: | a=9.5729(5) | b=17.3947(15) | c=17.8473(10) |
| | alpha=63.221(7) | beta=75.085(5) | gamma=83.549(5) |
| Temperature: | 110 K | | |

| | Calculated | Reported |
|---|---|---|
| Volume | 2563.8(3) | 2563.7(3) |
| Space group | P -1 | P -1 |
| Hall group | -P 1 | -P 1 |
| Moiety formula | C36 H27 N9 Ru, 2(Cl) [+ solvent] | C36 H27 N9 Ru, 2(Cl) |
| Sum formula | C36 H27 Cl2 N9 Ru [+ solvent] | C36 H27 Cl2 N9 Ru |
| Mr | 757.67 | 757.63 |
| Dx,g cm-3 | 0.982 | 0.981 |
| Z | 2 | 2 |
| Mu (mm-1) | 3.651 | 3.650 |
| F000 | 768.0 | 768.0 |
| F000' | 771.37 | |
| h,k,lmax | 11,21,22 | 11,21,22 |
| Nref | 10070 | 10035 |
| Tmin,Tmax | 0.544,0.916 | 0.560,0.915 |
| Tmin' | 0.319 | |

Correction method= # Reported T Limits: Tmin=0.560 Tmax=0.915
AbsCorr = ANALYTICAL

Data completeness= 0.997    Theta(max)= 71.881

R(reflections)= 0.0761( 7703)

wR2(reflections)= 0.2443( 10035)

S = 1.059

Npar= 870

---

The following ALERTS were generated. Each ALERT has the format
**test-name_ALERT_alert-type_alert-level**.
Click on the hyperlinks for more details of the test.

---

### Alert level C

PLAT250_ALERT_2_C Large U3/U1 Ratio for <U(i,j)> Tensor(Resd 1) 2.2 Note
PLAT250_ALERT_2_C Large U3/U1 Ratio for <U(i,j)> Tensor(Resd 2) 2.2 Note
PLAT260_ALERT_2_C Large Average Ueq of Residue Including Cl1 0.121 Check
PLAT260_ALERT_2_C Large Average Ueq of Residue Including Cl2 0.127 Check
PLAT260_ALERT_2_C Large Average Ueq of Residue Including Cl2’ 0.147 Check
PLAT260_ALERT_2_C Large Average Ueq of Residue Including Cl2C 0.131 Check
PLAT905_ALERT_3_C Negative K value in the Analysis of Variance ... -0.039 Report
PLAT911_ALERT_3_C Missing FCF Refl Between Thmin & STh/L= 0.600 16 Report
-2 1 0, 1 0 2, 1 3 2, -9 11 2, 0 2 3, 0 3 3,
1 7 4, 7 -9 5, 10 1 13, 9 4 16, 9 6 17, 9 7 17,
8 4 18, 8 5 18, 0 6 20, 3 12 20,

---

### Alert level G

PLAT002_ALERT_2_G Number of Distance or Angle Restraints on AtSite 92 Note
PLAT003_ALERT_2_G Number of Uiso or U(i,j) Restrained non-H-Atoms 95 Report
PLAT007_ALERT_5_G Number of Unrefined Donor-H Atoms .............. 6 Report
H2A H6 H9A H2’1 H9’1 H6’
PLAT174_ALERT_4_G The CIF-Embedded .res File Contains FLAT Records 6 Report
PLAT175_ALERT_4_G The CIF-Embedded .res File Contains SAME Records 1 Report
PLAT176_ALERT_4_G The CIF-Embedded .res File Contains SADI Records 9 Report
PLAT178_ALERT_4_G The CIF-Embedded .res File Contains SIMU Records 2 Report
PLAT188_ALERT_3_G A Non-default SIMU Restraint Value has been used 0.0100 Report
PLAT188_ALERT_3_G A Non-default SIMU Restraint Value has been used 0.0100 Report
PLAT191_ALERT_3_G A Non-default SADI Restraint Value has been used 0.0300 Report
PLAT191_ALERT_3_G A Non-default SADI Restraint Value has been used 0.0300 Report
PLAT191_ALERT_3_G A Non-default SADI Restraint Value has been used 0.0300 Report
PLAT191_ALERT_3_G A Non-default SADI Restraint Value has been used 0.0300 Report
PLAT191_ALERT_3_G A Non-default SADI Restraint Value has been used 0.0100 Report
PLAT191_ALERT_3_G A Non-default SADI Restraint Value has been used 0.0300 Report
PLAT191_ALERT_3_G A Non-default SADI Restraint Value has been used 0.0100 Report
PLAT191_ALERT_3_G A Non-default SADI Restraint Value has been used 0.0300 Report
PLAT191_ALERT_3_G A Non-default SADI Restraint Value has been used 0.0300 Report
PLAT301_ALERT_3_G Main Residue Disorder ..............(Resd 1) 100% Note
PLAT302_ALERT_4_G Anion/Solvent/Minor-Residue Disorder (Resd 2) 100% Note
PLAT302_ALERT_4_G Anion/Solvent/Minor-Residue Disorder (Resd 4) 100% Note
PLAT302_ALERT_4_G Anion/Solvent/Minor-Residue Disorder (Resd 5) 100% Note
PLAT302_ALERT_4_G Anion/Solvent/Minor-Residue Disorder (Resd 6) 100% Note
PLAT304_ALERT_4_G Non-Integer Number of Atoms in ..... (Resd 1) 49.64 Check
PLAT304_ALERT_4_G Non-Integer Number of Atoms in ..... (Resd 2) 23.36 Check
PLAT304_ALERT_4_G Non-Integer Number of Atoms in ..... (Resd 4) 0.58 Check
PLAT304_ALERT_4_G Non-Integer Number of Atoms in ..... (Resd 5) 0.32 Check
PLAT304_ALERT_4_G Non-Integer Number of Atoms in ..... (Resd 6) 0.10 Check
PLAT606_ALERT_4_G Solvent Accessible VOID(S) in Crystal Structure ! Info
PLAT720_ALERT_4_G Number of Unusual/Non-Standard Labels .......... 2 Note

```
             H2'1    H9'1
PLAT790_ALERT_4_G Centre of Gravity not Within Unit Cell: Resd.  #          3 Note
             Cl
PLAT811_ALERT_5_G No ADDSYM Analysis: Too Many Excluded Atoms ....          ! Info
PLAT860_ALERT_3_G Number of Least-Squares Restraints ............        1811 Note
PLAT869_ALERT_4_G ALERTS Related to the Use of SQUEEZE  Suppressed          ! Info
PLAT912_ALERT_4_G Missing # of FCF Reflections Above STh/L=  0.600         19 Note
PLAT933_ALERT_2_G Number of HKL-OMIT Records in Embedded .res File          5 Note
               1  0  2,   1  3  2,   0  2  3,  -2  1  0,   0  3  3,
PLAT941_ALERT_3_G Average HKL Measurement Multiplicity ..........         3.8 Low
PLAT967_ALERT_5_G Note: Two-Theta Cutoff Value in Embedded .res ..      143.8 Degree
PLAT969_ALERT_5_G The 'Henn et al.' R-Factor-gap value ..........      7.055 Note
             Predicted wR2: Based on SigI**2  3.46 or SHELX Weight 23.06
```

---

0 **ALERT level A** = Most likely a serious problem - resolve or explain
0 **ALERT level B** = A potentially serious problem, consider carefully
8 **ALERT level C** = Check. Ensure it is not caused by an omission or oversight
39 **ALERT level G** = General information/check it is not something unexpected

0 ALERT type 1 CIF construction/syntax error, inconsistent or missing data
9 ALERT type 2 Indicator that the structure model may be wrong or deficient
16 ALERT type 3 Indicator that the structure quality may be low
18 ALERT type 4 Improvement, methodology, query or suggestion
4 ALERT type 5 Informative message, check

---

---

It is advisable to attempt to resolve as many as possible of the alerts in all categories. Often the minor alerts point to easily fixed oversights, errors and omissions in your CIF or refinement strategy, so attention to these fine details can be worthwhile. In order to resolve some of the more serious problems it may be necessary to carry out additional measurements or structure refinements. However, the purpose of your study may justify the reported deviations and the more serious of these should normally be commented upon in the discussion or experimental section of a paper or in the "special_details" fields of the CIF. checkCIF was carefully designed to identify outliers and unusual parameters, but every test has its limitations and alerts that are not important in a particular case may appear. Conversely, the absence of alerts does not guarantee there are no aspects of the results needing attention. It is up to the individual to critically assess their own results and, if necessary, seek expert advice.

**Publication of your CIF in IUCr journals**

A basic structural check has been run on your CIF. These basic checks will be run on all CIFs submitted for publication in IUCr journals (*Acta Crystallographica*, *Journal of Applied Crystallography*, *Journal of Synchrotron Radiation*); however, if you intend to submit to *Acta Crystallographica Section C* or *E* or *IUCrData*, you should make sure that full publication checks are run on the final version of your CIF prior to submission.

**Publication of your CIF in other journals**

Please refer to the *Notes for Authors* of the relevant journal for any special instructions relating to CIF submission.

---

**PLATON version of 04/06/2025; check.def file version of 30/05/2025**

**Datablock 10_Cl_2** - ellipsoid plot

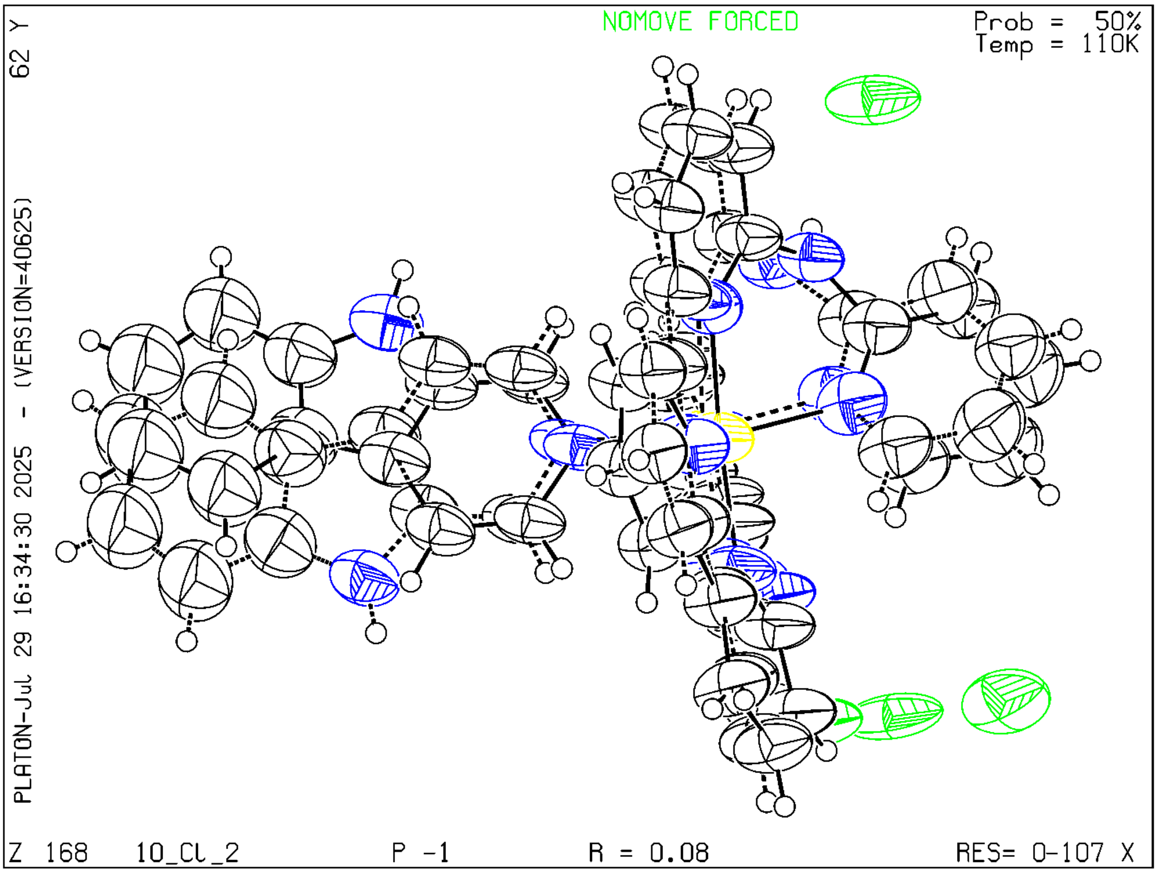

Supplement: Supplementary file 2 [file ja5c14772_si_002.zip › XRD/10_Cl_2_checkCIF_report.pdf]
