# Supplementary material for: A Sterically Open Ruthenium-Based Photocage Activated by Red and Far-Red Light for a Wide Range of Drugs
Source: J Am Chem Soc. 2025 Nov 18;147(48):44356–71. doi: 10.1021/jacs.5c14772 (PMC12679631; doi:10.1021/jacs.5c14772)
Supplement: Supplementary file 2 [file ja5c14772_si_002.zip › XRD/17_PF6_ClO4.doc]

**Experimental – XR0251a**

**To be inserted within the Acknowledgements:**

**The NIH (1S10OD030352 to M.A.S.) is gratefully acknowledged for financial support.**

All reflection intensities were measured at 110.00(10) K using a Rigaku XtaLAB Synergy R (equipped with a rotating-anode X-ray source and HyPix-6000HE detector) with Cu *K*α radiation (λ = 1.54178 Å) under the program CrysAlisPro (Version CrysAlisPro 1.171.42.49, Rigaku OD, 2022). The same program was used to refine the cell dimensions and for data reduction. The structure was solved with the program SHELXT-2018/2 (Sheldrick, 2018) and was refined on *F^2^* with SHELXL-2019/3 (Sheldrick, 2018). Analytical numeric absorption correction using a multifaceted crystal model was applied using CrysAlisPro. The temperature of the data collection was controlled using the system Cryostream 1000 from Oxford Cryosystems. The H atoms were placed at calculated positions (unless otherwise specified) using the instructions AFIX 43 or AFIX 137 with isotropic displacement parameters having values 1.2 or 1.5 *U*_eq_ of the attached C atoms. The H atoms attached to N2 and N6 were found from difference Fourier maps, and their coordinates were refined pseudofreely using the DFIX instruction in order to keep the N−H bond distances within an acceptable range.

The structure is partly disordered. There is a disordered mixture of ClO_4_^−^ (major component with occupancy of 0.721(7)) / PF_6_^−^ (minor component with occupancy of 0.279(7)) counterions found at one site in the asymmetric unit. The other PF_6_^−^ counterion is ordered and fully occupied.

**Table 1. Experimental details**

|  | XR0251a |
| --- | --- |
| Crystal data | |
| Chemical formula | C_27_H_22_N_8_Ru·1.279(F_6_P)·0.721(ClO_4_)·C_2_H_3_N |
| *M*_r_ | 857.62 |
| Crystal system, space group | Triclinic, *P*-1 |
| Temperature (K) | 110 |
| *a*, *b*, *c* (Å) | 8.2958 (6), 11.5374 (8), 18.2115 (13) |
| α, β, γ (°) | 83.572 (6), 78.365 (6), 71.903 (6) |
| *V* (Å^3^) | 1620.5 (2) |
| *Z* | 2 |
| Radiation type | Cu *K*α |
| μ (mm^-1^) | 5.90 |
| Crystal size (mm) | 0.06 × 0.03 × 0.01 |
|  | |
| Data collection | |
| Diffractometer | XtaLAB Synergy R, HyPix |
| Absorption correction | Analytical  *CrysAlis PRO* 1.171.42.95a (Rigaku Oxford Diffraction, 2023) Analytical numeric absorption correction using a multifaceted crystal model based on expressions derived by R.C. Clark & J.S. Reid. (Clark, R. C. & Reid, J. S. (1995). Acta Cryst. A51, 887-897) Empirical absorption correction using spherical harmonics, implemented in SCALE3 ABSPACK scaling algorithm. |
| *T*_min_, *T*_max_ | 0.772, 0.931 |
| No. of measured, independent and  observed [*I* > 2σ(*I*)] reflections | 28015, 5792, 5356 |
| *R*_int_ | 0.041 |
| (sin θ/λ)_max_ (Å^-1^) | 0.598 |
|  | |
| Refinement | |
| *R*[*F*^2^ > 2σ(*F*^2^)], *wR*(*F*^2^), *S* | 0.057, 0.139, 1.10 |
| No. of reflections | 5792 |
| No. of parameters | 532 |
| No. of restraints | 234 |
| H-atom treatment | H atoms treated by a mixture of independent and constrained refinement |
|  | *w* = 1/[σ^2^(*F*_o_^2^) + (0.0448*P*)^2^ + 11.1431*P*]  where *P* = (*F*_o_^2^ + 2*F*_c_^2^)/3 |
| Δρ_max_, Δρ_min_ (e Å^-3^) | 1.95, -2.21 |

Computer programs: *CrysAlis PRO* 1.171.42.49 (Rigaku OD, 2022), *SHELXT2018*/2 (Sheldrick, 2018), *SHELXL2019*/3 (Sheldrick, 2018), *SHELXTL* v6.10 (Sheldrick, 2008).

**Reference**:

Sheldrick, G. M. (2015). Acta Cryst. C71, 3-8.
