# Supplementary material for: A Sterically Open Ruthenium-Based Photocage Activated by Red and Far-Red Light for a Wide Range of Drugs
Source: J Am Chem Soc. 2025 Nov 18;147(48):44356–71. doi: 10.1021/jacs.5c14772 (PMC12679631; doi:10.1021/jacs.5c14772)
Supplement: Supplementary file 2 [file ja5c14772_si_002.zip › XRD/14_PF6_2.doc]

**Experimental – XR0013a**

**To be inserted within the Acknowledgements:**

**The NIH (1S10OD030352-01A1 to M.A.S.) is gratefully acknowledged for financial support.**

All reflection intensities were measured at 250.00(10) K using a Rigaku XtaLAB Synergy R (equipped with a rotating-anode X-ray source and HyPix-6000HE detector) with Cu *K*α radiation (λ = 1.54178 Å) under the program CrysAlisPro (Version CrysAlisPro 1.171.42.49, Rigaku OD, 2022). The same program was used to refine the cell dimensions and for data reduction. The structure was solved with the program SHELXS-2018/3 (Sheldrick, 2018) and was refined on *F^2^* with SHELXL-2018/3 (Sheldrick, 2018). Analytical numeric absorption correction using a multifaceted crystal was applied using CrysAlisPro. The temperature of the data collection was controlled using the system Cryostream 1000 from Oxford Cryosystems). The H (or D) atoms were placed at calculated positions using the instructions AFIX 23, AFIX 43 or AFIX 137 with isotropic displacement parameters having values 1.2 or 1.5 *U*_eq_ of the attached C or N atoms. The H atom attached to O1S (partially occupied MeOH) could not be retrieved from difference Fourier map.

The structure is partly disordered. The moiety N1→C5 and the two PF_6_^−^ counterions are disordered over two orientations, and the occupancy factors of the major components of the disorder refine to 0.504(13), 0.61(2) and 0.555(6), respectively. The asymmetric unit has one site that contains a mixture of lattice MeOH (occupancy: 0.42(2)) and deuterated acetone (occupancy: 0.58(2)) solvent molecules. The asymmetric unit also contains some unresolved disordered (and most likely partially occupied) lattice solvent molecule, and their contribution was removed from the final refinement using the SQUEEZE procedure in Platon (Spek, 2009).

**Table 1. Experimental details**

|  | XR0013a |
| --- | --- |
| Crystal data | |
| Chemical formula | C_37_H_34_N_10_O_2_RuS·2(F_6_P)·0.58(C_3_D_6_O)·0.42(CH_3_O) |
| *M*_r_ | 1123.95 |
| Crystal system, space group | Triclinic, *P*-1 |
| Temperature (K) | 250 |
| *a*, *b*, *c* (Å) | 9.2424 (5), 14.0980 (5), 20.5291 (8) |
| α, β, γ (°) | 87.006 (3), 79.279 (4), 70.961 (4) |
| *V* (Å^3^) | 2484.4 (2) |
| *Z* | 2 |
| Radiation type | Cu *K*α |
| μ (mm^-1^) | 4.38 |
| Crystal size (mm) | 0.23 × 0.02 × 0.02 |
|  | |
| Data collection | |
| Diffractometer | XtaLAB Synergy R, HyPix |
| Absorption correction | Analytical  *CrysAlis PRO* 1.171.42.80a (Rigaku Oxford Diffraction, 2023) Analytical numeric absorption correction using a multifaceted crystal model based on expressions derived by R.C. Clark & J.S. Reid. (Clark, R. C. & Reid, J. S. (1995). Acta Cryst. A51, 887-897) Empirical absorption correction using spherical harmonics, implemented in SCALE3 ABSPACK scaling algorithm. |
| *T*_min_, *T*_max_ | 0.555, 0.934 |
| No. of measured, independent and  observed [*I* > 2σ(*I*)] reflections | 36290, 8852, 6825 |
| *R*_int_ | 0.068 |
| (sin θ/λ)_max_ (Å^-1^) | 0.598 |
|  | |
| Refinement | |
| *R*[*F*^2^ > 2σ(*F*^2^)], *wR*(*F*^2^), *S* | 0.060, 0.169, 1.04 |
| No. of reflections | 8852 |
| No. of parameters | 811 |
| No. of restraints | 846 |
| H-atom treatment | H-atom parameters constrained |
| Δρ_max_, Δρ_min_ (e Å^-3^) | 1.83, -0.50 |

Computer programs: *CrysAlis PRO* 1.171.42.49 (Rigaku OD, 2022), *SHELXS2018*/3 (Sheldrick, 2018), *SHELXL2018*/3 (Sheldrick, 2018), *SHELXTL* v6.10 (Sheldrick, 2008).

**References**:

Sheldrick, G. M. (2015). Acta Cryst. C71, 3-8.

Spek, A.L. (2009). Acta Cryst. D65, 148-155.
