# Supplementary material for: A Sterically Open Ruthenium-Based Photocage Activated by Red and Far-Red Light for a Wide Range of Drugs
Source: J Am Chem Soc. 2025 Nov 18;147(48):44356–71. doi: 10.1021/jacs.5c14772 (PMC12679631; doi:10.1021/jacs.5c14772)
Supplement: Supplementary file 2 [file ja5c14772_si_002.zip › XRD/9_BF4_2.doc]

**Experimental – xs2986a**

All reflection intensities were measured at 110(2) K using a SuperNova diffractometer (equipped with Atlas detector) with Mo *K*α radiation (λ = 0.71073 Å) under the program CrysAlisPro (Version CrysAlisPro 1.171.42.49, Rigaku OD, 2022). The same program was used to refine the cell dimensions and for data reduction. The structure was solved with the program SHELXS-2018/3 (Sheldrick, 2018) and was refined on *F^2^* with SHELXL-2018/3 (Sheldrick, 2018). Analytical numeric absorption correction using a multifaceted crystal model was performed using CrysAlisPro. The temperature of the data collection was controlled using the system Cryojet (manufactured by Oxford Instruments). The H atoms were placed at calculated positions (unless otherwise specified) using the instructions AFIX 13, AFIX 23, AFIX 43 or AFIX 137 with isotropic displacement parameters having values 1.2 or 1.5 *U*_eq_ of the attached C atoms. The H atoms attached to N2 and N6 were found from difference Fourier maps, and their coordinates were refined pseudofreely using the DFIX instruction in order to keep the N−H bond distances within an acceptable range.

The structure is partly disordered. One of the two BF_4_^−^ counterions and the lattice MeOH solvent molecule were found to be disordered over two orientations, and the occupancy factors of the major components of the disorder refine to 0.838(9) and 0.694(10). The absolute configuration has been established by anomalous-dispersion effects in diffraction measurements on the crystal, and the Flack and Hooft parameters refine to -0.020(9) and -0.015(8), respectively.

**Table 1. Experimental details**

|  | xs2986a |
| --- | --- |
| Crystal data | |
| Chemical formula | C_40_H_39_N_9_ORu·2(BF_4_)·CH_4_O |
| *M*_r_ | 968.53 |
| Crystal system, space group | Triclinic, *P*1 |
| Temperature (K) | 110 |
| *a*, *b*, *c* (Å) | 9.6576 (2), 10.4002 (2), 12.2969 (3) |
| α, β, γ (°) | 69.155 (2), 77.481 (2), 64.622 (2) |
| *V* (Å^3^) | 1039.86 (4) |
| *Z* | 1 |
| Radiation type | Mo *K*α |
| μ (mm^-1^) | 0.46 |
| Crystal size (mm) | 0.34 × 0.21 × 0.10 |
|  | |
| Data collection | |
| Diffractometer | SuperNova, Dual, Cu at zero, Atlas |
| Absorption correction | Analytical  *CrysAlis PRO* 1.171.42.49 (Rigaku Oxford Diffraction, 2022) Analytical numeric absorption correction using a multifaceted crystal model based on expressions derived by R.C. Clark & J.S. Reid. (Clark, R. C. & Reid, J. S. (1995). Acta Cryst. A51, 887-897) Empirical absorption correction using spherical harmonics, implemented in SCALE3 ABSPACK scaling algorithm. |
| *T*_min_, *T*_max_ | 0.903, 0.961 |
| No. of measured, independent and  observed [*I* > 2σ(*I*)] reflections | 24148, 9463, 9350 |
| *R*_int_ | 0.029 |
| (sin θ/λ)_max_ (Å^-1^) | 0.650 |
|  | |
| Refinement | |
| *R*[*F*^2^ > 2σ(*F*^2^)], *wR*(*F*^2^), *S* | 0.029, 0.066, 1.03 |
| No. of reflections | 9463 |
| No. of parameters | 637 |
| No. of restraints | 184 |
| H-atom treatment | H atoms treated by a mixture of independent and constrained refinement |
| Δρ_max_, Δρ_min_ (e Å^-3^) | 0.60, -0.26 |
| Absolute structure | Flack x determined using 4480 quotients [(I+)-(I-)]/[(I+)+(I-)] (Parsons, Flack and Wagner, Acta Cryst. B69 (2013) 249-259). |
| Absolute structure parameter | -0.020 (9) |

Computer programs: *CrysAlis PRO* 1.171.42.49 (Rigaku OD, 2022), *SHELXS2018*/3 (Sheldrick, 2018), *SHELXL2018*/3 (Sheldrick, 2018), *SHELXTL* v6.10 (Sheldrick, 2008).

**References**:

Sheldrick, G. M. (2015). Acta Cryst. C71, 3-8.
