# Supplementary material for: A Sterically Open Ruthenium-Based Photocage Activated by Red and Far-Red Light for a Wide Range of Drugs
Source: J Am Chem Soc. 2025 Nov 18;147(48):44356–71. doi: 10.1021/jacs.5c14772 (PMC12679631; doi:10.1021/jacs.5c14772)
Supplement: Supplementary file 2 [file ja5c14772_si_002.zip › XRD/10_Cl2.doc]

**Experimental – XR0064a**

**To be inserted within the Acknowledgements:**

**The NIH (1S10OD030352 to M.A.S.) is gratefully acknowledged for financial support.**

All reflection intensities were measured at 110.00(10) K using a Rigaku XtaLAB Synergy R (equipped with a rotating-anode X-ray source and HyPix-6000HE detector) with Cu *K*α radiation (λ = 1.54178 Å) under the program CrysAlisPro (Version CrysAlisPro 1.171.42.49, Rigaku OD, 2022). The same program was used to refine the cell dimensions and for data reduction. The structure was solved with the program SHELXS-2018/3 (Sheldrick, 2018) and was refined on *F^2^* with SHELXL-2018/3 (Sheldrick, 2018). Analytical numeric absorption correction using a multifaceted crystal was applied using CrysAlisPro. Crystals were initially deposited on a microscope slide in some Parabar 10312 and were quickly cooled under a cold N_2_(g) stream in order to prevent any decomposition of the crystals. One single crystal was then quickly picked and mounted on the diffractometer while being flash-cooled at 110 K. The temperature of the data collection was controlled using the system Cryostream 1000 from Oxford Cryosystems. The H atoms were placed at calculated positions using the instructions AFIX 43 with isotropic displacement parameters having values 1.2 *U*_eq_ of the attached C or N atoms.

The structure is disordered. The Ru complex and one of the two Cl− counterions are disordered over two and three orientations, respectively. All occupancy factors can be retrieved from the final .cif file. The crystal lattice also contains some amount of very disordered lattice solvent molecules, and their contribution was removed from the final refinement using the SQUEEZE procedure in Platon (Spek, 2009).

**Table 1. Experimental details**

|  | xr0064a |
| --- | --- |
| Crystal data | |
| Chemical formula | C_36_H_27_N_9_Ru·2(Cl) |
| *M*_r_ | 757.63 |
| Crystal system, space group | Triclinic, *P*-1 |
| Temperature (K) | 110 |
| *a*, *b*, *c* (Å) | 9.5729 (5), 17.3947 (15), 17.8473 (10) |
| α, β, γ (°) | 63.221 (7), 75.085 (5), 83.549 (5) |
| *V* (Å^3^) | 2563.7 (3) |
| *Z* | 2 |
| Radiation type | Cu *K*α |
| μ (mm^-1^) | 3.65 |
| Crystal size (mm) | 0.30 × 0.15 × 0.02 |
|  | |
| Data collection | |
| Diffractometer | XtaLAB Synergy R, HyPix |
| Absorption correction | Analytical  *CrysAlis PRO* 1.171.42.80a (Rigaku Oxford Diffraction, 2023) Analytical numeric absorption correction using a multifaceted crystal model based on expressions derived by R.C. Clark & J.S. Reid. (Clark, R. C. & Reid, J. S. (1995). Acta Cryst. A51, 887-897) Empirical absorption correction using spherical harmonics, implemented in SCALE3 ABSPACK scaling algorithm. |
| *T*_min_, *T*_max_ | 0.560, 0.915 |
| No. of measured, independent and  observed [*I* > 2σ(*I*)] reflections | 38374, 10035, 7703 |
| *R*_int_ | 0.035 |
| (sin θ/λ)_max_ (Å^-1^) | 0.616 |
|  | |
| Refinement | |
| *R*[*F*^2^ > 2σ(*F*^2^)], *wR*(*F*^2^), *S* | 0.076, 0.244, 1.06 |
| No. of reflections | 10035 |
| No. of parameters | 870 |
| No. of restraints | 1811 |
| H-atom treatment | H-atom parameters constrained |
| Δρ_max_, Δρ_min_ (e Å^-3^) | 1.35, -0.69 |

Computer programs: *CrysAlis PRO* 1.171.42.49 (Rigaku OD, 2022), *SHELXS2018*/3 (Sheldrick, 2018), *SHELXL2018*/3 (Sheldrick, 2018), *SHELXTL* v6.10 (Sheldrick, 2008).

**References**:

Sheldrick, G. M. (2015). Acta Cryst. C71, 3-8.

Spek, A.L. (2009). Acta Cryst. D65, 148-155.
