# Supplementary material for: A Sterically Open Ruthenium-Based Photocage Activated by Red and Far-Red Light for a Wide Range of Drugs
Source: J Am Chem Soc. 2025 Nov 18;147(48):44356–71. doi: 10.1021/jacs.5c14772 (PMC12679631; doi:10.1021/jacs.5c14772)
Supplement: Supplementary file 2 [file ja5c14772_si_002.zip › XRD/4_PF6_2.doc]

**Experimental – xs2929a**

All reflection intensities were measured at 253(2) K using a SuperNova diffractometer (equipped with Atlas detector) with Mo *K*α radiation (λ = 0.71073 Å) under the program CrysAlisPro (Version CrysAlisPro 1.171.42.49, Rigaku OD, 2022). The same program was used to refine the cell dimensions and for data reduction. The structure was solved with the program SHELXS-2018/3 (Sheldrick, 2018) and was refined on *F^2^* with SHELXL-2018/3 (Sheldrick, 2018). Numerical absorption correction based on gaussian integration over a multifaceted crystal model was performed using CrysAlisPro. The temperature of the data collection was controlled using the system Cryojet (manufactured by Oxford Instruments). The H atoms were placed at calculated positions (unless otherwise specified) using the instruction AFIX 43 with isotropic displacement parameters having values 1.2 *U*_eq_ of the attached C atoms. The H atoms attached to N2 and N6 were found from difference Fourier maps, and their coordinates were refined pseudofreely using the DFIX instruction in order to keep the N−H distances within an acceptable range.

The structure is partly disordered. The PF_6_^−^ (P1→P6) counterion is found to be disordered over two orientations, and the occupancy factor of the major component of the disorder refines to 0.709(7). The asymmetric unit also contains some partially occupied and disordered amount of HPF_6_. The occupancy factors of the two components refine to 0.321(8) and 0.351(8). The location of the H atom could not be located with certainty. The asymmetric unit contains some small amount of disordered solvent molecule, and its contribution was removed from the final refinement using the SQUEEZE procedure in Platon (Spek, 2009).

**Table 1. Experimental details**

|  | xs2929a |
| --- | --- |
| Crystal data | |
| Chemical formula | C_25_H_19_ClN_7_Ru·1.672(F_6_P) |
| *M*_r_ | 796.43 |
| Crystal system, space group | Monoclinic, *I*2/*a* |
| Temperature (K) | 253 |
| *a*, *b*, *c* (Å) | 22.230 (1), 12.1936 (4), 22.8833 (12) |
| β (°) | 109.167 (5) |
| *V* (Å^3^) | 5859.0 (5) |
| *Z* | 8 |
| Radiation type | Mo *K*α |
| μ (mm^-1^) | 0.81 |
| Crystal size (mm) | 0.31 × 0.10 × 0.05 |
|  | |
| Data collection | |
| Diffractometer | SuperNova, Dual, Cu at zero, Atlas |
| Absorption correction | Gaussian  *CrysAlis PRO* 1.171.42.49 (Rigaku Oxford Diffraction, 2022) Numerical absorption correction based on gaussian integration over a multifaceted crystal model Empirical absorption correction using spherical harmonics, implemented in SCALE3 ABSPACK scaling algorithm. |
| *T*_min_, *T*_max_ | 0.740, 1.000 |
| No. of measured, independent and  observed [*I* > 2σ(*I*)] reflections | 31230, 5746, 4650 |
| *R*_int_ | 0.065 |
| (sin θ/λ)_max_ (Å^-1^) | 0.617 |
|  | |
| Refinement | |
| *R*[*F*^2^ > 2σ(*F*^2^)], *wR*(*F*^2^), *S* | 0.040, 0.105, 1.07 |
| No. of reflections | 5746 |
| No. of parameters | 563 |
| No. of restraints | 524 |
| H-atom treatment | H atoms treated by a mixture of independent and constrained refinement |
|  | *w* = 1/[σ^2^(*F*_o_^2^) + (0.0446*P*)^2^ + 12.0267*P*]  where *P* = (*F*_o_^2^ + 2*F*_c_^2^)/3 |
| Δρ_max_, Δρ_min_ (e Å^-3^) | 0.78, -0.73 |

Computer programs: *CrysAlis PRO* 1.171.42.49 (Rigaku OD, 2022), *SHELXS2018*/3 (Sheldrick, 2018), *SHELXL2018*/3 (Sheldrick, 2018), *SHELXTL* v6.10 (Sheldrick, 2008).

**References**:

Sheldrick, G. M. (2015). Acta Cryst. C71, 3-8.
