# Supplementary material for: A Sterically Open Ruthenium-Based Photocage Activated by Red and Far-Red Light for a Wide Range of Drugs
Source: J Am Chem Soc. 2025 Nov 18;147(48):44356–71. doi: 10.1021/jacs.5c14772 (PMC12679631; doi:10.1021/jacs.5c14772)
Supplement: Supplementary file 2 [file ja5c14772_si_002.zip › XRD/7_BF4_2.doc]

**Experimental – xs2997a**

All reflection intensities were measured at 110(2) K using a SuperNova diffractometer (equipped with Atlas detector) with Mo *K*α radiation (λ = 0.71073 Å) under the program CrysAlisPro (Version CrysAlisPro 1.171.42.49, Rigaku OD, 2022). The same program was used to refine the cell dimensions and for data reduction. The structure was solved with the program SHELXS-2018/3 (Sheldrick, 2018) and was refined on *F^2^* with SHELXL-2018/3 (Sheldrick, 2018). Numerical absorption correction based on gaussian integration over a multifaceted crystal model was performed using CrysAlisPro. The temperature of the data collection was controlled using the system Cryojet (manufactured by Oxford Instruments). The H atoms were placed at calculated positions (unless otherwise specified) using the instructions AFIX 23, AFIX 43 or AFIX 137 with isotropic displacement parameters having values 1.2 or 1.5 *U*_eq_ of the attached C or N atoms. The H atoms attached to N9, N10, O1W and O2W were found from difference Fourier maps, and their coordinates were refined pseudofreely using the DFIX instructions in order to keep the N−H, O−H bond and H…H distances within some acceptable ranges.

The structure is party disordered. The moiety Ru1→C25 and the two BF_4_^−^ counterions are disordered over either two or three orientations, and all occupancy factors of the major / minor components of the disorder can be retrieved from the final .cif file. The asymmetric unit also contains two lattice water solvent molecules (O1W is fully occupied but O2W is only partially occupied, and the value of the occupancy factor refines to 0.624(11).

**Table 1. Experimental details**

|  | xs2997a |
| --- | --- |
| Crystal data | |
| Chemical formula | C_48_H_44_N_10_O_3_RuS·2(BF_4_)·1.624(H_2_O) |
| *M*_r_ | 1144.88 |
| Crystal system, space group | Triclinic, *P*-1 |
| Temperature (K) | 110 |
| *a*, *b*, *c* (Å) | 13.0211 (4), 13.4609 (5), 14.2536 (2) |
| α, β, γ (°) | 80.732 (2), 82.5504 (19), 82.385 (2) |
| *V* (Å^3^) | 2428.93 (12) |
| *Z* | 2 |
| Radiation type | Mo *K*α |
| μ (mm^-1^) | 0.46 |
| Crystal size (mm) | 0.33 × 0.24 × 0.20 |
|  | |
| Data collection | |
| Diffractometer | SuperNova, Dual, Cu at zero, Atlas |
| Absorption correction | Gaussian  *CrysAlis PRO* 1.171.42.49 (Rigaku Oxford Diffraction, 2022) Numerical absorption correction based on gaussian integration over a multifaceted crystal model Empirical absorption correction using spherical harmonics, implemented in SCALE3 ABSPACK scaling algorithm. |
| *T*_min_, *T*_max_ | 0.465, 1.000 |
| No. of measured, independent and  observed [*I* > 2σ(*I*)] reflections | 51058, 9543, 8850 |
| *R*_int_ | 0.037 |
| (sin θ/λ)_max_ (Å^-1^) | 0.617 |
|  | |
| Refinement | |
| *R*[*F*^2^ > 2σ(*F*^2^)], *wR*(*F*^2^), *S* | 0.047, 0.110, 1.20 |
| No. of reflections | 9543 |
| No. of parameters | 1123 |
| No. of restraints | 1935 |
| H-atom treatment | H atoms treated by a mixture of independent and constrained refinement |
| Δρ_max_, Δρ_min_ (e Å^-3^) | 0.93, -0.62 |

Computer programs: *CrysAlis PRO* 1.171.42.49 (Rigaku OD, 2022), *SHELXS2018*/3 (Sheldrick, 2018), *SHELXL2018*/3 (Sheldrick, 2018), *SHELXTL* v6.10 (Sheldrick, 2008).

**References**:

Sheldrick, G. M. (2015). Acta Cryst. C71, 3-8.
